# Integrated Analysis of Large-Scale Omics Data Revealed Relationship Between Tissue Specificity and Evolutionary Dynamics of Small RNAs in Maize (*Zea mays*)

**DOI:** 10.3389/fgene.2020.00051

**Published:** 2020-02-11

**Authors:** Yu Xu, Ting Zhang, Yuchen Li, Zhenyan Miao

**Affiliations:** ^1^State Key Laboratory of Crop Stress Biology for Arid Areas, College of Life Sciences, Northwest A&F University, Yangling, China; ^2^Center of Bioinformatics, College of Life Sciences, Northwest A&F University, Yangling, China

**Keywords:** maize, microRNA, phasiRNAs, tissue specificity, genome duplication

## Abstract

The evolutionary dynamics and tissue specificity of protein-coding genes are well documented in plants. However, the evolutionary consequences of small RNAs (sRNAs) on tissue-specific functions remain poorly understood. Here, we performed integrated analysis of 195 deeply sequenced sRNA libraries of maize B73, representing more than 10 tissues, and identified a comprehensive list of 419 maize microRNA (miRNA) genes, 271 of which were newly discovered in this study. We further characterized the evolutionary dynamics and tissue specificity of miRNA genes and corresponding miRNA isoforms (isomiRs). Our analysis revealed that tissue specificity of isomiR events tends to be associated with miRNA gene abundance and suggested that the frequencies of isomiR types are affected by the local genomic regions. Moreover, genome duplication (GD) events have dramatic effect on evolutionary dynamics of maize miRNA genes, and the abundance divergence for tissue-specific miRNA genes is associated with GD events. Further study indicated that duplicate miRNA genes with tissue-specific expression patterns, such as *miR2275a*, a phased siRNA (phasiRNA) trigger, contribute to phenotypic traits in maize. Additionally, our study revealed the expression preference of 21- and 24-nt phasiRNAs in relation to tissue specificity. This large-scale sRNAomic study depicted evolutionary implications of tissue-specific maize sRNAs, which coordinate genome duplication, isomiR modification, phenotypic traits and phasiRNAs differentiation.

## Introduction

Plants are multicellular eukaryotes with diverse tissues comprising various cell types, which carry out common processes essential for survival. However, within the physical context of the tissue environment, cells also exhibit unique functions that help define tissue-specific phenotypes (Edwards and Coruzzi, 1990). These common and tissue-specific processes are ultimately controlled by gene regulatory networks that alter the extent of gene expression. The tissue specificity of these processes is often described based on the expression level of protein-coding genes (PCGs). These analyses only partially capture the variety of processes that distinguish different tissues, due to the ignorance of other regulatory elements, such as small RNAs (sRNAs). Plant sRNAs include microRNAs (miRNAs) and small interfering RNAs (siRNAs), which are typically 20–24 nucleotides (nt) in length. These sRNAs regulate the expression of PCGs involved in a variety of biological processes associated with growth, development, and stress responses in plants (Meyers et al., 2008). MiRNAs also play roles in triggering the biogenesis of secondary siRNAs, termed epigenetically activated siRNAs (easiRNAs), *trans*-acting siRNAs (ta-siRNAs) or phased siRNAs (phasiRNAs) (Allen et al., 2005; Creasey et al., 2014). As miRNAs, phasiRNAs function in a homology-dependent manner to suppress the expression of their targets (Fei et al., 2013). The number of phasiRNA-generating loci (*PHAS* loci) varies substantially among species, ranging from over 800 in wild rice (*Oryza rufipogon*) (Liu et al., 2013b) to less than 30 in *Arabidopsis thaliana* (Columbia-0 ecotype) (Fei et al., 2013). Therefore, the investigation of tissue-specific miRNAs and phasiRNAs will help to understand the regulatory mechanisms of tissue specific phenotypes, thereby facilitating crop improvement via genetic engineering and precision breeding.

Maize (*Zea mays*) is not only one of the most economically important crops but also a model monocot plant for genetic and genomic studies. A growing body of molecular genetics and transgenic studies support the role of sRNAs in regulating diverse agronomic traits in maize (Li et al., 2017; Tang and Chu, 2017). However, the large-scale functional characterization of sRNAs is still considerably lagging in maize compared with the progress made in the model species *Arabidopsis*. In the field of bioinformatics and computational biology, there are still plenty of obstacles to further progress. One of those is the deficiency of comprehensive and high-quality annotation of miRNA genes in maize. To date, only 202 distinct mature miRNAs encoded by 169 miRNA genes in maize have been reported in miRBase (release 22) (Kozomara and Griffiths-Jones, 2014). The number of annotated miRNAs in maize is unexpectedly lower than that in other model plants, such as *Arabidopsis* (349 miRNA genes), soybean (*Glycine max*; 482 miRNA genes), and rice (553 miRNA genes) reported in miRBase. Currently, more than 100 maize sRNA-seq datasets are available in the National Center for Biotechnology Information (NCBI) database. These sRNA-seq datasets are of great value in investigating the expression profiles and potential function of miRNAs. However, these datasets were generated from different laboratories worldwide, which have different sequencing qualities and represent miRNAs in different tissues. Given that the incomplete maize reference genome and impropriate approaches used to identify maize miRNAs ranging from prediction to validation of target cleavage (Jiao et al., 2017; Axtell and Meyers, 2018), it is inevitable that some miRNAs remain to be discovered. Thus, an integrated analysis of sRNA-seq datasets will be very helpful in enriching the set of annotated maize miRNAs, exploring tissue-specific miRNA genes, and uncovering the expression of different miRNA variants.

The complexity of maize genome is another major obstacle. Maize was domesticated from its wild progenitor species, teosinte (*Zea mexiccana*), ~10,000 years ago (Doebley, 2004). It has been proposed that the *Zea* lineage has undergone several rounds of genomic duplication (GDs) events, such as whole genome duplications (WGDs), large-scale segmental duplications (SDs), and tandem duplications (TDs), after its divergence from the lineage that gave rise to sorghum (*Sorghum bicolor*) ~5–12 million years ago (Schnable et al., 2011). GDs are a common phenomenon in plant genome evolution, resulting in the expansion and diversification of many gene families. Post-duplication, many genes have been retained in the genome as paralogous pairs, and individual genes comprising the pair have been either sub-functionalized (partitioning and sharing the original gene function) and/or neo-functionalized (gaining novel functions) via sequence and/or expression divergence (Freeling, 2009). Studies in different species have revealed that ancient miRNA gene families have evolved via several of GD events (Baldrich et al., 2018). For example, the miR166 family contains seven members in *Arabidopsis*. These miRNA members resulted from WGD, SD, and TD, followed by tissue-specific sub-functionalization, as well as the deletion of one of the members (Maher et al., 2006). Similarly in soybean, post-WGD, the majority of miRNA gene singletons originated from rapid decay, and the retained miRNA gene duplicates evolved slower than singletons, regardless of whether they originated from WGD or TDs (Zhao et al., 2015). With the availability of the maize B73 reference genome sequence and genome resequencing data from dozens of maize accessions, this gramineous crop was made more accessible and has become an important model system suitable for investigation of the evolutionary dynamics and consequences of recurrent GD events.

In this study, we attempted to investigate evolutionary implications of tissue-specific sRNA genes in maize. To achieve this, we broadly collected 195 maize sRNA-seq datasets across 14 tissues and performed an integrated analysis to build a comprehensive list of maize miRNA and phasiRNA genes. A genome-wide study on maize miRNA genes was performed to examine isomiR modification, tissue specificity, and duplication status. We also investigated agronomic traits associated with tissue-specific miRNA genes and selected by artificial improvement. Furthermore, we dissected differentiation of phasiRNAs in relation to tissue specificity.

## Materials and Methods

### Libraries Construction for High-Throughput Sequencing

Maize B73 inbred lines were grown in pots under a controlled growth chamber (28 °C day / 26 °C night, 14 h light / 10 h dark). Well-watered (WW) seedlings were ensured a normal water supply with 80% of soil field moisture capacity. The drought-stressed (DS) seedlings were subjected to progressive stress by withholding water, and the soil relative water content were maintained at 40% of soil field moisture capacity. The two experimental samples (three biological replicates for each) were collected when three fully expanded leaves appeared, and immediately frozen in liquid nitrogen, and stored at −80 °C for RNA extraction.

Total RNA for small RNA sequencing was extracted using TRIzol reagent (Invitrogen, Carlsbad, USA). Total RNA was separated through 17% denaturing polyacrylamide gels and small RNAs between 10- and 60-nt were collected. Then, 5′ and 3′ RNA adaptors were ligated to small RNAs and followed by reverse transcription to produce cDNAs. These cDNAs were subsequently amplified by PCR and subjected to Illumina sequencing by Novogene Company (http://www.novogene.com/).

### Computational Analysis of sRNA-Seq Data and miRNA Annotation

A total of 195 maize sRNA-seq libraries (189 downloaded from NCBI database and six generated in this study) were examined in this study (**Supplementary Table 1**). These libraries were constructed from sRNAs extracted from different tissues, including seedlings, leaves, ears, silks, tassels, anthers, pollen, seeds, embryos, endosperm, stalk, shoot apical meristem, early-prophase meiocytes, and roots at different developmental stages. To detect miRNAs, sRNA-seq datasets were analyzed using a bioinformatics pipeline referring to the recently updated pipeline (Axtell and Meyers, 2018), with slight modifications for the maize genome. Raw miRNA reads were normalized to transcripts per million (TPM) by multiplying a factor of 1,000,000 divided by the total number of mapped reads. For samples with two or more replications, final TPM values were means of all replications in corresponding samples. One mismatch between the genome and sRNA sequence reads was allowed, and structural RNAs (transfer RNAs, ribosomal RNAs, small nuclear RNAs, and small nucleolar RNAs), low abundance RNAs (total abundance ≤100 TPM and abundance in at least one library ≤10 TPM), RNA with irregular sizes (retaining 18–26 nt), and highly repetitive sRNAs (hit number on genome >20) were removed. The modified script of miREAP software (Jeong et al., 2011) was used to predict miRNA precursors and potential pairing of miRNA and miRNA*, and miRNA precursors longer than 300 nt were discarded. For each miRNA precursor, the most abundant miRNA was regarded as canonical miRNA. Variants of canonical miRNAs (isomiRs) were identified using Jasmine pipeline (Zhong et al., 2019) and classified into 5′ isomiR, 3′ isomiR, and polymorphic isomiRs. To distinguish between miRNAs and possible siRNAs, precursor sequences with strong bias (strand bias ≥0.9 and abundance bias ≥0.75) were selected, and stem-loop structures were predicted using CentroidFold (Sato et al., 2009). Strand bias was calculated by dividing the number of reads from the sense strand with the sum of the number of reads from both strands. Abundance bias was calculated as the ratio of miRNA, miRNA* and their isomiRs to the number of reads from the sense strand. Precursor sequences that do not have a typical stem-loop structure (mismatch between the miRNA and miRNA* no more than 5 and asymmetrical bulge number no more than 3) were removed. Identified miRNA precursors were divided into previously annotated precursors and novel precursors by BLAST searching against maize precursors in miRBase v22 (http://www.mirbase.org/ftp.shtml). Maize PCGs and transposable elements (TEs) were downloaded from Gramene database (http://ensembl.gramene.org/Zea_mays).

### Identification of Differentially Expressed miRNAs

Raw miRNA reads were normalized to TPM by multiplying a factor of 1,000,000 divided by the total number of mapped reads. Final TPM values were means of three replications. The significance level of differential expression (*P*-value) was determined by the following equation (Audic and Claverie, 1997):

$$P(x|y)\;=\;(\frac{N_{2}}{N_{1}})\frac{\left(x+y\right)!}{x!y!(1+\frac{N_{2}}{N_{1}})x+y+1}$$

In this equation, *N*_1_ and *N*_2_ were replaced with 1,000,000; *x* and *y* represent TPM values under well-watered and drought-stressed conditions, respectively.

### Identification of Tissue-Specific miRNA Genes

112 of 195 sRNA-seq libraries representing 14 specific tissues and non-stressed conditions were used to define tissue-specific miRNA genes. Shannon entropy and *Z* score methods were combined to identify miRNA genes specifically expressed in a single tissue. Shannon entropy is used to measure the concentration ratio of gene expression levels in different samples and *Z* score is used to detect outliers. Considering an expression vector *x* for a miRNA gene, expression vectors *x_1_*, *x_2_*, ..., *x_n_* for *n* tissues, and an observation *x_i_* for tissue *I*, the entropy (*H*) of miRNA genes was calculated as:

$$H=-\sum_{i=1}^{n}E_{i}log_{2}E_{i}$$

where, *E_i_* is the relative expression of miRNA *x* for tissue *i* and is defined as:

$$E_{i}=x_{i}/\sum_{i=1}^{n}x_{i}.$$

modH is calculated after using a one-step Tukey's biweight to improve robustness of the expression data as described previously (Kadota et al., 2006). The *Z* score is calculated as:

$$Z_{i}=(x_{i}-\mu )/\sigma$$

where, *µ* is the average expression of miRNA genes *x_1_*, *x_2_*, ..., *x_n_* in all tissues, and *σ* is the standard deviation. The quantity of the *Z* score represents the distance between the raw expression and average expression. For tissue-specific miRNAs, we used Shannon entropy modH <1.8 and the maximum *Z* score (*Z*max) >3.

### Identification of *PHAS* Loci

The PHASIS software (minDepth was set as 3) (Kakrana et al., 2017) was used to identify *PHAS* loci from 195 sRNA-seq datasets. Candidate *PHAS* loci were selected using *P* < 1 × 10^−5^. The summarization of *PHAS* loci was generated using *phasmerge* component. The triggers were identified using *phastrigs* component. Recent studies have shown that preference for production of easiRNAs versus other secondary siRNAs (which include phasiRNAs) may in part be the result of mono-uridylation of 22-nt miRNAs such as miR170 and miR171a which triggers the production of easiRNAs (Zhai et al., 2013; Tu et al., 2015). The defining characteristic that sets easiRNAs apart from other secondary siRNAs is that they arise from transcriptionally active retrotransposons and function in the RNA-directed DNA methylation pathway (Creasey et al., 2014). Therefore, the *PHAS* loci originated from retrotransposons and triggered by 22-nt miR170 and miR171a were excluded from our analyses.

### Chromosomal Location and Duplication Analysis of miRNA Genes

The chromosomal distribution of miRNA genes was visualized using MapChart software (Voorrips, 2002). Syntenic blocks were analyzed with the CoGe Synmap program using ZmB73 gramene v4.36 masked coding sequence with default parameters (https://genomevolution.org/coge/SynMap.pl). The miRNA genes with no more than two mismatches with the genome were considered as conserved. *PHAS* loci with the same miRNA triggers and at least two pairs of similar phasiRNAs were considered as conserved. Conserved sequence pairs belonging to syntenic regions were defined as syntenic duplication. Conserved sequences with less than 200 kb between them were considered as tandem duplicates (Holub, 2001).

### Identification of miRNA Genes Potentially Related to Agronomic Traits

Datasets from genome-wide association study (GWAS) of 35 agronomic traits in 10 different populations were downloaded (**Supplementary Table 2**) and analyzed using the compressed mixed linear model with the R package GAPIT (Lipka et al., 2012). For each population, single nucleotide polymorphisms (SNPs) were filtered using linkage disequilibrium-based variant pruner implemented in PLINK (Purcell et al., 2007), with the parameters as “--indep-pairwise 1000 100 0.2 --geno 0.1 --maf 0.05”. The parameters “--geno” and “--maf” were used for controlling missing rate and minor allele frequency (MAF), respectively. In addition, the first three principal components (“Q” matrix) were also used to control population structure. All SNPs with *P* < 1 × 10^−5^ were considered as trait-related SNPs.

### Expression Analysis of the miRNA Using Stem-Loop qRT-PCR

Maize B73 plants were cultivated in a phytotron at 25 °C with 65% relative humidity under a 14-h/10-h light/dark cycle. Tissues at the V5 stage (leaves and roots), R1 stage (tassels, ears, anther, pollen, and silks), and 20 days after pollination (DAP) stage (seeds, embryos, and endosperms) were collected and immediately frozen in liquid nitrogen. Samples from at least five plants were pooled for each biological replicate, and three biological replicates were performed. Total RNA was extracted following above described methods. To confirm the miRNA expression data, stem-loop qRT-PCR was performed referring to previously described methods (Chen et al., 2005). Typically, 200ng of RNA was digested by DNase I (Thermo scientific, USA) and reverse transcribed using a TaKaRa Mir-X miRNA First-Strand Synthesis Kit (Cat. No.638313). The stem-loop RT-PCR was performed on the ABI7500 Real-Time System (Applied Biosystems, USA) using EvaGreen 2 × qPCR MasterMix-ROX (Abm, Canada). The reactions were incubated in a 96-well plate at 55 °C for 2 min, 95 °C for 10 min, followed by 40 cycles of 95 °C for 30 s and 60 °C for 1 min. All reactions were assayed in three biological replicates, and 18S rRNA was used as the internal control for stem-loop RT-PCR. Normalized expression levels were calculated as previously described methods (Schmittgen and Livak, 2008). Primers used in the qRT-PCR are listed in **Supplementary Table 3**.

### Identification of miRNA Target

Potential target transcripts of miRNAs were predicted using psRNATarget (2017 update; http://plantgrn.noble.org/psRNATarget) (Dai and Zhao, 2011), psRobot (v1.2; http://omicslab.genetics.ac.cn/psRobot/downloads.php) (Wu et al., 2012), and TargetFinder (https://github.com/carringtonlab/TargetFinder) (Fahlgren and Carrington, 2010) with default parameters, except for the maximum expectation of five in psRNATarget. Predicted sRNA targets were verified with degradome sequencing data of maize B73. The raw degradome sequencing datasets were downloaded from the NCBI's Sequence Read Archive (SRA) database (SRR768486, SRR768488, SRR768490, SRR768493, SRR895786, SRR895787, SRR895788, SRR895789, SRR1028862, SRR1028864, SRR1028865, SRR1028866, SRR2917866, SRR4183498, SRR4183499, SRR3347501, SRR3348073, SRR3470769, and SRR3470770). Raw reads were preprocessed using the FASTX toolkit (http://hannonlab.cshl.edu/fastx_toolkit) to remove adaptors, low-quality bases (score <20), and short reads (<18 nt). Clean reads and maize cDNA sequences were input into CleaveLand4 (setting: -p 0.05) (Addo-Quaye et al., 2009) to identify potential cleavage sites.

### Statistical Analysis

The Student's *t*-test was performed using *t.test* function in R package. The χ^2^ test was performed using *chisq.test* function in R package.

## Results

### Genome-Wide Identification of Maize miRNA Genes and Their Mature Sequences

**Figure 1:**
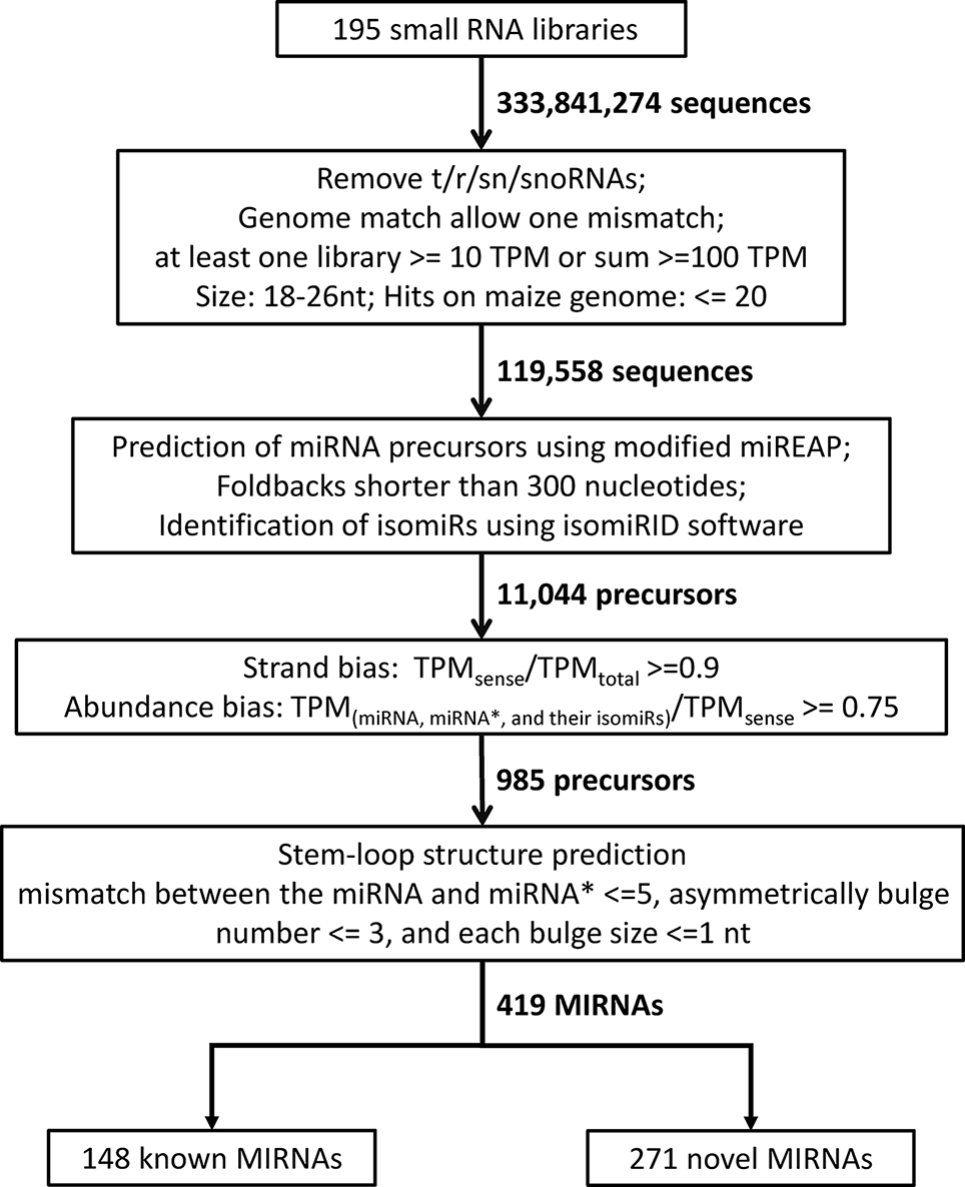
Bioinformatics pipeline for the identification of miRNA genes in maize.

**Figure 2:**
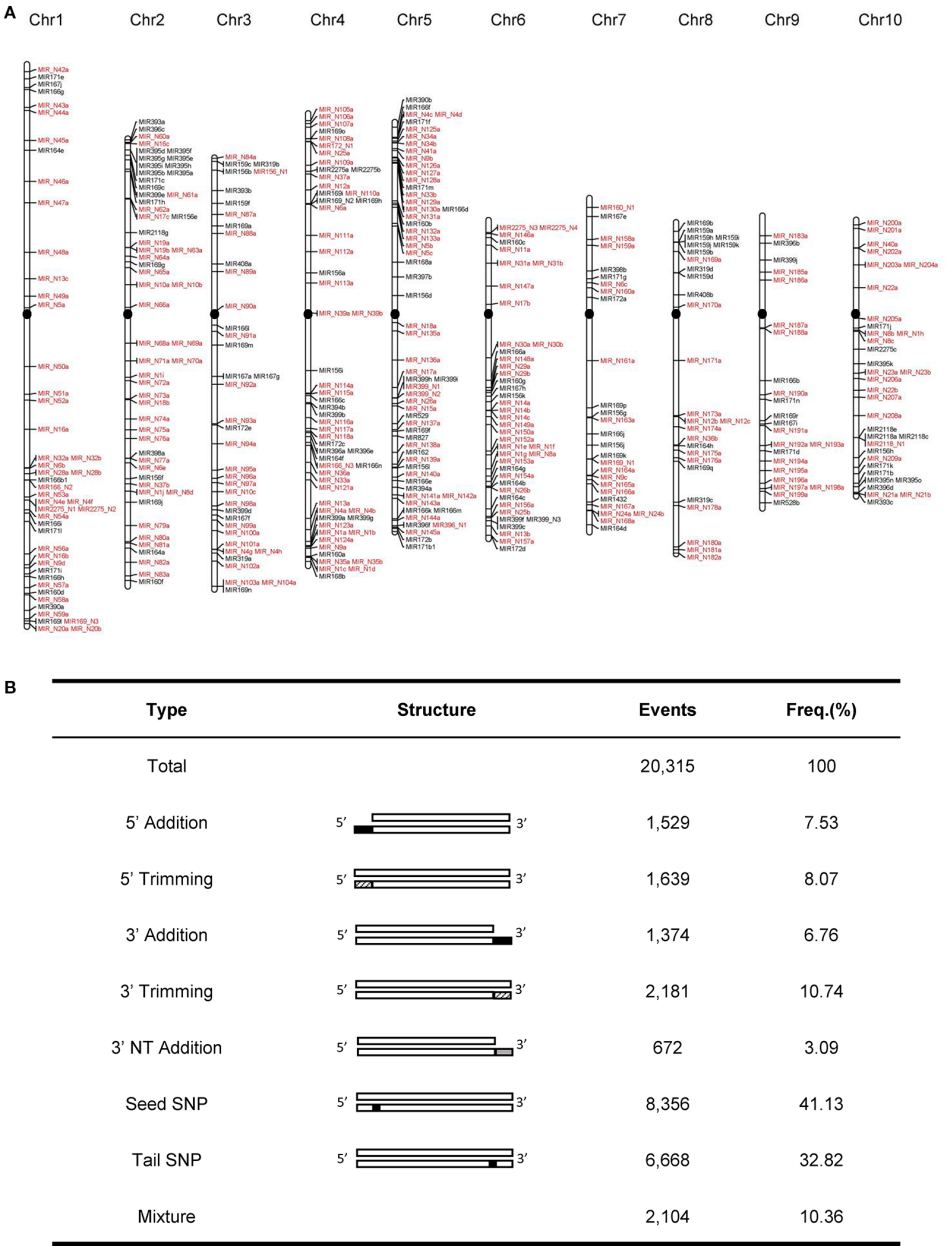
Genome-wide characteristics of maize miRNA genes. **(A)** Chromosomal distribution of 419 maize miRNA genes. Newly identified miRNA genes were labeled as red. The black filled circles represent centromeres **(B)** Statistics of different isomiR types in the maize genome.

In this study, we developed a bioinformatics pipeline to identify maize miRNAs and their mature sequences referring to the recently updated pipeline (Axtell and Meyers, 2018) (**Figure 1**). This miRNA identification pipeline was applied to process ~3.83 billion sequencing reads from 195 maize sRNA-seq datasets covering diverse tissues and conditions (**Supplementary Table 1**). As a result, we identified a total of 419 maize miRNA genes (**Supplementary Table 4**), consisting of 148 annotated **miRNA** genes in miRBase v22, as well as 271 miRNA genes newly discovered in this study. Like the miRNA genes present in miRBase, these novel miRNA genes mapped throughput the maize B73 reference genome with an uneven distribution on 10 chromosomes, and most of these genes were in regions of chromosome arms (**Figure 2A**). The sequence structures of 419 maize miRNA genes were visualized in Supplementary Data 1. DICER-LIKE1 (DCL1) is one of central components of the plant miRNA biogenesis pathway. By analyzing previously published *dcl1* mutants sequencing data (Petsch et al., 2015), we found the expression levels of both known and novel miRNAs were reduced in mutation samples (**Supplementary Figure 1**). Of the 419 miRNA genes, 125 (29.8%) were located within PCGs, 125 (29.8%) were surrounded by TEs, and 196 (46.8%) were located within unclassified intergenic sequences (UIs; **Supplementary Table 5**).

Growing evidence suggested that a single miRNA gene can generate multiple mature isomiRs that differ in their length and/or sequence composition (Zhai et al., 2013). From the 419 miRNA genes, we identified 20,315 isomiRs, which can be categorized into seven classes: nucleotide substitutions within the seed sequence (seed SNP; 41.1%), nucleotide substitutions within tail sequence (tail SNP; 32.8%), 3′ trimming (10.7%), 5′ trimming (8.1%), 5′ addition (7.5%), 3′ addition (6.8%), and non-templated 3′ addition (3′ nt addition; 3.1%) (**Figure 2B**). Biased nucleotide composition was observed at the ends of isomiRs. For instance, the 5′ and 3′ ends of truncated isomiRs were likely to be ‘G' and ‘C' nucleotides, respectively. By Contrast, nucleotides on the 3′ end of non-template added isomiRs were generally ‘A' and ‘U' (**Supplementary Figure 2**).

Together, the large-scale analysis of sRNA-seq datasets provides a more comprehensive profiling of maize miRNA genes and the corresponding isomiRs (**Supplementary Table 6**). An overview of the maize sRNA profiling supported by JBrowse (Buels et al., 2016) can be accessed in the Maize sRNA Data Browser, which is publicly available at http://bioinfo.nwafu.edu.cn/MSDB/index.html.

### Survey of Differential Expressed miRNAs in Maize Response to Drought Stress

To study the expression dynamics of miRNAs and their potential roles in gene expression regulation in maize drought responses, we constructed and sequenced six sRNA libraries from WW and DS maize B73 inbred lines. Of the 419 miRNAs genes, 43 differentially expressed miRNAs (DEMs) induced by drought stress were detected (**Supplementary Table 7**). To understand the potential regulatory roles of drought stress-associated miRNAs, we identified the target genes of DEMs using three bioinformatics programs applying different alignment algorithms, then further validated by using sequencing datasets from 19 publicly available PARE libraries of maize B73. Of the 43 DEMs, 24 were found to target 30 transcripts (**Supplementary Table 8**). These include 13 transcription factors, such as MYB domain-containing, NAC domain-containing, Scarecrow like, and squamosa promoter binding, as well as 17 other functional genes, such as ubiquitin, zinc ion binding, and Syg1/Pho81/XPRI.

### Ninety-Four miRNA Genes Show Highly Tissue-Specific Expression Patterns

**Figure 3:**
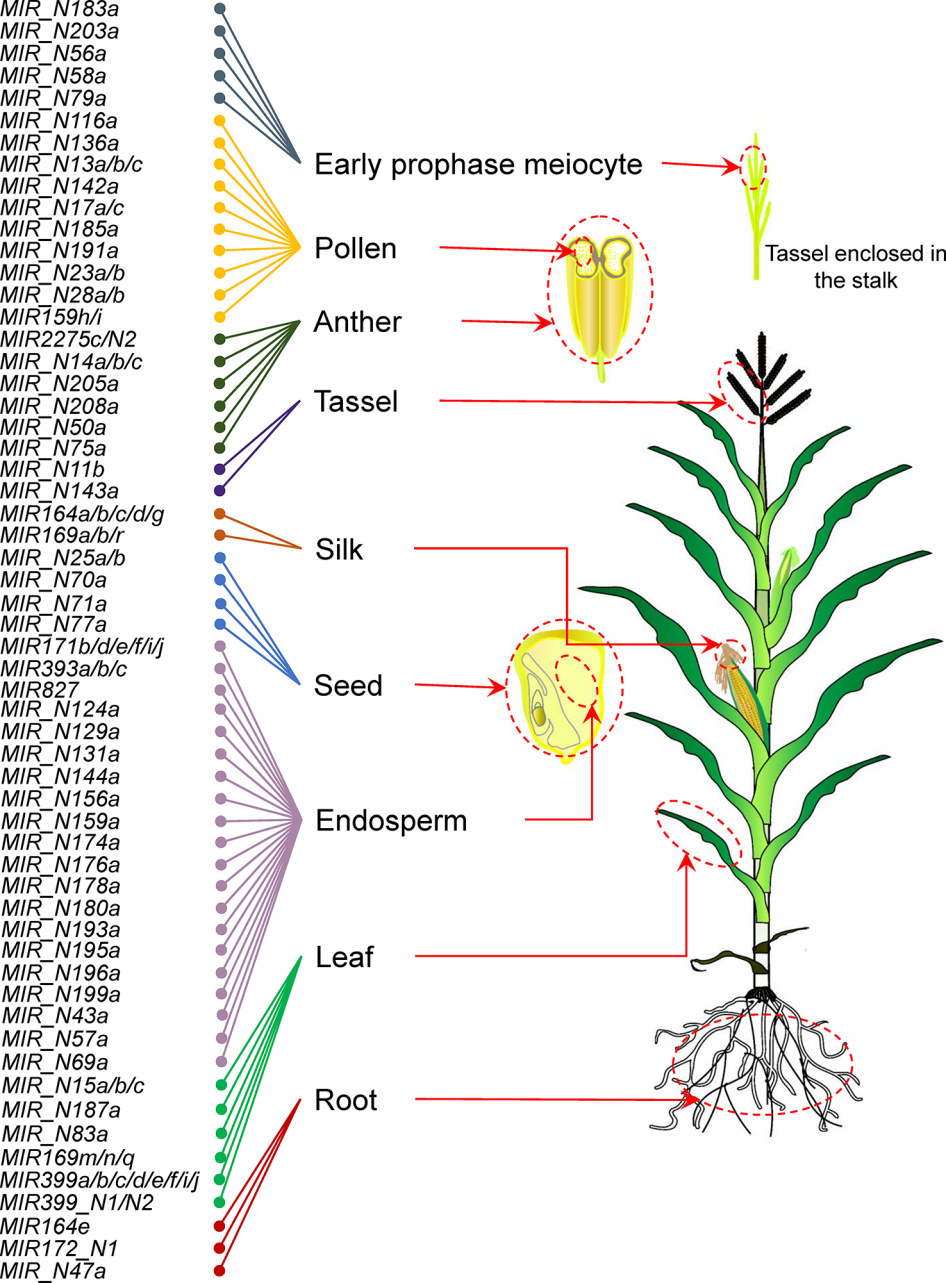
Overview of tissue-specific patterns of maize miRNA genes.

A tissue-specific miRNA gene is defined as one that is highly expressed in a specific tissue. Based on the expression profile of maize sRNAs in 14 specific tissues under normal conditions, we identified a total of 94 miRNA genes (34 previously annotated and 60 newly discovered miRNA genes) using Shannon entropy and *Z* score. These genes were specifically expressed in nine tissues (**Figure 3** and **Supplementary Figure 3**). We noticed that endosperm, pollen, and leaf tissues required relatively more specific miRNA genes than other tissues. Some miRNA gene families (e.g. *miR164* and *miR169*) were predominantly expressed in multiple tissues. For example, *miR164e* was specifically expressed in root, whereas *miR164a*, *miR164b*, *miR164c*, *miR164d*, and *miR164g* showed silk-specific expression. Similarly, *miR169m*, *miR169n*, and *miR169q* were preferentially expressed in the leaf, whereas *miR169a*, *miR169b*, and *miR169r* were preferentially expressed in the silk. Then, we performed qRT-PCR assay to further confirm the expression levels of nine miRNAs. As shown in **Supplementary Figure 4**, the results of qRT-PCR assay are consistent with those of sRNA-seq. These results indicated that tissue-specific miRNA gene family members, which differ by only a few nucleotides may have redundant or diverse functions.

### Dynamic Patterns of Isomir Events in Terms of Tissue Types and Genomic Environments

**Figure 4:**
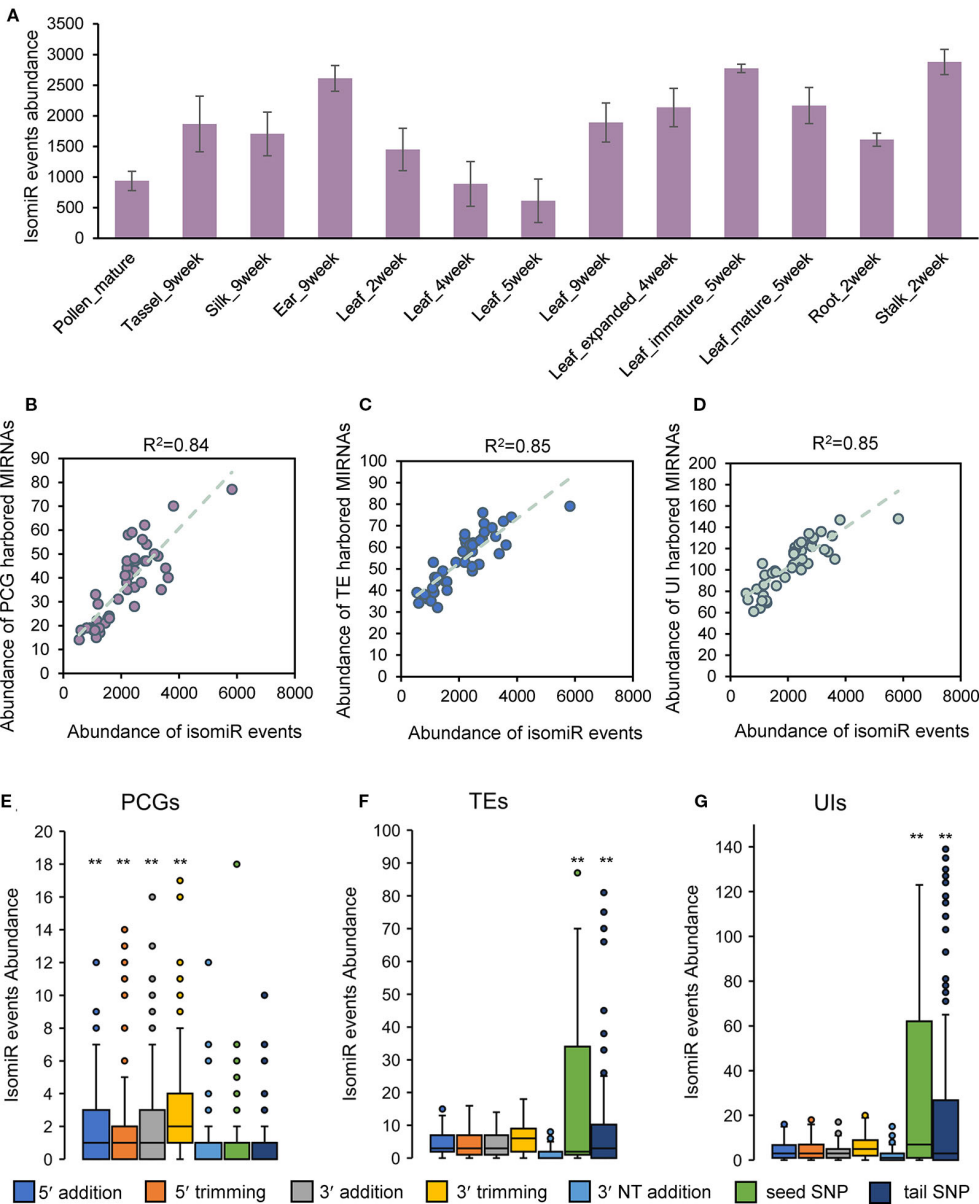
Dynamic patterns of isomiR events in terms of tissue types and genomic environments. **(A)** Abundance of isomiRs varied widely among individual tissues. **(B)** Correlation between the abundance of isomiR events and PCG-harbored miRNA genes in all tissue samples. **(C)** Correlation between the abundance of isomiR events and TE-harbored miRNA genes in all tissue samples. **(D)** Correlation between the abundance of isomiR events and UI-harbored miRNA genes in all tissue samples. **(E)** Comparisons of isomiR abundance among seven isomiR types in PCG-harbored miRNA genes. **(F)** Comparisons of isomiR abundance among seven isomiR types in TE-harbored miRNA genes. **(G)** Comparisons of isomiR abundance among seven isomiR types in UI-harbored miRNA genes. Statistically significant differences in **(E, F** and **G)** were determined using Student's *t*-test and **indicated *P-value* < 0.05.

Considering the tissue specificity of miRNA expression and regulation, we are interested that whether isomiRs exhibit distinctive properties in different tissues. Statistical analysis showed that the abundance of isomiRs varied widely among tissues (**Figure 4A**). There were more isomiRs events in the ear and stalk tissues, and the abundance of isomiRs varied during development of leaf tissue (**Figure 4A**). The composition of seven isomiR types changed remarkably among the individual tissues (**Supplementary Figure 5**). Nucleotide bias was also observed at the 5′ and 3′ ends of isomiRs among tissues (**Supplementary Figure 6**). Correlation analysis using data from all individual tissue samples showed that the abundance of isomiRs were highly correlated with the abundance of expressed miRNA genes located in PCGs, TEs, and UIs (**Figures 4B–D**). Comparisons of isomiR abundance among seven isomiR types were conducted in three categories of miRNA genes (PCGs, TEs, and UIs) separately. For miRNA genes located in PCGs, 5′ and 3′ isomiR types (5′ addition, 5′ trimming, 3′ addition and 3′ trimming) exhibited significantly higher abundance than polymorphic isomiR types (seed SNP and tail SNP; **Figure 4E**). In contrast, the abundance of 5′ and 3′ isomiR types were significantly lower for miRNA genes harbored by TEs and UIs (**Figures 4F, G**). These observations indicated that tissue specificity of isomiR events tends to be associated with miRNA gene abundance and suggested that the frequencies of isomiR types are affected by the local genomic regions.

### Evolutionary Implications of Duplicated miRNA Genes on Tissue Development

It has been proposed that maize underwent a series of GD events after its divergence from sorghum ~5–12 million years ago (Swigonova et al., 2004; Schnable et al., 2011). GDs are recognized as essential sources of gene family expansion and functional specialization (Panchy et al., 2016). Due to evolutionary dynamics of duplicated genes, one copy of many duplicated gene pairs in maize was eliminated, leaving the other one as a singleton after the genomic duplication events. Therefore, the singleton-to-duplicate ratio represents the degree of fragmentation of duplicated genomes. To understand the evolutionary process and consequences of tissue-specific miRNA genes after GD, based on the newly annotated set of PCGs in B73 reference genome (Jiao et al., 2017), we reanalyzed the large GDs encompassing the 419 miRNA genes and found that these genes can be classified into 305 singletons and 114 duplicates, with a ratio of 2.68:1 (**Table 1**). For miRNAs located in PCGs, TEs, and UIs, the corresponding singleton-to-duplicate ratio were 8.62:1, 2.79:1, and 1.76:1, respectively (**Table 1**). The chi-square "goodness-of-fit" test showed that the singleton-to-duplicate ratios for miRNA genes in PCGs and UIs were significantly different from that for miRNA genes in the whole maize genome (**Table 1**). In addition, the average abundance of miRNA genes in PCGs was the lowest compared with those in TEs and UIs, and the genome-wide miRNA duplicates showed relatively higher expression than singletons (**Supplementary Table 9**). These analyses suggested that the duplication status of miRNA genes was affected by their locations in the genome.

**Table 1:** Statistical analysis of singleton-to-duplicate ratios of miRNA genes located in three categories of genomic components.

	Singleton	Duplication	Ratio (S/D)^a^	*P*^b^
**Total**	305	114	2.68:1	
**PCG**	112	13	8.62:1	9.68 × 10^−5^
**TE**	92	33	2.79:1	0.858
**UI**	125	71	1.76:1	0.023

a The ratio of singletons to duplicates.

b The statistical analysis was conducted on the ratios of singletons to duplicates between total miRNAs and miRNAs in each category of genomic components by χ^2^ goodness of fit test.

For the 94 tissue-specific miRNA genes, 72 were singletons and 22 were duplicates, corresponding to a singleton-to-duplicate ratio of 3.27:1, which was significantly higher than that in the whole maize genome (2.68:1; χ^2^ test, *P* < 0.05). This indicated an overrepresentation of singletons compared with duplicates in tissue-specific miRNA genes. To further investigate if and how GD contributed to the tissue specificity in maize, we compared the relative proportions of miRNA singletons and duplicates expressed in individual tissue samples. The ratios of expressed miRNA singletons to duplicates (ranging from 0.82:1 to 2.28:1) in all tissues, except endosperm, were significantly lower than that in the whole maize genome (χ^2^ test, *P* < 0.05) (**Table 2**). With leaf development, the ratio of expressed miRNA singletons to duplicates decreased after 2 weeks after germination and then increased after 6 weeks, however, the opposite trend was observed during silk development. Moreover, the singleton-to-duplicate ratio decreased during ear development, and the similar trend was also observed in the tassel (**Table 2**). We also compared the ratios of expressed miRNA singletons to duplicates located in the three categories of genomic compartments in each individual tissue sample. In general, among the three categories of miRNA genes, the singleton-to-duplicate ratio was the lowest in UIs and the highest in PCGs in most tissues (**Supplementary Table 10**). Collectively, these observations revealed extensive divergence in the duplication status of miRNA genes among tissues.

**Table 2:** Statistical analysis of singleton-to-duplicate ratios of miRNA genes in individual tissue samples.

	Singleton	Duplicate	Ratio (S/D)^a^	*P*^b^
**Total**	305	114	2.68:1	
**Anther**	142	78	1.82:1	0.031
**Ear_6week**	72	64	1.12:1	1.64 × 10^−5^
**Ear_8week**	74	68	1.09:1	5.39 × 10^−6^
**Ear_9week**	72	74	0.97:1	2.17 × 10^−7^
**Ear_10week**	55	54	1.02:1	8.21 × 10^−6^
**Early prophase meiocytes**	131	85	1.54:1	3.91 × 10^−3^
**Embryo_1d**	87	63	1.38:1	7.84 × 10^−4^
**Embryo_3week**	57	46	1.24:1	5.78 × 10^−4^
**Embryo_9DAP**	85	73	1.16:1	1.38 × 10^−5^
**Embryo_15DAP**	89	66	1.35:1	4.24 × 10^−4^
**Embryo_20DAP**	67	61	1.10:1	1.42 × 10^−5^
**Endosperm_9DAP**	146	68	2.15:1	0.230
**Endosperm_15DAP**	155	70	2.21:1	0.296
**Endosperm_20DAP**	157	69	2.28:1	0.372
**Leaf1_2week**	126	74	1.70:1	1.32 × 10^−2^
**Leaf2_2week**	102	83	1.23:1	1.98 × 10^−5^
**Leaf1_3week**	122	71	1.72:1	1.65 × 10^−2^
**Leaf2_3week**	101	74	1.36:1	3.16 × 10^−4^
**Leaf3_3week**	92	76	1.21:1	2.44 × 10^−5^
**Leaf_4week**	88	85	1.04:1	2.81 × 10^−7^
**Leaf_5week**	77	82	0.94:1	3.29 × 10^−8^
**Leaf_6week**	112	67	1.67:1	1.27 × 10^−2^
**Leaf_9week**	116	85	1.36:1	1.67 × 10^−4^
**Leaf_expanded_4week**	135	89	1.52:1	1.13 × 10^−3^
**Leaf_immature_5week**	120	84	1.43:1	4.41 × 10^−4^
**Leaf_mature_5week**	138	85	1.62:1	4.43 × 10^−3^
**Leaf_wrapped_4week**	116	87	1.33:1	9.11 × 10^−5^
**Pollen_early**	89	66	1.35:1	4.24 × 10^−4^
**Pollen_germination**	54	45	1.20:1	4.00 × 10^−4^
**Pollen1_mature**	70	49	1.43:1	3.43 × 10^−3^
**Pollen2_mature**	84	46	1.83:1	4.31 × 10^−2^
**Root1_2week**	96	79	1.22:1	2.09 × 10^−5^
**Root2_2week**	112	66	1.70:1	1.62 × 10^−2^
**Seed**	123	68	1.81:1	3.56 × 10^−2^
**Shoot apical meristem_4week**	81	67	1.21:1	5.08 × 10^−5^
**Stalk_2week**	103	81	1.27:1	4.81 × 10^−5^
**Silk_9week**	64	78	0.82:1	1.77 × 10^−9^
**Silk_11week**	115	84	1.37:1	1.88 × 10^−4^
**Silk_12week**	101	89	1.13:1	1.92 × 10^−6^
**Tassel_4week**	101	76	1.33:1	1.66 × 10^−4^
**Tassel_8week**	65	58	1.12:1	2.93 × 10^−5^
**Tassel_9week**	100	91	1.10:1	7.21 × 10^−7^
**Vegetative apex_2week**	72	64	1.12:1	1.64 × 10^−5^

a Singleton-to-duplicate ratios.

b P values were calculated using the χ^2^ goodness of fit test to compare the ratios of singletons to duplicates between miRNA genes in the whole genome and those expressed in different tissues.

### Evolution of Tissue-Specific miRNA Genes Contributed to Phenotypic Traits in Maize

Maize was domesticated from its wild relative teosinte approximately 10,000 years ago and was subsequently subjected to intensive breeding efforts to improve its adaptation to modern agricultural practices, resulting in dramatic morphological and physiological modifications (Lyu, 2017). Recent studies demonstrated that SNPs that occurred at miRNA-related regions (pre-miRNAs and mature miRNAs) may affect miRNA biogenesis and function and cause serious phenotype changes (Sun et al., 2009; Hung et al., 2012; Liu et al., 2013a). Therefore, we investigated whether miRNA loci and quantitative trait loci were simultaneously selected by linkage drag, or whether miRNA loci have effects on tissue-specific traits. To address this question, we collected 10 representative maize B73 populations comprising and integrated the results of GWAS on the genetic diversity in various traits, such as ear height, leaf width, days to anthesis (DTA), days to silk production (DTS), and plant height (**Supplementary Table 2**).

**Figure 5:**
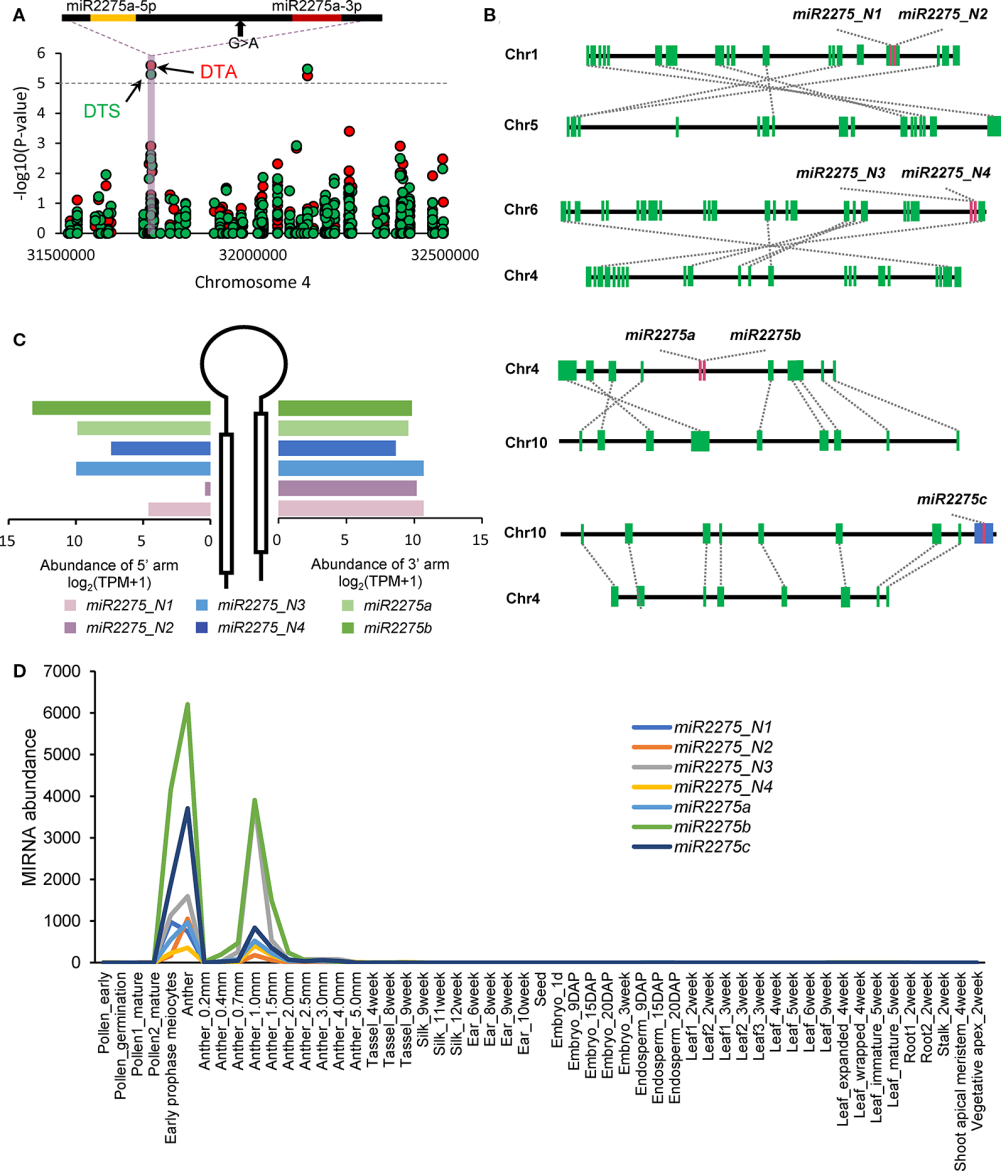
Evolutionary consequence of the *miR2275* family in maize. **(A)** GWAS results, which overlapped with *miR2275* loci. The gray horizontal dashed lines indicate the significance threshold of GWAS (1 × 10^−5^). The mature miRNA sequences are highlighted in red and the miRNA star sequences are highlighted in yellow. Black arrows indicate significant mutation sites. Days to anthesis (DTA) and days to silk production (DTS) are labeled in red and green, respectively. **(B)** Duplication status of *miR2275* family members. Green boxes indicate PCGs, red boxes represent *miR2275* gene family members, and the blue box represents helitron transposon. **(C)** Comparison of mature miRNA accumulation between 5′ and 3′ arms in individual *miR2275* members. **(D)** Accumulation levels of seven *miR2275* genes in individual tissue samples. The accumulation levels were calculated as sum of miRNA mature sequences, miRNA star sequences and isomiRs.

We detected 13 trait-associated mutation sites co-localizing with seven miRNA loci (**Supplementary Figure 7** and **Figure 5A**). Of these seven miRNA loci, four loci were associated with at least two traits. An interesting example is *miR2275a*, which belongs to the anther-specific *miR2275* family and is associated with DTA and DTS (**Figure 5A**). According to our previous analyses of duplicated miRNA loci in maize, we observed that *miR2275_N1*/*miR2275_N2*, *miR2275_N3*/*miR2275_N4*, and *miR2275a*/*miR2275b* were three homoeologous pairs formed by TD (**Figure 5B**). These results promoted us try to understand the process of selection of *miR2275* family members during evolution. Divergencies of the dominant arms were observed among the three homoeologous pairs (**Figure 5C**). Further analysis revealed that *miR2275c* co-localized with a helitron transposon, implying that TE-mediated mechanisms were responsible for the amplification of this miRNA gene family. To shed light on how genomic duplication affects the functional diversity of these homoeologous pairs, we analyzed the expression levels of seven *miR2275* loci in individual tissues. All *miR2275* loci were highly abundant in early-prophase meiocytes and anthers (**Figure 5D**). These observations, together with the association analysis, indicated that although these miRNA loci dominated in anthers, the evolutionary consequences of the *miR2275* family may have been impacted by duplication events leading to functional divergence of individual members. Subsequently, specific *miR2275* family member was evolved as causative locus for the phenotypic transition in anthers by artificial selection.

### Differentiation of 21- and 24-Nt *PHAS* Loci in Relation to Tissue Specificity

**Figure 6:**
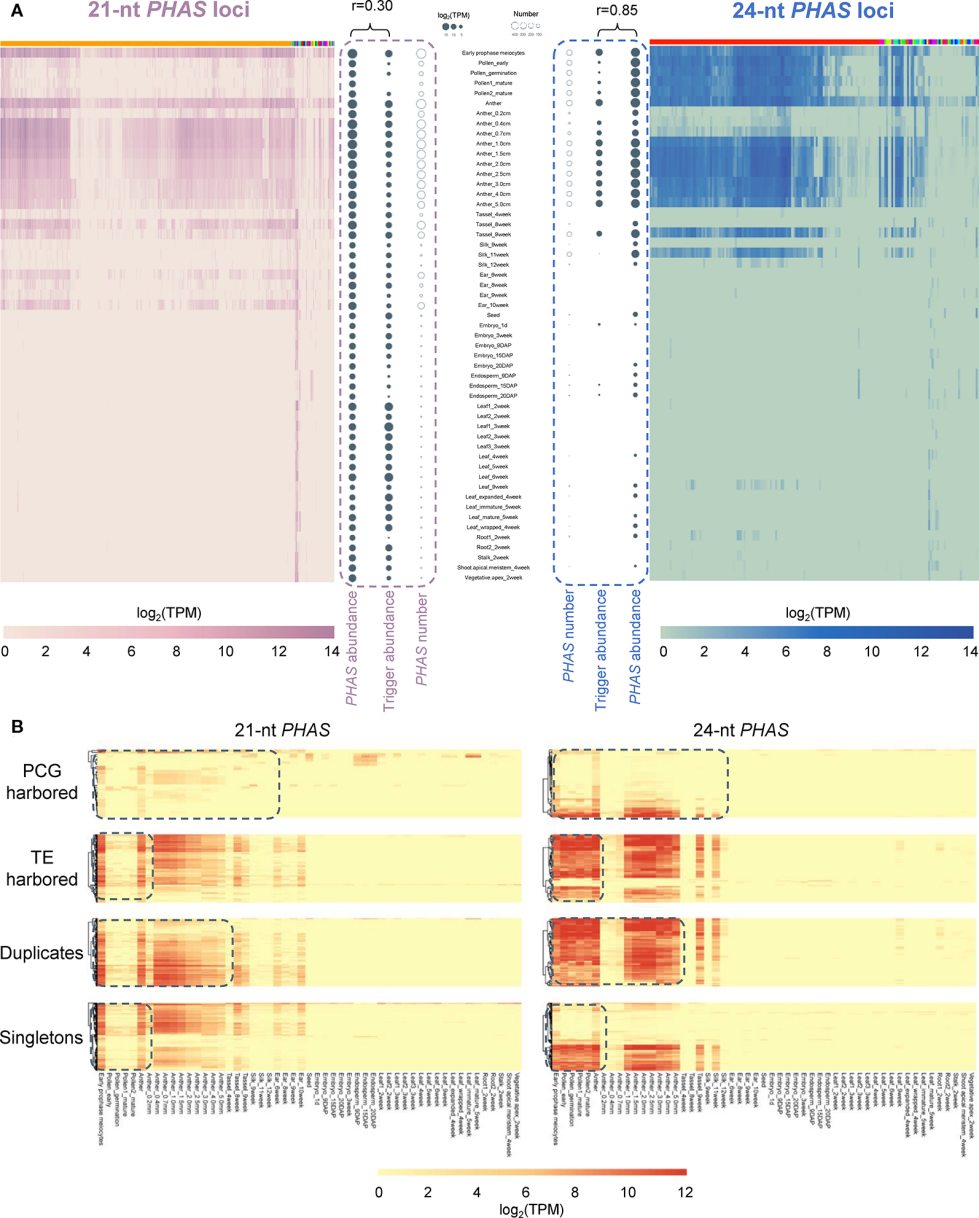
Differentiation of 21- and 24-nt *PHAS* loci in relation to tissue specificity. **(A)** Heat maps depicted the abundance of 21-nt *PHAS* loci (left) and 24-nt *PHAS* loci (right) in individual tissue samples. Solid bubbles represent the total abundance of phasiRNAs and corresponding miRNA triggers. Circles represent the number of *PHAS* loci. In the heatmaps, *PHAS* loci were clustered according to the miRNA triggers. Color bars on the top of heatmaps represent different triggers, among which orange represents miR2118 and red represents miR2275. **(B)** Heat maps depicted the abundance of 21- and 24-nt *PHAS* loci in four categories (PCG harbored, TE harbored, duplicates, and singletons). The dashed boxes indicated the divergence between the abundance of 21- and 24-nt *PHAS* loci in corresponding tissue samples.

The 22-nt mature sequences of *miR2275* family members were recognized as critical triggers for 24-nt *PHAS* loci to generate phasiRNAs (Fei et al., 2013). In addition to the *miR2275* family, there is growing evidence supporting the tissue specificity of numerous *PHAS* triggers, such as *miR156*, *miR529*, and *miR2118*. Therefore, we further examined whether the expression patterns of *PHAS* loci are tissue-specific in maize. To address this question, a genome-wide identification of *PHAS* loci was performed using sRNA-seq data collected from 14 tissues. A total of 469 21-nt and 190 24-nt *PHAS* loci were identified with high confidence (*P* ≤ 10^−5^), respectively (**Supplementary Table 11**, **Supplementary Data 2**, and Supplementary Data 3). Genome-wide identification of *PHAS* loci in maize W23 inbred line was previously performed by Zhai et al (Zhai et al., 2015). In our study, 375 21-nt *PHAS* loci and 113 24-nt *PHAS* loci were overlapped with those identified by Zhai et al, respectively (**Supplementary Table 11**). Meanwhile, 94 21-nt *PHAS* loci and 77 24-nt *PHAS* loci were novel. Integrating the results from two studies together, we found that the two classes of *PHAS* loci were highly expressed in reproductive tissues (TPM ≥5; **Figure 6A**). The number of *PHAS* loci expressed in individual tissues varied from 0 to 580, indicating tissue-specific expression (**Supplementary Table 12**). To gain insight into the contribution of tissue type on the production of phasiRNAs, the accumulations of phasiRNAs and corresponding trigger miRNAs were compared in individual tissues. The accumulations of phasiRNAs were positively correlated with the expression levels of corresponding trigger miRNAs (**Figure 6A**). The 21- and 24-nt phasiRNAs exhibited strikingly different spatiotemporal regulation. Compared with 21-nt phasiRNAs, the 24-nt phasiRNAs were preferentially expressed in pollen tissues and suppressed in early anther development (**Figure 6A**).

Then we analyzed the genomic distribution of *PHAS* loci and found that the ratio of 21-nt *PHAS* loci in TEs to those in PCGs (2.94:1) was significantly higher than that of 24-nt *PHAS* loci (1.85:1; χ^2^ test, *P* < 0.05). This observation indicated evolutionary divergence of 21- and 24-nt *PHAS* loci, which was also reflected by the distinct expression patterns of the two categories *PHAS* loci in **Figure 6B**. Although the singleton-to-duplicate ratio of 21-nt *PHAS* loci (2.34:1) was approximately consistent with that of 24-nt *PHAS* loci (2.21:1), there were obvious differences between the expression patterns of 21- and 24-nt *PHAS* loci (**Figure 6B**).

Taken together, these results suggested that divergence on local genomic environments and expression patterns occurred between 21- and 24-nt *PHAS* loci, appearing to promote the formation of intraspecific homogenization and interspecific heterogenization in terms of tissue-specific function.

## Discussion

### Comprehensive and High-Quality Annotation of miRNA Genes in Maize

It is increasingly recognized that miRNAs are key components of gene regulation, and a diversity of mechanisms of action have been proposed (Yu et al., 2017). However, the evolutionary implications of maize miRNAs on tissue specificity are still uncharacterized. Studies of miRNA genes have been frequently confounded by questionable miRNA annotations and a lack of standardized criteria for the identification of plant miRNAs from the sRNA-seq datasets. In addition, miRNA genes are highly tissue specific, making it difficult to detect accurate abundance levels, unless massive amounts of tissues are available. In our study, we addressed these shortcomings by integrating 195 deeply sequenced sRNA libraries and by surveying 14 tissues in maize. This large-scale analysis was performed based on the recently updated criteria which can simultaneously emphasize accuracy and minimize false positives in the genome-wide annotation of plant miRNAs (Axtell and Meyers, 2018; Kuang et al., 2018). To our knowledge, this collection of sRNA-seq datasets is the largest effort to date for the genome-wide identification of miRNA genes in maize.

miRNAs as gene regulators are reported to participate in the regulation of drought responsive PCGs in many plant species (Ferdous et al., 2015). For instance, *miR162* (Tian et al., 2015) and *miR164* (Fang et al., 2014) are induced by drought stress in rice. Our study confirmed this regulation that, to our knowledge, had not been reported in maize. This implied functional conservation of these miRNAs between rice and maize. However, some of the drought-responsive miRNAs in plants, such as *miR319* (Zhou et al., 2013), *miR393* (Bian et al., 2012), and *miR396* (Liu et al., 2009), had very low abundance in our samples and relatively stable expressions under drought stress. This is probably because the promoters of these miRNA genes are not active in the tissues analyzed in our experiment. Nevertheless, we cannot rule out the possibility that these miRNAs do not function in response to drought stress in maize.

### Local Genomic Stability Contributes to the Tissue Specificity of miRNAs and Variants

Local genomic features, such as the density of PCGs, proportion of TEs and rates of recombination, affect the removal of both genic and TE sequences (Wicker et al., 2007; Tian et al., 2009). We demonstrated that most of maize miRNA genes were distributed in chromosome arms, thus reflecting a preferential evolution driven by genetic recombination. The disruptive effects of TEs have been documented extensively, as they integrate into the regulatory or coding region of host genes or induce ectopic/nonallelic recombination (Jangam et al., 2017). We observed that there were significant differences among expression levels of miRNAs located in PCGs, TEs, and UIs. Although TEs constitute approximately 90% of the maize genome (SanMiguel et al., 1996), only 31.1% of miRNA genes were located in TEs. Such a low retention rate in TEs is more likely due to a faster purging rate of miRNA genes from TEs, which generally has limited deleterious effects on gene function and is, thus, more easily purified. We observed an extremely high abundance of miRNA genes in UIs compared with PCGs and TEs. If UIs are nearly neutral, the detected higher abundance for miRNA genes in UIs indicated that these intergenic sequences are under some level of specific functional constraints.

The modification of miRNAs, which gives rise to many isomiRs, occurs extensively and affects miRNA diversity or functional specificity (Sablok et al., 2015). Our analysis demonstrated that the number of isomiRs and frequency of isomiR types varied dramatically in different tissues. The first nucleotides on both 5′ and 3′ ends of miRNAs were generally ‘U’. It is known that 3′ adenylation increases miRNA stability, and 3′ uridylation enhances miRNA degradation in plants (Lu et al., 2009; Wang et al., 2019). Thus, the presence of ‘U’ on the terminus of 5′ addition isomiRs in all maize tissues suggested that the modification of isomiRs probably occurred to avoid degradation. Additionally, we observed diverse terminal nucleotides on the 3′ end of isomiRs among the individual maize tissues, implying a tissue-specific development landmark, where the activities of enzymes involved in miRNA degradation or modification are dramatically adjusted. The abundance of isomiRs was highly associated with the genomic location of miRNA genes, implying that local genomic stability might be a principal factor contributing to the evolution of tissue specificity among maize isomiRs.

### Complex Interplays May Exist Among Duplication Status, Local Genome Stability, Tissue Specificity, and miRNA Abundance

Gene duplication is a major factor responsible for the evolution of genes with novel functions. When both members of a duplicated gene pair are retained, it is believed that neither member was involved in local genomic recombination. By contrast, a singleton originates solely from a local deletion, insertion, or translocation (Panchy et al., 2016). In general, PCGs often accumulate in genomic regions with active recombination. In this study, we found that the singleton-to-duplicate ratio of miRNA genes in PCGs was significantly higher than that in TEs and UIs, suggesting that miRNA singletons located in PCGs were likely formed through the accumulation of small deletions generated via genetic recombination, which removed single gene. These observations suggested that most miRNA singletons in maize, particularly those located in PCGs, were formed by the rapid removal of their duplicate copies after neofunctionalization. Like miRNA genes in PCGs, relatively high rates of preservation of singletons were also observed in tissue-specific miRNAs. This indicated that relatively stronger selection acted on tissue-specific miRNA singletons, which contributed to functional constraints associated with tissue specificity.

In addition, we observed that the singleton-to-duplicate ratios of expressed miRNAs in individual tissues were significantly lower than the whole genome-wide ratio. This implied a relatively higher number of non-expressed miRNA singletons than duplicates. Together with the higher genome-wide abundance miRNA duplicates than singletons, these phenomena support the gene balance hypothesis, according to which a successful genome has evolved an optimum balance of gene products binding with one another to produce multi-subunit complexes. If a gene pair is fractionated, dosage imbalance may reduce fitness or have lethal consequences. Thus, gene duplicates are generally retained (Freeling, 2009). We found that the singleton-to-duplicate ratio of miRNA genes in PCGs was significantly higher than those in TEs and UIs, and in PCGs, the accumulations of miRNA singletons were significantly lower than those of miRNA duplicates. These observations implied that the ratio of miRNA singletons in PCGs may be affected largely by gene dosage of the resident PCGs, and highly expressed miRNA genes were preferentially retained as duplicates.

### GD Events Have Effects on Evolutionary Dynamics of Maize miRNA Loci in Relation to Tissue Specificity

Gene families develop via duplication of an original ancestral gene, and gene duplicates subsequently diverge from the original gene under selection (Chauve et al., 2008). This divergence occurs at varied rates, depending on the selective forces. In the case of *miR164* and *miR399* families, gene duplication and divergence appeared to result in the sub-functionalization of members of these two families. Although both families were reported to function in the development of maize roots (Tang and Chu, 2017; Hashimoto et al., 2018), we showed that *miR164a* and *miR164d* were specifically expressed in silk, and *miR399b* and *miR399e* exhibited leaf-specific expression. These two cases are typical examples of sub-functionalization, where gene copies originated from an ancestral gene eventually acquire specialized function in different tissues. Often, the functional diversification of miRNA gene duplicates occurs over time through a series of modifications to archetype miRNAs, such as nucleotide polymorphisms, resulting in the specialization of miRNA gene function in a particular tissue (Ameres and Zamore, 2013). This typically involves changes in complementary pairing with target genes that drive the expression dynamics in relevant pathways (Baldrich et al., 2018). In animals, the seed region is considered as the basic determinant of miRNA specificity (Chandradoss et al., 2015). Here, we showed that despite the divergence in the expression profiles of *miR164* and *miR399* family members, the seed region was considerably conserved (**Supplementary Figure 8**). Based on these observations, we proposed that SNPs preferentially occurred in the 3′ tail region of miRNA genes after GD, thus leading to the functional divergence of each duplicate copy. In the case of *miR2275* family, of which one member showed anther-specific expression, three homoeologous pairs formed by TD were detected. However, their syntenic copies were absent from the maize genome. Together with their consistent expression profiles, we hypothesized that paralogs produced by the WGD event lost function and were eventually deleted prior to the TD event.

### Further Perspectives: Exploring the Dynamics of Newly Identified miRNAs in Different Maize Tissues With Spatial, Temporal, and Environmental Dimensions

In this study, we newly identified 271 maize miRNA genes and uncovered the tissues-specific expression patterns. As important regulatory elements of PCGs, miRNAs have gained increasing interests in the last few years to understand their dynamics roles of regulation mechanism involved in a variety of biological processes associated with growth, development, and stress responses in plants. We expect that, in the future, these new miRNA genes will be explored using large-scale sRNA-seq data from different developmental stages and tissues of maize under diverse environmental conditions. Moreover, further in-depth structural and functional analysis using genetic and molecular biology technologies will be performed to validate these new miRNA genes in maize.

## Data Availability Statement

The six sRNA-seq datasets generated in our study have been deposited into the NCBI's SRA database under the accession numbers SRR6322649, SRR6322650, SRR6322651, SRR6322652, SRR6322653, and SRP125654.

## Author Contributions

YX and TZ performed the research and analyzed the data. YL contributed analytic tools and methods. ZM conceived the project and wrote the article.

## Funding

This work was supported by the Natural Science Foundation Research Project of Shaanxi Province of China (2019JQ-096), the College Students' Platform for Innovation Training Program from Northwest A&F University (201803094), and the Fund of Northwest A&F University (Z111021603).

## Conflict of Interest

The authors declare that the research was conducted in the absence of any commercial or financial relationships that could be construed as a potential conflict of interest.

## Abbreviations

DAP, days after pollination; DTA, days to anthesis; DTS, days to silk production; DCL1, DICER-LIKE1; DEMs, differentially expressed microRNAs; DS, drought-stressed; easiRNAs, epigenetically activated small interfering RNAs; GD, genome duplication; GWAS, genome-wide association study; miRNAs, microRNAs; isomiRs, microRNA isoforms; NCBI, National Center for Biotechnology Information; nt, nucleotide; phasiRNAs, phased small interfering RNAs; *PHAS* loci, phasiRNA-generating loci; PCGs, protein-coding genes; SD, segmental duplication; SRA, Sequence Read Archive; SNPs, single nucleotide polymorphisms; sRNAs, small RNAs; siRNAs, small interfering RNAs; TD, tandem duplication; ta-siRNAs, *trans*-acting small interfering RNAs; TPM, transcript per million; TE, transposable element; UIs, unclassified intergenic sequences; WGD, whole genome duplication; WW, well-watered.
