# Supplementary material for: Integrated Analysis of Large-Scale Omics Data Revealed Relationship Between Tissue Specificity and Evolutionary Dynamics of Small RNAs in Maize (Zea mays)
Source: Front Genet. 2020 Feb 11;11:51. doi: 10.3389/fgene.2020.00051 (PMC7026458; doi:10.3389/fgene.2020.00051)
Supplement: Supplementary file 2 [file Table_2.docx]

**Supplementary Table 2.** Ten populations of SNP data collected for GWAS.

| Population name | Population size | SNP number | Trait number | Traits | Reference |
| --- | --- | --- | --- | --- | --- |
| USNAM | 4699 | 2216729 | 17 | Branch Number; Branch Zone; Cob Diameter; Cob Length; Days to Anthesis; Days to Silking; Ear Row Number; Ear Height; Leaf Length; Leaf Width; Oil Content; Plant Height; Protein; Spike Length; Starch; Tassel Length; Upper Leaf Angle | Tian et al., Nature Genetics, 2011, 43: 159-162 |
| NCRPIS | 2659 | 681257 | 6 | Ear Height; Days to Silking; Days to Anthesis; Kernel Color; Plant Height; Sweet | Lu et al., Nature Communication, 2015, 6: 6914 |
| IAP368 | 368 | 2649402 | 14 | Cob Diameter; Cob Weight; Ear Diameter; Ear height; Ear Leaf Length; Ear Leaf Width; Ear Length; Heading Time; Kernel Width; Kernel Number Per Row; Leaf Number Per Row; Plant Height; Days to Anthesis; Days to Silking | Liu et al., Molecular Plant, 2017, 10: 414-426 |
| IAP disease resistance | 854 | 56110 | 4 | Puebla in Mexico 2010; Puebla in Mexico 2011; Morelos in Mexico 2011; Combine | Chen et al., G3-Genes Genomes Genetics, 2016, 6: 3803-3815 |
| IAP insect resistance | 289 | 246476 | 6 | Days to Anthesis; Days to Silking; Kernel Resistance; Plant Height; Stem Damage; Tunnel Length | Samayoa et al., BMC Plant Biology, 2015, 15:35 |
| MAGIC | 529 | 54234 | 4 | Days to anthesis; Ear Height; Grain Yield; Plant Height | Dell’Acqua et al., Genome Biology, 2015, 16: 167 |
| IBM | 193 | 2216729 | 6 | Days to anthesis; Days to Silking; Leaf Angle; Leaf Length; Leaf Width; Upper Leaf Angle | Hung et al., PNAS, 2012, 109: E1913-1921 |
| Maize282 | 282 | 2216729 | 4 | Days to anthesis; Days to Silking; Plant Height; Ear height | Peiffer et al., Genetics, 2014, 196: 1337-1356 |
| Maize pan genome | 503 | 458181 | 3 | Days to anthesis; Days to Silking; Last Juvenile Leaf | Hirsch et al., The Plant Cell, 2014, 26: 121-135 |
| ROAM | 1260 | 43832 | 4 | Cob Weight; Ear Length; Ear Weight; Ear Branch Number | Xiao et al., New Phytologist, 2016, 10: 1095-1106 |
