# Supplementary material for: Integrated Analysis of Large-Scale Omics Data Revealed Relationship Between Tissue Specificity and Evolutionary Dynamics of Small RNAs in Maize (Zea mays)
Source: Front Genet. 2020 Feb 11;11:51. doi: 10.3389/fgene.2020.00051 (PMC7026458; doi:10.3389/fgene.2020.00051)
Supplement: Supplementary file 3 [file Table_3.docx]

**Supplementary Table 3.** Primers for miRNA qRT-PCR assay

| **miRNAs** | **Primer type** | **Primer sequence** | **Tm** | **Length** | **GC%** |
| --- | --- | --- | --- | --- | --- |
| zma-miR_N143a-3p | Forward primer | CGCAAAGATCTATGGATATGGC | 59.3 | 22 | 45.5 |
|  | RT primer | GTCGTATCCAGTGCAGGGTCCGAGGTATTCGCACTGGATACGACATGCCA | 74 | 50 | 56 |
| zma-miR_N25a-5p | Forward primer | CGTTTGGGAGGAAGAGTAATGG | 60.6 | 22 | 50 |
|  | RT primer | GTCGTATCCAGTGCAGGGTCCGAGGTATTCGCACTGGATACGACTCCATT | 73 | 50 | 50 |
| zma-miR159h-3p | Forward primer | TTTGGAGTGAAGGGAGCTCTG | 59.4 | 21 | 52.4 |
|  | RT primer | GTCGTATCCAGTGCAGGGTCCGAGGTATTCGCACTGGATACGACCAGAGC | 74 | 50 | 58 |
| zma-miR164a-5p | Forward primer | TGGAGAAGCAGGGCACGT | 59.3 | 18 | 61.1 |
|  | RT primer | GTCGTATCCAGTGCAGGGTCCGAGGTATTCGCACTGGATACGACTGCACG | 75 | 50 | 58 |
| zma-miR164e-5p | Forward primer | TGGAGAAGCAGGACACGTGAG | 60.9 | 21 | 57.1 |
|  | RT primer | GTCGTATCCAGTGCAGGGTCCGAGGTATTCGCACTGGATACGACCTCACG | 74 | 50 | 58 |
| zma-miR169b-5p | Forward primer | CAGCCAAGGATGACTTGCC | 58.5 | 19 | 57.9 |
|  | RT primer | GTCGTATCCAGTGCAGGGTCCGAGGTATTCGCACTGGATACGACTCGGCA | 75 | 50 | 58 |
| zma-miR169n-5p | Forward primer | CTTAGCCAAGAATGGCTTGCC | 61.7 | 21 | 52.4 |
|  | RT primer | GTCGTATCCAGTGCAGGGTCCGAGGTATTCGCACTGGATACGACAGGCAA | 74 | 50 | 56 |
| zma-miR171f-3p | Forward primer | TGATTGAGCCGTGCCAATATC | 60.9 | 21 | 47.6 |
|  | RT primer | GTCGTATCCAGTGCAGGGTCCGAGGTATTCGCACTGGATACGACGATATT | 72 | 50 | 52 |
| zma-miR2275c-5p | Forward primer | GCAGGATTAGAGGGACTTGAACC | 61.2 | 23 | 52.2 |
|  | RT primer | GTCGTATCCAGTGCAGGGTCCGAGGTATTCGCACTGGATACGACGGTTCA | 74 | 50 | 56 |
| Universal Reverse |  | GTGCAGGGTCCGAGGT | 60.2 | 16 | 68 |
| 18S rRNA | Forward primer | CCATCCCTCCGTAGTTAGCTTCT | 61.1 | 23 | 52.2 |
|  | Reverse primer | CCTGTCGGCCAAGGCTATATAC | 60.8 | 22 | 54.5 |
