# Supplementary material for: Integrated Analysis of Large-Scale Omics Data Revealed Relationship Between Tissue Specificity and Evolutionary Dynamics of Small RNAs in Maize (Zea mays)
Source: Front Genet. 2020 Feb 11;11:51. doi: 10.3389/fgene.2020.00051 (PMC7026458; doi:10.3389/fgene.2020.00051)
Supplement: Supplementary file 9 [file Table_9.docx]

**Supplementary Table 9.** Comparison between abundance of miRNA singletons and duplicates.

|  | Singleton(TPM) | Duplication(TPM) | P^a^ |
| --- | --- | --- | --- |
| Total | 74,515 ± 423,544 | 486,872 ± 989,085 | 2.85 × 10^-9^ |
| PCG | 3,891 ± 28,788 | 96,931 ± 208,882 | 1.50 × 10^-5^ |
| TE | 17,598 ± 59,648 | 561,400 ± 1,099,632 | 3.59 × 10^-6^ |
| UI | 165,438 ± 648,295 | 497,346 ± 982,164 | 2.59 × 10^-3^ |

^a^Student’s t test
