# Supplementary material for: Integrated Analysis of Large-Scale Omics Data Revealed Relationship Between Tissue Specificity and Evolutionary Dynamics of Small RNAs in Maize (Zea mays)
Source: Front Genet. 2020 Feb 11;11:51. doi: 10.3389/fgene.2020.00051 (PMC7026458; doi:10.3389/fgene.2020.00051)
Supplement: Supplementary file 10 [file Table_10.docx]

**Supplementary Table 10.** Comparison of singleton-to-duplicate ratios of miRNAs located in three categories of genomic components in maize tissues.

| Tissue | PCGs | | | TEs | | | UIs | | |
| --- | --- | --- | --- | --- | --- | --- | --- | --- | --- |
|  | **Singleton** | **Duplicate** | **Ratio(S/D)^a^** | **Singleton** | **Duplicate** | **Ratio(S/D)^a^** | **Singleton** | **Duplicate** | **Ratio(S/D)^a^** |
| Ear_6week | 41 | 8 | 5.12:1 | 31 | 20 | 1.55:1 | 70 | 50 | 1.4:1 |
| Ear_8week | 7 | 3 | 2.33:1 | 24 | 14 | 1.71:1 | 41 | 47 | 0.87:1 |
| Ear_9week | 10 | 6 | 1.67:1 | 22 | 14 | 1.57:1 | 42 | 48 | 0.88:1 |
| Ear_10week | 9 | 3 | 3:01 | 22 | 19 | 1.16:1 | 41 | 52 | 0.79:1 |
| Early prophase meiocytes | 10 | 4 | 2.5:1 | 17 | 13 | 1.31:1 | 28 | 37 | 0.76:1 |
| Embryo_1d | 36 | 8 | 4.5:1 | 32 | 26 | 1.23:1 | 63 | 51 | 1.24:1 |
| Embryo_3week | 22 | 5 | 4.4:1 | 16 | 15 | 1.07:1 | 49 | 43 | 1.14:1 |
| Embryo_9DAP | 11 | 4 | 2.75:1 | 14 | 10 | 1.4:1 | 32 | 32 | 1:01 |
| Embryo_15DAP | 21 | 7 | 3:01 | 23 | 16 | 1.44:1 | 41 | 50 | 0.82:1 |
| Embryo_20DAP | 21 | 5 | 4.2:1 | 23 | 13 | 1.77:1 | 45 | 48 | 0.94:1 |
| Endosperm_9DAP | 8 | 5 | 1.6:1 | 20 | 12 | 1.67:1 | 39 | 44 | 0.89:1 |
| Endosperm_15DAP | 60 | 7 | 8.57:1 | 24 | 16 | 1.5:1 | 62 | 45 | 1.38:1 |
| Endosperm_20DAP | 69 | 7 | 9.86:1 | 26 | 14 | 1.86:1 | 60 | 49 | 1.22:1 |
| Leaf1_2week | 70 | 7 | 10:01 | 25 | 14 | 1.79:1 | 62 | 48 | 1.29:1 |
| Leaf2_2week | 51 | 7 | 7.29:1 | 37 | 16 | 2.31:1 | 68 | 51 | 1.33:1 |
| Leaf1_3week | 27 | 8 | 3.38:1 | 25 | 23 | 1.09:1 | 50 | 52 | 0.96:1 |
| Leaf2_3week | 31 | 7 | 4.43:1 | 31 | 14 | 2.21:1 | 60 | 50 | 1.2:1 |
| Leaf3_3week | 29 | 5 | 5.8:1 | 25 | 13 | 1.92:1 | 47 | 56 | 0.84:1 |
| Leaf_4week | 28 | 7 | 4:01 | 19 | 13 | 1.46:1 | 45 | 56 | 0.8:1 |
| Leaf_5week | 16 | 7 | 2.29:1 | 26 | 20 | 1.3:1 | 46 | 58 | 0.79:1 |
| Leaf_6week | 15 | 7 | 2.14:1 | 19 | 19 | 1:01 | 43 | 56 | 0.77:1 |
| Leaf_9week | 37 | 5 | 7.4:1 | 24 | 13 | 1.85:1 | 51 | 49 | 1.04:1 |
| Leaf_expanded_4week | 41 | 6 | 6.83:1 | 21 | 23 | 0.91:1 | 54 | 56 | 0.96:1 |
| Leaf_immature_5week | 44 | 7 | 6.29:1 | 30 | 20 | 1.5:1 | 61 | 62 | 0.98:1 |
| Leaf_mature_5week | 38 | 7 | 5.43:1 | 28 | 19 | 1.47:1 | 54 | 58 | 0.93:1 |
| Leaf_wrapped_4week | 48 | 7 | 6.86:1 | 29 | 19 | 1.53:1 | 61 | 59 | 1.03:1 |
| Pollen_early | 34 | 5 | 6.8:1 | 30 | 20 | 1.5:1 | 52 | 62 | 0.84:1 |
| Pollen_germination | 16 | 5 | 3.2:1 | 22 | 16 | 1.38:1 | 51 | 45 | 1.13:1 |
| Pollen1_mature | 13 | 1 | 13:01 | 10 | 13 | 0.77:1 | 31 | 31 | 1:01 |
| Pollen2_mature | 22 | 3 | 7.33:1 | 13 | 14 | 0.93:1 | 35 | 32 | 1.09:1 |
| Root1_2week | 23 | 2 | 11.5:1 | 17 | 14 | 1.21:1 | 44 | 30 | 1.47:1 |
| Root2_2week | 27 | 5 | 5.4:1 | 23 | 21 | 1.1:1 | 46 | 53 | 0.87:1 |
| Seed | 31 | 6 | 5.17:1 | 35 | 12 | 2.92:1 | 46 | 48 | 0.96:1 |
| Shoot apical meristem_4week | 34 | 7 | 4.86:1 | 32 | 15 | 2.13:1 | 57 | 46 | 1.24:1 |
| Stalk_2week | 17 | 3 | 5.67:1 | 19 | 16 | 1.19:1 | 45 | 48 | 0.94:1 |
| Silk_9week | 28 | 7 | 4:01 | 21 | 21 | 1:01 | 54 | 53 | 1.02:1 |
| Silk_11week | 10 | 5 | 2:01 | 16 | 23 | 0.7:1 | 38 | 50 | 0.76:1 |
| Silk_12week | 36 | 8 | 4.5:1 | 29 | 21 | 1.38:1 | 50 | 55 | 0.91:1 |
| Tassel_4week | 32 | 8 | 4:01 | 21 | 21 | 1:01 | 48 | 60 | 0.8:1 |
| Tassel_8week | 23 | 5 | 4.6:1 | 26 | 23 | 1.13:1 | 52 | 48 | 1.08:1 |
| Tassel_9week | 10 | 4 | 2.5:1 | 20 | 14 | 1.43:1 | 35 | 40 | 0.88:1 |
| Vegetative apex_2week | 19 | 7 | 2.71:1 | 26 | 25 | 1.04:1 | 55 | 59 | 0.93:1 |

^a^ The ratio of singletons to duplicates.
