# Supplementary material for: Integrated Analysis of Large-Scale Omics Data Revealed Relationship Between Tissue Specificity and Evolutionary Dynamics of Small RNAs in Maize (Zea mays)
Source: Front Genet. 2020 Feb 11;11:51. doi: 10.3389/fgene.2020.00051 (PMC7026458; doi:10.3389/fgene.2020.00051)
Supplement: Supplementary file 13 [file DataSheet_1.pdf]

Supplementary Data 1. 419 miRNA loci identified in this study.

zma-MIR1432

```
Chromosome:     chr7
Start:          165932774
End:            165932896
Strand:         -
Strand bias:    1.0
Abundance bias:0.984
MFE:            -78.30
```

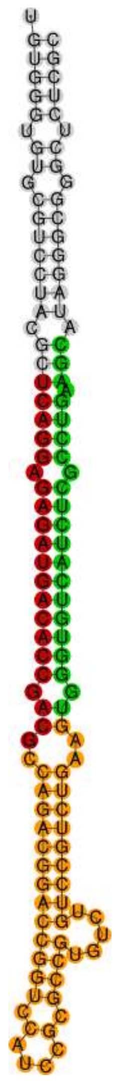

```
                    Mature                                                                        Star
UGUGGGUGUGCGUCCUACGCUCAGGAGAGAUGACACCGACGCCAGACGGACCGGUCCAUCCGCGCCGUGUCUGUCCGUCUGAAGUGGGUGUCAUCUCGCCUGAAGCAUAGGGCGGGCUCUCGC    8730.
.(((((.((.(((((((.(((((((.(((((((((((.((..(((((((((((((.(....).)))).....)))))))))..)).))))))))))).)))).))).))))))).)).)))))
....................UCAGGAGAGAUGACACCGACG..................................................................................    6343.375289
....................................................................................UGGGUGUCAUCUCGCCUGAAGC.................    175.145476
..................GCUCAGGAGAGAUGACACCGA....................................................................................    0.568493
....................UCAGGAGAGAUGACACCGAC...................................................................................    532.998134
....................UCAGGAGAGAUGACACCG.....................................................................................    41.63415
....................UCAGGAGAGAUGACACCGACGCC................................................................................    33.388253
....................UCAGGAGAGAUGACACCGA....................................................................................    446.726226
....................UCAGGAGAGAUGACACCGACGC.................................................................................    16.315076
....................UCAGGAGAGAUGACACCGAA...................................................................................    9.78035
....................UCAGGAGAGAUGACACCGAU...................................................................................    19.64596
....................UCGGGAGAGAUGACACCGACG..................................................................................    2.07492
....................UCAGGAGAGAUGACACCGACU..................................................................................    72.263126
....................UCAGGAGAGAUGACACCGACC..................................................................................    16.17945
....................UCAGGAGAGAUGACACCGACA..................................................................................    584.351377
....................UCAGGAGAGAUGACACAGACG..................................................................................    12.11528
....................UCAGGAGAGAUGACAACGACG..................................................................................    5.80868
....................UCAGUAGAGAUGACACCGACG..................................................................................    2.07492
....................UCAGGAGGGAUGACACCGACG..................................................................................    4.38673
....................UCAGGAGAGAUGACAUCGACG..................................................................................    1.11605
....................CCAGGAGAGAUGACACCGACG..................................................................................    1.92837
....................UUAGGAGAGAUGACACCGACG..................................................................................    4.69492
....................UCAGGAGAGAUGACACCGAAG..................................................................................    10.203384
....................UCAGGAGAGGUGACACCGACG..................................................................................    4.60976
....................UCAGGCGAGAUGACACCGACG..................................................................................    2.184295
....................UCAGGAGAGAUGACACCGAUG..................................................................................    1.83169
....................UCAGGAGAGAUUACACCGACG..................................................................................    6.90316
....................UCAGGAGAGAUGACACCGCCG..................................................................................    11.6128
....................UAAGGAGAGAUGACACCGACG..................................................................................    2.13697
....................UCAUGAGAGAUGACACCGACG..................................................................................    1.83169
....................UCAGGAGAGAUGACACCGU....................................................................................    0.97644
....................UCAGGAGAGAUGACACCGCC...................................................................................    1.11605
....................UCAGGAGAGAUGACACCGACGA.................................................................................    9.477382
....................UCAGGAGAGAUGACACCGACGU.................................................................................    91.015348
....................UCAGGAGAGAUGCCACCGACG..................................................................................    1.692613
.....................CAGGAGAGAUGACACCGACG..................................................................................    12.103239
.....................CAGGAGAGAUGACACCGAC...................................................................................    0.371561
......................AGGAGAGAUGACACCGACGCC................................................................................    2.053871
....................................................................................UGGGUGUCAUCUCGCCUGAAG..................    2.548064
....................................................................................UGGGUGUCAUCUCGCCUGAAGCU................    0.86284
......................................................................................GGUGUCAUCUCGCCUGAAGC.................    98.274307
......................................................................................GGUGUCAUCUCGCCUGAAG..................    0.243195
```

zma-MIR156d

Chromosome: chr5
Start: 94725821
End: 94725991
Strand: -
Strand bias: 1.0
Abundance bias: 0.991
MFE: -88.40

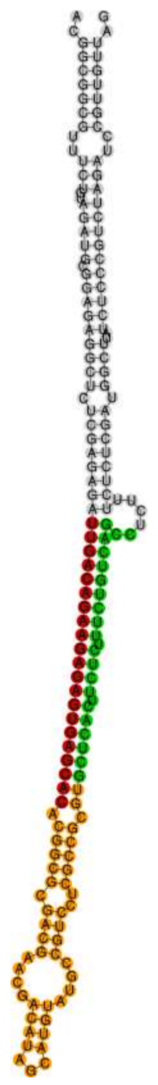

```
                                          Mature                                                              Star
ACGGCGGCGUUUCUGUAGAUGCGGAGAGGCUCUCGAGAGAUUGACAGAAGAGAGUGAGCACACGGCGCGACGAACGACAUAGCAUGUAUGCCGUCCUCGCCGCGUGCUCACUUCUCUUUCUGUCAGCCUCUUUCUCUCGAUGGCUUAUCUCCCGUCUAGAUCCGUUGUUAG   1056637.8
..(((((((..(((..(((((.(((((((((.(((((((((((((((((((((((((((((.(((((.((((....((((...))))....))))..))))).)))))))).)))).)))))))))......)))))))).))))..)))))))))))))..)))))))..
........................................UUGACAGAAGAGAGUGAGCAC..............................................................................................................   454754.154608
.........................................................................................................GCUCACUUCUCUUUCUGUCAGCC...........................................   897.86943
.......................................AUUGACAGAAGAGAGUGAGCAC..............................................................................................................   378.544806
.......................................AUUGACAGAAGAGAGUGAGCA...............................................................................................................   311.399901
.......................................AUUGACAGAAGAGAGCGAGC................................................................................................................   255.437318
.......................................AUUGACAGAAGAGAGCGAGCAC..............................................................................................................   6890.155689
........................................UUGACAGAAGAGAGUGAGCA...............................................................................................................   5663.212102
........................................UUGACAGAAGAGAGUGAGC................................................................................................................   7053.501975
........................................UUGACAGAAGAGAGUGAGCACA.............................................................................................................   585.977629
........................................UUGACAGAAGAGAGCGAGCAC..............................................................................................................   49762.477181
........................................UUGACAGAAGAGAGUGAGUAC..............................................................................................................   261.030552
........................................UUGACGGAAGAGAGUGAGCAC..............................................................................................................   383.686177
........................................UUGCCAGAAGAGAGUGAGCAC..............................................................................................................   246.183721
........................................UUGACAGAAGAGAGUGAGCCC..............................................................................................................   1011.740781
........................................UUGACAGAAGAGAGUGAGU................................................................................................................   1342.258838
........................................UUGACAGAAGAGAGUGAGA................................................................................................................   337.190553
........................................UUGACAGAAGAGCGUGAGCAC..............................................................................................................   305.621051
........................................UUGACAGAAGAGAUUGAGCAC..............................................................................................................   521.73085
........................................UUGACAGAAGAGAGUGAGCACC.............................................................................................................   551.421093
........................................UUGACAGAAGAGAGUGAGCACU.............................................................................................................   1633.850316
........................................UUGACAGAAGGGAGUGAGCAC..............................................................................................................   368.058968
........................................UUGACAGAAGAGAGCGAGC................................................................................................................   625.846718
........................................UUGACAGAGGAGAGUGAGCAC..............................................................................................................   350.73692
........................................UCGACAGAAGAGAGUGAGCAC..............................................................................................................   474.923344
........................................UUGACAGAAGAUAGUGAGCAC..............................................................................................................   259.0594
........................................UUGACAGGAGAGAGUGAGCAC..............................................................................................................   299.99901
........................................UUGACAGAAGAGAGUGAGAAC..............................................................................................................   624.690479
........................................UUGGCAGAAGAGAGUGAGCAC..............................................................................................................   424.222591
........................................UUGACAGAAGAGAGUGAUCAC..............................................................................................................   274.323042
........................................UUGACAGAAGAGGGUGAGCAC..............................................................................................................   392.089028
........................................UUGACAGAAGAGAGUGGGCAC..............................................................................................................   525.521175
........................................UUGACAGAAGAGAGUGCGCAC..............................................................................................................   497.568994
........................................UUGACAGAAGAGAGGGAGCAC..............................................................................................................   433.26253
........................................UUGACAGAAGAGAGCGAGCA...............................................................................................................   319.290445
........................................UUGACAGAAGAGAGUGAGCAA..............................................................................................................   1999.230613
........................................UUGACAGAAGAGAGUGAGCAG..............................................................................................................   235.468368
........................................UUGACAGAAGAGAGUGAGCAU..............................................................................................................   5226.120249
........................................UUGACAGAAGAGAGUGAGCGC..............................................................................................................   526.33982
.........................................UGACAGAAGAGAGUGAGCUC..............................................................................................................   281.048229
.........................................UGACAGAAGAGAGUGAGCACC.............................................................................................................   1828.279371
.........................................UGACAGAAGAGAGUGAGCACU.............................................................................................................   6098.791209
.........................................UGACAGAAGAGAGUGACCAC..............................................................................................................   646.770901
.........................................UGACAGAAGAGAGUAAGCAC..............................................................................................................   488.193738
.........................................UGACAGAAGAGAUUGAGCAC..............................................................................................................   1505.804512
.........................................UGACAGAAGAGAGUGAGA................................................................................................................   554.015096
.........................................UGACAGAAGAGAGUGAGU................................................................................................................   2232.969562
.........................................UGACAGAAGAGCGUGAGCAC..............................................................................................................   706.951071
.........................................UGACAGAAGAGAGUGAGCGC..............................................................................................................   1109.259318
.........................................UGACAGAAGGGAGUGAGCAC..............................................................................................................   1001.809665
.........................................UGACAGAAGAGAGUGAGAAC..............................................................................................................   1871.723337
.........................................UGACAGAAGAGACUGAGCAC..............................................................................................................   428.37133
.........................................UGACAGAAGAGAGUGAACAC..............................................................................................................   1044.717943
.........................................UGACAGAAGCGAGUGAGCAC..............................................................................................................   495.361686
.........................................UGACAGAAGAGAAUGAGCAC..............................................................................................................   260.689562
.........................................UGACAGAAUAGAGUGAGCAC..............................................................................................................   480.6204
.........................................UGACAGAAGAGAGUGUGCAC..............................................................................................................   252.70159
.........................................UGACAGAAGAGAGUGAGCAA..............................................................................................................   10411.15771
.........................................UGACAGAAGAGAGUGAGCAU..............................................................................................................   9796.070943
.........................................UGACAGAAGAGAGUGAUCAC..............................................................................................................   585.074974
.........................................UGACAGAAGAGAGUGAGCAG..............................................................................................................   765.609921
.........................................UGACAGAAGAGAGUGCGCAC..............................................................................................................   1154.036353
.........................................UGACAGAAGAGAGUGGGCAC..............................................................................................................   1099.312511
.........................................UGACAGAAGAGAGUGAGUAC..............................................................................................................   588.143268
.........................................UGACAGAAGAGAGUUAGCAC..............................................................................................................   541.985546
.........................................UGACAGAAGAAAGUGAGCAC..............................................................................................................   246.464492
.........................................UGACAGAAGAUAGUGAGCAC..............................................................................................................   611.235981
.........................................UGACAGACGAGAGUGAGCAC..............................................................................................................   352.374538
.........................................UGACAGAAAAGAGUGAGCAC..............................................................................................................   408.181761
.........................................UGACAGAGGAGAGUGAGCAC..............................................................................................................   903.11262
.........................................UGACAGAAGAGGGUGAGCAC..............................................................................................................   940.452296
.........................................UGACAGAAGAGAGUGAGCCC..............................................................................................................   3924.353668
........................................................................................................UGCUCACUUCUCUUUCUGUCAGC............................................   46602.614945
........................................................................................................UGCUCACUUCUCUUUCUGUCAG.............................................   947.328945
........................................................................................................UGCUCACUUCUCUUUCUGUCAGU............................................   467.182479
........................................................................................................UGCUCACUUCUCUUUCUGUCAGA............................................   329.83814
.........................................................................................................GCUCACUUCUCUUUCUGUCA..............................................   1394.92418
.........................................................................................................GCUCACUUCUCUUUCUGUCAGCCU..........................................   439.547642
.........................................................................................................GCUCACUUCUCUUUCUGUCAGC............................................   364945.120691
.........................................................................................................GCUCACUUCUCUUUCUGUCAG.............................................   5692.0141
.........................................................................................................GCUCACUUCUCCUUCUGUCAGC............................................   807.484845
.........................................................................................................GCUCACUUCUCUUUCCGUCAGC............................................   689.69269
.........................................................................................................GCUCACUUCUCUUCCUGUCAGC............................................   611.91629
.........................................................................................................GCUCACUUCCCUUUCUGUCAGC............................................   709.862347
.........................................................................................................GCUCACUUCUCUUUCUGUCAGU............................................   3699.902946
.........................................................................................................GCUCACUUCUCUUUCUGUCAGA............................................   1703.20216
.........................................................................................................GCUCACUUCUUUUUCUGUCAGC............................................   401.250028
.........................................................................................................GCUCACUUCUCUUUCUGUUAGC............................................   297.73002
.........................................................................................................GCUCACUUCUCUUUCUGUCAAC............................................   267.94471
.........................................................................................................GCUCACUUUUCUUUCUGUCAGC............................................   321.30935
.........................................................................................................GCUCACUUCUCUUUCUGUCAUC............................................   317.18406
.........................................................................................................GCUCACUUCUCUUUCUGCCAGC............................................   709.20568
.........................................................................................................GCUCACUUCUCUUUCUGUCAGCA...........................................   400.173986
.........................................................................................................GCUCACUUCUCUUUCUGUCGGC............................................   815.711125
.........................................................................................................GCUCACUUCUCUUUCUGUCACC............................................   311.0367
.........................................................................................................GCUCACUUCUCUUUCUUUCAGC............................................   282.18636
..........................................................................................................CUCACUUCUCUUUCUGUCAGC............................................   1172.328604
...........................................................................................................UCACUUCUCUUUCUGUCAGC............................................   251.08668
............................................................................................................CACUUCUCUUUCUGUCAGC............................................   478.815288
```

zma-MIR156j

Chromosome: chr7
Start: 134663764
End: 134663890
Strand: -
Strand bias: 1.0
Abundance bias: 1.0
MFE: -78.40

```
                     Mature                                                             Star
GUCGAGUGGACCUCGGGAGCGAUGACAGAAGAGAGAGAGCACAACCCAGCACCAGCGAGGAAAAGCCUCGCUUCUGCGAGGGCCGUGUGCUCUCUGCUCUCACUGUCAUCGCCCACAGGCCACCGAA    2450.4
.(((.((((.(((..((.((((((((((.((((((((((((((.(((.(((..(((((((.....)))))))..)))..)))...)))))))))).)))))..)))))))))))))..)))))))).
.....................AUGACAGAAGAGAGAGAGCAC.....................................................................................    1817.089913
........................................................................................GCUCUCUGCUCUCACUGUCAUCG................    8.877873
...................CGAUGACAGAAGAGAGAGAGCAC.....................................................................................    6.71503
.....................AUGACAGAAGAGAGAGAGCCC.....................................................................................    3.07183
.....................AUGACAGAAGAGAGAGAGGAC.....................................................................................    0.182396
.....................AUGACAGAAGAGAGUGAGCAC.....................................................................................    210.70001
.....................AUGACAGAAGAGAGAGAGAAC.....................................................................................    1.4323
.....................AUGACAGAAGAGAGCGAGCAC.....................................................................................    10.643896
.....................AUGAUAGAAGAGAGAGAGCAC.....................................................................................    0.182396
.....................AUGACAGAAGAUAGAGAGCAC.....................................................................................    0.925992
......................UGACAGAAGAGAGAGCGCAC.....................................................................................    7.14283
......................UGACAGAAGAGAGAGAGCCC.....................................................................................    18.64309
......................UGACAGAAGAGCGAGAGCAC.....................................................................................    3.776767
......................UGACAGAAGAGAGAGAGAAC.....................................................................................    15.27574
......................UGACAGAAGAGAGAGAACAC.....................................................................................    3.534425
......................UGACAGAAGAGACAGAGCAC.....................................................................................    0.790382
......................UGACAGAAGGGAGAGAGCAC.....................................................................................    0.182396
......................UGACAGAAGAGUGAGAGCAC.....................................................................................    0.273594
......................UGACAGAAGAGAGAGAGCACC....................................................................................    3.030125
......................UGACAGAAGAGAGAGAGCACU....................................................................................    8.987345
......................UGACAGAAGAGAGUGAGCACAAC..................................................................................    0.306198
......................UGACAGAAGAGAGAGAGCGC.....................................................................................    0.729584
......................UGACAGAAGAGAGAGGGCAC.....................................................................................    0.212795
......................UGACAGAAGAGAGAGACCAC.....................................................................................    1.4323
......................UGACAGAAGCGAGAGAGCAC.....................................................................................    1.84152
......................UGACAGAAGAUAGAGAGCAC.....................................................................................    7.47396
......................UGACAGAUGAGAGAGAGCAC.....................................................................................    0.182396
......................UGACAGAAGAGAGUGAGCACAA...................................................................................    127.59336
......................UGACAGAAGAGAGCGAGCACAA...................................................................................    4.68598
......................UGACAGAAGAAAGAGAGCAC.....................................................................................    0.45599
......................UGACAGAAGAGAGAGAUCAC.....................................................................................    0.212795
......................UGACAGAAGAGAGGGAGCACA....................................................................................    0.212795
......................UGACAGAAGAGAGAUAGCAC.....................................................................................    0.303993
........................................................................................GCUCUCUGCUCUCACUGUCAUC.................    181.215092
........................................................................................GCUCUCUGCUCUCACUGUCAU..................    2.153684
```

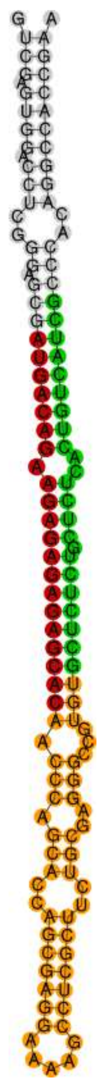

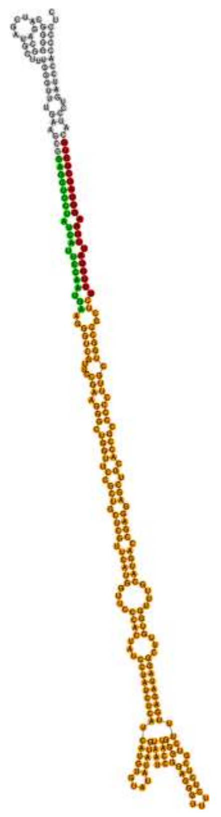

zma-MIR159a

```
Chromosome:     chr8
Start:          10753063
End:            10753317
Strand:         +
Strand bias:    1.0
Abundance bias:0.992
MFE:            -115.70
```

```
                                Star                                                                                                                                                                                                                Mature
CGGGGGCAGAUCGAUGCUUUGGGUUUGAAGCGGAGCUCCUAUCAUUCCAAUGAAGGGUCGUUCCGAAGGGCUGGUUCCGCUGCUCGUUCAUGGUUCCCACUAUCCUAUCUCAUCAUGUGUAUAUAUGUAAUCCAUGGGGGAGGGUUUCUCUCGUCUUUGAGAUAGGCUUGUGGUUUGCAUGACCGAGGAGCUGCACCGCCCCCUUGCUGGCCGCUCUUUGGAUUGAAGGGAGCUCUGCAUCCUGAUCCACCCCUC   2420602.2
.(((((((......)))..((((((.((.(((((((((((.(((.(((((.((..(((((...(((.(((.((((.(.(((.((((.(((((....((((...(((((((((.(((((....)))))........(((.(((((...))))).))).)))))))))...))))....))))).)))).))).).)))).))).))).)))))..)).))))).))).))))))))))).))..))))))))))..
........................................................................................................................................................................................................................UUUGGAUUGAAGGGAGCUCUG..................   2094847.30758
................................GAGCUCCUAUCAUUCCAAUGA..........................................................................................................................................................................................................   4402.31272
..........................................................................................................................................................................................................................UGGAUUGAAGGGAGCUCUG..................   1374.373211
.........................................................................................................................................................................................................................UUGGAUUGAAGGGAGCUCUG..................   13633.689879
.........................................................................................................................................................................................................................UUGGAUUGAAGGGAGCUCUGC.................   1423.052809
.........................................................................................................................................................................................................................UUGGAUUGAAGGGAGCUCU...................   7514.391484
.........................................................................................................................................................................................................................UUGGAUUGAAGGGAGCUCA...................   2528.435527
........................................................................................................................................................................................................................UUUGGAUUGAAGGGAGCUCU...................   57808.408198
........................................................................................................................................................................................................................UUUGGAUUGAAGGGAGCUC....................   9606.984822
........................................................................................................................................................................................................................UUUGGAUUGAAGGGAGCUCUGC.................   13232.256586
........................................................................................................................................................................................................................UUUGGAUUGAAGGGAGCU.....................   28200.28837
........................................................................................................................................................................................................................CUUGGAUUGAAGGGAGCUCUG..................   5017.204677
........................................................................................................................................................................................................................UUUGGAUUGAAGCGAGCUCUG..................   941.898487
........................................................................................................................................................................................................................GUUGGAUUGAAGGGAGCUCUG..................   1128.47105
........................................................................................................................................................................................................................UUUGGAUUGAAGGGAGCCCUG..................   3828.556262
........................................................................................................................................................................................................................UUUGGAUUGAAGGGAGUUCUG..................   3526.787305
........................................................................................................................................................................................................................UUUGGAUUGAAGGGGGCUCUG..................   5599.041951
........................................................................................................................................................................................................................UUUGGAUUGGAGGGAGCUCUG..................   4808.677193
........................................................................................................................................................................................................................UUUGGAUUGAAGGGAGCUCA...................   1934.464014
........................................................................................................................................................................................................................UUUGGAUUGAAGGGAGCUCG...................   1201.450566
........................................................................................................................................................................................................................UUUGGAUUGAAGGGAUCUCUG..................   1907.029509
........................................................................................................................................................................................................................UUUGGAUUGAAGUGAGCUCUG..................   2000.996833
........................................................................................................................................................................................................................UUUGAAUUGAAGGGAGCUCUG..................   961.65235
........................................................................................................................................................................................................................UUUGGAUUAAAGGGAGCUCUG..................   1477.829011
........................................................................................................................................................................................................................UUUGGAUCGAAGGGAGCUCUG..................   3057.222592
........................................................................................................................................................................................................................UUUGGAUUGAAGGGAGCUAUG..................   1036.570326
........................................................................................................................................................................................................................UUUGGAUUGAAGGGACCUCUG..................   1062.213095
........................................................................................................................................................................................................................UUUGGAUUGAAGGGAGCUU....................   1364.933593
........................................................................................................................................................................................................................UUUGGAUUGAAGGGAGCUCUGU.................   11612.660635
........................................................................................................................................................................................................................UUUGGAUUGAAGGGAGCUCUGA.................   7785.789427
........................................................................................................................................................................................................................UUUGGAUUGAGGGGAGCUCUG..................   5412.52836
........................................................................................................................................................................................................................UUUGGAUUGAAGGGAGCC.....................   2738.745178
........................................................................................................................................................................................................................UUUCGAUUGAAGGGAGCUCUG..................   1021.09699
........................................................................................................................................................................................................................UUUGGAUUGAAGGGAGCUUUG..................   2318.275848
........................................................................................................................................................................................................................UUUGGAUUGAAGGUAGCUCUG..................   1065.50587
........................................................................................................................................................................................................................UUCGGAUUGAAGGGAGCUCUG..................   3206.71264
........................................................................................................................................................................................................................UUUAGAUUGAAGGGAGCUCUG..................   1295.452203
........................................................................................................................................................................................................................UUUGGAUUGAAGGGAGCA.....................   1767.630002
........................................................................................................................................................................................................................UUUGGAUUGAAGGGCGCUCUG..................   3214.762723
........................................................................................................................................................................................................................AUUGGAUUGAAGGGAGCUCUG..................   1368.697405
........................................................................................................................................................................................................................UUUGGAUUGAAGGGAGCUCUU..................   5736.170604
........................................................................................................................................................................................................................UUUGGAUUGAAGGGAGCUCUA..................   18034.942634
........................................................................................................................................................................................................................UUUUGAUUGAAGGGAGCUCUG..................   1599.959962
........................................................................................................................................................................................................................UUUGUAUUGAAGGGAGCUCUG..................   1901.063226
........................................................................................................................................................................................................................UUUGGAUUUAAGGGAGCUCUG..................   1098.602285
........................................................................................................................................................................................................................UUUGGAUUGAAGGGUGCUCUG..................   993.680022
........................................................................................................................................................................................................................UUUGGAUUGAAGGGAACUCUG..................   1170.296412
........................................................................................................................................................................................................................UCUGGAUUGAAGGGAGCUCUG..................   2698.461968
........................................................................................................................................................................................................................UUUGGAUUGAAAGGAGCUCUG..................   1401.933651
........................................................................................................................................................................................................................UUUGGAUUGAAGGGAGCUCCG..................   3891.1139
........................................................................................................................................................................................................................UUUGGAUUGAAUGGAGCUCUG..................   1720.388241
........................................................................................................................................................................................................................UUUGGAUUGACGGGAGCUCUG..................   1817.725125
........................................................................................................................................................................................................................UUUGGACUGAAGGGAGCUCUG..................   3565.656643
........................................................................................................................................................................................................................UUUGGAUUGAAGGGAGCUCUC..................   2467.109776
........................................................................................................................................................................................................................UUUGGAUUGAAGGGAGCUCUUC.................   1736.730806
........................................................................................................................................................................................................................UUUGGGUUGAAGGGAGCUCUG..................   4141.167563
........................................................................................................................................................................................................................UUUGGAUUGAAGAGAGCUCUG..................   1191.13955
........................................................................................................................................................................................................................UUUGGAUUGAAGGGAGCUCC...................   4114.78917
```

zma-MIR159b

```
Chromosome:    chr8
Start:         10950937
End:           10951152
Strand:        +
Strand bias:   1.0
Abundance bias:0.993
MFE:           -103.70
```

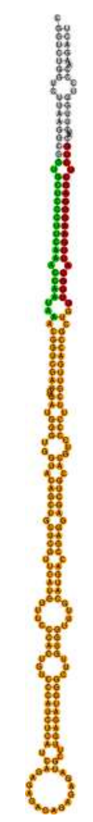

```
                           Star                                                                                                                                                           Mature
CGGUCUGGUCUUAAGGCGGUGCUCCCUUCAAACCAAUAAACGGUCGAUCUGAUGGGUGGUACAGCUGCUCGUUCAUGGUUCCCACUGUCCCAUCUCAUCAGAGAGAGAGAGAGAUGCUUGAGAUGGGCUUGUGGUUUGCAUGACCGAGGAGCUGCACGUCCCCUUCGUUGACCGCUGUUUGGAUUGAAGGGAGCUCUGCAUCUUGGUCCCAAGACU    2412330.4
.(((((((..(((((((((.(((((((((((.((((..(.((((((((..((.(((..((.(((((.((((.(((((....((((...(((((((((.((..............))..)))))))))...))))....))))).)))).))))).))...))).)))))))))).)..)))).))))))))))).))))..)))))..)).)))))
.................................................................................................................................................................................UUUGGAUUGAAGGGAGCUCUG..................    2094847.30758
..................GUGCUCCCUUCAAACCAAUAA.................................................................................................................................................................................    17.34942
...................................................................................................................................................................................UGGAUUGAAGGGAGCUCUG..................    1374.373211
..................................................................................................................................................................................UUGGAUUGAAGGGAGCUCUG..................    13633.689879
..................................................................................................................................................................................UUGGAUUGAAGGGAGCUCUGC.................    1423.052809
..................................................................................................................................................................................UUGGAUUGAAGGGAGCUCU...................    7514.391484
..................................................................................................................................................................................UUGGAUUGAAGGGAGCUCA...................    2528.435527
.................................................................................................................................................................................UUUGGAUUGAAGGGAGCUCU...................    57808.408198
.................................................................................................................................................................................UUUGGAUUGAAGGGAGCUC....................    9606.984822
.................................................................................................................................................................................UUUGGAUUGAAGGGAGCUCUGC.................    13232.256586
.................................................................................................................................................................................UUUGGAUUGAAGGGAGCU.....................    28200.28837
.................................................................................................................................................................................CUUGGAUUGAAGGGAGCUCUG..................    5017.204677
.................................................................................................................................................................................UUUGGAUUGAAGCGAGCUCUG..................    941.898487
.................................................................................................................................................................................GUUGGAUUGAAGGGAGCUCUG..................    1128.47105
.................................................................................................................................................................................UUUGGAUUGAAGGGAGCCCUG..................    3828.556262
.................................................................................................................................................................................UUUGGAUUGAAGGGAGUUCUG..................    3526.787305
.................................................................................................................................................................................UUUGGAUUGAAGGGGGCUCUG..................    5599.041951
.................................................................................................................................................................................UUUGGAUUGGAGGGAGCUCUG..................    4808.677193
.................................................................................................................................................................................UUUGGAUUGAAGGGAGCUCA...................    1934.464014
.................................................................................................................................................................................UUUGGAUUGAAGGGAGCUCG...................    1201.450566
.................................................................................................................................................................................UUUGGAUUGAAGGGAUCUCUG..................    1907.029509
.................................................................................................................................................................................UUUGGAUUGAAGUGAGCUCUG..................    2000.996833
.................................................................................................................................................................................UUUGAAUUGAAGGGAGCUCUG..................    961.65235
.................................................................................................................................................................................UUUGGAUUAAAGGGAGCUCUG..................    1477.829011
.................................................................................................................................................................................UUUGGAUCGAAGGGAGCUCUG..................    3057.222592
.................................................................................................................................................................................UUUGGAUUGAAGGGAGCUAUG..................    1036.570326
.................................................................................................................................................................................UUUGGAUUGAAGGGACCUCUG..................    1062.213095
.................................................................................................................................................................................UUUGGAUUGAAGGGAGCUU....................    1364.933593
.................................................................................................................................................................................UUUGGAUUGAAGGGAGCUCUGU.................    11612.660635
.................................................................................................................................................................................UUUGGAUUGAAGGGAGCUCUGA.................    7785.789427
.................................................................................................................................................................................UUUGGAUUGAGGGGAGCUCUG..................    5412.52836
.................................................................................................................................................................................UUUGGAUUGAAGGGAGCC.....................    2738.745178
.................................................................................................................................................................................UUUCGAUUGAAGGGAGCUCUG..................    1021.09699
.................................................................................................................................................................................UUUGGAUUGAAGGGAGCUUUG..................    2318.275848
.................................................................................................................................................................................UUUGGAUUGAAGGUAGCUCUG..................    1065.50587
.................................................................................................................................................................................UUCGGAUUGAAGGGAGCUCUG..................    3206.71264
.................................................................................................................................................................................UUUAGAUUGAAGGGAGCUCUG..................    1295.452203
.................................................................................................................................................................................UUUGGAUUGAAGGGAGCA.....................    1767.630002
.................................................................................................................................................................................UUUGGAUUGAAGGGCGCUCUG..................    3214.762723
.................................................................................................................................................................................AUUGGAUUGAAGGGAGCUCUG..................    1368.697405
.................................................................................................................................................................................UUUGGAUUGAAGGGAGCUCUU..................    5736.170604
.................................................................................................................................................................................UUUGGAUUGAAGGGAGCUCUA..................    18034.942634
.................................................................................................................................................................................UUUUGAUUGAAGGGAGCUCUG..................    1599.959962
.................................................................................................................................................................................UUUGUAUUGAAGGGAGCUCUG..................    1901.063226
.................................................................................................................................................................................UUUGGAUUUAAGGGAGCUCUG..................    1098.602285
.................................................................................................................................................................................UUUGGAUUGAAGGGUGCUCUG..................    993.680022
.................................................................................................................................................................................UUUGGAUUGAAGGGAACUCUG..................    1170.296412
.................................................................................................................................................................................UCUGGAUUGAAGGGAGCUCUG..................    2698.461968
.................................................................................................................................................................................UUUGGAUUGAAAGGAGCUCUG..................    1401.933651
.................................................................................................................................................................................UUUGGAUUGAAGGGAGCUCCG..................    3891.1139
.................................................................................................................................................................................UUUGGAUUGAAUGGAGCUCUG..................    1720.388241
.................................................................................................................................................................................UUUGGAUUGACGGGAGCUCUG..................    1817.725125
.................................................................................................................................................................................UUUGGACUGAAGGGAGCUCUG..................    3565.656643
.................................................................................................................................................................................UUUGGAUUGAAGGGAGCUCUC..................    2467.109776
.................................................................................................................................................................................UUUGGAUUGAAGGGAGCUCUUC.................    1736.730806
.................................................................................................................................................................................UUUGGGUUGAAGGGAGCUCUG..................    4141.167563
.................................................................................................................................................................................UUUGGAUUGAAGAGAGCUCUG..................    1191.13955
.................................................................................................................................................................................UUUGGAUUGAAGGGAGCUCC...................    4114.78917
```

zma-MIR159c

Chromosome: chr3
Start: 2020763
End: 2020975
Strand: -
Strand bias: 1.0
Abundance bias: 0.99
MFE: -109.60

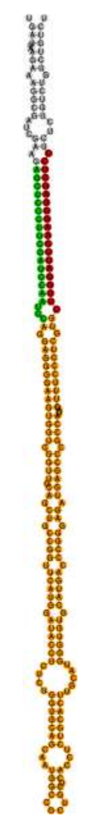

```
                      Star                                                                                                                                                              Mature
UGAUUAAGAAAGGCGAUCGAAGAGCUCCCUUCGAUCCAAUCCAGGAGGGGAAGUGGUCGGUUGCAGCUGCCGGUUCAUGGAUACCUCUCUGGUGCAGCAAUGGCCGCUGCUCACCUCUGCACUUGCAUGGGUGUGCAUGACCCGGGAGAUGAGCCCGCCAUCAUCUUUCCCUCGUGCUUGGAUUGAAGGGAGCUCCUCUCUGUCUGUCUGUCU	68326.6
.(((..(((.(((((...((.((((((((((((((((((..((.(((((((((((((.((((.((.((.((((.(((((.((((((....(((((((...((((....)).))...)))))))).....)))))).))))).)))).)).)))))).))))....))))))))).)).))))))))))))))))).))..))))).)))))).
...............................................................................................................................................................................CUUGGAUUGAAGGGAGCUCC.................	21108.794179
......................AGCUCCCUUCGAUCCAAUCC..........................................................................................................................................................................	365.772585
.................................................................................................................................................................................UGGAUUGAAGGGAGCUCC.................	85.496093
................................................................................................................................................................................UUGGAUUGAAGGGAGCUCCUC...............	54.673827
................................................................................................................................................................................UUGGAUUGAAGGGAGCUC..................	258.093567
................................................................................................................................................................................UUGGAUUGAAGGGAGCUCCU................	120.037154
................................................................................................................................................................................UUGGAUUGAAGGGAGCUCC.................	29731.908604
................................................................................................................................................................................UUGGAUUAAAGGGAGCUCC.................	37.73562
................................................................................................................................................................................UUGGAUUGAAUGGAGCUCC.................	20.801281
................................................................................................................................................................................UUGGAUUGAAGUGAGCUCC.................	13.801621
................................................................................................................................................................................UUGGAUUUAAGGGAGCUCC.................	15.85548
................................................................................................................................................................................UUGGAUUGAGGGGAGCUCC.................	63.895326
................................................................................................................................................................................UUGGAUUGAAGGGAGCUCG.................	185.46674
................................................................................................................................................................................UUGGAUUGAAGGGAGCGCC.................	145.77109
................................................................................................................................................................................UUGGAUUGAAGGGAGCACC.................	52.47929
................................................................................................................................................................................UUGGAUUGAAGGGAACUCC.................	13.301541
................................................................................................................................................................................UUGGAUUGAAGGGAGCUAC.................	21.94921
................................................................................................................................................................................UUGGAUUGAAGGGAGCUU..................	14.50895
................................................................................................................................................................................UUGGAUUGAAGGUAGCUCC.................	14.186797
................................................................................................................................................................................UUGGAUUGAAGGAAGCUCC.................	13.81626
................................................................................................................................................................................UUGGAUUGAAGGGAGUUCC.................	41.08726
................................................................................................................................................................................UUGGAUUGAAGGGGGCUCC.................	62.838344
................................................................................................................................................................................UUGGAUUGAAGGGAGCUUC.................	46.485587
................................................................................................................................................................................UUGGAUUGAAGGGAGCUCCC................	63.866144
................................................................................................................................................................................UUGGAUUGAAGGGAGCUCCA................	100.89685
................................................................................................................................................................................UUGGAUUGAAGGGAGCUCCG................	21.452526
................................................................................................................................................................................UUGGAUUGAAGGGAGCCCC.................	105.05678
................................................................................................................................................................................UUGGAUUGAAGGGAGCUCA.................	2528.435527
................................................................................................................................................................................UUGGAUUGGAGGGAGCUCC.................	75.715505
...............................................................................................................................................................................CUUGGAUUGAAGGGAGCUC..................	132.84121
...............................................................................................................................................................................CUUGGAUUGAAGGGAGCUCCU................	100.532462
...............................................................................................................................................................................CUUGGAUUGAAGGGAGCUCCUC...............	59.243127
...............................................................................................................................................................................CUUGGAUUGAAGGGAGCU...................	162.060625
...............................................................................................................................................................................CUUGGAUUGAAGGGAGCUCCC................	26.043812
...............................................................................................................................................................................CUUGGAUUGGAGGGAGCUCC.................	35.58194
...............................................................................................................................................................................CUUGGAUUGAAGGGAGCUUC.................	26.37201
...............................................................................................................................................................................CUUGGAUUGAAGGGAGCUCU.................	4379.057342
...............................................................................................................................................................................CUUGGAUUGAAGGGAGCUCA.................	1641.890107
...............................................................................................................................................................................CUUGGACUGAAGGGAGCUCC.................	13.84856
...............................................................................................................................................................................CUUGGAUUGAAGGGAGCUCCA................	98.9953
...............................................................................................................................................................................CUUGGAUUGAAGGGAGCACC.................	30.94016
...............................................................................................................................................................................CUUGGAUUGAGGGGAGCUCC.................	23.253323
...............................................................................................................................................................................CUUGGGUUGAAGGGAGCUCC.................	28.870887
...............................................................................................................................................................................CUUGGAUUGAAGGGGGCUCC.................	29.29743
...............................................................................................................................................................................CUUGGAUCGAAGGGAGCUCC.................	18.842926
...............................................................................................................................................................................CUUGGAUUGAAGGGAGCGCC.................	120.813811
...............................................................................................................................................................................CUUGGAUUGAAGGGAGCUCG.................	115.03188
...............................................................................................................................................................................CUUGGAUUGAAGGGAGUUCC.................	16.28426
...............................................................................................................................................................................CUUGGAUUAAAGGGAGCUCC.................	15.04464
...............................................................................................................................................................................CUCGGAUUGAAGGGAGCUCC.................	13.33291
...............................................................................................................................................................................CCUGGAUUGAAGGGAGCUCC.................	21.70419
...............................................................................................................................................................................CUUGGAUUGAAGGGAGCCCC.................	79.2603
...............................................................................................................................................................................UUUGGAUUGAAGGGAGCUCC.................	4114.78917
.............................................................................................................................................................................UGCUUGGAUUGAAGGGAGC....................	12.25846
......................AGCUCCCUUCGAUCCAAUC...........................................................................................................................................................................	659.013824
......................AGCUCCCUUCGAUCCAAUCCA.........................................................................................................................................................................	32.25944
......................AGCUCCCUUCGAUCCAAU............................................................................................................................................................................	59.818512
......................AGCUCCCUUCGAUCCAAUCU..........................................................................................................................................................................	19.066058
```

zma-MIR159d

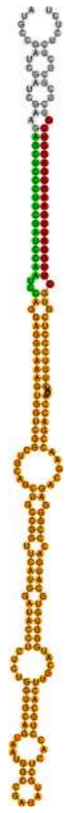

```
Chromosome:    chr8
Start:         27675692
End:           27675897
Strand:        +
Strand bias:   1.0
Abundance bias:0.988
MFE:           -105.70
```

```
                Star                                                                                                                                                         Mature
AUGCCGAUCGAUCGAAGAGCUCCCUUCGAUCCAAUCCAGGAGGGAAAGUGGUCGGCUGCAGCUGCCGGUUCAUGGAUACCUCUCUGGUGCAGCAAUGGCCGAGAUGCUCACCUGCACUUGCAUGGGUGUGCAUGACCCGGGAGACGAACCGACCAUCAUCUUUCCCUCGUGCUUGGAUUGAAGGGAGCUCCUCUCUCUCUCUCUCU    68519.3
.....((..((..((.((((((((((((((((((..((.((((((((((((((((......((.((((.(((((.((((((....(((((((....(((......)))...))))))).....)))))).))))).)))).)).....))))))).....))))))))).)).)))))))))))))))))).))..))..)).....
...........................................................................................................................................................................CUUGGAUUGAAGGGAGCUCC...............    21108.794179
.................AGCUCCCUUCGAUCCAAUCC.........................................................................................................................................................................    365.772585
.............................................................................................................................................................................UGGAUUGAAGGGAGCUCC...............    85.496093
............................................................................................................................................................................UUGGAUUGAAGGGAGCUCCUC.............    54.673827
............................................................................................................................................................................UUGGAUUGAAGGGAGCUC................    258.093567
............................................................................................................................................................................UUGGAUUGAAGGGAGCUCCU..............    120.037154
............................................................................................................................................................................UUGGAUUGAAGGGAGCUCC...............    29731.908604
............................................................................................................................................................................UUGGAUUAAAGGGAGCUCC...............    37.73562
............................................................................................................................................................................UUGGAUUGAAUGGAGCUCC...............    20.801281
............................................................................................................................................................................UUGGAUUGAAGUGAGCUCC...............    13.801621
............................................................................................................................................................................UUGGAUUUAAGGGAGCUCC...............    15.85548
............................................................................................................................................................................UUGGAUUGAGGGGAGCUCC...............    63.895326
............................................................................................................................................................................UUGGAUUGAAGGGAGCUCG...............    185.46674
............................................................................................................................................................................UUGGAUUGAAGGGAGCGCC...............    145.77109
............................................................................................................................................................................UUGGAUUGAAGGGAGCACC...............    52.47929
............................................................................................................................................................................UUGGAUUGAAGGGAACUCC...............    13.301541
............................................................................................................................................................................UUGGAUUGAAGGGAGCUAC...............    21.94921
............................................................................................................................................................................UUGGAUUGAAGGGAGCUU................    14.50895
............................................................................................................................................................................UUGGAUUGAAGGUAGCUCC...............    14.186797
............................................................................................................................................................................UUGGAUUGAAGGAAGCUCC...............    13.81626
............................................................................................................................................................................UUGGAUUGAAGGGAGUUCC...............    41.08726
............................................................................................................................................................................UUGGAUUGAAGGGGGCUCC...............    62.838344
............................................................................................................................................................................UUGGAUUGAAGGGAGCUUC...............    46.485587
............................................................................................................................................................................UUGGAUUGAAGGGAGCUCCC..............    63.866144
............................................................................................................................................................................UUGGAUUGAAGGGAGCUCCA..............    100.89685
............................................................................................................................................................................UUGGAUUGAAGGGAGCUCCG..............    21.452526
............................................................................................................................................................................UUGGAUUGAAGGGAGCCCC...............    105.05678
............................................................................................................................................................................UUGGAUUGAAGGGAGCUCA...............    2528.435527
............................................................................................................................................................................UUGGAUUGGAGGGAGCUCC...............    75.715505
...........................................................................................................................................................................CUUGGAUUGAAGGGAGCUC................    132.84121
...........................................................................................................................................................................CUUGGAUUGAAGGGAGCUCCU..............    100.532462
...........................................................................................................................................................................CUUGGAUUGAAGGGAGCUCCUC.............    59.243127
...........................................................................................................................................................................CUUGGAUUGAAGGGAGCU..................    162.060625
...........................................................................................................................................................................CUUGGAUUGAAGGGAGCUCCC..............    26.043812
...........................................................................................................................................................................CUUGGAUUGGAGGGAGCUCC...............    35.58194
...........................................................................................................................................................................CUUGGAUUGAAGGGAGCUUC...............    26.37201
...........................................................................................................................................................................CUUGGAUUGAAGGGAGCUCU...............    4379.057342
...........................................................................................................................................................................CUUGGAUUGAAGGGAGCUCA...............    1641.890107
...........................................................................................................................................................................CUUGGACUGAAGGGAGCUCC...............    13.84856
...........................................................................................................................................................................CUUGGAUUGAAGGGAGCUCCA..............    98.9953
...........................................................................................................................................................................CUUGGAUUGAAGGGAGCACC...............    30.94016
...........................................................................................................................................................................CUUGGAUUGAGGGGAGCUCC...............    23.253323
...........................................................................................................................................................................CUUGGGUUGAAGGGAGCUCC...............    28.870887
...........................................................................................................................................................................CUUGGAUUGAAGGGGGCUCC...............    29.29743
...........................................................................................................................................................................CUUGGAUCGAAGGGAGCUCC...............    18.842926
...........................................................................................................................................................................CUUGGAUUGAAGGGAGCGCC...............    120.813811
...........................................................................................................................................................................CUUGGAUUGAAGGGAGCUCG...............    115.03188
...........................................................................................................................................................................CUUGGAUUGAAGGGAGUUCC...............    16.28426
...........................................................................................................................................................................CUUGGAUUAAAGGGAGCUCC...............    15.04464
...........................................................................................................................................................................CUCGGAUUGAAGGGAGCUCC...............    13.33291
...........................................................................................................................................................................CCUGGAUUGAAGGGAGCUCC...............    21.70419
...........................................................................................................................................................................CUUGGAUUGAAGGGAGCCCC...............    79.2603
...........................................................................................................................................................................UUUGGAUUGAAGGGAGCUCC...............    4114.78917
.........................................................................................................................................................................UGCUUGGAUUGAAGGGAGC..................    12.25846
.................AGCUCCCUUCGAUCCAAUC..........................................................................................................................................................................    659.013824
.................AGCUCCCUUCGAUCCAAUCCA........................................................................................................................................................................    32.25944
.................AGCUCCCUUCGAUCCAAU...........................................................................................................................................................................    59.818512
.................AGCUCCCUUCGAUCCAAUCU.........................................................................................................................................................................    19.066058
```

zma-MIR159f

Chromosome: chr3
Start: 25007818
End: 25008065
Strand: -
Strand bias: 1.0
Abundance bias: 0.992
MFE: -107.00

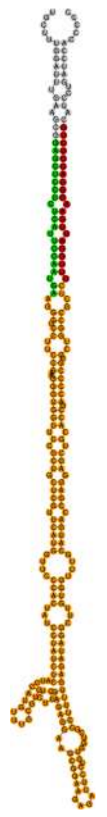

```
                  Star                                                                                                                                                                                                  Mature
UGCUUUGGAUUUGAAGCGGAGCUCCUCUCAUUCCAAUGAAGGGUCCUUCCGAAGGGUGGUUCCGCUGCUCGUUCAUGGUUCCCACUAUCCUAUCUCAUCCAUGUUUGCGUGUCUUCGAGAGGAAGCGGAAGAGAUUCCUGUGGUCGCCUUUUGAGAUAGGCUUGUGGUUUGCAUGACCGAGGAGCUGCACCGACCCCGUGCUGGCCGCUCUUUGGAUUGAAGGGAGCUCUGCAUCCUGAUCCACCCCC   2415981.2
.....((((((.((.((((((((((.(((.(((((.((..((.((..(((..(((((((.(.(((.((((.(((((....((((...((((((((...((((....)))).....((((((..((((((....)))).)).....)))))))))))))...))))....))))).)))).))).).))).))))))).)..))))..)).))))).))).))))))))))).))..))))))..... 
..................................................................................................................................................................................................................UUUGGAUUGAAGGGAGCUCUG.................   2094847.30758
..................GAGCUCCUCUCAUUCCAAUGA.................................................................................................................................................................................................................   417.085501
....................................................................................................................................................................................................................UGGAUUGAAGGGAGCUCUG.................   1374.373211
...................................................................................................................................................................................................................UUGGAUUGAAGGGAGCUCUG.................   13633.689879
...................................................................................................................................................................................................................UUGGAUUGAAGGGAGCUCUGC................   1423.052809
...................................................................................................................................................................................................................UUGGAUUGAAGGGAGCUCU..................   7514.391484
...................................................................................................................................................................................................................UUGGAUUGAAGGGAGCUCA..................   2528.435527
..................................................................................................................................................................................................................UUUGGAUUGAAGGGAGCUCU..................   57808.408198
..................................................................................................................................................................................................................UUUGGAUUGAAGGGAGCUC...................   9606.984822
..................................................................................................................................................................................................................UUUGGAUUGAAGGGAGCUCUGC................   13232.256586
..................................................................................................................................................................................................................UUUGGAUUGAAGGGAGCU....................   28200.28837
..................................................................................................................................................................................................................CUUGGAUUGAAGGGAGCUCUG.................   5017.204677
..................................................................................................................................................................................................................UUUGGAUUGAAGCGAGCUCUG.................   941.898487
..................................................................................................................................................................................................................GUUGGAUUGAAGGGAGCUCUG.................   1128.47105
..................................................................................................................................................................................................................UUUGGAUUGAAGGGAGCCCUG.................   3828.556262
..................................................................................................................................................................................................................UUUGGAUUGAAGGGAGUUCUG.................   3526.787305
..................................................................................................................................................................................................................UUUGGAUUGAAGGGGGCUCUG.................   5599.041951
..................................................................................................................................................................................................................UUUGGAUUGGAGGGAGCUCUG.................   4808.677193
..................................................................................................................................................................................................................UUUGGAUUGAAGGGAGCUCA..................   1934.464014
..................................................................................................................................................................................................................UUUGGAUUGAAGGGAGCUCG..................   1201.450566
..................................................................................................................................................................................................................UUUGGAUUGAAGGGAUCUCUG.................   1907.029509
..................................................................................................................................................................................................................UUUGGAUUGAAGUGAGCUCUG.................   2000.996833
..................................................................................................................................................................................................................UUUGAAUUGAAGGGAGCUCUG.................   961.65235
..................................................................................................................................................................................................................UUUGGAUUAAAGGGAGCUCUG.................   1477.829011
..................................................................................................................................................................................................................UUUGGAUCGAAGGGAGCUCUG.................   3057.222592
..................................................................................................................................................................................................................UUUGGAUUGAAGGGAGCUAUG.................   1036.570326
..................................................................................................................................................................................................................UUUGGAUUGAAGGGACCUCUG.................   1062.213095
..................................................................................................................................................................................................................UUUGGAUUGAAGGGAGCUU...................   1364.933593
..................................................................................................................................................................................................................UUUGGAUUGAAGGGAGCUCUGU................   11612.660635
..................................................................................................................................................................................................................UUUGGAUUGAAGGGAGCUCUGA................   7785.789427
..................................................................................................................................................................................................................UUUGGAUUGAGGGGAGCUCUG.................   5412.52836
..................................................................................................................................................................................................................UUUGGAUUGAAGGGAGCC....................   2738.745178
..................................................................................................................................................................................................................UUUCGAUUGAAGGGAGCUCUG.................   1021.09699
..................................................................................................................................................................................................................UUUGGAUUGAAGGGAGCUUUG.................   2318.275848
..................................................................................................................................................................................................................UUUGGAUUGAAGGUAGCUCUG.................   1065.50587
..................................................................................................................................................................................................................UUCGGAUUGAAGGGAGCUCUG.................   3206.71264
..................................................................................................................................................................................................................UUUAGAUUGAAGGGAGCUCUG.................   1295.452203
..................................................................................................................................................................................................................UUUGGAUUGAAGGGAGCA....................   1767.630002
..................................................................................................................................................................................................................UUUGGAUUGAAGGGCGCUCUG.................   3214.762723
..................................................................................................................................................................................................................AUUGGAUUGAAGGGAGCUCUG.................   1368.697405
..................................................................................................................................................................................................................UUUGGAUUGAAGGGAGCUCUU.................   5736.170604
..................................................................................................................................................................................................................UUUGGAUUGAAGGGAGCUCUA.................   18034.942634
..................................................................................................................................................................................................................UUUUGAUUGAAGGGAGCUCUG.................   1599.959962
..................................................................................................................................................................................................................UUUGUAUUGAAGGGAGCUCUG.................   1901.063226
..................................................................................................................................................................................................................UUUGGAUUUAAGGGAGCUCUG.................   1098.602285
..................................................................................................................................................................................................................UUUGGAUUGAAGGGUGCUCUG.................   993.680022
..................................................................................................................................................................................................................UUUGGAUUGAAGGGAACUCUG.................   1170.296412
..................................................................................................................................................................................................................UCUGGAUUGAAGGGAGCUCUG.................   2698.461968
..................................................................................................................................................................................................................UUUGGAUUGAAAGGAGCUCUG.................   1401.933651
..................................................................................................................................................................................................................UUUGGAUUGAAGGGAGCUCCG.................   3891.1139
..................................................................................................................................................................................................................UUUGGAUUGAAUGGAGCUCUG.................   1720.388241
..................................................................................................................................................................................................................UUUGGAUUGACGGGAGCUCUG.................   1817.725125
..................................................................................................................................................................................................................UUUGGACUGAAGGGAGCUCUG.................   3565.656643
..................................................................................................................................................................................................................UUUGGAUUGAAGGGAGCUCUC.................   2467.109776
..................................................................................................................................................................................................................UUUGGAUUGAAGGGAGCUCUUC................   1736.730806
..................................................................................................................................................................................................................UUUGGGUUGAAGGGAGCUCUG.................   4141.167563
..................................................................................................................................................................................................................UUUGGAUUGAAGAGAGCUCUG.................   1191.13955
..................................................................................................................................................................................................................UUUGGAUUGAAGGGAGCUCC..................   4114.78917
```

zma-MIR159j

Chromosome: chr8
Start: 10920936
End: 10921169
Strand: +
Strand bias: 1.0
Abundance bias:0.993
MFE: -105.00

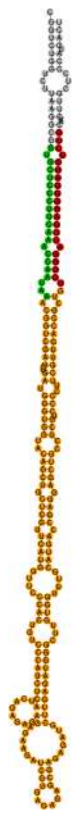

```
                         Star                                                                                                                                                                       Mature
CGGUCUGGUCUUAAGGCGGUGCUCCCUUCAAACCAAUAAACGGUCGAUCUGAUGGGUGGUACAGCUGCUCGUUCAUGGUUCCCACUGUCCCAUCUCAUCAGAGAGAGAGAAGAUCGUGAGAGAGCGAGAGAUGCUUGAGAUGGGCUUGUGGUUUGCAUGACCGAGGAGCUGCCUCGUCCCCUUCGUUGACCGCUGUUUGGAUUGAAGGGAGCUCUGCAUCUUGCUCCCAUGACU   2412353.3
.(((((((...((((((((.(((((((((((.((((..(.((((((((..((.((((((..(((((.((((.(((((....((((...(((((((((........((......((((......))))......)))))))))))...))))....))))).)))).)))))..))).)))..))))))))).)..)))).))))))))))).))))..))))...))).))))
...................................................................................................................................................................................................UUUGGAUUGAAGGGAGCUCUG..................   2094847.30758
..................GUGCUCCCUUCAAACCAAUAA...................................................................................................................................................................................................   17.34942
.....................................................................................................................................................................................................UGGAUUGAAGGGAGCUCUG..................   1374.373211
....................................................................................................................................................................................................UUGGAUUGAAGGGAGCUCUG..................   13633.689879
....................................................................................................................................................................................................UUGGAUUGAAGGGAGCUCUGC.................   1423.052809
....................................................................................................................................................................................................UUGGAUUGAAGGGAGCUCU...................   7514.391484
....................................................................................................................................................................................................UUGGAUUGAAGGGAGCUCA...................   2528.435527
...................................................................................................................................................................................................UUUGGAUUGAAGGGAGCUCU...................   57808.408198
...................................................................................................................................................................................................UUUGGAUUGAAGGGAGCUC....................   9606.984822
...................................................................................................................................................................................................UUUGGAUUGAAGGGAGCUCUGC.................   13232.256586
...................................................................................................................................................................................................UUUGGAUUGAAGGGAGCU.....................   28200.28837
...................................................................................................................................................................................................CUUGGAUUGAAGGGAGCUCUG..................   5017.204677
...................................................................................................................................................................................................UUUGGAUUGAAGCGAGCUCUG..................   941.898487
...................................................................................................................................................................................................GUUGGAUUGAAGGGAGCUCUG..................   1128.47105
...................................................................................................................................................................................................UUUGGAUUGAAGGGAGCCCUG..................   3828.556262
...................................................................................................................................................................................................UUUGGAUUGAAGGGAGUUCUG..................   3526.787305
...................................................................................................................................................................................................UUUGGAUUGAAGGGGGCUCUG..................   5599.041951
...................................................................................................................................................................................................UUUGGAUUGGAGGGAGCUCUG..................   4808.677193
...................................................................................................................................................................................................UUUGGAUUGAAGGGAGCUCA...................   1934.464014
...................................................................................................................................................................................................UUUGGAUUGAAGGGAGCUCG...................   1201.450566
...................................................................................................................................................................................................UUUGGAUUGAAGGGAUCUCUG..................   1907.029509
...................................................................................................................................................................................................UUUGGAUUGAAGUGAGCUCUG..................   2000.996833
...................................................................................................................................................................................................UUUGAAUUGAAGGGAGCUCUG..................   961.65235
...................................................................................................................................................................................................UUUGGAUUAAAGGGAGCUCUG..................   1477.829011
...................................................................................................................................................................................................UUUGGAUCGAAGGGAGCUCUG..................   3057.222592
...................................................................................................................................................................................................UUUGGAUUGAAGGGAGCUAUG..................   1036.570326
...................................................................................................................................................................................................UUUGGAUUGAAGGGACCUCUG..................   1062.213095
...................................................................................................................................................................................................UUUGGAUUGAAGGGAGCUU....................   1364.933593
...................................................................................................................................................................................................UUUGGAUUGAAGGGAGCUCUGU.................   11612.660635
...................................................................................................................................................................................................UUUGGAUUGAAGGGAGCUCUGA.................   7785.789427
...................................................................................................................................................................................................UUUGGAUUGAGGGGAGCUCUG..................   5412.52836
...................................................................................................................................................................................................UUUGGAUUGAAGGGAGCC.....................   2738.745178
...................................................................................................................................................................................................UUUCGAUUGAAGGGAGCUCUG..................   1021.09699
...................................................................................................................................................................................................UUUGGAUUGAAGGGAGCUUUG..................   2318.275848
...................................................................................................................................................................................................UUUGGAUUGAAGGUAGCUCUG..................   1065.50587
...................................................................................................................................................................................................UUCGGAUUGAAGGGAGCUCUG..................   3206.71264
...................................................................................................................................................................................................UUUAGAUUGAAGGGAGCUCUG..................   1295.452203
...................................................................................................................................................................................................UUUGGAUUGAAGGGAGCA.....................   1767.630002
...................................................................................................................................................................................................UUUGGAUUGAAGGGCGCUCUG..................   3214.762723
...................................................................................................................................................................................................AUUGGAUUGAAGGGAGCUCUG..................   1368.697405
...................................................................................................................................................................................................UUUGGAUUGAAGGGAGCUCUU..................   5736.170604
...................................................................................................................................................................................................UUUGGAUUGAAGGGAGCUCUA..................   18034.942634
...................................................................................................................................................................................................UUUUGAUUGAAGGGAGCUCUG..................   1599.959962
...................................................................................................................................................................................................UUUGUAUUGAAGGGAGCUCUG..................   1901.063226
...................................................................................................................................................................................................UUUGGAUUUAAGGGAGCUCUG..................   1098.602285
...................................................................................................................................................................................................UUUGGAUUGAAGGGUGCUCUG..................   993.680022
...................................................................................................................................................................................................UUUGGAUUGAAGGGAACUCUG..................   1170.296412
...................................................................................................................................................................................................UCUGGAUUGAAGGGAGCUCUG..................   2698.461968
...................................................................................................................................................................................................UUUGGAUUGAAAGGAGCUCUG..................   1401.933651
...................................................................................................................................................................................................UUUGGAUUGAAGGGAGCUCCG..................   3891.1139
...................................................................................................................................................................................................UUUGGAUUGAAUGGAGCUCUG..................   1720.388241
...................................................................................................................................................................................................UUUGGAUUGACGGGAGCUCUG..................   1817.725125
...................................................................................................................................................................................................UUUGGACUGAAGGGAGCUCUG..................   3565.656643
...................................................................................................................................................................................................UUUGGAUUGAAGGGAGCUCUC..................   2467.109776
...................................................................................................................................................................................................UUUGGAUUGAAGGGAGCUCUUC.................   1736.730806
...................................................................................................................................................................................................UUUGGGUUGAAGGGAGCUCUG..................   4141.167563
...................................................................................................................................................................................................UUUGGAUUGAAGAGAGCUCUG..................   1191.13955
...................................................................................................................................................................................................UUUGGAUUGAAGGGAGCUCC...................   4114.78917
```

zma-MIR159k

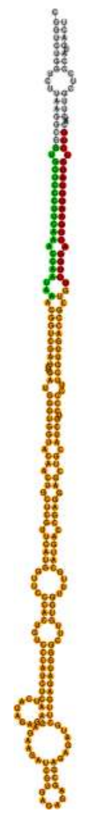

Chromosome: chr8
Start: 10947041
End: 10947274
Strand: +
Strand bias: 1.0
Abundance bias: 0.993
MFE: -100.70

Star / Mature

```
CGGUCUGGUCUUAAGGCGGUGCUCCCUUCAAACCAAUAAACGGUCGAUCUGAUGGGUGGUACAACUGCUCGUUCAUGGUUCCCACUGUCCCAUCUCAUCAGAGAGAGAGAAGAUCGUGAGAGAGCGAGAGAUGCUUGAGAUGGGCUUGUGGUUUGCAUGACCGAGGAGCUGCAUCGUCCCCUUCGUUGACCGCUGUUUGGAUUGAAGGGAGCUCUGCAUCUUGCUCCCAUGACU   2412165.3
.(((((((...((((((((.(((((((((((.((((..(.((((((((..((.(((((((.((.((.((((.(((((....((((...(((((((((........((......((((......))))......)))))))))))...))))....))))).)))).)).)).)))).)))..))))))))).)..)))).)))))))))))).)))))..))))...))).))))
```

Read	Abundance
UUUGGAUUGAAGGGAGCUCUG	2094847.30758
GUGCUCCCUUCAAACCAAUAA	17.34942
UGGAUUGAAGGGAGCUCUG	1374.373211
UUGGAUUGAAGGGAGCUCUG	13633.689879
UUGGAUUGAAGGGAGCUCUGC	1423.052809
UUGGAUUGAAGGGAGCUCU	7514.391484
UUGGAUUGAAGGGAGCUCA	2528.435527
UUUGGAUUGAAGGGAGCUCU	57808.408198
UUUGGAUUGAAGGGAGCUC	9606.984822
UUUGGAUUGAAGGGAGCUCUGC	13232.256586
UUUGGAUUGAAGGGAGCU	28200.28837
CUUGGAUUGAAGGGAGCUCUG	5017.204677
UUUGGAUUGAAGCGAGCUCUG	941.898487
GUUGGAUUGAAGGGAGCUCUG	1128.47105
UUUGGAUUGAAGGGAGCCCUG	3828.556262
UUUGGAUUGAAGGGAGUUCUG	3526.787305
UUUGGAUUGAAGGGGGCUCUG	5599.041951
UUUGGAUUGGAGGGAGCUCUG	4808.677193
UUUGGAUUGAAGGGAGCUCA	1934.464014
UUUGGAUUGAAGGGAGCUCG	1201.450566
UUUGGAUUGAAGGGAUCUCUG	1907.029509
UUUGGAUUGAAGUGAGCUCUG	2000.996833
UUUGAAUUGAAGGGAGCUCUG	961.65235
UUUGGAUUAAAGGGAGCUCUG	1477.829011
UUUGGAUCGAAGGGAGCUCUG	3057.222592
UUUGGAUUGAAGGGAGCUAUG	1036.570326
UUUGGAUUGAAGGGACCUCUG	1062.213095
UUUGGAUUGAAGGGAGCUU	1364.933593
UUUGGAUUGAAGGGAGCUCUGU	11612.660635
UUUGGAUUGAAGGGAGCUCUGA	7785.789427
UUUGGAUUGAGGGGAGCUCUG	5412.52836
UUUGGAUUGAAGGGAGCC	2738.745178
UUUCGAUUGAAGGGAGCUCUG	1021.09699
UUUGGAUUGAAGGGAGCUUUG	2318.275848
UUUGGAUUGAAGGUAGCUCUG	1065.50587
UUCGGAUUGAAGGGAGCUCUG	3206.71264
UUUAGAUUGAAGGGAGCUCUG	1295.452203
UUUGGAUUGAAGGGAGCA	1767.630002
UUUGGAUUGAAGGGCGCUCUG	3214.762723
AUUGGAUUGAAGGGAGCUCUG	1368.697405
UUUGGAUUGAAGGGAGCUCUU	5736.170604
UUUGGAUUGAAGGGAGCUCUA	18034.942634
UUUUGAUUGAAGGGAGCUCUG	1599.959962
UUUGUAUUGAAGGGAGCUCUG	1901.063226
UUUGGAUUUAAGGGAGCUCUG	1098.602285
UUUGGAUUGAAGGGUGCUCUG	993.680022
UUUGGAUUGAAGGGAACUCUG	1170.296412
UCUGGAUUGAAGGGAGCUCUG	2698.461968
UUUGGAUUGAAAGGAGCUCUG	1401.933651
UUUGGAUUGAAGGGAGCUCCG	3891.1139
UUUGGAUUGAAUGGAGCUCUG	1720.388241
UUUGGAUUGACGGGAGCUCUG	1817.725125
UUUGGACUGAAGGGAGCUCUG	3565.656643
UUUGGAUUGAAGGGAGCUCUC	2467.109776
UUUGGAUUGAAGGGAGCUCUUC	1736.730806
UUUGGGUUGAAGGGAGCUCUG	4141.167563
UUUGGAUUGAAGAGAGCUCUG	1191.13955
UUUGGAUUGAAGGGAGCUCC	4114.78917

zma-MIR160a

Chromosome: chr4
Start: 241638514
End: 241638645
Strand: +
Strand bias: 1.0
Abundance bias: 1.0
MFE: -58.20

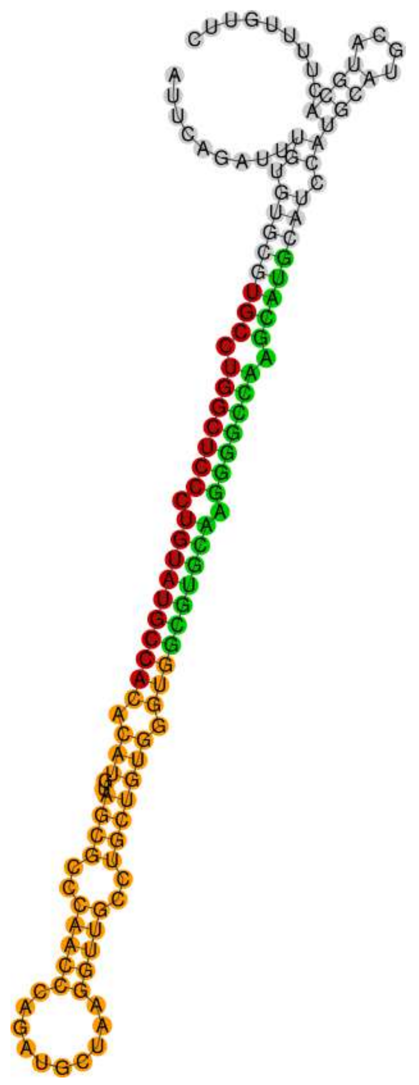

```
                 Mature                                                          Star
AUUCAGAUUUGUGUGCGUGCCUGGCUCCCUGUAUGCCACACAUGUAGCGCCCAACCCAGAUGCUAAGGUUGCCUGCUGUGGGUGGCGUGCAAGGGGCCAAGCAUGCAUCCAUGCAUGCAUGCACUUUUGUUC    88579.2
.........((.((((((((.(((((((.((((((((((.(((..((((..(((((..........)))))..))))))).)))))))))).))))))).)))))))).))((((....)))).........
.................UGCCUGGCUCCCUGUAUGCCA..............................................................................................    82649.23842
....................................................................................GCGUGCAAGGGGCCAAGCAUG...........................    94.833322
................GUGCCUGGCUCCCUGUAUGCC...............................................................................................    59.934456
................GUGCCUGGCUCCCUGUAUGCA...............................................................................................    0.963614
................GUGCCUGGCUCCCUGUAUGCU...............................................................................................    4.02036
.................UGCCUGGCUCCCUGUAUG.................................................................................................    240.304981
.................UGCCUGGCUCCCUGUAUGC................................................................................................    715.659827
.................UGCCUGGCUCCCUGUAUGCCAC.............................................................................................    26.8834
.................UGCCUGGCUCCCUGUAUGCC...............................................................................................    1682.996037
.................UGCCUGGCUCCCUGUAUGCCACA............................................................................................    63.53464
.................UGACUGGCUCCCUGUAUGCCA..............................................................................................    13.284509
.................UGCCAGGCUCCCUGUAUGCCA..............................................................................................    1.681203
.................UGCCUUGCUCCCUGUAUGCCA..............................................................................................    14.287606
.................UGCCUGGCUCCCCGUAUGCCA..............................................................................................    37.434275
.................UGCCUGGCUCCCUGUACGCCA..............................................................................................    74.973683
.................UGCCUGGCUCCCUUUAUGCCA..............................................................................................    1.976325
.................UGCUUGGCUCCCUGUAUGCCA..............................................................................................    25.084326
.................UGCCUGGCUUCCUGUAUGCCA..............................................................................................    10.300446
.................UGCCUGGCUCCCUGUGUGCCA..............................................................................................    37.737876
.................AGCCUGGCUCCCUGUAUGCCA..............................................................................................    6.183748
.................UACCUGGCUCCCUGUAUGCCA..............................................................................................    7.119409
.................UGCCUGGCUCCCUGGAUGCCA..............................................................................................    1.96397
.................UGCCUGGCUCCCUGUAUGUCA..............................................................................................    50.516819
.................UGCCUGGCUCCCUGUAUGCAA..............................................................................................    17.289173
.................UCCCUGGCUCCCUGUAUGCCA..............................................................................................    2.746981
.................UUCCUGGCUCCCUGUAUGCCA..............................................................................................    35.203458
.................UGCCUGGCUCCCUGUAUGCUA..............................................................................................    29.451449
.................UGCCUCGCUCCCUGUAUGCCA..............................................................................................    2.703818
.................UGCCUGGCUCCCUGUAUGACA..............................................................................................    2.665267
.................UGCCUGGCUCCCUGUAUACCA..............................................................................................    486.963489
.................UGCCUGGCUCCCUGUAUUC................................................................................................    13.3184
.................UGCCUGGCUCCCUGAAUGCCA..............................................................................................    1.789584
.................UGCCUGGCUCCUUGUAUGCCA..............................................................................................    4.518435
.................UGCCUGGCUCCCUGUAUCCCA..............................................................................................    6.594481
.................UGCCCGGCUCCCUGUAUGCCA..............................................................................................    28.17989
.................UGUCUGGCUCCCUGUAUGCCA..............................................................................................    27.670767
.................UGCCUGGCUCCCUGUAUGCA...............................................................................................    164.345598
.................UGCCUGGCUCCCUGUAUGGCA..............................................................................................    0.516788
.................UGCAUGGCUCCCUGUAUGCCA..............................................................................................    3.91656
.................CGCCUGGCUCCCUGUAUGCCA..............................................................................................    42.828633
.................UGCCUGGCCCCCUGUAUGCCA..............................................................................................    42.364152
.................UGCCUGGCUCUCUGUAUGCCA..............................................................................................    41.764425
.................UGCCUGGUUCCCUGUAUGCCA..............................................................................................    13.914989
.................UGCCUGGCUCCCUGUAUGCCAU.............................................................................................    38.086041
.................UGCCUGGCUCCCUGUAUGCCAA.............................................................................................    51.844668
.................UGCCUGGCUCCCUGUAUA.................................................................................................    4.73897
.................UGCCUGGCUCCCUGUAUC.................................................................................................    1.69914
.................UGCCUGGCUCCCUGUAUU.................................................................................................    63.701248
.................UGCCUGGCUCCCUGCAUGCCA..............................................................................................    27.12702
.................UGCCUGACUCCCUGUAUGCCA..............................................................................................    21.856695
.................UGCCUGGCUCCCUGUAUGCG...............................................................................................    0.72271
.................UGCCUGGCUCCCUGUAUGCU...............................................................................................    605.924741
.................UGCCUGUCUCCCUGUAUGCCA..............................................................................................    39.091006
.................GGCCUGGCUCCCUGUAUGCCA..............................................................................................    2.558705
.................UGCCUGGCUCCCUAUAUGCCA..............................................................................................    0.563734
.................UGCCUAGCUCCCUGUAUGCCA..............................................................................................    1.867975
.................UGCCUGCCUCCCUGUAUGCCA..............................................................................................    27.815727
.................UGCCUGGCACCCUGUAUGCCA..............................................................................................    0.61377
.................UGCCUGGCUCCCUGUAUGA................................................................................................    14.94996
.................UGCCUGGCUCCCUGUAUGU................................................................................................    32.487343
.................UGCCUGGCUCCCUGUAUGCCC..............................................................................................    56.852869
.................UGCCUGGCUCCCUGUAUGCCU..............................................................................................    460.805045
.................UGCCUGGCGCCCUGUAUGCCA..............................................................................................    1.87625
.................UGCCUGGCUCCCUGUAUUCCA..............................................................................................    16.158465
.................UGCCUGGCUCCCUGUCUGCCA..............................................................................................    16.09229
.................UGCCUGGCUCCCAGUAUGCCA..............................................................................................    1.31787
..................GCCUGGCUCCCUGUAUGCCA..............................................................................................    205.100799
...................CCUGGCUCCCUGUAUGCCA..............................................................................................    82.560615
....................CUGGCUCCCUGUAUGCCA..............................................................................................    13.152315
```

zma-MIR160b

Chromosome: chr5
Start: 44296769
End: 44296892
Strand: -
Strand bias: 1.0
Abundance bias: 1.0
MFE: -62.70

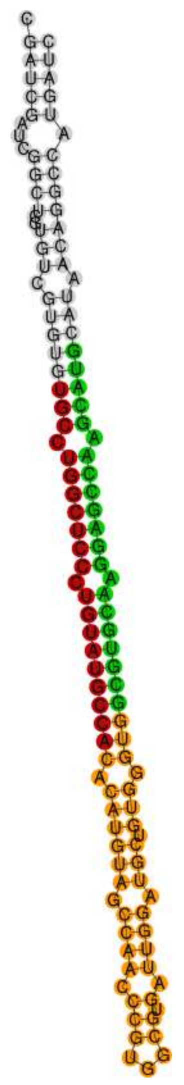

```
                                       Mature                                                      Star
CGAUCGAUCGGCUCGUGUCGUGUGUGCCUGGCUCCCUGUAUGCCACACAUGUAGCCAACCCGUGGCGUGAUUGGAUGCUGUGGGUGGCGUGCAAGGAGCCAAGCAUGCAUAACAGGCCAUGAUC    89802.4
.(((((...((((..(((.((((((((.(((((((.((((((((((.((((((.((((.(((...)).).)))).))).))).)))))))))).))))))).)))))))).))))))).)))))
........................UGCCUGGCUCCCUGUAUGCCA...............................................................................    82649.23842
......................................................................................GCGUGCAAGGAGCCAAGCAUG.................    1276.714909
......................UGUGCCUGGCUCCCUGUAUGCC................................................................................    25.62192
......................UGUGCCUGGCUCCCUGUAUGCU................................................................................    0.925475
.......................GUGCCUGGCUCCCUGUAUGCC................................................................................    59.934456
.......................GUGCCUGGCUCCCUGUAUGCA................................................................................    0.963614
.......................GUGCCUGGCUCCCUGUAUGCU................................................................................    4.02036
........................UGCCUGGCUCCCUGUAUG..................................................................................    240.304981
........................UGCCUGGCUCCCUGUAUGC.................................................................................    715.659827
........................UGCCUGGCUCCCUGUAUGCCAC..............................................................................    26.8834
........................UGCCUGGCUCCCUGUAUGCC................................................................................    1682.996037
........................UGCCUGGCUCCCUGUAUGCCACA.............................................................................    63.53464
........................UGACUGGCUCCCUGUAUGCCA...............................................................................    13.284509
........................UGCCAGGCUCCCUGUAUGCCA...............................................................................    1.681203
........................UGCCUUGCUCCCUGUAUGCCA...............................................................................    14.287606
........................UGCCUGGCUCCCCGUAUGCCA...............................................................................    37.434275
........................UGCCUGGCUCCCUGUACGCCA...............................................................................    74.973683
........................UGCCUGGCUCCCUUUAUGCCA...............................................................................    1.976325
........................UGCUUGGCUCCCUGUAUGCCA...............................................................................    25.084326
........................UGCCUGGCUUCCUGUAUGCCA...............................................................................    10.300446
........................UGCCUGGCUCCCUGUGUGCCA...............................................................................    37.737876
........................AGCCUGGCUCCCUGUAUGCCA...............................................................................    6.183748
........................UACCUGGCUCCCUGUAUGCCA...............................................................................    7.119409
........................UGCCUGGCUCCCUGGAUGCCA...............................................................................    1.96397
........................UGCCUGGCUCCCUGUAUGUCA...............................................................................    50.516819
........................UGCCUGGCUCCCUGUAUGCAA...............................................................................    17.289173
........................UCCCUGGCUCCCUGUAUGCCA...............................................................................    2.746981
........................UUCCUGGCUCCCUGUAUGCCA...............................................................................    35.203458
........................UGCCUGGCUCCCUGUAUGCUA...............................................................................    29.451449
........................UGCCUCGCUCCCUGUAUGCCA...............................................................................    2.703818
........................UGCCUGGCUCCCUGUAUGACA...............................................................................    2.665267
........................UGCCUGGCUCCCUGUAUACCA...............................................................................    486.963489
........................UGCCUGGCUCCCUGUAUUC.................................................................................    13.3184
........................UGCCUGGCUCCCUGAAUGCCA...............................................................................    1.789584
........................UGCCUGGCUCCUUGUAUGCCA...............................................................................    4.518435
........................UGCCUGGCUCCCUGUAUCCCA...............................................................................    6.594481
........................UGCCCGGCUCCCUGUAUGCCA...............................................................................    28.17989
........................UGUCUGGCUCCCUGUAUGCCA...............................................................................    27.670767
........................UGCCUGGCUCCCUGUAUGCA................................................................................    164.345598
........................UGCCUGGCUCCCUGUAUGGCA...............................................................................    0.516788
........................UGCAUGGCUCCCUGUAUGCCA...............................................................................    3.91656
........................CGCCUGGCUCCCUGUAUGCCA...............................................................................    42.828633
........................UGCCUGGCCCCCUGUAUGCCA...............................................................................    42.364152
........................UGCCUGGCUCUCUGUAUGCCA...............................................................................    41.764425
........................UGCCUGGUUCCCUGUAUGCCA...............................................................................    13.914989
........................UGCCUGGCUCCCUGUAUGCCAU..............................................................................    38.086041
........................UGCCUGGCUCCCUGUAUGCCAA..............................................................................    51.844668
........................UGCCUGGCUCCCUGUAUA..................................................................................    4.73897
........................UGCCUGGCUCCCUGUAUC..................................................................................    1.69914
........................UGCCUGGCUCCCUGUAUU..................................................................................    63.701248
........................UGCCUGGCUCCCUGCAUGCCA...............................................................................    27.12702
........................UGCCUGACUCCCUGUAUGCCA...............................................................................    21.856695
........................UGCCUGGCUCCCUGUAUGCG................................................................................    0.72271
........................UGCCUGGCUCCCUGUAUGCU................................................................................    605.924741
........................UGCCUGUCUCCCUGUAUGCCA...............................................................................    39.091006
........................GGCCUGGCUCCCUGUAUGCCA...............................................................................    2.558705
........................UGCCUGGCUCCCUAUAUGCCA...............................................................................    0.563734
........................UGCCUAGCUCCCUGUAUGCCA...............................................................................    1.867975
........................UGCCUGCCUCCCUGUAUGCCA...............................................................................    27.815727
........................UGCCUGGCACCCUGUAUGCCA...............................................................................    0.61377
........................UGCCUGGCUCCCUGUAUGA.................................................................................    14.94996
........................UGCCUGGCUCCCUGUAUGU.................................................................................    32.487343
........................UGCCUGGCUCCCUGUAUGCCC...............................................................................    56.852869
........................UGCCUGGCUCCCUGUAUGCCU...............................................................................    460.805045
........................UGCCUGGCGCCCUGUAUGCCA...............................................................................    1.87625
........................UGCCUGGCUCCCUGUAUUCCA...............................................................................    16.158465
........................UGCCUGGCUCCCUGUCUGCCA...............................................................................    16.09229
........................UGCCUGGCUCCCAGUAUGCCA...............................................................................    1.31787
.........................GCCUGGCUCCCUGUAUGCCA...............................................................................    205.100799
..........................CCUGGCUCCCUGUAUGCCA...............................................................................    82.560615
...........................CUGGCUCCCUGUAUGCCA...............................................................................    13.152315
....................................................................................UGGCGUGCAAGGAGCCAAGCA...................    0.212795
......................................................................................GCGUGCAAGGAGCCAAGCAU..................    1.769547
......................................................................................GCGUGCAAGGAGCCAAGCA...................    0.334392
......................................................................................GCGUGCGAGGAGCCAAGCAUG.................    2.30877
......................................................................................GCGUGUAAGGAGCCAAGCAUG.................    2.09301
......................................................................................GCGUGCAAGGAGUCAAGCAUG.................    1.56976
......................................................................................GCGUGCACGGAGCCAAGCAUG.................    1.56976
......................................................................................GCGUGAAAGGAGCCAAGCAUG.................    1.83139
......................................................................................GCGGGCAAGGAGCCAAGCAUG.................    2.85964
.......................................................................................CGUGCAAGGAGCCAAGCAUG.................    0.273594
```

zma-MIR160c

Chromosome: chr6
Start: 9551851
End: 9551970
Strand: +
Strand bias: 1.0
Abundance bias: 1.0
MFE: -50.50

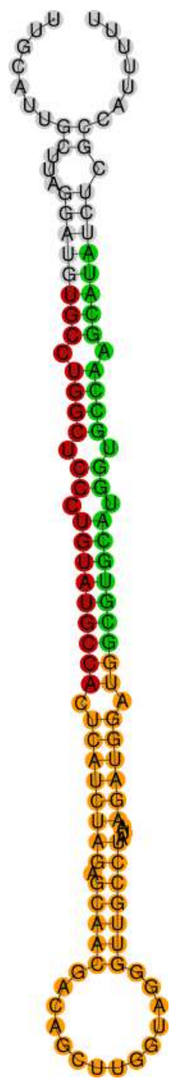

```
                  Mature                                                              Star
UUGCAUUGCUUAGGAUGUGCCUGGCUCCCUGUAUGCCACUCAUCUAGAGCAACGACAGCUUGGUAGGGUUGCCUAUAAGAUGGAUGGCGUGCAUGGUGCCAAGCAUAUCUCGCCAUUUUU    88447.6
.......((...((((((((.((((.((.(((((((((.((((((((.(((((..............)))))))...)))))).))))))))).)).)))).)))))))).)).......
.................UGCCUGGCUCCCUGUAUGCCA..................................................................................    82649.23842
......................................................................................GCGUGCAUGGUGCCAAGCAUA.............    0.212795
...............UGUGCCUGGCUCCCUGUAUGCC...................................................................................    25.62192
...............UGUGCCUGGCUCCCUGUAUGCU...................................................................................    0.925475
................GUGCCUGGCUCCCUGUAUGCC...................................................................................    59.934456
................GUGCCUGGCUCCCUGUAUGCA...................................................................................    0.963614
................GUGCCUGGCUCCCUGUAUGCU...................................................................................    4.02036
.................UGCCUGGCUCCCUGUAUG.....................................................................................    240.304981
.................UGCCUGGCUCCCUGUAUGC....................................................................................    715.659827
.................UGCCUGGCUCCCUGUAUGCCAC................................................................................    26.8834
.................UGCCUGGCUCCCUGUAUGCC...................................................................................    1682.996037
.................UGACUGGCUCCCUGUAUGCCA..................................................................................    13.284509
.................UGCCAGGCUCCCUGUAUGCCA..................................................................................    1.681203
.................UGCCUUGCUCCCUGUAUGCCA..................................................................................    14.287606
.................UGCCUGGCUCCCCGUAUGCCA..................................................................................    37.434275
.................UGCCUGGCUCCCUGUACGCCA..................................................................................    74.973683
.................UGCCUGGCUCCCUUUAUGCCA..................................................................................    1.976325
.................UGCUUGGCUCCCUGUAUGCCA..................................................................................    25.084326
.................UGCCUGGCUUCCUGUAUGCCA..................................................................................    10.300446
.................UGCCUGGCUCCCUGUGUGCCA..................................................................................    37.737876
.................AGCCUGGCUCCCUGUAUGCCA..................................................................................    6.183748
.................UACCUGGCUCCCUGUAUGCCA..................................................................................    7.119409
.................UGCCUGGCUCCCUGGAUGCCA..................................................................................    1.96397
.................UGCCUGGCUCCCUGUAUGUCA..................................................................................    50.516819
.................UGCCUGGCUCCCUGUAUGCAA..................................................................................    17.289173
.................UCCCUGGCUCCCUGUAUGCCA..................................................................................    2.746981
.................UUCCUGGCUCCCUGUAUGCCA..................................................................................    35.203458
.................UGCCUGGCUCCCUGUAUGCUA..................................................................................    29.451449
.................UGCCUCGCUCCCUGUAUGCCA..................................................................................    2.703818
.................UGCCUGGCUCCCUGUAUGACA..................................................................................    2.665267
.................UGCCUGGCUCCCUGUAUACCA..................................................................................    486.963489
.................UGCCUGGCUCCCUGUAUUC....................................................................................    13.3184
.................UGCCUGGCUCCCUGAAUGCCA..................................................................................    1.789584
.................UGCCUGGCUCCUUGUAUGCCA..................................................................................    4.518435
.................UGCCUGGCUCCCUGUAUCCCA..................................................................................    6.594481
.................UGCCCGGCUCCCUGUAUGCCA..................................................................................    28.17989
.................UGUCUGGCUCCCUGUAUGCCA..................................................................................    27.670767
.................UGCCUGGCUCCCUGUAUGCA...................................................................................    164.345598
.................UGCCUGGCUCCCUGUAUGGCA..................................................................................    0.516788
.................UGCAUGGCUCCCUGUAUGCCA..................................................................................    3.91656
.................CGCCUGGCUCCCUGUAUGCCA..................................................................................    42.828633
.................UGCCUGGCCCCCUGUAUGCCA..................................................................................    42.364152
.................UGCCUGGCUCUCUGUAUGCCA..................................................................................    41.764425
.................UGCCUGGUUCCCUGUAUGCCA..................................................................................    13.914989
.................UGCCUGGCUCCCUGUAUGCCAU.................................................................................    38.086041
.................UGCCUGGCUCCCUGUAUGCCAA.................................................................................    51.844668
.................UGCCUGGCUCCCUGUAUA.....................................................................................    4.73897
.................UGCCUGGCUCCCUGUAUC.....................................................................................    1.69914
.................UGCCUGGCUCCCUGUAUU.....................................................................................    63.701248
.................UGCCUGGCUCCCUGCAUGCCA..................................................................................    27.12702
.................UGCCUGACUCCCUGUAUGCCA..................................................................................    21.856695
.................UGCCUGGCUCCCUGUAUGCG...................................................................................    0.72271
.................UGCCUGGCUCCCUGUAUGCU...................................................................................    605.924741
.................UGCCUGUCUCCCUGUAUGCCA..................................................................................    39.091006
.................GGCCUGGCUCCCUGUAUGCCA..................................................................................    2.558705
.................UGCCUGGCUCCCUAUAUGCCA..................................................................................    0.563734
.................UGCCUAGCUCCCUGUAUGCCA..................................................................................    1.867975
.................UGCCUGCCUCCCUGUAUGCCA..................................................................................    27.815727
.................UGCCUGGCACCCUGUAUGCCA..................................................................................    0.61377
.................UGCCUGGCUCCCUGUAUGA....................................................................................    14.94996
.................UGCCUGGCUCCCUGUAUGU....................................................................................    32.487343
.................UGCCUGGCUCCCUGUAUGCCC..................................................................................    56.852869
.................UGCCUGGCUCCCUGUAUGCCU..................................................................................    460.805045
.................UGCCUGGCGCCCUGUAUGCCA..................................................................................    1.87625
.................UGCCUGGCUCCCUGUAUUCCA..................................................................................    16.158465
.................UGCCUGGCUCCCUGUCUGCCA..................................................................................    16.09229
.................UGCCUGGCUCCCAGUAUGCCA..................................................................................    1.31787
..................GCCUGGCUCCCUGUAUGCCA..................................................................................    205.100799
...................CCUGGCUCCCUGUAUGCCA..................................................................................    82.560615
....................CUGGCUCCCUGUAUGCCA..................................................................................    13.152315
```

zma-MIR160d

```
Chromosome:    chr1
Start:         288700632
End:           288700773
Strand:        -
Strand bias:   1.0
Abundance bias:1.0
MFE:           -68.80
```

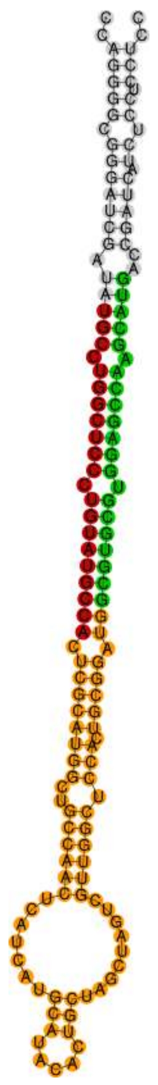

```
                  Mature                                                                         Star
CCAGGGGCGGGAUCGAUAUGCCUGGCUCCCUGUAUGCCACUCGCAUGGCUGCCAACUCAUCAUGCAUACACUGCUAGCUAGUCGUUGGCUCCACUGCGGAUGGCGUGCGUGGAGCCAAGCAUGACCGAUCAUCUCCUCCUCC    88985.0
..(((((.(((((((.(((((.(((((((.(((((((((.((((((((..((((((.......(((.....))).........)))))).))).))))).))))))))).))))))).)))))..))))).)).)).)))..
..................UGCCUGGCUCCCUGUAUGCCA.......................................................................................................    82649.23842
......................................................................................................GCGUGCGUGGAGCCAAGCAUG...................    1.29681
.................AUGCCUGGCUCCCUGUAUGC.........................................................................................................    7.57396
.................AUGCCUGGCUCCCUGUAUGCCA.......................................................................................................    305.794103
.................AUGCCUGGCUCCCUGUAUGCC........................................................................................................    312.148988
..................UGCCUGGCUCCCUGUAUG..........................................................................................................    240.304981
..................UGCCUGGCUCCCUGUAUGC.........................................................................................................    715.659827
..................UGCCUGGCUCCCUGUAUGCCAC......................................................................................................    26.8834
..................UGCCUGGCUCCCUGUAUGCC........................................................................................................    1682.996037
..................UGACUGGCUCCCUGUAUGCCA.......................................................................................................    13.284509
..................UGCCAGGCUCCCUGUAUGCCA.......................................................................................................    1.681203
..................UGCCUUGCUCCCUGUAUGCCA.......................................................................................................    14.287606
..................UGCCUGGCUCCCCGUAUGCCA.......................................................................................................    37.434275
..................UGCCUGGCUCCCUGUACGCCA.......................................................................................................    74.973683
..................UGCCUGGCUCCCUUUAUGCCA.......................................................................................................    1.976325
..................UGCUUGGCUCCCUGUAUGCCA.......................................................................................................    25.084326
..................UGCCUGGCUUCCUGUAUGCCA.......................................................................................................    10.300446
..................UGCCUGGCUCCCUGUGUGCCA.......................................................................................................    37.737876
..................AGCCUGGCUCCCUGUAUGCCA.......................................................................................................    6.183748
..................UACCUGGCUCCCUGUAUGCCA.......................................................................................................    7.119409
..................UGCCUGGCUCCCUGGAUGCCA.......................................................................................................    1.96397
..................UGCCUGGCUCCCUGUAUGUCA.......................................................................................................    50.516819
..................UGCCUGGCUCCCUGUAUGCAA.......................................................................................................    17.289173
..................UCCCUGGCUCCCUGUAUGCCA.......................................................................................................    2.746981
..................UUCCUGGCUCCCUGUAUGCCA.......................................................................................................    35.203458
..................UGCCUGGCUCCCUGUAUGCUA.......................................................................................................    29.451449
..................UGCCUCGCUCCCUGUAUGCCA.......................................................................................................    2.703818
..................UGCCUGGCUCCCUGUAUGACA.......................................................................................................    2.665267
..................UGCCUGGCUCCCUGUAUACCA.......................................................................................................    486.963489
..................UGCCUGGCUCCCUGUAUUC.........................................................................................................    13.3184
..................UGCCUGGCUCCCUGAAUGCCA.......................................................................................................    1.789584
..................UGCCUGGCUCCUUGUAUGCCA.......................................................................................................    4.518435
..................UGCCUGGCUCCCUGUAUCCCA.......................................................................................................    6.594481
..................UGCCCGGCUCCCUGUAUGCCA.......................................................................................................    28.17989
..................UGUCUGGCUCCCUGUAUGCCA.......................................................................................................    27.670767
..................UGCCUGGCUCCCUGUAUGCA........................................................................................................    164.345598
..................UGCCUGGCUCCCUGUAUGGCA.......................................................................................................    0.516788
..................UGCAUGGCUCCCUGUAUGCCA.......................................................................................................    3.91656
..................CGCCUGGCUCCCUGUAUGCCA.......................................................................................................    42.828633
..................UGCCUGGCCCCCUGUAUGCCA.......................................................................................................    42.364152
..................UGCCUGGCUCUCUGUAUGCCA.......................................................................................................    41.764425
..................UGCCUGGUUCCCUGUAUGCCA.......................................................................................................    13.914989
..................UGCCUGGCUCCCUGUAUGCCAU......................................................................................................    38.086041
..................UGCCUGGCUCCCUGUAUGCCAA......................................................................................................    51.844668
..................UGCCUGGCUCCCUGUAUA..........................................................................................................    4.73897
..................UGCCUGGCUCCCUGUAUC..........................................................................................................    1.69914
..................UGCCUGGCUCCCUGUAUU..........................................................................................................    63.701248
..................UGCCUGGCUCCCUGCAUGCCA.......................................................................................................    27.12702
..................UGCCUGACUCCCUGUAUGCCA.......................................................................................................    21.856695
..................UGCCUGGCUCCCUGUAUGCG........................................................................................................    0.72271
..................UGCCUGGCUCCCUGUAUGCU........................................................................................................    605.924741
..................UGCCUGUCUCCCUGUAUGCCA.......................................................................................................    39.091006
..................GGCCUGGCUCCCUGUAUGCCA.......................................................................................................    2.558705
..................UGCCUGGCUCCCUAUAUGCCA.......................................................................................................    0.563734
..................UGCCUAGCUCCCUGUAUGCCA.......................................................................................................    1.867975
..................UGCCUGCCUCCCUGUAUGCCA.......................................................................................................    27.815727
..................UGCCUGGCACCCUGUAUGCCA.......................................................................................................    0.61377
..................UGCCUGGCUCCCUGUAUGA.........................................................................................................    14.94996
..................UGCCUGGCUCCCUGUAUGU.........................................................................................................    32.487343
..................UGCCUGGCUCCCUGUAUGCCC.......................................................................................................    56.852869
..................UGCCUGGCUCCCUGUAUGCCU.......................................................................................................    460.805045
..................UGCCUGGCGCCCUGUAUGCCA.......................................................................................................    1.87625
..................UGCCUGGCUCCCUGUAUUCCA.......................................................................................................    16.158465
..................UGCCUGGCUCCCUGUCUGCCA.......................................................................................................    16.09229
..................UGCCUGGCUCCCAGUAUGCCA.......................................................................................................    1.31787
...................GCCUGGCUCCCUGUAUGCCA.......................................................................................................    205.100799
....................CCUGGCUCCCUGUAUGCCA.......................................................................................................    82.560615
.....................CUGGCUCCCUGUAUGCCA.......................................................................................................    13.152315
......................................................................................................GCGUGCGAGGAGCCAAGCAUG...................    2.30877
```

zma-MIR160g

Chromosome: chr6
Start: 94036683
End: 94036811
Strand: +
Strand bias: 1.0
Abundance bias: 1.0
MFE: -55.40

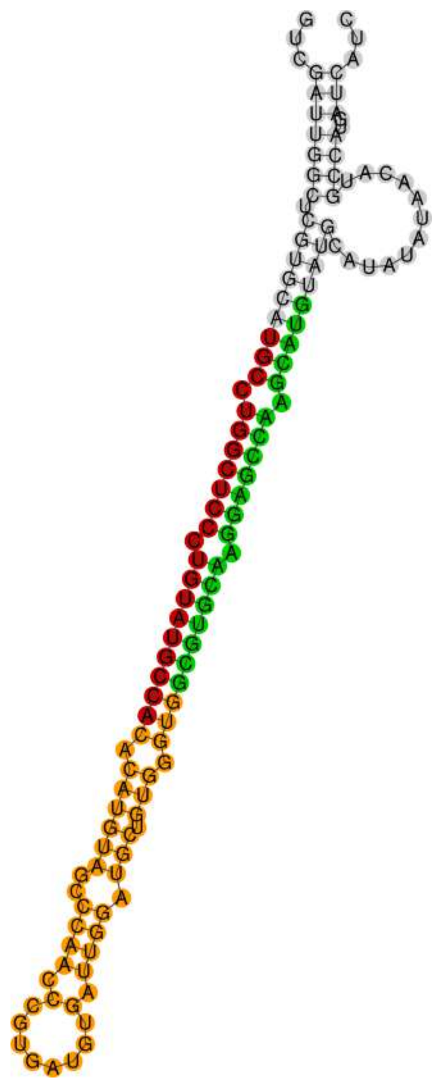

```
                     Mature                                                   Star
GUCGAUUGGCUCGUGCAUGCCUGGCUCCCUGUAUGCCACACAUGUAGCCCAACCCGUGAUGUGAUUGGAUGCUGUGGGUGGCGUGCAAGGAGCCAAGCAUGUAUGCAUAUAUAACAUGCCAUGAUCAUC    90338.4
...(((((((.((((((((.(((((((.((((((((((.((((((..((((.(........).)))).))).))).))))))))))).)))))))).)))))))))............))))..)))...
.................UGCCUGGCUCCCUGUAUGCCA...........................................................................................    82649.23842
................................................................................GCGUGCAAGGAGCCAAGCAUG............................    1276.714909
...............CAUGCCUGGCUCCCUGUAUGCCA...........................................................................................    1.95174
................AUGCCUGGCUCCCUGUAUGC.............................................................................................    7.57396
................AUGCCUGGCUCCCUGUAUGCCA...........................................................................................    305.794103
................AUGCCUGGCUCCCUGUAUGCC............................................................................................    312.148988
.................UGCCUGGCUCCCUGUAUG..............................................................................................    240.304981
.................UGCCUGGCUCCCUGUAUGC.............................................................................................    715.659827
.................UGCCUGGCUCCCUGUAUGCCAC.........................................................................................    26.8834
.................UGCCUGGCUCCCUGUAUGCC............................................................................................    1682.996037
.................UGCCUGGCUCCCUGUAUGCCACA........................................................................................    63.53464
.................UGACUGGCUCCCUGUAUGCCA...........................................................................................    13.284509
.................UGCCAGGCUCCCUGUAUGCCA...........................................................................................    1.681203
.................UGCCUUGCUCCCUGUAUGCCA...........................................................................................    14.287606
.................UGCCUGGCUCCCCGUAUGCCA...........................................................................................    37.434275
.................UGCCUGGCUCCCUGUACGCCA...........................................................................................    74.973683
.................UGCCUGGCUCCCUUUAUGCCA...........................................................................................    1.976325
.................UGCUUGGCUCCCUGUAUGCCA...........................................................................................    25.084326
.................UGCCUGGCUUCCUGUAUGCCA...........................................................................................    10.300446
.................UGCCUGGCUCCCUGUGUGCCA...........................................................................................    37.737876
.................AGCCUGGCUCCCUGUAUGCCA...........................................................................................    6.183748
.................UACCUGGCUCCCUGUAUGCCA...........................................................................................    7.119409
.................UGCCUGGCUCCCUGGAUGCCA...........................................................................................    1.96397
.................UGCCUGGCUCCCUGUAUGUCA...........................................................................................    50.516819
.................UGCCUGGCUCCCUGUAUGCAA...........................................................................................    17.289173
.................UCCCUGGCUCCCUGUAUGCCA...........................................................................................    2.746981
.................UUCCUGGCUCCCUGUAUGCCA...........................................................................................    35.203458
.................UGCCUGGCUCCCUGUAUGCUA...........................................................................................    29.451449
.................UGCCUCGCUCCCUGUAUGCCA...........................................................................................    2.703818
.................UGCCUGGCUCCCUGUAUGACA...........................................................................................    2.665267
.................UGCCUGGCUCCCUGUAUACCA...........................................................................................    486.963489
.................UGCCUGGCUCCCUGUAUUC.............................................................................................    13.3184
.................UGCCUGGCUCCCUGAAUGCCA...........................................................................................    1.789584
.................UGCCUGGCUCCUUGUAUGCCA...........................................................................................    4.518435
.................UGCCUGGCUCCCUGUAUCCCA...........................................................................................    6.594481
.................UGCCCGGCUCCCUGUAUGCCA...........................................................................................    28.17989
.................UGUCUGGCUCCCUGUAUGCCA...........................................................................................    27.670767
.................UGCCUGGCUCCCUGUAUGCA............................................................................................    164.345598
.................UGCCUGGCUCCCUGUAUGGCA...........................................................................................    0.516788
.................UGCAUGGCUCCCUGUAUGCCA...........................................................................................    3.91656
.................CGCCUGGCUCCCUGUAUGCCA...........................................................................................    42.828633
.................UGCCUGGCCCCCUGUAUGCCA...........................................................................................    42.364152
.................UGCCUGGCUCUCUGUAUGCCA...........................................................................................    41.764425
.................UGCCUGGUUCCCUGUAUGCCA...........................................................................................    13.914989
.................UGCCUGGCUCCCUGUAUGCCAU..........................................................................................    38.086041
.................UGCCUGGCUCCCUGUAUGCCAA..........................................................................................    51.844668
.................UGCCUGGCUCCCUGUAUA..............................................................................................    4.73897
.................UGCCUGGCUCCCUGUAUC..............................................................................................    1.69914
.................UGCCUGGCUCCCUGUAUU..............................................................................................    63.701248
.................UGCCUGGCUCCCUGCAUGCCA...........................................................................................    27.12702
.................UGCCUGACUCCCUGUAUGCCA...........................................................................................    21.856695
.................UGCCUGGCUCCCUGUAUGCG............................................................................................    0.72271
.................UGCCUGGCUCCCUGUAUGCU............................................................................................    605.924741
.................UGCCUGUCUCCCUGUAUGCCA...........................................................................................    39.091006
.................GGCCUGGCUCCCUGUAUGCCA...........................................................................................    2.558705
.................UGCCUGGCUCCCUAUAUGCCA...........................................................................................    0.563734
.................UGCCUAGCUCCCUGUAUGCCA...........................................................................................    1.867975
.................UGCCUGCCUCCCUGUAUGCCA...........................................................................................    27.815727
.................UGCCUGGCACCCUGUAUGCCA...........................................................................................    0.61377
.................UGCCUGGCUCCCUGUAUGA.............................................................................................    14.94996
.................UGCCUGGCUCCCUGUAUGU.............................................................................................    32.487343
.................UGCCUGGCUCCCUGUAUGCCC...........................................................................................    56.852869
.................UGCCUGGCUCCCUGUAUGCCU...........................................................................................    460.805045
.................UGCCUGGCGCCCUGUAUGCCA...........................................................................................    1.87625
.................UGCCUGGCUCCCUGUAUUCCA...........................................................................................    16.158465
.................UGCCUGGCUCCCUGUCUGCCA...........................................................................................    16.09229
.................UGCCUGGCUCCCAGUAUGCCA...........................................................................................    1.31787
..................GCCUGGCUCCCUGUAUGCCA...........................................................................................    205.100799
...................CCUGGCUCCCUGUAUGCCA...........................................................................................    82.560615
....................CUGGCUCCCUGUAUGCCA...........................................................................................    13.152315
..............................................................................UGGCGUGCAAGGAGCCAAGCA..............................    0.212795
................................................................................GCGUGCAAGGAGCCAAGCAU.............................    1.769547
................................................................................GCGUGCAAGGAGCCAAGCA..............................    0.334392
................................................................................GCGUGCGAGGAGCCAAGCAUG............................    2.30877
................................................................................GCGUGUAAGGAGCCAAGCAUG............................    2.09301
................................................................................GCGUGCAAGGAGUCAAGCAUG............................    1.56976
................................................................................GCGUGCACGGAGCCAAGCAUG............................    1.56976
................................................................................GCGUGAAAGGAGCCAAGCAUG............................    1.83139
................................................................................GCGGGCAAGGAGCCAAGCAUG............................    2.85964
.................................................................................CGUGCAAGGAGCCAAGCAUG............................    0.273594
```

zma-MIR160_N1

Chromosome: chr7
Start: 5206352
End: 5206472
Strand: -
Strand bias: 1.0
Abundance bias: 0.998
MFE: -60.10

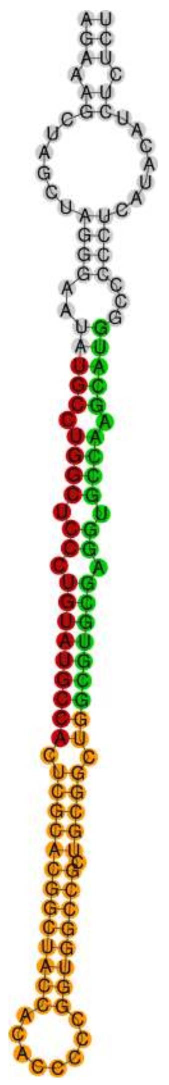

```
                    Mature                                   Star
AGAAAGCUAGCUAGGGAAUAUGCCUGGCUCCCUGUAUGCCACUCGCACGGCUACCACACCCCCGGUGGCCGCUGCGGCUGGCGUGCGAGGUGCCAAGCAUGGCCCCCUCAUACAUCUCUCU    89729.4
(((.((......((((..(((((.((((.((.(((((((((.(((((((((((((........))))))))).))))).))))))))).)).)))).)))))...)))).......)).)))
....................UGCCUGGCUCCCUGUAUGCCA................................................................................    82649.23842
................................................................................GCGUGCGAGGUGCCAAGCAUG....................    113.504293
...................AUGCCUGGCUCCCUGUAUGC..................................................................................    7.57396
...................AUGCCUGGCUCCCUGUAUGCCA................................................................................    305.794103
...................AUGCCUGGCUCCCUGUAUGCC.................................................................................    312.148988
....................UGCCUGGCUCCCUGUAUG...................................................................................    240.304981
....................UGCCUGGCUCCCUGUAUGC..................................................................................    715.659827
....................UGCCUGGCUCCCUGUAUGCCAC..............................................................................    26.8834
....................UGCCUGGCUCCCUGUAUGCC.................................................................................    1682.996037
....................UGACUGGCUCCCUGUAUGCCA................................................................................    13.284509
....................UGCCAGGCUCCCUGUAUGCCA................................................................................    1.681203
....................UGCCUUGCUCCCUGUAUGCCA................................................................................    14.287606
....................UGCCUGGCUCCCCGUAUGCCA................................................................................    37.434275
....................UGCCUGGCUCCCUGUACGCCA................................................................................    74.973683
....................UGCCUGGCUCCCUUUAUGCCA................................................................................    1.976325
....................UGCUUGGCUCCCUGUAUGCCA................................................................................    25.084326
....................UGCCUGGCUUCCUGUAUGCCA................................................................................    10.300446
....................UGCCUGGCUCCCUGUGUGCCA................................................................................    37.737876
....................AGCCUGGCUCCCUGUAUGCCA................................................................................    6.183748
....................UACCUGGCUCCCUGUAUGCCA................................................................................    7.119409
....................UGCCUGGCUCCCUGGAUGCCA................................................................................    1.96397
....................UGCCUGGCUCCCUGUAUGUCA................................................................................    50.516819
....................UGCCUGGCUCCCUGUAUGCAA................................................................................    17.289173
....................UCCCUGGCUCCCUGUAUGCCA................................................................................    2.746981
....................UUCCUGGCUCCCUGUAUGCCA................................................................................    35.203458
....................UGCCUGGCUCCCUGUAUGCUA................................................................................    29.451449
....................UGCCUCGCUCCCUGUAUGCCA................................................................................    2.703818
....................UGCCUGGCUCCCUGUAUGACA................................................................................    2.665267
....................UGCCUGGCUCCCUGUAUACCA................................................................................    486.963489
....................UGCCUGGCUCCCUGUAUUC..................................................................................    13.3184
....................UGCCUGGCUCCCUGAAUGCCA................................................................................    1.789584
....................UGCCUGGCUCCUUGUAUGCCA................................................................................    4.518435
....................UGCCUGGCUCCCUGUAUCCCA................................................................................    6.594481
....................UGCCCGGCUCCCUGUAUGCCA................................................................................    28.17989
....................UGUCUGGCUCCCUGUAUGCCA................................................................................    27.670767
....................UGCCUGGCUCCCUGUAUGCA.................................................................................    164.345598
....................UGCCUGGCUCCCUGUAUGGCA................................................................................    0.516788
....................UGCAUGGCUCCCUGUAUGCCA................................................................................    3.91656
....................CGCCUGGCUCCCUGUAUGCCA................................................................................    42.828633
....................UGCCUGGCCCCCUGUAUGCCA................................................................................    42.364152
....................UGCCUGGCUCUCUGUAUGCCA................................................................................    41.764425
....................UGCCUGGUUCCCUGUAUGCCA................................................................................    13.914989
....................UGCCUGGCUCCCUGUAUGCCAU...............................................................................    38.086041
....................UGCCUGGCUCCCUGUAUGCCAA...............................................................................    51.844668
....................UGCCUGGCUCCCUGUAUA...................................................................................    4.73897
....................UGCCUGGCUCCCUGUAUC...................................................................................    1.69914
....................UGCCUGGCUCCCUGUAUU...................................................................................    63.701248
....................UGCCUGGCUCCCUGCAUGCCA................................................................................    27.12702
....................UGCCUGACUCCCUGUAUGCCA................................................................................    21.856695
....................UGCCUGGCUCCCUGUAUGCG.................................................................................    0.72271
....................UGCCUGGCUCCCUGUAUGCU.................................................................................    605.924741
....................UGCCUGUCUCCCUGUAUGCCA................................................................................    39.091006
....................GGCCUGGCUCCCUGUAUGCCA................................................................................    2.558705
....................UGCCUGGCUCCCUAUAUGCCA................................................................................    0.563734
....................UGCCUAGCUCCCUGUAUGCCA................................................................................    1.867975
....................UGCCUGCCUCCCUGUAUGCCA................................................................................    27.815727
....................UGCCUGGCACCCUGUAUGCCA................................................................................    0.61377
....................UGCCUGGCUCCCUGUAUGA..................................................................................    14.94996
....................UGCCUGGCUCCCUGUAUGU..................................................................................    32.487343
....................UGCCUGGCUCCCUGUAUGCCC................................................................................    56.852869
....................UGCCUGGCUCCCUGUAUGCCU................................................................................    460.805045
....................UGCCUGGCGCCCUGUAUGCCA................................................................................    1.87625
....................UGCCUGGCUCCCUGUAUUCCA................................................................................    16.158465
....................UGCCUGGCUCCCUGUCUGCCA................................................................................    16.09229
....................UGCCUGGCUCCCAGUAUGCCA................................................................................    1.31787
.....................GCCUGGCUCCCUGUAUGCCA................................................................................    205.100799
......................CCUGGCUCCCUGUAUGCCA................................................................................    82.560615
.......................CUGGCUCCCUGUAUGCCA................................................................................    13.152315
................................................................................GCGUGCGAGGUGCCAAGC.......................    7.57396
................................................................................GCGUGCGAGGUGCCAAGCAUGGC..................    1.95174
................................................................................GCGUGCGAGGUGCCAAGCA......................    21.09859
................................................................................GCGUGCGAGGUGCCAAGCAUGG...................    455.149668
................................................................................GCGUGCGAGGAGCCAAGCAUG....................    2.30877
................................................................................GCGUGCGAGGUGCCAAGCAUGA...................    2.92761
```

zma-MIR162

Chromosome: chr5
Start: 186614101
End: 186614266
Strand: +
Strand bias: 1.0
Abundance bias:0.997
MFE: -63.40

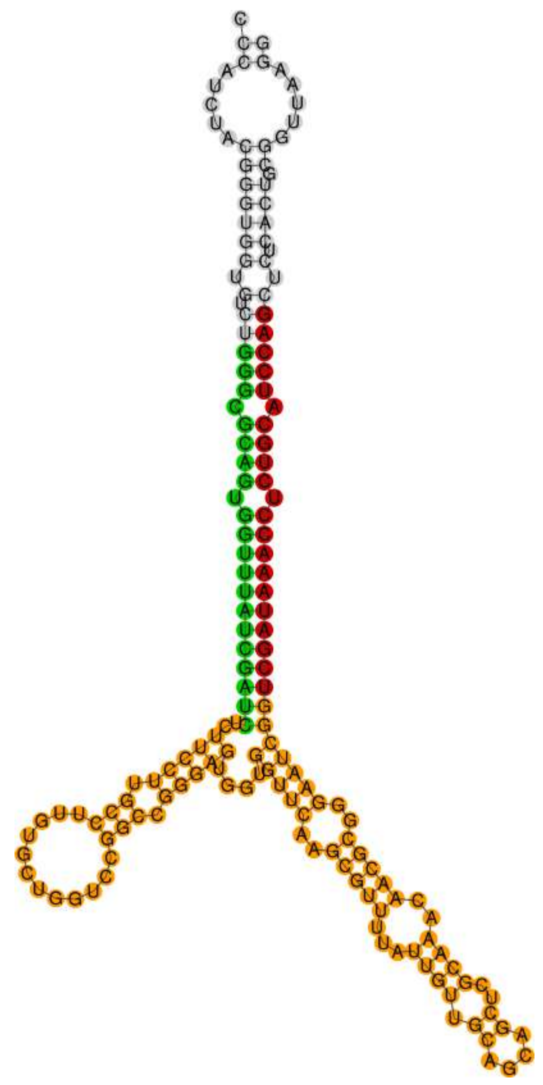

```
                    Star                                                                                                              Mature
CCCAUCUACGGGUGGUGUCUGGGCGCAGUGGUUUAUCGAUCUCUUCCUUGCCUUGUGCUGGUCCGGCCGGGAGUGGUGGUUCAAGCGUUUUAUUGUUGCAGCAGCUCGCAAACAACGCGGGAAUCGGUCGAUAAACCUCUGCAUCCAGCUCUCACUGCGGUUAAGG    28046.8
.((.....(((((((.(.(((((.((((.((((((((((((..(((((.(((............))).)))))....(((((..(((((...((((.((....))..))))..)))))..))))))))))))))))).)))).)))))).).)))).)).....))
...............................................................................................................................UCGAUAAACCUCUGCAUCCAG..................    22344.00719
....................GGGCGCAGUGGUUUAUCGAUC.............................................................................................................................    18.920508
.................................................................................................................................GAUAAACCUCUGCAUCCAG..................    2.903311
................................................................................................................................CGAUAAACCUCUGCAUCCAG..................    76.950137
................................................................................................................................CGAUAAACCUCUGCAUCCAGC.................    5.840006
...............................................................................................................................UCGAUAAACCUCUGCAUC.....................    200.87959
...............................................................................................................................UCGAUAAACCUCUGCAUCCAGC.................    978.563689
...............................................................................................................................UCGAUAAACCUCUGCAUCCA...................    83.867504
...............................................................................................................................UCGAUAAACCUCUGCAUCC....................    1396.090159
...............................................................................................................................UCGAUAAACCUCUGCAUCCUG..................    5.95704
...............................................................................................................................UGGAUAAACCUCUGCAUCCAG..................    0.72271
...............................................................................................................................UCGAUAAACCUCUUCAUCCAG..................    2.633194
...............................................................................................................................UCGAUAAACCUCUGCAUUCAG..................    9.40806
...............................................................................................................................UCGAUAAACCUCUGCCUCCAG..................    13.45613
...............................................................................................................................UCGAUAAACCUCUGCACCCAG..................    4.02659
...............................................................................................................................UCGAUAGACCUCUGCAUCCAG..................    3.080174
...............................................................................................................................UCGAUAAACCUCUGCAUCCCG..................    0.703221
...............................................................................................................................UCGAUAAACCUCUGAAUCCAG..................    1.44062
...............................................................................................................................UCGAUAAACCUCUGCAUCCAC..................    26.03317
...............................................................................................................................UCGAUAAACCUCUGCAUCCAU..................    111.769548
...............................................................................................................................ACGAUAAACCUCUGCAUCCAG..................    0.72271
...............................................................................................................................UCGAUAAACCUCUGCGUCCAG..................    11.562466
...............................................................................................................................UCGAUAAACCUCUGCAUCCAGG.................    0.934938
...............................................................................................................................UCGAUAAAUCUCUGCAUCCAG..................    7.09654
...............................................................................................................................UCGAUAAACCUCUGGAUCCAG..................    1.23482
...............................................................................................................................UCGAUAAACCUCUGUAUCCAG..................    4.858584
...............................................................................................................................UCGAUAAACCUUUGCAUCCAG..................    0.703221
...............................................................................................................................UAGAUAAACCUCUGCAUCCAG..................    3.179074
...............................................................................................................................UCGAUAAACCUAUGCAUCCAG..................    4.35243
...............................................................................................................................UCGAUAAACCUCUGCAUU.....................    25.618721
...............................................................................................................................UCGAUAAACCUCUGCAUCCGG..................    36.380777
...............................................................................................................................UCGAUAAACUUCUGCAUCCAG..................    1.28924
...............................................................................................................................UCGAUAAACAUCUGCAUCCAG..................    4.364461
...............................................................................................................................UCGAUAAACCCCUGCAUCCAG..................    9.20688
...............................................................................................................................UCGAUAAACCUCUGCAUCUAG..................    2.55506
...............................................................................................................................UUGAUAAACCUCUGCAUCCAG..................    14.377126
...............................................................................................................................UCGAUAAACCUCUGCAUCCAA..................    1243.066795
...............................................................................................................................UCGAUAAACCUCUGCAUCCAGA.................    168.971048
...............................................................................................................................UCGAUAAACCUCUGCAUCCAGU.................    246.221486
...............................................................................................................................UCGAUACACCUCUGCAUCCAG..................    5.266451
...............................................................................................................................UCGAUAACCCUCUGCAUCCAG..................    1.26157
...............................................................................................................................UCUAUAAACCUCUGCAUCCAG..................    12.91619
...............................................................................................................................UCGGUAAACCUCUGCAUCCAG..................    10.856285
...............................................................................................................................UCGCUAAACCUCUGCAUCCAG..................    8.366844
...............................................................................................................................UCAAUAAACCUCUGCAUCCAG..................    0.820425
...............................................................................................................................UCGAUAAACCUCUGCAGCCAG..................    0.963614
...............................................................................................................................UCGAUAAACCUCUACAUCCAG..................    1.267108
...............................................................................................................................UCGAUAAUCCUCUGCAUCCAG..................    0.843162
...............................................................................................................................UCGAUAAGCCUCUGCAUCCAG..................    15.641131
...............................................................................................................................UCGAUGAACCUCUGCAUCCAG..................    3.639054
...............................................................................................................................UCGAUAAACCUCUGCAUCAAG..................    15.31144
...............................................................................................................................UCGAUAAACCUCUGCAUCA....................    176.124124
...............................................................................................................................UCGAUAAACCUCUGCAUCG....................    2.375558
...............................................................................................................................UCGAUAAACCUCUGCAUCU....................    601.08127
...............................................................................................................................CCGAUAAACCUCUGCAUCCAG..................    8.185388
...............................................................................................................................UCGAUAAACCUCUGCAUACAG..................    2.534911
...............................................................................................................................UCGAUCAACCUCUGCAUCCAG..................    0.86284
...............................................................................................................................UCGAUAAACCUCUGCAUUC....................    1.46894
...............................................................................................................................UCGAUAAACCUCCGCAUCCAG..................    16.04381
..................UGGGCGCAGUGGUUUAUCGAUC..............................................................................................................................    2.782856
..................UGGGCGCAGUGGUUUAUCGAU...............................................................................................................................    0.243195
```

zma-MIR164a

Chromosome: chr2
Start: 223401157
End: 223401308
Strand: -
Strand bias: 1.0
Abundance bias: 1.0
MFE: -67.50

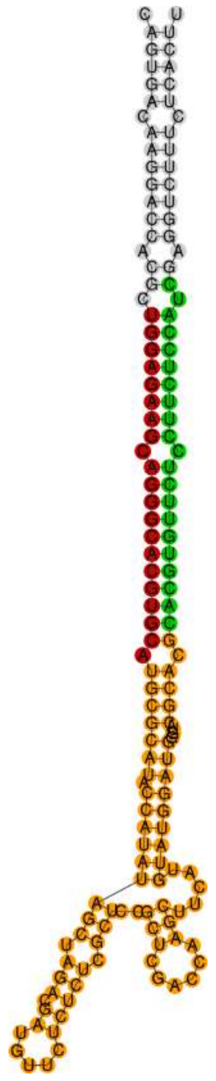

```
                          Mature                                                                                  Star
CAGUGACAAGGACCACGCUGGAGAAGCAGGGCACGUGCAUGCGCAUACCAUAUAGCUAGACGAUGUUCUCUCUCGCUCCGCUCGACCAAGCUUCAUGUAUGGAUGGGUACGCACGCACGUGUUCUCCUUCUCCAUCGAGGUCUUUCUCACUU    71470.9
.(((((.(((((((.((.((((((((.((((((((((.(((((((.(((((((((.(((.((.....))))).)))..(((......))).....)))))))))....)))).))))))))))).)))))))).)).))))))).))))).
..................UGGAGAAGCAGGGCACGUGCA.................................................................................................................    61695.51887
...................................................................................................................CACGUGUUCUCCUUCUCCAUC................    4.87148
.................CUGGAGAAGCAGGGCACGUGC..................................................................................................................    21.374255
.................CUGGAGAAGCAGGGCACGUGCA.................................................................................................................    25.93005
..................UGGAGAAGCAGGGCACGUGCAUG...............................................................................................................    2.267772
..................UGGAGAAGCAGGGCACGU....................................................................................................................    164.379322
..................UGGAGAAGCAGGGCACGUG...................................................................................................................    489.534144
..................UGGAGAAGCAGGGCACGUGC..................................................................................................................    3089.04901
..................UGGAGAAGCAGGGCACGUGCAU................................................................................................................    298.390444
..................UGGAGAAGCAGGGCACCUGCA.................................................................................................................    35.30953
..................UGGAGAAGCAGGGCAGGUGCA.................................................................................................................    2.921215
..................UGGAGAAGCAGCGCACGUGCA.................................................................................................................    48.68017
..................UGGGGAAGCAGGGCACGUGCA.................................................................................................................    67.470451
..................UGGAGAAGCAGGGCACGUGA..................................................................................................................    175.819208
..................UGGAGAAGCAGGGCACGUGG..................................................................................................................    3.6078
..................UGGAGAAGCAAGGCACGUGCA.................................................................................................................    16.503446
..................UGGAGAAGCAGGGCACGUGGA.................................................................................................................    9.06684
..................UGGAGAAGCAGGGCACGUGCG.................................................................................................................    368.762493
..................UUGAGAAGCAGGGCACGUGCA.................................................................................................................    12.476702
..................UGGAGAAGCAGUGCACGUGCA.................................................................................................................    47.074189
..................UGGAGAUGCAGGGCACGUGCA.................................................................................................................    3.82874
..................UGGAGAAGCAGGGCAAGUGCA.................................................................................................................    39.214026
..................UGGACAAGCAGGGCACGUGCA.................................................................................................................    5.511794
..................UGGAGAAGCGGGGCACGUGCA.................................................................................................................    78.541126
..................UGGAGAACCAGGGCACGUGCA.................................................................................................................    23.729777
..................UGGAGAAGCACGGCACGUGCA.................................................................................................................    27.970371
..................UGGAGAAGCAGGGCGCGUGCA.................................................................................................................    58.533162
..................UGUAGAAGCAGGGCACGUGCA.................................................................................................................    23.529335
..................UGGAGAAGCAGGGCAUGUGCA.................................................................................................................    61.033004
..................UGGAGAAGCAGGUCACGUGCA.................................................................................................................    60.137855
..................UGGAGAAGCAGGGCACGUACA.................................................................................................................    23.845858
..................UGGAGAAGCAGGGCACAUGCA.................................................................................................................    59.845226
..................UGGAGAAGCAGGGCACGUGCC.................................................................................................................    212.266876
..................UGGAGAAGCAUGGCACGUGCA.................................................................................................................    31.219393
..................UAGAGAAGCAGGGCACGUGCA.................................................................................................................    13.489773
..................UGGAGAAGCUGGGCACGUGCA.................................................................................................................    38.45266
..................UGGAGAAGCAGGGCACGUU...................................................................................................................    9.3391
..................UGGAGAAGCAGGGCACGUA...................................................................................................................    3.62316
..................UGGAGAAACAGGGCACGUGCA.................................................................................................................    11.771913
..................UGGAGAAGCAGGGCACGC....................................................................................................................    3.80678
..................AGGAGAAGCAGGGCACGUGCA.................................................................................................................    11.9467
..................UGGAGAAGCAGGCCACGUGCA.................................................................................................................    29.547603
..................UGGAGAAGCAGGGCACGCGCA.................................................................................................................    94.118581
..................UGGAGAAGCAGAGCACGUGCA.................................................................................................................    23.116839
..................UGGAGGAGCAGGGCACGUGCA.................................................................................................................    55.282792
..................UGGAUAAGCAGGGCACGUGCA.................................................................................................................    44.045782
..................UCGAGAAGCAGGGCACGUGCA.................................................................................................................    3.454779
..................UGGAGAAGCAGGGCACGUGUA.................................................................................................................    56.900708
..................UGGAGAAGCAGGGCCCGUGC..................................................................................................................    3.250584
..................UGGAGAAGCAGGGCACGUUCA.................................................................................................................    33.866684
..................UGGAGUAGCAGGGCACGUGCA.................................................................................................................    8.46783
..................UGGAGAAGUAGGGCACGUGCA.................................................................................................................    67.880815
..................UGGAGCAGCAGGGCACGUGCA.................................................................................................................    40.83863
..................UGAAGAAGCAGGGCACGUGCA.................................................................................................................    3.3813
..................UGGAGAAGCAGGGAACGUGCA.................................................................................................................    76.18624
..................UGGAGAAGCAGGGCCCGUGCA.................................................................................................................    149.47749
..................UGGAGAGGCAGGGCACGUGCA.................................................................................................................    47.689179
..................CGGAGAAGCAGGGCACGUGCA.................................................................................................................    42.353063
..................UGGAGAAGCAGGGUACGUGCA.................................................................................................................    53.023948
..................UGGAGAAGCAGGGCACGUGCUU................................................................................................................    2.35464
..................UGGAGAAGCAGGACACGUGCA.................................................................................................................    29.22728
..................UGGAGAAGCAGGGCACGUCCA.................................................................................................................    61.366398
..................UGGAGAAGCAGGGCACGUGCAC................................................................................................................    29.533976
..................UGGAAAAGCAGGGCACGUGCA.................................................................................................................    2.734852
..................UGGAGAAGCAGGGCACGUCC..................................................................................................................    1.83618
..................UGGAGAAGCAGGGCACGGGCA.................................................................................................................    37.73864
..................UGGAGAAGAAGGGCACGUGCA.................................................................................................................    13.989545
..................UGGAGAAUCAGGGCACGUGCA.................................................................................................................    61.363695
..................UGGAGAAGCCGGGCACGUGCA.................................................................................................................    62.712505
..................UGGAGAAGCAGGGCACGAGCA.................................................................................................................    13.329253
..................UGGAGACGCAGGGCACGUGCA.................................................................................................................    49.74311
..................UGGAGAAGCAGGGCACUUGCA.................................................................................................................    47.277932
..................UGGAGAAGCAGGGCUCGUGCA.................................................................................................................    10.16059
..................UGGUGAAGCAGGGCACGUGCA.................................................................................................................    5.57998
..................UGGAGAAGCAGGGCACGUGCAA................................................................................................................    43.0612
..................UGGAGAAGCAGGGCACGUGAA.................................................................................................................    120.73405
..................UGGCGAAGCAGGGCACGUGCA.................................................................................................................    46.76727
...................GGAGAAGCAGGGCACGUGCA.................................................................................................................    2374.791889
...................GGAGAAGCAGGGCACGUG...................................................................................................................    6.55789
...................GGAGAAGCAGGGCACGUGC..................................................................................................................    135.908292
...................GGAGAAGCAGCGCACGUGCA.................................................................................................................    2.89319
...................GGAGAAGCAGGGCACGCGCA.................................................................................................................    8.48668
...................GGAGAAGCAGAGCACGUGCA.................................................................................................................    2.31455
...................GGAGAAGCAGGGUACGUGCA.................................................................................................................    4.24334
...................GGAGAAGCAGGACACGUGCA.................................................................................................................    2.50743
...................GGAGAAGUAGGGCACGUGCA.................................................................................................................    2.31455
...................GGAGAAGCAGGGCACGUGUA.................................................................................................................    2.70031
...................GGAGAAGCAGGGCACGAGCA.................................................................................................................    3.27895
...................GGAGAAGCAGGGCGCGUGCA.................................................................................................................    4.6291
...................GGAGAAGCACGGCACGUGCA.................................................................................................................    7.90804
...................GGAGAAGCAGGGCAUGUGCA.................................................................................................................    7.71517
...................GGAGAAGCAUGGCACGUGCA.................................................................................................................    5.20774
...................GGAGAAGCAGGGCACGUGCG.................................................................................................................    2.70031
...................GGAGAAGCAGGGCAGGUGCA.................................................................................................................    2.89319
...................GGAGAAGCGGGGCACGUGCA.................................................................................................................    4.43622
...................GGAGAAGCAAGGCACGUGCA.................................................................................................................    4.24334
....................GAGAAGCAGGGCACGUGCA.................................................................................................................    6.09992
.....................AGAAGCAGGGCACGUGCA.................................................................................................................    10.28547
```

zma-MIR164c

```
Chromosome:    chr6
Start:         157477822
End:           157478120
Strand:        +
Strand bias:   1.0
Abundance bias:0.946
MFE:           -187.60
```

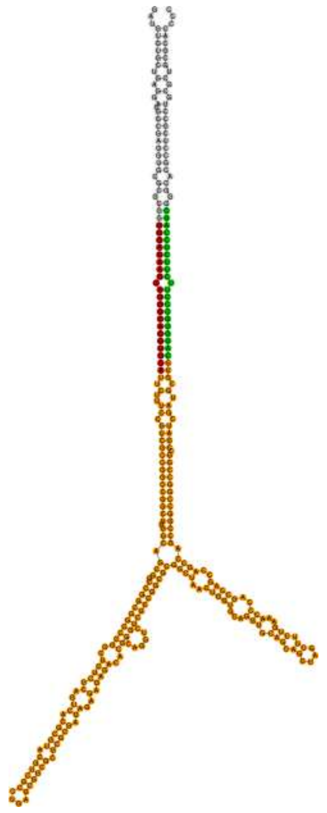

```
                              Mature                                                                                                                                                                                                            Star
GAUGUGUGCUGAGGAUGGCGAGGUGCGCGGUGGAGAAGCAGGGCACGUGCAUUCUUUCCGUCGCCGGCCGGCUUGGCAGCGGCCGGCGGCCCGGCUCUCGCAGUCACGCGUACGUCGCCUGAGCGGCGCGCGAGAGAGAGAGACACGGCAGGUCGUCGCCGGCGCGGCUAACUGGUGCAGGUGCAGCAGCUAGCUUCUGAAACCCAGCCAGCCAGCCAGCCGGCCGGCCGGCCGAUCGAUGCGUGCAUGUGCCCUUCUUCUCCAUCGGGCACGCCUCGCCUGCGCUGCGCACCCC   77680.0
...((((((.(.(.(.(((((((((.((.((((((((((.((((((((((((.(..((.(((((((((((((((.(((.(((.((((((((((..((((.(..((.(((((.((((((....)))))).))))).))..).))))..)))......))))))))))((((..(((((...(((.(((.((....)).)))..)))..)))))..)))).))))))))))))).)))).)).).)))))))))))))).))))))))))).)).)))))))))).).).))))))...
..............................UGGAGAAGCAGGGCACGUGCA....................................................................................................................................................................................................................................................   61695.51887
.....................................................................................................................................................................................................................................................CAUGUGCCCUUCUUCUCCAUC.............................   1630.298321
..............................UGGAGAAGCAGGGCACGU.......................................................................................................................................................................................................................................................   164.379322
..............................UGGAGAAGCAGGGCACGUG......................................................................................................................................................................................................................................................   489.534144
..............................UGGAGAAGCAGGGCACGUGC.....................................................................................................................................................................................................................................................   3089.04901
..............................UGGAGAAGCAGGGCACGUGCAU...................................................................................................................................................................................................................................................   298.390444
..............................UGGAGAAGCAGGGCACCUGCA....................................................................................................................................................................................................................................................   35.30953
..............................UGGAGAAGCAGCGCACGUGCA....................................................................................................................................................................................................................................................   48.68017
..............................UGGGGAAGCAGGGCACGUGCA....................................................................................................................................................................................................................................................   67.470451
..............................UGGAGAAGCAGGGCACGUGA.....................................................................................................................................................................................................................................................   175.819208
..............................UGGAGAAGCAAGGCACGUGCA....................................................................................................................................................................................................................................................   16.503446
..............................UGGAGAAGCAGGGCACGUGCG....................................................................................................................................................................................................................................................   368.762493
..............................UGGAGAAGCAGUGCACGUGCA....................................................................................................................................................................................................................................................   47.074189
..............................UGGAGAAGCAGGGCAAGUGCA....................................................................................................................................................................................................................................................   39.214026
..............................UGGAGAAGCGGGGCACGUGCA....................................................................................................................................................................................................................................................   78.541126
..............................UGGAGAACCAGGGCACGUGCA....................................................................................................................................................................................................................................................   23.729777
..............................UGGAGAAGCACGGCACGUGCA....................................................................................................................................................................................................................................................   27.970371
..............................UGGAGAAGCAGGGCGCGUGCA....................................................................................................................................................................................................................................................   58.533162
..............................UGUAGAAGCAGGGCACGUGCA....................................................................................................................................................................................................................................................   23.529335
..............................UGGAGAAGCAGGGCAUGUGCA....................................................................................................................................................................................................................................................   61.033004
..............................UGGAGAAGCAGGUCACGUGCA....................................................................................................................................................................................................................................................   60.137855
..............................UGGAGAAGCAGGGCACGUACA....................................................................................................................................................................................................................................................   23.845858
..............................UGGAGAAGCAGGGCACAUGCA....................................................................................................................................................................................................................................................   59.845226
..............................UGGAGAAGCAGGGCACGUGCC....................................................................................................................................................................................................................................................   212.266876
..............................UGGAGAAGCAUGGCACGUGCA....................................................................................................................................................................................................................................................   31.219393
..............................UGGAGAAGCUGGGCACGUGCA....................................................................................................................................................................................................................................................   38.45266
..............................UGGAGAAGCAGGCCACGUGCA....................................................................................................................................................................................................................................................   29.547603
..............................UGGAGAAGCAGGGCACGCGCA....................................................................................................................................................................................................................................................   94.118581
..............................UGGAGAAGCAGAGCACGUGCA....................................................................................................................................................................................................................................................   23.116839
..............................UGGAGGAGCAGGGCACGUGCA....................................................................................................................................................................................................................................................   55.282792
..............................UGGAUAAGCAGGGCACGUGCA....................................................................................................................................................................................................................................................   44.045782
..............................UGGAGAAGCAGGGCACGUGUA....................................................................................................................................................................................................................................................   56.900708
..............................UGGAGAAGCAGGGCACGUUCA....................................................................................................................................................................................................................................................   33.866684
..............................UGGAGAAGUAGGGCACGUGCA....................................................................................................................................................................................................................................................   67.880815
..............................UGGAGCAGCAGGGCACGUGCA....................................................................................................................................................................................................................................................   40.83863
..............................UGGAGAAGCAGGGAACGUGCA....................................................................................................................................................................................................................................................   76.18624
..............................UGGAGAAGCAGGGCCCGUGCA....................................................................................................................................................................................................................................................   149.47749
..............................UGGAGAGGCAGGGCACGUGCA....................................................................................................................................................................................................................................................   47.689179
..............................CGGAGAAGCAGGGCACGUGCA....................................................................................................................................................................................................................................................   42.353063
..............................UGGAGAAGCAGGGUACGUGCA....................................................................................................................................................................................................................................................   53.023948
..............................UGGAGAAGCAGGACACGUGCA....................................................................................................................................................................................................................................................   29.22728
..............................UGGAGAAGCAGGGCACGUCCA....................................................................................................................................................................................................................................................   61.366398
..............................UGGAGAAGCAGGGCACGUGCAC...................................................................................................................................................................................................................................................   29.533976
..............................UGGAGAAGCAGGGCACGGGCA....................................................................................................................................................................................................................................................   37.73864
..............................UGGAGAAUCAGGGCACGUGCA....................................................................................................................................................................................................................................................   61.363695
..............................UGGAGAAGCCGGGCACGUGCA....................................................................................................................................................................................................................................................   62.712505
..............................UGGAGACGCAGGGCACGUGCA....................................................................................................................................................................................................................................................   49.74311
..............................UGGAGAAGCAGGGCACUUGCA....................................................................................................................................................................................................................................................   47.277932
..............................UGGAGAAGCAGGGCACGUGCAA...................................................................................................................................................................................................................................................   43.0612
..............................UGGAGAAGCAGGGCACGUGAA....................................................................................................................................................................................................................................................   120.73405
..............................UGGCGAAGCAGGGCACGUGCA....................................................................................................................................................................................................................................................   46.76727
...............................GGAGAAGCAGGGCACGUGCA....................................................................................................................................................................................................................................................   2374.791889
...............................GGAGAAGCAGGGCACGUGC.....................................................................................................................................................................................................................................................   135.908292
................................GAGAAGCAGGGCACGUGCAUU..................................................................................................................................................................................................................................................   16.531614
...................................................................................................................................................................................................................................................UGCAUGUGCCCUUCUUCUCCA...............................   36.108042
.....................................................................................................................................................................................................................................................CAUGUGCCCUUCUUCUCCAUU.............................   58.836506
.....................................................................................................................................................................................................................................................CAUGUGCCCUUCUUUUCCAUC.............................   97.222909
......................................................................................................................................................................................................................................................AUGUGCCCUUCUUCUCCAUC.............................   162.87783
```

zma-MIR164d

Chromosome: chr7
Start: 178154788
End: 178155000
Strand: +
Strand bias: 1.0
Abundance bias: 0.999
MFE: -103.30

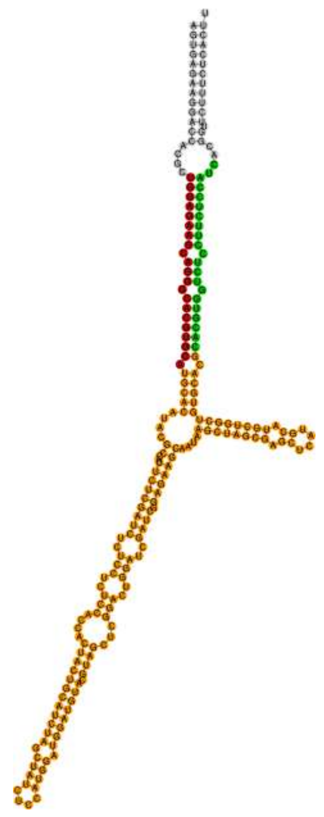

```
                  Mature                                                                                                                                                                    Star
AGUGAGAAGGACCACGCUGGAGAAGCAGGGCACGUGCAUGCACAUACGCCAUUCUCGAUCUCUCCUCUCCACCACUACUGCAUCUAGCUAUCUCCAUGGAUGGAUGUACGUAGCUCGGACUGGAUCGAUCGGAGAAGCAAUAAGCUAGCGAGCUCAUGCAUGCUGGCUGUGCACGCACGUGGUCUCCUUCUCCAUCACGGUUCUUUCUCACUU 71816.9
((((((((((((....((((((((.(((.(((((((.(((((....((..(((((((((..(((..(((....((((((((((((.((((....)))).)))))))).))))...)))..)))..)))).)))))))....((((((((.((....)).))))))))))))).))))))).))).))))))))....)).))))))))))).
.................UGGAGAAGCAGGGCACGUGCA............................................................................................................................................................................... 61695.51887
...............................................................................................................................................................................CACGUGGUCUCCUUCUCCAUC................. 271.238498
................CUGGAGAAGCAGGGCACGUGC................................................................................................................................................................................ 21.374255
................CUGGAGAAGCAGGGCACGUGCA............................................................................................................................................................................... 25.93005
.................UGGAGAAGCAGGGCACGU.................................................................................................................................................................................. 164.379322
.................UGGAGAAGCAGGGCACGUG................................................................................................................................................................................. 489.534144
.................UGGAGAAGCAGGGCACGUGC................................................................................................................................................................................ 3089.04901
.................UGGAGAAGCAGGGCACGUGCAU.............................................................................................................................................................................. 298.390444
.................UGGAGAAGCAGGGCACCUGCA............................................................................................................................................................................... 35.30953
.................UGGAGAAGCAGCGCACGUGCA............................................................................................................................................................................... 48.68017
.................UGGGGAAGCAGGGCACGUGCA............................................................................................................................................................................... 67.470451
.................UGGAGAAGCAGGGCACGUGA................................................................................................................................................................................ 175.819208
.................UGGAGAAGCAAGGCACGUGCA............................................................................................................................................................................... 16.503446
.................UGGAGAAGCAGGGCACGUGCG............................................................................................................................................................................... 368.762493
.................UGGAGAAGCAGUGCACGUGCA............................................................................................................................................................................... 47.074189
.................UGGAGAAGCAGGGCAAGUGCA............................................................................................................................................................................... 39.214026
.................UGGAGAAGCGGGGCACGUGCA............................................................................................................................................................................... 78.541126
.................UGGAGAACCAGGGCACGUGCA............................................................................................................................................................................... 23.729777
.................UGGAGAAGCACGGCACGUGCA............................................................................................................................................................................... 27.970371
.................UGGAGAAGCAGGGCGCGUGCA............................................................................................................................................................................... 58.533162
.................UGUAGAAGCAGGGCACGUGCA............................................................................................................................................................................... 23.529335
.................UGGAGAAGCAGGGCAUGUGCA............................................................................................................................................................................... 61.033004
.................UGGAGAAGCAGGUCACGUGCA............................................................................................................................................................................... 60.137855
.................UGGAGAAGCAGGGCACGUACA............................................................................................................................................................................... 23.845858
.................UGGAGAAGCAGGGCACAUGCA............................................................................................................................................................................... 59.845226
.................UGGAGAAGCAGGGCACGUGCC............................................................................................................................................................................... 212.266876
.................UGGAGAAGCAUGGCACGUGCA............................................................................................................................................................................... 31.219393
.................UAGAGAAGCAGGGCACGUGCA............................................................................................................................................................................... 13.489773
.................UGGAGAAGCUGGGCACGUGCA............................................................................................................................................................................... 38.45266
.................UGGAGAAGCAGGCCACGUGCA............................................................................................................................................................................... 29.547603
.................UGGAGAAGCAGGGCACGCGCA............................................................................................................................................................................... 94.118581
.................UGGAGAAGCAGAGCACGUGCA............................................................................................................................................................................... 23.116839
.................UGGAGGAGCAGGGCACGUGCA............................................................................................................................................................................... 55.282792
.................UGGAUAAGCAGGGCACGUGCA............................................................................................................................................................................... 44.045782
.................UGGAGAAGCAGGGCACGUGUA............................................................................................................................................................................... 56.900708
.................UGGAGAAGCAGGGCACGUUCA............................................................................................................................................................................... 33.866684
.................UGGAGAAGUAGGGCACGUGCA............................................................................................................................................................................... 67.880815
.................UGGAGCAGCAGGGCACGUGCA............................................................................................................................................................................... 40.83863
.................UGGAGAAGCAGGGAACGUGCA............................................................................................................................................................................... 76.18624
.................UGGAGAAGCAGGGCCCGUGCA............................................................................................................................................................................... 149.47749
.................UGGAGAGGCAGGGCACGUGCA............................................................................................................................................................................... 47.689179
.................CGGAGAAGCAGGGCACGUGCA............................................................................................................................................................................... 42.353063
.................UGGAGAAGCAGGGUACGUGCA............................................................................................................................................................................... 53.023948
.................UGGAGAAGCAGGACACGUGCA............................................................................................................................................................................... 29.22728
.................UGGAGAAGCAGGGCACGUCCA............................................................................................................................................................................... 61.366398
.................UGGAGAAGCAGGGCACGUGCAC.............................................................................................................................................................................. 29.533976
.................UGGAGAAGCAGGGCACGGGCA............................................................................................................................................................................... 37.73864
.................UGGAGAAGAAGGGCACGUGCA............................................................................................................................................................................... 13.989545
.................UGGAGAAUCAGGGCACGUGCA............................................................................................................................................................................... 61.363695
.................UGGAGAAGCCGGGCACGUGCA............................................................................................................................................................................... 62.712505
.................UGGAGACGCAGGGCACGUGCA............................................................................................................................................................................... 49.74311
.................UGGAGAAGCAGGGCACUUGCA............................................................................................................................................................................... 47.277932
.................UGGAGAAGCAGGGCACGUGCAA.............................................................................................................................................................................. 43.0612
.................UGGAGAAGCAGGGCACGUGAA............................................................................................................................................................................... 120.73405
.................UGGCGAAGCAGGGCACGUGCA............................................................................................................................................................................... 46.76727
..................GGAGAAGCAGGGCACGUGCA............................................................................................................................................................................... 2374.791889
..................GGAGAAGCAGGGCACGUGC................................................................................................................................................................................ 135.908292
................................................................................................................................................................................ACGUGGUCUCCUUCUCCAUC................. 14.205959
```

zma-MIR164e

Chromosome: chr1
Start: 47137780
End: 47137928
Strand: +
Strand bias: 1.0
Abundance bias: 0.983
MFE: -93.40

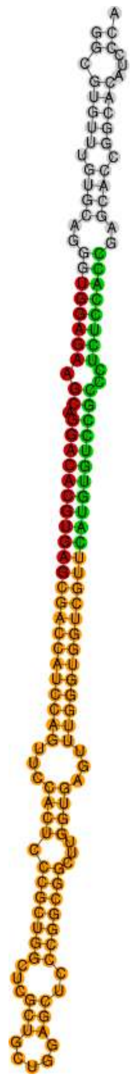

```
                 Mature                                                                                   Star
GGCGUGUUUGUGCAGGGUGGAGAAGCAGGACACGUGAGCGACCAUCCAGUUCCACUCCCGCUGGCUCGCUGCUGGAGCUCCCGGCGGCUUGGUGAGUUUGGGUGGUCGUUCAUGUGUCCGCCCUCUCCACCGAGCACCGGCACAUCCCA    21852.3
((.(((((.((((..((((((((.((.((((((((((((((((((((((...((((.(((((((...(((.....)))..)))))))...))))...))))))))))))))))))))))))..))))))))..)))).)))))...)).
.................UGGAGAAGCAGGACACGUGAG...............................................................................................................    19337.97987
..............................................................................................................CAUGUGUCCGCCCUCUCCACC..................    0.371561
.................UGGAGAAGCAGGACACGUGAGC..............................................................................................................    84.087189
.................UGGAGAAGCAGGACACGUG.................................................................................................................    945.865487
.................UGGAGAAGCAGGACACGUGAGCGA............................................................................................................    2.54354
.................UGGAGAAGCAGGACACGU..................................................................................................................    4.33077
.................UGGAGAAGCAGGACACGUGA................................................................................................................    97.333454
.................UGGAGAAGCAGGACACGUGAGCG.............................................................................................................    0.306198
.................UUGAGAAGCAGGACACGUGAG...............................................................................................................    4.263952
.................UGGAGAAGCAGGACACGCGAG...............................................................................................................    9.524502
.................UGGAGAAUCAGGACACGUGAG...............................................................................................................    2.99775
.................UGGAGAAGCAGGGCACGUGA................................................................................................................    175.819208
.................UGGAGAAGCAGGACACGUUAG...............................................................................................................    2.491903
.................UGGAGAAGCCGGACACGUGAG...............................................................................................................    4.167
.................UGGAGAAGCAGGACACGUGAU...............................................................................................................    11.738337
.................UGGAGAAGCAGGACACGUGAA...............................................................................................................    347.47558
.................UGGAGAAGCAGGACACGUGAC...............................................................................................................    1.13918
.................CGGAGAAGCAGGACACGUGAG...............................................................................................................    3.84152
.................UGUAGAAGCAGGACACGUGAG...............................................................................................................    2.47335
.................UGGAGAAGCAGGAUACGUGAG...............................................................................................................    2.612752
.................UGGAGAAGCAGGACACCUGAG...............................................................................................................    6.9794
.................UGGAGAAGCAGGACACGUGUG...............................................................................................................    0.303993
.................UGGAGAAGCAGGCCACGUGAG...............................................................................................................    27.31351
.................UGGAGAAGCAGGACAAGUGAG...............................................................................................................    14.82831
.................UGGAGAAGCGGGACACGUGAG...............................................................................................................    0.243195
.................UGGAGGAGCAGGACACGUGAG...............................................................................................................    0.212795
.................UGGAGAAGCAGGACACAUGAG...............................................................................................................    14.804693
.................UGGAGACGCAGGACACGUGAG...............................................................................................................    4.74372
.................UGGAGAAGCACGACACGUGAG...............................................................................................................    0.97644
.................UGGAGAAGCAGGAGACGUGAG...............................................................................................................    1.13918
.................UGGAGAAGCAGGACACGGGAG...............................................................................................................    13.07755
.................UGGUGAAGCAGGACACGUGAG...............................................................................................................    1.189235
.................UGGAGAAGCAGGACACGUAAG...............................................................................................................    0.64059
.................UGGAGAAGUAGGACACGUGAG...............................................................................................................    5.395591
.................UGGAGAAGCAGGACGCGUGAG...............................................................................................................    0.97644
.................UGGAGAAGCAUGACACGUGAG...............................................................................................................    3.351513
.................UAGAGAAGCAGGACACGUGAG...............................................................................................................    1.55331
.................UGGAGAAGCAGGACACUUGAG...............................................................................................................    9.019739
.................AGGAGAAGCAGGACACGUGAG...............................................................................................................    3.02782
.................UGGAAAAGCAGGACACGUGAG...............................................................................................................    0.182396
.................UGGAGAAGCAGGACACGCG.................................................................................................................    5.05256
.................UGGAGCAGCAGGACACGUGAG...............................................................................................................    7.53658
.................UGGAGAAGCAGCACACGUGAG...............................................................................................................    0.756944
.................UGAAGAAGCAGGACACGUGAG...............................................................................................................    0.212795
.................UGGAGAAGCAGGACACGAGAG...............................................................................................................    5.143271
.................UGGAGAAGCAGGACACGUGCG...............................................................................................................    25.18417
.................UGGAGAAGCAGUACACGUGAG...............................................................................................................    1.13918
.................UGGAGAAGCAGGAAACGUGAG...............................................................................................................    22.672872
.................UGGAGAAACAGGACACGUGAG...............................................................................................................    1.189235
.................UGGAGAAGCAGGACCCGUGAG...............................................................................................................    26.16285
.................UGGGGAAGCAGGACACGUGAG...............................................................................................................    3.792897
.................UGGAUAAGCAGGACACGUGAG...............................................................................................................    0.64295
.................UGCAGAAGCAGGACACGUGAG...............................................................................................................    0.212795
.................UCGAGAAGCAGGACACGUGAG...............................................................................................................    1.445378
.................UGGAGAAGCAGGACACGUGAGU..............................................................................................................    25.01459
.................UGGAGAAGCAGGACACGUGAGA..............................................................................................................    18.912619
.................UGGAGAAGCAGGACACGUA.................................................................................................................    86.08103
.................UGGCGAAGCAGGACACGUGAG...............................................................................................................    9.004545
.................UGGAGAAGAAGGACACGUGAG...............................................................................................................    9.887042
.................UGGAGAAGCAGGACAUGUGAG...............................................................................................................    8.243394
.................UGGAGAAGCAGGGCACGUGAG...............................................................................................................    1.30192
.................UGGAGAAGCAGGACACGUGGG...............................................................................................................    23.279875
..................GGAGAAGCAGGACACGUGAG...............................................................................................................    11.781824
....................AGAAGCAGGACACGUGAGCGA............................................................................................................    24.145497
```

zma-MIR164g

Chromosome: chr6
Start: 142646339
End: 142646544
Strand: -
Strand bias: 1.0
Abundance bias: 1.0
MFE: -120.20

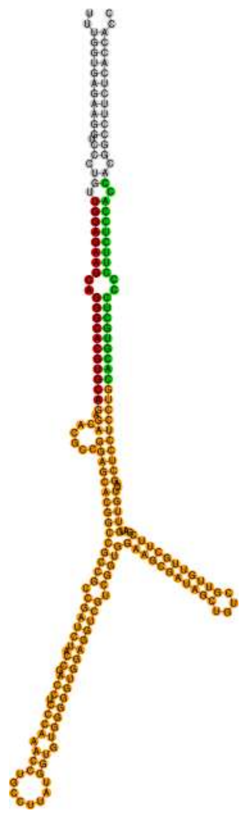

```
                   Mature                                                                                                                                                          Star
UUUGGUGAGAAGGUCCCUGUUGGAGAAGCAGGGCACGUGCAGAGACACGCCGGAGCACGGCCGCCGCCGAUCUACCGACCUCCCAAACCUGCCUUAUGGUGUGGGGGUGGAGGUCGUCGGUGGAAGCGAUAGCUGUCGUUGUUGCUUCGAUGUUGUUAGCUCCUCCUGCACGUGCUCCCCUUCUCCACCACGGCCUUCUCACCACC    71488.7
..(((((((((((.((.((.((((((((..((((((((((((.((......(((((((((((((((.((((((.((.(((.((((.(((........))).))))))))))))))).)))))(((((((((((....)))))))))))...)))))..)))))))))))))))))))..)))))))).)).))))))))))))).. 
....................UGGAGAAGCAGGGCACGUGCA.....................................................................................................................................................................    61695.51887
........................................................................................................................................................................CACGUGCUCCCCUUCUCCACC.................    358.690358
....................UGGAGAAGCAGGGCACGU.........................................................................................................................................................................    164.379322
....................UGGAGAAGCAGGGCACGUG........................................................................................................................................................................    489.534144
....................UGGAGAAGCAGGGCACGUGC.......................................................................................................................................................................    3089.04901
....................UGGAGAAGCAGGGCACCUGCA.....................................................................................................................................................................    35.30953
....................UGGAGAAGCAGCGCACGUGCA.....................................................................................................................................................................    48.68017
....................UGGGGAAGCAGGGCACGUGCA.....................................................................................................................................................................    67.470451
....................UGGAGAAGCAGGGCACGUGA.......................................................................................................................................................................    175.819208
....................UGGAGAAGCAAGGCACGUGCA.....................................................................................................................................................................    16.503446
....................UGGAGAAGCAGGGCACGUGCG.....................................................................................................................................................................    368.762493
....................UUGAGAAGCAGGGCACGUGCA.....................................................................................................................................................................    12.476702
....................UGGAGAAGCAGUGCACGUGCA.....................................................................................................................................................................    47.074189
....................UGGAGAAGCAGGGCAAGUGCA.....................................................................................................................................................................    39.214026
....................UGGAGAAGCGGGGCACGUGCA.....................................................................................................................................................................    78.541126
....................UGGAGAACCAGGGCACGUGCA.....................................................................................................................................................................    23.729777
....................UGGAGAAGCACGGCACGUGCA.....................................................................................................................................................................    27.970371
....................UGGAGAAGCAGGGCGCGUGCA.....................................................................................................................................................................    58.533162
....................UGUAGAAGCAGGGCACGUGCA.....................................................................................................................................................................    23.529335
....................UGGAGAAGCAGGGCAUGUGCA.....................................................................................................................................................................    61.033004
....................UGGAGAAGCAGGUCACGUGCA.....................................................................................................................................................................    60.137855
....................UGGAGAAGCAGGGCACGUACA.....................................................................................................................................................................    23.845858
....................UGGAGAAGCAGGGCACAUGCA.....................................................................................................................................................................    59.845226
....................UGGAGAAGCAGGGCACGUGCC.....................................................................................................................................................................    212.266876
....................UGGAGAAGCAUGGCACGUGCA.....................................................................................................................................................................    31.219393
....................UAGAGAAGCAGGGCACGUGCA.....................................................................................................................................................................    13.489773
....................UGGAGAAGCUGGGCACGUGCA.....................................................................................................................................................................    38.45266
....................UGGAGAAACAGGGCACGUGCA.....................................................................................................................................................................    11.771913
....................AGGAGAAGCAGGGCACGUGCA.....................................................................................................................................................................    11.9467
....................UGGAGAAGCAGGCCACGUGCA.....................................................................................................................................................................    29.547603
....................UGGAGAAGCAGGGCACGCGCA.....................................................................................................................................................................    94.118581
....................UGGAGAAGCAGAGCACGUGCA.....................................................................................................................................................................    23.116839
....................UGGAGGAGCAGGGCACGUGCA.....................................................................................................................................................................    55.282792
....................UGGAUAAGCAGGGCACGUGCA.....................................................................................................................................................................    44.045782
....................UGGAGAAGCAGGGCACGUGUA.....................................................................................................................................................................    56.900708
....................UGGAGAAGCAGGGCACGUUCA.....................................................................................................................................................................    33.866684
....................UGGAGAAGUAGGGCACGUGCA.....................................................................................................................................................................    67.880815
....................UGGAGCAGCAGGGCACGUGCA.....................................................................................................................................................................    40.83863
....................UGGAGAAGCAGGGAACGUGCA.....................................................................................................................................................................    76.18624
....................UGGAGAAGCAGGGCCCGUGCA.....................................................................................................................................................................    149.47749
....................UGGAGAGGCAGGGCACGUGCA.....................................................................................................................................................................    47.689179
....................CGGAGAAGCAGGGCACGUGCA.....................................................................................................................................................................    42.353063
....................UGGAGAAGCAGGGUACGUGCA.....................................................................................................................................................................    53.023948
....................UGGAGAAGCAGGACACGUGCA.....................................................................................................................................................................    29.22728
....................UGGAGAAGCAGGGCACGUCCA.....................................................................................................................................................................    61.366398
....................UGGAGAAGCAGGGCACGUGCAC....................................................................................................................................................................    29.533976
....................UGGAGAAGCAGGGCACGGGCA.....................................................................................................................................................................    37.73864
....................UGGAGAAGAAGGGCACGUGCA.....................................................................................................................................................................    13.989545
....................UGGAGAAUCAGGGCACGUGCA.....................................................................................................................................................................    61.363695
....................UGGAGAAGCCGGGCACGUGCA.....................................................................................................................................................................    62.712505
....................UGGAGAAGCAGGGCACGAGCA.....................................................................................................................................................................    13.329253
....................UGGAGACGCAGGGCACGUGCA.....................................................................................................................................................................    49.74311
....................UGGAGAAGCAGGGCACUUGCA.....................................................................................................................................................................    47.277932
....................UGGAGAAGCAGGGCACGUGCAA....................................................................................................................................................................    43.0612
....................UGGAGAAGCAGGGCACGUGAA.....................................................................................................................................................................    120.73405
....................UGGCGAAGCAGGGCACGUGCA.....................................................................................................................................................................    46.76727
.....................GGAGAAGCAGGGCACGUGCA.....................................................................................................................................................................    2374.791889
.....................GGAGAAGCAGGGCACGUGC......................................................................................................................................................................    135.908292
```

zma-MIR166a

Chromosome: chr6
Start: 87381717
End: 87381950
Strand: +
Strand bias: 1.0
Abundance bias:1.0
MFE: -83.42

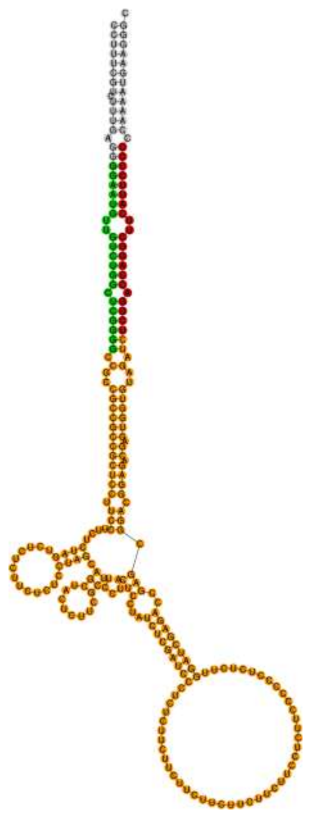

```
                Star                                                                                                                                                                                                       Mature
CCUUUCGUCUUUGAGGGGAAUGUUGUCUGGCUCGGGGCCGCCGCCGCCGCUCCUUCCUUCUCUAGUCUCUCUUCUCUCCUAGCAGCUACUCUUCGCUUCCUACUCCUAUCUCGAUCCCUCUCUUCUUCUUCUUCUUCUUCUUCUUCCUCUUCCCCCCUCUCUUGGAUCGAGACCGAGCGGACGGAGACGAGUGGUGUAGAUCUCGGACCAGGCUUCAUUCCCCCCAAAAUGAAGGGC 3259425.8
(((((((.((((.((((((((..((((((.((((((.(..((((((((((((.(((....((((.............)))).(((........))).....(((...(((((((((.........................................)))))))))..))).))).)))).)).))))))..).)))))).))))))..))))))))).)))))))))))))).
........................................................................................................................................................................................................UCGGACCAGGCUUCAUUCCCC............. 2961896.0766
................GGAAUGUUGUCUGGCUCGGGG..................................................................................................................................................................................................... 1958.905433
..........................................................................................................................................................................................................GGACCAGGCUUCAUUCCCC............. 850.718575
.........................................................................................................................................................................................................CGGACCAGGCUUCAUUCCCC............. 1981.517186
........................................................................................................................................................................................................UCGGACCAGGCUUCAUUCCC.............. 25276.65239
........................................................................................................................................................................................................UCGGACCAGGCUUCAUUCCCCC............ 1735.661278
........................................................................................................................................................................................................UUGGACCAGGCUUCAUUCCCC............. 6166.826765
........................................................................................................................................................................................................UCGGACCAGGCUUUAUUCCCC............. 3533.163648
........................................................................................................................................................................................................UCGGACCAGGCUUCAGUCCCC............. 1007.658931
........................................................................................................................................................................................................UCGGACCAGGCGUCAUUCCCC............. 834.884155
........................................................................................................................................................................................................UCGGACCAGGCUUCACUCCCC............. 1291.80307
........................................................................................................................................................................................................ACGGACCAGGCUUCAUUCCCC............. 995.77943
........................................................................................................................................................................................................UCGGACCAGGCUUCCUUCCCC............. 8766.353245
........................................................................................................................................................................................................UCAGACCAGGCUUCAUUCCCC............. 1351.88353
........................................................................................................................................................................................................UCGGACCAGGCUUCAUUUCCC............. 4299.307935
........................................................................................................................................................................................................UCGGACCAGGCUUCAUUU................ 896.57008
........................................................................................................................................................................................................UCGUACCAGGCUUCAUUCCCC............. 3689.419818
........................................................................................................................................................................................................UCGGACCAGGCCUCAUUCCCC............. 1796.88093
........................................................................................................................................................................................................CCGGACCAGGCUUCAUUCCCC............. 1566.461868
........................................................................................................................................................................................................UCGGACCAGGUUUCAUUCCCC............. 1530.66488
........................................................................................................................................................................................................UCGGACCAGGCUUCAUUCCA.............. 882.275967
........................................................................................................................................................................................................UCGGACCAGGCUUCAUUCCCA............. 19371.06785
........................................................................................................................................................................................................UCGGACCAGGCUUCAUUCCCG............. 1866.084944
........................................................................................................................................................................................................UCGGACCAGGCUUCGUUCCCC............. 2381.191261
........................................................................................................................................................................................................UCGGACCAGGCUUCAUUCACC............. 1299.806243
........................................................................................................................................................................................................UCGGACCGGGCUUCAUUCCCC............. 1473.378566
........................................................................................................................................................................................................UCGGACAAGGCUUCAUUCCCC............. 871.121313
........................................................................................................................................................................................................UCGGACCAGACUUCAUUCCCC............. 742.625098
........................................................................................................................................................................................................UCGGACCAGGCUCCAUUCCCC............. 1557.683496
........................................................................................................................................................................................................UCUGACCAGGCUUCAUUCCCC............. 3855.713456
........................................................................................................................................................................................................UCGGACCAGGCUUCAAUCCCC............. 6228.669769
........................................................................................................................................................................................................UCGGACCAGGCUUCAUGCCCC............. 742.940595
........................................................................................................................................................................................................UCGGGCCAGGCUUCAUUCCCC............. 2017.66487
........................................................................................................................................................................................................UCGGACCAGGCUUCAUUUC............... 791.817105
........................................................................................................................................................................................................UAGGACCAGGCUUCAUUCCCC............. 1004.401772
........................................................................................................................................................................................................UCGGACCAGGCUUCAUUCCAC............. 1433.934665
........................................................................................................................................................................................................UCGGACCAGGCUUCAUUACCC............. 1394.886589
........................................................................................................................................................................................................UCGGAUCAGGCUUCAUUCCCC............. 1152.339525
........................................................................................................................................................................................................GCGGACCAGGCUUCAUUCCCC............. 1373.489964
........................................................................................................................................................................................................UCGGACCAGGCUUCUUUCCCC............. 1025.081502
........................................................................................................................................................................................................UCGGACCAGGCUUAAUUCCCC............. 2595.787516
........................................................................................................................................................................................................UCGGACCAGUCUUCAUUCCCC............. 1698.045668
........................................................................................................................................................................................................UCGGACCAGGAUUCAUUCCCC............. 3361.421404
........................................................................................................................................................................................................UCGAACCAGGCUUCAUUCCCC............. 1222.225559
........................................................................................................................................................................................................UCGGACCAUGCUUCAUUCCCC............. 2915.09744
........................................................................................................................................................................................................UCGGACCAGGCUUCAUCCCCC............. 3088.3769
........................................................................................................................................................................................................UCGGACCAGGGUUCAUUCCCC............. 1443.742425
........................................................................................................................................................................................................UCGGACUAGGCUUCAUUCCCC............. 1395.703481
........................................................................................................................................................................................................UCGGACCAAGCUUCAUUCCCC............. 754.854373
........................................................................................................................................................................................................UCGGACCAGGCUUCAUUCCCU............. 54756.65173
........................................................................................................................................................................................................UCGGACCAGGCUUCAUUCA............... 774.926224
........................................................................................................................................................................................................UCGGACCAGGCUUCAUUCUCC............. 3115.46708
........................................................................................................................................................................................................UCGGACCAGGCUUCAUUCU............... 5070.280857
.......................................................................................................................................................................................................CUCGGACCAGGCUUCAUUCCCC............. 2369.518445
.......................................................................................................................................................................................................CUCGGACCAGGCUUCAUUCCC.............. 8074.545384
......................................................................................................................................................................................................UCUCGGACCAGGCUUCAUUCC............... 66118.43634
.....................................................................................................................................................................................................AUCUCGGACCAGGCUUCAUUCCCC............. 2698.974923
.................GAAUGUUGUCUGGCUCGGGGC.................................................................................................................................................................................................... 3118.435612
```

zma-MIR166b

Chromosome: chr9
Start: 90025262
End: 90025441
Strand: +
Strand bias: 1.0
Abundance bias:1.0
MFE: -67.80

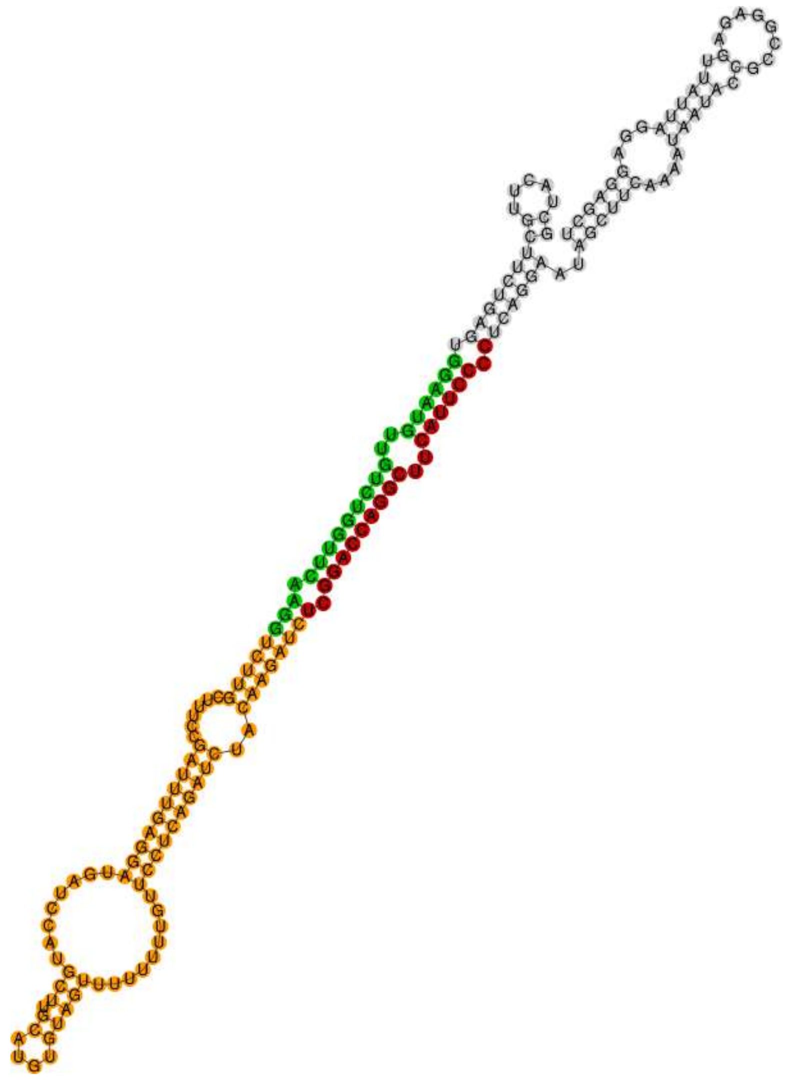

```
                 Star                                                                                       Mature
GCUACUUGCUUCUGAGUGGAAUGUUGUCUGGUUCAAGGUCUUGCUUUCCGAUUUGAGGAUGAUCCAUGCUUGCAUGUGUAGUUUUUUUUGUUCCUCAGAUCUACAAGAUCUCGGACCAGGCUUCAUUCCCCUCAGGAAUAGCUUCAAAAUAAUACCGCCGGAGAGUUAUUAGGAGGAGCU    3283807.2
((.....))(((((((.((((((..(((((((((.((((((((......((((((((((........(((.((....))))).........))))))))))..)))))))).)))))))))..)))))).)))))))..((((((....(((((.(........).)))))...))))))
..............................................................................................................UCGGACCAGGCUUCAUUCCCC.................................................    2961896.0766
.................GGAAUGUUGUCUGGUUCAAGG..............................................................................................................................................    10309.99817
................................................................................................................GGACCAGGCUUCAUUCCCC.................................................    850.718575
...............................................................................................................CGGACCAGGCUUCAUUCCCCU................................................    267.040425
...............................................................................................................CGGACCAGGCUUCAUUCCCC.................................................    1981.517186
..............................................................................................................UCGGACCAGGCUUCAUUCCC..................................................    25276.65239
..............................................................................................................UCGGACCAGGCUUCAUUCCCCU................................................    18743.33647
..............................................................................................................UUGGACCAGGCUUCAUUCCCC.................................................    6166.826765
..............................................................................................................UCGGACGAGGCUUCAUUCCCC.................................................    170.447553
..............................................................................................................UCGGACCAGGCUUUAUUCCCC.................................................    3533.163648
..............................................................................................................UCGGACCAGGCUUCAGUCCCC.................................................    1007.658931
..............................................................................................................UCGGACCAGGCGUCAUUCCCC.................................................    834.884155
..............................................................................................................UCGGACCAGGCUUCACUCCCC.................................................    1291.80307
..............................................................................................................ACGGACCAGGCUUCAUUCCCC.................................................    995.77943
..............................................................................................................UCGGACCAGGCUUCAUUCCGC.................................................    519.213536
..............................................................................................................UCGGACCAGGCUUCCUUCCCC.................................................    8766.353245
..............................................................................................................UCAGACCAGGCUUCAUUCCCC.................................................    1351.88353
..............................................................................................................UCGGACCAGGCUUCAUUUCCC.................................................    4299.307935
..............................................................................................................UCGGACCAGGCUUCAUUU....................................................    896.57008
..............................................................................................................UCGUACCAGGCUUCAUUCCCC.................................................    3689.419818
..............................................................................................................UCGGACCAGGCCUCAUUCCCC.................................................    1796.88093
..............................................................................................................UCGGACCAGGCUUCAUUCCCUU................................................    209.06847
..............................................................................................................UCGGACCAGGCUUCCUUCCC..................................................    96.91076
..............................................................................................................CCGGACCAGGCUUCAUUCCCC.................................................    1566.461868
..............................................................................................................UCGCACCAGGCUUCAUUCCCC.................................................    563.824914
..............................................................................................................UCGGACCAGGUUUCAUUCCCC.................................................    1530.66488
..............................................................................................................UCGGACCAGGCUUCAUUCCA..................................................    882.275967
..............................................................................................................UCGGACCAGGCUUCAUUCCCA.................................................    19371.06785
..............................................................................................................UCGGACCAGGCUUCAUUCCCG.................................................    1866.084944
..............................................................................................................UCGGACCAGGCUUCAUCC....................................................    213.397153
..............................................................................................................UCGGACCAGGCUUCGUUCCCC.................................................    2381.191261
..............................................................................................................UCGGACCAGGCUUCAUUCACC.................................................    1299.806243
..............................................................................................................UGGGACCAGGCUUCAUUCCCC.................................................    548.708034
..............................................................................................................UCGGACCGGGCUUCAUUCCCC.................................................    1473.378566
..............................................................................................................UCGGACAAGGCUUCAUUCCCC.................................................    871.121313
..............................................................................................................UCGGACCUGGCUUCAUUCCCC.................................................    644.270222
..............................................................................................................UCGGACCCGGCUUCAUUCCCC.................................................    464.050619
..............................................................................................................UCGGACCAGACUUCAUUCCCC.................................................    742.625098
..............................................................................................................UCGGACCAGGCUCCAUUCCCC.................................................    1557.683496
..............................................................................................................UCUGACCAGGCUUCAUUCCCC.................................................    3855.713456
..............................................................................................................UCGGACCAGGCUUCAAUCCCC.................................................    6228.669769
..............................................................................................................UCGGACCAGGCUUCAUGCCCC.................................................    742.940595
..............................................................................................................UCGGACCAGGCUUGAUUCCCC.................................................    318.168133
..............................................................................................................UCGGACCAGGCUUCAUUCUC..................................................    223.097641
..............................................................................................................UCGGACCACGCUUCAUUCCCC.................................................    177.12601
..............................................................................................................UCGGGCCAGGCUUCAUUCCCC.................................................    2017.66487
..............................................................................................................UCGGACCAGGCUUCAUCCCC..................................................    201.411416
..............................................................................................................UCGGACCAGGCUUCAUUUC...................................................    791.817105
..............................................................................................................UCGGACCAGGCUUCAUUGCCC.................................................    325.308415
..............................................................................................................UCGGAACAGGCUUCAUUCCCC.................................................    305.306124
..............................................................................................................UAGGACCAGGCUUCAUUCCCC.................................................    1004.401772
..............................................................................................................UCGGACCAGGCUUCAUUCCAC.................................................    1433.934665
..............................................................................................................UCGGACCAGGCUUCAUUCGCC.................................................    168.967216
..............................................................................................................UCGGACCAGGCUUCAUUACCC.................................................    1394.886589
..............................................................................................................UCGGAUCAGGCUUCAUUCCCC.................................................    1152.339525
..............................................................................................................GCGGACCAGGCUUCAUUCCCC.................................................    1373.489964
..............................................................................................................UCGGACCAGGCUUCUUUCCCC.................................................    1025.081502
..............................................................................................................UCGGACCAGCCUUCAUUCCCC.................................................    249.973803
..............................................................................................................UCGGACCAGGCUUAAUUCCCC.................................................    2595.787516
..............................................................................................................UCGGACCAGGCUUCAUACCCC.................................................    615.957197
..............................................................................................................UCGGACCAGGCAUCAUUCCCC.................................................    304.313191
..............................................................................................................UCGGACCAGUCUUCAUUCCCC.................................................    1698.045668
..............................................................................................................UCGGACCAGGAUUCAUUCCCC.................................................    3361.421404
..............................................................................................................UCGAACCAGGCUUCAUUCCCC.................................................    1222.225559
..............................................................................................................UCGGACCAUGCUUCAUUCCCC.................................................    2915.09744
..............................................................................................................UCGGACCAGGCUGCAUUCCCC.................................................    494.068714
..............................................................................................................UCGGACCAGGCUUCAUCCCCC.................................................    3088.3769
..............................................................................................................UCGGACCAGGGUUCAUUCCCC.................................................    1443.742425
..............................................................................................................UCGGACCAGGCUUCAAUCCCCU................................................    220.165543
..............................................................................................................UCGGACUAGGCUUCAUUCCCC.................................................    1395.703481
..............................................................................................................UCGGACCAAGCUUCAUUCCCC.................................................    754.854373
..............................................................................................................UCGGACCAGGCUUCAUUCCCU.................................................    54756.65173
..............................................................................................................UCGGACCAGGCUUCAUUCCUCU................................................    186.355772
..............................................................................................................UCGGACCAGGCUACAUUCCCC.................................................    282.049971
..............................................................................................................UCGGACCAGGCUUCAUUCA...................................................    774.926224
..............................................................................................................UCCGACCAGGCUUCAUUCCCC.................................................    642.164282
..............................................................................................................UCGGACCAGGCUUCAUUCUCC.................................................    3115.46708
..............................................................................................................UCGGCCCAGGCUUCAUUCCCC.................................................    380.166369
..............................................................................................................UCGGACCAGGCUUCAUUCU...................................................    5070.280857
..............................................................................................................UCGGUCCAGGCUUCAUUCCCC.................................................    488.345507
..............................................................................................................UCGGACCAGGCUUCAUCCC...................................................    590.988535
.............................................................................................................CUCGGACCAGGCUUCAUUCCCC.................................................    2369.518445
.............................................................................................................CUCGGACCAGGCUUCAUUCCC..................................................    8074.545384
............................................................................................................UCUCGGACCAGGCUUCAUUCC...................................................    66118.43634
............................................................................................................UCUCGGACCAGGCUUCAUUCCC..................................................    117.475902
............................................................................................................UCUCGGACCAGGCUUCAUUCCCC.................................................    133.426924
............................................................................................................UCUCGGACCAGGCUUCAUUC....................................................    86.021335
............................................................................................................UCUCGGACCAGUCUUCAUUCC...................................................    88.42751
............................................................................................................UCUCGGACCAGGCUUCAUUCU...................................................    500.66045
............................................................................................................UCUCGGACCAGGCUUCAUUCA...................................................    169.326233
............................................................................................................UCUCGGACCAUGCUUCAUUCC...................................................    87.88165
...........................................................................................................AUCUCGGACCAGGCUUCAUUCCCC.................................................    2698.974923
...........................................................................................................AUCUCGGACCAGGCUUCAUUC....................................................    170.31007
.................GGAAUGUUGUCUGGUUCAAGGU.............................................................................................................................................    197.844328
.................GGAAUGUUGUCUGGUUCAA................................................................................................................................................    74.347792
.................GGAAUGUUGUCUGGUUCAAG...............................................................................................................................................    266.42226
.................GGAAUGUUGUCUGGUUCAAGGA.............................................................................................................................................    124.863184
.................GGAAUGUUGUCUGGUUCAAGA..............................................................................................................................................    82.643721
```

zma-MIR166b1

Chromosome: chr1
Start: 226759239
End: 226759392
Strand: -
Strand bias: 1.0
Abundance bias: 1.0
MFE: -67.20

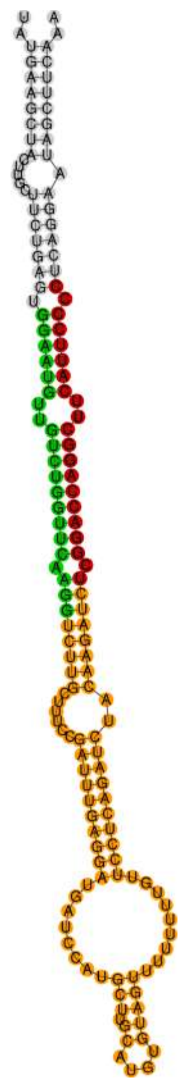

```
                                  Star                                                                                        Mature
UAUGAAGCUACUUGCUUCUGAGUGGAAUGUUGUCUGGUUCAAGGUCUUGCUUUCCGAUUUGAGGAUGAUCCAUGCUUGCAUGUGUAGUUUUUUUUGUUCCUCAGAUCUACAAGAUCUCGGACCAGGCUUCAUUCCCCUCAGGAAUAGCUUCAAA    3283807.2
..((((((((.....(((((((.((((((..(((((((((.((((((((......((((((((((........(((.((....))))).........))))))))))..)))))))).)))))))))..)))))).))))))).))))))))..
....................................................................................................................UCGGACCAGGCUUCAUUCCCC.................    2961896.0766
.......................GGAAUGUUGUCUGGUUCAAGG..............................................................................................................    10309.99817
......................................................................................................................GGACCAGGCUUCAUUCCCC.................    850.718575
.....................................................................................................................CGGACCAGGCUUCAUUCCCCU................    267.040425
.....................................................................................................................CGGACCAGGCUUCAUUCCCC.................    1981.517186
....................................................................................................................UCGGACCAGGCUUCAUUCCC..................    25276.65239
....................................................................................................................UCGGACCAGGCUUCAUUCCCCU................    18743.33647
....................................................................................................................UUGGACCAGGCUUCAUUCCCC.................    6166.826765
....................................................................................................................UCGGACGAGGCUUCAUUCCCC.................    170.447553
....................................................................................................................UCGGACCAGGCUUUAUUCCCC.................    3533.163648
....................................................................................................................UCGGACCAGGCUUCAGUCCCC.................    1007.658931
....................................................................................................................UCGGACCAGGCGUCAUUCCCC.................    834.884155
....................................................................................................................UCGGACCAGGCUUCACUCCCC.................    1291.80307
....................................................................................................................ACGGACCAGGCUUCAUUCCCC.................    995.77943
....................................................................................................................UCGGACCAGGCUUCAUUCCGC.................    519.213536
....................................................................................................................UCGGACCAGGCUUCCUUCCCC.................    8766.353245
....................................................................................................................UCAGACCAGGCUUCAUUCCCC.................    1351.88353
....................................................................................................................UCGGACCAGGCUUCAUUUCCC.................    4299.307935
....................................................................................................................UCGGACCAGGCUUCAUUU....................    896.57008
....................................................................................................................UCGUACCAGGCUUCAUUCCCC.................    3689.419818
....................................................................................................................UCGGACCAGGCCUCAUUCCCC.................    1796.88093
....................................................................................................................UCGGACCAGGCUUCAUUCCCCUU...............    209.06847
....................................................................................................................UCGGACCAGGCUUCCUUCCC..................    96.91076
....................................................................................................................CCGGACCAGGCUUCAUUCCCC.................    1566.461868
....................................................................................................................UCGCACCAGGCUUCAUUCCCC.................    563.824914
....................................................................................................................UCGGACCAGGUUUCAUUCCCC.................    1530.66488
....................................................................................................................UCGGACCAGGCUUCAUUCCA..................    882.275967
....................................................................................................................UCGGACCAGGCUUCAUUCCCA.................    19371.06785
....................................................................................................................UCGGACCAGGCUUCAUUCCCG.................    1866.084944
....................................................................................................................UCGGACCAGGCUUCAUCC....................    213.397153
....................................................................................................................UCGGACCAGGCUUCGUUCCCC.................    2381.191261
....................................................................................................................UCGGACCAGGCUUCAUUCACC.................    1299.806243
....................................................................................................................UGGGACCAGGCUUCAUUCCCC.................    548.708034
....................................................................................................................UCGGACCGGGCUUCAUUCCCC.................    1473.378566
....................................................................................................................UCGGACAAGGCUUCAUUCCCC.................    871.121313
....................................................................................................................UCGGACCUGGCUUCAUUCCCC.................    644.270222
....................................................................................................................UCGGACCCGGCUUCAUUCCCC.................    464.050619
....................................................................................................................UCGGACCAGACUUCAUUCCCC.................    742.625098
....................................................................................................................UCGGACCAGGCUCCAUUCCCC.................    1557.683496
....................................................................................................................UCUGACCAGGCUUCAUUCCCC.................    3855.713456
....................................................................................................................UCGGACCAGGCUUCAAUCCCC.................    6228.669769
....................................................................................................................UCGGACCAGGCUUCAUGCCCC.................    742.940595
....................................................................................................................UCGGACCAGGCUUGAUUCCCC.................    318.168133
....................................................................................................................UCGGACCAGGCUUCAUUCUC..................    223.097641
....................................................................................................................UCGGACCACGCUUCAUUCCCC.................    177.12601
....................................................................................................................UCGGGCCAGGCUUCAUUCCCC.................    2017.66487
....................................................................................................................UCGGACCAGGCUUCAUCCCC..................    201.411416
....................................................................................................................UCGGACCAGGCUUCAUUUC...................    791.817105
....................................................................................................................UCGGACCAGGCUUCAUUGCCC.................    325.308415
....................................................................................................................UCGGAACAGGCUUCAUUCCCC.................    305.306124
....................................................................................................................UAGGACCAGGCUUCAUUCCCC.................    1004.401772
....................................................................................................................UCGGACCAGGCUUCAUUCCAC.................    1433.934665
....................................................................................................................UCGGACCAGGCUUCAUUCGCC.................    168.967216
....................................................................................................................UCGGACCAGGCUUCAUUACCC.................    1394.886589
....................................................................................................................UCGGAUCAGGCUUCAUUCCCC.................    1152.339525
....................................................................................................................GCGGACCAGGCUUCAUUCCCC.................    1373.489964
....................................................................................................................UCGGACCAGGCUUCUUUCCCC.................    1025.081502
....................................................................................................................UCGGACCAGCCUUCAUUCCCC.................    249.973803
....................................................................................................................UCGGACCAGGCUUAAUUCCCC.................    2595.787516
....................................................................................................................UCGGACCAGGCUUCAUACCCC.................    615.957197
....................................................................................................................UCGGACCAGGCAUCAUUCCCC.................    304.313191
....................................................................................................................UCGGACCAGUCUUCAUUCCCC.................    1698.045668
....................................................................................................................UCGGACCAGGAUUCAUUCCCC.................    3361.421404
....................................................................................................................UCGAACCAGGCUUCAUUCCCC.................    1222.225559
....................................................................................................................UCGGACCAUGCUUCAUUCCCC.................    2915.09744
....................................................................................................................UCGGACCAGGCUGCAUUCCCC.................    494.068714
....................................................................................................................UCGGACCAGGCUUCAUCCCCC.................    3088.3769
....................................................................................................................UCGGACCAGGGUUCAUUCCCC.................    1443.742425
....................................................................................................................UCGGACCAGGCUUCAAUCCCCU................    220.165543
....................................................................................................................UCGGACUAGGCUUCAUUCCCC.................    1395.703481
....................................................................................................................UCGGACCAAGCUUCAUUCCCC.................    754.854373
....................................................................................................................UCGGACCAGGCUUCAUUCCCU.................    54756.65173
....................................................................................................................UCGGACCAGGCUUCAUUCCUCU................    186.355772
....................................................................................................................UCGGACCAGGCUACAUUCCCC.................    282.049971
....................................................................................................................UCGGACCAGGCUUCAUUCA...................    774.926224
....................................................................................................................UCCGACCAGGCUUCAUUCCCC.................    642.164282
....................................................................................................................UCGGACCAGGCUUCAUUCUCC.................    3115.46708
....................................................................................................................UCGGCCCAGGCUUCAUUCCCC.................    380.166369
....................................................................................................................UCGGACCAGGCUUCAUUCU...................    5070.280857
....................................................................................................................UCGGUCCAGGCUUCAUUCCCC.................    488.345507
....................................................................................................................UCGGACCAGGCUUCAUCCC...................    590.988535
...................................................................................................................CUCGGACCAGGCUUCAUUCCCC.................    2369.518445
...................................................................................................................CUCGGACCAGGCUUCAUUCCC..................    8074.545384
..................................................................................................................UCUCGGACCAGGCUUCAUUCC...................    66118.43634
..................................................................................................................UCUCGGACCAGGCUUCAUUCCC..................    117.475902
..................................................................................................................UCUCGGACCAGGCUUCAUUCCCC.................    133.426924
..................................................................................................................UCUCGGACCAGGCUUCAUUC....................    86.021335
..................................................................................................................UCUCGGACCAGUCUUCAUUCC...................    88.42751
..................................................................................................................UCUCGGACCAGGCUUCAUUCU...................    500.66045
..................................................................................................................UCUCGGACCAGGCUUCAUUCA...................    169.326233
..................................................................................................................UCUCGGACCAUGCUUCAUUCC...................    87.88165
.................................................................................................................AUCUCGGACCAGGCUUCAUUCCCC.................    2698.974923
.................................................................................................................AUCUCGGACCAGGCUUCAUUC....................    170.31007
.......................GGAAUGUUGUCUGGUUCAAGGU.............................................................................................................    197.844328
.......................GGAAUGUUGUCUGGUUCAA.................................................................................................................    74.347792
.......................GGAAUGUUGUCUGGUUCAAG................................................................................................................    266.42226
.......................GGAAUGUUGUCUGGUUCAAGGA.............................................................................................................    124.863184
.......................GGAAUGUUGUCUGGUUCAAGA...............................................................................................................    82.643721
```

zma-MIR166c

```
Chromosome:    chr4
Start:         153858438
End:           153858556
Strand:        +
Strand bias:   1.0
Abundance bias:1.0
MFE:           -58.40
```

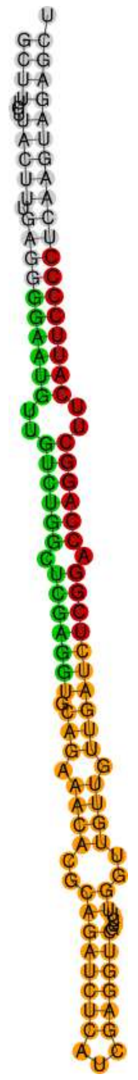

```
                 Star                                                                 Mature
GCUUUCUUACUUUGAGGGGAAUGUUGUCUGGCUCGAGGUGCAGAAACACGCAGAUCUCAUCGAGGUCGUCUUGGUUGUUGUUGAUCUCGGACCAGGCUUCAUUCCCCUCAAGUAGAGCU    3285755.9
((((...(((((.((((((((((..((((((.(((((((.(((.((((..((((((((...))))))....))..)))).)))))))))).))))))..))))))))))))))))))).
......................................................................................UCGGACCAGGCUUCAUUCCCC............    2961896.0766
.................GGAAUGUUGUCUGGCUCGAGG.................................................................................    8419.41475
........................................................................................GGACCAGGCUUCAUUCCCC............    850.718575
.......................................................................................CGGACCAGGCUUCAUUCCCCU...........    267.040425
.......................................................................................CGGACCAGGCUUCAUUCCCC............    1981.517186
......................................................................................UCGGACCAGGCUUCAUUCCC.............    25276.65239
......................................................................................UCGGACCAGGCUUCAUUCCCCU...........    18743.33647
......................................................................................UUGGACCAGGCUUCAUUCCCC............    6166.826765
......................................................................................UCGGACGAGGCUUCAUUCCCC............    170.447553
......................................................................................UCGGACCAGGCUUUAUUCCCC............    3533.163648
......................................................................................UCGGACCAGGCUUCAGUCCCC............    1007.658931
......................................................................................UCGGACCAGGCGUCAUUCCCC............    834.884155
......................................................................................UCGGACCAGGCUUCACUCCCC............    1291.80307
......................................................................................ACGGACCAGGCUUCAUUCCCC............    995.77943
......................................................................................UCGGACCAGGCUUCAUUCCGC............    519.213536
......................................................................................UCGGACCAGGCUUCCUUCCCC............    8766.353245
......................................................................................UCAGACCAGGCUUCAUUCCCC............    1351.88353
......................................................................................UCGGACCAGGCUUCAUUUCCC............    4299.307935
......................................................................................UCGGACCAGGCUUCAUUU...............    896.57008
......................................................................................UCGUACCAGGCUUCAUUCCCC............    3689.419818
......................................................................................UCGGACCAGGCCUCAUUCCCC............    1796.88093
......................................................................................UCGGACCAGGCUUCAUUCCCUU...........    209.06847
......................................................................................UCGGACCAGGCUUCCUUCCC.............    96.91076
......................................................................................CCGGACCAGGCUUCAUUCCCC............    1566.461868
......................................................................................UCGCACCAGGCUUCAUUCCCC............    563.824914
......................................................................................UCGGACCAGGUUUCAUUCCCC............    1530.66488
......................................................................................UCGGACCAGGCUUCAUUCCA.............    882.275967
......................................................................................UCGGACCAGGCUUCAUUCCCA............    19371.06785
......................................................................................UCGGACCAGGCUUCAUUCCCG............    1866.084944
......................................................................................UCGGACCAGGCUUCAUCC...............    213.397153
......................................................................................UCGGACCAGGCUUCGUUCCCC............    2381.191261
......................................................................................UCGGACCAGGCUUCAUUCACC............    1299.806243
......................................................................................UGGGACCAGGCUUCAUUCCCC............    548.708034
......................................................................................UCGGACCGGGCUUCAUUCCCC............    1473.378566
......................................................................................UCGGACAAGGCUUCAUUCCCC............    871.121313
......................................................................................UCGGACCUGGCUUCAUUCCCC............    644.270222
......................................................................................UCGGACCCGGCUUCAUUCCCC............    464.050619
......................................................................................UCGGACCAGACUUCAUUCCCC............    742.625098
......................................................................................UCGGACCAGGCUCCAUUCCCC............    1557.683496
......................................................................................UCUGACCAGGCUUCAUUCCCC............    3855.713456
......................................................................................UCGGACCAGGCUUCAAUCCCC............    6228.669769
......................................................................................UCGGACCAGGCUUCAUGCCCC............    742.940595
......................................................................................UCGGACCAGGCUUGAUUCCCC............    318.168133
......................................................................................UCGGACCAGGCUUCAUUCUC.............    223.097641
......................................................................................UCGGACCACGCUUCAUUCCCC............    177.12601
......................................................................................UCGGGCCAGGCUUCAUUCCCC............    2017.66487
......................................................................................UCGGACCAGGCUUCAUCCCC.............    201.411416
......................................................................................UCGGACCAGGCUUCAUUUC..............    791.817105
......................................................................................UCGGACCAGGCUUCAUUGCCC............    325.308415
......................................................................................UCGGAACAGGCUUCAUUCCCC............    305.306124
......................................................................................UAGGACCAGGCUUCAUUCCCC............    1004.401772
......................................................................................UCGGACCAGGCUUCAUUCCAC............    1433.934665
......................................................................................UCGGACCAGGCUUCAUUCGCC............    168.967216
......................................................................................UCGGACCAGGCUUCAUUACCC............    1394.886589
......................................................................................UCGGAUCAGGCUUCAUUCCCC............    1152.339525
......................................................................................GCGGACCAGGCUUCAUUCCCC............    1373.489964
......................................................................................UCGGACCAGGCUUCUUUCCCC............    1025.081502
......................................................................................UCGGACCAGCCUUCAUUCCCC............    249.973803
......................................................................................UCGGACCAGGCUUAAUUCCCC............    2595.787516
......................................................................................UCGGACCAGGCUUCAUACCCC............    615.957197
......................................................................................UCGGACCAGGCAUCAUUCCCC............    304.313191
......................................................................................UCGGACCAGUCUUCAUUCCCC............    1698.045668
......................................................................................UCGGACCAGGAUUCAUUCCCC............    3361.421404
......................................................................................UCGAACCAGGCUUCAUUCCCC............    1222.225559
......................................................................................UCGGACCAUGCUUCAUUCCCC............    2915.09744
......................................................................................UCGGACCAGGCUGCAUUCCCC............    494.068714
......................................................................................UCGGACCAGGCUUCAUCCCCC............    3088.3769
......................................................................................UCGGACCAGGGUUCAUUCCCC............    1443.742425
......................................................................................UCGGACCAGGCUUCAAUCCCCU...........    220.165543
......................................................................................UCGGACUAGGCUUCAUUCCCC............    1395.703481
......................................................................................UCGGACCAAGCUUCAUUCCCC............    754.854373
......................................................................................UCGGACCAGGCUUCAUUCCCU............    54756.65173
......................................................................................UCGGACCAGGCUUCAUUCCUCU...........    186.355772
......................................................................................UCGGACCAGGCUACAUUCCCC............    282.049971
......................................................................................UCGGACCAGGCUUCAUUCA..............    774.926224
......................................................................................UCCGACCAGGCUUCAUUCCCC............    642.164282
......................................................................................UCGGACCAGGCUUCAUUCUCC............    3115.46708
......................................................................................UCGGCCCAGGCUUCAUUCCCC............    380.166369
......................................................................................UCGGACCAGGCUUCAUUCU..............    5070.280857
......................................................................................UCGGUCCAGGCUUCAUUCCCC............    488.345507
......................................................................................UCGGACCAGGCUUCAUCCC..............    590.988535
.....................................................................................CUCGGACCAGGCUUCAUUCCCC............    2369.518445
.....................................................................................CUCGGACCAGGCUUCAUUCCC.............    8074.545384
....................................................................................UCUCGGACCAGGCUUCAUUCC..............    66118.43634
....................................................................................UCUCGGACCAGGCUUCAUUCCC.............    117.475902
....................................................................................UCUCGGACCAGGCUUCAUUCCCC............    133.426924
....................................................................................UCUCGGACCAGUCUUCAUUCC..............    88.42751
....................................................................................UCUCGGACCAGGCUUCAUUCU..............    500.66045
....................................................................................UCUCGGACCAGGCUUCAUUCA..............    169.326233
...................................................................................AUCUCGGACCAGGCUUCAUUCCCC............    2698.974923
...................................................................................AUCUCGGACCAGGCUUCAUUC...............    170.31007
...................AAUGUUGUCUGGCUCGAGGUG...............................................................................    1637.267923
..................GAAUGUUGUCUGGCUCGAGGU................................................................................    1114.559003
.................GGAAUGUUGUCUGGCUCGA...................................................................................    128.259348
.................GGAAUGUUGUCUGGCUCGAG..................................................................................    168.802377
.................GGAAUGUUGGCUGGCUCGAGG.................................................................................    1308.734216
.................GGAAUGUUGUCUGGCUCGAGA.................................................................................    151.39844
................GGGAAUGUUGUCUGGCUCGAG..................................................................................    206.880563
```

zma-MIR166d

Chromosome: chr5
Start: 22443517
End: 22443658
Strand: -
Strand bias: 1.0
Abundance bias: 1.0
MFE: -61.90

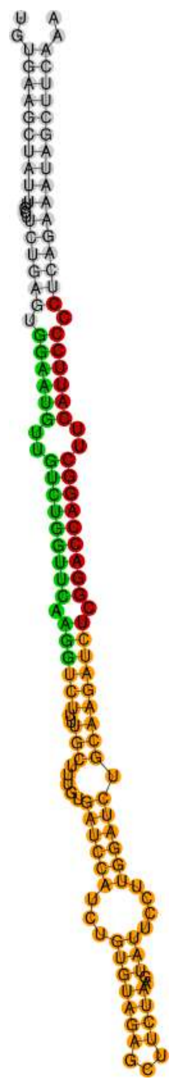

```
                       Star                                                                                 Mature
UGUGAAGCUAUUUGCUUCUGAGUGGAAUGUUGUCUGGUUCAAGGUCUUUUGCUUUGUGAUCCAUCUGUGUAGAGCUUCUAAGUAUUCCUUGGAUCUGCAAGAUCUCGGACCAGGCUUCAUUCCCCUCAGAAAUAGCUUCAAA    3283804.2
..((((((((((....((((((.((((((..(((((((((.((((((..(((.....((((((...(((((((...))))..)))....)))))).))))))))).)))))))))..)))))).))))))))))))))))..
........................................................................................................UCGGACCAGGCUUCAUUCCCC.................    2961896.0766
.......................GGAAUGUUGUCUGGUUCAAGG..................................................................................................    10309.99817
..........................................................................................................GGACCAGGCUUCAUUCCCC.................    850.718575
.........................................................................................................CGGACCAGGCUUCAUUCCCCU................    267.040425
.........................................................................................................CGGACCAGGCUUCAUUCCCC.................    1981.517186
........................................................................................................UCGGACCAGGCUUCAUUCCC..................    25276.65239
........................................................................................................UCGGACCAGGCUUCAUUCCCCU................    18743.33647
........................................................................................................UUGGACCAGGCUUCAUUCCCC.................    6166.826765
........................................................................................................UCGGACGAGGCUUCAUUCCCC.................    170.447553
........................................................................................................UCGGACCAGGCUUUAUUCCCC.................    3533.163648
........................................................................................................UCGGACCAGGCUUCAGUCCCC.................    1007.658931
........................................................................................................UCGGACCAGGCGUCAUUCCCC.................    834.884155
........................................................................................................UCGGACCAGGCUUCACUCCCC.................    1291.80307
........................................................................................................ACGGACCAGGCUUCAUUCCCC.................    995.77943
........................................................................................................UCGGACCAGGCUUCAUUCCGC.................    519.213536
........................................................................................................UCGGACCAGGCUUCCUUCCCC.................    8766.353245
........................................................................................................UCAGACCAGGCUUCAUUCCCC.................    1351.88353
........................................................................................................UCGGACCAGGCUUCAUUUCCC.................    4299.307935
........................................................................................................UCGGACCAGGCUUCAUUU....................    896.57008
........................................................................................................UCGUACCAGGCUUCAUUCCCC.................    3689.419818
........................................................................................................UCGGACCAGGCCUCAUUCCCC.................    1796.88093
........................................................................................................UCGGACCAGGCUUCAUUCCCUU................    209.06847
........................................................................................................UCGGACCAGGCUUCCUUCCC..................    96.91076
........................................................................................................CCGGACCAGGCUUCAUUCCCC.................    1566.461868
........................................................................................................UCGCACCAGGCUUCAUUCCCC.................    563.824914
........................................................................................................UCGGACCAGGUUUCAUUCCCC.................    1530.66488
........................................................................................................UCGGACCAGGCUUCAUUCCA..................    882.275967
........................................................................................................UCGGACCAGGCUUCAUUCCCA.................    19371.06785
........................................................................................................UCGGACCAGGCUUCAUUCCCG.................    1866.084944
........................................................................................................UCGGACCAGGCUUCAUCC....................    213.397153
........................................................................................................UCGGACCAGGCUUCGUUCCCC.................    2381.191261
........................................................................................................UCGGACCAGGCUUCAUUCACC.................    1299.806243
........................................................................................................UGGGACCAGGCUUCAUUCCCC.................    548.708034
........................................................................................................UCGGACCGGGCUUCAUUCCCC.................    1473.378566
........................................................................................................UCGGACAAGGCUUCAUUCCCC.................    871.121313
........................................................................................................UCGGACCUGGCUUCAUUCCCC.................    644.270222
........................................................................................................UCGGACCCGGCUUCAUUCCCC.................    464.050619
........................................................................................................UCGGACCAGACUUCAUUCCCC.................    742.625098
........................................................................................................UCGGACCAGGCUCCAUUCCCC.................    1557.683496
........................................................................................................UCUGACCAGGCUUCAUUCCCC.................    3855.713456
........................................................................................................UCGGACCAGGCUUCAAUCCCC.................    6228.669769
........................................................................................................UCGGACCAGGCUUCAUGCCCC.................    742.940595
........................................................................................................UCGGACCAGGCUUGAUUCCCC.................    318.168133
........................................................................................................UCGGACCAGGCUUCAUUCUC..................    223.097641
........................................................................................................UCGGACCACGCUUCAUUCCCC.................    177.12601
........................................................................................................UCGGGCCAGGCUUCAUUCCCC.................    2017.66487
........................................................................................................UCGGACCAGGCUUCAUCCCC..................    201.411416
........................................................................................................UCGGACCAGGCUUCAUUUC...................    791.817105
........................................................................................................UCGGACCAGGCUUCAUUGCCC.................    325.308415
........................................................................................................UCGGAACAGGCUUCAUUCCCC.................    305.306124
........................................................................................................UAGGACCAGGCUUCAUUCCCC.................    1004.401772
........................................................................................................UCGGACCAGGCUUCAUUCCAC.................    1433.934665
........................................................................................................UCGGACCAGGCUUCAUUCGCC.................    168.967216
........................................................................................................UCGGACCAGGCUUCAUUACCC.................    1394.886589
........................................................................................................UCGGAUCAGGCUUCAUUCCCC.................    1152.339525
........................................................................................................GCGGACCAGGCUUCAUUCCCC.................    1373.489964
........................................................................................................UCGGACCAGGCUUCUUUCCCC.................    1025.081502
........................................................................................................UCGGACCAGCCUUCAUUCCCC.................    249.973803
........................................................................................................UCGGACCAGGCUUAAUUCCCC.................    2595.787516
........................................................................................................UCGGACCAGGCUUCAUACCCC.................    615.957197
........................................................................................................UCGGACCAGGCAUCAUUCCCC.................    304.313191
........................................................................................................UCGGACCAGUCUUCAUUCCCC.................    1698.045668
........................................................................................................UCGGACCAGGAUUCAUUCCCC.................    3361.421404
........................................................................................................UCGAACCAGGCUUCAUUCCCC.................    1222.225559
........................................................................................................UCGGACCAUGCUUCAUUCCCC.................    2915.09744
........................................................................................................UCGGACCAGGCUGCAUUCCCC.................    494.068714
........................................................................................................UCGGACCAGGCUUCAUCCCCC.................    3088.3769
........................................................................................................UCGGACCAGGGUUCAUUCCCC.................    1443.742425
........................................................................................................UCGGACCAGGCUUCAAUCCCCU................    220.165543
........................................................................................................UCGGACUAGGCUUCAUUCCCC.................    1395.703481
........................................................................................................UCGGACCAAGCUUCAUUCCCC.................    754.854373
........................................................................................................UCGGACCAGGCUUCAUUCCCU.................    54756.65173
........................................................................................................UCGGACCAGGCUUCAUUCCUCU................    186.355772
........................................................................................................UCGGACCAGGCUACAUUCCCC.................    282.049971
........................................................................................................UCGGACCAGGCUUCAUUCA...................    774.926224
........................................................................................................UCCGACCAGGCUUCAUUCCCC.................    642.164282
........................................................................................................UCGGACCAGGCUUCAUUCUCC.................    3115.46708
........................................................................................................UCGGCCCAGGCUUCAUUCCCC.................    380.166369
........................................................................................................UCGGACCAGGCUUCAUUCU...................    5070.280857
........................................................................................................UCGGUCCAGGCUUCAUUCCCC.................    488.345507
........................................................................................................UCGGACCAGGCUUCAUCCC...................    590.988535
.......................................................................................................CUCGGACCAGGCUUCAUUCCCC.................    2369.518445
.......................................................................................................CUCGGACCAGGCUUCAUUCCC..................    8074.545384
......................................................................................................UCUCGGACCAGGCUUCAUUCC...................    66118.43634
......................................................................................................UCUCGGACCAGGCUUCAUUCCC..................    117.475902
......................................................................................................UCUCGGACCAGGCUUCAUUCCCC.................    133.426924
......................................................................................................UCUCGGACCAGGCUUCAUUC....................    86.021335
......................................................................................................UCUCGGACCAGUCUUCAUUCC...................    88.42751
......................................................................................................UCUCGGACCAGGCUUCAUUCU...................    500.66045
......................................................................................................UCUCGGACCAGGCUUCAUUCA...................    169.326233
......................................................................................................UCUCGGACCAUGCUUCAUUCC...................    87.88165
.....................................................................................................AUCUCGGACCAGGCUUCAUUCCCC.................    2698.974923
.....................................................................................................AUCUCGGACCAGGCUUCAUUC....................    170.31007
.......................GGAAUGUUGUCUGGUUCAAGGU.................................................................................................    197.844328
.......................GGAAUGUUGUCUGGUUCAA....................................................................................................    74.347792
.......................GGAAUGUUGUCUGGUUCAAG...................................................................................................    266.42226
.......................GGAAUGUUGUCUGGUUCAAGGA.................................................................................................    124.863184
.......................GGAAUGUUGUCUGGUUCAAGA..................................................................................................    82.643721
```

zma-MIR166e

Chromosome: chr5
Start: 195602229
End: 195602342
Strand: +
Strand bias: 1.0
Abundance bias: 1.0
MFE: -54.00

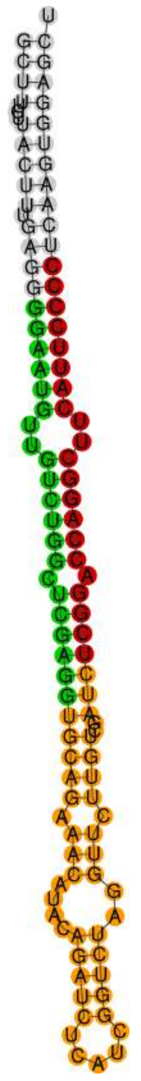

```
                    Star                                                            Mature
GCUUUCUUACUUUGAGGGGAAUGUUGUCUGGCUCGAGGUGCAGAAACAUACAGAUCUCAUCGGUCUAGGUUCUUGUCGAUCUCGGACCAGGCUUCAUUCCCCUCAAGUGGAGCU    3285763.2
((((...(((((.((((((((((..((((((.(((((((((((.(((....(((((.....)))))..))).))))..))))))).))))))..))))))))))))))))))).
.................................................................................UCGGACCAGGCUUCAUUCCCC............    2961896.0766
.................GGAAUGUUGUCUGGCUCGAGG............................................................................    8419.41475
...................................................................................GGACCAGGCUUCAUUCCCC............    850.718575
..................................................................................CGGACCAGGCUUCAUUCCCCU...........    267.040425
..................................................................................CGGACCAGGCUUCAUUCCCC............    1981.517186
.................................................................................UCGGACCAGGCUUCAUUCCC.............    25276.65239
.................................................................................UCGGACCAGGCUUCAUUCCCCU...........    18743.33647
.................................................................................UUGGACCAGGCUUCAUUCCCC............    6166.826765
.................................................................................UCGGACGAGGCUUCAUUCCCC............    170.447553
.................................................................................UCGGACCAGGCUUUAUUCCCC............    3533.163648
.................................................................................UCGGACCAGGCUUCAGUCCCC............    1007.658931
.................................................................................UCGGACCAGGCGUCAUUCCCC............    834.884155
.................................................................................UCGGACCAGGCUUCACUCCCC............    1291.80307
.................................................................................ACGGACCAGGCUUCAUUCCCC............    995.77943
.................................................................................UCGGACCAGGCUUCAUUCCGC............    519.213536
.................................................................................UCGGACCAGGCUUCCUUCCCC............    8766.353245
.................................................................................UCAGACCAGGCUUCAUUCCCC............    1351.88353
.................................................................................UCGGACCAGGCUUCAUUUCCC............    4299.307935
.................................................................................UCGGACCAGGCUUCAUUU...............    896.57008
.................................................................................UCGUACCAGGCUUCAUUCCCC............    3689.419818
.................................................................................UCGGACCAGGCCUCAUUCCCC............    1796.88093
.................................................................................UCGGACCAGGCUUCAUUCCCUU...........    209.06847
.................................................................................UCGGACCAGGCUUCCUUCCC.............    96.91076
.................................................................................CCGGACCAGGCUUCAUUCCCC............    1566.461868
.................................................................................UCGCACCAGGCUUCAUUCCCC............    563.824914
.................................................................................UCGGACCAGGUUUCAUUCCCC............    1530.66488
.................................................................................UCGGACCAGGCUUCAUUCCA.............    882.275967
.................................................................................UCGGACCAGGCUUCAUUCCCA............    19371.06785
.................................................................................UCGGACCAGGCUUCAUUCCCG............    1866.084944
.................................................................................UCGGACCAGGCUUCAUCC...............    213.397153
.................................................................................UCGGACCAGGCUUCGUUCCCC............    2381.191261
.................................................................................UCGGACCAGGCUUCAUUCACC............    1299.806243
.................................................................................UGGGACCAGGCUUCAUUCCCC............    548.708034
.................................................................................UCGGACCGGGCUUCAUUCCCC............    1473.378566
.................................................................................UCGGACAAGGCUUCAUUCCCC............    871.121313
.................................................................................UCGGACCUGGCUUCAUUCCCC............    644.270222
.................................................................................UCGGACCCGGCUUCAUUCCCC............    464.050619
.................................................................................UCGGACCAGACUUCAUUCCCC............    742.625098
.................................................................................UCGGACCAGGCUCCAUUCCCC............    1557.683496
.................................................................................UCUGACCAGGCUUCAUUCCCC............    3855.713456
.................................................................................UCGGACCAGGCUUCAAUCCCC............    6228.669769
.................................................................................UCGGACCAGGCUUCAUGCCCC............    742.940595
.................................................................................UCGGACCAGGCUUGAUUCCCC............    318.168133
.................................................................................UCGGACCAGGCUUCAUUCUC.............    223.097641
.................................................................................UCGGACCACGCUUCAUUCCCC............    177.12601
.................................................................................UCGGGCCAGGCUUCAUUCCCC............    2017.66487
.................................................................................UCGGACCAGGCUUCAUCCCC.............    201.411416
.................................................................................UCGGACCAGGCUUCAUUUC..............    791.817105
.................................................................................UCGGACCAGGCUUCAUUGCCC............    325.308415
.................................................................................UCGGAACAGGCUUCAUUCCCC............    305.306124
.................................................................................UAGGACCAGGCUUCAUUCCCC............    1004.401772
.................................................................................UCGGACCAGGCUUCAUUCCAC............    1433.934665
.................................................................................UCGGACCAGGCUUCAUUCGCC............    168.967216
.................................................................................UCGGACCAGGCUUCAUUACCC............    1394.886589
.................................................................................UCGGAUCAGGCUUCAUUCCCC............    1152.339525
.................................................................................GCGGACCAGGCUUCAUUCCCC............    1373.489964
.................................................................................UCGGACCAGGCUUCUUUCCCC............    1025.081502
.................................................................................UCGGACCAGCCUUCAUUCCCC............    249.973803
.................................................................................UCGGACCAGGCUUAAUUCCCC............    2595.787516
.................................................................................UCGGACCAGGCUUCAUACCCC............    615.957197
.................................................................................UCGGACCAGGCAUCAUUCCCC............    304.313191
.................................................................................UCGGACCAGUCUUCAUUCCCC............    1698.045668
.................................................................................UCGGACCAGGAUUCAUUCCCC............    3361.421404
.................................................................................UCGAACCAGGCUUCAUUCCCC............    1222.225559
.................................................................................UCGGACCAUGCUUCAUUCCCC............    2915.09744
.................................................................................UCGGACCAGGCUGCAUUCCCC............    494.068714
.................................................................................UCGGACCAGGCUUCAUCCCCC............    3088.3769
.................................................................................UCGGACCAGGGUUCAUUCCCC............    1443.742425
.................................................................................UCGGACCAGGCUUCAAUCCCCU...........    220.165543
.................................................................................UCGGACUAGGCUUCAUUCCCC............    1395.703481
.................................................................................UCGGACCAAGCUUCAUUCCCC............    754.854373
.................................................................................UCGGACCAGGCUUCAUUCCCU............    54756.65173
.................................................................................UCGGACCAGGCUUCAUUCCUCU...........    186.355772
.................................................................................UCGGACCAGGCUACAUUCCCC............    282.049971
.................................................................................UCGGACCAGGCUUCAUUCA..............    774.926224
.................................................................................UCCGACCAGGCUUCAUUCCCC............    642.164282
.................................................................................UCGGACCAGGCUUCAUUCUCC............    3115.46708
.................................................................................UCGGCCCAGGCUUCAUUCCCC............    380.166369
.................................................................................UCGGACCAGGCUUCAUUCU..............    5070.280857
.................................................................................UCGGUCCAGGCUUCAUUCCCC............    488.345507
.................................................................................UCGGACCAGGCUUCAUCCC..............    590.988535
................................................................................CUCGGACCAGGCUUCAUUCCCC............    2369.518445
................................................................................CUCGGACCAGGCUUCAUUCCC.............    8074.545384
...............................................................................UCUCGGACCAGGCUUCAUUCC..............    66118.43634
...............................................................................UCUCGGACCAGGCUUCAUUCCC.............    117.475902
...............................................................................UCUCGGACCAGGCUUCAUUCCCC............    133.426924
...............................................................................UCUCGGACCAGUCUUCAUUCC..............    88.42751
...............................................................................UCUCGGACCAGGCUUCAUUCU..............    500.66045
...............................................................................UCUCGGACCAGGCUUCAUUCA..............    169.326233
..............................................................................AUCUCGGACCAGGCUUCAUUCCCC............    2698.974923
..............................................................................AUCUCGGACCAGGCUUCAUUC...............    170.31007
...................AAUGUUGUCUGGCUCGAGGUG..........................................................................    1637.267923
..................GAAUGUUGUCUGGCUCGAGGU...........................................................................    1114.559003
.................GGAAUGUUGUCUGGCUCGA..............................................................................    128.259348
.................GGAAUGUUGUCUGGCUCGAG.............................................................................    168.802377
.................GGAAUGUUGGCUGGCUCGAGG............................................................................    1308.734216
.................GGAAUGUUGUCUGGCUCGAGA............................................................................    151.39844
................GGGAAUGUUGUCUGGCUCGAG.............................................................................    206.880563
```

zma-MIR166g

Chromosome: chr1
Start: 13782613
End: 13782780
Strand: +
Strand bias: 1.0
Abundance bias: 1.0
MFE: -75.60

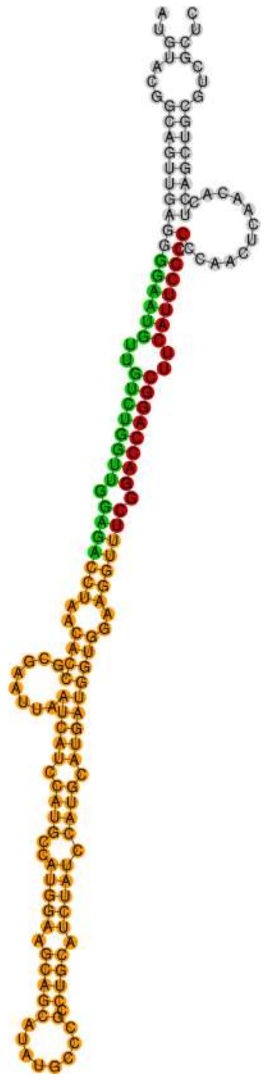

```
                 Star                                                                                               Mature
AUGUACGGCAGUUGAGGGGAAUGUUGUCUGGUUGGAGACCUAACACCGCGAAUUAAUCAUCCAUGCCAUGGAAGCAGCAUAUGCCCGCCUGCAUCUAUCCAUGCAUGAUGGUGGAAGGUUUCGGACCAGGCUUCAUUCCCCCCAACUCAACACUCAGCUGCGUCGCUC   3482676.9
..((...((((((((((((((((..((((((((.(((((((..((((........(((((.((((..(((((.(((((........).)))).)))))).)))).)))))))))..))))))).))))))))..))))))))............))))))))...))..
........................................................................................................................UCGGACCAGGCUUCAUUCCCC...........................   2961896.0766
.................GGAAUGUUGUCUGGUUGGAGA..................................................................................................................................   1148.868806
..........................................................................................................................GGACCAGGCUUCAUUCCCC...........................   850.718575
.........................................................................................................................CGGACCAGGCUUCAUUCCCC...........................   1981.517186
........................................................................................................................UCGGACCAGGCUUCAUUCCC............................   25276.65239
........................................................................................................................UCGGACCAGGCUUCAUUCCCCC..........................   1735.661278
........................................................................................................................UUGGACCAGGCUUCAUUCCCC...........................   6166.826765
........................................................................................................................UCGGACGAGGCUUCAUUCCCC...........................   170.447553
........................................................................................................................UCGGACCAGGCUUUAUUCCCC...........................   3533.163648
........................................................................................................................UCGGACCAGGCUUCAGUCCCC...........................   1007.658931
........................................................................................................................UCGGACCAGGCGUCAUUCCCC...........................   834.884155
........................................................................................................................UCGGACCAGGCUUCACUCCCC...........................   1291.80307
........................................................................................................................ACGGACCAGGCUUCAUUCCCC...........................   995.77943
........................................................................................................................UCGGACCAGGCUUCAUUCCGC...........................   519.213536
........................................................................................................................UCGGACCAGGCUUCCUUCCCC...........................   8766.353245
........................................................................................................................UCAGACCAGGCUUCAUUCCCC...........................   1351.88353
........................................................................................................................UCGGACCAGGCUUCAUUUCCC...........................   4299.307935
........................................................................................................................UCGGACCAGGCUUCAUUU..............................   896.57008
........................................................................................................................UCGUACCAGGCUUCAUUCCCC...........................   3689.419818
........................................................................................................................UCGGACCAGGCCUCAUUCCCC...........................   1796.88093
........................................................................................................................CCGGACCAGGCUUCAUUCCCC...........................   1566.461868
........................................................................................................................UCGCACCAGGCUUCAUUCCCC...........................   563.824914
........................................................................................................................UCGGACCAGGUUUCAUUCCCC...........................   1530.66488
........................................................................................................................UCGGACCAGGCUUCAUUCCA............................   882.275967
........................................................................................................................UCGGACCAGGCUUCAUUCCCA...........................   19371.06785
........................................................................................................................UCGGACCAGGCUUCAUUCCCG...........................   1866.084944
........................................................................................................................UCGGACCAGGCUUCAUCC..............................   213.397153
........................................................................................................................UCGGACCAGGCUUCGUUCCCC...........................   2381.191261
........................................................................................................................UCGGACCAGGCUUCAUUCACC...........................   1299.806243
........................................................................................................................UGGGACCAGGCUUCAUUCCCC...........................   548.708034
........................................................................................................................UCGGACCGGGCUUCAUUCCCC...........................   1473.378566
........................................................................................................................UCGGACAAGGCUUCAUUCCCC...........................   871.121313
........................................................................................................................UCGGACCUGGCUUCAUUCCCC...........................   644.270222
........................................................................................................................UCGGACCCGGCUUCAUUCCCC...........................   464.050619
........................................................................................................................UCGGACCAGACUUCAUUCCCC...........................   742.625098
........................................................................................................................UCGGACCAGGCUCCAUUCCCC...........................   1557.683496
........................................................................................................................UCUGACCAGGCUUCAUUCCCC...........................   3855.713456
........................................................................................................................UCGGACCAGGCUUCAAUCCCC...........................   6228.669769
........................................................................................................................UCGGACCAGGCUUCAUGCCCC...........................   742.940595
........................................................................................................................UCGGACCAGGCUUGAUUCCCC...........................   318.168133
........................................................................................................................UCGGACCAGGCUUCAUUCUC............................   223.097641
........................................................................................................................UCGGACCACGCUUCAUUCCCC...........................   177.12601
........................................................................................................................UCGGGCCAGGCUUCAUUCCCC...........................   2017.66487
........................................................................................................................UCGGACCAGGCUUCAUCCCC............................   201.411416
........................................................................................................................UCGGACCAGGCUUCAUUUC.............................   791.817105
........................................................................................................................UCGGACCAGGCUUCAUUGCCC...........................   325.308415
........................................................................................................................UCGGAACAGGCUUCAUUCCCC...........................   305.306124
........................................................................................................................UAGGACCAGGCUUCAUUCCCC...........................   1004.401772
........................................................................................................................UCGGACCAGGCUUCAUUCCAC...........................   1433.934665
........................................................................................................................UCGGACCAGGCUUCAUUCGCC...........................   168.967216
........................................................................................................................UCGGACCAGGCUUCAUUACCC...........................   1394.886589
........................................................................................................................UCGGAUCAGGCUUCAUUCCCC...........................   1152.339525
........................................................................................................................GCGGACCAGGCUUCAUUCCCC...........................   1373.489964
........................................................................................................................UCGGACCAGGCUUCUUUCCCC...........................   1025.081502
........................................................................................................................UCGGACCAGCCUUCAUUCCCC...........................   249.973803
........................................................................................................................UCGGACCAGGCUUAAUUCCCC...........................   2595.787516
........................................................................................................................UCGGACCAGGCUUCAUACCCC...........................   615.957197
........................................................................................................................UCGGACCAGGCAUCAUUCCCC...........................   304.313191
........................................................................................................................UCGGACCAGUCUUCAUUCCCC...........................   1698.045668
........................................................................................................................UCGGACCAGGAUUCAUUCCCC...........................   3361.421404
........................................................................................................................UCGAACCAGGCUUCAUUCCCC...........................   1222.225559
........................................................................................................................UCGGACCAUGCUUCAUUCCCC...........................   2915.09744
........................................................................................................................UCGGACCAGGCUGCAUUCCCC...........................   494.068714
........................................................................................................................UCGGACCAGGCUUCAUCCCCC...........................   3088.3769
........................................................................................................................UCGGACCAGGGUUCAUUCCCC...........................   1443.742425
........................................................................................................................UCGGACUAGGCUUCAUUCCCC...........................   1395.703481
........................................................................................................................UCGGACCAAGCUUCAUUCCCC...........................   754.854373
........................................................................................................................UCGGACCAGGCUUCAUUCCCU...........................   54756.65173
........................................................................................................................UCGGACCAGGCUACAUUCCCC...........................   282.049971
........................................................................................................................UCGGACCAGGCUUCAUUCA.............................   774.926224
........................................................................................................................UCCGACCAGGCUUCAUUCCCC...........................   642.164282
........................................................................................................................UCGGACCAGGCUUCAUUCUCC...........................   3115.46708
........................................................................................................................UCGGCCCAGGCUUCAUUCCCC...........................   380.166369
........................................................................................................................UCGGACCAGGCUUCAUUCU.............................   5070.280857
........................................................................................................................UCGGUCCAGGCUUCAUUCCCC...........................   488.345507
........................................................................................................................UCGGACCAGGCUUCAUCCC.............................   590.988535
.......................................................................................................................UUCGGACCAGGCUUCAUUC..............................   549.988645
.......................................................................................................................UUCGGACCAGGCUUCAUUCCCC...........................   194.236285
.......................................................................................................................UUCGGACCAGGCUUCAUUCC.............................   1196.755557
.......................................................................................................................UUCGGACCAGGCUUCAUUCCC............................   292333.56287
.......................................................................................................................UUCGGACCAGGCUUCCUUCCC............................   260.79545
.......................................................................................................................UUCGGACCAGGCUUCAUUCCCA...........................   325.754909
.......................................................................................................................UUCGGACCAGUCUUCAUUCCC............................   654.632519
.......................................................................................................................UUCGGACCAGGCUUCAUUCU.............................   188.659332
.......................................................................................................................UUCGGACCAUGCUUCAUUCCC............................   204.87594
.......................................................................................................................UUCGGACCAGGCUUCAUUCAC............................   127.238408
.......................................................................................................................UUCGGACCAGGCUUCAUUUCC............................   233.314464
.......................................................................................................................UUCGGACCAGGCUUCAUUCUC............................   451.13295
.......................................................................................................................UUCGGACCAGGCCUCAUUCCC............................   119.147222
.......................................................................................................................UUCGGACCAGGCUUAAUUCCC............................   166.52754
.......................................................................................................................UUCGGACCAGGCUUCAUUCCG............................   185.760058
.......................................................................................................................UUCGGACCAGGCUUCAUUCCA............................   2347.486747
.......................................................................................................................UUCGGACCAGGCUUCAUUCCU............................   6167.484099
.......................................................................................................................UUCGGACCAGGCUUUAUUCCC............................   206.792225
.......................................................................................................................UUCGGACCAGGAUUCAUUCCC............................   143.759502
.......................................................................................................................UUCGGACCAGGUUUCAUUCCC............................   128.503174
.......................................................................................................................UUCGGACCAGGCUUCAUCCCC............................   159.448734
......................................................................................................................UUUCGGACCAGGCUUCAUUCC.............................   268.596509
```

zma-MIR166i

```
Chromosome:    chr1
Start:         244940000
End:           244940132
Strand:        +
Strand bias:   1.0
Abundance bias:1.0
MFE:           -71.00
```

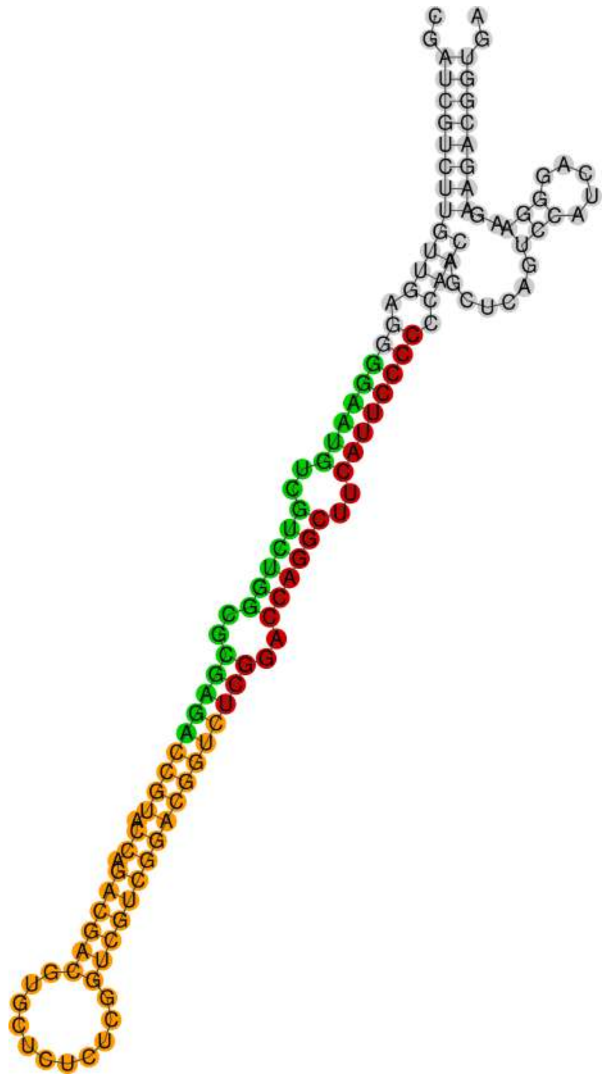

```
                         Star                                                          Mature
CGAUCGUCUUGUUGAGGGGAAUGUCGUCUGGCGCGAGACCGUACCAGACGACGUGCUCUCUCGGUCGUCGGACGGUCUCGGACCAGGCUUCAUUCCCCCCAACAGCUCAGUCCAUCAGGGAAGAAGACGGUGA    3251103.0
..((((((((((((.((((((((..((((((..(((((((((.((.((((((...........)))))))))))))))))..))))))..)))))))).)))).......(((.....)))..))))))))..
.............................................................................UCGGACCAGGCUUCAUUCCCC...................................    2961896.0766
.................GGAAUGUCGUCUGGCGCGAGA...............................................................................................    127.477788
...............................................................................GGACCAGGCUUCAUUCCCC...................................    850.718575
..............................................................................CGGACCAGGCUUCAUUCCC....................................    36.936678
..............................................................................CGGACCAGGCUUCAUUCCCC...................................    1981.517186
.............................................................................UCGGACCAGGCUUCAUUCCCCCA.................................    85.36061
.............................................................................UCGGACCAGGCUUCAUUCCC....................................    25276.65239
.............................................................................UCGGACCAGGCUUCAUUCCCCC..................................    1735.661278
.............................................................................UUGGACCAGGCUUCAUUCCCC...................................    6166.826765
.............................................................................UCGGACGAGGCUUCAUUCCCC...................................    170.447553
.............................................................................UCGGACCAGGCUUUAUUCCCC...................................    3533.163648
.............................................................................UCGGACCAGGCUUCAGUCCCC...................................    1007.658931
.............................................................................UCGGACCAGGCGUCAUUCCCC...................................    834.884155
.............................................................................UCGGACCAGGCUUCACUCCCC...................................    1291.80307
.............................................................................ACGGACCAGGCUUCAUUCCCC...................................    995.77943
.............................................................................UCGGACCAGGCUUCAUUCCGC...................................    519.213536
.............................................................................UCGGACCAGGCUUCCUUCCCC...................................    8766.353245
.............................................................................UCAGACCAGGCUUCAUUCCCC...................................    1351.88353
.............................................................................UCGGACCAGGCUUCAUUUCCC...................................    4299.307935
.............................................................................UCGGACCAGGCUUCAUUU......................................    896.57008
.............................................................................UCGUACCAGGCUUCAUUCCCC...................................    3689.419818
.............................................................................UCGGACCAGGCCUCAUUCCCC...................................    1796.88093
.............................................................................UCGGACCAGGCUUCCUUCCC....................................    96.91076
.............................................................................UCGGAGCAGGCUUCAUUCCCC...................................    61.3444
.............................................................................CCGGACCAGGCUUCAUUCCCC...................................    1566.461868
.............................................................................UCGCACCAGGCUUCAUUCCCC...................................    563.824914
.............................................................................UCGGACCAGGUUUCAUUCCCC...................................    1530.66488
.............................................................................UCGGACCAGGCUUCCUUCC.....................................    50.891188
.............................................................................UCGGACCAGGCUUCAUUCCA....................................    882.275967
.............................................................................UCGGACCAGGCUUCAUUCCG....................................    70.47754
.............................................................................UCGGACCAGGCUUCAUUCCCA...................................    19371.06785
.............................................................................UCGGACCAGGCUUCAUUCCCG...................................    1866.084944
.............................................................................UCGGACCAGGCUUCAUCC......................................    213.397153
.............................................................................UCGGACCAGGCUUCGUUCCCC...................................    2381.191261
.............................................................................UCGGACCAGGCUUCAUUCACC...................................    1299.806243
.............................................................................UGGGACCAGGCUUCAUUCCCC...................................    548.708034
.............................................................................UCGGACCGGGCUUCAUUCCCC...................................    1473.378566
.............................................................................UCGGACAAGGCUUCAUUCCCC...................................    871.121313
.............................................................................UCGGACCUGGCUUCAUUCCCC...................................    644.270222
.............................................................................UCGGACCCGGCUUCAUUCCCC...................................    464.050619
.............................................................................UCGGACCAGACUUCAUUCCCC...................................    742.625098
.............................................................................UCGGACCAGGCUCCAUUCCCC...................................    1557.683496
.............................................................................UCUGACCAGGCUUCAUUCCCC...................................    3855.713456
.............................................................................UCGGACCAGGCUUCAAUCCCC...................................    6228.669769
.............................................................................UCGGACCAGGCUUCAUGCCCC...................................    742.940595
.............................................................................UCGGACCAGGCUUGAUUCCCC...................................    318.168133
.............................................................................UCGGACCAGGCUUCAUUCUC....................................    223.097641
.............................................................................UCGGACCACGCUUCAUUCCCC...................................    177.12601
.............................................................................UCGGGCCAGGCUUCAUUCCCC...................................    2017.66487
.............................................................................UCGGACCAGGCUUCAUUCCCUC..................................    28.679633
.............................................................................UCGGACCAGGCUUCAUCCCC....................................    201.411416
.............................................................................UCGGACCAGGCUUCAUUUC.....................................    791.817105
.............................................................................UCGGACCAGGCUUCAUUGCCC...................................    325.308415
.............................................................................UCGGAACAGGCUUCAUUCCCC...................................    305.306124
.............................................................................UCGGACCAGGCUUCAUUUCC....................................    54.470543
.............................................................................UAGGACCAGGCUUCAUUCCCC...................................    1004.401772
.............................................................................UCGGACCAGGCUUCAUUCCUCC..................................    35.755782
.............................................................................UCGGACCAGGCUUCAUUCCAC...................................    1433.934665
.............................................................................UCGGACCAGGCUUCAUUCGCC...................................    168.967216
.............................................................................UCGGACCAGGCUUCAUUACCC...................................    1394.886589
.............................................................................UCGGAUCAGGCUUCAUUCCCC...................................    1152.339525
.............................................................................GCGGACCAGGCUUCAUUCCCC...................................    1373.489964
.............................................................................UCGGACCAGGCUUCUUUCCCC...................................    1025.081502
.............................................................................UCGGACCAGCCUUCAUUCCCC...................................    249.973803
.............................................................................UCGGACCAGGCUUAAUUCCCC...................................    2595.787516
.............................................................................UCGGACCAGGCUUCAUACCCC...................................    615.957197
.............................................................................UCGGACCAGGCAUCAUUCCCC...................................    304.313191
.............................................................................UCGGACCAGUCUUCAUUCCCC...................................    1698.045668
.............................................................................UCGGACCAGGAUUCAUUCCCC...................................    3361.421404
.............................................................................UCGAACCAGGCUUCAUUCCCC...................................    1222.225559
.............................................................................UCGGACCAUGCUUCAUUCCCC...................................    2915.09744
.............................................................................UCGGACCAGGCUGCAUUCCCC...................................    494.068714
.............................................................................UCGGACCAGGCUUCAUCCCCC...................................    3088.3769
.............................................................................UCGGACCAGGGUUCAUUCCCC...................................    1443.742425
.............................................................................UCGGACCAGGCUUCAUUA......................................    37.313501
.............................................................................UCGGACUAGGCUUCAUUCCCC...................................    1395.703481
.............................................................................UCGGACCAAGCUUCAUUCCCC...................................    754.854373
.............................................................................UCGGACCAGGCUUCAUUCCCU...................................    54756.65173
.............................................................................UCGGACCAGGCUACAUUCCCC...................................    282.049971
.............................................................................UCGGACCAGGCUUCAUUCA.....................................    774.926224
.............................................................................UCCGACCAGGCUUCAUUCCCC...................................    642.164282
.............................................................................UCGGACCAGGCUUCAUUCUCC...................................    3115.46708
.............................................................................UCGGCCCAGGCUUCAUUCCCC...................................    380.166369
.............................................................................UCGGACCAGGAUUCAUUCCC....................................    30.498268
.............................................................................UCGGACCAGGCUUCAUUCU.....................................    5070.280857
.............................................................................UCGGACCAGGCUUCAUUCG.....................................    53.77499
.............................................................................UCGGUCCAGGCUUCAUUCCCC...................................    488.345507
.............................................................................UCGGACCAGGCUUCAUCCC.....................................    590.988535
............................................................................CUCGGACCAGGCUUCAUUCCCC...................................    2369.518445
............................................................................CUCGGACCAGGCUUCAUUCCC....................................    8074.545384
...........................................................................UCUCGGACCAGGCUUCAUUCC.....................................    66118.43634
...........................................................................UCUCGGACCAGGCUUCAUUCCC....................................    117.475902
...........................................................................UCUCGGACCAGGCUUCAUUCCCC...................................    133.426924
...........................................................................UCUCGGACCAGGCUUCAUUC......................................    86.021335
...........................................................................UCUCGGACCAGUCUUCAUUCC.....................................    88.42751
...........................................................................UCUCGGACCAGGCUUCAUUCU.....................................    500.66045
...........................................................................UCUCGGACCAGGCUUCAUUCA.....................................    169.326233
...........................................................................UCUCGGACCAUGCUUCAUUCC.....................................    87.88165
```

zma-MIR166j

Chromosome: chr7
Start: 128589399
End: 128589545
Strand: +
Strand bias: 1.0
Abundance bias: 1.0
MFE: -63.00

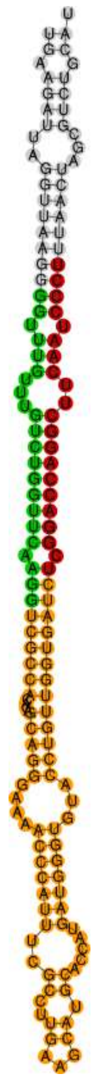

```
                      Star                                                                                           Mature
UGAAGAUUAGGUUAAGGGGUUUGUUUGUCUGGUUCAAGGUCGCCACACAGCAGGGAAAACCCAUUUCGCCUUGAAGCAUGCACCAUGAUGGGUGUACCUGUUGGUGAUCUCGGACCAGGCUUCAAUCCCUUUAACUAGCGUCUGCAU    184363.7
((.((((..((((((((((.(((...(((((((((.(((((((((....(((((....(((((((..((..((...)).)).....)))))))...)))))))))))))).)))))))))..))).))))))))))...)))).)).
.............................................................................................................UCGGACCAGGCUUCAAUCCCU.................    103467.8647
.................GGUUUGUUUGUCUGGUUCAAGG............................................................................................................    435.688965
...............................................................................................................GGACCAGGCUUCAAUCCCU.................    47.71572
..............................................................................................................CGGACCAGGCUUCAAUCC...................    2.50743
..............................................................................................................CGGACCAGGCUUCAAUCCCU.................    56.36684
.............................................................................................................UCGGACCAGGCUUCAAUCCCUUU...............    221.743793
.............................................................................................................UCGGACCAGGCUUCAAUCC...................    1039.58448
.............................................................................................................UCGGACCAGGCUUCAAUC....................    485.878826
.............................................................................................................UCGGACCAGGCUUCAAUCCC..................    6806.25201
.............................................................................................................UCGGACCAGGCUUCAAUCCCUU................    436.050205
.............................................................................................................UCGGACCAGGCUUCAAUCCCUUUA..............    5.35573
.............................................................................................................UCGGAACAGGCUUCAAUCCCU.................    5.311145
.............................................................................................................UCGGACCAGGCUUCAGUCCCU.................    139.121737
.............................................................................................................UCGGACCAGGCUUCACUCCCU.................    105.083018
.............................................................................................................UUGGACCAGGCUUCAAUCCCU.................    172.22749
.............................................................................................................UCGGACCAGGCUUCAAUCGCU.................    4.29329
.............................................................................................................UGGGACCAGGCUUCAAUCCCU.................    3.514192
.............................................................................................................UCGGACCAGGCUUCAAUCA...................    23.226533
.............................................................................................................UCGCACCAGGCUUCAAUCCCU.................    5.344995
.............................................................................................................UCGGUCCAGGCUUCAAUCCCU.................    10.963947
.............................................................................................................UCGGACCAGGCUUCAUUCCCUUUA..............    4.318222
.............................................................................................................UCGGACCAGGCUUCAGUCCC..................    7.057599
.............................................................................................................UAGGACCAGGCUUCAAUCCCU.................    23.536103
.............................................................................................................UCGGACCAUGCUUCAAUCCCU.................    50.298082
.............................................................................................................UCGGACCAGGCUUCAAUU....................    68.985944
.............................................................................................................UCGGACCGGGCUUCAAUCCCU.................    113.712733
.............................................................................................................UCGGACCAGGCUUCAUUCCCUU................    209.06847
.............................................................................................................ACGGACCAGGCUUCAAUCCCU.................    24.001311
.............................................................................................................UCGGACCAGGCCUCAAUCCCU.................    116.907837
.............................................................................................................UCGGACCAGGCUUCAACCCCU.................    114.112174
.............................................................................................................UCGGACCAGGCUUCAAUCCGU.................    11.38905
.............................................................................................................UCGGACUAGGCUUCAAUCCCU.................    256.076199
.............................................................................................................UCGGACCAGGCUUCAAUCCU..................    220.025166
.............................................................................................................UCGGACCAGGCUUCAAUCCA..................    44.82911
.............................................................................................................UCGGACCCGGCUUCAAUCCCU.................    6.69711
.............................................................................................................UCGGGCCAGGCUUCAAUCCCU.................    132.963788
.............................................................................................................UCGGACCAGGCUUUAAUCCCU.................    136.608835
.............................................................................................................UCGGACCAGGCUUCAAGCCCU.................    6.73853
.............................................................................................................UCGGACCAGGCUUCAAUACCU.................    34.658446
.............................................................................................................UCGGACCAGGCAUCAAUCCCU.................    2.684761
.............................................................................................................UCGGACCAGGCUUCUAUCCCU.................    5.588762
.............................................................................................................UCGGACCAGGCUUCAAUCCCUA................    174.551963
.............................................................................................................UCGGACCAGGCUUCAAUCCCUC................    58.739469
.............................................................................................................UCGGACCAGGCUUCAAUCCCUG................    13.14989
.............................................................................................................UCGGACCAGGGUUCAAUCCCU.................    2.838243
.............................................................................................................UCUGACCAGGCUUCAAUCCCU.................    65.616243
.............................................................................................................CCGGACCAGGCUUCAAUCCCU.................    109.887137
.............................................................................................................UCGGACCAGGCUUCAAACCCU.................    8.909681
.............................................................................................................UCGGACCAGGCUUAAAUCCCU.................    41.430807
.............................................................................................................UCGGACCAGGCUUCAAUCCCC.................    6228.669769
.............................................................................................................UCGGACCAGGCUUCAAUCCCA.................    3168.169293
.............................................................................................................UCGGACCAGGCUUCAAUCCCG.................    1661.346693
.............................................................................................................UCGGACCAGGCUACAAUCCCU.................    5.707179
.............................................................................................................UCGGAGCAGGCUUCAAUCCCU.................    4.27034
.............................................................................................................UCGGACCAGGCUCCAAUCCCU.................    127.613422
.............................................................................................................UCGGACCAGUCUUCAAUCCCU.................    19.200622
.............................................................................................................UCGUACCAGGCUUCAAUCCCU.................    78.542811
.............................................................................................................UCGGACCAGGCUUCAAUCU...................    285.399916
.............................................................................................................UCGGACCAGGCUUCGAUCCCU.................    138.027624
.............................................................................................................UCGAACCAGGCUUCAAUCCCU.................    21.231983
.............................................................................................................UCGGCCCAGGCUUCAAUCCCU.................    7.853308
.............................................................................................................UCGGACCAGGCUUCACUCCC..................    2.759936
.............................................................................................................UCGGACCAGGCUUCAUUCCCUUU...............    291.026563
.............................................................................................................UCGGACCAUGCUUCAAUCCC..................    3.27553
.............................................................................................................UCGGACCAGGCUUCAAUCUCU.................    98.562814
.............................................................................................................UCCGACCAGGCUUCAAUCCCU.................    9.169066
.............................................................................................................UCGGACCAGGCUUCAAUUCCU.................    190.612429
.............................................................................................................UCGGACCAAGCUUCAAUCCCU.................    13.765845
.............................................................................................................UCGGACCAGGAUUCAAUCCCU.................    13.872648
.............................................................................................................UCGGACCAGGUUUCAAUCCCU.................    36.624052
.............................................................................................................UCGGACCAGACUUCAAUCCCU.................    24.048557
.............................................................................................................UCGGACCUGGCUUCAAUCCCU.................    25.106938
.............................................................................................................UCGGACCAGGCUUCAAUCCAU.................    93.21301
.............................................................................................................UCGGAUCAGGCUUCAAUCCCU.................    72.608827
.............................................................................................................UCGGACCAGGCUUCAAUCCCCU................    220.165543
.............................................................................................................UCGGACCAGGCUUCAAUCACU.................    40.803387
.............................................................................................................UCGGACCAGGCUUCAUUCCCU.................    54756.65173
.............................................................................................................UCGGACCAGGCUUCAGUCC...................    8.138179
.............................................................................................................UCGGACCAGGCUUCAAUCCUU.................    112.963679
.............................................................................................................UCGGACGAGGCUUCAAUCCCU.................    13.059859
.............................................................................................................UCGGACCAGGCUUCCAUCCCU.................    130.104418
.............................................................................................................UCGGACCAGGCUUCAGUC....................    13.5645
.............................................................................................................UCAGACCAGGCUUCAAUCCCU.................    66.53424
.............................................................................................................GCGGACCAGGCUUCAAUCCCU.................    10.549925
.............................................................................................................UCGGACAAGGCUUCAAUCCCU.................    43.831925
.............................................................................................................UCGGACCAGGCUUCAAUUC...................    45.003994
............................................................................................................CUCGGACCAGGCUUCAAUCCC..................    58.597704
............................................................................................................CUCGGACCAGGCUUCAAUCCCU.................    78.909274
............................................................................................................CUCGGACCAGGCUUCAUUCCCU.................    10.622715
...........................................................................................................UCUCGGACCAGGCUUCAAUCCCU.................    2.365836
...........................................................................................................UCUCGGACCAGGCUUCAAUCC...................    15.430653
...........................................................................................................UCUCGGACCAGGCUUCAAUCCC..................    5.978707
...........................................................................................................UCUCGGACCAGGCUUCAGUCC...................    8.73091
..........................................................................................................AUCUCGGACCAGGCUUCAAUCCC..................    35.804657
..........................................................................................................AUCUCGGACCAGGCUUCAAUCCCU.................    437.062638
..........................................................................................................AUCUCGGACCAGGCUUCAUUCCCU.................    59.77035
.................GGUUUGUUUGUCUGGUUCAAG.............................................................................................................    6.831221
.................GGUUUGUUUGUCUGGUUCAAGGU...........................................................................................................    8.070199
```

zma-MIR166k

Chromosome: chr5
Start: 216483768
End: 216483933
Strand: -
Strand bias: 1.0
Abundance bias: 1.0
MFE: -87.60

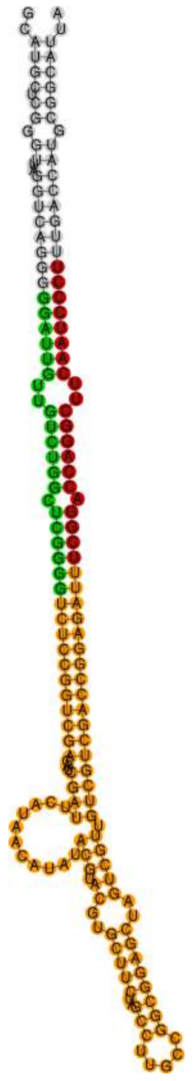

```
                      Star                                                                                                      Mature
GCAUGCUCGGGUUAGGUCAGGGGGAUUGUUGUCUGGCUCGGGGUCUCCGGUCGAGAUCGAUUCAUAACAUAUACGUACGUGCUUCUAGCCUUGCCGGCGGAGCUAGUCGUUGUCGUCGACCGGAGAUUUCGGACCAGGCUUCAAUCCCUUUGACCAUGCGGCAUUA    183629.5
..((((.((.((..((((((((((((((..((((((.(((((((((((((((((...((((...........(((.((..(((((..(((.....))))))))..))))).))))))))))))))))))))).))))))..)))))))))))))))).))))))..
................................................................................................................................UCGGACCAGGCUUCAAUCCCU.................    103467.8647
......................GGAUUGUUGUCUGGCUCGGGG...........................................................................................................................    92.781644
..................................................................................................................................GGACCAGGCUUCAAUCCCU.................    47.71572
.................................................................................................................................CGGACCAGGCUUCAAUCC...................    2.50743
.................................................................................................................................CGGACCAGGCUUCAAUCCCU.................    56.36684
.................................................................................................................................CGGACCAGGCUUCAUUCCCU.................    0.76742
.................................................................................................................................CGGACCAGGCUUCAAUCCCUC................    0.639285
................................................................................................................................UCGGACCAGGCUUCAAUCCCUUU...............    221.743793
................................................................................................................................UCGGACCAGGCUUCAAUCC...................    1039.58448
................................................................................................................................UCGGACCAGGCUUCAAUC....................    485.878826
................................................................................................................................UCGGACCAGGCUUCAAUCCC..................    6806.25201
................................................................................................................................UCGGACCAGGCUUCAAUCCCUU................    436.050205
................................................................................................................................UCGGAACAGGCUUCAAUCCCU.................    5.311145
................................................................................................................................UCGGACCAGGCUUCAGUCCCU.................    139.121737
................................................................................................................................UCGGACCAGGCUUCACUCCCU.................    105.083018
................................................................................................................................UUGGACCAGGCUUCAAUCCCU.................    172.22749
................................................................................................................................UCGGACCAGGCUUCAAUCGCU.................    4.29329
................................................................................................................................UGGGACCAGGCUUCAAUCCCU.................    3.514192
................................................................................................................................UCGGACCAGGCUUCAAUCA...................    23.226533
................................................................................................................................UCGCACCAGGCUUCAAUCCCU.................    5.344995
................................................................................................................................UCGGUCCAGGCUUCAAUCCCU.................    10.963947
................................................................................................................................UCGGACCAGGCUUCAGUCCC..................    7.057599
................................................................................................................................UAGGACCAGGCUUCAAUCCCU.................    23.536103
................................................................................................................................UCGGACCAUGCUUCAAUCCCU.................    50.298082
................................................................................................................................UCGGACCAGGCUUCAAUU....................    68.985944
................................................................................................................................UCGGACCGGGCUUCAAUCCCU.................    113.712733
................................................................................................................................UCGGACCAGGCUUCAUUCCCUU................    209.06847
................................................................................................................................ACGGACCAGGCUUCAAUCCCU.................    24.001311
................................................................................................................................UCGGACCAGGCCUCAAUCCCU.................    116.907837
................................................................................................................................UCGGACCAGGCUUCAACCCCU.................    114.112174
................................................................................................................................UCGGACCAGGCUUCAAUCCGU.................    11.38905
................................................................................................................................UCGGACUAGGCUUCAAUCCCU.................    256.076199
................................................................................................................................UCGGACCAGGCUUCAAUCCU..................    220.025166
................................................................................................................................UCGGACCAGGCUUCAAUCCA..................    44.82911
................................................................................................................................UCGGACCCGGCUUCAAUCCCU.................    6.69711
................................................................................................................................UCGGACCACGCUUCAAUCCCU.................    0.732446
................................................................................................................................UCGGGCCAGGCUUCAAUCCCU.................    132.963788
................................................................................................................................UCGGACCAGGCUUUAAUCCCU.................    136.608835
................................................................................................................................UCGGACCAGGCUUCAAUCCUUU................    1.361524
................................................................................................................................UCGGACCAGGCUUCAAGCCCU.................    6.73853
................................................................................................................................UCGGACCAGGCUUCAAUACCU.................    34.658446
................................................................................................................................UCGGACCAGGCAUCAAUCCCU.................    2.684761
................................................................................................................................UCGGACCAGGCUUCUAUCCCU.................    5.588762
................................................................................................................................UCGGACCAGGCUUCAAUCCCUA................    174.551963
................................................................................................................................UCGGACCAGGCUUCAAUCCCUC................    58.739469
................................................................................................................................UCGGACCAGGCUUCAAUCCCUG................    13.14989
................................................................................................................................UCGGACCAGGGUUCAAUCCCU.................    2.838243
................................................................................................................................UCUGACCAGGCUUCAAUCCCU.................    65.616243
................................................................................................................................UCGGACCAGCCUUCAAUCCCU.................    1.824596
................................................................................................................................CCGGACCAGGCUUCAAUCCCU.................    109.887137
................................................................................................................................UCGGACCAGGCUUCAAACCCU.................    8.909681
................................................................................................................................UCGGACCAGGCUUAAAUCCCU.................    41.430807
................................................................................................................................UCGGACCAGGCUUCAAUCCCC.................    6228.669769
................................................................................................................................UCGGACCAGGCUUCAAUCCCA.................    3168.169293
................................................................................................................................UCGGACCAGGCUUCAAUCCCG.................    1661.346693
................................................................................................................................UCGGACCAGGCUACAAUCCCU.................    5.707179
................................................................................................................................UCGGAGCAGGCUUCAAUCCCU.................    4.27034
................................................................................................................................UCGGACCAGGCUCCAAUCCCU.................    127.613422
................................................................................................................................UCGGACCAGUCUUCAAUCCCU.................    19.200622
................................................................................................................................UCGUACCAGGCUUCAAUCCCU.................    78.542811
................................................................................................................................UCGGACCAGGCUUCAAUCU...................    285.399916
................................................................................................................................UCGGACCAGGCUUCGAUCCCU.................    138.027624
................................................................................................................................UCGAACCAGGCUUCAAUCCCU.................    21.231983
................................................................................................................................UCGGCCCAGGCUUCAAUCCCU.................    7.853308
................................................................................................................................UCGGACCAGGCUUCACUCCC..................    2.759936
................................................................................................................................UCGGACCAGGCUUCAUUCCCUUU...............    291.026563
................................................................................................................................UCGGACCAUGCUUCAAUCCC..................    3.27553
................................................................................................................................UCGGACCAGGCUUCAAUCUCU.................    98.562814
................................................................................................................................UCCGACCAGGCUUCAAUCCCU.................    9.169066
................................................................................................................................UCGGACCAGGCUUCAAUUCCU.................    190.612429
................................................................................................................................UCGGACCAAGCUUCAAUCCCU.................    13.765845
................................................................................................................................UCGGACCAGGAUUCAAUCCCU.................    13.872648
................................................................................................................................UCGGACCAGGUUUCAAUCCCU.................    36.624052
................................................................................................................................UCGGACCAGACUUCAAUCCCU.................    24.048557
................................................................................................................................UCGGACCUGGCUUCAAUCCCU.................    25.106938
................................................................................................................................UCGGACCAGGCUUCAAUCCAU.................    93.21301
................................................................................................................................UCGGAUCAGGCUUCAAUCCCU.................    72.608827
................................................................................................................................UCGGACCAGGCUUCAAUCCCCU................    220.165543
................................................................................................................................UCGGACCAGGCGUCAAUCCCU.................    0.561676
................................................................................................................................UCGGACCAGGCUUCAAUCACU.................    40.803387
................................................................................................................................UCGGACCAGGCUUCAUUCCCU.................    54756.65173
................................................................................................................................UCGGACCAGGCUUCAGUCC...................    8.138179
................................................................................................................................UCGGACCAGGCUUCAAUCCUU.................    112.963679
................................................................................................................................UCGGACGAGGCUUCAAUCCCU.................    13.059859
................................................................................................................................UCGGACCAGGCUUCCAUCCCU.................    130.104418
................................................................................................................................UCGGACCAGGCUUCAGUC....................    13.5645
................................................................................................................................UCAGACCAGGCUUCAAUCCCU.................    66.53424
................................................................................................................................GCGGACCAGGCUUCAAUCCCU.................    10.549925
................................................................................................................................UCGGACAAGGCUUCAAUCCCU.................    43.831925
................................................................................................................................UCGGACCAGGCUUCAAUUC...................    45.003994
...............................................................................................................................UUCGGACCAGGCUUCAAUCCC..................    68.221188
...............................................................................................................................UUCGGACCAGGCUUCAAUCCCU.................    18.686705
...............................................................................................................................UUCGGACCAGGCUUCAUUCCCU.................    77.907981
...............................................................................................................................UUCGGACCAGGCUUCACUCCC..................    79.64526
...............................................................................................................................UUCGGACCAGGCUUCAGUCCC..................    93.37103
......................GGAUUGUUGUCUGGCUCGGG............................................................................................................................    2.597953
......................GGAUUGUUGUCUGGCUCG..............................................................................................................................    1.924927
......................GGAUUGUUGUCUGGCUCGG.............................................................................................................................    7.180118
```

zma-MIR166_N1

Chromosome: chrB73V4_ctg73
Start: 2440
End: 2619
Strand: +
Strand bias: 1.0
Abundance bias: 1.0
MFE: -67.80

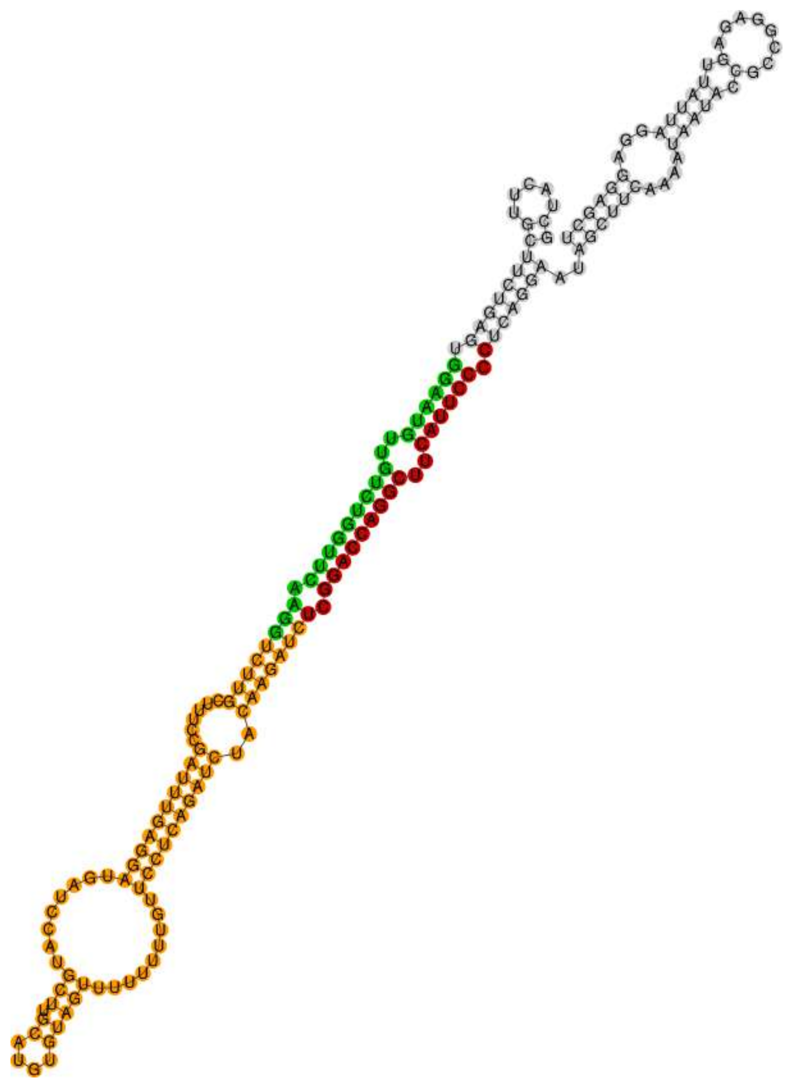

Star / Mature

```
GCUACUUGCUUCUGAGUGGAAUGUUGUCUGGUUCAAGGUCUUGCUUUCCGAUUUGAGGAUGAUCCAUGCUUGCAUGUGUAGUUUUUUUUGUUCCUCAGAUCUACAAGAUCUCGGACCAGGCUUCAUUCCCCUCAGGAAUAGCUUCAAAAUAAUACCGCCGGAGAGUUAUUAGGAGGAGCU   3283807.2
((.....))(((((((.((((((..(((((((((.((((((((......((((((((((........(((.((....)))))).........))))))))))..)))))))).)))))))))..)))))).)))))))..((((((....(((((.(........).)))))...))))))
```

Read	Abundance
UCGGACCAGGCUUCAUUCCCC (Mature)	2961896.0766
GGAAUGUUGUCUGGUUCAAGG (Star)	10309.99817
GGACCAGGCUUCAUUCCCC	850.718575
CGGACCAGGCUUCAUUCCCCU	267.040425
CGGACCAGGCUUCAUUCCCC	1981.517186
UCGGACCAGGCUUCAUUCCC	25276.65239
UCGGACCAGGCUUCAUUCCCCU	18743.33647
UUGGACCAGGCUUCAUUCCCC	6166.826765
UCGGACGAGGCUUCAUUCCCC	170.447553
UCGGACCAGGCUUUAUUCCCC	3533.163648
UCGGACCAGGCUUCAGUCCCC	1007.658931
UCGGACCAGGCGUCAUUCCCC	834.884155
UCGGACCAGGCUUCACUCCCC	1291.80307
ACGGACCAGGCUUCAUUCCCC	995.77943
UCGGACCAGGCUUCAUUCCGC	519.213536
UCGGACCAGGCUUCCUUCCCC	8766.353245
UCAGACCAGGCUUCAUUCCCC	1351.88353
UCGGACCAGGCUUCAUUUCCC	4299.307935
UCGGACCAGGCUUCAUUU	896.57008
UCGUACCAGGCUUCAUUCCCC	3689.419818
UCGGACCAGGCCUCAUUCCCC	1796.88093
UCGGACCAGGCUUCAUUCCCUU	209.06847
UCGGACCAGGCUUCCUUCCC	96.91076
CCGGACCAGGCUUCAUUCCCC	1566.461868
UCGCACCAGGCUUCAUUCCCC	563.824914
UCGGACCAGGUUUCAUUCCCC	1530.66488
UCGGACCAGGCUUCAUUCCA	882.275967
UCGGACCAGGCUUCAUUCCCA	19371.06785
UCGGACCAGGCUUCAUUCCCG	1866.084944
UCGGACCAGGCUUCAUCC	213.397153
UCGGACCAGGCUUCGUUCCCC	2381.191261
UCGGACCAGGCUUCAUUCACC	1299.806243
UGGGACCAGGCUUCAUUCCCC	548.708034
UCGGACCGGGCUUCAUUCCCC	1473.378566
UCGGACAAGGCUUCAUUCCCC	871.121313
UCGGACCUGGCUUCAUUCCCC	644.270222
UCGGACCCGGCUUCAUUCCCC	464.050619
UCGGACCAGACUUCAUUCCCC	742.625098
UCGGACCAGGCUCCAUUCCCC	1557.683496
UCUGACCAGGCUUCAUUCCCC	3855.713456
UCGGACCAGGCUUCAAUCCCC	6228.669769
UCGGACCAGGCUUCAUGCCCC	742.940595
UCGGACCAGGCUUGAUUCCCC	318.168133
UCGGACCAGGCUUCAUUCUC	223.097641
UCGGACCACGCUUCAUUCCCC	177.12601
UCGGGCCAGGCUUCAUUCCCC	2017.66487
UCGGACCAGGCUUCAUCCCC	201.411416
UCGGACCAGGCUUCAUUUC	791.817105
UCGGACCAGGCUUCAUUGCCC	325.308415
UCGGAACAGGCUUCAUUCCCC	305.306124
UAGGACCAGGCUUCAUUCCCC	1004.401772
UCGGACCAGGCUUCAUUCCAC	1433.934665
UCGGACCAGGCUUCAUUCGCC	168.967216
UCGGACCAGGCUUCAUUACCC	1394.886589
UCGGAUCAGGCUUCAUUCCCC	1152.339525
GCGGACCAGGCUUCAUUCCCC	1373.489964
UCGGACCAGGCUUCUUUCCCC	1025.081502
UCGGACCAGCCUUCAUUCCCC	249.973803
UCGGACCAGGCUUAAUUCCCC	2595.787516
UCGGACCAGGCUUCAUACCCC	615.957197
UCGGACCAGGCAUCAUUCCCC	304.313191
UCGGACCAGUCUUCAUUCCCC	1698.045668
UCGGACCAGGAUUCAUUCCCC	3361.421404
UCGAACCAGGCUUCAUUCCCC	1222.225559
UCGGACCAUGCUUCAUUCCCC	2915.09744
UCGGACCAGGCUGCAUUCCCC	494.068714
UCGGACCAGGCUUCAUCCCCC	3088.3769
UCGGACCAGGGUUCAUUCCCC	1443.742425
UCGGACCAGGCUUCAAUCCCCU	220.165543
UCGGACUAGGCUUCAUUCCCC	1395.703481
UCGGACCAAGCUUCAUUCCCC	754.854373
UCGGACCAGGCUUCAUUCCCU	54756.65173
UCGGACCAGGCUUCAUUCCUCU	186.355772
UCGGACCAGGCUACAUUCCCC	282.049971
UCGGACCAGGCUUCAUUCA	774.926224
UCCGACCAGGCUUCAUUCCCC	642.164282
UCGGACCAGGCUUCAUUCUCC	3115.46708
UCGGCCCAGGCUUCAUUCCCC	380.166369
UCGGACCAGGCUUCAUUCU	5070.280857
UCGGUCCAGGCUUCAUUCCCC	488.345507
UCGGACCAGGCUUCAUCCC	590.988535
CUCGGACCAGGCUUCAUUCCCC	2369.518445
CUCGGACCAGGCUUCAUUCCC	8074.545384
UCUCGGACCAGGCUUCAUUCC	66118.43634
UCUCGGACCAGGCUUCAUUCCC	117.475902
UCUCGGACCAGGCUUCAUUCCCC	133.426924
UCUCGGACCAGGCUUCAUUC	86.021335
UCUCGGACCAGUCUUCAUUCC	88.42751
UCUCGGACCAGGCUUCAUUCU	500.66045
UCUCGGACCAGGCUUCAUUCA	169.326233
UCUCGGACCAUGCUUCAUUCC	87.88165
AUCUCGGACCAGGCUUCAUUCCCC	2698.974923
AUCUCGGACCAGGCUUCAUUC	170.31007
GGAAUGUUGUCUGGUUCAAGGU	197.844328
GGAAUGUUGUCUGGUUCAA	74.347792
GGAAUGUUGUCUGGUUCAAG	266.42226
GGAAUGUUGUCUGGUUCAAGGA	124.863184
GGAAUGUUGUCUGGUUCAAGA	82.643721

zma-MIR166_N2

```
Chromosome:    chr1
Start:         226854045
End:           226854196
Strand:        -
Strand bias:   1.0
Abundance bias:1.0
MFE:           -58.70
```

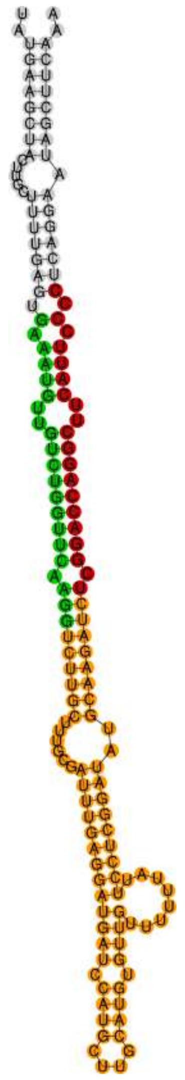

```
                                          Star                                                                                       Mature
UAUGAAGCUACUUGCUUUUGAGUGAAAUGUUGUCUGGUUCAAGGUCUUGCUUUGCGAUUUGAGGAUGAUCCAUGCUUGCAUGUGUUGUUUUUUAUUCCUCGGAUAUGCAAGAUCUCGGACCAGGCUUCAUUCCCCUCAGGAAUAGCUUCAAA    3272388.3
..((((((((.....(((((((.(.((((..(((((((((.(((((((((......(((((((((((((.((((....)))).)))).........)))))))))..)))))))))).)))))))))..)))).).))))))).))))))))..
..................................................................................................................UCGGACCAGGCUUCAUUCCCC.................    2961896.0766
.......................GAAAUGUUGUCUGGUUCAAGG............................................................................................................    274.22094
....................................................................................................................GGACCAGGCUUCAUUCCCC.................    850.718575
...................................................................................................................CGGACCAGGCUUCAUUCCCCU................    267.040425
...................................................................................................................CGGACCAGGCUUCAUUCCCC.................    1981.517186
..................................................................................................................UCGGACCAGGCUUCAUUCCC..................    25276.65239
..................................................................................................................UCGGACCAGGCUUCAUUCCCCU................    18743.33647
..................................................................................................................UUGGACCAGGCUUCAUUCCCC.................    6166.826765
..................................................................................................................UCGGACGAGGCUUCAUUCCCC.................    170.447553
..................................................................................................................UCGGACCAGGCUUUAUUCCCC.................    3533.163648
..................................................................................................................UCGGACCAGGCUUCAGUCCCC.................    1007.658931
..................................................................................................................UCGGACCAGGCGUCAUUCCCC.................    834.884155
..................................................................................................................UCGGACCAGGCUUCACUCCCC.................    1291.80307
..................................................................................................................ACGGACCAGGCUUCAUUCCCC.................    995.77943
..................................................................................................................UCGGACCAGGCUUCAUUCCGC.................    519.213536
..................................................................................................................UCGGACCAGGCUUCCUUCCCC.................    8766.353245
..................................................................................................................UCAGACCAGGCUUCAUUCCCC.................    1351.88353
..................................................................................................................UCGGACCAGGCUUCAUUUCCC.................    4299.307935
..................................................................................................................UCGGACCAGGCUUCAUUU....................    896.57008
..................................................................................................................UCGUACCAGGCUUCAUUCCCC.................    3689.419818
..................................................................................................................UCGGACCAGGCCUCAUUCCCC.................    1796.88093
..................................................................................................................UCGGACCAGGCUUCAUUCCCUU................    209.06847
..................................................................................................................UCGGACCAGGCUUCCUUCCC..................    96.91076
..................................................................................................................UCGGAGCAGGCUUCAUUCCCC.................    61.3444
..................................................................................................................CCGGACCAGGCUUCAUUCCCC.................    1566.461868
..................................................................................................................UCGCACCAGGCUUCAUUCCCC.................    563.824914
..................................................................................................................UCGGACCAGGUUUCAUUCCCC.................    1530.66488
..................................................................................................................UCGGACCAGGCUUCAUUCCCUUC...............    56.459352
..................................................................................................................UCGGACCAGGCUUCAUUCCA..................    882.275967
..................................................................................................................UCGGACCAGGCUUCAUUCCG..................    70.47754
..................................................................................................................UCGGACCAGGCUUCAUUCCCA.................    19371.06785
..................................................................................................................UCGGACCAGGCUUCAUUCCCG.................    1866.084944
..................................................................................................................UCGGACCAGGCUUCAUCC....................    213.397153
..................................................................................................................UCGGACCAGGCUUCGUUCCCC.................    2381.191261
..................................................................................................................UCGGACCAGGCUUCAUUCACC.................    1299.806243
..................................................................................................................UGGGACCAGGCUUCAUUCCCC.................    548.708034
..................................................................................................................UCGGACCGGGCUUCAUUCCCC.................    1473.378566
..................................................................................................................UCGGACAAGGCUUCAUUCCCC.................    871.121313
..................................................................................................................UCGGACCUGGCUUCAUUCCCC.................    644.270222
..................................................................................................................UCGGACCCGGCUUCAUUCCCC.................    464.050619
..................................................................................................................UCGGACCAGACUUCAUUCCCC.................    742.625098
..................................................................................................................UCGGACCAGGCUCCAUUCCCC.................    1557.683496
..................................................................................................................UCUGACCAGGCUUCAUUCCCC.................    3855.713456
..................................................................................................................UCGGACCAGGCUUCAAUCCCC.................    6228.669769
..................................................................................................................UCGGACCAGGCUUCAUGCCCC.................    742.940595
..................................................................................................................UCGGACCAGGCUUGAUUCCCC.................    318.168133
..................................................................................................................UCGGACCAGGCUUCAUUCUC..................    223.097641
..................................................................................................................UCGGACCACGCUUCAUUCCCC.................    177.12601
..................................................................................................................UCGGGCCAGGCUUCAUUCCCC.................    2017.66487
..................................................................................................................UCGGACCAGGCUUCAUCCCC..................    201.411416
..................................................................................................................UCGGACCAGGCUUCAUUUC...................    791.817105
..................................................................................................................UCGGACCAGGCUUCAUUGCCC.................    325.308415
..................................................................................................................UCGGAACAGGCUUCAUUCCCC.................    305.306124
..................................................................................................................UAGGACCAGGCUUCAUUCCCC.................    1004.401772
..................................................................................................................UCGGACCAGGCUUCAUUCCAC.................    1433.934665
..................................................................................................................UCGGACCAGGCUUCAUUCGCC.................    168.967216
..................................................................................................................UCGGACCAGGCUUCAUUACCC.................    1394.886589
..................................................................................................................UCGGAUCAGGCUUCAUUCCCC.................    1152.339525
..................................................................................................................GCGGACCAGGCUUCAUUCCCC.................    1373.489964
..................................................................................................................UCGGACCAGGCUUCUUUCCCC.................    1025.081502
..................................................................................................................UCGGACCAGCCUUCAUUCCCC.................    249.973803
..................................................................................................................UCGGACCAGGCUUAAUUCCCC.................    2595.787516
..................................................................................................................UCGGACCAGGCUUCAUACCCC.................    615.957197
..................................................................................................................UCGGACCAGGCAUCAUUCCCC.................    304.313191
..................................................................................................................UCGGACCAGUCUUCAUUCCCC.................    1698.045668
..................................................................................................................UCGGACCAGGAUUCAUUCCCC.................    3361.421404
..................................................................................................................UCGAACCAGGCUUCAUUCCCC.................    1222.225559
..................................................................................................................UCGGACCAUGCUUCAUUCCCC.................    2915.09744
..................................................................................................................UCGGACCAGGCUGCAUUCCCC.................    494.068714
..................................................................................................................UCGGACCAGGCUUCAUCCCCC.................    3088.3769
..................................................................................................................UCGGACCAGGGUUCAUUCCCC.................    1443.742425
..................................................................................................................UCGGACCAGGCUUCAAUCCCCU................    220.165543
..................................................................................................................UCGGACUAGGCUUCAUUCCCC.................    1395.703481
..................................................................................................................UCGGACCAAGCUUCAUUCCCC.................    754.854373
..................................................................................................................UCGGACCAGGCUUCAUUCCCU.................    54756.65173
..................................................................................................................UCGGACCAGGCUUCAUUCCUCU................    186.355772
..................................................................................................................UCGGACCAGGCUACAUUCCCC.................    282.049971
..................................................................................................................UCGGACCAGGCUUCAUUCA...................    774.926224
..................................................................................................................UCCGACCAGGCUUCAUUCCCC.................    642.164282
..................................................................................................................UCGGACCAGGCUUCAUUCUCC.................    3115.46708
..................................................................................................................UCGGCCCAGGCUUCAUUCCCC.................    380.166369
..................................................................................................................UCGGACCAGGCUUCAUUCU...................    5070.280857
..................................................................................................................UCGGUCCAGGCUUCAUUCCCC.................    488.345507
..................................................................................................................UCGGACCAGGCUUCAUCCC...................    590.988535
.................................................................................................................CUCGGACCAGGCUUCAUUCCCC.................    2369.518445
.................................................................................................................CUCGGACCAGGCUUCAUUCCC..................    8074.545384
................................................................................................................UCUCGGACCAGGCUUCAUUCC...................    66118.43634
................................................................................................................UCUCGGACCAGGCUUCAUUCCC..................    117.475902
................................................................................................................UCUCGGACCAGGCUUCAUUCCCC.................    133.426924
................................................................................................................UCUCGGACCAGGCUUCAUUC....................    86.021335
................................................................................................................UCUCGGACCAGUCUUCAUUCC...................    88.42751
................................................................................................................UCUCGGACCAGGCUUCAUUCU...................    500.66045
................................................................................................................UCUCGGACCAGGCUUCAUUCA...................    169.326233
................................................................................................................UCUCGGACCAUGCUUCAUUCC...................    87.88165
...............................................................................................................AUCUCGGACCAGGCUUCAUUCCCC.................    2698.974923
...............................................................................................................AUCUCGGACCAGGCUUCAUUC....................    170.31007
...............................................................................................................AUCUCGGACCAGGCUUCAUUCCCU.................    59.77035
........................AAAUGUUGUCUGGUUCAAGGUC..........................................................................................................    92.312642
```

zma-MIR166_N3

Chromosome: chr4
Start: 181111971
End: 181112251
Strand: +
Strand bias: 1.0
Abundance bias:0.996
MFE: -98.10

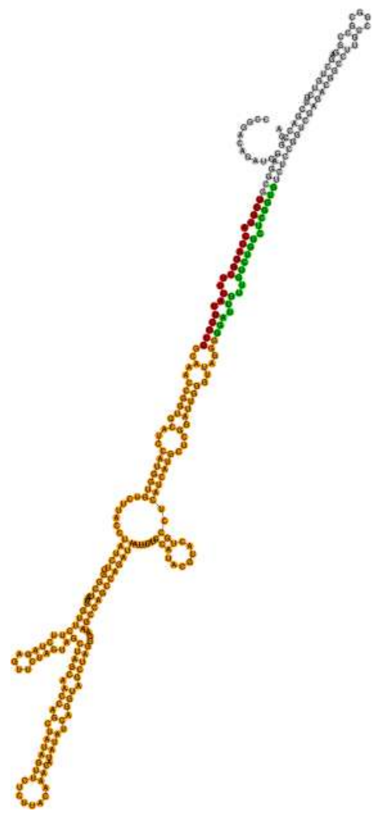

```
                 Mature                                                                                                                                                                                                              Star
CCGGACAGAUCGAGGCGUCGGACCAGGCUUCAUUCCUCGCAAACCGGUGCAUCCAUGGUGUCUUACCUAUCUCGGCUCAUGGUUCUUCUAGAGUUCUAGUAGCUAGCAACCAGCUAUAGUUCUCUUACAAACAUAUAUCAGGUAGCUAGGGAUAGCCAGCCAGAUAAUUUCAUGCAUACGUACUGCCUCAUCAUGCUCGGAUUGGGUUAGGGGGAUUGUUGUCUGGCUCGGUGUCUCCGGUCGAGACGGCCUUGCCGGCGCCGGAGCUGUCGUCGACCGGA   128559.4
...........((((((((((.((((((..((.(((((..(..(((((.(...(((((((.......(((((.((((...((((((.((((....)))).))(((((.(((.(.(((((((........))).)))).).))).)))))....))))))))))))).......(((.......)))..)))))))...).)))))..)..))))).))..)))))).))))))))))(((((((((((((((.((....)).)).)))))).)))))))..
.................UCGGACCAGGCUUCAUUCCUC...................................................................................................................................................................................................................................................   104300.9535
....................................................................................................................................................................................................................GGAUUGUUGUCUGGCUCGGUG................................................   497.319142
...............CGUCGGACCAGGCUUCAUUCC.....................................................................................................................................................................................................................................................   36.122784
................GUCGGACCAGGCUUCAUUCCC....................................................................................................................................................................................................................................................   147.622453
................GUCGGACCAGGCUUCAUUCCCC...................................................................................................................................................................................................................................................   82.48096
.................UCGGACCAGGCUUCAUUCCGC...................................................................................................................................................................................................................................................   519.213536
.................UCGGACCAGGCUUCAUUU......................................................................................................................................................................................................................................................   896.57008
.................UCGGACCAGGCUUUAUUCCUC...................................................................................................................................................................................................................................................   197.60323
.................UCGGACCAGGCUUCACUCCUC...................................................................................................................................................................................................................................................   79.64383
.................UCGGACCAGGCUUCAAUCCU....................................................................................................................................................................................................................................................   220.025166
.................UCGGACCAUGCUUCAUUCCUC...................................................................................................................................................................................................................................................   32.016302
.................UCGGACCAGGCUUCAUUUCUC...................................................................................................................................................................................................................................................   185.599463
.................UCGGACCAGGCUUCCUUCC.....................................................................................................................................................................................................................................................   50.891188
.................UCGGACCAGGCUUCAUUCCA....................................................................................................................................................................................................................................................   882.275967
.................UCGGACCAGGCUUCAUUCCG....................................................................................................................................................................................................................................................   70.47754
.................UCGGACCAGGCUGCAUUCCU....................................................................................................................................................................................................................................................   56.370578
.................UCGGACCAGGCUUCAUCC......................................................................................................................................................................................................................................................   213.397153
.................UCUGACCAGGCUUCAUUCCUC...................................................................................................................................................................................................................................................   52.897801
.................UCGGACCAGGCCUCAUUCCUC...................................................................................................................................................................................................................................................   102.500851
.................UCGGACCAGGCUUAAUUCCUC...................................................................................................................................................................................................................................................   81.513964
.................UCGGAUCAGGCUUCAUUCCUC...................................................................................................................................................................................................................................................   73.732414
.................UCGGACCAGGCUUCAUUCUUC...................................................................................................................................................................................................................................................   657.246487
.................UCGGACCAGGCUUCCUUCCUC...................................................................................................................................................................................................................................................   670.884554
.................UCGGACCAGGCUUCAUUCAUC...................................................................................................................................................................................................................................................   40.399994
.................UCGGACAAGGCUUCAUUCCUC...................................................................................................................................................................................................................................................   33.843552
.................UCGGACCAGGCUUCAUUCUU....................................................................................................................................................................................................................................................   588.635931
.................UCGUACCAGGCUUCAUUCCUC...................................................................................................................................................................................................................................................   49.710609
.................UCGGACCAGGCUUCGUUCCUC...................................................................................................................................................................................................................................................   123.853541
.................UCGGACCAGGCUUCAUUUC.....................................................................................................................................................................................................................................................   791.817105
.................UCAGACCAGGCUUCAUUCCUC...................................................................................................................................................................................................................................................   50.213245
.................UCGGGCCAGGCUUCAUUCCUC...................................................................................................................................................................................................................................................   93.281483
.................UCGGACCAGGCUUCUUUCCUC...................................................................................................................................................................................................................................................   33.4535
.................UCGGACCAGGCUUCAUUUCU....................................................................................................................................................................................................................................................   316.908588
.................UCGGACCAGGCUUCAUUCCUG...................................................................................................................................................................................................................................................   64.577884
.................UCGGACCAGGCUUCAUUCCUA...................................................................................................................................................................................................................................................   1443.928467
.................UCGGACCAGGCUUCAUUCCUU...................................................................................................................................................................................................................................................   4536.935147
.................UCGGACCAGGCUUCAUUCCUCC..................................................................................................................................................................................................................................................   35.755782
.................UCGGACCAGGCUUCAUUCCCCGC.................................................................................................................................................................................................................................................   76.126509
.................UCGGACCAGGCUUCAUUCCAC...................................................................................................................................................................................................................................................   1433.934665
.................UCGGACCAGGUUUCAUUCCUC...................................................................................................................................................................................................................................................   38.046818
.................UCGGACCAGGCUUCAUUCCCCGCA................................................................................................................................................................................................................................................   39.83337
.................UCGGACCGGGCUUCAUUCCUC...................................................................................................................................................................................................................................................   85.2778
.................UCGGACUAGGCUUCAUUCCUC...................................................................................................................................................................................................................................................   88.53342
.................UCGGACCAGGCUUCAUCCCUC...................................................................................................................................................................................................................................................   108.432302
.................UCGGACCAGGCUUCAUUACUC...................................................................................................................................................................................................................................................   42.638267
.................UCGGACCAGGCUCCAUUCCUC...................................................................................................................................................................................................................................................   98.228261
.................UCGGACCAGGCUUCAUUA......................................................................................................................................................................................................................................................   37.313501
.................CCGGACCAGGCUUCAUUCCUC...................................................................................................................................................................................................................................................   86.828145
.................UCGGACCAGGAUUCAUUCCUC...................................................................................................................................................................................................................................................   101.181944
.................UUGGACCAGGCUUCAUUCCUC...................................................................................................................................................................................................................................................   129.840899
.................UCGGACCAGGCUUCAUUCCUCU..................................................................................................................................................................................................................................................   186.355772
.................UCGGACCAGGCUUCAUUCA.....................................................................................................................................................................................................................................................   774.926224
.................UCGGACCAGGCUUCAUUCU.....................................................................................................................................................................................................................................................   5070.280857
.................UCGGACCAGGCUUCAUUCG.....................................................................................................................................................................................................................................................   53.77499
.................UCGGACCAGGCUUCAUCCC.....................................................................................................................................................................................................................................................   590.988535
..................CGGACCAGGCUUCAUUCCUC...................................................................................................................................................................................................................................................   65.633058
...................GGACCAGGCUUCAUUCCUC...................................................................................................................................................................................................................................................   110.36317
....................................................................................................................................................................................................................GGAUUGUUGUCUGGCUCGGU.................................................   31.585184
```

zma-MIR167a

Chromosome:	chr3
Start:	119392620
End:	119392813
Strand:	+
Strand bias:	1.0
Abundance bias:	1.0
MFE:	-90.60

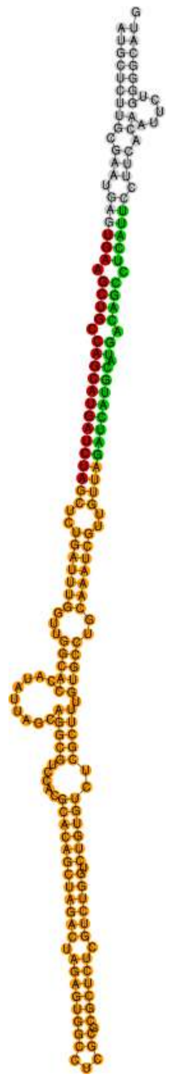

Mature Star

AUGCUCUUGCGAAUGAGUGAAGCUGCCAGCAUGAUCUAGCUCUGAUUUGGUUGGCACCAUAUUAGCAGGCGUCCACGCACAGCUAGACUAGAGUGGCCUCGCGCGCUCUCGUCUGGUCUGUGUCUCGCUUUGUGCCUGCAAAUCGUUGUUAGAUCAUGCAUGACAGCCUCAUUCCUUCACAAUUCUGGGGCAUG	128518.
((((((((.(((.((((((.((((.((((((((((((((..(((((((...(((((.........(((((.....(((((((((((.((((((((...)).)))))).)))))).))))))..))))).)))))..)))))))..)))))))))))).)).)))).)))))).))).))....))))))).	
.................UGAAGCUGCCAGCAUGAUCUA..	99232.2202
....GAUCAUGCAUGACAGCCUCAUU.....................	536.070655
................GUGAAGCUGCCAGCAUGAUCUA..	5.71994
.................UGAAGCUGCCAGCAUGAUCUAG...	313.877669
.................UGAAGCUGCCAGCAUGAUCU...	10812.468169
.................UGAAGCUGCCAGCAUGAU...	853.107838
.................UGAAGCUGCCAGCAUGAUC..	7600.3824
.................UGAAGCUGCCAGCAUGAUCUAGC..	30.35982
.................UGAAGCUGCCAGCAUAAUCUA..	24.860265
.................UGAAGCAGCCAGCAUGAUCUA..	9.565033
.................UGAAGCCGCCAGCAUGAUCUA..	77.61399
.................UGAAGCUGCCAGCAUUAUCUA..	39.742856
.................UGAAGCUGCUAGCAUGAUCUA..	145.032102
.................UGAAGCUGCCAGCCUGAUCU...	56.97454
.................UGAAACUGCCAGCAUGAUCUA..	12.369591
.................UGAAGCUGCCAGCAUGAUCGA..	39.428725
.................UGAAGCUGCCAGCAUGCUCU...	11.36722
.................UGAAGCUGCCAGCAUCAUCUA..	20.250455
.................UGAAGCUGUCAGCAUGAUCU...	8.12898
.................UGAAGCUGCCAGCAUGAGC..	31.62458
.................AGAAGCUGCCAGCAUGAUCUA..	20.827442
.................UGAAGCUGCCAGCAUGAUUU...	303.296332
.................UGAAGCUGCCCGCAUGAUCUA..	120.300014
.................UGAAGCUGCGAGCAUGAUCUA..	16.458766
.................UGAAGUUGCCAGCAUGAUCUA..	107.669343
.................UGAAGCUUCCAGCAUGAUCUA..	90.178542
.................UGAAGAUGCCAGCAUGAUCUA..	91.203955
.................UGAAGCUGCCAGCAUGAUUUA..	55.257578
.................UGAAGCUGCCAGCAUGGUCUA..	71.665755
.................UGAAGCUGCCAGCAUGAUCUAC...	30.211429
.................UGAAGCUGCCAGCAUGAUCUAU...	152.958622
.................UGAAGCUGCCAACAUGAUCUA..	14.012034
.................UGAAGCUGCCAGCAUGAUAU...	7.647396
.................UGAAGCUGCCAGCUUGAUCUA..	5.37231
.................UGAAGCUGCCAGCAUGAUCCA..	608.974265
.................CGAAGCUGCCAGCAUGAUCUA..	94.101388
.................UGAAGCUCCCAGCAUGAUCUA..	39.090401
.................UGAAGCUGCCAGCAUGAUCUUG...	102.16566
.................UGAAGCUGUCAGCAUGAUCUA..	141.816993
.................UGAAGCUGCCAGCAUGUUCUA..	16.219033
.................UGAAGCUGCCAGCAUGACC..	89.30017
.................UGAAGCUGACAGCAUGAUCUA..	83.228023
.................UGAAGCUGCCAGCGUGAUCUA..	55.803821
.................UGAAGCUGCCAGCAUGAUGUA..	22.31246
.................UGAAGCUGCCGGCAUGAUCUA..	67.32297
.................UGAAGCGGCCAGCAUGAUCUA..	24.348454
.................UGAGGCUGCCAGCAUGAUCUA..	72.665007
.................UGAAUCUGCCAGCAUGAUCUA..	59.432581
.................UGAAGCUGCCAGCAUGAUCUU..	1312.566292
.................UGAAGCUGCCAGCAUGAGCUA..	16.6244
.................UGAUGCUGCCAGCAUGAUCUA..	3.839524
.................UGAAGCUGCCAGCAUGCUCUA..	226.868906
.................UGAACCUGCCAGCAUGAUCUA..	23.529588
.................UGACGCUGCCAGCAUGAUCUA..	39.52832
.................UGGAGCUGCCAGCAUGAUCUA..	56.768044
.................UGAAGCUGCUAGCAUGAUCU...	7.50062
.................UGUAGCUGCCAGCAUGAUCUA..	3.840756
.................GGAAGCUGCCAGCAUGAUCUA..	35.595045
.................UGAAGCUGCCAUCAUGAUCUA..	33.737431
.................UGAAGCUGCCAGCAUGAUCUCG...	59.21133
.................UGAAGCUGCCUGCAUGAUCUA..	7.312193
.................UGAAGCUGCCAGCCUGAUC..	268.02408
.................UGAAGCUGCCCGCAUGAUCU...	6.52728
.................UGAAGCUGCCAGCAGGAUCUA..	6.926469
.................UAAAGCUGCCAGCAUGAUCUA..	14.465838
.................UGAAGCUGCCAGUAUGAUCUA..	33.534444
.................UCAAGCUGCCAGCAUGAUCUA..	5.839388
.................UGAAGCUGCCAGCAUGAUCC...	701.157019
.................UGAAGCUGCCAGCAUGAUCA...	410.926106
.................UGAAGCUGCCAGCAUGAUCG...	140.794267
.................UGAAGCUGCCAGCAUGAACUA..	4.776826
.................UGAAGCUGCCAGAAUGAUCUA..	62.469389
.................UGAAGCUGCCACCAUGAUCUA..	20.888063
.................UGAAGCUGCCAGCCUGAUCUA..	396.633176
.................UGAAGCUGCCAGCAUGAUAUA..	124.74369
.................UGAAGCUGGCAGCAUGAUCUA..	10.77235
.................UUAAGCUGCCAGCAUGAUCUA..	54.270944
.................UGAAGCUGCCAGCAUGACCUA..	63.033499
.................UGAAGCUGCCAGCAUGAUCUC..	366.816998
.................UGAAGCUGCCAGCACGAUCUA..	54.923415
.................UGCAGCUGCCAGCAUGAUCUA..	100.5801
.................UGAAGCUACCAGCAUGAUCUA..	40.101431
.................UGAAGCUGCCAGCAUGAUA..	100.890336
.................UGAAGCUGCCAGCAUGAUU..	496.456597
.................UGAAGCUGCCAGCAUGAUCAA..	13.14109
.................UGAAGCUGCAAGCAUGAUCUA..	102.643988
..................GAAGCUGCCAGCAUGAUC..	17.82409
..................GAAGCUGCCAGCAUGAUCUA..	313.394184
..................GAAGCUGCCAGCAUGAUCU...	22.646317
..................GAAGCUGCCAGCAUGAUCCA..	3.66002
...................AAGCUGCCAGCAUGAUCUA..	10.008406
...................AAGCUGCCAGCAUGAUCUAGC..	93.790949
....................AGCUGCCAGCAUGAUCUA..	6.96595
....AGAUCAUGCAUGACAGCCUCAUU.....................	32.52693
....GAUCAUGCAUGACAGCCUCAU......................	6.184211
....UAUCAUGCAUGACAGCCUCAUU.....................	5.21425
....AUCAUGCAUGACAGCCUCAUU.....................	94.55223
....UCAUGCAUGACAGCCUCAUU.....................	4.68806

zma-MIR167g

Chromosome: chr3
Start: 119394587
End: 119394825
Strand: +
Strand bias: 1.0
Abundance bias: 0.997
MFE: -123.20

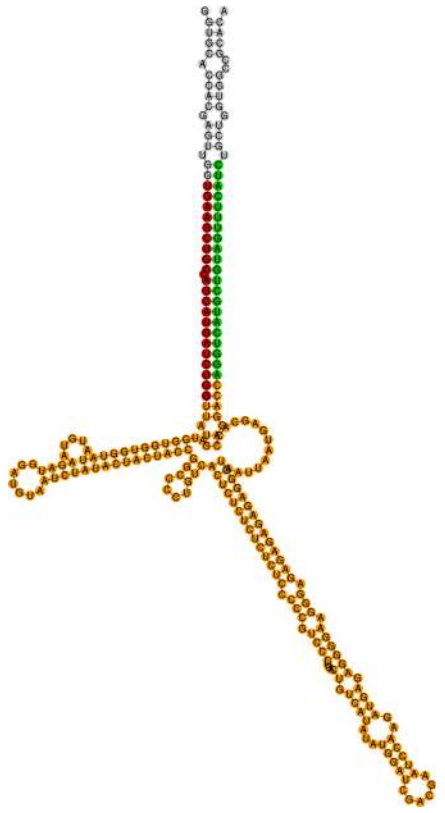

```
                 Mature                                                                                                                                                                                                       Star
GGUGCACCACGAGUUGGUGAAGCUGCCAGCAUGAUCUGGUUAUGAUGGUGGUGGUAUAUGUAAGAUGGAUGUAAUCUAUACUACUACCGGCCCCUGUCACUCUCUCUCUCUCCCCCGUCCCUGACUGUCAUAUAUGGAUCGACGAAUCCAAGAUGAGAGGGGAAGGGAGAGAGAGAGAGGGUAAUUAAUGAGCACCAGGACCAGGUCAUGCUGUAGUUUCAUCUGCUGGUGGCCGCACA 274815.7
.((((.((((.(((.((((((((((.((((((((((((((.((..((((((((((.....((((.......))))))))))))))))(((....)))((((((((((((((.(((.((((...((.((((...(((((......)))))..)))).)))))).))).))))))))))))))............)).)))))))))))))))))))))))).))).))))..)))).
.................UGAAGCUGCCAGCAUGAUCUGG........................................................................................................................................................................................................ 109430.519178
..........................................................................................................................................................................................................AGGUCAUGCUGUAGUUUCAUC................ 29902.593911
..............UGGUGAAGCUGCCAGCAUGAUC........................................................................................................................................................................................................... 174.03624
.................UGAAGCUGCCAGCAUGAUCUGGU....................................................................................................................................................................................................... 133.489927
.................UGAAGCUGCCAGCAUGAUCUG......................................................................................................................................................................................................... 87373.08142
.................UGAAGCUGCCAGCAUGAUCU.......................................................................................................................................................................................................... 10812.468169
.................UGAAGCUGCCAGCAUGAUC........................................................................................................................................................................................................... 7600.3824
.................UGCAGCUGCCAGCAUGAUCUGG........................................................................................................................................................................................................ 99.02676
.................UGAAGCUGCCAGCAUGCUCUGG........................................................................................................................................................................................................ 313.06315
.................UGAAGCUGCCAGCAUGAUCUGU........................................................................................................................................................................................................ 2498.179587
.................UGAAGCUGCCAGCAUGAUCUGC........................................................................................................................................................................................................ 1106.906571
.................UUAAGCUGCCAGCAUGAUCUGG........................................................................................................................................................................................................ 109.29366
.................UGAAGCUGCCGGCAUGAUCUGG........................................................................................................................................................................................................ 99.095138
.................UGAAGCUGCCAGCAUGAUUU.......................................................................................................................................................................................................... 303.296332
.................UGAAGCUGCCCGCAUGAUCUG......................................................................................................................................................................................................... 90.249355
.................UGAAGCUGCCAGCAUGGUCUGG........................................................................................................................................................................................................ 104.654672
.................UGAAGCUGCCAGCAUGGUCUG......................................................................................................................................................................................................... 93.724288
.................UGAGGCUGCCAGCAUGAUCUGG........................................................................................................................................................................................................ 90.006787
.................UGAAGCUGCCAUCAUGAUCUGG........................................................................................................................................................................................................ 190.091693
.................UGAAGCUGCCAGCAUGAUCUGUU....................................................................................................................................................................................................... 115.571651
.................UGAAGCUGCCAGCAUGAUCCG......................................................................................................................................................................................................... 377.87098
.................UGAAGCUUCCAGCAUGAUCUGG........................................................................................................................................................................................................ 404.560234
.................UGAAGCUGCCAGCAUGAUCGG......................................................................................................................................................................................................... 221.064558
.................UGAAGCUGCCAGCAUGAUCUGGA....................................................................................................................................................................................................... 704.197381
.................UGAAGAUGCCAGCAUGAUCUGG........................................................................................................................................................................................................ 106.80359
.................UGAAGCUGCCAGCAUGAUCUUG........................................................................................................................................................................................................ 102.16566
.................UGAAGCUGCCCGCAUGAUCUGG........................................................................................................................................................................................................ 170.41865
.................UGAAGCUGCUAGCAUGAUCUG......................................................................................................................................................................................................... 319.235784
.................UGAAGCUGCCAGCAUGAUCUU......................................................................................................................................................................................................... 1312.566292
.................UGAAGCUGCCAGCAUGCUCUG......................................................................................................................................................................................................... 170.22137
.................UGAAGCUGUCAGCAUGAUCUG......................................................................................................................................................................................................... 262.231569
.................UGAAGCUGCCAGCCUGAUC........................................................................................................................................................................................................... 268.02408
.................UGGAGCUGCCAGCAUGAUCUGG........................................................................................................................................................................................................ 91.253925
.................UGAAGCUGCCAGCAUGAUCC.......................................................................................................................................................................................................... 701.157019
.................UGAAGCUGCCAGCAUGAUCA.......................................................................................................................................................................................................... 410.926106
.................UGAAGCUGCCAGCAUGAUCG.......................................................................................................................................................................................................... 140.794267
.................CGAAGCUGCCAGCAUGAUCUGG........................................................................................................................................................................................................ 111.45279
.................UGAAGUUGCCAGCAUGAUCUGG........................................................................................................................................................................................................ 113.319655
.................UGAAGCUGCCAGCAUGACCUGG........................................................................................................................................................................................................ 107.245853
.................UGAAGCUGCCAGCCUGAUCUG......................................................................................................................................................................................................... 378.91246
.................UGAAGCUGCCAGCCUGAUCUGG........................................................................................................................................................................................................ 637.12086
.................UGAAGCUGCCAGCAUGAUCUC......................................................................................................................................................................................................... 366.816998
.................UGAAGCUCCCAGCAUGAUCUGG........................................................................................................................................................................................................ 179.36519
.................UGAAGCUGCUAGCAUGAUCUGG........................................................................................................................................................................................................ 437.520254
.................UGAAGCUGCCAGCAUGAUCCGG........................................................................................................................................................................................................ 104.250782
.................UGAAGCUGUCAGCAUGAUCUGG........................................................................................................................................................................................................ 517.480833
.................UGAAGCUGCCAGCAUGAUA........................................................................................................................................................................................................... 100.890336
.................UGAAGCUGCCAGCAUGAUU........................................................................................................................................................................................................... 496.456597
.................UGAAGCCGCCAGCAUGAUCUGG........................................................................................................................................................................................................ 120.680931
..................GAAGCUGCCAGCAUGAUCUG......................................................................................................................................................................................................... 118.106332
..................GAAGCUGCCAGCAUGAUCUGG........................................................................................................................................................................................................ 410.159089
...................AAGCUGCCAGCAUGAUCUGG........................................................................................................................................................................................................ 126.0603
.........................................................................................................................................................................................................CAGGUCAUGCUGUAGUUUCAUC................ 90.203784
..........................................................................................................................................................................................................AGGUCAUGCUGUAGUUUCAUCU............... 90.16923
..........................................................................................................................................................................................................AGGUCAUGCUGUAGUUUCAUA................ 182.182298
..........................................................................................................................................................................................................AGGUCAUGCUGUAGUUUCACC................ 100.59922
..........................................................................................................................................................................................................AGGUCAUGCUGUAGUUUCAUU................ 710.696847
.............................................................................................................................................................................................................UCAUGCUGUAGUUUCAUCUGC............. 8050.00258
```

zma-MIR167h

```
Chromosome:    chr6
Start:         95985872
End:           95986012
Strand:        -
Strand bias:   1.0
Abundance bias:0.997
MFE:           -86.70
```

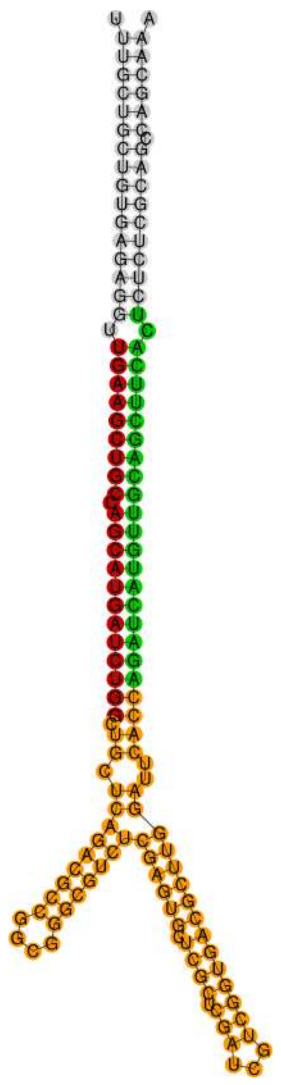

```
                          Mature                                                                          Star
UUUGCUGCUGUGAGAGGUUGAAGCUGCCAGCAUGAUCUGGCUGCUCAGACGCCGGCGGGCGUCUCGAGUGCUCGCUCGAUCGUCGGUGACGCUUGGAUUCACCAGAUCAUGUUGCAGCUUCACUCUCUCGCAGCCAGCAAA    286705.7
.((((((((((((((((.(((((((((.((((((((((((.((.(((((((((....)))))))((((((.((((.(((...)))))))))))))))..))))))))))))))))))))))).)))))))))).)))))).
..................UGAAGCUGCCAGCAUGAUCUGG.....................................................................................................    109430.519178
.......................................................................................................AGAUCAUGUUGCAGCUUCACU.................    41968.433459
.................UUGAAGCUGCCAGCAUGAUCUG......................................................................................................    4454.665034
.................UUGAAGCUGCCAGCAUGAUCU.......................................................................................................    545.365217
.................UUGAAGCUGCCAGCAUGAUCUGG.....................................................................................................    147.550835
.................UUGAAGCUGCCAGCAUGAUC........................................................................................................    301.685582
.................UUGAAGCUGCCAGCAUGAUCUA......................................................................................................    182.287853
.................UUGAAGCUGCCAGCAUGAUCUGA.....................................................................................................    56.60575
..................UGAAGCUGCCAGCAUGAUCUGGC....................................................................................................    51.16447
..................UGAAGCUGCCAGCAUGAUCUG......................................................................................................    87373.08142
..................UGAAGCUGCCAGCAUGAUCU.......................................................................................................    10812.468169
..................UGAAGCUGCCAGCAUGAUC........................................................................................................    7600.3824
..................UGAAGCUGCCAGCACGAUCUG......................................................................................................    57.250546
..................UGAAGCUGCCAGCAUUAUCUG......................................................................................................    54.428535
..................UGAAGCUGCCAGUAUGAUCUGG.....................................................................................................    65.271183
..................UGCAGCUGCCAGCAUGAUCUGG.....................................................................................................    99.02676
..................UGAAGCUGCCAGCCUGAUCU.......................................................................................................    56.97454
..................UGAAGCUGCCAGCAUGCUCUGG.....................................................................................................    313.06315
..................UGAAGCUGCCAGCAUGAUCUGU.....................................................................................................    2498.179587
..................UGAAGCUGCCAGCAUGAUCUGC.....................................................................................................    1106.906571
..................UUAAGCUGCCAGCAUGAUCUGG.....................................................................................................    109.29366
..................UGAAGCUGCCGGCAUGAUCUGG.....................................................................................................    99.095138
..................UGAAGCUGCCAGCAUGAUUU.......................................................................................................    303.296332
..................UGAAGCUGCCCGCAUGAUCUG......................................................................................................    90.249355
..................UGAAGCUGCCAGCAUAAUCUGG.....................................................................................................    47.278933
..................UGACGCUGCCAGCAUGAUCUGG.....................................................................................................    72.70245
..................UGAAGCUGCCAGCAUGGUCUGG.....................................................................................................    104.654672
..................UGAAGCUGCCAGCAUGGUCUG......................................................................................................    93.724288
..................UGAGGCUGCCAGCAUGAUCUGG.....................................................................................................    90.006787
..................UGAAGCUGACAGCAUGAUCUGG.....................................................................................................    75.207593
..................UGAAGCUGCCAUCAUGAUCUGG.....................................................................................................    190.091693
..................UGAAGCUGCCAGCAUGAUCCG......................................................................................................    377.87098
..................UGAAGCUUCCAGCAUGAUCUGG.....................................................................................................    404.560234
..................UGAAGCUGCCAGCAUGAUCGG......................................................................................................    221.064558
..................UGAAGCUACCAGCAUGAUCUGG.....................................................................................................    70.221167
..................UGAAGCUGCCAGCAUGAUCUGGA....................................................................................................    704.197381
..................UGAAGCGGCCAGCAUGAUCUGG.....................................................................................................    53.28603
..................UGAAGCUGCCAGCAUGAUCUGGG....................................................................................................    58.964747
..................UGAAGCUGCCAGCAUGAUUUGG.....................................................................................................    78.667039
..................UGAAGAUGCCAGCAUGAUCUGG.....................................................................................................    106.80359
..................UGAAGCUGCCAGCAUGAUCUUG.....................................................................................................    102.16566
..................UGAAGCUGCCAGCAUGACC........................................................................................................    89.30017
..................GGAAGCUGCCAGCAUGAUCUGG.....................................................................................................    51.763653
..................UGAAGCUGCCGGCAUGAUCUG......................................................................................................    70.24741
..................UGAAGCUGCCCGCAUGAUCUGG.....................................................................................................    170.41865
..................UGAAGCUGCUAGCAUGAUCUG......................................................................................................    319.235784
..................UGAAGCUGCCACCAUGAUCUGG.....................................................................................................    69.303513
..................UGAAGCUGCCAGCAUGAUCUU......................................................................................................    1312.566292
..................UGAAGCUGCCAGCAUGCUCUG......................................................................................................    170.22137
..................UGAAUCUGCCAGCAUGAUCUGG.....................................................................................................    61.140512
..................UGAAGCUGCCAGCGUGAUCUG......................................................................................................    57.90805
..................UGAAGCUGUCAGCAUGAUCUG......................................................................................................    262.231569
..................UGAAGCUGCCAUCAUGAUCUG......................................................................................................    84.182363
..................UGAAGCUGCCAGCAUGAUCUCG.....................................................................................................    59.21133
..................UGAAGCUGCCAGCCUGAUC........................................................................................................    268.02408
..................UGGAGCUGCCAGCAUGAUCUGG.....................................................................................................    91.253925
..................UGAAGCUGCAAGCAUGAUCUGG.....................................................................................................    85.25771
..................UGAAGCUGCCAGCAUGAUCC.......................................................................................................    701.157019
..................UGAAGCUGCCAGCAUGAUCA.......................................................................................................    410.926106
..................UGAAGCUGCCAGCAUGAUCG.......................................................................................................    140.794267
..................UGAAGCUGCAAGCAUGAUCUG......................................................................................................    66.905988
..................UGAAGCUGCCAGCAUGAUAUGG.....................................................................................................    81.60812
..................CGAAGCUGCCAGCAUGAUCUGG.....................................................................................................    111.45279
..................UGAAGCUGCCAGCAUGAGCUGG.....................................................................................................    61.13327
..................UGAAGUUGCCAGCAUGAUCUGG.....................................................................................................    113.319655
..................UGAAGCUGCCAGCAUGACCUGG.....................................................................................................    107.245853
..................UGAAGCUGCCAGCAUGAUCUGAC....................................................................................................    183.785896
..................UGAAGCUGCCAGCCUGAUCUG......................................................................................................    378.91246
..................UGAAGCUGCCAGCCUGAUCUGG.....................................................................................................    637.12086
..................UGAAGCUGCCAGCAUGAUAUG......................................................................................................    69.665306
..................UGAAGCUGCCAGCGUGAUCUGG.....................................................................................................    80.488142
..................UGAAGCUGCCAGCAUGACCUG......................................................................................................    82.41033
..................UGAAGCUGCCAGCAUGAUCUC......................................................................................................    366.816998
..................UGAAGCUCCCAGCAUGAUCUGG.....................................................................................................    179.36519
..................UGAAGCUGCUAGCAUGAUCUGG.....................................................................................................    437.520254
..................UGAAGCUGCCAGCAUGAUCCGG.....................................................................................................    104.250782
..................UGAAGCUGUCAGCAUGAUCUGG.....................................................................................................    517.480833
..................UGAAGCUGCCAGCACGAUCUGG.....................................................................................................    89.208769
..................UGAAGCUGCCAGCAUGAUA........................................................................................................    100.890336
..................UGAAGCUGCCAGCAUGAUU........................................................................................................    496.456597
..................UGAAGCUGCCAGCAUGAUCAG......................................................................................................    48.591311
..................UGAAGCCGCCAGCAUGAUCUGG.....................................................................................................    120.680931
...................GAAGCUGCCAGCAUGAUCUG......................................................................................................    118.106332
...................GAAGCUGCCAGCAUGAUCUGG.....................................................................................................    410.159089
....................AAGCUGCCAGCAUGAUCUGG.....................................................................................................    126.0603
......................................................................................................CAGAUCAUGUUGCAGCUUCAC..................    58.720194
.......................................................................................................AGAUCAUGUUGCAGCUUCAC..................    282.781388
.......................................................................................................AGAUCAUGUUGCAGCUUC....................    152.29094
.......................................................................................................AGAUCAUGUUGCAGCUUCA...................    141.429515
.......................................................................................................AGAUCAUGUUGCAGCUUCACG.................    217.328671
.......................................................................................................GGAUCAUGUUGCAGCUUCACU.................    49.93946
.......................................................................................................AGAUCAUGUUGCAGCUUCACA.................    532.174562
.......................................................................................................AGAUCAUGUUGCAGCUUCACC.................    1042.237012
.......................................................................................................AUAUCAUGUUGCAGCUUCACU.................    51.912046
........................................................................................................GAUCAUGUUGCAGCUUCACUC................    321.363106
........................................................................................................GAUCAUGUUGCAGCUUCACU.................    198.70222
.........................................................................................................AUCAUGUUGCAGCUUCACUC................    382.053296
.........................................................................................................AUCAUGUUGCAGCUUCACU.................    111.675919
```

zma-MIR167i

Chromosome: chr9
Start: 113476126
End: 113476252
Strand: +
Strand bias: 1.0
Abundance bias: 0.995
MFE: -77.10

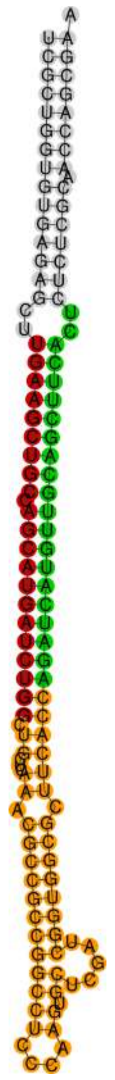

```
                 Mature                                                          Star
UCGCUGGUGUGAGAGCUUGAAGCUGCCAGCAUGAUCUGGCUGCUCAAACGCCGCCGGCCUCCCAAGUGCUCGAUCGGUGGCGCUUCACCAGAUCAUGUUGCAGCUUCACUCUCUCGCAACCAGCGAA 287305.
((((((((((((((..(((((((((.(((((((((((.((...((.((((((((((((....)).)).....)))))))).))))))))))))))))))))))))..))))))).)))))))).
.................UGAAGCUGCCAGCAUGAUCUGG........................................................................................ 109430.519178
.........................................................................................AGAUCAUGUUGCAGCUUCACU................. 41968.433459
................UUGAAGCUGCCAGCAUGAUCUG......................................................................................... 4454.665034
................UUGAAGCUGCCAGCAUGAUCU.......................................................................................... 545.365217
................UUGAAGCUGCCAGCAUGAUCUGG........................................................................................ 147.550835
................UUGAAGCUGCCAGCAUGAUC........................................................................................... 301.685582
................UUGAAGCUGCCAGCAUGAUCUA......................................................................................... 182.287853
................UUGAAGCUGCCAGCAUGAUCUGA........................................................................................ 56.60575
.................UGAAGCUGCCAGCAUGAUCUGGC....................................................................................... 51.16447
.................UGAAGCUGCCAGCAUGAUCUG......................................................................................... 87373.08142
.................UGAAGCUGCCAGCAUGAUCU.......................................................................................... 10812.468169
.................UGAAGCUGCCAGCAUGAUC........................................................................................... 7600.3824
.................UGAAGCUGCCAGCACGAUCUG......................................................................................... 57.250546
.................UGAAGCUGCCAGCAUUAUCUG......................................................................................... 54.428535
.................UGAAGCUGCCAGUAUGAUCUGG........................................................................................ 65.271183
.................UGCAGCUGCCAGCAUGAUCUGG........................................................................................ 99.02676
.................UGAAGCUGCCAGCCUGAUCU.......................................................................................... 56.97454
.................UGAAGCUGCCAGCAUGCUCUGG........................................................................................ 313.06315
.................UGAAGCUGCCAGCAUGAUCUGU........................................................................................ 2498.179587
.................UGAAGCUGCCAGCAUGAUCUGC........................................................................................ 1106.906571
.................UUAAGCUGCCAGCAUGAUCUGG........................................................................................ 109.29366
.................UGAAGCUGCCGGCAUGAUCUGG........................................................................................ 99.095138
.................UGAAGCUGCCAGCAUGAUUU.......................................................................................... 303.296332
.................UGAAGCUGCCCGCAUGAUCUG......................................................................................... 90.249355
.................UGAAGCUGCCAGCAUAAUCUGG........................................................................................ 47.278933
.................UGACGCUGCCAGCAUGAUCUGG........................................................................................ 72.70245
.................UGAAGCUGCCAGCAUGGUCUGG........................................................................................ 104.654672
.................UGAAGCUGCCAGCAUGGUCUG......................................................................................... 93.724288
.................UGAGGCUGCCAGCAUGAUCUGG........................................................................................ 90.006787
.................UGAAGCUGACAGCAUGAUCUGG........................................................................................ 75.207593
.................UGAAGCUGCCAUCAUGAUCUGG........................................................................................ 190.091693
.................UGAAGCUGCCAGCAUGAUCCG......................................................................................... 377.87098
.................UGAAGCUUCCAGCAUGAUCUGG........................................................................................ 404.560234
.................UGAAGCUGCCAGCAUGAUCGG......................................................................................... 221.064558
.................UGAAGCUACCAGCAUGAUCUGG........................................................................................ 70.221167
.................UGAAGCUGCCAGCAUGAUCUGGA....................................................................................... 704.197381
.................UGAAGCGGCCAGCAUGAUCUGG........................................................................................ 53.28603
.................UGAAGCUGCCAGCAUGAUCUGGG....................................................................................... 58.964747
.................UGAAGCUGCCAGCAUGAUUUGG........................................................................................ 78.667039
.................UGAAGAUGCCAGCAUGAUCUGG........................................................................................ 106.80359
.................UGAAGCUGCCAGCAUGAUCUUG........................................................................................ 102.16566
.................UGAAGCUGCCAGCAUGACC........................................................................................... 89.30017
.................GGAAGCUGCCAGCAUGAUCUGG........................................................................................ 51.763653
.................UGAAGCUGCCGGCAUGAUCUG......................................................................................... 70.24741
.................UGAAGCUGCCCGCAUGAUCUGG........................................................................................ 170.41865
.................UGAAGCUGCUAGCAUGAUCUG......................................................................................... 319.235784
.................UGAAGCUGCCACCAUGAUCUGG........................................................................................ 69.303513
.................UGAAGCUGCCAGCAUGAUCUU......................................................................................... 1312.566292
.................UGAAGCUGCCAGCAUGCUCUG......................................................................................... 170.22137
.................UGAAUCUGCCAGCAUGAUCUGG........................................................................................ 61.140512
.................UGAAGCUGCCAGCGUGAUCUG......................................................................................... 57.90805
.................UGAAGCUGUCAGCAUGAUCUG......................................................................................... 262.231569
.................UGAAGCUGCCAUCAUGAUCUG......................................................................................... 84.182363
.................UGAAGCUGCCAGCAUGAUCUCG........................................................................................ 59.21133
.................UGAAGCUGCCAGCCUGAUC........................................................................................... 268.02408
.................UGGAGCUGCCAGCAUGAUCUGG........................................................................................ 91.253925
.................UGAAGCUGCAAGCAUGAUCUGG........................................................................................ 85.25771
.................UGAAGCUGCCAGCAUGAUCC.......................................................................................... 701.157019
.................UGAAGCUGCCAGCAUGAUCA.......................................................................................... 410.926106
.................UGAAGCUGCCAGCAUGAUCG.......................................................................................... 140.794267
.................UGAAGCUGCAAGCAUGAUCUG......................................................................................... 66.905988
.................UGAAGCUGCCAGCAUGAUAUGG........................................................................................ 81.60812
.................CGAAGCUGCCAGCAUGAUCUGG........................................................................................ 111.45279
.................UGAAGCUGCCAGCAUGAGCUGG........................................................................................ 61.13327
.................UGAAGUUGCCAGCAUGAUCUGG........................................................................................ 113.319655
.................UGAAGCUGCCAGCAUGACCUGG........................................................................................ 107.245853
.................UGAAGCUGCCAGCAUGAUCUGAC....................................................................................... 183.785896
.................UGAAGCUGCCAGCCUGAUCUG......................................................................................... 378.91246
.................UGAAGCUGCCAGCCUGAUCUGG........................................................................................ 637.12086
.................UGAAGCUGCCAGCAUGAUAUG......................................................................................... 69.665306
.................UGAAGCUGCCAGCGUGAUCUGG........................................................................................ 80.488142
.................UGAAGCUGCCAGCAUGACCUG......................................................................................... 82.41033
.................UGAAGCUGCCAGCAUGAUCUC......................................................................................... 366.816998
.................UGAAGCUCCCAGCAUGAUCUGG........................................................................................ 179.36519
.................UGAAGCUGCUAGCAUGAUCUGG........................................................................................ 437.520254
.................UGAAGCUGCCAGCAUGAUCCGG........................................................................................ 104.250782
.................UGAAGCUGUCAGCAUGAUCUGG........................................................................................ 517.480833
.................UGAAGCUGCCAGCACGAUCUGG........................................................................................ 89.208769
.................UGAAGCUGCCAGCAUGAUA........................................................................................... 100.890336
.................UGAAGCUGCCAGCAUGAUU........................................................................................... 496.456597
.................UGAAGCUGCCAGCAUGAUCAG......................................................................................... 48.591311
.................UGAAGCCGCCAGCAUGAUCUGG........................................................................................ 120.680931
..................GAAGCUGCCAGCAUGAUCUG......................................................................................... 118.106332
..................GAAGCUGCCAGCAUGAUCUGG........................................................................................ 410.159089
...................AAGCUGCCAGCAUGAUCUGG........................................................................................ 126.0603
........................................................................................CAGAUCAUGUUGCAGCUUCAC.................. 58.720194
.........................................................................................AGAUCAUGUUGCAGCUUCAC.................. 282.781388
.........................................................................................AGAUCAUGUUGCAGCUUC.................... 152.29094
.........................................................................................AGAUCAUGUUGCAGCUUCA................... 141.429515
.........................................................................................AGAUCAUGUUGCAGCUUCACG................. 217.328671
.........................................................................................GGAUCAUGUUGCAGCUUCACU................. 49.93946
.........................................................................................AGAUCAUGUUGCAGCUUCACA................. 532.174562
.........................................................................................AGAUCAUGUUGCAGCUUCACC................. 1042.237012
.........................................................................................AUAUCAUGUUGCAGCUUCACU................. 51.912046
..........................................................................................GAUCAUGUUGCAGCUUCACUC................ 321.363106
..........................................................................................GAUCAUGUUGCAGCUUCACU................. 198.70222
...........................................................................................AUCAUGUUGCAGCUUCACUC................ 382.053296
...........................................................................................AUCAUGUUGCAGCUUCACU................. 111.675919
```

zma-MIR167j

Chromosome: chr1
Start: 12791783
End: 12791923
Strand: +
Strand bias: 1.0
Abundance bias: 0.997
MFE: -67.20

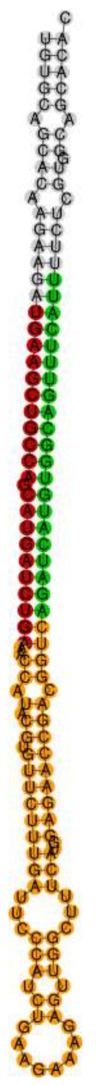

```
                 Mature                                                                                     Star
UGUGCAGCACAAGAAGAUGAAGCUGCCAGCAUGAUCUGAAACCAUACGUGUUCUUUGAUUCCCAUCUGAAGAAAGAGUUGGCUUUCAUGGAGAACCGACGGUCAGAUCAUGUGGCAGUUUCAUUUUCUCGUGGCAGCACAC    321402.7
(((((.((((.((((((((((((((((.(((((((((..((.(.((.(((((((((...(((.((........)).)))...)))..))))))))).))))))))))))))))))))))))))).)).)).))))).
.................UGAAGCUGCCAGCAUGAUCUGA......................................................................................................    190321.638405
.......................................................................................................AGAUCAUGUGGCAGUUUCAUU.................    2603.166394
................AUGAAGCUGCCAGCAUGAUCUGG......................................................................................................    156.389146
................AUGAAGCUGCCAGCAUGAUCUA.......................................................................................................    44.47113
.................UGAAGCUGCCAGCAUGAUCUG.......................................................................................................    87373.08142
.................UGAAGCUGCCAGCAUGAUCU........................................................................................................    10812.468169
.................UGAAGCUGCCAGCAUGAUCUGAA.....................................................................................................    350.265363
.................UGAAGCUGCCAGCAUGAUCUGAAA....................................................................................................    254.401463
.................UGAAGCUGCCAGCAUGAUC.........................................................................................................    7600.3824
.................UGAAGCUGCCAGCACGAUCUG.......................................................................................................    57.250546
.................UGAAGCUGCCAGCAUUAUCUG.......................................................................................................    54.428535
.................UGCAGCUGCCAGCAUGAUCUGA......................................................................................................    191.23146
.................UGAAGCUGCCAGCCUGAUCU........................................................................................................    56.97454
.................UGAAGCUGCCAGUAUGAUCUG.......................................................................................................    46.20878
.................UGAAGCUGCCUGCAUGAUCUGA......................................................................................................    65.733528
.................UGAAGCUGCCAGCAUGCUCUGA......................................................................................................    329.205711
.................UGAAGCUCCCAGCAUGAUCUGA......................................................................................................    72.558829
.................UUAAGCUGCCAGCAUGAUCUGA......................................................................................................    139.981775
.................UGAAGCUGCCAGCAUUAUCUGA......................................................................................................    106.403597
.................UGAAGCUGCUAGCAUGAUCUGA......................................................................................................    217.573975
.................UAAAGCUGCCAGCAUGAUCUGA......................................................................................................    101.522412
.................UGAAGCUGCCAGCAUGAUCUGU......................................................................................................    2498.179587
.................UGAAGCUGCCAGCAUGAUCUGC......................................................................................................    1106.906571
.................UGAAGCUGCCAGCAUGAUUU........................................................................................................    303.296332
.................UGAAGCUGCCCGCAUGAUCUG.......................................................................................................    90.249355
.................UGAAGCUGACAGCAUGAUCUGA......................................................................................................    103.380295
.................UGACGCUGCCAGCAUGAUCUGA......................................................................................................    90.181403
.................UGAACCUGCCAGCAUGAUCUGA......................................................................................................    59.794643
.................UGAAGCUGCCAGCAUGGUCUG.......................................................................................................    93.724288
.................UGAAGCUGCCAGCAUGGUCUGA......................................................................................................    281.801836
.................UGAAGCUGCCAUCAUGAUCUGA......................................................................................................    81.12687
.................UGAAGCUGCCAGCAUGAUCCG.......................................................................................................    377.87098
.................UGAAGCUUCCAGCAUGAUCUGA......................................................................................................    121.400555
.................UGUAGCUGCCAGCAUGAUCUGA......................................................................................................    40.438589
.................UGAAACUGCCAGCAUGAUCUGA......................................................................................................    55.567834
.................UGAAGCUGCCAGCAUGAUCGG.......................................................................................................    221.064558
.................UGAAGCUACCAGCAUGAUCUGA......................................................................................................    93.822657
.................UGAAGCUGCCAGCAUGAUCUGGA.....................................................................................................    704.197381
.................UGAAGCUGCCAGAAUGAUCUGA......................................................................................................    50.958286
.................UGAAGCUGCCAGCAUGAUUUGA......................................................................................................    149.630725
.................UGAAGCUGCCAGCAUGUUCUGA......................................................................................................    40.698538
.................UGAAGAUGCCAGCAUGAUCUGA......................................................................................................    85.782049
.................UGAAGCUGCCAGCAUGAUCUUA......................................................................................................    84.496084
.................UGAAGCUGCCAGCAUGACC.........................................................................................................    89.30017
.................GGAAGCUGCCAGCAUGAUCUGA......................................................................................................    68.19713
.................UGAAGCUGACAGCAUGAUCUG.......................................................................................................    42.117292
.................UGAAGCUGCCAGCAUAAUCUGA......................................................................................................    93.573188
.................UGAAGCUGCCGGCAUGAUCUG.......................................................................................................    70.24741
.................UGAAGCUGCCCGCAUGAUCUGA......................................................................................................    180.57913
.................UGAAGCUGCUAGCAUGAUCUG.......................................................................................................    319.235784
.................UGAAGCUGCCAGCAUGAUCUU.......................................................................................................    1312.566292
.................UGAAGCUGUCAGCAUGAUCUGA......................................................................................................    222.347364
.................UGAAGCUGCCAACAUGAUCUGA......................................................................................................    69.907627
.................UGAAGCUGCCAGCACGAUCUGA......................................................................................................    192.4512
.................UGAAGCUGCCAGCAUGCUCUG.......................................................................................................    170.22137
.................UGAAGCUGCCAGCAUGAUCUCA......................................................................................................    51.863964
.................UGAAUCUGCCAGCAUGAUCUGA......................................................................................................    166.209286
.................UGAAGCUGCCAGCGUGAUCUG.......................................................................................................    57.90805
.................UGAAGCUGCCGGCAUGAUCUGA......................................................................................................    249.39207
.................UGAAGCAGCCAGCAUGAUCUGA......................................................................................................    56.747034
.................UGAAGCUGUCAGCAUGAUCUG.......................................................................................................    262.231569
.................UGAAGCUGCCAUCAUGAUCUG.......................................................................................................    84.182363
.................UGAAGCUGCCAGCCUGAUC.........................................................................................................    268.02408
.................CGAAGCUGCCAGCAUGAUCUGA......................................................................................................    261.08718
.................UGGAGCUGCCAGCAUGAUCUGA......................................................................................................    261.262175
.................UGAAGCUGCAAGCAUGAUCUGA......................................................................................................    146.964335
.................UGAAGCUGCCAGCAUGAUCUGCA.....................................................................................................    42.30903
.................UGAGGCUGCCAGCAUGAUCUGA......................................................................................................    285.387524
.................UGAAGCUGCCAGCAUGAUCC........................................................................................................    701.157019
.................UGAAGCUGCCAGCAUGAUCA........................................................................................................    410.926106
.................UGAAGCUGCCAGCAUGAUCG........................................................................................................    140.794267
.................UGAAGCUGCAAGCAUGAUCUG.......................................................................................................    66.905988
.................UGAAGCUGCCAGCAUGAUAUGA......................................................................................................    194.530758
.................UGAAGUUGCCAGCAUGAUCUGA......................................................................................................    179.483606
.................UGAAGCUGCCAGCAUGACCUGA......................................................................................................    193.93929
.................UGAAGCUGCCAGCAUGAUCUGAC.....................................................................................................    183.785896
.................UGAAGCUGCCAGCCUGAUCUG.......................................................................................................    378.91246
.................UGAAGCUGCCAGUAUGAUCUGA......................................................................................................    130.096887
.................UGAAGCUGCCAGCCUGAUCUGA......................................................................................................    926.975749
.................UGAAGCUGCCAGCAUGAUAUG.......................................................................................................    69.665306
.................UGAAGCUGCCAGCGUGAUCUGA......................................................................................................    214.150885
.................UGAAGCUGCCAGCAUGAUCGGA......................................................................................................    121.190122
.................UGAAGCUGCCAGCAUGACCUG.......................................................................................................    82.41033
.................UGAAGCUGCCAGCAUGAUCUC.......................................................................................................    366.816998
.................UGAAGCUGCCAGCAUGAUCAGA......................................................................................................    54.066961
.................AGAAGCUGCCAGCAUGAUCUGA......................................................................................................    76.736765
.................UGAAGCUGCCAGCAUGAUCCGA......................................................................................................    209.23258
.................UGAAGCCGCCAGCAUGAUCUGA......................................................................................................    245.45403
.................UGAAGCGGCCAGCAUGAUCUGA......................................................................................................    48.117644
.................UGAAGCUGCCAGCAUGAUA.........................................................................................................    100.890336
.................UGAAGCUGCCAGCAUGAUU.........................................................................................................    496.456597
.................UGAAGCUGCCAGCAUGAUCAG.......................................................................................................    48.591311
.................UGAAGCUGCCAGCAUGAUGUGA......................................................................................................    104.729463
..................GAAGCUGCCAGCAUGAUCUG.......................................................................................................    118.106332
..................GAAGCUGCCAGCAUGAUCUGA......................................................................................................    310.842898
...................AAGCUGCCAGCAUGAUCUGA......................................................................................................    586.619841
....................................................................................................GUCAGAUCAUGUGGCAGUUUCAUU.................    58.682219
.......................................................................................................AGAUCAUGUGGCAGUUUCAUC.................    158.56424
```

zma-MIR168a

Chromosome: chr5
Start: 76534928
End: 76535030
Strand: -
Strand bias: 1.0
Abundance bias: 0.999
MFE: -64.00

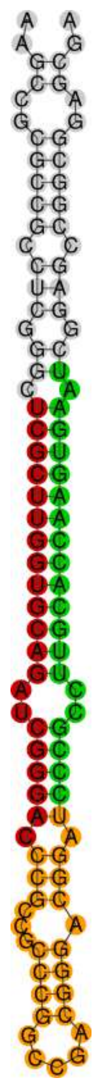

```
                    Mature                                     Star
AAGCCGCGCCGCCUCGGGCUCGCUUGGUGCAGAUCGGGACCCGCCGCCCGGCCGACGGGACGGAUCCCGCCUUGCACCAAGUGAAUCGGAGCCGGCGGAGCGA    2841528.2
..((..(((((.(((.((.((((((((((((..(((((.(((...((((.....)))).))).)))))..)))))))))))).)).))).)))))..))..
...................UCGCUUGGUGCAGAUCGGGAC...............................................................    2401712.7451
.................................................................CCCGCCUUGCACCAAGUGAAU.................    151119.87152
...................UCGCUUGGUGCAGAUCGGGACC..............................................................    22341.52625
...................UCGCUUGGUGCAGAUCGGGACCC.............................................................    5958.485524
...................UCGCUUGGUGCAGAUCGGGA................................................................    37880.68826
...................UCGCUUGGUGCAGAUCGGGACCCG............................................................    56082.98012
...................UCGCUUGGUGCAGAUCGG..................................................................    847.907522
...................UCGCUUGGUGCAGAUCGGG.................................................................    2224.831679
...................UCGGUUGGUGCAGAUCGGGAC...............................................................    356.414809
...................UCGCUAGGUGCAGAUCGGGAC...............................................................    538.691174
...................UCGCUUGGUGCAGGUCGGGAC...............................................................    2558.52117
...................UCGCUUGGUACAGAUCGGGAC...............................................................    1310.784953
...................UCGCUUGGAGCAGAUCGGGAC...............................................................    936.930397
...................UCGCUUGGUGCAGAUCGGGUC...............................................................    770.23264
...................UCGCUUGGUGCGGAUCGGGAC...............................................................    4008.375462
...................UCGCUGGGUGCAGAUCGGGAC...............................................................    245.224559
...................UCGCUUGAUGCAGAUCGGGAC...............................................................    1180.749754
...................UCGCUUGGUGCAGAUCGGGACA..............................................................    8213.867196
...................UCGCUUGGUGCAGAUCGGGACG..............................................................    239.515315
...................UCGCUUGGUGCAGAUCGGGACU..............................................................    6182.32771
...................UCGCUUGGUGCAGCUCGGGAC...............................................................    2158.384017
...................UCGCUUGGUGCAGAUCGGGAG...............................................................    390.695034
...................UCGCUUGGUGCAGAUCGGGAU...............................................................    12717.414882
...................UCGCUUGGUGGAGAUCGGGAC...............................................................    293.985011
...................UCGCCUGGUGCAGAUCGGGAC...............................................................    1729.13194
...................UAGCUUGGUGCAGAUCGGGAC...............................................................    752.84319
...................UUGCUUGGUGCAGAUCGGGAC...............................................................    3282.852644
...................CCGCUUGGUGCAGAUCGGGAC...............................................................    3211.702988
...................UCGCUUGGUGCAGAUCGAGAC...............................................................    1190.860723
...................UCGCUUUGUGCAGAUCGGGAC...............................................................    1397.32773
...................UCGCUUGGUGCAGAUCCGGAC...............................................................    982.402492
...................UCGCUUAGUGCAGAUCGGGAC...............................................................    1450.194343
...................UCGCAUGGUGCAGAUCGGGAC...............................................................    258.797276
...................UCGCUUGGUGCAGACCGGGAC...............................................................    1822.289898
...................UCGCUUGUUGCAGAUCGGGAC...............................................................    1637.325137
...................UCGCUUGGUGCAGAUCGUGA................................................................    179.677731
...................UCGCUUGGUGCCGAUCGGGAC...............................................................    1418.015747
...................UCGCUUGGUGCAGAUUGGGAC...............................................................    2012.10534
...................UCGCUUGGUGAAGAUCGGGAC...............................................................    1099.52664
...................UCGCUUGGUGCAGAUCGCGAC...............................................................    676.827982
...................UGGCUUGGUGCAGAUCGGGAC...............................................................    387.052164
...................UCGCUUGGUGCACAUCGGGAC...............................................................    417.963373
...................UCGCUUGGUGCAGAUCGGGGC...............................................................    3848.556647
...................UCGCUUGGUGCAGAUCGGUAC...............................................................    832.58667
...................UCGCUUGGUGCAGAUAGGGAC...............................................................    1537.994902
...................UCGCUUGGUGCAGAUCGGCAC...............................................................    411.609446
...................UCUCUUGGUGCAGAUCGGGAC...............................................................    3099.624262
...................UCCCUUGGUGCAGAUCGGGAC...............................................................    831.083522
...................UCGCUUGGUGCAGAUCGGGAA...............................................................    14881.02407
...................UCGCGUGGUGCAGAUCGGGAC...............................................................    266.061397
...................UCGAUUGGUGCAGAUCGGGAC...............................................................    980.793299
...................UCGCUUGGUGUAGAUCGGGAC...............................................................    1858.352963
...................UCGCUUGGUGCAGAUCAGGAC...............................................................    2517.647609
...................UCGCUCGGUGCAGAUCGGGAC...............................................................    2412.219152
...................UCGCUUGGUCCAGAUCGGGAC...............................................................    790.929837
...................UCGCUUGGUGCAGAUGGGGAC...............................................................    250.519193
...................UCGCUUGGUGCUGAUCGGGAC...............................................................    619.427055
...................UCGCUUGGUGCAGAACGGGAC...............................................................    348.195242
...................UCGCUUGGGGCAGAUCGGGAC...............................................................    1565.807622
...................UCGCUUGGUGCAGAUCGGGCC...............................................................    5555.977925
...................UCGCUUGCUGCAGAUCGGGAC...............................................................    556.768504
...................UCGCUUGGUGCAUAUCGGGAC...............................................................    1591.951032
...................UCGCUUGGUGCAGAUCGGAAC...............................................................    690.420174
...................UCGCUUGGCGCAGAUCGGGAC...............................................................    4048.488426
...................UCGCUUGGUGCAGAUCUGGAC...............................................................    2621.093434
...................UCGUUUGGUGCAGAUCGGGAC...............................................................    2383.987552
...................UCACUUGGUGCAGAUCGGGAC...............................................................    1978.264072
...................UCGCUUGGUGCAAAUCGGGAC...............................................................    730.410712
...................ACGCUUGGUGCAGAUCGGGAC...............................................................    1361.755367
...................GCGCUUGGUGCAGAUCGGGAC...............................................................    872.869206
...................UCGCUUGGUGCAGAUCGUGAC...............................................................    2087.811401
...................UCGCUUGGUUCAGAUCGGGAC...............................................................    1246.525634
...................UCGCUUGGUGCAGAGCGGGAC...............................................................    294.894498
...................UCGCUUCGUGCAGAUCGGGAC...............................................................    1163.394725
...................UCGCUUGGUGCAGUUCGGGAC...............................................................    434.036843
....................CGCUUGGUGCAGAUCGGGAC...............................................................    3282.524645
....................CGCUUGGUGCAGAUCGGGACCCG............................................................    320.481526
....................CGCUUGGUGCAGAUCGGGA................................................................    324.469661
.....................GCUUGGUGCAGAUCGGGACC..............................................................    588.870868
.....................GCUUGGUGCAGAUCGGGACCC.............................................................    2439.801214
.....................GCUUGGUGCAGAUCGGGAC...............................................................    2458.957538
......................CUUGGUGCAGAUCGGGAC...............................................................    155.410411
......................CUUGGUGCAGAUCGGGACCCG............................................................    411.597374
..............................................................GAUCCCGCCUUGCACCAAGUGAAU.................    1931.10781
...............................................................AUCCCGCCUUGCACCAAGUGA...................    2355.998477
.................................................................CCCGCCUUGCACCAAGUGAA..................    5112.755647
.................................................................CCCGCCUUGCACCAAGUGA...................    985.910526
.................................................................CCCGCCUUGCACCAAUUGAAU.................    157.757693
.................................................................CUCGCCUUGCACCAAGUGAAU.................    205.643349
.................................................................CCCUCCUUGCACCAAGUGAAU.................    215.213522
.................................................................CCCGCCUUGCACCAAGUGAAUA................    303.781397
.................................................................CCCGCCUUGCACCAAGUGAAUU................    810.965582
.................................................................CCCGCCUUGCACCAAGUGGAU.................    159.78022
.................................................................CCCGCCUUGCGCCAAGUGAAU.................    160.93791
.................................................................CCCGCCUUGCACCAAGUGAAC.................    452.224378
.................................................................CCCGCCUUGCACCAAGUGAAA.................    402.971836
..................................................................CCGCCUUGCACCAAGUGAAU.................    2382.393194
...................................................................CGCCUUGCACCAAGUGAAU.................    311.114292
```

zma-MIR168b

```
Chromosome:    chr4
Start:         244520636
End:           244520738
Strand:        -
Strand bias:   1.0
Abundance bias:1.0
MFE:           -66.40
```

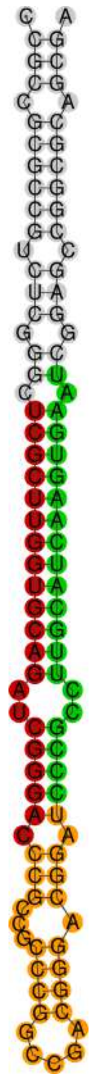

```
                                         Mature                                              Star
CCGCCGCGCCGUCUCGGGCUCGCUUGGUGCAGAUCGGGACCCGCCGCCCGGCCGACGGGACGGAUCCCGCCUUGCAUCAAGUGAAUCGGAGCCGGCGCAGCGA    2778765.4
.(((.((((((.(((.((.(((((((((((((..(((((.(((...((((.....)))).))).)))))..))))))))))))).)).))).)))))).))).
...................UCGCUUGGUGCAGAUCGGGAC...............................................................    2401712.7451
.................................................................CCCGCCUUGCAUCAAGUGAAU.................    95260.731315
...................UCGCUUGGUGCAGAUCGGGACC..............................................................    22341.52625
...................UCGCUUGGUGCAGAUCGGGACCC.............................................................    5958.485524
...................UCGCUUGGUGCAGAUCGGGA................................................................    37880.68826
...................UCGCUUGGUGCAGAUCGGGACCCG............................................................    56082.98012
...................UCGCUUGGUGCAGAUCGG..................................................................    847.907522
...................UCGCUUGGUGCAGAUCGGG.................................................................    2224.831679
...................UCGGUUGGUGCAGAUCGGGAC...............................................................    356.414809
...................UCGCUAGGUGCAGAUCGGGAC...............................................................    538.691174
...................UCGCUUGGUGCAGGUCGGGAC...............................................................    2558.52117
...................UCGCUUGGUACAGAUCGGGAC...............................................................    1310.784953
...................UCGCUUGGAGCAGAUCGGGAC...............................................................    936.930397
...................UCGCUUGGUGCAGAUCGGGUC...............................................................    770.23264
...................UCGCUUGGUGCGGAUCGGGAC...............................................................    4008.375462
...................UCGCUGGGUGCAGAUCGGGAC...............................................................    245.224559
...................UCGCUUGAUGCAGAUCGGGAC...............................................................    1180.749754
...................UCGCUUGGUGCAGAUCGGGACA..............................................................    8213.867196
...................UCGCUUGGUGCAGAUCGGGACG..............................................................    239.515315
...................UCGCUUGGUGCAGAUCGGGACU..............................................................    6182.32771
...................UCGCUUGGUGCAGCUCGGGAC...............................................................    2158.384017
...................UCGCUUGGUGCAGAUCGGGAG...............................................................    390.695034
...................UCGCUUGGUGCAGAUCGGGAU...............................................................    12717.414882
...................UCGCUUGGUGGAGAUCGGGAC...............................................................    293.985011
...................UCGCCUGGUGCAGAUCGGGAC...............................................................    1729.13194
...................UAGCUUGGUGCAGAUCGGGAC...............................................................    752.84319
...................UUGCUUGGUGCAGAUCGGGAC...............................................................    3282.852644
...................CCGCUUGGUGCAGAUCGGGAC...............................................................    3211.702988
...................UCGCUUGGUGCAGAUCGAGAC...............................................................    1190.860723
...................UCGCUUUGUGCAGAUCGGGAC...............................................................    1397.32773
...................UCGCUUGGUGCAGAUCCGGAC...............................................................    982.402492
...................UCGCUUGGUGCAGAUCGGGG................................................................    129.962002
...................UCGCUUAGUGCAGAUCGGGAC...............................................................    1450.194343
...................UCGCAUGGUGCAGAUCGGGAC...............................................................    258.797276
...................UCGCUUGGUGCAGACCGGGAC...............................................................    1822.289898
...................UCGCUUGUUGCAGAUCGGGAC...............................................................    1637.325137
...................UCGCUUGGUGCAGAUCGUGA................................................................    179.677731
...................UCGCUUGGUGCCGAUCGGGAC...............................................................    1418.015747
...................UCGCUUGGUGCAGAUUGGGAC...............................................................    2012.10534
...................UCGCUUGGUGAAGAUCGGGAC...............................................................    1099.52664
...................UCGCUUGGUGCAGAUCGCGAC...............................................................    676.827982
...................UGGCUUGGUGCAGAUCGGGAC...............................................................    387.052164
...................UCGCUUGGUGCACAUCGGGAC...............................................................    417.963373
...................UCGCUUGGUGCAGAUCGGGGC...............................................................    3848.556647
...................UCGCUUGGUGCAGAUCGGUAC...............................................................    832.58667
...................UCGCUUGGUGCAGAUAGGGAC...............................................................    1537.994902
...................UCGCUUGGUGCAGAUCGGCAC...............................................................    411.609446
...................UCUCUUGGUGCAGAUCGGGAC...............................................................    3099.624262
...................UCCCUUGGUGCAGAUCGGGAC...............................................................    831.083522
...................UCGCUUGGUGCAGAUCGGGAA...............................................................    14881.02407
...................UCGCGUGGUGCAGAUCGGGAC...............................................................    266.061397
...................UCGAUUGGUGCAGAUCGGGAC...............................................................    980.793299
...................UCGCUUGGUGUAGAUCGGGAC...............................................................    1858.352963
...................UCGCUUGGUGCAGAUCAGGAC...............................................................    2517.647609
...................UCGCUCGGUGCAGAUCGGGAC...............................................................    2412.219152
...................UCGCUUGGUCCAGAUCGGGAC...............................................................    790.929837
...................UCGCUUGGUGCAGAUGGGGAC...............................................................    250.519193
...................UCGCUUGGUGCUGAUCGGGAC...............................................................    619.427055
...................UCGCUUGGUGCAGAACGGGAC...............................................................    348.195242
...................UCGCUUGGGGCAGAUCGGGAC...............................................................    1565.807622
...................UCGCUUGGUGCAGAUCGGGCC...............................................................    5555.977925
...................UCGCUUGCUGCAGAUCGGGAC...............................................................    556.768504
...................UCGCUUGGUGCAUAUCGGGAC...............................................................    1591.951032
...................UCGCUUGGUGCAGAUCGGAAC...............................................................    690.420174
...................UCGCUUGGCGCAGAUCGGGAC...............................................................    4048.488426
...................UCGCUUGGUGCAGAUCUGGAC...............................................................    2621.093434
...................UCGUUUGGUGCAGAUCGGGAC...............................................................    2383.987552
...................UCACUUGGUGCAGAUCGGGAC...............................................................    1978.264072
...................UCGCUUGGUGCAAAUCGGGAC...............................................................    730.410712
...................ACGCUUGGUGCAGAUCGGGAC...............................................................    1361.755367
...................GCGCUUGGUGCAGAUCGGGAC...............................................................    872.869206
...................UCGCUUGGUGCAGAUCGUGAC...............................................................    2087.811401
...................UCGCUUGGUUCAGAUCGGGAC...............................................................    1246.525634
...................UCGCUUGGUGCAGAGCGGGAC...............................................................    294.894498
...................UCGCUUCGUGCAGAUCGGGAC...............................................................    1163.394725
...................UCGCUUGGUGCAGUUCGGGAC...............................................................    434.036843
....................CGCUUGGUGCAGAUCGGGAC...............................................................    3282.524645
....................CGCUUGGUGCAGAUCGGGACCCG............................................................    320.481526
....................CGCUUGGUGCAGAUCGGGA................................................................    324.469661
.....................GCUUGGUGCAGAUCGGGACC..............................................................    588.870868
.....................GCUUGGUGCAGAUCGGGACCC.............................................................    2439.801214
.....................GCUUGGUGCAGAUCGGGAC...............................................................    2458.957538
......................CUUGGUGCAGAUCGGGAC...............................................................    155.410411
......................CUUGGUGCAGAUCGGGACCCG............................................................    411.597374
..............................................................GAUCCCGCCUUGCAUCAAGUGAAU.................    1219.758685
...............................................................AUCCCGCCUUGCAUCAAGUGAAU.................    121.22218
.................................................................CCCGCCUUGCAUCAAGUGAA..................    3900.080282
.................................................................CCCGCCUUGCAUCAAGUGA...................    756.338417
.................................................................CCCGCUUUGCAUCAAGUGAAU.................    130.086865
.................................................................CUCGCCUUGCAUCAAGUGAAU.................    154.655082
.................................................................CCCUCCUUGCAUCAAGUGAAU.................    122.438012
.................................................................CCCGCCUUGCGUCAAGUGAAU.................    121.308014
.................................................................CCCGCCUUGCAUCAAGUGAAUA................    153.283691
.................................................................CCCGCCUUGCAUCAAGUGAAUU................    316.590706
.................................................................CCCGCCUUGCAUCAAGUGAAA.................    150.557935
.................................................................CCCGCCUUGCAUCAAGUGAAC.................    318.557785
..................................................................CCGCCUUGCAUCAAGUGAAU.................    2778.690419
...................................................................CGCCUUGCAUCAAGUGAAU.................    243.737544
```

zma-MIR169a

```
Chromosome:    chr3
Start:         37346668
End:           37346839
Strand:        +
Strand bias:   1.0
Abundance bias:0.803
MFE:           -95.10
```

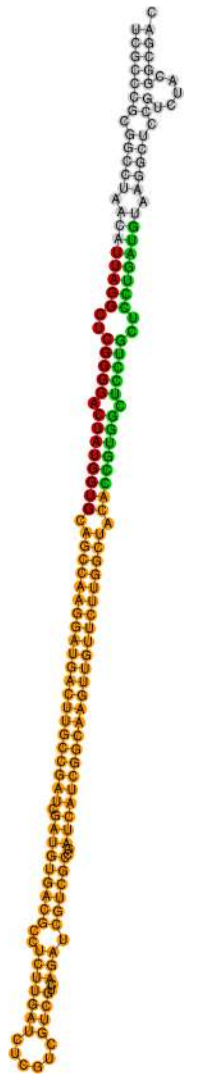

```
                  Mature                                                                                                       Star
UCGCCCGCGGCCUAACAUUAGGCUCGGGGACUAUGGUGCAGCCAAGGAUGACUUGCCGAUCGAUGUGACGCCUCUUGAUCUCGUCGUCGUCAGAUCGUCGCCGAUCAUCGGCAAGUUGUUCUUGGCUACACCGUGGCUCCUGCUCCUGAUGUAAGGCUCCGUCUACGGCGAC    21854.
(((((((.(((((.((((((((..(((((.((((((((.(((((((((((((((((((((.(((((((((..(((((((......))))..))).))))))..))))))))))))))))))))))))).)))))))).)))))..)))))))).))))).)).....))))).
.................UUAGGCUCGGGGACUAUGGUG..............................................................................................................................    7074.886708
..................................................................................................................................CCGUGGCUCCUGCUCCUGAUG.....................    906.65207
.................UUAGGCUCGGGGACUAUGGU...............................................................................................................................    9.712116
.................UUAGGCUCGGGGACUAUGG................................................................................................................................    58.393319
.................UUAGGCUCGGGGACUAUGGUGC.............................................................................................................................    444.758439
.................UUAGGCUCGGGGACUAUGGUGCA............................................................................................................................    1.95174
.................UUGGGCUCGGGGACUAUGGUG..............................................................................................................................    3.921292
.................UUAGGCUCGGGGACUAUGGCG..............................................................................................................................    2.43754
.................UUAGGCACGGGGACUAUGGUG..............................................................................................................................    1.577763
.................UUAGGCUCGGGGCCUAUGGUG..............................................................................................................................    1.37654
.................UUAGGCCCGGGGACUAUGGUG..............................................................................................................................    5.473758
.................CUAGGCUCGGGGACUAUGGUG..............................................................................................................................    1.28027
.................UUAGGCGCGGGGACUAUGGUG..............................................................................................................................    0.695843
.................UUAUGCUCGGGGACUAUGGUG..............................................................................................................................    0.874166
.................UUAGGCUUGGGGACUAUGGUG..............................................................................................................................    12.071571
.................UUAGGCUCGGGGACUCUGGUG..............................................................................................................................    0.963578
.................UUAGGCUCGGGGACCAUGGUG..............................................................................................................................    0.695843
.................UUAGGCUCGUGGACUAUGGUG..............................................................................................................................    3.07308
.................UUAGGCUCGGGGACUAUUGUG..............................................................................................................................    0.702532
.................UCAGGCUCGGGGACUAUGGUG..............................................................................................................................    567.389453
.................UUAGGCUCGGGGACUGUGGUG..............................................................................................................................    3.401603
.................UUAGGUUCGGGGACUAUGGUG..............................................................................................................................    4.214221
.................UUAGUCUCGGGGACUAUGGUG..............................................................................................................................    1.690413
.................UUAGGCUCGGGGACUAUAGUG..............................................................................................................................    1.14862
.................UUAGGCUCGGGGACUAUGGUC..............................................................................................................................    5.57475
.................UUAGGCUCGGGGACUAUGGUA..............................................................................................................................    36.73991
.................UUAGGCUCGGGGACUAUGGUU..............................................................................................................................    3.07164
.................UUAGGCUCGGGGGCUAUGGUG..............................................................................................................................    6.01308
.................UUAGGCUCGGGGACUAUGGGG..............................................................................................................................    0.699579
.................UUAGGCUCAGGGACUAUGGUG..............................................................................................................................    0.802164
.................UUAGGCUCGCGGACUAUGGUG..............................................................................................................................    3.65172
.................UUAGGCUCGGGGACUAUGGUGA.............................................................................................................................    4.263296
.................UUAGGCUCGGGGACUAUGGUGU.............................................................................................................................    3.691937
.................UUAGGCUCGAGGACUAUGGUG..............................................................................................................................    1.5315
.................UUAGGCUCGGGGACUAAGGUG..............................................................................................................................    10.68087
.................UUAGGCUCGGAGACUAUGGUG..............................................................................................................................    1.35015
..................UAGGCUCGGGGACUAUGGUG..............................................................................................................................    15.42026
...............................................................................................................................ACACCGUGGCUCCUGCUCCUGAUG.....................    29.994106
.................................................................................................................................ACCGUGGCUCCUGCUCCUGAUG.....................    8.42953
..................................................................................................................................CCGUGGCUCCUGCUCCUGAU......................    15.350934
..................................................................................................................................CCGUGGCUCCUGCUCCUGAUA.....................    38.63644
..................................................................................................................................CCGUGGCUCCUGCUCCUGAUU.....................    0.72271
```

zma-MIR169b

Chromosome: chr8
Start: 5012311
End: 5012517
Strand: -
Strand bias: 1.0
Abundance bias:0.984
MFE: -100.10

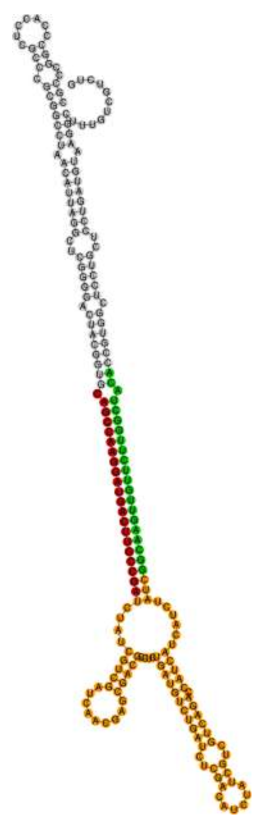

```
                                                   Mature                                                                                         Star
CGCCCGGCCCACCUCGCCCGCGGCCUAACAUUAGGCUCGGGGACUACGGUGCAGCCAAGGAUGACUUGCCGAUCUAUCGUCGAUCAACGAGCGACGCCUCUGAUGUCUGAUCUCGACAUCUAUCGUCGUCAGACCAUCAUCAUCUAUCGGCAAGUUGUUCUUGGCUACACCGUGGCUCCUGCUCCUGAUGUAAGGCUUUGUCGUCUG    17149.7
(((..(((.......))).)))((((.((((((((..(((((.((((((((.((((((((((((((((((((....(((((.........)))))....(((((((((((..(((......)))..)))))).)))))......)))))))))))))))))))).)))))))).)))))..)))))))).)))).............
...................................................CAGCCAAGGAUGACUUGCCGA.......................................................................................................................................    6631.483597
....................................................................................................................................................GGCAAGUUGUUCUUGGCUACA......................................    9.52403
.................................................UGCAGCCAAGGAUGACUUGCC.........................................................................................................................................    268.374131
..................................................GCAGCCAAGGAUGACUUGCCG........................................................................................................................................    17.006301
...................................................CAGCCAAGGAUGACUUGCCGAUCU....................................................................................................................................    8.07118
...................................................CAGCCAAGGAUGACUUGCCG........................................................................................................................................    473.978624
...................................................CAGCCAAGGAUGACUUGCC.........................................................................................................................................    50.579529
...................................................CAGCCAAGGAUGACUUGCCGAU......................................................................................................................................    5.050775
...................................................CAGCCAAGGAUGACUUGC..........................................................................................................................................    72.683654
...................................................CAGCCAAGGAUGACUUGCAGA.......................................................................................................................................    3.624994
...................................................CAGCCAAGGAUGACUUGCCUA.......................................................................................................................................    15.598
...................................................CAGCCAAGGAUGACUCGCCGA.......................................................................................................................................    1.679984
...................................................CAGCCAAGGAUGGCUUGCCGA.......................................................................................................................................    7.727846
...................................................CAGCCAAGGAUGACUUGCUGA.......................................................................................................................................    9.775503
...................................................CAUCCAAGGAUGACUUGCCGA.......................................................................................................................................    3.348398
...................................................CAGCCAAGGUUGACUUGCCGA.......................................................................................................................................    2.69021
...................................................CAGCCAAGGAUGACUUGCU.........................................................................................................................................    11.201855
...................................................CAGCCAAGGAUGCCUUGCCGA.......................................................................................................................................    8.15509
...................................................CAGCCAAGGAUGACUUGCCGGU......................................................................................................................................    294.662487
...................................................CAGCCAAGGAUGACUUGCCGGUC.....................................................................................................................................    0.905431
...................................................CAGCGAAGGAUGACUUGCCGA.......................................................................................................................................    1.56855
...................................................CAGCCACGGAUGACUUGCCGA.......................................................................................................................................    4.348008
...................................................CAGCCAAAGAUGACUUGCCGA.......................................................................................................................................    1.15727
...................................................CAGCCGAGGAUGACUUGCCGA.......................................................................................................................................    2.219697
...................................................CAGCCAAGGGUGACUUGCCGA.......................................................................................................................................    3.60541
...................................................CAGCCAAGGAUGACUUGCCGU.......................................................................................................................................    317.296539
...................................................CAGCCAAGGAUGACUUGCCGC.......................................................................................................................................    270.570039
...................................................CAGCCAAGGAUGACUUGUCGA.......................................................................................................................................    2.496357
...................................................CAGCUAAGGAUGACUUGCCGA.......................................................................................................................................    1.15727
...................................................CAGUCAAGGAUGACUUGCCGA.......................................................................................................................................    3.65461
...................................................CAGCCAAGGCUGACUUGCCGA.......................................................................................................................................    2.29725
...................................................CAGCCAAGGAUGACUUCCCGA.......................................................................................................................................    1.35015
...................................................CAGCCAAGGAUGAUUUGCCGA.......................................................................................................................................    6.466969
...................................................UAGCCAAGGAUGACUUGCCGA.......................................................................................................................................    18.729533
...................................................CAGCCAAGGAUGACUUGCCGAA......................................................................................................................................    10.070754
...................................................CAGCCAGGGAUGACUUGCCGA.......................................................................................................................................    1.616528
...................................................CGGCCAAGGAUGACUUGCCGA.......................................................................................................................................    6.365082
...................................................CAGCCAAGGACGACUUGCCGA.......................................................................................................................................    4.371661
...................................................CAGCCAAGGAUGACCUGCCGA.......................................................................................................................................    4.866592
....................................................AGCCAAGGAUGACUUGCCGAU......................................................................................................................................    338.121005
....................................................AGCCAAGGAUGACUUGCCGA.......................................................................................................................................    279.790175
....................................................AGCCAAGGAUGACUUGCCG........................................................................................................................................    73.961898
....................................................AGCCAAGGAUGACUUGCCGU.......................................................................................................................................    1.5598
.....................................................GCCAAGGAUGACUUGCCG........................................................................................................................................    3.39119
......................................................CCAAGGAUGACUUGCCGAUCU....................................................................................................................................    2.383709
.................................................................................................................................................AUCGGCAAGUUGUUCUUGGCU.........................................    861.426235
.................................................................................................................................................AUCGGCAAGUUGUUCUUGGCUACA......................................    13.32237
.................................................................................................................................................AUCGGCAAGUUGUUCUUGGCUAC.......................................    64.638996
.................................................................................................................................................AUCGGCAAGUUGUUCUUGGCUA........................................    3.001902
.................................................................................................................................................AUCGGCAAGUUGUUCUUGGCA.........................................    4.55406
.................................................................................................................................................AUCGGCAAGUUGUUCUUGGCUACU......................................    41.029711
..................................................................................................................................................UCGGCAAGUUGUUCUUGGCUAC.......................................    115.027521
...................................................................................................................................................CGGCAAGUUGUUCUUGGCUA........................................    46.682934
...................................................................................................................................................CGGCAAGUUGUUCUUGGCUAC.......................................    274.98141
...................................................................................................................................................CGGCAAGUUGUUCUUGGCU.........................................    15.322515
....................................................................................................................................................GGCAAGUUGUUCUUGGCUAC.......................................    41.41629
....................................................................................................................................................GGCAAGUUGUUCUUGGCUA........................................    31.909212
....................................................................................................................................................GGCAAGUUGUUCUUGGCUACU......................................    1.676894
```

zma-MIR169c

```
Chromosome:     chr2
Start:          11553341
End:            11553470
Strand:         +
Strand bias:    1.0
Abundance bias:0.971
MFE:            -66.00
```

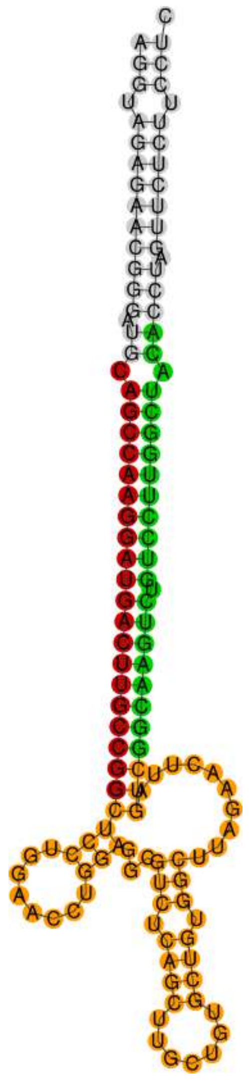

```
                 Mature                                                                     Star
AGGUAGAGAACGGGAUGCAGCCAAGGAUGACUUGCCGGCUCCUGGAACCUGGAGGCGUCUCAGCUUGCUGUGCUGUGGCUUAGAACUUAGUCGGCAAGUCUGUCCUUGGCUACACCUAGUUCUCUUCCUC   278183.1
(((.(((((((((.((.((((((((((((((((((((((........)))...(((.((((.......)))).)))..........)))))))))).))))))))).))))).))))))).))).
.................CAGCCAAGGAUGACUUGCCGG............................................................................................   247937.35687
............................................................................................GGCAAGUCUGUCCUUGGCUACA................   591.523483
..............AUGCAGCCAAGGAUGACUUGCC..............................................................................................   2.60232
...............UGCAGCCAAGGAUGACUUGCC..............................................................................................   268.374131
................GCAGCCAAGGAUGACUUGCCG.............................................................................................   17.006301
................GCAGCCAAGGAUGACUUGCCGG............................................................................................   2.859052
.................CAGCCAAGGAUGACUUGCCGGCU..........................................................................................   55.993032
.................CAGCCAAGGAUGACUUGCCG.............................................................................................   473.978624
.................CAGCCAAGGAUGACUUGCCGGC...........................................................................................   385.705689
.................CAGCCAAGGAUGACUUGCC..............................................................................................   50.579529
.................CAGCCAAGGAUGACUUGC...............................................................................................   72.683654
.................CAGCCAAGGAUGACGUGCCGG............................................................................................   35.045053
.................CAGCAAAGGAUGACUUGCCGG............................................................................................   44.839435
.................CAGCCAAGGAUGACUUGCAGG............................................................................................   93.848025
.................CAGCCAAGGAGGACUUGCCGG............................................................................................   62.751172
.................CAGCCAAGGAUGACUCGCCGG............................................................................................   377.867184
.................CAGCCAAGGAUGGCUUGCCGG............................................................................................   574.818153
.................CAGCCAACGAUGACUUGCCGG............................................................................................   199.179311
.................CUGCCAAGGAUGACUUGCCGG............................................................................................   158.522553
.................CAUCCAAGGAUGACUUGCCGG............................................................................................   374.883267
.................CAGCCAAGGAUGACUUUCCGG............................................................................................   122.35452
.................CCGCCAAGGAUGACUUGCCGG............................................................................................   75.540159
.................CAGCCAAGGAUGACUUGCU..............................................................................................   11.201855
.................CAGACAAGGAUGACUUGCCGG............................................................................................   85.223964
.................CAGCCAAGGUUGACUUGCCGG............................................................................................   50.584114
.................CAGGCAAGGAUGACUUGCCGG............................................................................................   46.649705
.................CAGCCAAGCAUGACUUGCCGG............................................................................................   40.466661
.................CAGCCAAGGAUGAGUUGCCGG............................................................................................   12.644183
.................CAGCCAAGGAUGACUUGCCUG............................................................................................   94.083396
.................CAGCCAAGGAUGACUUGGCGG............................................................................................   16.403735
.................CAGCCAAGGAUGCCUUGCCGG............................................................................................   247.681843
.................CAGCCAAGGAUGACUUGCCGGU...........................................................................................   294.662487
.................CAGCCAAGGAUGACUUGCCGGA...........................................................................................   125.435506
.................CAGCCAAGGAUCACUUGCCGG............................................................................................   68.161118
.................CAGCCAAUGAUGACUUGCCGG............................................................................................   463.53416
.................CAGCCAAGGAUGACUUGCGGG............................................................................................   46.586351
.................CAGCCAAGGAUGACUAGCCGG............................................................................................   28.298691
.................CACCCAAGGAUGACUUGCCGG............................................................................................   143.405442
.................CAGCCAAGUAUGACUUGCCGG............................................................................................   66.868994
.................CAGCCAAGGAUGACAUGCCGG............................................................................................   57.942346
.................CAACCAAGGAUGACUUGCCGG............................................................................................   261.360798
.................CAGCCAAGGAUGACUUGCCAG............................................................................................   237.1791
.................CAGCCAAGGAUGACUUACCGG............................................................................................   179.22605
.................CAGCGAAGGAUGACUUGCCGG............................................................................................   25.653136
.................CAGCCACGGAUGACUUGCCGG............................................................................................   391.935194
.................CAGCCAUGGAUGACUUGCCGG............................................................................................   88.807161
.................CAGCCAAAGAUGACUUGCCGG............................................................................................   95.218492
.................CAGCCAAGGAUUACUUGCCGG............................................................................................   195.977061
.................CAGCCAAGGGUGACUUGCCGG............................................................................................   496.100604
.................CAGCCAAGGAUGACUGGCCGG............................................................................................   15.236471
.................AAGCCAAGGAUGACUUGCCGG............................................................................................   185.461084
.................CAGCCAAGGAUGACUUGCCGU............................................................................................   317.296539
.................CAGCCAAGGAUGACUUGCCGC............................................................................................   270.570039
.................CAGCCAAGGAUGAAUUGCCGG............................................................................................   43.926747
.................CAGCUAAGGAUGACUUGCCGG............................................................................................   203.866094
.................CAGCCUAGGAUGACUUGCCGG............................................................................................   95.555312
.................CAGUCAAGGAUGACUUGCCGG............................................................................................   239.597198
.................CAGCCAAGGCUGACUUGCCGG............................................................................................   50.454235
.................GAGCCAAGGAUGACUUGCCGG............................................................................................   124.290936
.................CAGCCAAGGAUGUCUUGCCGG............................................................................................   107.777957
.................CAGCCAAGGAUGACUUCCCGG............................................................................................   121.29565
.................CAGCCAAGGAUGAUUUGCCGG............................................................................................   218.488921
.................CAGCCAAGGAAGACUUGCCGG............................................................................................   35.647398
.................CAGCCAGGGAUGACUUGCCGG............................................................................................   487.04577
.................CAGCCAAGAAUGACUUGCCGG............................................................................................   108.850438
.................CAGCCAAGGAUGACUUGUCGG............................................................................................   280.20557
.................CAGCCAAGGAUGACUUGCCCG............................................................................................   48.602191
.................CAGCCAAGGAUGACUUGCUGG............................................................................................   282.58343
.................CGGCCAAGGAUGACUUGCCGG............................................................................................   424.57992
.................CAGCCGAGGAUGACUUGCCGG............................................................................................   450.82641
.................CAGCCAAGGACGACUUGCCGG............................................................................................   425.836338
.................CAGCCCAGGAUGACUUGCCGG............................................................................................   74.94026
.................CAGCCAAGGAUAACUUGCCGG............................................................................................   108.585573
.................CAGCCAAGGAUGACUUGACGG............................................................................................   70.403765
.................CAGCCAAGGAUGACCUGCCGG............................................................................................   420.010511
..................AGCCAAGGAUGACUUGCCGGC...........................................................................................   182.467562
..................AGCCAAGGAUGACUUGCCGG............................................................................................   3626.68943
..................AGCCAAGGAUGACUUGCCG.............................................................................................   73.961898
...................GCCAAGGAUGACUUGCCGG............................................................................................   76.694391
...................GCCAAGGAUGACUUGCCG.............................................................................................   3.39119
....................CCAAGGAUGACUUGCCGG............................................................................................   95.567775
..........................................................................................UCGGCAAGUCUGUCCUUGGCUAC.................   342.620363
..........................................................................................UCGGCAAGUCUGUCCUUGGCUACA................   26.10732
..........................................................................................UCGGCAAGUCUGUCCUUGGCUACU................   14.7349
...........................................................................................CGGCAAGUCUGUCCUUGGCUAC.................   58.674552
............................................................................................GGCAAGUCUGUCCUUGGCUA..................   1106.262822
............................................................................................GGCAAGUCUGUCCUUGGCU...................   243.638665
............................................................................................GGCAAGUCUGUCCUUGGCUAC.................   2470.730053
............................................................................................GGCAAGUCUGUCCUUGGCUACUC...............   3.57819
............................................................................................GGCAAGUCUGUCCUUGGCUAU.................   36.540561
............................................................................................GGCAAGUCUGUCCUUGGCUAA.................   52.711078
............................................................................................GGCAAGUCUGUCCUUGGCUACC................   4.40738
............................................................................................GGCAAGUCUGUCCUUGGCUACU................   287.111631
............................................................................................GGCAAGUCUGUCCUUGGCUACAU...............   4.233832
............................................................................................GGCAAGUCUGUCCUUGGCCA..................   3.57819
.............................................................................................GCAAGUCUGUCCUUGGCUAC.................   12.680768
..............................................................................................CAAGUCUGUCCUUGGCUAC.................   5.846639
..............................................................................................CAAGUCUGUCCUUGGCUACACC..............   304.129005
```

zma-MIR169g

Chromosome: chr2
Start: 67281152
End: 67281286
Strand: +
Strand bias: 1.0
Abundance bias: 0.991
MFE: -57.90

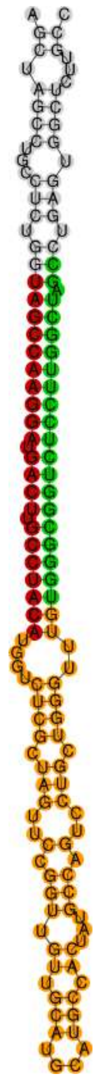

```
                         Mature                                                                         Star
AGCUAGCCUGCCUCUGGUAGCCAAGGAUGACUUGCCUACAUGGUCUCGCUAGUUCCGGUUGUUGCAUGCAUGCCACUAUGCCAGUCCUGCUGGGUUUGUGGGCGGUCUCCUUGGCUAGCCUGAGUGGCUCUUGCC    7849.6
.((.((((...(((.((((((((((((.((((.(((((((....((((.(((..(.(((.((.(((....))).))...))).)..))).))))..))))))))))))))))))))).))).))).))))...)).
.................UAGCCAAGGAUGACUUGCCUACA...............................................................................................    367.301421
..................................................................................................UGGGCGGUCUCCUUGGCUAGC................    3315.264803
.................UAGCCAAGGAUGACUUGCCU..................................................................................................    1202.958549
.................UAGCCAAGGAUGACUUGCCUA.................................................................................................    1737.40401
.................UAGCCAAGGAUGACUUGCCUAC................................................................................................    2.4669
.................UAGCCAAGGAUGACUUGUCUA.................................................................................................    1.5598
.................UAGCCAAGGAUGACUUGCCA..................................................................................................    2.633131
.................UAGCCAAGGAUGACUCGCCUA.................................................................................................    2.59967
.................UAGCCAAGGAUGACUUGCCUU.................................................................................................    50.494015
.................UAGCCAAGGAUGACUUGCCUC.................................................................................................    6.51412
.................UAGCCAAGGAUGACUUGCCUACU..............................................................................................    3.210962
.................UAGCCAAGGAUGAUUUGCCUA.................................................................................................    2.85964
.................UAGCCAAGGAUGAAUUGCCUA.................................................................................................    1.81977
.................UAGCCAAGGAUGACCUGCCUA.................................................................................................    3.37957
.................UAGCCAAGGAUGACUUGACUA.................................................................................................    2.3397
.................UAGCCAAGGAUGCCUUGCCUACA...............................................................................................    3.92556
.................UAGCCAAGGAUCACUUGCCUA.................................................................................................    2.07974
.................UAGCCAAGGAUGACUUGCAUA.................................................................................................    2.3397
.................UAGCCAAGGAUGACUGGCCUA.................................................................................................    1.5598
.................UAGCCAAGGAUGACUUGCCUAAA..............................................................................................    1.1864
.................UAGCCAAGGAUGACUUGCCGA.................................................................................................    18.729533
.................UAGCCAAGGAUUACUUGCCUA.................................................................................................    1.5598
.................UAGCCAAGGAUGACUUUCCUA.................................................................................................    5.19934
.................UAGCCAAGGAGGACUUGCCUA.................................................................................................    2.3397
.................UAGCCAAGGAUGACGUGCCUA.................................................................................................    2.07974
.................UAGCCAAGGAUGCCUUGCCUA.................................................................................................    3.096958
.................UAGCCACGGAUGACUUGCCUACA...............................................................................................    0.64713
.................UAGCCAAGGAUGACUUCCCUA.................................................................................................    2.3397
..................AGCCAAGGAUGACUUGCCU..................................................................................................    25.069596
..................AGCCAAGGAUGACUUGCCUACA...............................................................................................    6.94365
...................GCCAAGGAUGACUUGCCU..................................................................................................    2.047482
.................................................................................................GUGGGCGGUCUCCUUGGCUAGC................    1.44198
..................................................................................................UGGGCGGUCUCCUUGGCUAGCC...............    801.245466
..................................................................................................UGGGCGGUCUCCUUGGCUAG.................    31.626995
..................................................................................................UGGGCGGUCUCCUUGGCU...................    20.72864
..................................................................................................UGGGCGGUCUCCUUGGCUAGCU...............    0.182396
..................................................................................................UGGGCGGUCUCCUUGGCUAGU................    100.075683
..................................................................................................UGGGCGGUCUCCUUGGCUAGA................    34.770279
..................................................................................................UGGGCGGUCUCCUUGGCA...................    1.037
..................................................................................................UGGGCGGUCUCCUUGGCC...................    1.752719
..................................................................................................UGGUCGGUCUCCUUGGCUAGC................    3.544
..................................................................................................UGGGCGGUCUCCUUGGCUAGGC...............    0.530802
...................................................................................................GGGCGGUCUCCUUGGCUAGC................    0.318481
...................................................................................................GGGCGGUCUCCUUGGCUAGCC...............    0.530802
```

zma-MIR169h

Chromosome: chr4
Start: 49625335
End: 49625509
Strand: +
Strand bias: 1.0
Abundance bias: 0.978
MFE: -79.30

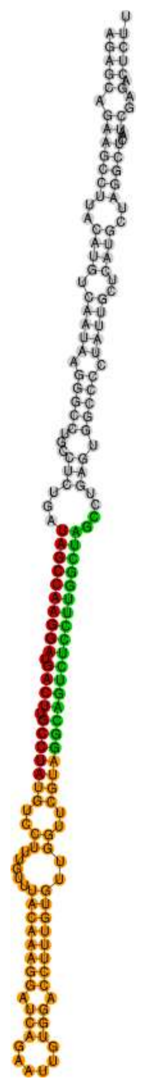

```
                                        Mature                                                                              Star
AGAGCAGAAGCCUUACAUGUCAAUAAGGGCCUGCCUCUGAUAGCCAAGGAUGACUUGCCUAUGUCCUUUGUUUACAAAGGAUCAGAAUUGUGGACCUUUGUGUUGGUUCGUAGGCAGUCUCCUUGGCUAGCCUGAGUGGCCCCUAUUGCUCAUGCUAGGCUCAAUCGAGACUCUU   9661.
(((((.(((((((..((((.(((((.(((((...(((...((((((((((.((((.(((((((..((.....((((((((.(((......))).))))))))..))..)))))))))))))))))))))....))).))))).))))).))))..))))..)))))...))..).)))).
........................................UAGCCAAGGAUGACUUGCCUA..................................................................................................................   1737.40401
................................................................................................................GGCAGUCUCCUUGGCUAGC............................................   4934.447462
........................................UAGCCAAGGAUGACUUGCCU...................................................................................................................   1202.958549
........................................UAGCCAAGGAUGACUUGC.....................................................................................................................   50.854203
........................................UAGCCAAGGAUGACUUGCC....................................................................................................................   17.032323
........................................UAGCCAAGGAUGACUUGUCUA..................................................................................................................   1.5598
........................................UAGCCAAGGAUGACUUGCCA...................................................................................................................   2.633131
........................................UAGCCAAGGAUGACUCGCCUA..................................................................................................................   2.59967
........................................CAGCCAAGGAUGACUUGCCUA..................................................................................................................   15.598
........................................UAGCCAAGGAUGACUUGCCUU..................................................................................................................   50.494015
........................................UAGCCAAGGAUGACUUGCCUC..................................................................................................................   6.51412
........................................UAGCCAAGGAUGACUUGU.....................................................................................................................   5.4679
........................................UAGCCACGGAUGACUUGCCUA..................................................................................................................   1.81977
........................................UAGCCAAGGAUGAUUUGCCUA..................................................................................................................   2.85964
........................................UAGCCAAGGAUGAAUUGCCUA..................................................................................................................   1.81977
........................................UAGCCAAGGAUGACCUGCCUA..................................................................................................................   3.37957
........................................UAUCCAAGGAUGACUUGCCUA..................................................................................................................   3.89951
........................................UAGCUAAGGAUGACUUGCCUA..................................................................................................................   2.07974
........................................UAGCCAAGGAUGACUUGACUA..................................................................................................................   2.3397
........................................UAGCCAAGGAUCACUUGCCUA..................................................................................................................   2.07974
........................................UAGCCAAGGAUGACUUGCAUA..................................................................................................................   2.3397
........................................UAGCCAAGGAUGACUGGCCUA..................................................................................................................   1.5598
........................................UAGCCAAGGAUGACUUGCCGA..................................................................................................................   18.729533
........................................UAGCCAAGGAUUACUUGCCUA..................................................................................................................   1.5598
........................................UAGCCAAGGAUGACUUUCCUA..................................................................................................................   5.19934
........................................UAGCCAAGGAGGACUUGCCUA..................................................................................................................   2.3397
........................................UAGCCAAGGAUGACGUGCCUA..................................................................................................................   2.07974
........................................UAGCCAAGGAUGCCUUGCCUA..................................................................................................................   3.096958
........................................UAGACAAGGAUGACUUGCCUA..................................................................................................................   1.81977
........................................UAGCCAAGGAUGACUUGCU....................................................................................................................   8.984663
........................................UAGCAAAGGAUGACUUGCCUA..................................................................................................................   2.59967
........................................UAGCCAAGGAUGACUUCCCUA..................................................................................................................   2.3397
.........................................AGCCAAGGAUGACUUGCCU...................................................................................................................   25.069596
..........................................GCCAAGGAUGACUUGCCU...................................................................................................................   2.047482
.............................................................................................................GUAGGCAGUCUCCUUGGCUAGC............................................   0.530802
..............................................................................................................UAGGCAGUCUCCUUGGCUAGC............................................   16.781196
...............................................................................................................AGGCAGUCUCCUUGGCUAG.............................................   0.371561
...............................................................................................................AGGCAGUCUCCUUGGCUAGCC...........................................   1.39692
...............................................................................................................AGGCAGUCUCCUUGGCUAGC............................................   113.434077
...............................................................................................................AGGCAGUCUCCUUGGCUAGA............................................   0.212795
...............................................................................................................AGGCAGUCUCCUUGGCUAGGC...........................................   0.318481
................................................................................................................GGCAGUCUCCUUGGCUAGCC...........................................   996.802148
................................................................................................................GGCAGUCUCCUUGGCUAG.............................................   61.029283
................................................................................................................GGCAGUCUCCUUGGCUAGCCU..........................................   0.477722
................................................................................................................GGCAGUCUCCUUGGCUAGA............................................   7.31334
................................................................................................................GGCAGUCUCCUUGGCUAGUCU..........................................   0.530802
................................................................................................................GGCAGGCUCCUUGGCUAGC............................................   0.849283
................................................................................................................GGCAGUCUCCUUGGCUAACC...........................................   1.245067
................................................................................................................GGCAGUCUCCUCGGCUAGC............................................   0.552991
................................................................................................................GGCAGUCUCCUUGGCUAGGC...........................................   2.76017
................................................................................................................GGCAGUCUCCUUGGCUAGCA...........................................   2.92761
................................................................................................................GGCAGUCUCCUUGGCUAGCU...........................................   15.56201
................................................................................................................GGCAGUCUCUUUGGCUAGC............................................   0.395191
................................................................................................................GGCAGUCUCCUUGGCUAAC............................................   0.424642
.................................................................................................................GCAGUCUCCUUGGCUAGCCU..........................................   1.95174
.................................................................................................................GCAGUCUCCUUGGCUAGCC...........................................   74.918436
.................................................................................................................GCAGUCUCCUUGGCUAGC............................................   21.106916
..................................................................................................................CAGUCUCCUUGGCUAGCC...........................................   1.22084
```

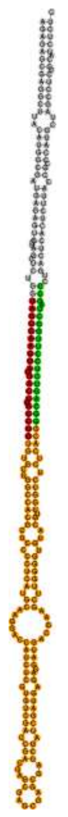

zma-MIR169i

Chromosome: chr4
Start: 49299317
End: 49299552
Strand: +
Strand bias: 1.0
Abundance bias:0.873
MFE: -115.20

```
                                         Mature                                                                                                                      Star
AGAGAGCGAGGCUUACAUGGCGAUGAGAGUGGUAGCUCUGGUAGCCAAGGAUGACUUGCCUGUGUGCUGGCCACGCUCCCCUCAUGCAAGGACCAUCUCGUGUCGACCGACGAGCGAGCGAGCGAUCGAUCGAUGAGAGGAUGACGAAGCUUGGGGUGUACGUUGGUCUCUCACGGGCAGUCUCCUUGGCUAGCCCUGACUCACUCUUACCGCCCAUGCUAGCCUCUGCAUCUCUC   10973.1
(((((((((((((..(((((((.((((((((..((.((.((((((((((((.((((.(((((((.(..(((((((..(.(((((.((......((((((.((((((.(((...((......))..))).)))))).)).)))).....)).))))).)..)).))))).).)))))))))))))))))))).)))..)))))))))))).)).)))))..)))))).)).))))).
.........................................UAGCCAAGGAUGACUUGCCUG..............................................................................................................................................................................   1421.834235
...............................................................................................................................................................................GGCAGUCUCCUUGGCUAGCC.........................................   996.802148
.......................................GGUAGCCAAGGAUGACUUGCCUG..............................................................................................................................................................................   2.761795
.........................................UAGCCAAGGAUGACUUGCCU...............................................................................................................................................................................   1202.958549
.........................................UAGCCAAGGAUGACUUGC.................................................................................................................................................................................   50.854203
.........................................UAGCCAAGGAUGACUUGCCUGU.............................................................................................................................................................................   159.179932
.........................................UAGCCAAGGAUGACUUGCC................................................................................................................................................................................   17.032323
.........................................UAGCCAAGGAUGACUUGCCA...............................................................................................................................................................................   2.633131
.........................................UAGCCAAGGAUGCCUUGCCUG..............................................................................................................................................................................   0.754985
.........................................UAGCCAAGGAUGACUUGCCUU..............................................................................................................................................................................   50.494015
.........................................UAGCCAAGGAUGACUUGCCUC..............................................................................................................................................................................   6.51412
.........................................UAGCCAAGGAUGACUUGU.................................................................................................................................................................................   5.4679
.........................................UAGCCAAGGAUGACUCGCCUG..............................................................................................................................................................................   1.56976
.........................................CAGCCAAGGAUGACUUGCCUG..............................................................................................................................................................................   94.083396
.........................................UAGCCCAGGAUGACUUGCCUG..............................................................................................................................................................................   3.40115
.........................................UAGCCAAGGAUGACCUGCCUG..............................................................................................................................................................................   9.38869
.........................................UAGCCAAUGAUGACUUGCCUG..............................................................................................................................................................................   1.56976
.........................................GAGCCAAGGAUGACUUGCCUG..............................................................................................................................................................................   3.12956
.........................................UAGCCUAGGAUGACUUGCCUG..............................................................................................................................................................................   2.09301
.........................................UAGCCAAGGAUGACUUGCCUGA.............................................................................................................................................................................   60.555545
.........................................UAGCCGAGGAUGACUUGCCUG..............................................................................................................................................................................   3.13952
.........................................UAGCCAAGGAUGAGUUGCCUG..............................................................................................................................................................................   6.2641
.........................................UAGCCAAGGAUGACUUUCCUG..............................................................................................................................................................................   1.5598
.........................................UAGCCAAGGAUGACUUGCU................................................................................................................................................................................   8.984663
.........................................UAGCCAAGGAUGACUUCCUG...............................................................................................................................................................................   1.5598
..........................................AGCCAAGGAUGACUUGCCU...............................................................................................................................................................................   25.069596
..........................................AGCCAAGGAUGACUUGCCUG..............................................................................................................................................................................   2.860526
..........................................AGCCAAGGAUGACUUGCCGGU.............................................................................................................................................................................   0.552991
...........................................GCCAAGGAUGACUUGCCU...............................................................................................................................................................................   2.047482
............................................................................................................................................................................ACGGGCAGUCUCCUUGGCUAGCC.........................................   0.371561
............................................................................................................................................................................ACGGGCAGUCUCCUUGGCUAGC..........................................   8.88795
............................................................................................................................................................................ACGGGCAGUCUCCUUGGCUA............................................   0.273594
.............................................................................................................................................................................CGGGCAGUCUCCUUGGCUAGC..........................................   27.930455
.............................................................................................................................................................................CGGGCAGUCUCCUUGGCUAGCC.........................................   6.5832
...............................................................................................................................................................................GGCAGUCUCCUUGGCUAGC..........................................   4934.447462
...............................................................................................................................................................................GGCAGUCUCCUUGGCUAG...........................................   61.029283
...............................................................................................................................................................................GGCAGUCUCCUUGGCUAGCCC........................................   162.34696
...............................................................................................................................................................................GGCAGUCUCCUUGGCUAGA..........................................   7.31334
...............................................................................................................................................................................GGCAGUCUCCUUGGCUAACC.........................................   1.245067
...............................................................................................................................................................................GGCAGUCUCCUCGGCUAGC..........................................   0.552991
...............................................................................................................................................................................GGCAGUCUCCUUGGCUAGCCA........................................   7.80695
...............................................................................................................................................................................GGCAGUCUCCUUGGCUAGGC.........................................   2.76017
...............................................................................................................................................................................GGCAGUCUCCUUGGCUAGCA.........................................   2.92761
...............................................................................................................................................................................GGCAGUCUCCUUGGCUAGCU.........................................   15.56201
...............................................................................................................................................................................GGCAGUCUCUUUGGCUAGC..........................................   0.395191
...............................................................................................................................................................................GGCAGUCUCCUUGGCUAAC..........................................   0.424642
................................................................................................................................................................................GCAGUCUCCUUGGCUAGCC.........................................   74.918436
................................................................................................................................................................................GCAGUCUCCUUGGCUAGCCC........................................   85.78191
................................................................................................................................................................................GCAGUCUCCUUGGCUAGC..........................................   21.106916
................................................................................................................................................................................GCAGUCUCCUUGGCUAGCCA........................................   5.85522
.................................................................................................................................................................................CAGUCUCCUUGGCUAGCCC........................................   0.796203
.................................................................................................................................................................................CAGUCUCCUUGGCUAGCC.........................................   1.22084
```

zma-MIR169j

Chromosome: chr2
Start: 198435911
End: 198436059
Strand: +
Strand bias: 1.0
Abundance bias: 1.0
MFE: -70.20

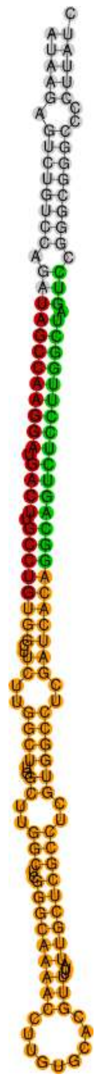

Mature   Star

```
AUAAGAGUCUGUCCAGAUAGCCAAGGAUGACUUGCCUGUGGCUUCUUGGCUUGGCUUGGCUCGGGCAAAACCUUGUGCACGUUUUAUUGCUCGCCUCGUGGCCUCGAUCACAGGCAGUCUCCUUGGCUAGUCCGGGCGGGCCCCUUAUC    4057.2
(((((.((((((((.((((((((((((.((((.((((((((..((..((((..((..(((..(((((((((.........)))...)))))))))..))))))..))))))))))))))))))))))))).))).))))))))..))))).
.................UAGCCAAGGAUGACUUGCCUG...............................................................................................................    1421.834235
................................................................................................................GGCAGUCUCCUUGGCUAGUC.................    500.134793
.................UAGCCAAGGAUGACUUGCCU................................................................................................................    1202.958549
.................UAGCCAAGGAUGACUUGC..................................................................................................................    50.854203
.................UAGCCAAGGAUGACUUGCCUGU..............................................................................................................    159.179932
.................UAGCCAAGGAUGACUUGCC.................................................................................................................    17.032323
.................UAGCCAAGGAUGACUUGCCA................................................................................................................    2.633131
.................UAGCCAAGGAUGCCUUGCCUG...............................................................................................................    0.754985
.................UAGCCAAGGAUGACUUGCCUU...............................................................................................................    50.494015
.................UAGCCAAGGAUGACUUGCCUC...............................................................................................................    6.51412
.................UAGCCAAGGAUGACUUGU..................................................................................................................    5.4679
.................UAGCCAAGGAUGACUCGCCUG...............................................................................................................    1.56976
.................CAGCCAAGGAUGACUUGCCUG...............................................................................................................    94.083396
.................UAGCCCAGGAUGACUUGCCUG...............................................................................................................    3.40115
.................UAGCCAAGGAUGACCUGCCUG...............................................................................................................    9.38869
.................UAGCCAAUGAUGACUUGCCUG...............................................................................................................    1.56976
.................GAGCCAAGGAUGACUUGCCUG...............................................................................................................    3.12956
.................UAGCCUAGGAUGACUUGCCUG...............................................................................................................    2.09301
.................UAGCCAAGGAUGACUUGCCUGA..............................................................................................................    60.555545
.................UAGCCGAGGAUGACUUGCCUG...............................................................................................................    3.13952
.................UAGCCAAGGAUGAGUUGCCUG...............................................................................................................    6.2641
.................UAGCCAAGGAUGACUUUCCUG...............................................................................................................    1.5598
.................UAGCCAAGGAUGACUUGCU.................................................................................................................    8.984663
.................UAGCCAAGGAUGACUUCCCUG...............................................................................................................    1.5598
..................AGCCAAGGAUGACUUGCCU................................................................................................................    25.069596
..................AGCCAAGGAUGACUUGCCUG...............................................................................................................    2.860526
..................AGCCAAGGAUGACUUGCCGGU..............................................................................................................    0.552991
...................GCCAAGGAUGACUUGCCU................................................................................................................    2.047482
.............................................................................................................ACAGGCAGUCUCCUUGGCUAG...................    0.212795
..............................................................................................................CAGGCAGUCUCCUUGGCUAGU..................    4.68033
..............................................................................................................CAGGCAGUCUCCUUGGCUAG...................    0.797151
..............................................................................................................CAGGCAGUCUCCUUGGCUAGUU.................    0.318481
...............................................................................................................AGGCAGUCUCCUUGGCUAG...................    0.371561
...............................................................................................................AGGCAGUCUCCUUGGCUAGUC.................    25.232044
...............................................................................................................AGGCAGUCUCCUUGGCUAGU..................    48.55055
...............................................................................................................AGGCAGUCUCCUUGGCUAGA..................    0.212795
...............................................................................................................AGGCAGUCUCCUUGGCUAGGC.................    0.318481
................................................................................................................GGCAGUCUCCUUGGCUAGU..................    250.874701
................................................................................................................GGCAGUCUCCUUGGCUAG...................    61.029283
................................................................................................................GGCAGUCUCCUUGGCUAGA..................    7.31334
................................................................................................................GGCAGUCUCCUUGGCUAAUC.................    1.64549
................................................................................................................GGCAGUCUCCUUGGCUAGUCU................    0.530802
................................................................................................................GGCAGUCUCCUUGGCUAGGC.................    2.76017
................................................................................................................GGCAGUCUCCUUGGCUAGUU.................    6.688392
```

zma-MIR169k

Chromosome: chr7
Start: 142286583
End: 142286758
Strand: +
Strand bias: 1.0
Abundance bias: 0.925
MFE: -106.50

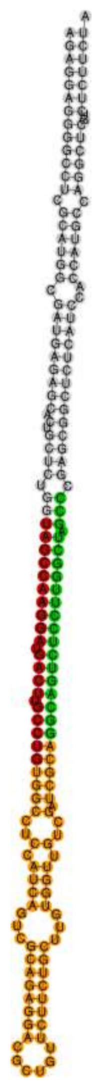

```
                                        Mature                                                                           Star
AGAGGAGGGGCCUCGCAUGGCGAUGAGAGCACUGCUCUGGUAGCCAAGGAUGACUUGCCUGUGGCCUCCAUCAGUCGCAGAGGACGCUGUUCUUCUGCUUGUGGUUGUCGAUCGCAGGCAGUCUCCUUGGCUAGCCCGAGCGGCUCUCAUCCACCAUGCCAGGCUCGUCUCUUCUA    10434.
((((((((((((.((((((.(((((((((.(.((((.(((((((((((.((((.((((((((..(.((((...((((((((......))))))))...)))).)..).))))))))))))))))))))))).))).))))))))))))))..)))))).))))))..))))))).
........................................UAGCCAAGGAUGACUUGCCUG...................................................................................................................    1421.834235
....................................................................................................................GGCAGUCUCCUUGGCUAGCC........................................    996.802148
......................................GGUAGCCAAGGAUGACUUGCCUG...................................................................................................................    2.761795
........................................UAGCCAAGGAUGACUUGCCU....................................................................................................................    1202.958549
........................................UAGCCAAGGAUGACUUGC......................................................................................................................    50.854203
........................................UAGCCAAGGAUGACUUGCCUGU..................................................................................................................    159.179932
........................................UAGCCAAGGAUGACUUGCC.....................................................................................................................    17.032323
........................................UAGCCAAGGAUGACUUGCCA....................................................................................................................    2.633131
........................................UAGCCAAGGAUGCCUUGCCUG...................................................................................................................    0.754985
........................................UAGCCAAGGAUGACUUGCCUU...................................................................................................................    50.494015
........................................UAGCCAAGGAUGACUUGCCUC...................................................................................................................    6.51412
........................................UAGCCAAGGAUGACUUGU......................................................................................................................    5.4679
........................................UAGCCAAGGAUGACUCGCCUG...................................................................................................................    1.56976
........................................CAGCCAAGGAUGACUUGCCUG...................................................................................................................    94.083396
........................................UAGCCCAGGAUGACUUGCCUG...................................................................................................................    3.40115
........................................UAGCCAAGGAUGACCUGCCUG...................................................................................................................    9.38869
........................................UAGCCAAUGAUGACUUGCCUG...................................................................................................................    1.56976
........................................GAGCCAAGGAUGACUUGCCUG...................................................................................................................    3.12956
........................................UAGCCUAGGAUGACUUGCCUG...................................................................................................................    2.09301
........................................UAGCCAAGGAUGACUUGCCUGA..................................................................................................................    60.555545
........................................UAGCCGAGGAUGACUUGCCUG...................................................................................................................    3.13952
........................................UAGCCAAGGAUGAGUUGCCUG...................................................................................................................    6.2641
........................................UAGCCAAGGAUGACUUUCCUG...................................................................................................................    1.5598
........................................UAGCCAAGGAUGACUUGCU.....................................................................................................................    8.984663
........................................UAGCCAAGGAUGACUUCCCUG...................................................................................................................    1.5598
.........................................AGCCAAGGAUGACUUGCCU....................................................................................................................    25.069596
.........................................AGCCAAGGAUGACUUGCCUG...................................................................................................................    2.860526
.........................................AGCCAAGGAUGACUUGCCGGU..................................................................................................................    0.552991
..........................................GCCAAGGAUGACUUGCCU....................................................................................................................    2.047482
..................................................................................................................CAGGCAGUCUCCUUGGCUAG..........................................    0.797151
...................................................................................................................AGGCAGUCUCCUUGGCUAG..........................................    0.371561
...................................................................................................................AGGCAGUCUCCUUGGCUAGCC........................................    1.39692
...................................................................................................................AGGCAGUCUCCUUGGCUAGC.........................................    113.434077
...................................................................................................................AGGCAGUCUCCUUGGCUAGA.........................................    0.212795
...................................................................................................................AGGCAGUCUCCUUGGCUAGGC........................................    0.318481
....................................................................................................................GGCAGUCUCCUUGGCUAGC.........................................    4934.447462
....................................................................................................................GGCAGUCUCCUUGGCUAG..........................................    61.029283
....................................................................................................................GGCAGUCUCCUUGGCUAGCCC.......................................    162.34696
....................................................................................................................GGCAGUCUCCUUGGCUAGA.........................................    7.31334
....................................................................................................................GGCAGUCUCCUUGGCUAACC........................................    1.245067
....................................................................................................................GGCAGUCUCCUCGGCUAGC.........................................    0.552991
....................................................................................................................GGCAGUCUCCUUGGCUAGCCA.......................................    7.80695
....................................................................................................................GGCAGUCUCCUUGGCUAGGC........................................    2.76017
....................................................................................................................GGCAGUCUCCUUGGCUAGCA........................................    2.92761
....................................................................................................................GGCAGUCUCCUUGGCUAGCU........................................    15.56201
....................................................................................................................GGCAGUCUCUUUGGCUAGC.........................................    0.395191
....................................................................................................................GGCAGUCUCCUUGGCUAAC.........................................    0.424642
.....................................................................................................................GCAGUCUCCUUGGCUAGCC........................................    74.918436
.....................................................................................................................GCAGUCUCCUUGGCUAGCCC.......................................    85.78191
.....................................................................................................................GCAGUCUCCUUGGCUAGC.........................................    21.106916
.....................................................................................................................GCAGUCUCCUUGGCUAGCCA.......................................    5.85522
......................................................................................................................CAGUCUCCUUGGCUAGCCC.......................................    0.796203
......................................................................................................................CAGUCUCCUUGGCUAGCC........................................    1.22084
```

zma-MIR169m

Chromosome: chr3
Start: 102365443
End: 102365598
Strand: +
Strand bias: 1.0
Abundance bias: 0.933
MFE: -73.40

```
                 Mature                                                                            Star
ACGAGGAGCCUAUCUCCCUUAGCCAAGAAUGGCUUGCCUAUCUCCAUUAUUUUGUUCAUCGCAGGAAGCCCUUCCGUGAUGGAUGAAAUGUGGAUGAUGGCAUCCAUUCUUGGCUAAGUGAGUAAUUCUCUUCCACCAUGCUAGGCUUUCCCUCUU    6682.3
..((((((((((.(((.(((((((((((((((..((((.((((((((.((((((((((((((.((((....))))))))))))))))))))))))).))))))).))))))))))))))).)))...................))))))...))))..
.................CUUAGCCAAGAAUGGCUUGCCU.....................................................................................................................    3323.761894
...................................................................................................GCAUCCAUUCUUGGCUAAGUG....................................    2205.077143
................CCUUAGCCAAGAAUGGCUUGCCU.....................................................................................................................    0.477722
.................CUUAGCCAAGAAUGGCUUGCC......................................................................................................................    110.331863
.................CUUAGCCAAGAAUGGCUUGCCA.....................................................................................................................    88.001851
.................CCUAGCCAAGAAUGGCUUGCCU.....................................................................................................................    2.61104
.................CUUAGCCAAGAAUGGCUUGCCC.....................................................................................................................    2.74391
.................CUUAGCCAAGAAUGGCUUGCCG.....................................................................................................................    13.897776
.................CUUAGCCAAGAAUGGCUUUCCU.....................................................................................................................    16.852706
.................CUGAGCCAAGAAUGGCUUGCCU.....................................................................................................................    2.31465
.................CUUAGCCAAGAAUGGCUUGGCU.....................................................................................................................    1.0616
..................UUAGCCAAGAAUGGCUUGCCUA....................................................................................................................    2.519999
..................UUAGCCAAGAAUGGCUUGCCU.....................................................................................................................    19.976499
..................UUAGCCAAGAAUGACUUGCCU.....................................................................................................................    3.20072
...................UAGCCAAGAAUGGCUUGCCUAUC..................................................................................................................    17.31172
................................................................................................AUGGCAUCCAUUCUUGGCUAAGUG....................................    15.60709
.................................................................................................UGGCAUCCAUUCUUGGCUAAGUG....................................    4.09196
.................................................................................................UGGCAUCCAUUCUUGGCUAAG......................................    369.312131
.................................................................................................UGGCAUCCAUUCUUGGCUAA.......................................    1.13918
.................................................................................................UGGCAUCCAUUCUUGGCUAAGU.....................................    2.3397
...................................................................................................GCAUCCAUUCUUGGCUAAG......................................    0.371561
...................................................................................................GCAUCCAUUCUUGGCUAAGU.....................................    4.52818
...................................................................................................GCAUCCAUUCUUGGCUAAGGG....................................    0.849283
...................................................................................................UCAUCCAUUCUUGGCUAAGUG....................................    6.58171
...................................................................................................GCAUCCAUUCUUGGCUAAGUA....................................    3.29805
...................................................................................................GCAUCCAUUCUUGGCUAAGUU....................................    8.634906
....................................................................................................CAUCCAUUCUUGGCUAAGUG....................................    2.18896
....................................................................................................CAUCCAUUCUUGGCUAAGUGA...................................    2.49113
```

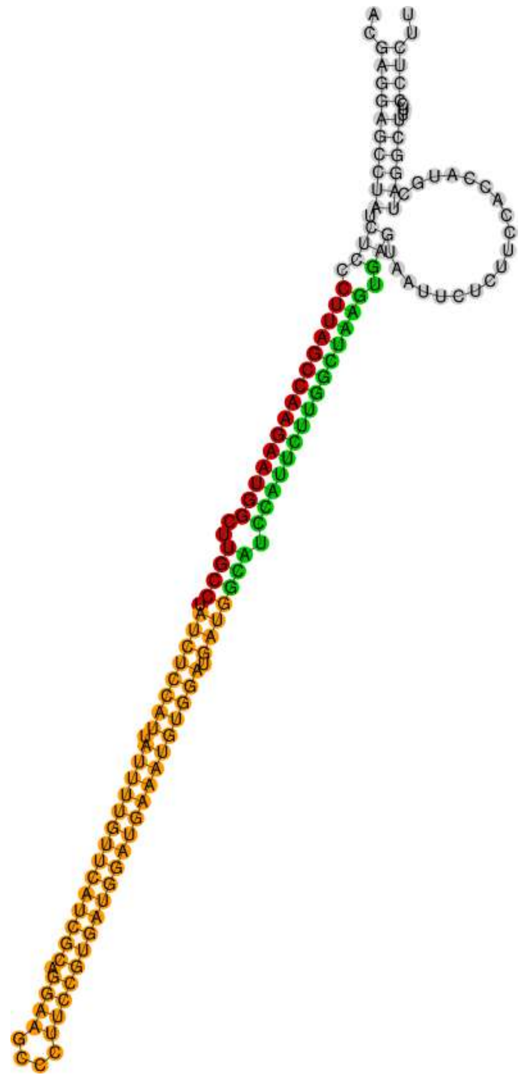

zma-MIR169n

Chromosome: chr3
Start: 233919741
End: 233919896
Strand: +
Strand bias: 1.0
Abundance bias: 1.0
MFE: -73.20

```
                 Mature                                                                         Star
UGGGGGAGCCUAUCUCCCUUAGCCAAGAAUGGCUUGCCUAUCUCCACUGUUUGGUUCGUCUUGGGAGACCACCCUGAUGAUGGGUGUACUGUGUGGAUGAUGGCAGGCCUUCUUGGCUAAGCGAGUGAUUCUCUUCCACCAUGCUAGGCUUGCCCU    3622.7
.(((.(((((((.(((.(((((((((((.((((((((.((((((((..........((((....))))(((((.......)))))......))))).)))))))))))))))))))))))).))).((......))........))))))).))).
.................CUUAGCCAAGAAUGGCUUGCCU.....................................................................................................................    3323.761894
......................................................................................................GCAGGCCUUCUUGGCUAAGCG.................................    8.05898
................CCUUAGCCAAGAAUGGCUUGCCU.....................................................................................................................    0.477722
.................CUUAGCCAAGAAUGGCUUGCC......................................................................................................................    110.331863
.................CUUAGCCAAGAAUGGCUUGCCA.....................................................................................................................    88.001851
.................CCUAGCCAAGAAUGGCUUGCCU.....................................................................................................................    2.61104
.................CUUAGCCAAGAAUGGCUUGCCC.....................................................................................................................    2.74391
.................CUUAGCCAAGAAUGGCUUGCCG.....................................................................................................................    13.897776
.................CUUAGCCAAGAAUGGCUUUCCU.....................................................................................................................    16.852706
.................CUGAGCCAAGAAUGGCUUGCCU.....................................................................................................................    2.31465
.................CUUAGCCAAGAAUGGCUUGGCU.....................................................................................................................    1.0616
..................UUAGCCAAGAAUGGCUUGCCUA....................................................................................................................    2.519999
..................UUAGCCAAGAAUGGCUUGCCU.....................................................................................................................    19.976499
..................UUAGCCAAGAAUGACUUGCCU.....................................................................................................................    3.20072
...................UAGCCAAGAAUGGCUUGCCUAUC..................................................................................................................    17.31172
....................................................................................................UGGCAGGCCUUCUUGGCUAAG...................................    6.29663
.......................................................................................................CAGGCCUUCUUGGCUAAGCG.................................    3.360517
```

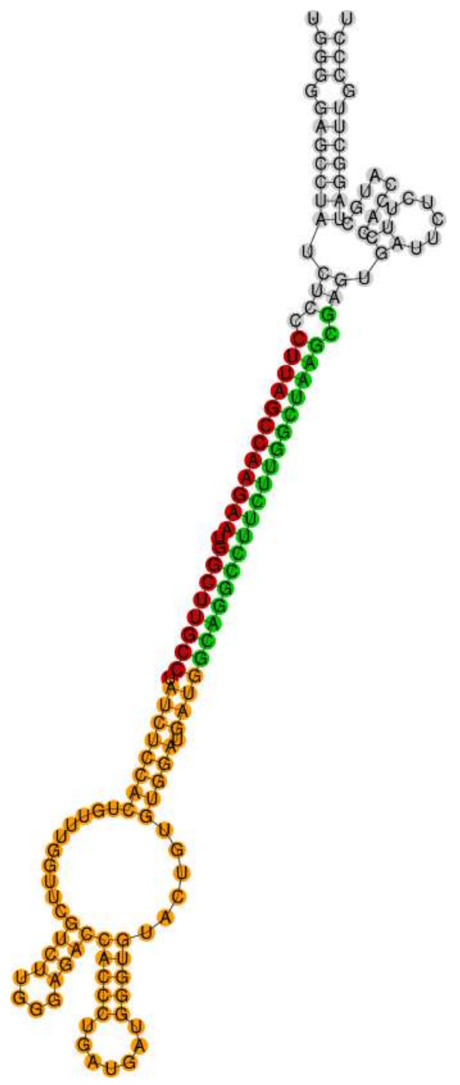

zma-MIR169_N1

Chromosome: chr7
Start: 142353897
End: 142354054
Strand: +
Strand bias: 1.0
Abundance bias: 0.999
MFE: -75.40

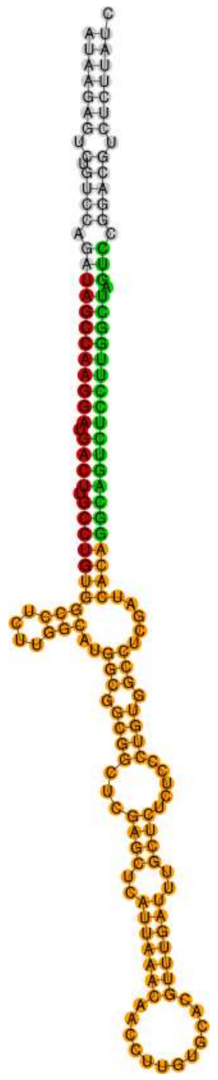

```
                 Mature                                                                                                   Star
AUAAGAGUCUGUCCAGAUAGCCAAGGAUGACUUGCCUGUGGCCUCUUGGCAUGGCGGCGGCUCGAGCUCAUUAAACAACCUUGUGCACGUUUGAUUUGCUCUCUCCCUGUGGCCUCGAUCACAGGCAGUCUCCUUGGCUAGUCCGGACGUCUCUUAUC    4062.0
(((((((.(.((((.((((((((((((.((((.((((((((((....)))..(((.((((...((((..(((((((............)))))))..)))).....)))).))).....)))))))))))))))))))).))).))))).))))))).
.................UAGCCAAGGAUGACUUGCCUG........................................................................................................................    1421.834235
...........................................................................................................................GGCAGUCUCCUUGGCUAGUC...............    500.134793
.................UAGCCAAGGAUGACUUGCCU.........................................................................................................................    1202.958549
.................UAGCCAAGGAUGACUUGC...........................................................................................................................    50.854203
.................UAGCCAAGGAUGACUUGCCUGU.......................................................................................................................    159.179932
.................UAGCCAAGGAUGACUUGCC..........................................................................................................................    17.032323
.................UAGCCAAGGAUGACUUGCCA.........................................................................................................................    2.633131
.................UAGCCAAGGAUGCCUUGCCUG........................................................................................................................    0.754985
.................UAGCCAAGGAUGACUUGCCUU........................................................................................................................    50.494015
.................UAGCCAAGGAUGACUUGCCUC........................................................................................................................    6.51412
.................UAGCCAAGGAUGACUUGU...........................................................................................................................    5.4679
.................UAGCCAAGGAUGACUCGCCUG........................................................................................................................    1.56976
.................CAGCCAAGGAUGACUUGCCUG........................................................................................................................    94.083396
.................UAGCCCAGGAUGACUUGCCUG........................................................................................................................    3.40115
.................UAGCCAAGGAUGACCUGCCUG........................................................................................................................    9.38869
.................UAGCCAAUGAUGACUUGCCUG........................................................................................................................    1.56976
.................GAGCCAAGGAUGACUUGCCUG........................................................................................................................    3.12956
.................UAGCCUAGGAUGACUUGCCUG........................................................................................................................    2.09301
.................UAGCCAAGGAUGACUUGCCUGA.......................................................................................................................    60.555545
.................UAGCCGAGGAUGACUUGCCUG........................................................................................................................    3.13952
.................UAGCCAAGGAUGAGUUGCCUG........................................................................................................................    6.2641
.................UAGCCAAGGAUGACUUUCCUG........................................................................................................................    1.5598
.................UAGCCAAGGAUGACUUGCU..........................................................................................................................    8.984663
.................UAGCCAAGGAUGACUUCCCUG........................................................................................................................    1.5598
..................AGCCAAGGAUGACUUGCCU.........................................................................................................................    25.069596
..................AGCCAAGGAUGACUUGCCUG........................................................................................................................    2.860526
..................AGCCAAGGAUGACUUGCCGGU.......................................................................................................................    0.552991
...................GCCAAGGAUGACUUGCCU.........................................................................................................................    2.047482
........................................................................................................................ACAGGCAGUCUCCUUGGCUAG.................    0.212795
.........................................................................................................................CAGGCAGUCUCCUUGGCUAGU................    4.68033
.........................................................................................................................CAGGCAGUCUCCUUGGCUAG.................    0.797151
.........................................................................................................................CAGGCAGUCUCCUUGGCUAGUU...............    0.318481
..........................................................................................................................AGGCAGUCUCCUUGGCUAG.................    0.371561
..........................................................................................................................AGGCAGUCUCCUUGGCUAGUC...............    25.232044
..........................................................................................................................AGGCAGUCUCCUUGGCUAGU................    48.55055
..........................................................................................................................AGGCAGUCUCCUUGGCUAGA................    0.212795
..........................................................................................................................AGGCAGUCUCCUUGGCUAGGC...............    0.318481
...........................................................................................................................GGCAGUCUCCUUGGCUAGU................    250.874701
...........................................................................................................................GGCAGUCUCCUUGGCUAG.................    61.029283
...........................................................................................................................GGCAGUCUCCUUGGCUAGA................    7.31334
...........................................................................................................................GGCAGUCUCCUUGGCUAAUC...............    1.64549
...........................................................................................................................GGCAGUCUCCUUGGCUAGUCU..............    0.530802
...........................................................................................................................GGCAGUCUCCUUGGCUAGGC...............    2.76017
...........................................................................................................................GGCAGUCUCCUUGGCUAGUU...............    6.688392
```

zma-MIR169_N2

Chromosome: chr4
Start: 49565618
End: 49565794
Strand: +
Strand bias: 1.0
Abundance bias: 0.917
MFE: -80.40

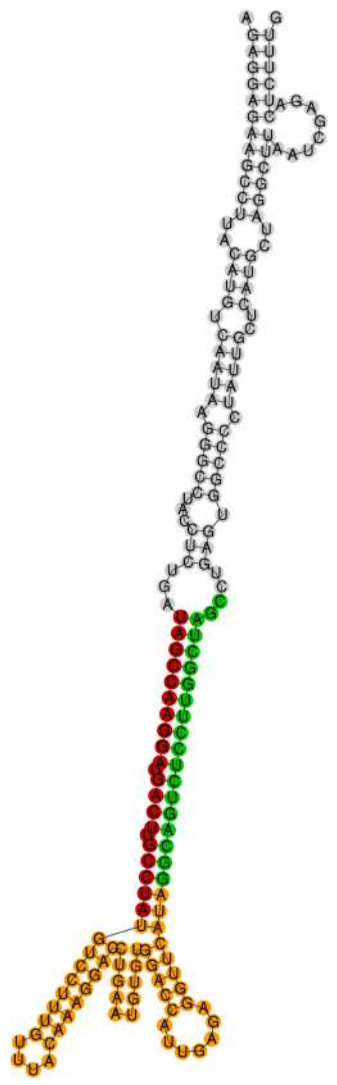

```
                                        Mature                                                                                    Star
AGAGGAGAAGCCUUACAUGUCAAUAAGGGCCUACCUCUGAUAGCCAAGGAUGACUUGCCUAUGUCCUUUGUUUACAAAGGACCUGAAUGUGUGGACCAUUGAGAGGUUCAUAGGCAGUCUCCUUGGCUAGCCUGAGUGGCCCCUAUUGCUCAUGCUAGGCUUAAUCGAGACUCUUUG    10303.3
.((((((((((((..((((.(((((.(((((...(((...(((((((((.((((.(((((((((((((((....))))))))..........(((((.......)))))))))))))))))))))))))....))).))))).)))))..))))..))))))........)))))).
........................................UAGCCAAGGAUGACUUGCCUA....................................................................................................................    1737.40401
................................................................................................................GGCAGUCUCCUUGGCUAGC..............................................    4934.447462
........................................UAGCCAAGGAUGACUUGCCU.....................................................................................................................    1202.958549
........................................UAGCCAAGGAUGACUUGC.......................................................................................................................    50.854203
........................................UAGCCAAGGAUGACUUGCC......................................................................................................................    17.032323
........................................UAGCCAAGGAUGACUUGUCUA....................................................................................................................    1.5598
........................................UAGCCAAGGAUGACUUGCCA.....................................................................................................................    2.633131
........................................UAGCCAAGGAUGACUCGCCUA....................................................................................................................    2.59967
........................................CAGCCAAGGAUGACUUGCCUA....................................................................................................................    15.598
........................................UAGCCAAGGAUGACUUGCCUU....................................................................................................................    50.494015
........................................UAGCCAAGGAUGACUUGCCUC....................................................................................................................    6.51412
........................................UAGCCAAGGAUGACUUGU.......................................................................................................................    5.4679
........................................UAGCCACGGAUGACUUGCCUA....................................................................................................................    1.81977
........................................UAGCCAAGGAUGAUUUGCCUA....................................................................................................................    2.85964
........................................UAGCCAAGGAUGAAUUGCCUA....................................................................................................................    1.81977
........................................UAGCCAAGGAUGACCUGCCUA....................................................................................................................    3.37957
........................................UAUCCAAGGAUGACUUGCCUA....................................................................................................................    3.89951
........................................UAGCUAAGGAUGACUUGCCUA....................................................................................................................    2.07974
........................................UAGCCAAGGAUGACUUGACUA....................................................................................................................    2.3397
........................................UAGCCAAGGAUCACUUGCCUA....................................................................................................................    2.07974
........................................UAGCCAAGGAUGACUUGCAUA....................................................................................................................    2.3397
........................................UAGCCAAGGAUGACUGGCCUA....................................................................................................................    1.5598
........................................UAGCCAAGGAUGACUUGCCGA....................................................................................................................    18.729533
........................................UAGCCAAGGAUUACUUGCCUA....................................................................................................................    1.5598
........................................UAGCCAAGGAUGACUUUCCUA....................................................................................................................    5.19934
........................................UAGCCAAGGAGGACUUGCCUA....................................................................................................................    2.3397
........................................UAGCCAAGGAUGACGUGCCUA....................................................................................................................    2.07974
........................................UAGCCAAGGAUGCCUUGCCUA....................................................................................................................    3.096958
........................................UAGACAAGGAUGACUUGCCUA....................................................................................................................    1.81977
........................................UAGCCAAGGAUGACUUGCU......................................................................................................................    8.984663
........................................UAGCAAAGGAUGACUUGCCUA....................................................................................................................    2.59967
........................................UAGCCAAGGAUGACUUCCCUA....................................................................................................................    2.3397
.........................................AGCCAAGGAUGACUUGCCU.....................................................................................................................    25.069596
..........................................GCCAAGGAUGACUUGCCU.....................................................................................................................    2.047482
..............................................................................................................UAGGCAGUCUCCUUGGCUAGC..............................................    16.781196
...............................................................................................................AGGCAGUCUCCUUGGCUAG...............................................    0.371561
...............................................................................................................AGGCAGUCUCCUUGGCUAGCC.............................................    1.39692
...............................................................................................................AGGCAGUCUCCUUGGCUAGC..............................................    113.434077
...............................................................................................................AGGCAGUCUCCUUGGCUAGA..............................................    0.212795
...............................................................................................................AGGCAGUCUCCUUGGCUAGGC.............................................    0.318481
................................................................................................................GGCAGUCUCCUUGGCUAGCC.............................................    996.802148
................................................................................................................GGCAGUCUCCUUGGCUAG...............................................    61.029283
................................................................................................................GGCAGUCUCCUUGGCUAGCCU............................................    0.477722
................................................................................................................GGCAGUCUCCUUGGCUAGA..............................................    7.31334
................................................................................................................GGCAGUCUCCUUGGCUAGUCU............................................    0.530802
................................................................................................................GGCAGGCUCCUUGGCUAGC..............................................    0.849283
................................................................................................................GGCAGUCUCCUUGGCUAACC.............................................    1.245067
................................................................................................................GGCAGUCUCCUCGGCUAGC..............................................    0.552991
................................................................................................................GGCAGUCUCCUUGGCUAGGC.............................................    2.76017
................................................................................................................GGCAGUCUCCUUGGCUAGCA.............................................    2.92761
................................................................................................................GGCAGUCUCCUUGGCUAGCU.............................................    15.56201
................................................................................................................GGCAGUCUCUUUGGCUAGC..............................................    0.395191
................................................................................................................GGCAGUCUCCUUGGCUAAC..............................................    0.424642
.................................................................................................................GCAGUCUCCUUGGCUAGCCU............................................    1.95174
.................................................................................................................GCAGUCUCCUUGGCUAGCC.............................................    74.918436
.................................................................................................................GCAGUCUCCUUGGCUAGC..............................................    21.106916
..................................................................................................................CAGUCUCCUUGGCUAGCC.............................................    1.22084
```

zma-MIR169o

Chromosome: chr4
Start: 13818162
End: 13818327
Strand: +
Strand bias: 1.0
Abundance bias: 1.0
MFE: -86.10

Mature / Star

```
UGGAGAGCCCCUUUUGGUAGCCAAGAAUGACUUGCCUAUGCACGCCCUCUGUUGGCAGUUCCGUCCGGCAGCCAUGGCGACGGUUGCACAAGGUGAGUUUUUGCGGCGUGGAUGAUGCAAUGUGGCUGCAUCGGCAGGUCUUCUUGGCUAGCCAGUGGGCUCUCGU    1814.9
..((((((((...((((((((((((((.((((((((.((.((((((((((.(((((((..(((((.(.((....)).)))))))))).))).).)))......)))))).))((((((.......))))))))))))))))))))))).))))).))))))))..
.................UAGCCAAGAAUGACUUGCCU.................................................................................................................................    970.434577
.....................................................................................................................................GCAGGUCUUCUUGGCUAGCC.............    194.109097
.................UAGCCAAGAAUGACUUGCCUAU...............................................................................................................................    4.38019
.................UAGCCAAGAAUGACUUGCC..................................................................................................................................    2.579415
.................UAGCCAAGAAUGACUUGC...................................................................................................................................    6.65534
.................UAGCCAAGAAUGACUUGCCUA................................................................................................................................    282.862588
.................UAGCCAAGAAUGACUUGCCA.................................................................................................................................    42.37024
.................UAGCCAAGAAUGACUUGCCC.................................................................................................................................    8.222305
.................UAGCCAAGAAUGACUUGCCG.................................................................................................................................    1.96985
.................UAGCCAAGAAUGACUUGCCUU................................................................................................................................    20.79642
.................UAGCCAAGAAUGACUUGCCCA................................................................................................................................    1.96985
..................AGCCAAGAAUGACUUGCCUA................................................................................................................................    3.735377
..................AGCCAAGAAUGACUUGCCU.................................................................................................................................    1.31324
...................................................................................................................................CGGCAGGUCUUCUUGGCUAG...............    0.17779
...................................................................................................................................CGGCAGGUCUUCUUGGCUAGC..............    45.056286
....................................................................................................................................GGCAGGUCUUCUUGGCUAG...............    5.864483
....................................................................................................................................GGCAGGUCUUCUUGGCUAGCC.............    8.53604
....................................................................................................................................GGCAGGUCUUCUUGGCUAGC..............    33.092885
....................................................................................................................................GGCAGGUCUUCUUGGCUA................    3.4939
.....................................................................................................................................GCAGGUCUUCUUGGCUAGCCA............    0.984927
.....................................................................................................................................GCAGGUCUUCUUGGCUAG...............    4.99241
.....................................................................................................................................GCAGGUCUUCUUGGCUAGC..............    145.91002
.....................................................................................................................................UCAGGUCUUCUUGGCUAGCC.............    1.47739
.....................................................................................................................................GCAGGUCUUCUUGGCUAGCA.............    1.8057
.....................................................................................................................................GCAGGUCUUCUUGGCUAGCCU............    4.549275
.....................................................................................................................................GCAGGUCUUCUUGGCUAGCCC............    0.984927
.....................................................................................................................................GCAGGUCUUCUUGGCUAGA..............    15.42516
......................................................................................................................................CAGGUCUUCUUGGCUAGCC.............    0.482573
......................................................................................................................................CAGGUCUUCUUGGCUAGC..............    0.749285
```

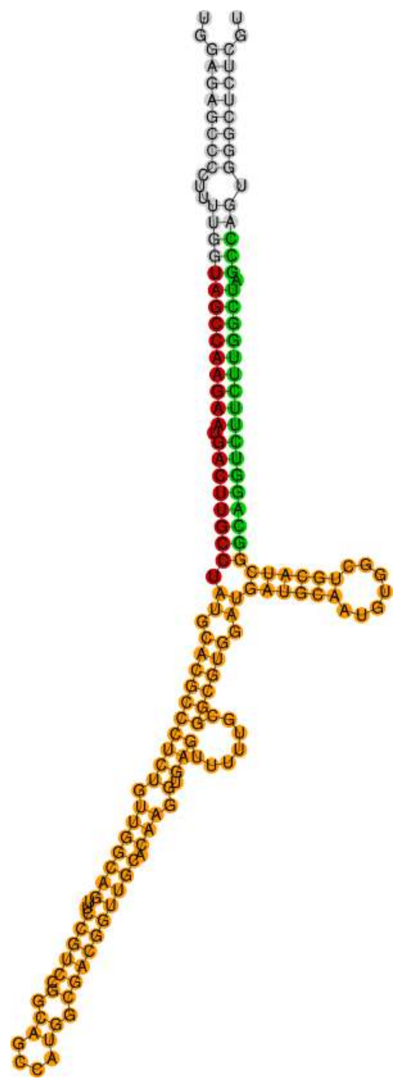

zma-MIR169r

```
Chromosome:    chr9
Start:         111791224
End:           111791425
Strand:        -
Strand bias:   1.0
Abundance bias:0.998
MFE:           -99.60
```

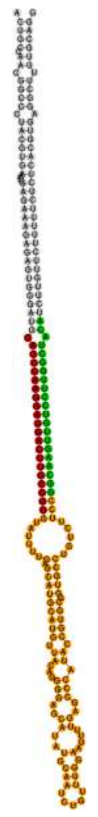

```
                                          Mature                                                                                                          Star
ACUGCCAACGGCCCUACGUGAAGCGAGAAAGAGAGUGGGAUGCAGCCAAGGAUGACUUGCCGGGUAUUUUGAGCAUGCAUGUUACAGGGGAGCAUAUGCAAUCUGUUUGGAAUUUUGAGCCCAUACCGUGCGGUGCCUGUCUUCCGGCAAGUUGUCCUUGGCUACAUCUUGUUCUUUUUCUCUCACGUGAGGCUUUUGCAGG    265100.1
.((((.((.((((.(((((((...(((((((((((((((((.((((((((((((((((((((......(.((((((((.((...(((..((..(.(((.....))).)....))..))).)).))))).))))).....)))))))))))))))))))))).))))))))))))))))))))))))).)))).)))))).
..........................................CAGCCAAGGAUGACUUGCCGG...........................................................................................................................................    247937.35687
.................................................................................................................................................GGCAAGUUGUCCUUGGCUACA....................................    61.783901
........................................UGCAGCCAAGGAUGACUUGCC.............................................................................................................................................    268.374131
..........................................CAGCCAAGGAUGACUUGCCG............................................................................................................................................    473.978624
..........................................CAGCCAAGGAUGACUUGCCGGG..........................................................................................................................................    182.103427
..........................................CAGCCAAGGAUGACUUGC..............................................................................................................................................    72.683654
..........................................CAGCCAAGGAUGACUUGCAGG...........................................................................................................................................    93.848025
..........................................CAGCCAAGGAUGACUCGCCGG...........................................................................................................................................    377.867184
..........................................CAGCCAAGGAUGGCUUGCCGG...........................................................................................................................................    574.818153
..........................................CAGCCAACGAUGACUUGCCGG...........................................................................................................................................    199.179311
..........................................CUGCCAAGGAUGACUUGCCGG...........................................................................................................................................    158.522553
..........................................CAUCCAAGGAUGACUUGCCGG...........................................................................................................................................    374.883267
..........................................CAGCCAAGGAUGACUUUCCGG...........................................................................................................................................    122.35452
..........................................CCGCCAAGGAUGACUUGCCGG...........................................................................................................................................    75.540159
..........................................CAGACAAGGAUGACUUGCCGG...........................................................................................................................................    85.223964
..........................................CAGCCAAGGAUGACUUGCCUG...........................................................................................................................................    94.083396
..........................................CAGCCAAGGAUGCCUUGCCGG...........................................................................................................................................    247.681843
..........................................CAGCCAAGGAUGACUUGCCGGU..........................................................................................................................................    294.662487
..........................................CAGCCAAGGAUGACUUGCCGGA..........................................................................................................................................    125.435506
..........................................CAGCCAAGGAUCACUUGCCGG...........................................................................................................................................    68.161118
..........................................CAGCCAAUGAUGACUUGCCGG...........................................................................................................................................    463.53416
..........................................CACCCAAGGAUGACUUGCCGG...........................................................................................................................................    143.405442
..........................................CAGCCAAGUAUGACUUGCCGG...........................................................................................................................................    66.868994
..........................................CAACCAAGGAUGACUUGCCGG...........................................................................................................................................    261.360798
..........................................CAGCCAAGGAUGACUUGCCAG...........................................................................................................................................    237.1791
..........................................CAGCCAAGGAUGACUUACCGG...........................................................................................................................................    179.22605
..........................................CAGCCACGGAUGACUUGCCGG...........................................................................................................................................    391.935194
..........................................CAGCCAUGGAUGACUUGCCGG...........................................................................................................................................    88.807161
..........................................CAGCCAAAGAUGACUUGCCGG...........................................................................................................................................    95.218492
..........................................CAGCCAAGGAUUACUUGCCGG...........................................................................................................................................    195.977061
..........................................CAGCCAAGGGUGACUUGCCGG...........................................................................................................................................    496.100604
..........................................AAGCCAAGGAUGACUUGCCGG...........................................................................................................................................    185.461084
..........................................CAGCCAAGGAUGACUUGCCGU...........................................................................................................................................    317.296539
..........................................CAGCCAAGGAUGACUUGCCGC...........................................................................................................................................    270.570039
..........................................CAGCUAAGGAUGACUUGCCGG...........................................................................................................................................    203.866094
..........................................CAGCCUAGGAUGACUUGCCGG...........................................................................................................................................    95.555312
..........................................CAGUCAAGGAUGACUUGCCGG...........................................................................................................................................    239.597198
..........................................GAGCCAAGGAUGACUUGCCGG...........................................................................................................................................    124.290936
..........................................CAGCCAAGGAUGUCUUGCCGG...........................................................................................................................................    107.777957
..........................................CAGCCAAGGAUGACUUCCCGG...........................................................................................................................................    121.29565
..........................................CAGCCAAGGAUGAUUUGCCGG...........................................................................................................................................    218.488921
..........................................CAGCCAGGGAUGACUUGCCGG...........................................................................................................................................    487.04577
..........................................CAGCCAAGAAUGACUUGCCGG...........................................................................................................................................    108.850438
..........................................CAGCCAAGGAUGACUUGUCGG...........................................................................................................................................    280.20557
..........................................CAGCCAAGGAUGACUUGCUGG...........................................................................................................................................    282.58343
..........................................CGGCCAAGGAUGACUUGCCGG...........................................................................................................................................    424.57992
..........................................CAGCCGAGGAUGACUUGCCGG...........................................................................................................................................    450.82641
..........................................CAGCCAAGGACGACUUGCCGG...........................................................................................................................................    425.836338
..........................................CAGCCCAGGAUGACUUGCCGG...........................................................................................................................................    74.94026
..........................................CAGCCAAGGAUAACUUGCCGG...........................................................................................................................................    108.585573
..........................................CAGCCAAGGAUGACUUGACGG...........................................................................................................................................    70.403765
..........................................CAGCCAAGGAUGACCUGCCGG...........................................................................................................................................    420.010511
...........................................AGCCAAGGAUGACUUGCCGG...........................................................................................................................................    3626.68943
...........................................AGCCAAGGAUGACUUGCCG............................................................................................................................................    73.961898
...........................................AGCCAAGGAUGACUUGCCGGG..........................................................................................................................................    247.80172
............................................GCCAAGGAUGACUUGCCGG...........................................................................................................................................    76.694391
.............................................CCAAGGAUGACUUGCCGG...........................................................................................................................................    95.567775
.................................................................................................................................................GGCAAGUUGUCCUUGGCUAC.....................................    72.298268
```

zma-MIR171d

Chromosome:	chr9
Start:	128212965
End:	128213076
Strand:	+
Strand bias:	1.0
Abundance bias:	1.0
MFE:	-50.30

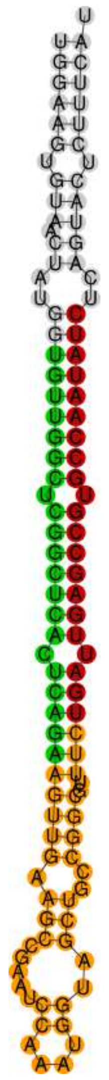

```
                 Star                                                       Mature
UGGAAGUGUAACUAUGGUGUUGGCUCGGCUCACUCAGAAGUUGAAGCCGAAUCCAAAUGGUAGCUGCCGGCGUUUCUGAUUGAGCCGUGCCAAUAUCUCAGUACUCUUUCAU   156773.2
((((((.(((.((..(((((((((.(((((((.((((((((((.(((.....((....))..)))..))))..)))))).))))))).)))))))))..))))).)))))).
............................................................................UGAUUGAGCCGUGCCAAUAUC...............   137861.56804
.................UGUUGGCUCGGCUCACUCAGA..........................................................................   8298.316326
...............................................................................UUGAGCCGUGCCAAUAUC...............   47.74514
..............................................................................AUUGAGCCGUGCCAAUAUC...............   93.864466
.............................................................................GAUUGAGCCGUGCCAAUAUC...............   2898.392626
.............................................................................GAUUGAGCCGUGCCAAUAU................   52.2702
.............................................................................GAUUGAGCCAUGCCAAUAUC...............   7.90804
.............................................................................GAUUGAGCUGUGCCAAUAUC...............   8.48668
............................................................................UGAUUGAGCCGUGCCAAUAU................   519.460044
............................................................................UGAUUGAGCCGUGCCAAU..................   80.757837
............................................................................UGAUUGAGCCGUGCCAAUAUCU..............   148.538991
............................................................................UGAUUGAGCCGUGCCAAUA.................   253.099266
............................................................................UGAUUGAGCCGUGCCAGUAUC...............   52.269209
............................................................................UGAUUGCGCCGUGCCAAUAUC...............   45.54109
............................................................................UGAUUGAGCCGGGCCAAUAUC...............   104.768169
............................................................................UGAUUGAGCCGUGCUAAUAUC...............   61.711192
............................................................................UGAUGGAGCCGUGCCAAUAUC...............   16.57303
............................................................................UGAUUGAGCCGAGCCAAUAUC...............   32.82447
............................................................................UGAUUGAGGCGUGCCAAUAUC...............   19.10187
............................................................................UGAUUGAGCCGUGCCGAUAUC...............   56.489393
............................................................................UGAGUGAGCCGUGCCAAUAUC...............   43.316798
............................................................................UGAUUGAGCCGUGCGAAUAUC...............   7.43492
............................................................................UGAUUGAGCAGUGCCAAUAUC...............   76.172984
............................................................................UGAUUGAGCCGUGUCAAUAUC...............   97.237935
............................................................................UGAUUGAGCCGUACCAAUAUC...............   54.340798
............................................................................UGAUUGAGCCGUUCCAAUAUC...............   111.306088
............................................................................UGAUUGAUCCGUGCCAAUAUC...............   67.193462
............................................................................UGAUUGAGCCGUGCCAAUUU................   32.03288
............................................................................UGAUUGAGCCGUGCCUAUAUC...............   24.070366
............................................................................UGAUUGAGCCGUGCCAAUUUC...............   29.55859
............................................................................UUAUUGAGCCGUGCCAAUAUC...............   111.300891
............................................................................UGAUUGAGCCGUGCCAAUAUG...............   22.035681
............................................................................UGAUUGAGCCCUGCCAAUAUC...............   30.08748
............................................................................UGAUUGAGUCGUGCCAAUAUC...............   178.071637
............................................................................UGAUUGAGCCGUGCCAAUACC...............   100.805743
............................................................................UGAUUGAGCCGUGGCAAUAUC...............   19.08152
............................................................................UGAUUGAGCCGUGCCAAUAUU...............   352.612951
............................................................................UGAUUGAGCCGUGCCAAUAUA...............   157.194939
............................................................................UGAUUGAGCCGUCCCAAUAUC...............   40.289629
............................................................................CGAUUGAGCCGUGCCAAUAUC...............   113.740769
............................................................................UGAUUGAGCCGUGCCAAGAUC...............   18.12679
............................................................................UGAUAGAGCCGUGCCAAUAUC...............   9.49441
............................................................................UGAUUGAGCCGUGCCAAUAUCG..............   9.82041
............................................................................UGAUUGAGCCGUGCCAAUAUCC..............   131.39027
............................................................................UGCUUGAGCCGUGCCAAUAUC...............   103.135059
............................................................................UGAUUGAGCGGUGCCAAUAUC...............   15.51776
............................................................................UGAUUGAACCGUGCCAAUAUC...............   50.866178
............................................................................AGAUUGAGCCGUGCCAAUAUC...............   32.918334
............................................................................GGAUUGAGCCGUGCCAAUAUC...............   126.493245
............................................................................UGAUUAAGCCGUGCCAAUAUC...............   25.060514
............................................................................UCAUUGAGCCGUGCCAAUAUC...............   18.4335
............................................................................UGAUUGAGCCGUGCCAACAUC...............   42.022085
............................................................................UGAUUGAGCUGUGCCAAUAUC...............   96.569206
............................................................................UGAUUGAGCCGUGCCAAUAUUU..............   108.05962
............................................................................UGAUUGAGCCGUGCCAAUGUC...............   40.229137
............................................................................UGAUUGAGCCUUGCCAAUAUC...............   103.005721
............................................................................UGAUUGAGCCGUGCCAAUCUC...............   36.758104
............................................................................UGAUCGAGCCGUGCCAAUAUC...............   52.909318
............................................................................UGAUUGACCCGUGCCAAUAUC...............   65.044061
............................................................................UGAUUGAGCCGUGCCAAUAGC...............   34.9994
............................................................................UAAUUGAGCCGUGCCAAUAUC...............   30.047405
............................................................................UGAUUGAGCCGUGACAAUAUC...............   91.7386
............................................................................UGAAUGAGCCGUGCCAAUAUC...............   6.924
............................................................................UGAUUGAGCCGUGCCAAUC.................   19.98699
............................................................................UGAUUGAGCCGUGCAAAUAUC...............   53.1025
............................................................................UGAUUGAGACGUGCCAAUAUC...............   64.913135
............................................................................UGAUUGAGCCAUGCCAAUAUC...............   62.387571
............................................................................UGGUUGAGCCGUGCCAAUAUC...............   53.74719
............................................................................UGAUUGAGCCGUGCCAAUAUUUC.............   6.61683
............................................................................UGAUUGGGCCGUGCCAAUAUC...............   79.264387
............................................................................UGAUUUAGCCGUGCCAAUAUC...............   75.421776
............................................................................UGAUUGAGCCGUGCCACUAUC...............   89.54748
............................................................................UGAUUCAGCCGUGCCAAUAUC...............   64.859455
............................................................................UGAUUGAGCCGUGCCCAUAUC...............   77.67629
............................................................................UGUUUGAGCCGUGCCAAUAUC...............   7.15019
............................................................................UGAUUGAGCCGUGCCAAUAAC...............   17.84542
............................................................................UGAUUGAGCCGUGCCAUUAUC...............   39.01943
...........................................................................CUGAUUGAGCCGUGCCAAUAUC...............   870.443047
...........................................................................CUGAUUGAGCCGUGCCAAUAU................   30.064876
..........................................................................UCUGAUUGAGCCGUGCCAAUA.................   7.625407
..........................................................................UCUGAUUGAGCCGUGCCAAUAUC...............   132.37052
.........................................................................UUCUGAUUGAGCCGUGCCAAU..................   8.074623
...................UUGGCUCGGCUCACUCAGAAG........................................................................   34.517205
..................GUUGGCUCGGCUCACUCAGA..........................................................................   55.320723
.................UGUUGGCUCGGCUCACUCA............................................................................   6.154042
.................UGUUGGCUCGGCUCACUCAG...........................................................................   366.652574
.................UGUUGGCUCGGCUCACUCAGAA.........................................................................   22.813284
.................UGUUGGCUCGGCUCACUC.............................................................................   513.075429
.................UGUUGGCUCGGCUCACUCAA...........................................................................   23.82091
.................UGUUGGCUCGGCUCACUU.............................................................................   78.73679
.................UGUUGGCUCGGCUCACUA.............................................................................   23.77127
.................UGUUGGCUCGGCUCACCCAGA..........................................................................   6.79201
.................UGUUGGCUCGGCUCCCUCAGA..........................................................................   19.75717
.................UGUUGGCUCGGCUCACUCCGA..........................................................................   10.81431
.................UGUUGGCUCGGCUCACUCAGU..........................................................................   50.896418
.................UGUUGGCUCGGCUCACUCAGG..........................................................................   67.23821
.................UGUUGGCUCGGUUCACUCAGA..........................................................................   5.86341
.................UGUUGGCUCGGCCCACUCAGA..........................................................................   5.827095
```

zma-MIR171e

Chromosome: chr1
Start: 7976717
End: 7976833
Strand: +
Strand bias: 1.0
Abundance bias: 1.0
MFE: -58.20

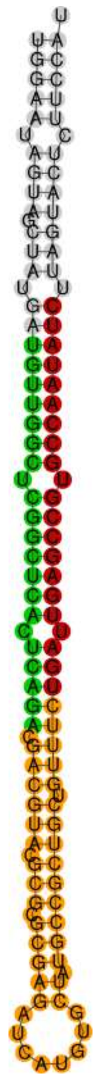

```
                 Star                                                      Mature
UGGAAUAGUAGCUAUGAUGUUGGCUCGGCUCACUCAGACGACGUACGCGCGAGAUCAUGUGCUAUGCCGCUGCUGUUUCUGAUUGAGCCGUGCCAAUAUCUUAGUACUCUUCCAU    158547.
(((((.((((.(((.(((((((((.(((((((.(((((.((((((.(((.(((((........)).))))))))).)))))))).))))))).))))))))).))))))).))))).
.................................................................................UGAUUGAGCCGUGCCAAUAUC...............    137861.56804
.................UGUUGGCUCGGCUCACUCAGA...............................................................................    8298.316326
....................................................................................UUGAGCCGUGCCAAUAUC...............    47.74514
...................................................................................AUUGAGCCGUGCCAAUAUC...............    93.864466
..................................................................................GAUUGAGCCGUGCCAAUAUC...............    2898.392626
..................................................................................GAUUGAGCCGUGCCAAUAU................    52.2702
..................................................................................GAUUGAGCCAUGCCAAUAUC...............    7.90804
..................................................................................GAUUGAGCUGUGCCAAUAUC...............    8.48668
.................................................................................UGAUUGAGCCGUGCCAAUAU................    519.460044
.................................................................................UGAUUGAGCCGUGCCAAU..................    80.757837
.................................................................................UGAUUGAGCCGUGCCAAUAUCU..............    148.538991
.................................................................................UGAUUGAGCCGUGCCAAUAUCUU.............    16.51509
.................................................................................UGAUUGAGCCGUGCCAAUA.................    253.099266
.................................................................................UGAUUGAGCCGUGCCAGUAUC...............    52.269209
.................................................................................UGAUUGCGCCGUGCCAAUAUC...............    45.54109
.................................................................................UGAUUGAGCCGGGCCAAUAUC...............    104.768169
.................................................................................UGAUUGAGCCGUGCUAAUAUC...............    61.711192
.................................................................................UGAUGGAGCCGUGCCAAUAUC...............    16.57303
.................................................................................UGAUUGAGCCGAGCCAAUAUC...............    32.82447
.................................................................................UGAUUGAGGCGUGCCAAUAUC...............    19.10187
.................................................................................UGAUUGAGCCGUGCCGAUAUC...............    56.489393
.................................................................................UGAGUGAGCCGUGCCAAUAUC...............    43.316798
.................................................................................UGAUUGAGCCGUGCGAAUAUC...............    7.43492
.................................................................................UGAUUGAGCAGUGCCAAUAUC...............    76.172984
.................................................................................UGAUUGAGCCGUGUCAAUAUC...............    97.237935
.................................................................................UGAUUGAGCCGUACCAAUAUC...............    54.340798
.................................................................................UGAUUGAGCCGUUCCAAUAUC...............    111.306088
.................................................................................UGAUUGAUCCGUGCCAAUAUC...............    67.193462
.................................................................................UGAUUGAGCCGUGCCAAUUU................    32.03288
.................................................................................UGAUUGAGCCGUGCCUAUAUC...............    24.070366
.................................................................................UGAUUGAGCCGUGCCAAUUUC...............    29.55859
.................................................................................UUAUUGAGCCGUGCCAAUAUC...............    111.300891
.................................................................................UGAUUGAGCCGUGCCAAUAUG...............    22.035681
.................................................................................UGAUUGAGCCCUGCCAAUAUC...............    30.08748
.................................................................................UGAUUGAGUCGUGCCAAUAUC...............    178.071637
.................................................................................UGAUUGAGCCGUGCCAAUACC...............    100.805743
.................................................................................UGAUUGAGCCGUGGCAAUAUC...............    19.08152
.................................................................................UGAUUGAGCCGUGCCAAUAUU...............    352.612951
.................................................................................UGAUUGAGCCGUGCCAAUAUA...............    157.194939
.................................................................................UGAUUGAGCCGUCCCAAUAUC...............    40.289629
.................................................................................CGAUUGAGCCGUGCCAAUAUC...............    113.740769
.................................................................................UGAUUGAGCCGUGCCAAGAUC...............    18.12679
.................................................................................UGAUAGAGCCGUGCCAAUAUC...............    9.49441
.................................................................................UGAUUGAGCCGUGCCAAUAUCG..............    9.82041
.................................................................................UGAUUGAGCCGUGCCAAUAUCC..............    131.39027
.................................................................................UGCUUGAGCCGUGCCAAUAUC...............    103.135059
.................................................................................UGAUUGAGCGGUGCCAAUAUC...............    15.51776
.................................................................................UGAUUGAACCGUGCCAAUAUC...............    50.866178
.................................................................................AGAUUGAGCCGUGCCAAUAUC...............    32.918334
.................................................................................GGAUUGAGCCGUGCCAAUAUC...............    126.493245
.................................................................................UGAUUAAGCCGUGCCAAUAUC...............    25.060514
.................................................................................UCAUUGAGCCGUGCCAAUAUC...............    18.4335
.................................................................................UGAUUGAGCCGUGCCAACAUC...............    42.022085
.................................................................................UGAUUGAGCUGUGCCAAUAUC...............    96.569206
.................................................................................UGAUUGAGCCGUGCCAAUAUUU..............    108.05962
.................................................................................UGAUUGAGCCGUGCCAAUGUC...............    40.229137
.................................................................................UGAUUGAGCCUUGCCAAUAUC...............    103.005721
.................................................................................UGAUUGAGCCGUGCCAAUCUC...............    36.758104
.................................................................................UGAUCGAGCCGUGCCAAUAUC...............    52.909318
.................................................................................UGAUUGACCCGUGCCAAUAUC...............    65.044061
.................................................................................UGAUUGAGCCGUGCCAAUAGC...............    34.9994
.................................................................................UAAUUGAGCCGUGCCAAUAUC...............    30.047405
.................................................................................UGAUUGAGCCGUGACAAUAUC...............    91.7386
.................................................................................UGAUUGAGCCGUGCCAAUC.................    19.98699
.................................................................................UGAUUGAGCCGUGCAAAUAUC...............    53.1025
.................................................................................UGAUUGAGACGUGCCAAUAUC...............    64.913135
.................................................................................UGAUUGAGCCAUGCCAAUAUC...............    62.387571
.................................................................................UGGUUGAGCCGUGCCAAUAUC...............    53.74719
.................................................................................UGAUUGAGCCGUGCCAAUAUUUU.............    50.18167
.................................................................................UGAUUGGGCCGUGCCAAUAUC...............    79.264387
.................................................................................UGAUUUAGCCGUGCCAAUAUC...............    75.421776
.................................................................................UGAUUGAGCCGUGCCACUAUC...............    89.54748
.................................................................................UGAUUCAGCCGUGCCAAUAUC...............    64.859455
.................................................................................UGAUUGAGCCGUGCCCAUAUC...............    77.67629
.................................................................................UGUUUGAGCCGUGCCAAUAUC...............    7.15019
.................................................................................UGAUUGAGCCGUGCCAAUAAC...............    17.84542
.................................................................................UGAUUGAGCCGUGCCAUUAUC...............    39.01943
................................................................................CUGAUUGAGCCGUGCCAAUAUC...............    870.443047
................................................................................CUGAUUGAGCCGUGCCAAUAU................    30.064876
...............................................................................UCUGAUUGAGCCGUGCCAAUA.................    7.625407
...............................................................................UCUGAUUGAGCCGUGCCAAUAUC...............    132.37052
..............................................................................UUCUGAUUGAGCCGUGCCAAU..................    8.074623
...................UUGGCUCGGCUCACUCAGACG.............................................................................    1269.414028
..................GUUGGCUCGGCUCACUCAGA...............................................................................    55.320723
..................GUUGGCUCGGCUCACUCAGACG.............................................................................    8.9264
..................GUUGGCUCGGCUCACUCAGAC..............................................................................    361.383142
.................UGUUGGCUCGGCUCACUCAG................................................................................    366.652574
.................UGUUGGCUCGGCUCACUCAGAC..............................................................................    97.418848
.................UGUUGGCUCGGCUCACUC..................................................................................    513.075429
.................UGUUGGCUCGGCUCACUCAGACGA............................................................................    19.913582
.................UGUUGGCUCGGCUCACUCAGACG.............................................................................    18.029078
.................UGUUGGCUCGGCUCACUCAA................................................................................    23.82091
.................UGUUGGCUCGGCUCACUU..................................................................................    78.73679
.................UGUUGGCUCGGCUCACUA..................................................................................    23.77127
.................UGUUGGCUCGGCUCCCUCAGA...............................................................................    19.75717
.................UGUUGGCUCGGCUCACUCCGA...............................................................................    10.81431
.................UGUUGGCUCGGCUCACUCAGU...............................................................................    50.896418
.................UGUUGGCUCGGCUCACUCAGG...............................................................................    67.23821
```

zma-MIR171f

Chromosome: chr5
Start: 8896286
End: 8896429
Strand: -
Strand bias: 1.0
Abundance bias: 1.0
MFE: -71.00

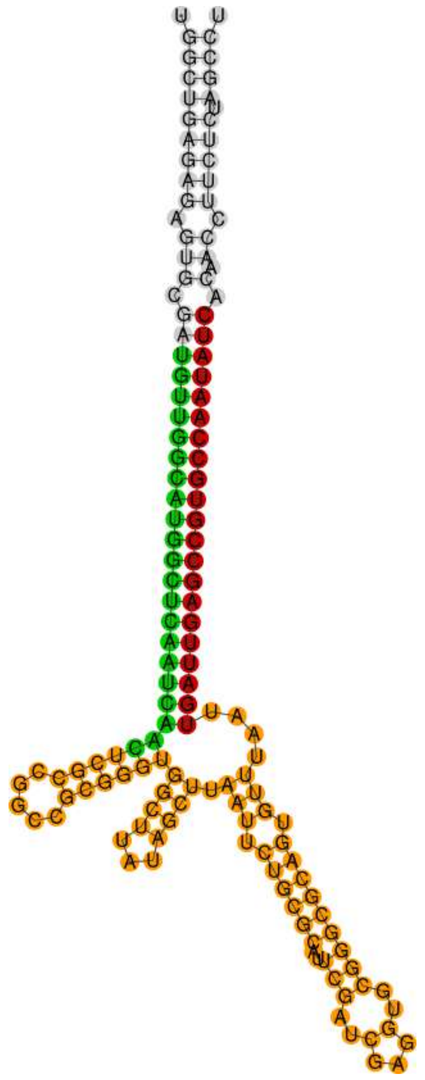

```
                 Star                                                                                      Mature
UGGCUGAGAGAGUGCGAUGUUGGCAUGGCUCAAUCAACUCGCCGGCCGCGGGUGGCUUAUAGCUUAAUUCUGCGCAUUCGAUCGAGGUGCGGGCGCAGUGUUUAAUUGAUUGAGCCGUGCCAAUAUCACAACCUUCUCUAGCCU   146230.2
.((((((((.(((.((((((((((((((((((((((((((((.....))))))((((...)))).(((.((((((..(((..(...)..))))))))).)))....))))))))))))))))))))).).)).))))).)))).
..........................................................................................................UGAUUGAGCCGUGCCAAUAUC.................   137861.56804
.................UGUUGGCAUGGCUCAAUCAAC..........................................................................................................   229.962788
.............................................................................................................UUGAGCCGUGCCAAUAUCAC...............   7.81576
.............................................................................................................UUGAGCCGUGCCAAUAUC.................   47.74514
.............................................................................................................UUGAGCCGUGCCAAUAUCACA..............   9.50334
............................................................................................................AUUGAGCCGUGCCAAUAUC.................   93.864466
...........................................................................................................GAUUGAGCCGUGCCAAUA...................   3.85758
...........................................................................................................GAUUGAGCCGUGCCAAUAUC.................   2898.392626
...........................................................................................................GAUUGAGCCGUGCCAAUAU..................   52.2702
...........................................................................................................GAUUGAGCCGUGCUAAUAUC.................   2.50743
...........................................................................................................GAUUGAGCCGUGUCAAUAUC.................   5.59349
...........................................................................................................GAUUGAGCCCUGCCAAUAUC.................   5.40062
...........................................................................................................GAUUGAGCCGUACCAAUAUC.................   2.50743
...........................................................................................................GAUUGAGCCGUGCCUAUAUC.................   3.27895
...........................................................................................................GAUUGAGUCGUGCCAAUAUC.................   5.78637
...........................................................................................................GAUUGAGCCGUGCCCAUAUC.................   3.47182
...........................................................................................................GAUUGAGCCUUGCCAAUAUC.................   4.24334
...........................................................................................................GAUUGAGCCGUGCCAAUAUU.................   4.82198
...........................................................................................................GAUUGAGCCGUGCCAAUAUA.................   2.12167
...........................................................................................................GAUUGAGCCGUGCCAAAAUC.................   3.85758
...........................................................................................................GAUUGAGCCAUGCCAAUAUC.................   7.90804
...........................................................................................................GAUUGAGCCGUGCCAACAUC.................   3.85758
...........................................................................................................GAUUGAGCCGUGCCAGUAUC.................   2.50743
...........................................................................................................GAUUGAGCUGUGCCAAUAUC.................   8.48668
...........................................................................................................GAUUGAGCCGUGCCGAUAUC.................   5.01486
...........................................................................................................GAUUGAGCCGUGCCAAUACC.................   2.70031
..........................................................................................................UGAUUGAGCCGUGCCAAUAU..................   519.460044
..........................................................................................................UGAUUGAGCCGUGCCAAU....................   80.757837
..........................................................................................................UGAUUGAGCCGUGCCAAUAUCA................   131.412047
..........................................................................................................UGAUUGAGCCGUGCCAAUA...................   253.099266
..........................................................................................................UGAUUGAGCCGUGCCAGUAUC.................   52.269209
..........................................................................................................UGAUUGCGCCGUGCCAAUAUC.................   45.54109
..........................................................................................................UGAUUGAGCCGGGCCAAUAUC.................   104.768169
..........................................................................................................UGAUUGAGCCGUGCUAAUAUC.................   61.711192
..........................................................................................................UGAUGGAGCCGUGCCAAUAUC.................   16.57303
..........................................................................................................UGAUUGAGCCGAGCCAAUAUC.................   32.82447
..........................................................................................................UGAUUGAGGCGUGCCAAUAUC.................   19.10187
..........................................................................................................UGAUUGAGCCGUGCCGAUAUC.................   56.489393
..........................................................................................................UGAGUGAGCCGUGCCAAUAUC.................   43.316798
..........................................................................................................UGAUUGAGCCGUGCGAAUAUC.................   7.43492
..........................................................................................................UGAUUGAGCAGUGCCAAUAUC.................   76.172984
..........................................................................................................UGAUUGAGCCGUGUCAAUAUC.................   97.237935
..........................................................................................................UGAUUGAGCCGUACCAAUAUC.................   54.340798
..........................................................................................................UGAUUGAGCCGUUCCAAUAUC.................   111.306088
..........................................................................................................UGAUUGAUCCGUGCCAAUAUC.................   67.193462
..........................................................................................................UGAUUGAGCCGUGCCAAUUU..................   32.03288
..........................................................................................................UGAUUGAGCCGUGCCUAUAUC.................   24.070366
..........................................................................................................UGAUUGAGCCGUGCCAAUUUC.................   29.55859
..........................................................................................................UUAUUGAGCCGUGCCAAUAUC.................   111.300891
..........................................................................................................UGAUUGAGCCGUGCCAAUAUG.................   22.035681
..........................................................................................................UGAUUGAGCCCUGCCAAUAUC.................   30.08748
..........................................................................................................UGAUUGAGUCGUGCCAAUAUC.................   178.071637
..........................................................................................................UGAUUGAGCCGUGCCAAUACC.................   100.805743
..........................................................................................................UGAUUGAGCCGUGGCAAUAUC.................   19.08152
..........................................................................................................UGAUUGAGCCGUGCCAAUAUU.................   352.612951
..........................................................................................................UGAUUGAGCCGUGCCAAUAUA.................   157.194939
..........................................................................................................UGAUUGAGCCGUCCCAAUAUC.................   40.289629
..........................................................................................................CGAUUGAGCCGUGCCAAUAUC.................   113.740769
..........................................................................................................UGAUUGAGCCGUGCCAAGAUC.................   18.12679
..........................................................................................................UGAUAGAGCCGUGCCAAUAUC.................   9.49441
..........................................................................................................UGAUUGAGCCGUGCCAAUAUCG................   9.82041
..........................................................................................................UGAUUGAGCCGUGCCAAUAUCC................   131.39027
..........................................................................................................UGCUUGAGCCGUGCCAAUAUC.................   103.135059
..........................................................................................................UGAUUGAGCGGUGCCAAUAUC.................   15.51776
..........................................................................................................UGAUUGAACCGUGCCAAUAUC.................   50.866178
..........................................................................................................AGAUUGAGCCGUGCCAAUAUC.................   32.918334
..........................................................................................................GGAUUGAGCCGUGCCAAUAUC.................   126.493245
..........................................................................................................UGAUUAAGCCGUGCCAAUAUC.................   25.060514
..........................................................................................................UCAUUGAGCCGUGCCAAUAUC.................   18.4335
..........................................................................................................UGAUUGAGCCGUGCCAACAUC.................   42.022085
..........................................................................................................UGAUUGAGCUGUGCCAAUAUC.................   96.569206
..........................................................................................................UGAUUGAGCCGUGCCAAUGUC.................   40.229137
..........................................................................................................UGAUUGAGCCUUGCCAAUAUC.................   103.005721
..........................................................................................................UGAUUGAGCCGUGCCAAAAUC.................   3.67141
..........................................................................................................UGAUUGAGCCGUGCCAAUCUC.................   36.758104
..........................................................................................................UGAUCGAGCCGUGCCAAUAUC.................   52.909318
..........................................................................................................UGAUUGACCCGUGCCAAUAUC.................   65.044061
..........................................................................................................UGAUUGAGCCGUGCCAAUAGC.................   34.9994
..........................................................................................................UAAUUGAGCCGUGCCAAUAUC.................   30.047405
..........................................................................................................UGAUUGAGCCGUGACAAUAUC.................   91.7386
..........................................................................................................UGAAUGAGCCGUGCCAAUAUC.................   6.924
..........................................................................................................UGAUUGAGCCGUGCCAAUC...................   19.98699
..........................................................................................................UGAUUGAGCCGUGCAAAUAUC.................   53.1025
..........................................................................................................UGAUUGAGACGUGCCAAUAUC.................   64.913135
..........................................................................................................UGAUUGAGCCAUGCCAAUAUC.................   62.387571
..........................................................................................................UGAUUGUGCCGUGCCAAUAUC.................   5.16365
..........................................................................................................UGGUUGAGCCGUGCCAAUAUC.................   53.74719
..........................................................................................................UGAUUGAGCCGUGCCAAUACCA................   22.06487
..........................................................................................................UGAUUGGGCCGUGCCAAUAUC.................   79.264387
..........................................................................................................UGAUUUAGCCGUGCCAAUAUC.................   75.421776
..........................................................................................................UGAUUGAGCCGUGCCACUAUC.................   89.54748
..........................................................................................................UGAUUCAGCCGUGCCAAUAUC.................   64.859455
..........................................................................................................UGAUUGAGCCGUGCCCAUAUC.................   77.67629
..........................................................................................................UGUUUGAGCCGUGCCAAUAUC.................   7.15019
..........................................................................................................UGAUUGAGCCGUGCCAAUAAC.................   17.84542
..........................................................................................................UGAUUGAGCCGUGCCAUUAUC.................   39.01943
.........................................................................................................UUGAUUGAGCCGUGCCAAUAUC.................   17.09109
........................................................................................................AUUGAUUGAGCCGUGCCAAUAUC.................   6.62952
```

```
                           Star                                                        Mature
AAGAACGAGGCGACAUGGCAUGGUAUUGACUUGGCUCAUCUCUCGCCCACACCAGAAUCCACAAGUGCCGGAGGAGGUGAGCCGAGCCAAUAUCACUUCAUGUCAUCUAGCGUUCU    752.6
.(((((((((.(((((((..((((((((.(((((((((((((.(.((..(((..(......)..)))..)).)))))))))))))).))))))))..))))))).)))..))))))
.............................................................................UGAGCCGAGCCAAUAUCACUUC.................    387.142491
..................CAUGGUAUUGACUUGGCUCAUC............................................................................    165.030548
.............................................................................UGAGCCGAGCCAAUAUCACU...................    21.72858
.............................................................................UGAGCCGAGCCAAUAUCACUU..................    117.007713
.............................................................................UGAGCCGAACCAAUAUCACUU..................    7.72458
.............................................................................UGAGCCGAGCCAAUAUCACUUU.................    2.44225
...................AUGGUAUUGACUUGGCUCAUC............................................................................    51.60041
```

zma-MIR171g

```
Chromosome:    chr7
Start:         44057811
End:           44057926
Strand:        -
Strand bias:   0.994
Abundance bias:1.0
MFE:           -54.10
```

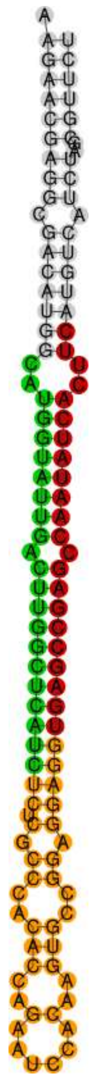

zma-MIR171i

Chromosome: chr1
Start: 278093497
End: 278093593
Strand: +
Strand bias: 1.0
Abundance bias:1.0
MFE: -53.40

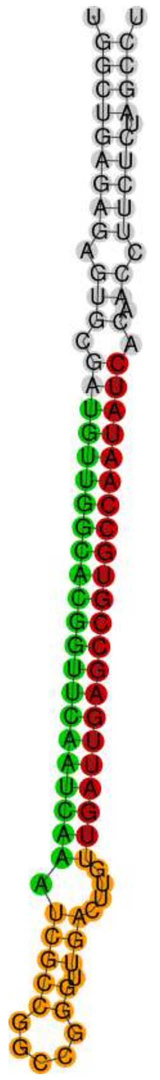

Star / Mature

```
UGGCUGAGAGAGUGCGAUGUUGGCACGGUUCAAUCAAAUCGCCGGCCGGGUUGACUUGUUGAUUGAGCCGUGCCAAUAUCACAACCUUCUCUAGCCU    146378.5
.(((((((((.(((.((((((((((((((((((((((.(((((.....)).)))....)))))))))))))))))))))).).)).))))).)))).
...........................................................UGAUUGAGCCGUGCCAAUAUC.................    137861.56804
.................UGUUGGCACGGUUCAAUCAAA...........................................................    361.04274
..............................................................UUGAGCCGUGCCAAUAUCAC...............    7.81576
..............................................................UUGAGCCGUGCCAAUAUC.................    47.74514
..............................................................UUGAGCCGUGCCAAUAUCACA..............    9.50334
.............................................................AUUGAGCCGUGCCAAUAUC.................    93.864466
............................................................GAUUGAGCCGUGCCAAUA...................    3.85758
............................................................GAUUGAGCCGUGCCAAUAUC.................    2898.392626
............................................................GAUUGAGCCGUGCCAAUAU..................    52.2702
............................................................GAUUGAGCCGUGCUAAUAUC.................    2.50743
............................................................GAUUGAGCCGUGUCAAUAUC.................    5.59349
............................................................GAUUGAGCCCUGCCAAUAUC.................    5.40062
............................................................GAUUGAGCCGUACCAAUAUC.................    2.50743
............................................................GAUUGAGCCGUGCCUAUAUC.................    3.27895
............................................................GAUUGAGUCGUGCCAAUAUC.................    5.78637
............................................................GAUUGAGCCGUGCCCAUAUC.................    3.47182
............................................................GAUUGAGCCUUGCCAAUAUC.................    4.24334
............................................................GAUUGAGCCGUGCCAAUAUU.................    4.82198
............................................................GAUUGAGCCGUGCCAAAAUC.................    3.85758
............................................................GAUUGAGCCAUGCCAAUAUC.................    7.90804
............................................................GAUUGAGCCGUGCCAACAUC.................    3.85758
............................................................GAUUGAGCCGUGCCAGUAUC.................    2.50743
............................................................GAUUGAGCUGUGCCAAUAUC.................    8.48668
............................................................GAUUGAGCCGUGCCGAUAUC.................    5.01486
............................................................GAUUGAGCCGUGCCAAUACC.................    2.70031
...........................................................UGAUUGAGCCGUGCCAAUAU..................    519.460044
...........................................................UGAUUGAGCCGUGCCAAU....................    80.757837
...........................................................UGAUUGAGCCGUGCCAAUAUCA................    131.412047
...........................................................UGAUUGAGCCGUGCCAAUA...................    253.099266
...........................................................UGAUUGAGCCGUGCCAGUAUC.................    52.269209
...........................................................UGAUUGCGCCGUGCCAAUAUC.................    45.54109
...........................................................UGAUUGAGCCGGGCCAAUAUC.................    104.768169
...........................................................UGAUUGAGCCGUGCUAAUAUC.................    61.711192
...........................................................UGAUGGAGCCGUGCCAAUAUC.................    16.57303
...........................................................UGAUUGAGCCGAGCCAAUAUC.................    32.82447
...........................................................UGAUUGAGGCGUGCCAAUAUC.................    19.10187
...........................................................UGAUUGAGCCGUGCCGAUAUC.................    56.489393
...........................................................UGAGUGAGCCGUGCCAAUAUC.................    43.316798
...........................................................UGAUUGAGCCGUGCGAAUAUC.................    7.43492
...........................................................UGAUUGAGCAGUGCCAAUAUC.................    76.172984
...........................................................UGAUUGAGCCGUGUCAAUAUC.................    97.237935
...........................................................UGAUUGAGCCGUACCAAUAUC.................    54.340798
...........................................................UGAUUGAGCCGUUCCAAUAUC.................    111.306088
...........................................................UGAUUGAUCCGUGCCAAUAUC.................    67.193462
...........................................................UGAUUGAGCCGUGCCAAUUU..................    32.03288
...........................................................UGAUUGAGCCGUGCCUAUAUC.................    24.070366
...........................................................UGAUUGAGCCGUGCCAAUUUC.................    29.55859
...........................................................UUAUUGAGCCGUGCCAAUAUC.................    111.300891
...........................................................UGAUUGAGCCGUGCCAAUAUG.................    22.035681
...........................................................UGAUUGAGCCCUGCCAAUAUC.................    30.08748
...........................................................UGAUUGAGUCGUGCCAAUAUC.................    178.071637
...........................................................UGAUUGAGCCGUGCCAAUACC.................    100.805743
...........................................................UGAUUGAGCCGUGGCAAUAUC.................    19.08152
...........................................................UGAUUGAGCCGUGCCAAUAUU.................    352.612951
...........................................................UGAUUGAGCCGUGCCAAUAUA.................    157.194939
...........................................................UGAUUGAGCCGUCCCAAUAUC.................    40.289629
...........................................................CGAUUGAGCCGUGCCAAUAUC.................    113.740769
...........................................................UGAUUGAGCCGUGCCAAGAUC.................    18.12679
...........................................................UGAUAGAGCCGUGCCAAUAUC.................    9.49441
...........................................................UGAUUGAGCCGUGCCAAUAUCG................    9.82041
...........................................................UGAUUGAGCCGUGCCAAUAUCC................    131.39027
...........................................................UGCUUGAGCCGUGCCAAUAUC.................    103.135059
...........................................................UGAUUGAGCGGUGCCAAUAUC.................    15.51776
...........................................................UGAUUGAACCGUGCCAAUAUC.................    50.866178
...........................................................AGAUUGAGCCGUGCCAAUAUC.................    32.918334
...........................................................GGAUUGAGCCGUGCCAAUAUC.................    126.493245
...........................................................UGAUUAAGCCGUGCCAAUAUC.................    25.060514
...........................................................UCAUUGAGCCGUGCCAAUAUC.................    18.4335
...........................................................UGAUUGAGCCGUGCCAACAUC.................    42.022085
...........................................................UGAUUGAGCUGUGCCAAUAUC.................    96.569206
...........................................................UGAUUGAGCCGUGCCAAUGUC.................    40.229137
...........................................................UGAUUGAGCCUUGCCAAUAUC.................    103.005721
...........................................................UGAUUGAGCCGUGCCAAAAUC.................    3.67141
...........................................................UGAUUGAGCCGUGCCAAUCUC.................    36.758104
...........................................................UGAUCGAGCCGUGCCAAUAUC.................    52.909318
...........................................................UGAUUGACCCGUGCCAAUAUC.................    65.044061
...........................................................UGAUUGAGCCGUGCCAAUAGC.................    34.9994
...........................................................UAAUUGAGCCGUGCCAAUAUC.................    30.047405
...........................................................UGAUUGAGCCGUGACAAUAUC.................    91.7386
...........................................................UGAAUGAGCCGUGCCAAUAUC.................    6.924
...........................................................UGAUUGAGCCGUGCCAAUC...................    19.98699
...........................................................UGAUUGAGCCGUGCAAAUAUC.................    53.1025
...........................................................UGAUUGAGACGUGCCAAUAUC.................    64.913135
...........................................................UGAUUGAGCCAUGCCAAUAUC.................    62.387571
...........................................................UGAUUGUGCCGUGCCAAUAUC.................    5.16365
...........................................................UGGUUGAGCCGUGCCAAUAUC.................    53.74719
...........................................................UGAUUGAGCCGUGCCAAUACCA................    22.06487
...........................................................UGAUUGGGCCGUGCCAAUAUC.................    79.264387
...........................................................UGAUUUAGCCGUGCCAAUAUC.................    75.421776
...........................................................UGAUUGAGCCGUGCCACUAUC.................    89.54748
...........................................................UGAUUCAGCCGUGCCAAUAUC.................    64.859455
...........................................................UGAUUGAGCCGUGCCCAUAUC.................    77.67629
...........................................................UGUUUGAGCCGUGCCAAUAUC.................    7.15019
...........................................................UGAUUGAGCCGUGCCAAUAAC.................    17.84542
...........................................................UGAUUGAGCCGUGCCAUUAUC.................    39.01943
..........................................................UUGAUUGAGCCGUGCCAAUAUC.................    17.09109
.........................................................GUUGAUUGAGCCGUGCCAAUAUC.................    2.72155
........................................................UGUUGAUUGAGCCGUGCCAAUAUC.................    3.66277
.................UGUUGGCACGGUUCAAUCAA............................................................    19.04832
```

zma-MIR171l

```
Chromosome:    chr1
Start:         249547352
End:           249547472
Strand:        +
Strand bias:   1.0
Abundance bias:0.992
MFE:           -75.30
```

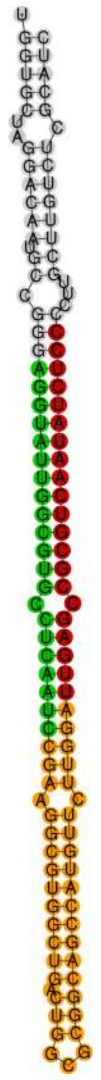

```
                         Star                                                              Mature
UGGUGCUAGGACAAUGCCGGGAGGUAUUGGCGUGCCUCAAUCCGAAGGCGUGGCUGACUGGCGCGGCAGCCAUGUUCUUGGAUUGAGCCGCGUCAAUAUCUCCCCUUGCUUGUCUCGCAUC    30985.1
.(((((..((((((.((.((((((((((((((((.((((((((((.((((((((((.(((...))))))))))))).)))))))))).))))))))))))))))...)))))))).)))))
..................................................................................UUGAGCCGCGUCAAUAUCUCC..................    28623.79294
.....................AGGUAUUGGCGUGCCUCAAUC...............................................................................    26.044552
...................................................................................UGAGCCGCGUCAAUAUCUCCC.................    1.488671
...................................................................................UGAGCCGCGUCAAUAUCUCC..................    135.303244
...................................................................................UGAGCCGCGUCAAUAUCUC...................    4.82198
...................................................................................UGAGCCGCGUCAAUAUCU....................    1.92879
..................................................................................UUGAGCCGCGUCAAUAUCUCCC.................    2.781539
..................................................................................UUGAGCCGCGUCAAUAUC.....................    4.66224
..................................................................................UUGAGCCGCGUCAAUAUCU....................    85.883222
..................................................................................UUGAGCCGCGUCAAUAUCUC...................    182.803701
..................................................................................UUGAGCCGCGUCAAUGUCUCC..................    17.482649
..................................................................................UUGAGCCGCGUCAAUCUCUCC..................    5.53353
..................................................................................UGGAGCCGCGUCAAUAUCUCC..................    2.61104
..................................................................................UUGCGCCGCGUCAAUAUCUCC..................    2.61104
..................................................................................UUGAGCCGCGUCAAUAUCUCCU.................    0.182396
..................................................................................UUGAACCGCGUCAAUAUCUCC..................    1.891437
..................................................................................UUGAGCCGCGUCGAUAUCUCC..................    46.083577
..................................................................................UUGAGCCGCGUCAAUAACUCC..................    0.568493
..................................................................................UUGAGCCGAGUCAAUAUCUCC..................    0.611936
..................................................................................UUGAGCCGCGUCACUAUCUCC..................    17.88324
..................................................................................UUGAGCCGCGCCAAUAUCUCC..................    54.371169
..................................................................................UUGAGCCGCGUUAAUAUCUCC..................    0.563734
..................................................................................UUGAGCGGCGUCAAUAUCUCC..................    9.94175
..................................................................................UUAAGCCGCGUCAAUAUCUCC..................    14.032933
..................................................................................UUGAGCCACGUCAAUAUCUCC..................    10.377519
..................................................................................UUGAGCCGCGUCAAUACCUCC..................    36.84548
..................................................................................UUGAGCCGUGUCAAUAUCUCC..................    16.88122
..................................................................................UUGAGCUGCGUCAAUAUCUCC..................    31.643008
..................................................................................UUGAGCCGCGUCAACAUCUCC..................    15.237448
..................................................................................UUUAGCCGCGUCAAUAUCUCC..................    23.213714
..................................................................................UUGAGCCGCGUCAGUAUCUCC..................    35.100987
..................................................................................CUGAGCCGCGUCAAUAUCUCC..................    40.786219
..................................................................................UUGAGCCGCGUCAAUAUCUCA..................    161.892972
..................................................................................UUGAGCCGCGUCAAUAUCUCU..................    739.816077
..................................................................................UUGAGCCGCGUCAAUAUCUCG..................    3.617155
..................................................................................UUGAGCCGCGUCAAUAUCUCCA.................    12.677216
..................................................................................UUGAGCCGCAUCAAUAUCUCC..................    6.021875
..................................................................................GUGAGCCGCGUCAAUAUCUCC..................    4.882646
..................................................................................UUGAUCCGCGUCAAUAUCUCC..................    21.282703
..................................................................................UAGAGCCGCGUCAAUAUCUCC..................    1.171678
..................................................................................UUGGGCCGCGUCAAUAUCUCC..................    41.365725
..................................................................................UUGAGCAGCGUCAAUAUCUCC..................    17.3525
..................................................................................UUGAGCCGCGUCAAUAUUU....................    0.429803
..................................................................................UCGAGCCGCGUCAAUAUCUCC..................    29.873785
..................................................................................UUGAGCCGCGUCAAUAUCCCC..................    43.794726
..................................................................................UUGAGCCGCGUCAAUAUCUAC..................    3.85978
..................................................................................UUGAGCCGCGUCAAUAUUUCC..................    65.377598
..................................................................................UUGACCCGCGUCAAUAUCUCC..................    7.103923
..................................................................................UUGAGCCUCGUCAAUAUCUCC..................    10.543702
..................................................................................AUGAGCCGCGUCAAUAUCUCC..................    6.499338
..................................................................................UUGAGCCGCGUCCAUAUCUCC..................    21.45061
..................................................................................UUCAGCCGCGUCAAUAUCUCC..................    17.137137
..................................................................................UUGAGUCGCGUCAAUAUCUCC..................    29.526183
..................................................................................UUGAGCCGCGUCAAUAUCUUC..................    26.20299
..................................................................................UUGAGCCGCGUCAAUAUCACC..................    0.837471
..................................................................................UUGAGCCGCGUCAAUAUAUCC..................    2.580149
.................................................................................AUUGAGCCGCGUCAAUAUCUCC..................    2.291639
................................................................................GAUUGAGCCGUGUCAAUAUC.....................    5.59349
```

zma-MIR171m

```
Chromosome:    chr5
Start:         20785909
End:           20786030
Strand:        -
Strand bias:   1.0
Abundance bias:0.977
MFE:           -69.60
```

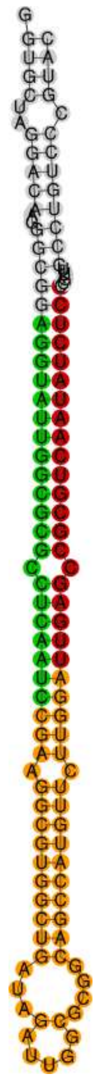

```
                     Star                                                            Mature
GGUGCUAGGACAAGGGCGGAGGUAUUGGCGCGCCUCAAUCCGAAGGCGUGGCUGAUAGAUUGGCGCGGCAGCCAUGUUCUUGGAUUGAGCCGCGUCAAUAUCUCCCGUUGCCUGUCCCGUAC    31457.
.((((..(((((..((((((((((((((((((.((((((((((.((((((((((...(.....)....)))))))))).)))))))))).))))))))))))))))....)))))))).))))
....................................................................................UUGAGCCGCGUCAAUAUCUCC.................    28623.79294
...................AGGUAUUGGCGCGCCUCAAUC..................................................................................    11.767362
.....................................................................................UGAGCCGCGUCAAUAUCUCCC................    1.488671
.....................................................................................UGAGCCGCGUCAAUAUCUCC.................    135.303244
.....................................................................................UGAGCCGCGUCAAUAUCUC..................    4.82198
.....................................................................................UGAGCCGCGUCAAUAUCU...................    1.92879
....................................................................................UUGAGCCGCGUCAAUAUCUCCC................    2.781539
....................................................................................UUGAGCCGCGUCAAUAUC....................    4.66224
....................................................................................UUGAGCCGCGUCAAUAUCU...................    85.883222
....................................................................................UUGAGCCGCGUCAAUAUCUC..................    182.803701
....................................................................................UUGAGCCGCGUCAAUGUCUCC.................    17.482649
....................................................................................UUGAGCCGCGUCAAUCUCUCC.................    5.53353
....................................................................................UGGAGCCGCGUCAAUAUCUCC.................    2.61104
....................................................................................UUGCGCCGCGUCAAUAUCUCC.................    2.61104
....................................................................................UUGAGCCGCGUCAAUAUCUCCU................    0.182396
....................................................................................UUGAACCGCGUCAAUAUCUCC.................    1.891437
....................................................................................UUGAGCCGCGUCGAUAUCUCC.................    46.083577
....................................................................................UUGAGCCGCGUCAAUAACUCC.................    0.568493
....................................................................................UUGAGCCGAGUCAAUAUCUCC.................    0.611936
....................................................................................UUGAGCCGCGUCACUAUCUCC.................    17.88324
....................................................................................UUGAGCCGCGCCAAUAUCUCC.................    54.371169
....................................................................................UUGAGCCGCGUUAAUAUCUCC.................    0.563734
....................................................................................UUGAGCGGCGUCAAUAUCUCC.................    9.94175
....................................................................................UUAAGCCGCGUCAAUAUCUCC.................    14.032933
....................................................................................UUGAGCCACGUCAAUAUCUCC.................    10.377519
....................................................................................UUGAGCCGCGUCAAUACCUCC.................    36.84548
....................................................................................UUGAGCCGUGUCAAUAUCUCC.................    16.88122
....................................................................................UUGAGCUGCGUCAAUAUCUCC.................    31.643008
....................................................................................UUGAGCCGCGUCAACAUCUCC.................    15.237448
....................................................................................UUUAGCCGCGUCAAUAUCUCC.................    23.213714
....................................................................................UUGAGCCGCGUCAGUAUCUCC.................    35.100987
....................................................................................CUGAGCCGCGUCAAUAUCUCC.................    40.786219
....................................................................................UUGAGCCGCGUCAAUAUCUCA.................    161.892972
....................................................................................UUGAGCCGCGUCAAUAUCUCU.................    739.816077
....................................................................................UUGAGCCGCGUCAAUAUCUCG.................    3.617155
....................................................................................UUGAGCCGCGUCAAUAUCUCCA................    12.677216
....................................................................................UUGAGCCGCAUCAAUAUCUCC.................    6.021875
....................................................................................GUGAGCCGCGUCAAUAUCUCC.................    4.882646
....................................................................................UUGAUCCGCGUCAAUAUCUCC.................    21.282703
....................................................................................UAGAGCCGCGUCAAUAUCUCC.................    1.171678
....................................................................................UUGGGCCGCGUCAAUAUCUCC.................    41.365725
....................................................................................UUGAGCAGCGUCAAUAUCUCC.................    17.3525
....................................................................................UUGAGCCGCGUCAAUAUUU...................    0.429803
....................................................................................UCGAGCCGCGUCAAUAUCUCC.................    29.873785
....................................................................................UUGAGCCGCGUCAAUAUCCCC.................    43.794726
....................................................................................UUGAGCCGCGUCAAUAUCUAC.................    3.85978
....................................................................................UUGAGCCGCGUCAAUAUUUCC.................    65.377598
....................................................................................UUGACCCGCGUCAAUAUCUCC.................    7.103923
....................................................................................UUGAGCCUCGUCAAUAUCUCC.................    10.543702
....................................................................................AUGAGCCGCGUCAAUAUCUCC.................    6.499338
....................................................................................UUGAGCCGCGUCCAUAUCUCC.................    21.45061
....................................................................................UUCAGCCGCGUCAAUAUCUCC.................    17.137137
....................................................................................UUGAGUCGCGUCAAUAUCUCC.................    29.526183
....................................................................................UUGAGCCGCGUCAAUAUCUUC.................    26.20299
....................................................................................UUGAGCCGCGUCAAUAUCACC.................    0.837471
....................................................................................UUGAGCCGCGUCAAUAUAUCC.................    2.580149
...................................................................................AUUGAGCCGCGUCAAUAUCUCC.................    2.291639
..................................................................................GAUUGAGCCGUGUCAAUAUC....................    5.59349
```

zma-MIR171n

Chromosome: chr9
Start: 100082902
End: 100083022
Strand: -
Strand bias: 1.0
Abundance bias: 1.0
MFE: -49.30

```
                                     Star                                                             Mature
UGUGUUUGAUCAAUGGAAAGAGCGAUAUUGGUGAGGUUCAAUCCGAUGUUAGGUUUUACAAAAGCUGUAAAAUCAGUAUCUGAUUGAGCCGCGCCAAUAUCUCUUCCUCCAAUUUGCCACU    1713.1
.(((...(((...((((..(((.((((((((((.(((((((((.((((((.(((((((((.....))))))))))))))).))))))))).)))))))))).)))..)))))))...))).
...................................................................................UUGAGCCGCGCCAAUAUCUCU.................    742.75145
......................CGAUAUUGGUGAGGUUCAAUC..............................................................................    27.277822
...................................................................................UUGAGCCGCGCCAAUAUCUC..................    1.772901
...................................................................................UUGAGCCGCGCCAAUAUCUCA.................    4.785427
...................................................................................UUGAGCCGCGCCAAUAUCUCC.................    54.371169
...................................................................................UUGAGCCGCGUCAAUAUCUCU.................    739.816077
...................................................................................UUGAGCCGCGCCAAUAUCACU.................    2.27703
.................................................................................GAUUGAGCCGAGCCAAUAUC....................    1.35015
.................................................................................GAUUGAGCCGGGCCAAUAUC....................    1.15179
................................................................................UGAUUGAGCCGGGCCAAUAUC....................    104.768169
................................................................................UGAUUGAGCCGAGCCAAUAUC....................    32.82447
```

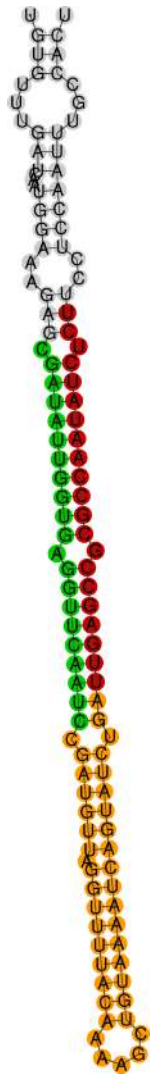

zma-MIR172a

Chromosome: chr7
Start: 54276690
End: 54276806
Strand: -
Strand bias: 1.0
Abundance bias: 1.0
MFE: -49.10

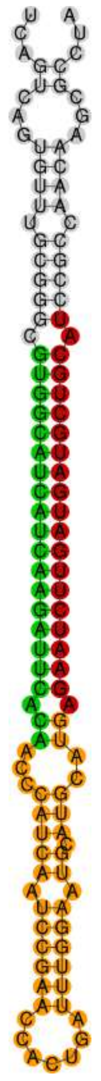

```
                   Star                                                      Mature
UCAGUCAGUGUUUGCGGGCGUGGCAUCAUCAAGAUUCACAACCCAUCAAUCCGAACCACUGAUUUGGAAUGCAUGCAUGAGAAUCUUGAUGAUGCUGCAUCCGCCAACAAGCGCCUA    10478.5
...((...((((.(((((.((((((((((((((((((.((...(((((.((((((.......)))))).)).)))..)).)))))))))))))))))).))))).))))...))...
...............................................................................AGAAUCUUGAUGAUGCUGCAU.................    9755.227011
...................GUGGCAUCAUCAAGAUUCACA.............................................................................    23.50382
.................................................................................AAUCUUGAUGAUGCUGCAU.................    0.182396
................................................................................GAAUCUUGAUGAUGCUGCAU.................    20.73049
...............................................................................AGAAUCUUGAUGAUGCUGCA..................    64.392534
...............................................................................AGAAUCUUGAUGAUGCUGC...................    86.888788
...............................................................................AGAAUCUUGAUGAUGCUGCAA.................    51.33575
...............................................................................AGAAUCUUGAUGAUGCUGCAC.................    218.877153
...............................................................................AGAAUUUUGAUGAUGCUGCAU.................    3.694693
...............................................................................AGAAUCUUGAUGAUGAUGCAU.................    1.75379
...............................................................................AGACUCUUGAUGAUGCUGCAU.................    1.7116
...............................................................................AGAAUCUUGAUGAUUCUGCAU.................    0.459297
...............................................................................AGAAUCUUGCUGAUGCUGCAU.................    3.260114
...............................................................................AGAAUCUUGAUGAAGCUGCAU.................    0.334392
...............................................................................AAAAUCUUGAUGAUGCUGCAU.................    0.303993
...............................................................................AGAAUCUUGAUGAUGCAGCAU.................    1.11605
...............................................................................AGAAUCUUGAUGAUGCUGUAU.................    0.45599
...............................................................................AGAGUCUUGAUGAUGCUGCAU.................    1.83169
...............................................................................AGAAUCUUAAUGAUGCUGCAU.................    0.212795
...............................................................................AGAAUCUUGAUGAUGCCGCAU.................    3.645418
...............................................................................AGAAUCUUGAUGAUGCUGU...................    21.734454
...............................................................................AGAAUCUUGAUGAUGCUGA...................    3.6059
...............................................................................AGAAUCUUGAUAAUGCUGCAU.................    2.56109
...............................................................................AGAAUCUUGGUGAUGCUGCAU.................    2.201964
...............................................................................CGAAUCUUGAUGAUGCUGCAU.................    7.989651
...............................................................................AGAAUCUUGAUGAUGCUGGAU.................    1.555508
...............................................................................AGAAUCUUGAUGAUGCUGCCU.................    20.37914
...............................................................................AGAAUCUUGAUGAUGCUGCAG.................    21.11597
...............................................................................AUAAUCUUGAUGAUGCUGCAU.................    0.182396
...............................................................................AGCAUCUUGAUGAUGCUGCAU.................    3.277351
...............................................................................AGAAUCUUGAUGAUGCUGAAU.................    11.368062
...............................................................................AGAAUCUUGAUGAUGCUACAU.................    1.27549
...............................................................................AGAAUCUUGAAGAUGCUGCAU.................    0.243195
...............................................................................AGAAUAUUGAUGAUGCUGCAU.................    1.07855
...............................................................................AGAAUCUUGAUGCUGCUGCAU.................    23.51772
...............................................................................AGAAUCUUGAUGAUGUUGCAU.................    5.422069
...............................................................................AGAAUCUUGAUUAUGCUGCAU.................    1.94521
...............................................................................AGAAUCUCGAUGAUGCUGCAU.................    0.243195
...............................................................................AGAAUCUUGAUGAUGCUGCGU.................    2.07492
...............................................................................AGAAUCUUUAUGAUGCUGCAU.................    0.93934
...............................................................................AGGAUCUUGAUGAUGCUGCAU.................    0.243195
...............................................................................AGAAUCUUGAUGAUGCUGCAUU................    16.463976
...............................................................................AGAAUCUUGAUGAUGCUGCAUA................    11.716362
..............................................................................GAGAAUCUUGAUGAUGCUGCAU.................    2.366459
..............................................................................GAGAAUCUUGAUGAUGCUGCA..................    4.548239
.............................................................................UGAGAAUCUUGAUGAUGCUGCAU.................    2.784927
.............................................................................UGAGAAUCUUGAUGAUGCUGC...................    44.358549
............................................................................AUGAGAAUCUUGAUGAUGCUGCAU.................    16.453117
...................GUGGCAUCAUCAAGAUUCAC..............................................................................    4.13443
...................GUAGCAUCAUCAAGAUUCACA.............................................................................    0.868372
```

zma-MIR172b

Chromosome: chr5
Start: 221721332
End: 221721491
Strand: -
Strand bias: 1.0
Abundance bias:1.0
MFE: -68.70

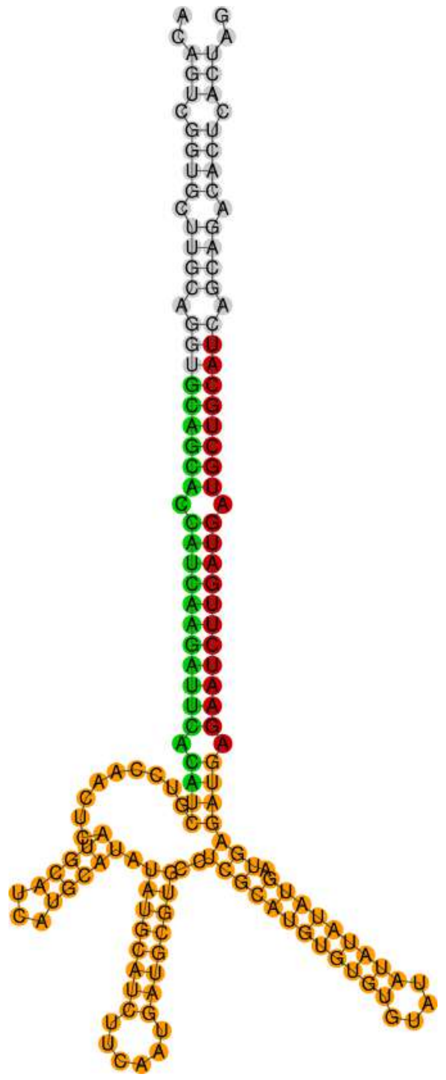

```
                  Star                                                                                                   Mature
ACAGUCGGUGCUUGCAGGUGCAGCACCAUCAAGAUUCACAUCGUCCAACUCAUGCAUCAUGCAUAUAUGCAUCUUCAAUGAUGCGUGCCUCGCAUGUGUGUGUAUAUAUAUAUGAUGAGAUGAGAAUCUUGAUGAUGCUGCAUCAGCAGACACUCACUAG    10871.3
..(((.((((.((((.((((((((.((((((((((.((((.........(((((...))))).((((((((......))))))))..((((((((((((((....))))))))).))))))).))))))))))).))))))))).)))).)))).)))..
..........................................................................................................................AGAAUCUUGAUGAUGCUGCAU.................    9755.227011
...................GCAGCACCAUCAAGAUUCACA........................................................................................................................    367.324447
............................................................................................................................AAUCUUGAUGAUGCUGCAU.................    0.182396
...........................................................................................................................GAAUCUUGAUGAUGCUGCAU.................    20.73049
..........................................................................................................................AGAAUCUUGAUGAUGCUGCA..................    64.392534
..........................................................................................................................AGAAUCUUGAUGAUGCUGC...................    86.888788
..........................................................................................................................AGAAUCUUGAUGAUGCUGCAUCA...............    7.80695
..........................................................................................................................AGAAUCUUGAUGAUGCUGCAA.................    51.33575
..........................................................................................................................AGAAUCUUGAUGAUGCUGCAC.................    218.877153
..........................................................................................................................AGAAUUUUGAUGAUGCUGCAU.................    3.694693
..........................................................................................................................AGAAUCUUGAUGAUGAUGCAU.................    1.75379
..........................................................................................................................AGACUCUUGAUGAUGCUGCAU.................    1.7116
..........................................................................................................................AGAAUCUUGAUGAUUCUGCAU.................    0.459297
..........................................................................................................................AGAAUCUUGCUGAUGCUGCAU.................    3.260114
..........................................................................................................................AGAAUCUUGAUGAAGCUGCAU.................    0.334392
..........................................................................................................................AAAAUCUUGAUGAUGCUGCAU.................    0.303993
..........................................................................................................................AGAAUCUUGAUGAUGCAGCAU.................    1.11605
..........................................................................................................................AGAAUCUUGAUGAUGCUGUAU.................    0.45599
..........................................................................................................................AGAGUCUUGAUGAUGCUGCAU.................    1.83169
..........................................................................................................................AGAAUCUUAAUGAUGCUGCAU.................    0.212795
..........................................................................................................................AGAAUCUUGAUGAUGCCGCAU.................    3.645418
..........................................................................................................................AGAAUCUUGAUGAUGCUGU...................    21.734454
..........................................................................................................................AGAAUCUUGAUGAUGCUGA...................    3.6059
..........................................................................................................................AGAAUCUUGAUAAUGCUGCAU.................    2.56109
..........................................................................................................................AGAAUCUUGGUGAUGCUGCAU.................    2.201964
..........................................................................................................................CGAAUCUUGAUGAUGCUGCAU.................    7.989651
..........................................................................................................................AGAAUCUUGAUGAUGCUGGAU.................    1.555508
..........................................................................................................................AGAAUCUUGAUGAUGCUGCCU.................    20.37914
..........................................................................................................................AGAAUCUUGAUGAUGCUGCAG.................    21.11597
..........................................................................................................................AUAAUCUUGAUGAUGCUGCAU.................    0.182396
..........................................................................................................................AGCAUCUUGAUGAUGCUGCAU.................    3.277351
..........................................................................................................................AGAAUCUUGAUGAUGCUGAAU.................    11.368062
..........................................................................................................................AGAAUCUUGAUGAUGCUACAU.................    1.27549
..........................................................................................................................AGAAUCUUGAAGAUGCUGCAU.................    0.243195
..........................................................................................................................AGAAUAUUGAUGAUGCUGCAU.................    1.07855
..........................................................................................................................AGAAUCUUGAUGCUGCUGCAU.................    23.51772
..........................................................................................................................AGAAUCUUGAUGAUGUUGCAU.................    5.422069
..........................................................................................................................AGAAUCUUGAUUAUGCUGCAU.................    1.94521
..........................................................................................................................AGAAUCUCGAUGAUGCUGCAU.................    0.243195
..........................................................................................................................AGAAUCUUGAUGAUGCUGCGU.................    2.07492
..........................................................................................................................AGAAUCUUUAUGAUGCUGCAU.................    0.93934
..........................................................................................................................AGGAUCUUGAUGAUGCUGCAU.................    0.243195
..........................................................................................................................AGAAUCUUGAUGAUGCUGCAUU................    16.463976
..........................................................................................................................AGAAUCUUGAUGAUGCUGCAUA................    11.716362
.........................................................................................................................GAGAAUCUUGAUGAUGCUGCAU.................    2.366459
.........................................................................................................................GAGAAUCUUGAUGAUGCUGCA..................    4.548239
........................................................................................................................UGAGAAUCUUGAUGAUGCUGCAU.................    2.784927
........................................................................................................................UGAGAAUCUUGAUGAUGCUGC...................    44.358549
.......................................................................................................................AUGAGAAUCUUGAUGAUGCUGCAU.................    16.453117
...................GCAGCACCAUCAAGAUUCA..........................................................................................................................    0.306198
...................GCAGCACCAUCAAGAUUCAC.........................................................................................................................    38.977665
...................GCAGCACCAUCAAGAUUCACAU.......................................................................................................................    0.561363
...................GCAGCAUCAUCAAGAUUCACA........................................................................................................................    5.742344
...................GCAGCACCAUCAAGAUUCACU........................................................................................................................    0.357231
```

zma-MIR172c

```
Chromosome:    chr4
Start:         174154913
End:           174155066
Strand:        +
Strand bias:   1.0
Abundance bias:1.0
MFE:           -73.80
```

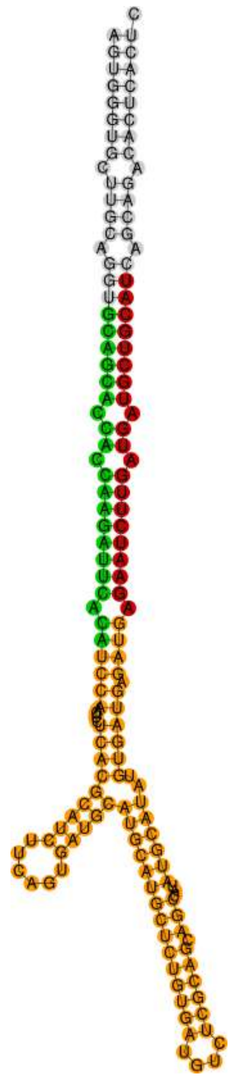

```
                                   Star                                                                                                   Mature
AGUGGGUGCUUGCAGGUGCAGCACCACCAAGAUUCACAUCCAACUCUCACGCAUCUUCAGUGAUGCAUGCAUGCUCUGUGAUGUCUCGCAGCAGCUAUAUGCAUAUGUGAUGAGAUGAGAAUCUUGAUGAUGCUGCAUCAGCAGACACUCACUC    12494.4
((((((((.((((.(((((((((.((.((((((((.((((((....(((((((((......)))))(((((((((((((((....)))))).)))...))))))..)))))).)))).)))))))).)).))))))))).)))).)))))))).
.....................................................................................................................AGAAUCUUGAUGAUGCUGCAU................    9755.227011
.................GCAGCACCACCAAGAUUCACA....................................................................................................................    1712.059348
.......................................................................................................................AAUCUUGAUGAUGCUGCAU................    0.182396
......................................................................................................................GAAUCUUGAUGAUGCUGCAU................    20.73049
.....................................................................................................................AGAAUCUUGAUGAUGCUGCA.................    64.392534
.....................................................................................................................AGAAUCUUGAUGAUGCUGC..................    86.888788
.....................................................................................................................AGAAUCUUGAUGAUGCUGCAUCA..............    7.80695
.....................................................................................................................AGAAUCUUGAUGAUGCUGCAA................    51.33575
.....................................................................................................................AGAAUCUUGAUGAUGCUGCAC................    218.877153
.....................................................................................................................AGAAUUUUGAUGAUGCUGCAU................    3.694693
.....................................................................................................................AGAAUCUUGAUGAUGAUGCAU................    1.75379
.....................................................................................................................AGACUCUUGAUGAUGCUGCAU................    1.7116
.....................................................................................................................AGAAUCUUGAUGAUUCUGCAU................    0.459297
.....................................................................................................................AGAAUCUUGCUGAUGCUGCAU................    3.260114
.....................................................................................................................AGAAUCUUGAUGAAGCUGCAU................    0.334392
.....................................................................................................................AAAAUCUUGAUGAUGCUGCAU................    0.303993
.....................................................................................................................AGAAUCUUGAUGAUGCAGCAU................    1.11605
.....................................................................................................................AGAAUCUUGAUGAUGCUGUAU................    0.45599
.....................................................................................................................AGAGUCUUGAUGAUGCUGCAU................    1.83169
.....................................................................................................................AGAAUCUUAAUGAUGCUGCAU................    0.212795
.....................................................................................................................AGAAUCUUGAUGAUGCCGCAU................    3.645418
.....................................................................................................................AGAAUCUUGAUGAUGCUGU..................    21.734454
.....................................................................................................................AGAAUCUUGAUGAUGCUGA..................    3.6059
.....................................................................................................................AGAAUCUUGAUAAUGCUGCAU................    2.56109
.....................................................................................................................AGAAUCUUGGUGAUGCUGCAU................    2.201964
.....................................................................................................................CGAAUCUUGAUGAUGCUGCAU................    7.989651
.....................................................................................................................AGAAUCUUGAUGAUGCUGGAU................    1.555508
.....................................................................................................................AGAAUCUUGAUGAUGCUGCCU................    20.37914
.....................................................................................................................AGAAUCUUGAUGAUGCUGCAG................    21.11597
.....................................................................................................................AUAAUCUUGAUGAUGCUGCAU................    0.182396
.....................................................................................................................AGCAUCUUGAUGAUGCUGCAU................    3.277351
.....................................................................................................................AGAAUCUUGAUGAUGCUGAAU................    11.368062
.....................................................................................................................AGAAUCUUGAUGAUGCUACAU................    1.27549
.....................................................................................................................AGAAUCUUGAAGAUGCUGCAU................    0.243195
.....................................................................................................................AGAAUAUUGAUGAUGCUGCAU................    1.07855
.....................................................................................................................AGAAUCUUGAUGCUGCUGCAU................    23.51772
.....................................................................................................................AGAAUCUUGAUGAUGUUGCAU................    5.422069
.....................................................................................................................AGAAUCUUGAUUAUGCUGCAU................    1.94521
.....................................................................................................................AGAAUCUCGAUGAUGCUGCAU................    0.243195
.....................................................................................................................AGAAUCUUGAUGAUGCUGCGU................    2.07492
.....................................................................................................................AGAAUCUUUAUGAUGCUGCAU................    0.93934
.....................................................................................................................AGGAUCUUGAUGAUGCUGCAU................    0.243195
.....................................................................................................................AGAAUCUUGAUGAUGCUGCAUU...............    16.463976
.....................................................................................................................AGAAUCUUGAUGAUGCUGCAUA...............    11.716362
....................................................................................................................GAGAAUCUUGAUGAUGCUGCAU................    2.366459
....................................................................................................................GAGAAUCUUGAUGAUGCUGCA.................    4.548239
...................................................................................................................UGAGAAUCUUGAUGAUGCUGCAU................    2.784927
...................................................................................................................UGAGAAUCUUGAUGAUGCUGC..................    44.358549
..................................................................................................................AUGAGAAUCUUGAUGAUGCUGCAU................    16.453117
..................CAGCACCACCAAGAUUCACA....................................................................................................................    0.17779
..................CAGCACCACCAAGAUUCACAU...................................................................................................................    6.599065
.................GCAGCACCACCAAGAUUCA......................................................................................................................    4.304393
.................GCAGCACCACCAAGAUUCAC.....................................................................................................................    315.411903
```

zma-MIR172d

Chromosome: chr6
Start: 172029771
End: 172029875
Strand: +
Strand bias: 1.0
Abundance bias:1.0
MFE: -48.70

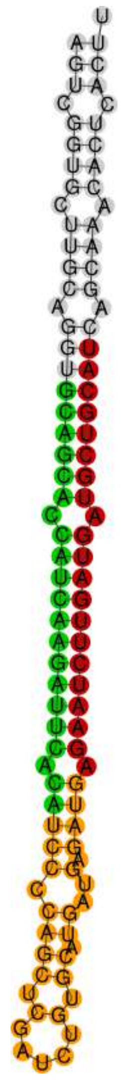

```
                 Star                                                  Mature
AGUCGGUGCUUGCAGGUGCAGCACCAUCAAGAUUCACAUCCCCAGCUCGAUCUGUGCAUGAUGAGAUGAGAAUCUUGAUGAUGCUGCAUCAGCAAACACUCACUU    10871.3
(((.((((.((((.(((((((((.((((((((((.(((((.((((.(.....).)).)).)..).)))).)))))))))).))))))))).)))).)))).))).
....................................................................AGAAUCUUGAUGAUGCUGCAU................    9755.227011
.................GCAGCACCAUCAAGAUUCACA...................................................................    367.324447
......................................................................AAUCUUGAUGAUGCUGCAU................    0.182396
.....................................................................GAAUCUUGAUGAUGCUGCAU................    20.73049
....................................................................AGAAUCUUGAUGAUGCUGCA.................    64.392534
....................................................................AGAAUCUUGAUGAUGCUGC..................    86.888788
....................................................................AGAAUCUUGAUGAUGCUGCAUCA..............    7.80695
....................................................................AGAAUCUUGAUGAUGCUGCAA................    51.33575
....................................................................AGAAUCUUGAUGAUGCUGCAC................    218.877153
....................................................................AGAAUUUUGAUGAUGCUGCAU................    3.694693
....................................................................AGAAUCUUGAUGAUGAUGCAU................    1.75379
....................................................................AGACUCUUGAUGAUGCUGCAU................    1.7116
....................................................................AGAAUCUUGAUGAUUCUGCAU................    0.459297
....................................................................AGAAUCUUGCUGAUGCUGCAU................    3.260114
....................................................................AGAAUCUUGAUGAAGCUGCAU................    0.334392
....................................................................AAAAUCUUGAUGAUGCUGCAU................    0.303993
....................................................................AGAAUCUUGAUGAUGCAGCAU................    1.11605
....................................................................AGAAUCUUGAUGAUGCUGUAU................    0.45599
....................................................................AGAGUCUUGAUGAUGCUGCAU................    1.83169
....................................................................AGAAUCUUAAUGAUGCUGCAU................    0.212795
....................................................................AGAAUCUUGAUGAUGCCGCAU................    3.645418
....................................................................AGAAUCUUGAUGAUGCUGU..................    21.734454
....................................................................AGAAUCUUGAUGAUGCUGA..................    3.6059
....................................................................AGAAUCUUGAUAAUGCUGCAU................    2.56109
....................................................................AGAAUCUUGGUGAUGCUGCAU................    2.201964
....................................................................CGAAUCUUGAUGAUGCUGCAU................    7.989651
....................................................................AGAAUCUUGAUGAUGCUGGAU................    1.555508
....................................................................AGAAUCUUGAUGAUGCUGCCU................    20.37914
....................................................................AGAAUCUUGAUGAUGCUGCAG................    21.11597
....................................................................AUAAUCUUGAUGAUGCUGCAU................    0.182396
....................................................................AGCAUCUUGAUGAUGCUGCAU................    3.277351
....................................................................AGAAUCUUGAUGAUGCUGAAU................    11.368062
....................................................................AGAAUCUUGAUGAUGCUACAU................    1.27549
....................................................................AGAAUCUUGAAGAUGCUGCAU................    0.243195
....................................................................AGAAUAUUGAUGAUGCUGCAU................    1.07855
....................................................................AGAAUCUUGAUGCUGCUGCAU................    23.51772
....................................................................AGAAUCUUGAUGAUGUUGCAU................    5.422069
....................................................................AGAAUCUUGAUUAUGCUGCAU................    1.94521
....................................................................AGAAUCUCGAUGAUGCUGCAU................    0.243195
....................................................................AGAAUCUUGAUGAUGCUGCGU................    2.07492
....................................................................AGAAUCUUUAUGAUGCUGCAU................    0.93934
....................................................................AGGAUCUUGAUGAUGCUGCAU................    0.243195
....................................................................AGAAUCUUGAUGAUGCUGCAUU...............    16.463976
....................................................................AGAAUCUUGAUGAUGCUGCAUA...............    11.716362
...................................................................GAGAAUCUUGAUGAUGCUGCAU................    2.366459
...................................................................GAGAAUCUUGAUGAUGCUGCA.................    4.548239
..................................................................UGAGAAUCUUGAUGAUGCUGCAU................    2.784927
..................................................................UGAGAAUCUUGAUGAUGCUGC..................    44.358549
.................................................................AUGAGAAUCUUGAUGAUGCUGCAU................    16.453117
.................GCAGCACCAUCAAGAUUCA.....................................................................    0.306198
.................GCAGCACCAUCAAGAUUCAC....................................................................    38.977665
.................GCAGCACCAUCAAGAUUCACAU..................................................................    0.561363
.................GCAGCAUCAUCAAGAUUCACA...................................................................    5.742344
.................GCAGCACCAUCAAGAUUCACU...................................................................    0.357231
```

zma-MIR172e

Chromosome: chr3
Start: 145801597
End: 145801762
Strand: +
Strand bias: 1.0
Abundance bias: 0.992
MFE: -69.80

```
                 Star                                                                                                                  Mature
AGCCGGUGAUUUCUGGAGUGGCAUCAUCAAGAUUCACACACUGCAUGCCAACAUAAUGCGCGUGUUCAUGCAUCCAUCGCCGCCGCUGCAUCAUGCAUCAUAUAUAAUAUAUAUAUAUGUGUAUGUGUGGGAAUCUUGAUGAUGCUGCAUUGGAUAUCAAGGGCUA    924.0
((((..((((.(((((.((((((((((((((((((.(((((.((((((((......)).))))))..(((((......((....))))))).(((((.((((((((.....)))))))))))))))))).)))))))))))))))))).))))).))))..)))).
.................................................................................................................................GGAAUCUUGAUGAUGCUGCAU................    826.302885
.................GUGGCAUCAUCAAGAUUCACA................................................................................................................................    23.50382
..................................................................................................................................AAUCUUGAUGAUGCUGCAU................    0.182396
.................................................................................................................................GAAUCUUGAUGAUGCUGCAU................    20.73049
.................................................................................................................................CGAAUCUUGAUGAUGCUGCAU................    7.989651
.................................................................................................................................GGAAUCUUGACGAUGCUGCAU................    1.29681
.................................................................................................................................GGAAUCUUGAUGAUGUUGCAU................    1.29681
................................................................................................................................GGGAAUCUUGAUGAUGCUGCAU................    7.88753
..............................................................................................................................GUGGGAAUCUUGAUGAUGCUGCAU................    22.89206
.................GUGGCAUCAUCAAGAUUCAC.................................................................................................................................    4.13443
.................GUAGCAUCAUCAAGAUUCACA................................................................................................................................    0.868372
```

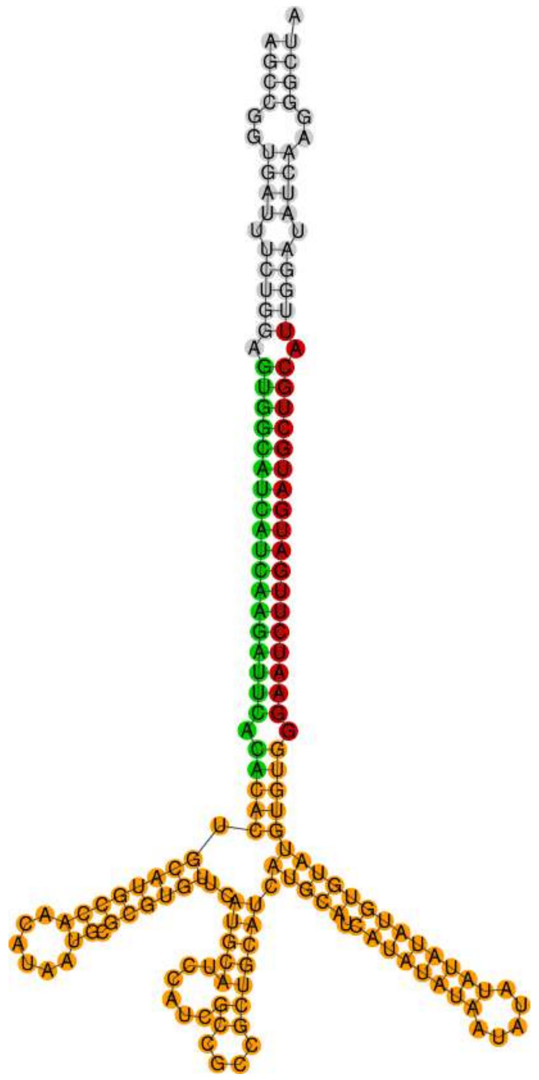

zma-MIR172_N1

Chromosome: chr4
Start: 15295173
End: 15295313
Strand: -
Strand bias: 1.0
Abundance bias: 1.0
MFE: -67.70

```
                  Star                                                                                     Mature
AAGUCACUGUUGUCUUGCGCGGCAUCAUCAGGAUUCACACAAGGUAAACAAAGUCGGAUGCCCAUGCACAUAUGUAGGUCUGGUUCGAUCCGUUGCUUGCGUGAGAAUCCUGAUGAUGCUGCAUUGGAGAACAAGUGACUG    397.2
.((((((((((.(((...((((((((((((((((((.(((.((((((.....(((((((..((.((((....))))))....)))))))...))))))..))).))))))))))))))))))...))).))).))))))).
.......................................................................................................AGAAUCCUGAUGAUGCUGCAU.................    368.000568
..................GCGGCAUCAUCAGGAUUCACA......................................................................................................    7.04256
.......................................................................................................AGAAUCCUGAUGAUGCUGCA..................    0.612396
.......................................................................................................AGAAUCCUGAUGAUGCUGCAUU................    0.714462
..................GCGGCAUCAUCAGGAUUCAC.......................................................................................................    20.2601
..................GCGGCAUCAUCAGGAUUCA........................................................................................................    0.306198
..................GCGGCAUCAUCAGGAUUCACAU.....................................................................................................    0.306198
```

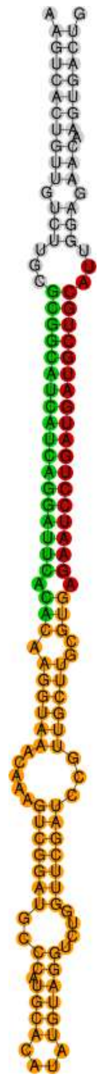

zma-MIR2275a

Chromosome: chr4
Start: 31733907
End: 31734029
Strand: +
Strand bias: 1.0
Abundance bias:0.996
MFE: -70.00

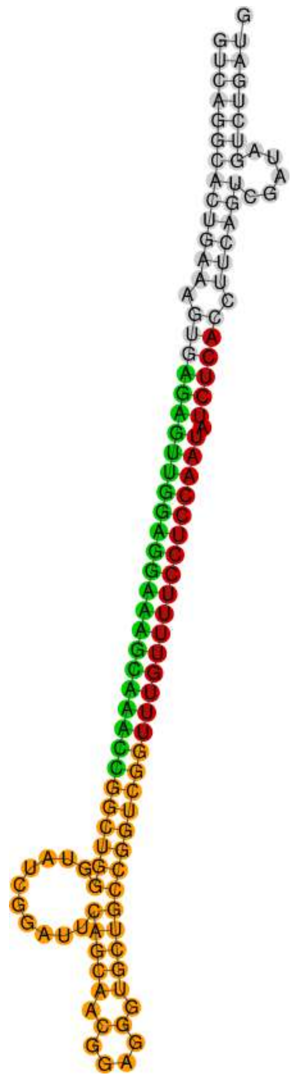

```
                 Star                                                          Mature
GUCAGGCACUGAAAGUGAGAGUUGGAGGAAAGCAAACCGGCUGGGUAUCGGAUUCAGCAACGGAGGGUGCUGCCGGUCGGUUUGUUUUCCUCCAAUAUCUCACCUUCAGUCGAUAGUCUGAUG 1843.4
(((((((((((((.((((((((((((((((((((((((((((((..........(((((.(....).))))))))))))))))))))))))))))))).)))))).)))))).....))))))).
................................................................................UUUGUUUUCCUCCAAUAUCUCA..................... 754.745289
.................AGAGUUGGAGGAAAGCAAACC..................................................................................... 941.243469
.................................................................................UUGUUUUCCUCCAAUAUCUCAC.................... 2.300322
.................................................................................UUGUUUUCCUCCAAUAUCUCA..................... 3.03688
................................................................................UUUGUUUUCCUCCAAUAUC........................ 10.12332
................................................................................UUUGUUUUCCUCCAAUAUCU....................... 2.196863
................................................................................UUUGUUUUCCUCCAAUAUCUC...................... 20.82128
................................................................................UUUGUUUUCCUCCAAUAUCUUA..................... 0.228587
................................................................................UUUGUUUUCCUCCAAUGUCUCA..................... 0.203188
................................................................................UUUGUUUUCCUCCAAUAUCUCAU.................... 0.152391
................................................................................UUUGUUUUCCUCCAAUAUCUCG..................... 0.152391
................................................................................UUUGUUUUCCUCCAAUAUCUCU..................... 6.19586
................................................................................UUUGUUUUCCUCCAAUAUUU....................... 1.017808
................................................................................UUUGUUUUCCUCCAAUAUU........................ 1.490595
................................................................................UUUGUUUUCCUCCAAUAUCUAA..................... 0.152391
................................................................................UUUGGUUUCCUCCAAUAUCUCA..................... 0.152391
................................................................................CUUGUUUUCCUCCAAUAUCUCA..................... 0.279384
................................................................................UUUGUUUUCCUCCAAUAUCUU...................... 0.507971
................................................................................UUUGUUUUCCUCCAAUAUCUG...................... 0.152391
...............................................................................GUUUGUUUUCCUCCAAUAUCUC...................... 2.358105
...............................................................................GUUUGUUUUCCUCCAAUAUCU....................... 0.279384
....................GUUGGAGGAAAGCAAACC..................................................................................... 0.427815
...................AGUUGGAGGAAAGCAAACC..................................................................................... 0.427815
...................AGUUGGAGGAAAGCAAAC...................................................................................... 0.427815
...................AGUUGGAGGAAAGCAAACCGG................................................................................... 0.427815
..................GAGUUGGAGGAAAGCAAACCG.................................................................................... 3.18834
..................GAGUUGGAGGAAAGCAAACC..................................................................................... 4.01181
.................AGAGUUGGAGGAAAGCAA........................................................................................ 1.15948
.................AGAGUUGGAGGAAAGCAAA....................................................................................... 3.40224
.................AGAGUUGGAGGAAAGCAAAC...................................................................................... 19.62544
.................AGAGUUGGAGGAAAGCAAACCG.................................................................................... 2.92456
.................AGAGUUGGAGGAAAGCAAACCGGC.................................................................................. 22.5076
.................AGAGUUGGAGGAAAGCAAACCGG................................................................................... 3.00868
.................AGAGUGGGAGGAAAGCAAACC..................................................................................... 0.152391
.................AGAGUUGGAGGAAAGCGAACC..................................................................................... 0.228587
.................GGAGUUGGAGGAAAGCAAACC..................................................................................... 0.253986
.................AGUGUUGGAGGAAAGCAAACC..................................................................................... 0.152391
.................AGAGCUGGAGGAAAGCAAACC..................................................................................... 0.380978
.................AGAGUUGGAGGAAAGAAAACC..................................................................................... 0.228587
.................AGAGUUGGAGGAAAGUAAACC..................................................................................... 0.228587
.................AGAGUUGGAGGAAAGCAAACU..................................................................................... 12.40569
.................AGAGUUGGAGGAAAGCAAACA..................................................................................... 0.330181
.................AGAGUUGGAGGAAAGCAAACG..................................................................................... 0.17779
.................AGAGAUGGAGGAAAGCAAACC..................................................................................... 0.253986
.................AGGGUUGGAGGAAAGCAAACC..................................................................................... 0.253986
.................AGAGUUGGAGGAAAGCAAAU...................................................................................... 0.53337
.................AGAGUUGGAGGAAAGCAAAA...................................................................................... 0.330181
.................AGAGUUGGAGGCAAGCAAACC..................................................................................... 0.17779
.................AGAGUUGGAGGGAAGCAAACC..................................................................................... 0.279384
.................AGAGUUGGGGGAAAGCAAACC..................................................................................... 0.304783
.................AGAGUUGGAGGAAAGCAAAUC..................................................................................... 0.203188
.................AGAGUUGGAGGAAAUCAAACC..................................................................................... 0.279384
.................AGAGUUAGAGGAAAGCAAACC..................................................................................... 0.203188
.................AGCGUUGGAGGAAAGCAAACC..................................................................................... 0.253986
.................AGAGUUGCAGGAAAGCAAACC..................................................................................... 0.203188
.................AGAGGUGGAGGAAAGCAAACC..................................................................................... 1.04134
.................AGAGUUGUAGGAAAGCAAACC..................................................................................... 0.228587
.................AGAGUUGGAGAAAAGCAAACC..................................................................................... 0.406377
.................AGAGUUGGAGGAAAGCAAACCC.................................................................................... 1.165306
.................AGAGUUGGAGGAAAGCAAACCU.................................................................................... 3.267558
.................AGAGUUGGAGUAAAGCAAACC..................................................................................... 0.152391
.................AGAGUUGGAGGAAAGCCAACC..................................................................................... 0.17779
.................AGAGUUGGAGGAAAGCAAAAC..................................................................................... 0.152391
.................AGAGUUGGAGGAAAACAAACC..................................................................................... 0.279384
................GAGAGUUGGAGGAAAGCAAAC...................................................................................... 2.69225
................GAGAGUUGGAGGAAAGCAAACCGG................................................................................... 0.17779
```

zma-MIR2275b

```
Chromosome:    chr4
Start:         31734058
End:           31734150
Strand:        +
Strand bias:   1.0
Abundance bias:1.0
MFE:           -37.60
```

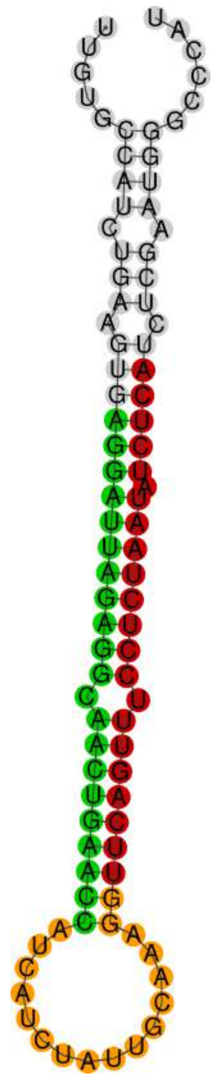

```
                 Star                                     Mature
UUGUGCCAUCUGAAGUGAGGAUUAGAGGCAACUGAACCAUCAUCUAUUGCAAAGGUUCAGUUUCCUCUAAUAUCUCAUCUCGAAUGGGCCCAU    10765.6
.....((((.(((.((((((((((((((.(((((((((...............))))))))).)))))))).)))))).))).)))).......
.......................................................UUCAGUUUCCUCUAAUAUCUCA................    906.577325
.................AGGAUUAGAGGCAACUGAACC.......................................................    9586.887689
........................................................UCAGUUUCCUCUAAUAUCUC.................    0.427815
........................................................UCAGUUUCCUCUAAUAUCUCA................    4.08498
.......................................................UUCAGUUUCCUCUAAUAUCUC.................    44.8178
.......................................................UUCAGUUUCCUCUAAUAUC...................    12.14658
.......................................................UUCAGUUUCCUCUAAUAUCU..................    6.90399
.......................................................UUCAGGUUCCUCUAAUAUCUCA................    1.642986
.......................................................UUCAGUUUCCUCUAAUAUCUCUU...............    0.228587
.......................................................UUCAGUUUCCUCUAAUAUU...................    1.011982
.......................................................UUCAGUUUCCUCUAAUAUCUCU................    16.93273
.......................................................UUCAGUUUCCUCUAAUAUCUCG................    0.427815
.......................................................UUCAGUUUCCUCUAAUAUUU..................    1.433971
.......................................................UUCAGUUUCCUCUAACAUCUCA................    0.152391
.......................................................UUCAGUUUCCCCUAAUAUCUCA................    0.676908
.......................................................UUCAGUUUCCUCUAAUAUCUU.................    0.854698
.......................................................UUCAGCUUCCUCUAAUAUCUCA................    0.17779
.......................................................UUCAGUUUCCUCUAAUAUCUUA................    0.152391
.......................................................UUCAGUUUCCUCUAAUUUCUCA................    0.152391
.......................................................UUCAGUUUCCUCUAAUAUCUAA................    0.17779
....................AUUAGAGGCAACUGAACC.......................................................    2.15888
...................GAUUAGAGGCAACUGAACC.......................................................    1.007089
..................GGAUUAGAGGCAACUGAACC.......................................................    4.16816
.................AGGAUUAGAGGCAACUGAA.........................................................    4.340128
.................AGGAUUAGAGGCAACUGA..........................................................    0.279384
.................AGGAUUAGAGGCAACUGAAC........................................................    19.32783
.................AGGAUUAGAGGCAACUGAACCA......................................................    1.261075
.................AGGAUUAGAGGCAACUGAACCAUC....................................................    0.152391
.................AGGAUUAGAGGAAACUGAACC.......................................................    5.00912
.................AGGGUUAGAGGCAACUGAACC.......................................................    2.009286
.................AGGAUUAGAGGCACCUGAACC.......................................................    0.761957
.................ACGAUUAGAGGCAACUGAACC.......................................................    0.888949
.................AGGAUUAGAGGCUACUGAACC.......................................................    0.203188
.................AGGAUUAGAGGCAAUUGAACC.......................................................    4.14557
.................AGGAUUAGAGGCAACCGAACC.......................................................    2.785921
.................AAGAUUAGAGGCAACUGAACC.......................................................    1.5111
.................AGGAUUAGAGGCAACUGAAA........................................................    2.69225
.................AGGAUUAGAGGCAACUGAUCC.......................................................    0.854698
.................AGGAUUAGAAGCAACUGAACC.......................................................    2.55826
.................AUGAUUAGAGGCAACUGAACC.......................................................    0.279384
.................AGGAUUAGACGCAACUGAACC.......................................................    1.362669
.................AGUAUUAGAGGCAACUGAACC.......................................................    0.406377
.................AGGAUUAGAGGCAACUGAAUC.......................................................    2.91874
.................AGGAUUAGUGGCAACUGAACC.......................................................    0.761957
.................AGGAUUAGAGGGAACUGAACC.......................................................    1.957555
.................AGGCUUAGAGGCAACUGAACC.......................................................    1.138974
.................AGGAUUAGAGGCAACUCAACC.......................................................    1.993673
.................AGGAUUAGAGGCGACUGAACC.......................................................    1.39296
.................AGGAUUAGGGGCAACUGAACC.......................................................    1.947771
.................AGGAUUAUAGGCAACUGAACC.......................................................    1.627373
.................AGGAUUAGAGGCAACUGAAAC.......................................................    3.79791
.................AGCAUUAGAGGCAACUGAACC.......................................................    1.725007
.................AGGAUUAGAGGCAUCUGAACC.......................................................    0.634964
.................AGGAUUAGAGGCAACUGUACC.......................................................    0.304783
.................AGGAUUAGAGGCAACUGAAGC.......................................................    0.431775
.................AGGAUUAGAGGCAACUGAGCC.......................................................    0.482573
.................AGAAUUAGAGGCAACUGAACC.......................................................    1.438864
.................AGGAGUAGAGGCAACUGAACC.......................................................    0.736558
.................AGGAAUAGAGGCAACUGAACC.......................................................    0.457174
.................AGGAUUAGAGGCAACUUAACC.......................................................    2.88356
.................CGGAUUAGAGGCAACUGAACC.......................................................    1.490595
.................AGGAUUAGAGGCAACUGGACC.......................................................    0.457174
.................GGGAUUAGAGGCAACUGAACC.......................................................    2.400053
.................AGGAUUAGAGGCAACGGAACC.......................................................    0.457174
.................AGGUUUAGAGGCAACUGAACC.......................................................    0.507971
.................AGGAUUAGAGGCAAGUGAACC.......................................................    1.851068
.................AGGAUUAGAGGCAACAGAACC.......................................................    0.884989
.................AGGAUUAGAGUCAACUGAACC.......................................................    1.108683
.................AGGAUUAGAGGUAACUGAACC.......................................................    2.201751
.................AGGAUUAGAGGCAACUGACCC.......................................................    1.011982
.................AGGAUUAGAGGCAACUGAACU.......................................................    27.49985
.................AGGAUUAGAGGCAACUGAACA.......................................................    4.63792
.................AGGAUUAGAGGCAACUGAACG.......................................................    2.033745
.................AGGAUUAGAGGCAACUGAAU........................................................    1.510166
.................AGGAUUAAAGGCAACUGAACC.......................................................    2.273053
.................AGGAUUAGAGGCAACUGCACC.......................................................    0.981691
.................AGGAUUAGAGGCCACUGAACC.......................................................    1.866679
.................AGGAUUAGAUGCAACUGAACC.......................................................    1.134082
.................AGGAUUAGAGCCAACUGAACC.......................................................    1.292299
.................AGGAUUGGAGGCAACUGAACC.......................................................    1.749473
.................AGGAUUAGAGGCAACUGAACCU......................................................    11.08706
.................AGGAUUAGAGGCAACUGAACCC......................................................    1.642048
.................AGGAUUAGAGGCAACUGAACCG......................................................    0.558768
.................AGGAUUCGAGGCAACUGAACC.......................................................    2.150961
.................AGGAUUAGAGGCAGCUGAACC.......................................................    0.457174
.................AGGAUCAGAGGCAACUGAACC.......................................................    2.318956
.................AGGAUGAGAGGCAACUGAACC.......................................................    1.84035
.................AGGAUAAGAGGCAACUGAACC.......................................................    0.253986
.................AGGAUUAGAGACAACUGAACC.......................................................    2.100161
.................AGGAUUAGAGGCAAAUGAACC.......................................................    5.5278
.................AGGAUUAGAGGCAACUAAACC.......................................................    2.654033
.................AGGAUUAGCGGCAACUGAACC.......................................................    0.35558
.................AGGAUUUGAGGCAACUGAACC.......................................................    1.378281
.................AGGAUUACAGGCAACUGAACC.......................................................    1.881355
.................AGGACUAGAGGCAACUGAACC.......................................................    1.11754
.................UGGAUUAGAGGCAACUGAACC.......................................................    1.286473
................GAGGAUUAGAGGCAACUGAACC.......................................................    0.152391
```

zma-MIR2275c

```
Chromosome:    chr10
Start:         70573568
End:           70573788
Strand:        +
Strand bias:   1.0
Abundance bias:0.983
MFE:           -85.90
```

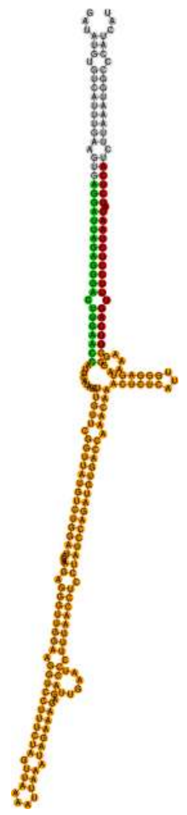

```
                    Star                                                                                                                                                                         Mature
GAUAUGUGUCAUUUGAAGUGAGGAUUAGAGGGACUUGAACCAUUCAUAGCUUGUUCGGUUAUGUCUGGAUCGAAGAGGGUUGGAAGGUCCUUUCUAGUUAAAAUUAAAUAGAAAGAGAUUGAAUCCCUUUAACCUCCUAUCCAGAUGUGACCAAACAAACUCUCAUUGGGAGUAAAAGGGGUUCAGUUUCCUCUAAUAUCUCAUCUUAAAUGGCCCAUCAU    5957.0
...(((.(((((((((.((((((((((((((((.(((((((.........(((((.((((((((((((((...((.((((((((.(((((((((((.(((....))).))))))).))......)).)))))))).)))))))))))))))).)))))((((((...))))))......))))))).)))))))))).)))))).))))))))).)))...
.....................................................................................................................................................................................UUCAGUUUCCUCUAAUAUCUCA..................    906.577325
....................AGGAUUAGAGGGACUUGAACC....................................................................................................................................................................................    4797.12553
......................................................................................................................................................................................UCAGUUUCCUCUAAUAUCUCA..................    4.08498
.....................................................................................................................................................................................UUCAGUUUCCUCUAAUAUCUC...................    44.8178
.....................................................................................................................................................................................UUCAGUUUCCUCUAAUAUC.....................    12.14658
.....................................................................................................................................................................................UUCAGUUUCCUCUAAUAUCU....................    6.90399
.....................................................................................................................................................................................UUCAGGUUCCUCUAAUAUCUCA..................    1.642986
.....................................................................................................................................................................................UUCAGUUUCCUCUAAUAUU.....................    1.011982
.....................................................................................................................................................................................UUCAGUUUCCUCUAAUAUCUCU..................    16.93273
.....................................................................................................................................................................................UUCAGUUUCCUCUAAUAUUU....................    1.433971
.....................................................................................................................................................................................UUCAGUUUCCCCUAAUAUCUCA..................    0.676908
.....................................................................................................................................................................................UUCAGUUUCCUCUAAUAUCUU...................    0.854698
.......................AUUAGAGGGACUUGAACC....................................................................................................................................................................................    1.551177
......................GAUUAGAGGGACUUGAACC....................................................................................................................................................................................    1.367561
.....................GGAUUAGAGGGACUUGAACC....................................................................................................................................................................................    2.11973
....................AGGAUUAGAGGGACUUGAAC.....................................................................................................................................................................................    4.05678
....................AGGAUUAGAGGGACUUGAACCU...................................................................................................................................................................................    2.359038
....................AGGAUUAGAGGGACUUGAACCC...................................................................................................................................................................................    0.965145
....................AGGCUUAGAGGGACUUGAACC....................................................................................................................................................................................    0.431775
....................AGGGUUAGAGGGACUUGAACC....................................................................................................................................................................................    1.06674
....................GGGAUUAGAGGGACUUGAACC....................................................................................................................................................................................    0.711159
....................AGGAUUAGAGGGACUUCAACC....................................................................................................................................................................................    0.761957
....................AGGAUGAGAGGGACUUGAACC....................................................................................................................................................................................    0.431775
....................AGGAUUAGAGGGGCUUGAACC....................................................................................................................................................................................    1.06674
....................AGGAUUACAGGGACUUGAACC....................................................................................................................................................................................    0.482573
....................AGGAUUAUAGGGACUUGAACC....................................................................................................................................................................................    0.685761
....................AGGAUUAGAGGGACUUAAACC....................................................................................................................................................................................    1.560964
....................AGGAUUAGAGGGAAUUGAACC....................................................................................................................................................................................    1.37152
....................AGGAUUAGAGGGACCUGAACC....................................................................................................................................................................................    1.591258
....................AGGAUUCGAGGGACUUGAACC....................................................................................................................................................................................    1.04134
....................AGGAUUAGAAGGACUUGAACC....................................................................................................................................................................................    0.761957
....................AGGAUUAGAGGGAUUUGAACC....................................................................................................................................................................................    2.704833
....................AGGAUUAGGGGGACUUGAACC....................................................................................................................................................................................    1.205384
....................AGGAUUAGAGGAACUUGAACC....................................................................................................................................................................................    1.42232
....................AGGAUUAGAGGUACUUGAACC....................................................................................................................................................................................    0.507971
....................AGGAUUAGAGGGACUUGCACC....................................................................................................................................................................................    0.431775
....................AAGAUUAGAGGGACUUGAACC....................................................................................................................................................................................    0.634964
....................AGGAUUAAAGGGACUUGAACC....................................................................................................................................................................................    0.634964
....................AGGAUUAGAGGGACGUGAACC....................................................................................................................................................................................    0.711159
....................AGGAUUAGAGGGACUUGAACU....................................................................................................................................................................................    1.49851
....................AGGAUUAGAGGGACUUGAACA....................................................................................................................................................................................    2.704833
....................AGGAUUAGAGGGACUUGAACG....................................................................................................................................................................................    1.09214
....................AGGAUUGGAGGGACUUGAACC....................................................................................................................................................................................    0.507971
....................AGGAUUAGUGGGACUUGAACC....................................................................................................................................................................................    0.507971
....................AGAAUUAGAGGGACUUGAACC....................................................................................................................................................................................    0.736558
....................AGGAUUAGAGGGACUUUAACC....................................................................................................................................................................................    1.667448
....................AGGAUUAGAGCGACUUGAACC....................................................................................................................................................................................    0.53337
....................AGGAUUAGAGAGACUUGAACC....................................................................................................................................................................................    0.685761
....................AGGAUUAGAGGGACUCGAACC....................................................................................................................................................................................    1.646945
....................AGGACUAGAGGGACUUGAACC....................................................................................................................................................................................    0.53337
....................AGGAUCAGAGGGACUUGAACC....................................................................................................................................................................................    0.736558
....................AGGAUUAGAGGGCCUUGAACC....................................................................................................................................................................................    0.660362
....................AGGAUUAGAGGGACUUGAAAC....................................................................................................................................................................................    2.465525
....................AGGAUUAGAGGGACUUGAAUC....................................................................................................................................................................................    0.990544
....................AGGAUUAGACGGACUUGAACC....................................................................................................................................................................................    0.431775
....................AGGAUUUGAGGGACUUGAACC....................................................................................................................................................................................    0.53337
...................GAGGAUUAGAGGGACUUGAACC....................................................................................................................................................................................    0.507971
...................GAGGAUUAGAGGGACUUGAACCAU..................................................................................................................................................................................    0.43814
...................GAGGAUUAGAGGGACUUGAACCA...................................................................................................................................................................................    0.431775
.................GUGAGGAUUAGAGGGACUUGAACC....................................................................................................................................................................................    3.131716
```

zma-MIR319a

Chromosome: chr3
Start: 215788709
End: 215788926
Strand: +
Strand bias: 1.0
Abundance bias:0.994
MFE: -101.80

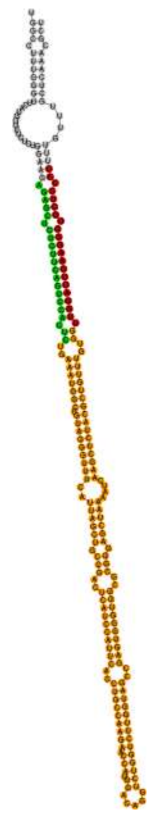

```
                                                       Star                                                                                                                                                       Mature
UGGCCUUUGGGUUCAUGUUUUCUCUGGAAGAGAGCUCUCUUCAGUCCACUCUGAAAUGGCUGUAGGGUUUCAUUAGCUGCCGACUCAUCCAUUCACCUGCCAAGAACCAUGGACAGGUCUGGUCUUGGUAGCCGAGUGGGUGGCGCGGGAGCUAAAAUCAAGCUCUACGCUGUUUGUGGUUGGACUGAAGGGUGCUCCCUUUUGUUUGCUCAAACGCU   627848.9
.(((.(((((((.............(((((.((((.((((((((((((.((..(((((((.(((((((((..((((((.(((..((((((((((..(((((((((.(((.((.....))))))))))))))..))))))))))..))).))))))....))))))))))))))))..)).)))))))))))).)))).)))))....))))))).)))
...................................................................................................................................................................................UUGGACUGAAGGGUGCUCCCU..................   418118.26271
..............................AGAGCUCUCUUCAGUCCACUC.......................................................................................................................................................................   374.513232
....................................................................................................................................................................................UGGACUGAAGGGUGCUCCCU..................   1676.845014
....................................................................................................................................................................................UGGACUGAAGGGUGCUCCC...................   707.07213
...................................................................................................................................................................................UUGGACUGAAGGGUGCUCC....................   8315.07552
...................................................................................................................................................................................UUGGACUGAAGGGUGCUCCCUU.................   23461.594238
...................................................................................................................................................................................UUGGACUGAAGGGUGCUCCC...................   54397.167655
...................................................................................................................................................................................UUGGACUGAAGGGUGCUCCCUUUU...............   965.075343
...................................................................................................................................................................................UUGGACUGAAGGGUGCUCCCUUU................   5141.715327
...................................................................................................................................................................................UUGGACUGAAGGGUGCUC.....................   2352.915079
...................................................................................................................................................................................UUGGACUGAAGGGUGCUCCCCU.................   1610.694648
...................................................................................................................................................................................UUGGACUGAAGGGUGCCCCCU..................   1171.307577
...................................................................................................................................................................................UCGGACUGAAGGGUGCUCCCU..................   788.890278
...................................................................................................................................................................................UUGUACUGAAGGGUGCUCCCU..................   1025.937631
...................................................................................................................................................................................UUGGACUGAAGGGUGCUCCU...................   7955.293757
...................................................................................................................................................................................UUGGACUGAAGGGUGCUCCUU..................   1144.126915
...................................................................................................................................................................................UAGGACUGAAGGGUGCUCCCU..................   157.567155
...................................................................................................................................................................................UUGGACUGAAGGGUGCUCU....................   1057.157562
...................................................................................................................................................................................UUGGACUGAAGGGUGCUCA....................   322.255009
...................................................................................................................................................................................UUGGACUGAAGGGUGUUCCCU..................   447.288077
...................................................................................................................................................................................UUGGACUGAAGGGUGCUCCA...................   2732.547481
...................................................................................................................................................................................UUGGACUGAAGGGUGCUCCG...................   247.287804
...................................................................................................................................................................................UUGGACUGAAGGGUGCUCCAU..................   608.859653
...................................................................................................................................................................................UUGGACUGAAGGGCGCUCCCU..................   872.752804
...................................................................................................................................................................................UUGGACUGAAGGGUGCUCUCU..................   443.074879
...................................................................................................................................................................................CUGGACUGAAGGGUGCUCCCU..................   808.683369
...................................................................................................................................................................................UUGGACUGAAGGGUGCUCCCC..................   40268.98298
...................................................................................................................................................................................UUGGACUGAAUGGUGCUCCCU..................   375.593347
...................................................................................................................................................................................UUGGGCUGAAGGGUGCUCCCU..................   1132.674157
...................................................................................................................................................................................UUGGACUAAAGGGUGCUCCCU..................   548.813267
...................................................................................................................................................................................UUUGACUGAAGGGUGCUCCCU..................   384.243505
...................................................................................................................................................................................AUGGACUGAAGGGUGCUCCCU..................   257.829989
...................................................................................................................................................................................UUGGACUGAAGGGUGCCCC....................   268.368677
...................................................................................................................................................................................UUGGACUGAAGGGUGCUCCCUA.................   1011.4904
...................................................................................................................................................................................UUGGACUGAAGGGUUCUCCCU..................   228.097843
...................................................................................................................................................................................UUGGACUGAAGAGUGCUCCCU..................   269.108516
...................................................................................................................................................................................UUGGAUUGAAGGGUGCUCCCU..................   307.746833
...................................................................................................................................................................................UUGGACUGAAGGGUGCUCCGU..................   179.554314
...................................................................................................................................................................................UUGGACUGAAGGGUACUCCCU..................   380.215306
...................................................................................................................................................................................UUGGACUGAAGGGGGCUCCCU..................   207.213561
...................................................................................................................................................................................UUGGACUGGAGGGUGCUCCCU..................   1034.462932
...................................................................................................................................................................................UUGGACUGAAGGGUGCACCCU..................   258.047838
...................................................................................................................................................................................UUGAACUGAAGGGUGCUCCCU..................   225.270418
...................................................................................................................................................................................UUGGACUGUAGGGUGCUCCCU..................   182.96287
...................................................................................................................................................................................UUGGACCGAAGGGUGCUCCCU..................   757.029182
...................................................................................................................................................................................UUGGACUGAAGGAUGCUCCCU..................   234.021906
...................................................................................................................................................................................UUGGACUGAAAGGUGCUCCCU..................   241.201862
...................................................................................................................................................................................UUAGACUGAAGGGUGCUCCCU..................   306.864608
...................................................................................................................................................................................UUGGACUGAGGGGUGCUCCCU..................   1152.284605
...................................................................................................................................................................................UUGGACUGAAGUGUGCUCCCU..................   370.248244
...................................................................................................................................................................................UUGGACUGAAGGGUGCUCCCA..................   18511.810851
...................................................................................................................................................................................UUGGACUGAAGGGUGCUCCCG..................   10524.289382
...................................................................................................................................................................................UUGGACUUAAGGGUGCUCCCU..................   311.941185
...................................................................................................................................................................................UUGGACUGAAGGGAGCUCCCU..................   235.138647
...................................................................................................................................................................................UUGGACUGAAGGGUGCUCACU..................   303.867267
...................................................................................................................................................................................UUGGACUGAAGGGUGCUCCCUG.................   318.301184
...................................................................................................................................................................................UUGGACUGAAGGGUGCUACCU..................   311.819303
...................................................................................................................................................................................UUGGACUGAAGGGUGCUUCCU..................   643.494289
```

zma-MIR319b

Chromosome: chr3
Start: 2038971
End: 2039201
Strand: +
Strand bias: 1.0
Abundance bias: 0.984
MFE: -104.60

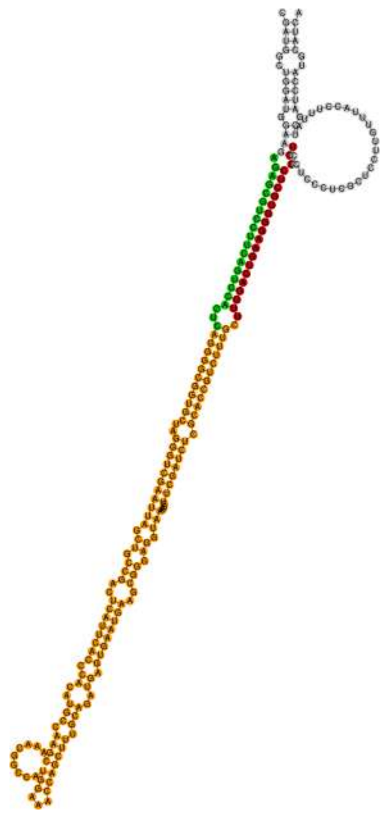

Star · Mature

```
CGAUGGCUGGAUGGAAGAGAGCGUCCUUCAGUCCACUCAGGGGCGGUGCUAGGGUCGAAUUAGCUGCCGACUCAUUCACCCACAUGCCAAGCAAACGGCCAUGGAAACCAGCUUUGCAGAUGAGUGAAUGAAGCGGGAGGUAAAAGCUUCGAUCUCGCACCGUCUUUGCUUGGACUGAAGGGUGCUCCCUCCUCCCUCGCUCCUUGUUUACCUUUAUCGAUCCAUGCAUCA   647441.1
.((((..(((((.((((.((((((((((((((((..((((((((((((..((((((((((.((.(((..((((((((.((..(((.((((.........(((...))))))).)))..)).))))))))..))).)).)))....)))))))).)))))))))))))..))))))))))))))))).))........................)).))))).))))).
```

Read	Abundance
UUGGACUGAAGGGUGCUCCCU (mature)	418118.26271
AGAGCGUCCUUCAGUCCACUC (star)	1230.467497
UGGACUGAAGGGUGCUCCCU	1676.845014
UGGACUGAAGGGUGCUCCC	707.07213
UUGGACUGAAGGGUGCUCC	8315.07552
UUGGACUGAAGGGUGCUCCCUC	3917.507372
UUGGACUGAAGGGUGCUCCC	54397.167655
UUGGACUGAAGGGUGCUC	2352.915079
UUGGACUGAAGGGUGCCCCCU	1171.307577
UCGGACUGAAGGGUGCUCCCU	788.890278
UUGUACUGAAGGGUGCUCCCU	1025.937631
UUGGACUGAAGGGUGCUCCU	7955.293757
UUGGACUGAAGGGUGCUCCUU	1144.126915
UUGGACUGAAGGGUGCUCU	1057.157562
UUGGACUGAAGGGUGCUCA	322.255009
UUGGACUGAAGGGUGUUCCCU	447.288077
UUGGACUGAAGGGUGCUCCA	2732.547481
UUGGACUGAAGGGUGCUCCAU	608.859653
UUGGACUGAAGGGCGCUCCCU	872.752804
UUGGACUGAAGGGUGCUCUCU	443.074879
CUGGACUGAAGGGUGCUCCCU	808.683369
UUGGACUGAAGGGUGCUCCCC	40268.98298
UUGGACUGAAUGGUGCUCCCU	375.593347
UUGGGCUGAAGGGUGCUCCCU	1132.674157
UUGGACUAAAGGGUGCUCCCU	548.813267
UUUGACUGAAGGGUGCUCCCU	384.243505
AUGGACUGAAGGGUGCUCCCU	257.829989
UUGGACUGAAGGGUGCCCC	268.368677
UUGGACUGAAGGGUGCUCCCUA	1011.4904
UUGGACUGAAGAGUGCUCCCU	269.108516
UUGGAUUGAAGGGUGCUCCCU	307.746833
UUGGACUGAAGGGUACUCCCU	380.215306
UUGGACUGGAGGGUGCUCCCU	1034.462932
UUGGACUGAAGGGUGCACCCU	258.047838
UUGGACCGAAGGGUGCUCCCU	757.029182
UUAGACUGAAGGGUGCUCCCU	306.864608
UUGGACUGAGGGGUGCUCCCU	1152.284605
UUGGACUGAAGUGUGCUCCCU	370.248244
UUGGACUGAAGGGUGCUCCCA	18511.810851
UUGGACUGAAGGGUGCUCCCG	10524.289382
UUGGACUUAAGGGUGCUCCCU	311.941185
UUGGACUGAAGGGUGCUCACU	303.867267
UUGGACUGAAGGGUGCUCCCUG	318.301184
UUGGACUGAAGGGUGCUACCU	311.819303
UUGGACUGAAGGGUGCUUCCU	643.494289
CUUGGACUGAAGGGUGCUCC	770.275733
CUUGGACUGAAGGGUGCUCCC	2109.078708
CUUGGACUGAAGGGUGCUCCCUC	673.668376
CUUGGACUGAAGGGUGCUCCCU	27007.61004
CUUGGACUGAAGGGUGCUCCU	452.156451
CUUGGACUGAAGGGUGCUCCCUU	1866.443918
CUUGGACUGAAGGGUGCUCCCG	719.368885
CUUGGACUGAAGGGUGCUCCCC	2373.306995
CUUGGACUGAAGGGUGCUCCCA	1187.864854
GCUUGGACUGAAGGGUGCUCC	601.533812
UGCUUGGACUGAAGGGUGCUC	388.504112
AGAGCGUCCUUCAGUCCACUCA	253.485677
AGAGCGUCCUUCAGUCCACU	334.98115

zma-MIR319c

Chromosome: chr8
Start: 152709451
End: 152709694
Strand: -
Strand bias: 1.0
Abundance bias: 0.994
MFE: -109.30

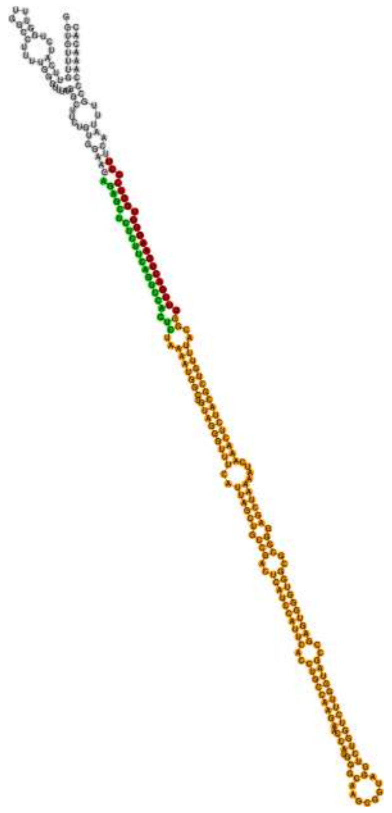

```
                                              Star                                                                                                                                                                   Mature
GGUGUUUGUUCAUCUGGUUUGGCCUUUUGGGUUUAUGGCUUCUGUGGAAGAGAGCUCUCUUCAGUCCACUCUAAAAUGGCUGUAGGGUUUCAUUAGCUGCCGACUCAUCCAUUCACCUGCCAAGAACCAUGGGCAAGCGGUAGGUCUGGUCUUGGUAGCCGAGUGGGUGGCGCGGGAGCUAAAAUCAAACUCUACGCUGUUUACGGUUGGACUGAAGGGUGCUCCCUUCAAUUUGCCCAAACAC   624456.2
.((((((((((...(((...)))...))))).....(((....((.((((.((((.(((((((((((.((..(((((((.(((((((((..((((((.(((..((((((((((..(((((((((.(((.((.(........).))))))))))))))..))))))))))..))).))))))....)))))))))))))))..)).))))))))))).)))).)))).))..))))))))))
..............................................................................................................................................................................................................UUGGACUGAAGGGUGCUCCCU.................   418118.26271
..................................................AGAGCUCUCUUCAGUCCACUC.............................................................................................................................................................................   374.513232
...............................................................................................................................................................................................................UGGACUGAAGGGUGCUCCCU.................   1676.845014
...............................................................................................................................................................................................................UGGACUGAAGGGUGCUCCC..................   707.07213
..............................................................................................................................................................................................................UUGGACUGAAGGGUGCUCC...................   8315.07552
..............................................................................................................................................................................................................UUGGACUGAAGGGUGCUCCCUU................   23461.594238
..............................................................................................................................................................................................................UUGGACUGAAGGGUGCUCCC..................   54397.167655
..............................................................................................................................................................................................................UUGGACUGAAGGGUGCUCCCUUC...............   2770.102752
..............................................................................................................................................................................................................UUGGACUGAAGGGUGCUC....................   2352.915079
..............................................................................................................................................................................................................UUGGACUGAAGGGUGCUCCCCU................   1610.694648
..............................................................................................................................................................................................................UUGGACUGAAGGGUGCCCCCU.................   1171.307577
..............................................................................................................................................................................................................UCGGACUGAAGGGUGCUCCCU.................   788.890278
..............................................................................................................................................................................................................UUGUACUGAAGGGUGCUCCCU.................   1025.937631
..............................................................................................................................................................................................................UUGGACUGAAGGGUGCUCCU..................   7955.293757
..............................................................................................................................................................................................................UUGGACUGAAGGGUGCUCCUU.................   1144.126915
..............................................................................................................................................................................................................UAGGACUGAAGGGUGCUCCCU.................   157.567155
..............................................................................................................................................................................................................UUGGACUGAAGGGUGCUCU...................   1057.157562
..............................................................................................................................................................................................................UUGGACUGAAGGGUGCUCA...................   322.255009
..............................................................................................................................................................................................................UUGGACUGAAGGGUGUUCCCU.................   447.288077
..............................................................................................................................................................................................................UUGGACUGAAGGGUGCUCCA..................   2732.547481
..............................................................................................................................................................................................................UUGGACUGAAGGGUGCUCCG..................   247.287804
..............................................................................................................................................................................................................UUGGACUGAAGGGUGCUCCAU.................   608.859653
..............................................................................................................................................................................................................UUGGACUGAAGGGCGCUCCCU.................   872.752804
..............................................................................................................................................................................................................UUGGACUGAAGGGUGCUCUCU.................   443.074879
..............................................................................................................................................................................................................CUGGACUGAAGGGUGCUCCCU.................   808.683369
..............................................................................................................................................................................................................UUGGACUGAAGGGUGCUCCCC.................   40268.98298
..............................................................................................................................................................................................................UUGGACUGAAUGGUGCUCCCU.................   375.593347
..............................................................................................................................................................................................................UUGGGCUGAAGGGUGCUCCCU.................   1132.674157
..............................................................................................................................................................................................................UUGGACUAAAGGGUGCUCCCU.................   548.813267
..............................................................................................................................................................................................................UUUGACUGAAGGGUGCUCCCU.................   384.243505
..............................................................................................................................................................................................................AUGGACUGAAGGGUGCUCCCU.................   257.829989
..............................................................................................................................................................................................................UUGGACUGAAGGGUGCCCC...................   268.368677
..............................................................................................................................................................................................................UUGGACUGAAGGGUGCUCCCUA................   1011.4904
..............................................................................................................................................................................................................UUGGACUGAAGGGUUCUCCCU.................   228.097843
..............................................................................................................................................................................................................UUGGACUGAAGAGUGCUCCCU.................   269.108516
..............................................................................................................................................................................................................UUGGACUGAAGGGUGCUCCUUU................   156.185005
..............................................................................................................................................................................................................UUGGAUUGAAGGGUGCUCCCU.................   307.746833
..............................................................................................................................................................................................................UUGGACUGAAGGGUGCUCCGU.................   179.554314
..............................................................................................................................................................................................................UUGGACUGAAGGGUACUCCCU.................   380.215306
..............................................................................................................................................................................................................UUGGACUGAAGGGGGCUCCCU.................   207.213561
..............................................................................................................................................................................................................UUGGACUGGAGGGUGCUCCCU.................   1034.462932
..............................................................................................................................................................................................................UUGGACUGAAGGGUGCACCCU.................   258.047838
..............................................................................................................................................................................................................UUGAACUGAAGGGUGCUCCCU.................   225.270418
..............................................................................................................................................................................................................UUGGACUGUAGGGUGCUCCCU.................   182.96287
..............................................................................................................................................................................................................UUGGACCGAAGGGUGCUCCCU.................   757.029182
..............................................................................................................................................................................................................UUGGACUGAAGGAUGCUCCCU.................   234.021906
..............................................................................................................................................................................................................UUGGACUGAAAGGUGCUCCCU.................   241.201862
..............................................................................................................................................................................................................UUAGACUGAAGGGUGCUCCCU.................   306.864608
..............................................................................................................................................................................................................UUGGACUGAGGGGUGCUCCCU.................   1152.284605
..............................................................................................................................................................................................................UUGGACUGAAGUGUGCUCCCU.................   370.248244
..............................................................................................................................................................................................................UUGGACUGAAGGGUGCUCCCA.................   18511.810851
..............................................................................................................................................................................................................UUGGACUGAAGGGUGCUCCCG.................   10524.289382
..............................................................................................................................................................................................................UUGGACUUAAGGGUGCUCCCU.................   311.941185
..............................................................................................................................................................................................................UUGGACUGAAGGGAGCUCCCU.................   235.138647
..............................................................................................................................................................................................................UUGGACUGAAGGGUGCUCACU.................   303.867267
..............................................................................................................................................................................................................UUGGACUGAAGGGUGCUCCCUG................   318.301184
..............................................................................................................................................................................................................UUGGACUGAAGGGUGCUACCU.................   311.819303
..............................................................................................................................................................................................................UUGGACUGAAGGGUGCUUCCU.................   643.494289
```

zma-MIR319d

Chromosome: chr8
Start: 27608248
End: 27608455
Strand: -
Strand bias: 1.0
Abundance bias:0.984
MFE: -102.30

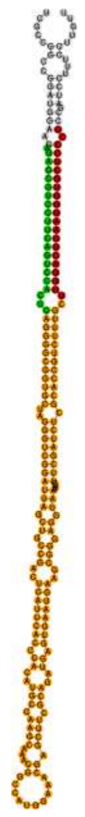

```
                  Star                                                                                                                                                  Mature
UCGCCGGCCGGAUGGAAGAGAGCGUCCUUCAGUCCACUCAGGGGCGGUGCUAGGGUCGGAUUAGCUGCCGACUCAUUCACCCACAUGCCAAGCAAACGGCCAUGGAAACGAGCUUCGCAGAUGAGUGAAUGAAGCGGGAGGUAAAAGCUUCGAUCUCGCACCGUCUUUGCUUGGACUGAAGGGUGCUCCCUCCGAUCCUUUCCUUGUU   647405.5
.....((..(((((((.(.(((((((((((((((((..(((((((((((((..((((((((((.((.(((..((((((((.((..(((.((((...((..........)).)))).)))..)).))))))))..))).)).)))....)))))))).))))))))))))..)))))))))))))))))).))).))))...)).....
..........................................................................................................................................................................UUGGACUGAAGGGUGCUCCCU.................   418118.26271
...........................................................................................................................................................................UGGACUGAAGGGUGCUCCCU.................   1676.845014
...........................................................................................................................................................................UGGACUGAAGGGUGCUCCC..................   707.07213
..........................................................................................................................................................................UUGGACUGAAGGGUGCUCC...................   8315.07552
..........................................................................................................................................................................UUGGACUGAAGGGUGCUCCCUC................   3917.507372
..........................................................................................................................................................................UUGGACUGAAGGGUGCUCCC..................   54397.167655
..........................................................................................................................................................................UUGGACUGAAGGGUGCUC....................   2352.915079
..........................................................................................................................................................................UUGGACUGAAGGGUGCCCCU..................   1171.307577
..........................................................................................................................................................................UCGGACUGAAGGGUGCUCCCU.................   788.890278
..........................................................................................................................................................................UUGUACUGAAGGGUGCUCCCU.................   1025.937631
..........................................................................................................................................................................UUGGACUGAAGGGUGCUCCU..................   7955.293757
..........................................................................................................................................................................UUGGACUGAAGGGUGCUCCCUU................   1144.126915
..........................................................................................................................................................................UUGGACUGAAGGGUGCUCU...................   1057.157562
..........................................................................................................................................................................UUGGACUGAAGGGUGCUCA...................   322.255009
..........................................................................................................................................................................UUGGACUGAAGGGUGUUCCCU.................   447.288077
..........................................................................................................................................................................UUGGACUGAAGGGUGCUCCA..................   2732.547481
..........................................................................................................................................................................UUGGACUGAAGGGUGCUCCAU.................   608.859653
..........................................................................................................................................................................UUGGACUGAAGGGCGCUCCCU.................   872.752804
..........................................................................................................................................................................UUGGACUGAAGGGUGCUCUCU.................   443.074879
..........................................................................................................................................................................CUGGACUGAAGGGUGCUCCCU.................   808.683369
..........................................................................................................................................................................UUGGACUGAAGGGUGCUCCCC.................   40268.98298
..........................................................................................................................................................................UUGGACUGAAUGGUGCUCCCU.................   375.593347
..........................................................................................................................................................................UUGGGCUGAAGGGUGCUCCCU.................   1132.674157
..........................................................................................................................................................................UUGGACUAAAGGGUGCUCCCU.................   548.813267
..........................................................................................................................................................................UUUGACUGAAGGGUGCUCCCU.................   384.243505
..........................................................................................................................................................................AUGGACUGAAGGGUGCUCCCU.................   257.829989
..........................................................................................................................................................................UUGGACUGAAGGGUGCCC....................   268.368677
..........................................................................................................................................................................UUGGACUGAAGGGUGCUCCCUA................   1011.4904
..........................................................................................................................................................................UUGGACUGAAGAGUGCUCCCU.................   269.108516
..........................................................................................................................................................................UUGGAUUGAAGGGUGCUCCCU.................   307.746833
..........................................................................................................................................................................UUGGACUGAAGGGUACUCCCU.................   380.215306
..........................................................................................................................................................................UUGGACUGGAGGGUGCUCCCU.................   1034.462932
..........................................................................................................................................................................UUGGACUGAAGGGUGCACCCU.................   258.047838
..........................................................................................................................................................................UUGGACCGAAGGGUGCUCCCU.................   757.029182
..........................................................................................................................................................................UUAGACUGAAGGGUGCUCCCU.................   306.864608
..........................................................................................................................................................................UUGGACUGAGGGGUGCUCCCU.................   1152.284605
..........................................................................................................................................................................UUGGACUGAAGUGUGCUCCCU.................   370.248244
..........................................................................................................................................................................UUGGACUGAAGGGUGCUCCCA.................   18511.810851
..........................................................................................................................................................................UUGGACUGAAGGGUGCUCCCG.................   10524.289382
..........................................................................................................................................................................UUGGACUUAAGGGUGCUCCCU.................   311.941185
..........................................................................................................................................................................UUGGACUGAAGGGUGCUCACU.................   303.867267
..........................................................................................................................................................................UUGGACUGAAGGGUGCUCCCUG................   318.301184
..........................................................................................................................................................................UUGGACUGAAGGGUGCUACCU.................   311.819303
..........................................................................................................................................................................UUGGACUGAAGGGUGCUUCCU.................   643.494289
.........................................................................................................................................................................CUUGGACUGAAGGGUGCUCC...................   770.275733
.........................................................................................................................................................................CUUGGACUGAAGGGUGCUCCC..................   2109.078708
.........................................................................................................................................................................CUUGGACUGAAGGGUGCUCCCUC................   673.668376
.........................................................................................................................................................................CUUGGACUGAAGGGUGCUCCCU.................   27007.61004
.........................................................................................................................................................................CUUGGACUGAAGGGUGCUCCU..................   452.156451
.........................................................................................................................................................................CUUGGACUGAAGGGUGCUCCCUU................   1866.443918
.........................................................................................................................................................................CUUGGACUGAAGGGUGCUCCCG.................   719.368885
.........................................................................................................................................................................CUUGGACUGAAGGGUGCUCCCC.................   2373.306995
.........................................................................................................................................................................CUUGGACUGAAGGGUGCUCCCA.................   1187.864854
........................................................................................................................................................................GCUUGGACUGAAGGGUGCUCC...................   601.533812
.......................................................................................................................................................................UGCUUGGACUGAAGGGUGCUC....................   388.504112
..................AGAGCGUCCUUCAGUCCACUC.........................................................................................................................................................................   1230.467497
..................AGAGCGUCCUUCAGUCCACU..........................................................................................................................................................................   334.98115
```

zma-MIR390a

Chromosome: chr1
Start: 298107380
End: 298107590
Strand: +
Strand bias: 1.0
Abundance bias: 0.99
MFE: -102.80

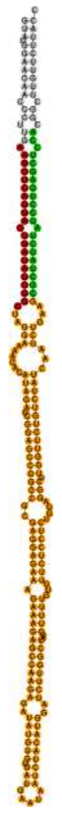

```
                 Mature                                                                                                                                                                  Star
GUAUGGAACAACCCUUGAAGCUCAGGAGGGAUAGCGCCUAGGAAAACACAUAGAUCGGAGCCGGCCGCGAGGAGAAAGAAAGAGCCAAGAGAUAUGUGUGAUGAAUAAUCUACAUGGAUCUUGGCUAGCUUUUGUGUUCUCCUCUGGAGGCGUGGUUCUGUCUAUCAAUCUGAAGCGCUAUCUAUCCUGAGCUCCACGGCUUGUUCUUACC 56195.9
(((.(((((((.((.((.(((((((((.(((((((((...(((......(((((.(((((((((((..((((((((.((((((((((((..((((((.(((.....)))))))))..))))))))..)))))...))))))))....))).)))))))))))))...)))...)))))))))).))))))))).)).)).)))))))))).
.................AAGCUCAGGAGGGAUAGCGCC............................................................................................................................................................................ 39037.4362
...............................................................................................................................................................................CGCUAUCUAUCCUGAGCUCCA............... 1628.131407
...............UGAAGCUCAGGAGGGAUAGCG.............................................................................................................................................................................. 23.394024
.................AAGCUCAGGAGGGAUAGC............................................................................................................................................................................... 10.81961
.................AAGCUCAGGAGGGAUAGCGC............................................................................................................................................................................. 1994.838807
.................AAGCUCAGGAGGGAUAGCGCCU........................................................................................................................................................................... 756.491892
.................AAGCUCAGGAGGGAUAGCG.............................................................................................................................................................................. 133.809645
.................ACGCUCAGGAGGGAUAGCGCC............................................................................................................................................................................ 8.26158
.................AAGCUCAGGAGGGCUAGCGCC............................................................................................................................................................................ 42.38484
.................AAGCUCAGGAGGGAUAUCGCC............................................................................................................................................................................ 9.625438
.................AAGCUCAGGAGGGAUAGAGCC............................................................................................................................................................................ 22.124713
.................GAGCUCAGGAGGGAUAGCGCC............................................................................................................................................................................ 17.399092
.................AAGCUCAGGAGGGAUAGCGU............................................................................................................................................................................. 16.707204
.................AAGCUCGGGAGGGAUAGCGCC............................................................................................................................................................................ 11.720566
.................AAGCUCAGGAGGGAUAGCUCC............................................................................................................................................................................ 6.233038
.................AAGUUCAGGAGGGAUAGCGCC............................................................................................................................................................................ 7.21826
.................AAGCUCAGGAUGGAUAGCGCC............................................................................................................................................................................ 16.100063
.................AGGCUCAGGAGGGAUAGCGCC............................................................................................................................................................................ 7.746143
.................AAGCUCAGGAGGGAUCGCGCC............................................................................................................................................................................ 11.71667
.................AAGCUCAGUAGGGAUAGCGCC............................................................................................................................................................................ 9.20278
.................AAUCUCAGGAGGGAUAGCGCC............................................................................................................................................................................ 33.357205
.................AAGCCCAGGAGGGAUAGCGCC............................................................................................................................................................................ 8.464297
.................AAGCUCAGGAGGGAUGGCGCC............................................................................................................................................................................ 6.651109
.................AAGCUCAGGAGGGGUAGCGCC............................................................................................................................................................................ 6.90973
.................AAGCUCAGGAGGGAUAGCGAC............................................................................................................................................................................ 18.125576
.................AAGCUCAGGAGGGAUAGCGUC............................................................................................................................................................................ 10.78326
.................AAGCUCCGGAGGGAUAGCGCC............................................................................................................................................................................ 21.173419
.................AAGCUCAGGAGGGAUAGCGCCA........................................................................................................................................................................... 87.046562
.................AAGCUCAGGGGGGAUAGCGCC............................................................................................................................................................................ 12.16278
.................AAGCUCAUGAGGGAUAGCGCC............................................................................................................................................................................ 10.0331
.................AAGCUCAGGAGGGAUAGUGCC............................................................................................................................................................................ 11.765319
.................AUGCUCAGGAGGGAUAGCGCC............................................................................................................................................................................ 15.2311
.................AAGCUCAGGAGGGAUAGCGCU............................................................................................................................................................................ 811.830975
.................AAGCUCAGGAGGGAUAGCGCA............................................................................................................................................................................ 452.73559
..................AGCUCAGGAGGGAUAGCGC.............................................................................................................................................................................. 8.725822
..................AGCUCAGGAGGGAUAGCGCC............................................................................................................................................................................. 126.090765
..................AGCUCAGGAGGGAUAGCGCCU............................................................................................................................................................................ 649.414323
..............................................................................................................................................................................GCGCUAUCUAUCCUGAGCUCC................ 119.94439
...............................................................................................................................................................................CGCUAUCUAUCCUGAGCUCC................ 4113.383148
...............................................................................................................................................................................CGCUAUCUAUCCUGAGCUC................. 51.637613
...............................................................................................................................................................................CGCUAUCUAUCCUGAGCU.................. 35.39723
...............................................................................................................................................................................CGCUAUCUAUCCUGAGCUCCU............... 29.100641
...............................................................................................................................................................................CGCUAUCUAUCCUGAGCUCCG............... 7.098721
...............................................................................................................................................................................CGCUAUCUAUCCUGAGCUCU................ 499.385856
...............................................................................................................................................................................CGCUAUCUAUCCUGAGCUCA................ 137.738258
................................................................................................................................................................................GCUAUCUAUCCUGAGCUC................. 195.160805
................................................................................................................................................................................GCUAUCUAUCCUGAGCUCC................ 1819.033498
................................................................................................................................................................................GCUAUCUAUCCUGAGCUCCA............... 298.712669
................................................................................................................................................................................GCUAUCUAUCCUGAGCUU................. 36.16525
................................................................................................................................................................................GCUAUCUAUCCUGAGCUA................. 11.56536
................................................................................................................................................................................GCUAUCUAUCCUGAGCUCA................ 168.672843
................................................................................................................................................................................GCUAUCUAUCCUGAGCUCU................ 1537.058564
................................................................................................................................................................................GCUAUCUAUCCUGAGCUCG................ 30.91665
................................................................................................................................................................................GCUAUCUAUCCUGAGCUCCU............... 21.623644
.................................................................................................................................................................................CUAUCUAUCCUGAGCUCCA............... 44.391434
.................................................................................................................................................................................CUAUCUAUCCUGAGCUCC................ 193.499583
.................................................................................................................................................................................CUAUCUAUCCUGAGCUCA................ 17.204364
.................................................................................................................................................................................CUAUCUAUCCUGAGCUCU................ 63.076659
```

zma-MIR390b

```
Chromosome:    chr5
Start:         2846311
End:           2846532
Strand:        +
Strand bias:   1.0
Abundance bias:0.989
MFE:           -117.10
```

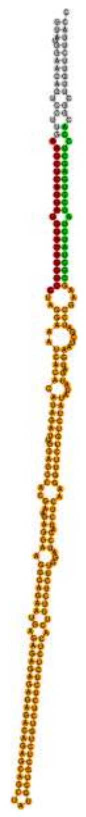

```
                  Mature                                                                                                                                                                     Star
GUAUGGAACAGUCCUUGAAGCUCAGGAGGGAUAGCGCCUAGGAAAUCACACAUAGAUUGGAGCCACGAAGAGGAAGGGAGAAUGAGAGAGAGAGAGACAGAGCAGCUAUGCUGCUCUGUCUCUCUCUCUCACUUUCUCUUGGAUCCAUCUCAAGGUUCUGUCUAUAUAUGAUGAAUCGAUCCGAAGCGCUAUCUAUCCUGAGCUCCACGGCUUGUUCUUACC   56199.3
(((.(((((((.((.((.(((((((((.(((((((((...(((..(((((.((((((.((((((..((.(((((.(((((((.(.((((((((((((((((((((...))))))))))))))))))))))).).)))))))...))).))))..))))))))))))...)).))).....)))...))))))))).))))))))).)).)).))))))))).
.................AAGCUCAGGAGGGAUAGCGCC........................................................................................................................................................................................   39037.4362
..........................................................................................................................................................................................CGCUAUCUAUCCUGAGCUCCA...............   1628.131407
...............UGAAGCUCAGGAGGGAUAGCG..........................................................................................................................................................................................   23.394024
.................AAGCUCAGGAGGGAUAGC...........................................................................................................................................................................................   10.81961
.................AAGCUCAGGAGGGAUAGCGC.........................................................................................................................................................................................   1994.838807
.................AAGCUCAGGAGGGAUAGCGCCU.......................................................................................................................................................................................   756.491892
.................AAGCUCAGGAGGGAUAGCG..........................................................................................................................................................................................   133.809645
.................ACGCUCAGGAGGGAUAGCGCC........................................................................................................................................................................................   8.26158
.................AAGCUCAGGAGGGCUAGCGCC........................................................................................................................................................................................   42.38484
.................AAGCUCAGGAGGGAUAUCGCC........................................................................................................................................................................................   9.625438
.................AAGCUCAGGAGGGAUAGAGCC........................................................................................................................................................................................   22.124713
.................GAGCUCAGGAGGGAUAGCGCC........................................................................................................................................................................................   17.399092
.................AAGCUCAGGAGGGAUAGCGU.........................................................................................................................................................................................   16.707204
.................AAGCUCGGGAGGGAUAGCGCC........................................................................................................................................................................................   11.720566
.................AAGCUCAGGAGGGAUAGCUCC........................................................................................................................................................................................   6.233038
.................AAGUUCAGGAGGGAUAGCGCC........................................................................................................................................................................................   7.21826
.................AAGCUCAGGAUGGAUAGCGCC........................................................................................................................................................................................   16.100063
.................AGGCUCAGGAGGGAUAGCGCC........................................................................................................................................................................................   7.746143
.................AAGCUCAGGAGGGAUCGCGCC........................................................................................................................................................................................   11.71667
.................AAGCUCAGUAGGGAUAGCGCC........................................................................................................................................................................................   9.20278
.................AAUCUCAGGAGGGAUAGCGCC........................................................................................................................................................................................   33.357205
.................AAGCCCAGGAGGGAUAGCGCC........................................................................................................................................................................................   8.464297
.................AAGCUCAGGAGGGAUGGCGCC........................................................................................................................................................................................   6.651109
.................AAGCUCAGGAGGGGUAGCGCC........................................................................................................................................................................................   6.90973
.................AAGCUCAGGAGGGAUAGCGAC........................................................................................................................................................................................   18.125576
.................AAGCUCAGGAGGGAUAGCGUC........................................................................................................................................................................................   10.78326
.................AAGCUCCGGAGGGAUAGCGCC........................................................................................................................................................................................   21.173419
.................AAGCUCAGGAGGGAUAGCGCCA.......................................................................................................................................................................................   87.046562
.................AAGCUCAGGGGGGAUAGCGCC........................................................................................................................................................................................   12.16278
.................AAGCUCAUGAGGGAUAGCGCC........................................................................................................................................................................................   10.0331
.................AAGCUCAGGAGGGAUAGUGCC........................................................................................................................................................................................   11.765319
.................AUGCUCAGGAGGGAUAGCGCC........................................................................................................................................................................................   15.2311
.................AAGCUCAGGAGGGAUAGCGCU........................................................................................................................................................................................   811.830975
.................AAGCUCAGGAGGGAUAGCGCA........................................................................................................................................................................................   452.73559
..................AGCUCAGGAGGGAUAGCGC.........................................................................................................................................................................................   8.725822
..................AGCUCAGGAGGGAUAGCGCC........................................................................................................................................................................................   126.090765
..................AGCUCAGGAGGGAUAGCGCCU.......................................................................................................................................................................................   649.414323
.........................................................................................................................................................................................GCGCUAUCUAUCCUGAGCUCC................   119.94439
..........................................................................................................................................................................................CGCUAUCUAUCCUGAGCUCC................   4113.383148
..........................................................................................................................................................................................CGCUAUCUAUCCUGAGCUC.................   51.637613
..........................................................................................................................................................................................CGCUAUCUAUCCUGAGCU..................   35.39723
..........................................................................................................................................................................................CGCUAUCUAUCCUGAGCUCCU...............   29.100641
..........................................................................................................................................................................................CGCUAUCUAUCCUGAGCUCCG...............   7.098721
..........................................................................................................................................................................................CGCUAUCUAUCCUGAGCUCU................   499.385856
..........................................................................................................................................................................................CGCUAUCUAUCCUGAGCUCA................   137.738258
...........................................................................................................................................................................................GCUAUCUAUCCUGAGCUC.................   195.160805
...........................................................................................................................................................................................GCUAUCUAUCCUGAGCUCC................   1819.033498
...........................................................................................................................................................................................GCUAUCUAUCCUGAGCUCCA...............   298.712669
...........................................................................................................................................................................................GCUAUCUAUCCUGAGCUU.................   36.16525
...........................................................................................................................................................................................GCUAUCUAUCCUGAGCUA.................   11.56536
...........................................................................................................................................................................................GCUAUCUAUCCUGAGCUCA................   168.672843
...........................................................................................................................................................................................GCUAUCUAUCCUGAGCUCU................   1537.058564
...........................................................................................................................................................................................GCUAUCUAUCCUGAGCUCG................   30.91665
...........................................................................................................................................................................................GCUAUCUAUCCUGAGCUCCU...............   21.623644
............................................................................................................................................................................................CUAUCUAUCCUGAGCUCCA...............   44.391434
............................................................................................................................................................................................CUAUCUAUCCUGAGCUCC................   193.499583
............................................................................................................................................................................................CUAUCUAUCCUGAGCUCA................   17.204364
............................................................................................................................................................................................CUAUCUAUCCUGAGCUCU................   63.076659
```

zma-MIR393a

Chromosome: chr2
Start: 738727
End: 738847
Strand: +
Strand bias: 1.0
Abundance bias:1.0
MFE: -58.90

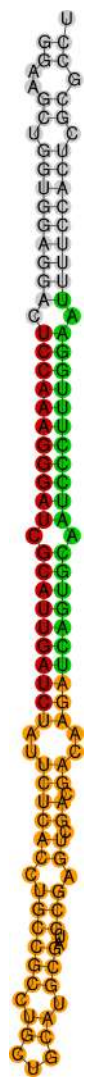

```
                  Mature                                                          Star
GGAAGCUGGUGGAGGACUCCAAAGGGAUCGCAUUGAUCUAUUCUCACCUGCCGCCUGCUGCAUGCGAUGCGAGUCGACGACAAGAUCAGUGCAAUCCCUUUGGAAUUUUCCACUCGCGCCU    79620.
((..((.(((((((((.((((((((((.(((((((((..((((((.((((((.((...)).)))..))).)).)).)).))..))))))))).)))))))))).)))))))).)).)).
.................UCCAAAGGGAUCGCAUUGAUC...................................................................................    35196.123795
.....................................................................................UCAGUGCAAUCCCUUUGGAAU...............    15065.4065
...............ACUCCAAAGGGAUCGCAUUGA.....................................................................................    6.640428
................CUCCAAAGGGAUCGCAUUGAUC...................................................................................    4010.085761
................CUCCAAAGGGAUCGCAUUGAU....................................................................................    16652.362984
................CUCCAAAGGGAUCGCAUUGAUCU..................................................................................    92.499009
................CUCCAAAGGGAUCGCAUUGAUCUA.................................................................................    75.210618
................CUCCAAAGGGAUCGCAUUGA.....................................................................................    379.701453
................CUCCAAAGGGAUCGCAUUG......................................................................................    78.336728
................CUCCAAAGGGAUCGCAUCGAU....................................................................................    12.389717
................CUCCAAAGGGAUUGCAUUGAU....................................................................................    5.126617
................CUCCAAAGGGAUCGCAUUGAUU...................................................................................    303.662694
................CUCCAAAGGGAUCGCAUUGAUA...................................................................................    146.306926
................CUCCAAAGGGAUCGCAUUGAUG...................................................................................    98.395578
................CUCCAAAGGGAUCGCAUUGAUUU..................................................................................    92.033215
................CUCCAAAGGGAUCGCAUUGAC....................................................................................    82.78585
................CUCCAAAGGGAUCGCAUUGAA....................................................................................    12.10799
................CUCCAAAGGGAUCGCAUUGAG....................................................................................    14.203506
................CUCCAAAGGGAUCGCAUUGAUCA..................................................................................    6.127536
................CUCCAAAGGGAUCGCAUUGGU....................................................................................    30.300269
................CUCCAAAGGGAUCACAUUGAU....................................................................................    5.598433
................CUCCAAAGGGAUCGCGUUGAU....................................................................................    23.674594
................CUCCAAAGGGAUCGCACUGAU....................................................................................    14.145071
................CUCCAAAGGGGUCGCAUUGAU....................................................................................    22.738671
................CUCCAAAGGGACCGCAUUGAU....................................................................................    13.071002
................CUCCAAAGGGAUCGCAUUGU.....................................................................................    71.804015
.................UCCAAAGGGAUCGCAUUGA.....................................................................................    15.526491
.................UCCAAAGGGAUCGCAUUGAUCU..................................................................................    146.724514
.................UCCAAAGGGAUCGCAUUGAU....................................................................................    1376.637276
.................UCCAAACGGAUCGCAUUGAUC...................................................................................    20.67167
.................UCCAAAGGGAUCGCAUUGAUCA..................................................................................    16.101364
.................UCCAAAGGGAUCGCAUUGGUC...................................................................................    70.862716
.................UCCGAAGGGAUCGCAUUGAUC...................................................................................    62.021855
.................CCCAAAGGGAUCGCAUUGAUC...................................................................................    71.228701
.................UCAAAAGGGAUCGCAUUGAUC...................................................................................    15.337147
.................UCCAAAGGGAUCGUAUUGAUC...................................................................................    26.324575
.................UCCAAAGUGAUCGCAUUGAUC...................................................................................    35.110721
.................UCGAAAGGGAUCGCAUUGAUC...................................................................................    6.250392
.................UCCAAAAGGAUCGCAUUGAUC...................................................................................    17.877483
.................UCCAAAGGGAUCGCAUCGAUC...................................................................................    53.654061
.................UCCAAAGAGAUCGCAUUGAUC...................................................................................    6.799802
.................ACCAAAGGGAUCGCAUUGAUC...................................................................................    16.218618
.................UCUAAAGGGAUCGCAUUGAUC...................................................................................    13.57551
.................UUCAAAGGGAUCGCAUUGAUC...................................................................................    23.961394
.................GCCAAAGGGAUCGCAUUGAUC...................................................................................    10.173493
.................UCCAAAGGGAUCGCAUUAAUC...................................................................................    16.674253
.................UCCAAGGGGAUCGCAUUGAUC...................................................................................    69.737008
.................UCCAAAGGGAUCGCAUUUAUC...................................................................................    37.431207
.................UCCAAAGGGAUCUCAUUGAUC...................................................................................    6.658281
.................UCCAAAGGGAUCGCACUGAUC...................................................................................    44.842826
.................UCCAAAGGGAUCGCAUUGAUUU..................................................................................    32.863212
.................UCCAAAGGGACCGCAUUGAUC...................................................................................    53.452136
.................UCCAAAGGGAUCACAUUGAUC...................................................................................    32.615166
.................UCCAGAGGGAUCGCAUUGAUC...................................................................................    48.878536
.................UCCAAAGGAAUCGCAUUGAUC...................................................................................    8.328224
.................UCCAAAGGGGUCGCAUUGAUC...................................................................................    65.828148
.................UCCAAAGGGAUCGCAUUGAUA...................................................................................    56.83887
.................UCCAAAGGGAUCGCAUUGAUU...................................................................................    231.195587
.................UCCAAAGGGAUCGCAUUGACC...................................................................................    54.835976
.................UCCAAAGGGAUCGCAUUCAUC...................................................................................    17.243853
.................UCCAAAGGGAUCGCGUUGAUC...................................................................................    53.121371
.................UCCAAAGGGAUUGCAUUGAUC...................................................................................    21.686731
..................CCAAAGGGAUCGCAUUGAUC...................................................................................    159.78583
..................CCAAAGGGAUCGCAUUGAU....................................................................................    8.712625
...................CAAAGGGAUCGCAUUGAU....................................................................................    16.349491
...................CAAAGGGAUCGCAUUGAUC...................................................................................    899.26815
...................................................................................GAUCAGUGCAAUCCCUUUGGAAUU..............    80.943196
.....................................................................................UCAGUGCAAUCCCUUUGGA.................    98.658589
.....................................................................................UCAGUGCAAUCCCUUUGGAA................    298.525417
.....................................................................................UCAGUGCAAUCCCUUUGGAAUU..............    1247.347239
.....................................................................................UCAGUGCCAUCCCUUUGGAAU...............    7.120944
.....................................................................................UCCGUGCAAUCCCUUUGGAAU...............    5.565692
.....................................................................................UCAGUGCAAUUCCUUUGGAAU...............    11.055181
.....................................................................................UCAGUGCACUCCCUUUGGAAU...............    5.82593
.....................................................................................UCAGUUCAAUCCCUUUGGAAU...............    8.453816
.....................................................................................UCGGUGCAAUCCCUUUGGAAU...............    11.796716
.....................................................................................CCAGUGCAAUCCCUUUGGAAU...............    11.446293
.....................................................................................UCAGUGCAAUCCCUUUGGAAA...............    6.827545
.....................................................................................UUAGUGCAAUCCCUUUGGAAU...............    15.744956
.....................................................................................UCAGUGCAAUCCCUUUGGAAG...............    5.8054
.....................................................................................UCAGUGCAAUCCCUUUGGAAC...............    91.93447
.....................................................................................UCAGUGAAAUCCCUUUGGAAU...............    10.697698
.....................................................................................UCAGUGCGAUCCCUUUGGAAU...............    20.383351
.....................................................................................UCAGUGUAAUCCCUUUGGAAU...............    8.37935
.....................................................................................UCAGUGCAAUCUCUUUGGAAU...............    18.064489
.....................................................................................UCAGCGCAAUCCCUUUGGAAU...............    18.678106
.....................................................................................UCAGUGCAAUCCUUUUGGAAU...............    9.897207
.....................................................................................UCAGUGCAGUCCCUUUGGAAU...............    9.348233
.....................................................................................UCAGUGCAACCCCUUUGGAAU...............    5.058099
.....................................................................................UCAGUGCAAUCCCUUUGGAGU...............    18.005926
.....................................................................................UCAGUGCAAUCCCUUUGGGAU...............    18.153393
.....................................................................................UCAUUGCAAUCCCUUUGGAAU...............    36.539988
.....................................................................................UCAGUGCAAUCCCCUUGGAAU...............    15.8965
......................................................................................CAGUGCAAUCCCUUUGGAAU...............    123.385724
......................................................................................CAGUGCAAUCCCUUUGGAA................    4.766222
......................................................................................CAGUGCAAUCCCUUUGGAAUU..............    629.031461
.......................................................................................AGUGCAAUCCCUUUGGAAUU..............    151.874464
.......................................................................................AGUGCAAUCCCUUUGGAAU...............    33.692112
```

zma-MIR393b

```
Chromosome:    chr3
Start:         17997181
End:           17997319
Strand:        +
Strand bias:   1.0
Abundance bias:1.0
MFE:           -69.30
```

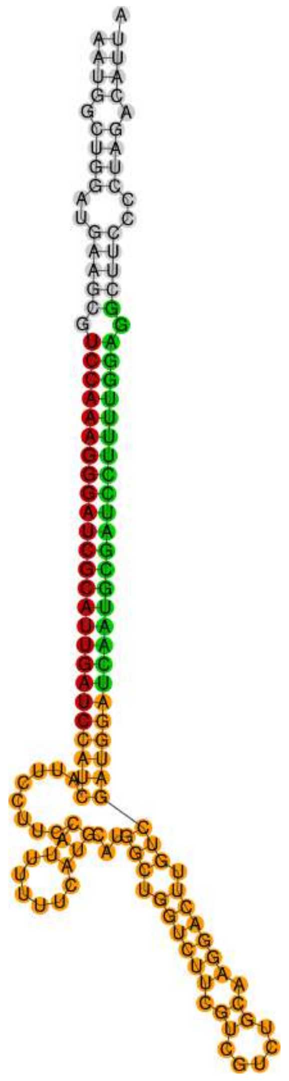

```
                     Mature                                                                   Star
AAUGGCUGGAUGAAGCGUCCAAAGGGAUCGCAUUGAUCCAUCAUUCCUUCCAUUUUUUCAUGCAUGGCUGGUCUUCGUCGUCUGCAAGGACUUGUCGAUGGAUCAAUGCGAUCCUUUUGGAGGCUUCCCCUAGACAUUA   39850.2
((((.((((..(((((.(((((((((((((((((((((((((........(((......)))...(((.((((((.((.....)).)))))).)))))))))))))))))))))))))))).)))))..)))).)))).
.................UCCAAAGGGAUCGCAUUGAUC.....................................................................................................   35196.123795
......................................................................................................UCAAUGCGAUCCUUUUGGAGG................   650.710622
................GUCCAAAGGGAUCGCAUUGAU......................................................................................................   2.811884
.................UCCAAAGGGAUCGCAUUGA.......................................................................................................   15.526491
.................UCCAAAGGGAUCGCAUUGAUCC....................................................................................................   165.529779
.................UCCAAAGGGAUCGCAUUGAU......................................................................................................   1376.637276
.................UCCAAAGGGAUCGCAUUGAUCCA...................................................................................................   7.99068
.................UCCAAAGGGAUCGCAUUGU.......................................................................................................   4.179091
.................UCCAAUGGGAUCGCAUUGAUC.....................................................................................................   0.670018
.................UCCAACGGGAUCGCAUUGAUC.....................................................................................................   1.83139
.................UCCAAACGGAUCGCAUUGAUC.....................................................................................................   20.67167
.................UCCAAAGGGAUCGCAUUGAUCA....................................................................................................   16.101364
.................UCCAAAGGGAUCGCAUUGGUC.....................................................................................................   70.862716
.................UCCGAAGGGAUCGCAUUGAUC.....................................................................................................   62.021855
.................UCCAAAGGUAUCGCAUUGAUC.....................................................................................................   0.644434
.................UCCCAAGGGAUCGCAUUGAUC.....................................................................................................   3.91656
.................UCCAAAGGGAUCGCCUUGAUC.....................................................................................................   2.88378
.................UCCAAAUGGAUCGCAUUGAUC.....................................................................................................   1.478134
.................UCCAAAGGGAUAGCAUUGAUC.....................................................................................................   0.815559
.................CCCAAAGGGAUCGCAUUGAUC.....................................................................................................   71.228701
.................UCAAAAGGGAUCGCAUUGAUC.....................................................................................................   15.337147
.................UCCAAAGGGAUCGCAUUGAAC.....................................................................................................   0.611669
.................UCCAAAGGGAUCGCAUUGAC......................................................................................................   0.563734
.................UCCAAAGGGAUCGUAUUGAUC.....................................................................................................   26.324575
.................UCCAAAGUGAUCGCAUUGAUC.....................................................................................................   35.110721
.................UCCAAAGGGAUCGCUUUGAUC.....................................................................................................   1.32857
.................UCGAAAGGGAUCGCAUUGAUC.....................................................................................................   6.250392
.................UCCAAAGCGAUCGCAUUGAUC.....................................................................................................   3.979902
.................UCCAAAAGGAUCGCAUUGAUC.....................................................................................................   17.877483
.................UCCAAAGGGAUCGCAUCGAUC.....................................................................................................   53.654061
.................UCCAAAGGGAGCGCAUUGAUC.....................................................................................................   1.364538
.................UCCUAAGGGAUCGCAUUGAUC.....................................................................................................   1.370931
.................UCCAAAGAGAUCGCAUUGAUC.....................................................................................................   6.799802
.................ACCAAAGGGAUCGCAUUGAUC.....................................................................................................   16.218618
.................UCUAAAGGGAUCGCAUUGAUC.....................................................................................................   13.57551
.................UUCAAAGGGAUCGCAUUGAUC.....................................................................................................   23.961394
.................GCCAAAGGGAUCGCAUUGAUC.....................................................................................................   10.173493
.................UCCAAAGGGAUCGCAUUAAUC.....................................................................................................   16.674253
.................UCCAAGGGGAUCGCAUUGAUC.....................................................................................................   69.737008
.................UCCAAAGGGAUCGCAUUUAUC.....................................................................................................   37.431207
.................UCCAAAGGGAUCUCAUUGAUC.....................................................................................................   6.658281
.................UCCAAAGGGAUCGCACUGAUC.....................................................................................................   44.842826
.................UCCAAAGGGAUCCCAUUGAUC.....................................................................................................   2.801781
.................UCCAAAGGGACCGCAUUGAUC.....................................................................................................   53.452136
.................UCCAAAGGGAUCACAUUGAUC.....................................................................................................   32.615166
.................UCCAUAGGGAUCGCAUUGAUC.....................................................................................................   1.959234
.................UCCAGAGGGAUCGCAUUGAUC.....................................................................................................   48.878536
.................UCCAAAGGAAUCGCAUUGAUC.....................................................................................................   8.328224
.................UCCAAAGGGGUCGCAUUGAUC.....................................................................................................   65.828148
.................UCCAAAGGGAUCGCAUUGAUA.....................................................................................................   56.83887
.................UCCAAAGGGAUCGCAUUGAUU.....................................................................................................   231.195587
.................UCCAAAGGGAUCGCAUUGACC.....................................................................................................   54.835976
.................UCCAAAGGGAACGCAUUGAUC.....................................................................................................   0.605866
.................UCCAAAGGGAUCGCAUUCAUC.....................................................................................................   17.243853
.................UCCAAAGGGAUCGCGUUGAUC.....................................................................................................   53.121371
.................UCCAAAGGGAUUGCAUUGAUC.....................................................................................................   21.686731
..................CCAAAGGGAUCGCAUUGAUC.....................................................................................................   159.78583
..................CCAAAGGGAUCGCAUUGAU......................................................................................................   8.712625
...................CAAAGGGAUCGCAUUGAUCC....................................................................................................   0.868372
...................CAAAGGGAUCGCAUUGAU......................................................................................................   16.349491
...................CAAAGGGAUCGCAUUGAUC.....................................................................................................   899.26815
.....................................................................................................AUCAAUGCGAUCCUUUUGGAG.................   1.56976
......................................................................................................UCAAUGCGAUCCUUUUGGAG.................   21.832278
......................................................................................................UCAAUGCGAUCCUUUUGUAGG................   2.09301
......................................................................................................UCAAUGCGAUCCUUUUGGAGA................   1.83139
........................................................................................................AAUGCGAUCCUUUUGGAGG................   5.725649
........................................................................................................AAUGCGAUCCUUUUGGAGGC...............   0.371561
```

zma-MIR393c

```
Chromosome:    chr10
Start:         149451794
End:           149451924
Strand:        -
Strand bias:   1.0
Abundance bias:1.0
MFE:           -61.90
```

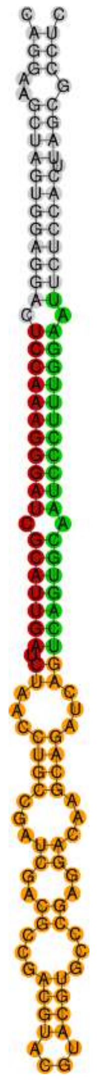

```
                   Mature                                                                       Star
CAGGAAGCUAGUGGAGGACUCCAAAGGGAUCGCAUUGAUCUAACCUGCCGAUCGACGCCGACGUACGUACGUGCCCGAGGACAAGCAGAUCAGUCAGUGCAAUCCCUUUGGAAUUCUCCACUUAGCGCCUC    79566.0
.(((..((((((((((((.((((((((((.(((((((.((...((((...((..((...((((....))))...))..))...))))...)))))))))).))))))))))).)))))))).)))).))).
...................UCCAAAGGGAUCGCAUUGAUC...........................................................................................    35196.123795
.............................................................................................UCAGUGCAAUCCCUUUGGAAU.................    15065.4065
.................ACUCCAAAGGGAUCGCAUUGA.............................................................................................    6.640428
..................CUCCAAAGGGAUCGCAUUGAUC...........................................................................................    4010.085761
..................CUCCAAAGGGAUCGCAUUGAU............................................................................................    16652.362984
..................CUCCAAAGGGAUCGCAUUGAUCU..........................................................................................    92.499009
..................CUCCAAAGGGAUCGCAUUGAUCUA.........................................................................................    75.210618
..................CUCCAAAGGGAUCGCAUUGA.............................................................................................    379.701453
..................CUCCAAAGGGAUCGCAUUG..............................................................................................    78.336728
..................CUCCAAAGGGAUCGCAUCGAU............................................................................................    12.389717
..................CUCCAAAGGGAUUGCAUUGAU............................................................................................    5.126617
..................CUCCAAAGGGAUCGCAUUGAUU...........................................................................................    303.662694
..................CUCCAAAGGGAUCGCAUUGAUA...........................................................................................    146.306926
..................CUCCAAAGGGAUCGCAUUGAUG...........................................................................................    98.395578
..................CUCCAAAGGGAUCGCAUUGAUUU..........................................................................................    92.033215
..................CUCCAAAGGGAUCGCAUUGAC............................................................................................    82.78585
..................CUCCAAAGGGAUCGCAUUGAA............................................................................................    12.10799
..................CUCCAAAGGGAUCGCAUUGAG............................................................................................    14.203506
..................CUCCAAAGGGAUCGCAUUGAUCA..........................................................................................    6.127536
..................CUCCAAAGGGAUCGCAUUGGU............................................................................................    30.300269
..................CUCCAAAGGGAUCACAUUGAU............................................................................................    5.598433
..................CUCCAAAGGGAUCGCGUUGAU............................................................................................    23.674594
..................CUCCAAAGGGAUCGCACUGAU............................................................................................    14.145071
..................CUCCAAAGGGGUCGCAUUGAU............................................................................................    22.738671
..................CUCCAAAGGGACCGCAUUGAU............................................................................................    13.071002
..................CUCCAAAGGGAUCGCAUUGU.............................................................................................    71.804015
...................UCCAAAGGGAUCGCAUUGA.............................................................................................    15.526491
...................UCCAAAGGGAUCGCAUUGAUCU..........................................................................................    146.724514
...................UCCAAAGGGAUCGCAUUGAU............................................................................................    1376.637276
...................UCCAAACGGAUCGCAUUGAUC...........................................................................................    20.67167
...................UCCAAAGGGAUCGCAUUGAUCA..........................................................................................    16.101364
...................UCCAAAGGGAUCGCAUUGGUC...........................................................................................    70.862716
...................UCCGAAGGGAUCGCAUUGAUC...........................................................................................    62.021855
...................CCCAAAGGGAUCGCAUUGAUC...........................................................................................    71.228701
...................UCAAAAGGGAUCGCAUUGAUC...........................................................................................    15.337147
...................UCCAAAGGGAUCGUAUUGAUC...........................................................................................    26.324575
...................UCCAAAGUGAUCGCAUUGAUC...........................................................................................    35.110721
...................UCGAAAGGGAUCGCAUUGAUC...........................................................................................    6.250392
...................UCCAAAAGGAUCGCAUUGAUC...........................................................................................    17.877483
...................UCCAAAGGGAUCGCAUCGAUC...........................................................................................    53.654061
...................UCCAAAGAGAUCGCAUUGAUC...........................................................................................    6.799802
...................ACCAAAGGGAUCGCAUUGAUC...........................................................................................    16.218618
...................UCUAAAGGGAUCGCAUUGAUC...........................................................................................    13.57551
...................UUCAAAGGGAUCGCAUUGAUC...........................................................................................    23.961394
...................GCCAAAGGGAUCGCAUUGAUC...........................................................................................    10.173493
...................UCCAAAGGGAUCGCAUUAAUC...........................................................................................    16.674253
...................UCCAAGGGGAUCGCAUUGAUC...........................................................................................    69.737008
...................UCCAAAGGGAUCGCAUUUAUC...........................................................................................    37.431207
...................UCCAAAGGGAUCUCAUUGAUC...........................................................................................    6.658281
...................UCCAAAGGGAUCGCACUGAUC...........................................................................................    44.842826
...................UCCAAAGGGAUCGCAUUGAUUU..........................................................................................    32.863212
...................UCCAAAGGGACCGCAUUGAUC...........................................................................................    53.452136
...................UCCAAAGGGAUCACAUUGAUC...........................................................................................    32.615166
...................UCCAGAGGGAUCGCAUUGAUC...........................................................................................    48.878536
...................UCCAAAGGAAUCGCAUUGAUC...........................................................................................    8.328224
...................UCCAAAGGGGUCGCAUUGAUC...........................................................................................    65.828148
...................UCCAAAGGGAUCGCAUUGAUA...........................................................................................    56.83887
...................UCCAAAGGGAUCGCAUUGAUU...........................................................................................    231.195587
...................UCCAAAGGGAUCGCAUUGACC...........................................................................................    54.835976
...................UCCAAAGGGAUCGCAUUCAUC...........................................................................................    17.243853
...................UCCAAAGGGAUCGCGUUGAUC...........................................................................................    53.121371
...................UCCAAAGGGAUUGCAUUGAUC...........................................................................................    21.686731
....................CCAAAGGGAUCGCAUUGAUC...........................................................................................    159.78583
....................CCAAAGGGAUCGCAUUGAU............................................................................................    8.712625
.....................CAAAGGGAUCGCAUUGAU............................................................................................    16.349491
.....................CAAAGGGAUCGCAUUGAUC...........................................................................................    899.26815
...........................................................................................AGUCAGUGCAAUCCCUUUGGA...................    28.643586
.............................................................................................UCAGUGCAAUCCCUUUGGA...................    98.658589
.............................................................................................UCAGUGCAAUCCCUUUGGAA..................    298.525417
.............................................................................................UCAGUGCAAUCCCUUUGGAAUU................    1247.347239
.............................................................................................UCAGUGCCAUCCCUUUGGAAU.................    7.120944
.............................................................................................UCCGUGCAAUCCCUUUGGAAU.................    5.565692
.............................................................................................UCAGUGCAAUUCCUUUGGAAU.................    11.055181
.............................................................................................UCAGUGCACUCCCUUUGGAAU.................    5.82593
.............................................................................................UCAGUUCAAUCCCUUUGGAAU.................    8.453816
.............................................................................................UCGGUGCAAUCCCUUUGGAAU.................    11.796716
.............................................................................................CCAGUGCAAUCCCUUUGGAAU.................    11.446293
.............................................................................................UCAGUGCAAUCCCUUUGGAAA.................    6.827545
.............................................................................................UUAGUGCAAUCCCUUUGGAAU.................    15.744956
.............................................................................................UCAGUGCAAUCCCUUUGGAAG.................    5.8054
.............................................................................................UCAGUGCAAUCCCUUUGGAAC.................    91.93447
.............................................................................................UCAGUGAAAUCCCUUUGGAAU.................    10.697698
.............................................................................................UCAGUGCGAUCCCUUUGGAAU.................    20.383351
.............................................................................................UCAGUGUAAUCCCUUUGGAAU.................    8.37935
.............................................................................................UCAGUGCAAUCUCUUUGGAAU.................    18.064489
.............................................................................................UCAGCGCAAUCCCUUUGGAAU.................    18.678106
.............................................................................................UCAGUGCAAUCCUUUUGGAAU.................    9.897207
.............................................................................................UCAGUGCAGUCCCUUUGGAAU.................    9.348233
.............................................................................................UCAGUGCAACCCCUUUGGAAU.................    5.058099
.............................................................................................UCAGUGCAAUCCCUUUGGAGU.................    18.005926
.............................................................................................UCAGUGCAAUCCCUUUGGGAU.................    18.153393
.............................................................................................UCAUUGCAAUCCCUUUGGAAU.................    36.539988
.............................................................................................UCAGUGCAAUCCCCUUGGAAU.................    15.8965
..............................................................................................CAGUGCAAUCCCUUUGGAAU.................    123.385724
..............................................................................................CAGUGCAAUCCCUUUGGAA..................    4.766222
..............................................................................................CAGUGCAAUCCCUUUGGAAUU................    629.031461
...............................................................................................AGUGCAAUCCCUUUGGAAUU................    151.874464
...............................................................................................AGUGCAAUCCCUUUGGAAU.................    33.692112
```

zma-MIR394a

Chromosome: chr5
Start: 199240965
End: 199241094
Strand: +
Strand bias: 1.0
Abundance bias: 0.991
MFE: -50.30

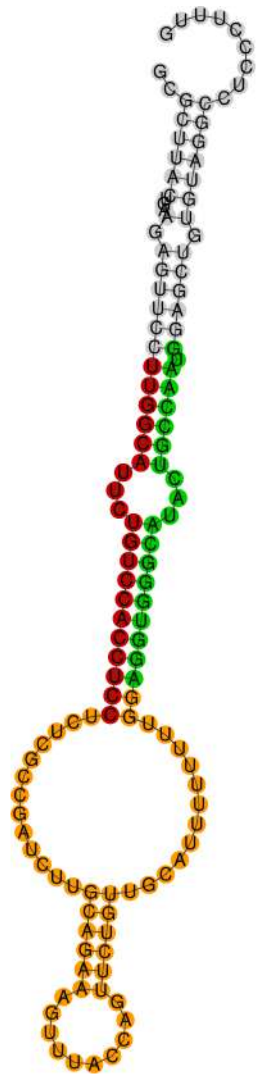

```
                   Mature                                                             Star
GCGCUUACUGAAGAGUUCCUUGGCAUUCUGUCCACCUCCUCUCGCCGAUCUUGCAGAAAGUUUACCAGUUCUGUUGCAUUUUUUUUGGAGGUGGGCAUACUGCCAAUGGAGCUGUGUAGGCCUCCCUUUG    23318.1
..((((((...(.((((((((((((...(((((((((((.............((((((..........))))))............)))))))))))...)))))).)))))).))))))).........
...................UUGGCAUUCUGUCCACCUCC...........................................................................................    20086.785581
.........................................................................................AGGUGGGCAUACUGCCAAUG.....................    0.17779
..................CUUGGCAUUCUGUCCACCUCC...........................................................................................    105.925491
...................UUGGCAUUCUGUCCACCUC............................................................................................    66.734893
...................UUGGCAUUCUGUCCACCU.............................................................................................    7.0708
...................UUGGCAUUCUGUCCACCUCCU..........................................................................................    13.819803
...................AUGGCAUUCUGUCCACCUCC...........................................................................................    4.22877
...................UAGGCAUUCUGUCCACCUCC...........................................................................................    0.501437
...................UGGGCAUUCUGUCCACCUCC...........................................................................................    6.96277
...................UUGGCAUUCUGUCCACCUCUUC.........................................................................................    1.932795
...................UUGGCAUUCUGUCCCCCUCC...........................................................................................    6.156869
...................UUGGCAUUCUGUCCACUUCC...........................................................................................    2.15869
...................UUGGCAUUCUGGCCACCUCC...........................................................................................    0.318481
...................UUGGCAUUCUGUCCACCUUC...........................................................................................    4.405235
...................UCGGCAUUCUGUCCACCUCC...........................................................................................    6.520944
...................UUGGCAUUCUGUCCACCCCC...........................................................................................    4.442118
...................UUGGCAUCCUGUCCACCUCC...........................................................................................    1.441198
...................UUGGCGUUCUGUCCACCUCC...........................................................................................    4.183384
...................UUGGCAUUCUGUCAACCUCC...........................................................................................    1.21014
...................UUGGCAUUCUGUCCGCCUCC...........................................................................................    1.135516
...................UUGGCACUCUGUCCACCUCC...........................................................................................    3.727068
...................UUGGCAUUCUGUCCACCUCU...........................................................................................    1884.580159
...................UUGGCAUUCUGUCCACCUCG...........................................................................................    29.76328
...................UUGGCAUUCUGUCCACCUCUU..........................................................................................    0.934938
...................UUGUCAUUCUGUCCACCUCC...........................................................................................    4.05028
...................UUGGCAUUCUGUCCACCACC...........................................................................................    0.76742
...................UUGGCAUUCUGUCCACCUU............................................................................................    3.22411
...................UUGGCAUUCUGCCCACCUCC...........................................................................................    4.896229
...................UUGGCAUUCCGUCCACCUCC...........................................................................................    3.115138
...................UUGGCAUUCUGUCCACCUCCA..........................................................................................    39.634308
...................CUGGCAUUCUGUCCACCUCC...........................................................................................    1.903409
...................UUGGCAUUCUGUCCACCA.............................................................................................    7.754314
...................UUGGCAUUCUGUCCACCUCA...........................................................................................    682.496986
...................UUUGCAUUCUGUCCACCUCC...........................................................................................    1.540003
...................UUGGCAUUCUGUCCACCGCC...........................................................................................    2.631384
....................UGGCAUUCUGUCCACCUCC...........................................................................................    9.44577
.....................GGCAUUCUGUCCACCUCC...........................................................................................    1.61614
.........................................................................................AGGUGGGCAUACUGCCAAUGGA...................    103.48155
.........................................................................................AGGUGGGCAUACUGCCAAUGG....................    4.916182
```

zma-MIR394b

```
Chromosome:    chr4
Start:         157728376
End:           157728501
Strand:        +
Strand bias:   1.0
Abundance bias:0.999
MFE:           -53.20
```

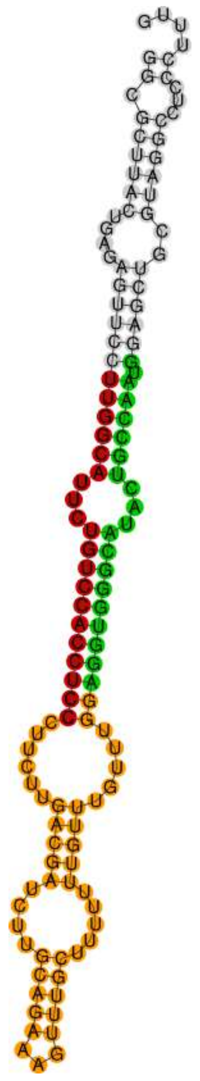

```
                   Mature                                                             Star
GGCGCUUACUGAGAGUUCCUUGGCAUUCUGUCCACCUCCCUUCUUGACGAUCUUGCAGAAAGUUUGCUUUUUUUGUUUGUUUGGAGGUGGGCAUACUGCCAAUGGAGCUGCGUAGGCCUCCCUUUG    23129.7
((.((((((....((((((((((((...(((((((((((......(((((....(((((...))))).....)))))......)))))))))))...)))))).))))))..))))))...))....
...................UUGGCAUUCUGUCCACCUCC.......................................................................................    20086.785581
....................................................................................AGGUGGGCAUACUGCCAAUG......................    0.17779
..................CUUGGCAUUCUGUCCACCUCC.......................................................................................    105.925491
...................UUGGCAUUCUGUCCACCUC........................................................................................    66.734893
...................UUGGCAUUCUGUCCACCUCCC......................................................................................    1.95174
...................UUGGCAUUCUGUCCACCU.........................................................................................    7.0708
...................AUGGCAUUCUGUCCACCUCC.......................................................................................    4.22877
...................UAGGCAUUCUGUCCACCUCC.......................................................................................    0.501437
...................UGGGCAUUCUGUCCACCUCC.......................................................................................    6.96277
...................UUGGCAUUCUGUCCCCCUCC.......................................................................................    6.156869
...................UUGGCAUUCUGUCCACUUCC.......................................................................................    2.15869
...................UUGGCAUUCUGGCCACCUCC.......................................................................................    0.318481
...................UUGGCAUUCUGUCCACCUUC.......................................................................................    4.405235
...................UCGGCAUUCUGUCCACCUCC.......................................................................................    6.520944
...................UUGGCAUUCUGUCCACCCCC.......................................................................................    4.442118
...................UUGGCAUCCUGUCCACCUCC.......................................................................................    1.441198
...................UUGGCGUUCUGUCCACCUCC.......................................................................................    4.183384
...................UUGGCAUUCUGUCAACCUCC.......................................................................................    1.21014
...................UUGGCAUUCUGUCCGCCUCC.......................................................................................    1.135516
...................UUGGCACUCUGUCCACCUCC.......................................................................................    3.727068
...................UUGGCAUUCUGUCCACCUCU.......................................................................................    1884.580159
...................UUGGCAUUCUGUCCACCUCG.......................................................................................    29.76328
...................UUGUCAUUCUGUCCACCUCC.......................................................................................    4.05028
...................UUGGCAUUCUGUCCACCACC.......................................................................................    0.76742
...................UUGGCAUUCUGUCCACCUU........................................................................................    3.22411
...................UUGGCAUUCUGCCCACCUCC.......................................................................................    4.896229
...................UUGGCAUUCCGUCCACCUCC.......................................................................................    3.115138
...................UUGGCAUUCUGUCCACCUCCA......................................................................................    39.634308
...................CUGGCAUUCUGUCCACCUCC.......................................................................................    1.903409
...................UUGGCAUUCUGUCCACCA.........................................................................................    7.754314
...................UUGGCAUUCUGUCCACCUCA.......................................................................................    682.496986
...................UUUGCAUUCUGUCCACCUCC.......................................................................................    1.540003
...................UUGGCAUUCUGUCCACCGCC.......................................................................................    2.631384
....................UGGCAUUCUGUCCACCUCC.......................................................................................    9.44577
.....................GGCAUUCUGUCCACCUCC.......................................................................................    1.61614
....................................................................................AGGUGGGCAUACUGCCAAUGGA....................    103.48155
....................................................................................AGGUGGGCAUACUGCCAAUGG.....................    4.916182
```

zma-MIR395a

```
Chromosome:    chr2
Start:         6288045
End:           6288188
Strand:        -
Strand bias:   1.0
Abundance bias:1.0
MFE:           -78.00
```

```
                                    Star                                                                                   Mature
UGGUUGGUUGUCACCUGGAGUUCUCCUCAAACCACUUCAGUUGCUGCACCUAGCUAGUUUAAUGGCACUGUGCUUGCGUGCAAGCGGCCGGAGCUGUGUGUAGCUAGUGAAGUGUUUGGGGAACUCUGGGUGUCAAUCAGCAA    6922.7
..(((((((.((((((((((((((.(((((.((((((.(.(((((((.(((((.......((((....((((((....)))))))))).))))).))))))).).)))))))))))))))))))))))))).))))))))..
...........................................................................................................UGAAGUGUUUGGGGAACUC.................    4586.749169
...................GUUCUCCUCAAACCACUUCAGU......................................................................................................    212.38762
..............................................................................................................AGUGUUUGGGGAACUCUGG..............    3.44333
.............................................................................................................AAGUGUUUGGGGAACUC.................    4.22703
.............................................................................................................AAGUGUUUGGGGAACUCU................    3.15555
............................................................................................................GAAGUGUUUGGGGAACU..................    43.736
............................................................................................................GAAGUGUUUGGGGAACUC.................    5.1317
...........................................................................................................UGAAGUGUUUGGGGAAC...................    117.318839
...........................................................................................................UGAAGUGUUUGGGGAACUCU................    66.540551
...........................................................................................................UGAAGUGUUUGGGGAACU..................    897.367501
...........................................................................................................UGAAGUGUUUGGGGAAU...................    13.78406
...........................................................................................................UGGAGUGUUUGGGGAACUC.................    3.28276
...........................................................................................................UGAAGUGUUUGGGGAACG..................    9.43161
...........................................................................................................UGAAGUGUUUGGGGAACA..................    48.00465
...........................................................................................................UGAAGUGUUUGGGGAACC..................    111.29543
...........................................................................................................CGAAGUGUUUGGGGAACUC.................    1.74419
...........................................................................................................UGAAGUGUUUGGGGAACUCA................    21.45689
...........................................................................................................UGAAGUGUUUGGGGAACUA.................    173.561085
...........................................................................................................UGAAGUGUUUGGGGAACUU.................    494.126627
...........................................................................................................UGAAGUGUUUGGGGCACUC.................    3.41142
...........................................................................................................UGAAGUGUUUGGGGAACGC.................    7.60419
...........................................................................................................UGAAGUGUUUGGGGGACUC.................    1.74419
...........................................................................................................UGAAGUGUUUGGGGAACCC.................    31.73744
...........................................................................................................UGAAGUGUUUGGGGAACUCC................    17.66074
..........................................................................................................GUGAAGUGUUUGGGGAAC...................    1.94188
..........................................................................................................GUGAAGUGUUUGGGGAACUC.................    8.66896
..........................................................................................................GUGAAGUGUUUGGGGAACU..................    14.84634
...................GUUCUCCUCAAACCACUUC.........................................................................................................    11.37031
...................GUUCUCCUCAAACCACUUCAGC......................................................................................................    5.075248
```

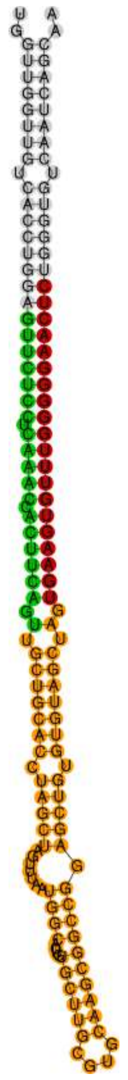

zma-MIR395b

Chromosome: chr2
Start: 6287273
End: 6287446
Strand: -
Strand bias: 1.0
Abundance bias: 0.999
MFE: -64.90

```
                    Mature                                                                                                                   Star
AAUAAAUUAAAGGCCAGUUGUGAAGUGUUUGGGGGAACUCUUGGUAUCAUGAAGCAUUUAGUUAUAAUAAUAUAUGAUGGCCAUAUGGAUACCAUGGGCGAUACAACUUUGAACAAUGUCUGGUGUUACCAUGAGUUCCCUUCAAGCACUUCACAUGAGGCACCAUUCAAAUAA    7366.3
............(((...((((((((((((((((((((((.(((((.(((.(.(((((..(((.(((.........((.(((.(((((...)))))))).))......))))))))))).).))).))))).)))))))).))))))))))))))...)))).............
....................UGAAGUGUUUGGGGGAACUC..............................................................................................................................    4586.749169
......................................................................................................................................GUUCCCUUCAAGCACUUCACA...................    367.525387
...................GUGAAGUGUUUGGGGGAAC........................................................................................................................................    1.94188
...................GUGAAGUGUUUGGGGGAACUC......................................................................................................................................    8.66896
...................GUGAAGUGUUUGGGGGAACU.......................................................................................................................................    14.84634
....................UGAAGUGUUUGGGGGAAC........................................................................................................................................    117.318839
....................UGAAGUGUUUGGGGGAACUCU.....................................................................................................................................    66.540551
....................UGAAGUGUUUGGGGGAACU.......................................................................................................................................    897.367501
....................UGAAGUGUUUGGGGGAAU........................................................................................................................................    13.78406
....................UGGAGUGUUUGGGGGAACUC......................................................................................................................................    3.28276
....................UGAAGUGUUUGGGGGAACG.......................................................................................................................................    9.43161
....................UGAAGUGUUUGGGGGAACA.......................................................................................................................................    48.00465
....................UGAAGUGUUUGGGGGAACC.......................................................................................................................................    111.29543
....................CGAAGUGUUUGGGGGAACUC......................................................................................................................................    1.74419
....................UGAAGUGUUUGGGGGAACUCA.....................................................................................................................................    21.45689
....................UGAAGUGUUUGGGGGAACUA......................................................................................................................................    173.561085
....................UGAAGUGUUUGGGGGAACUU......................................................................................................................................    494.126627
....................UGAAGUGUUUGGGGGCACUC......................................................................................................................................    3.41142
....................UGAAGUGUUUGGGGGAACGC......................................................................................................................................    7.60419
....................UGAAGUGUUUGGGGGGACUC......................................................................................................................................    1.74419
....................UGAAGUGUUUGGGGGAACUUUU....................................................................................................................................    5.28629
....................UGAAGUGUUUGGGGGAACCC......................................................................................................................................    31.73744
....................UGAAGUGUUUGGGGGAACUCC.....................................................................................................................................    17.66074
.....................GAAGUGUUUGGGGGAACU.......................................................................................................................................    43.736
.....................GAAGUGUUUGGGGGAACUC......................................................................................................................................    5.1317
......................AAGUGUUUGGGGGAACUC......................................................................................................................................    4.22703
......................AAGUGUUUGGGGGAACUCU.....................................................................................................................................    3.15555
.....................................................................................................................................AGUUCCCUUCAAGCACUUCACA...................    24.4443
......................................................................................................................................GUUCCCUUCAAGCACUUCA.....................    4.21907
......................................................................................................................................GUUCCCUUCAAGCACUUCACAU..................    1.45641
......................................................................................................................................GUUCCCUUCAAGCACUUC......................    1.45641
......................................................................................................................................GUUCCCUUCAAGCACUUCAC....................    57.451406
......................................................................................................................................GUUCUCUUCAAGCACUUCACA...................    2.73952
......................................................................................................................................GUUCCCUACAAGCACUUCACA...................    202.562794
```

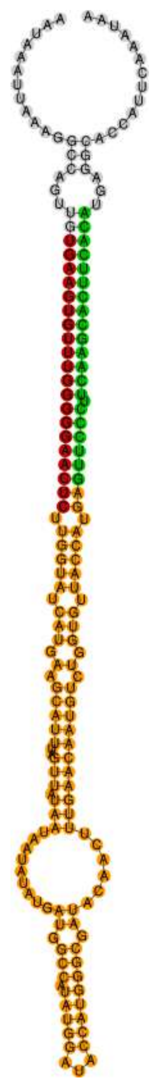

zma-MIR395n

Chromosome: chr10
Start: 145465894
End: 145466164
Strand: +
Strand bias: 1.0
Abundance bias: 0.994
MFE: -119.70

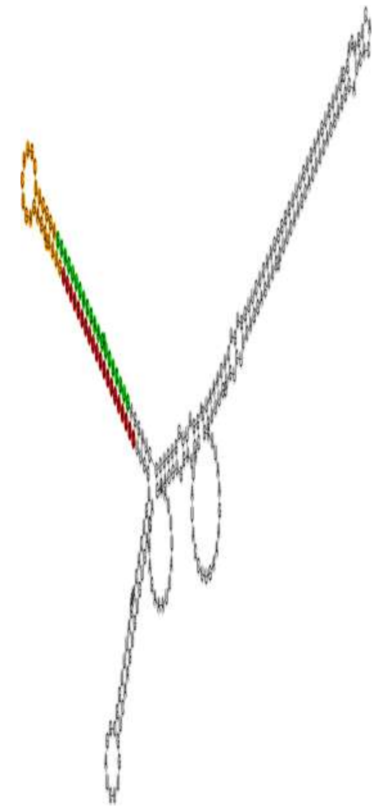

```
                  Star                                 Mature
GGUUUGGUGUCCCUAGGAGUUCUCUACAAGCACUUCACGAGGCUUCGUACUGAGAGCUUUUGUGAAGUGUUUGGGGGAACUCUUGGUGUCACAAAUCAUUUGGCGGAUCUUGGUCAAGUGUGUGACAGAAGAAAUUAUUUAAGGACGUGGAUCCAUAUACCUUGAAAGAAUUAGGUUUGGUGUCUUCAGGAGUUCUCUUCAAGCACUUCACGAGGCAUCACACUGAGAGCUUUUGUGAAGUGUUUGGGGGAACUCUUGGUGUCACAAAUCA 6950.5
((.((.((((((((((((((((((.((((((((((((((((((........)))).))))))))))))))))))))))))))))))(((((((...((((((((........)))))))))))))))..............))))))).)).))..................((((((.((.(.((((((((((.((((((((((((((((((((.(((...)))..)).))))))))))))))))))))))))))))).).)))))))).
..............................................................UGAAGUGUUUGGGGGAACUC............................................................................................................................................................................................. 4586.749169
..................GUUCUCUACAAGCACUUCACG........................................................................................................................................................................................................................................ 19.22097
................................................................AAGUGUUUGGGGGAACUC............................................................................................................................................................................................. 4.22703
................................................................AAGUGUUUGGGGGAACUCU............................................................................................................................................................................................ 3.15555
...............................................................GAAGUGUUUGGGGGAACU.............................................................................................................................................................................................. 43.736
...............................................................GAAGUGUUUGGGGGAACUC............................................................................................................................................................................................. 5.1317
..............................................................UGAAGUGUUUGGGGGAAC............................................................................................................................................................................................... 117.318839
..............................................................UGAAGUGUUUGGGGGAACUCU............................................................................................................................................................................................ 66.540551
..............................................................UGAAGUGUUUGGGGGAACU.............................................................................................................................................................................................. 897.367501
..............................................................UGAAGUGUUUGGGGGAAU............................................................................................................................................................................................... 13.78406
..............................................................UGGAGUGUUUGGGGGAACUC............................................................................................................................................................................................. 3.28276
..............................................................UGAAGUGUUUGGGGGAACG.............................................................................................................................................................................................. 9.43161
..............................................................UGAAGUGUUUGGGGGAACA.............................................................................................................................................................................................. 48.00465
..............................................................UGAAGUGUUUGGGGGAACC.............................................................................................................................................................................................. 111.29543
..............................................................CGAAGUGUUUGGGGGAACUC............................................................................................................................................................................................. 1.74419
..............................................................UGAAGUGUUUGGGGGAACUCA............................................................................................................................................................................................ 21.45689
..............................................................UGAAGUGUUUGGGGGAACUA............................................................................................................................................................................................. 173.561085
..............................................................UGAAGUGUUUGGGGGAACUU............................................................................................................................................................................................. 494.126627
..............................................................UGAAGUGUUUGGGGGCACUC............................................................................................................................................................................................. 3.41142
..............................................................UGAAGUGUUUGGGGGAACGC............................................................................................................................................................................................. 7.60419
..............................................................UGAAGUGUUUGGGGGGACUC............................................................................................................................................................................................. 1.74419
..............................................................UGAAGUGUUUGGGGGAACUUUU.......................................................................................................................................................................................... 5.28629
..............................................................UGAAGUGUUUGGGGGAACCC............................................................................................................................................................................................. 31.73744
..............................................................UGAAGUGUUUGGGGGAACUCC............................................................................................................................................................................................ 17.66074
.............................................................GUGAAGUGUUUGGGGGAAC............................................................................................................................................................................................... 1.94188
.............................................................GUGAAGUGUUUGGGGGAACUC............................................................................................................................................................................................. 8.66896
.............................................................GUGAAGUGUUUGGGGGAACU.............................................................................................................................................................................................. 14.84634
..................GUUCUCUACAAGCACUUCACGA....................................................................................................................................................................................................................................... 5.62794
..................GUUCCCUACAAGCACUUCACG........................................................................................................................................................................................................................................ 4.48371
.................AGUUCUCUACAAGCACUUCACG........................................................................................................................................................................................................................................ 185.495505
```

zma-MIR395f

Chromosome: chr2
Start: 6276814
End: 6276987
Strand: -
Strand bias: 1.0
Abundance bias: 1.0
MFE: -63.50

```
                                    Mature                                                                                                                      Star
GGCUUAAUAAAGGCCAGUUGUGAAGUGUUUGGGGGAACUCUUGGUAUCAUGAAGCAAUUAGUUAUAAUAAUAUAUUAUGGCCAUAUGGAUACCAUGGGCAAUACAACUUUGAACAAUGUCUGGUGUUACCAUGAGUUCCCUUCAAGCACUUCACAUGAGGCACCAUUCAAUAAC    7358.6
(((((.....)))))...((((((((((((((((((((((.(((((.(((.(.(((......(((((((...)))))))(((.(((((...)))))))).................))).).))).))))).)))))))).))))))))))))))((((......)))).....
....................UGAAGUGUUUGGGGGAACUC......................................................................................................................................    4586.749169
......................................................................................................................................GUUCCCUUCAAGCACUUCACA...................    367.525387
...................GUGAAGUGUUUGGGGGAAC........................................................................................................................................    1.94188
...................GUGAAGUGUUUGGGGGAACUC......................................................................................................................................    8.66896
...................GUGAAGUGUUUGGGGGAACU.......................................................................................................................................    14.84634
....................UGAAGUGUUUGGGGGAAC........................................................................................................................................    117.318839
....................UGAAGUGUUUGGGGGAACUCU.....................................................................................................................................    66.540551
....................UGAAGUGUUUGGGGGAACU.......................................................................................................................................    897.367501
....................UGAAGUGUUUGGGGGAAU........................................................................................................................................    13.78406
....................UGGAGUGUUUGGGGGAACUC......................................................................................................................................    3.28276
....................UGAAGUGUUUGGGGGAACG.......................................................................................................................................    9.43161
....................UGAAGUGUUUGGGGGAACA.......................................................................................................................................    48.00465
....................UGAAGUGUUUGGGGGAACC.......................................................................................................................................    111.29543
....................CGAAGUGUUUGGGGGAACUC......................................................................................................................................    1.74419
....................UGAAGUGUUUGGGGGAACUCA.....................................................................................................................................    21.45689
....................UGAAGUGUUUGGGGGAACUA......................................................................................................................................    173.561085
....................UGAAGUGUUUGGGGGAACUU......................................................................................................................................    494.126627
....................UGAAGUGUUUGGGGGCACUC......................................................................................................................................    3.41142
....................UGAAGUGUUUGGGGGAACGC......................................................................................................................................    7.60419
....................UGAAGUGUUUGGGGGGACUC......................................................................................................................................    1.74419
....................UGAAGUGUUUGGGGGAACUUUU....................................................................................................................................    5.28629
....................UGAAGUGUUUGGGGGAACCC......................................................................................................................................    31.73744
....................UGAAGUGUUUGGGGGAACUCC.....................................................................................................................................    17.66074
.....................GAAGUGUUUGGGGGAACU.......................................................................................................................................    43.736
.....................GAAGUGUUUGGGGGAACUC......................................................................................................................................    5.1317
......................AAGUGUUUGGGGGAACUC......................................................................................................................................    4.22703
......................AAGUGUUUGGGGGAACUCU.....................................................................................................................................    3.15555
.....................................................................................................................................AGUUCCCUUCAAGCACUUCACA...................    24.4443
......................................................................................................................................GUUCCCUUCAAGCACUUCA.....................    4.21907
......................................................................................................................................GUUCCCUUCAAGCACUUCACAU..................    1.45641
......................................................................................................................................GUUCCCUUCAAGCACUUC......................    1.45641
......................................................................................................................................GUUCCCUUCAAGCACUUCAC....................    57.451406
......................................................................................................................................GUUCUCUUCAAGCACUUCACA...................    2.73952
......................................................................................................................................GUUCCCUACAAGCACUUCACA...................    202.562794
```

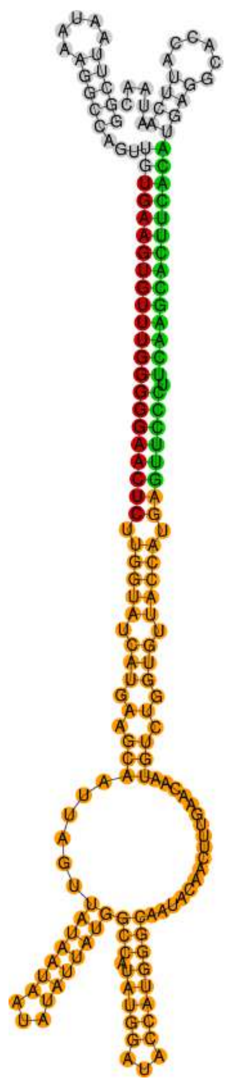

zma-MIR395i

```
Chromosome:    chr2
Start:         6286455
End:           6286628
Strand:        -
Strand bias:   1.0
Abundance bias:1.0
MFE:           -63.30
```

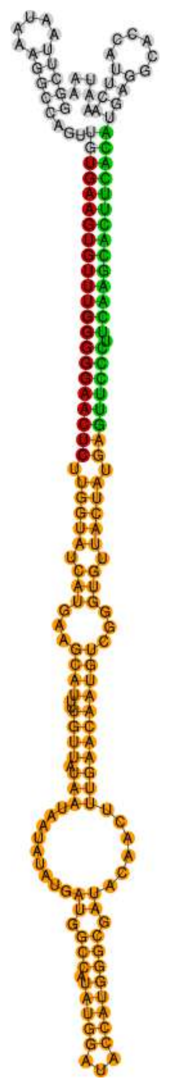

```
                    Mature                                                                                                                     Star
GGCUUAAUAAAGGCCAGUUGUGAAGUGUUUGGGGAACUCUUGGUAUCAUGAAGCAUUUUGUUAUAAUAAUAUAUGAUGGCCAUAUGGAUACCAUGGGCGAUACAACUUUGAACAAUGUCGGGUGUUACUAUGAGUUCCCUUCAAGCACUUCACAUGAGGCACCAUUCAAAUAA    7355.6
(((((.....)))))...(((((((((((((((((((((.(((((.(((...((((..((((.(((.........((.(((.(((((...)))))))).))......)))))))))))...))).))))).)))))))).))))))))))))))((((......)))).....
....................UGAAGUGUUUGGGGAACUC......................................................................................................................................    4586.749169
.....................................................................................................................................GUUCCCUUCAAGCACUUCACA...................    367.525387
...................GUGAAGUGUUUGGGGAAC........................................................................................................................................    1.94188
...................GUGAAGUGUUUGGGGAACUC......................................................................................................................................    8.66896
...................GUGAAGUGUUUGGGGAACU.......................................................................................................................................    14.84634
....................UGAAGUGUUUGGGGAAC........................................................................................................................................    117.318839
....................UGAAGUGUUUGGGGAACUCU.....................................................................................................................................    66.540551
....................UGAAGUGUUUGGGGAACU.......................................................................................................................................    897.367501
....................UGAAGUGUUUGGGGAAU........................................................................................................................................    13.78406
....................UGGAGUGUUUGGGGAACUC......................................................................................................................................    3.28276
....................UGAAGUGUUUGGGGAACG.......................................................................................................................................    9.43161
....................UGAAGUGUUUGGGGAACA.......................................................................................................................................    48.00465
....................UGAAGUGUUUGGGGAACC.......................................................................................................................................    111.29543
....................CGAAGUGUUUGGGGAACUC......................................................................................................................................    1.74419
....................UGAAGUGUUUGGGGAACUCA.....................................................................................................................................    21.45689
....................UGAAGUGUUUGGGGAACUA......................................................................................................................................    173.561085
....................UGAAGUGUUUGGGGAACUU......................................................................................................................................    494.126627
....................UGAAGUGUUUGGGGCACUC......................................................................................................................................    3.41142
....................UGAAGUGUUUGGGGAACGC......................................................................................................................................    7.60419
....................UGAAGUGUUUGGGGGACUC......................................................................................................................................    1.74419
....................UGAAGUGUUUGGGGAACUUUU....................................................................................................................................    5.28629
....................UGAAGUGUUUGGGGAACCC......................................................................................................................................    31.73744
....................UGAAGUGUUUGGGGAACUCC.....................................................................................................................................    17.66074
.....................GAAGUGUUUGGGGAACU.......................................................................................................................................    43.736
.....................GAAGUGUUUGGGGAACUC......................................................................................................................................    5.1317
......................AAGUGUUUGGGGAACUC......................................................................................................................................    4.22703
......................AAGUGUUUGGGGAACUCU.....................................................................................................................................    3.15555
....................................................................................................................................AGUUCCCUUCAAGCACUUCACA...................    24.4443
.....................................................................................................................................GUUCCCUUCAAGCACUUCA.....................    4.21907
.....................................................................................................................................GUUCCCUUCAAGCACUUCACAU..................    1.45641
.....................................................................................................................................GUUCCCUUCAAGCACUUC......................    1.45641
.....................................................................................................................................GUUCCCUUCAAGCACUUCAC....................    57.451406
.....................................................................................................................................GUUCUCUUCAAGCACUUCACA...................    2.73952
.....................................................................................................................................GUUCCCUACAAGCACUUCACA...................    202.562794
```

zma-MIR396a

Chromosome: chr4
Start: 176326884
End: 176327022
Strand: +
Strand bias: 1.0
Abundance bias: 1.0
MFE: -68.70

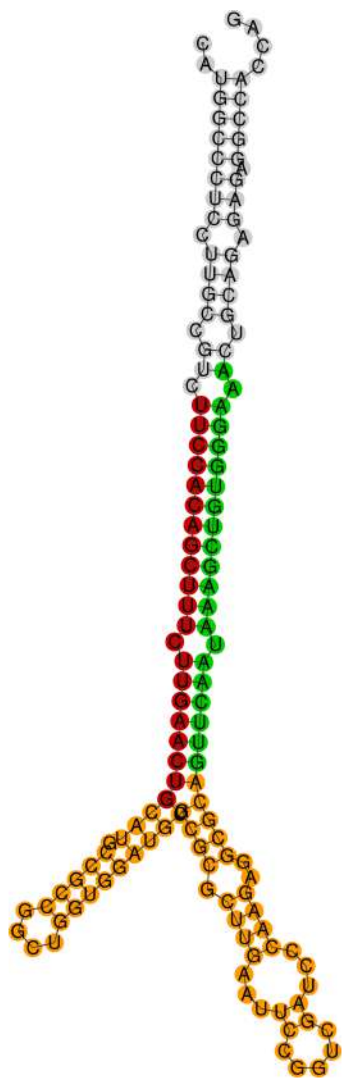

```
                   Mature                                                     Star
CAUGGCCCUCCUUGCCGUCUUCCACAGCUUUCUUGAACUGCAUGCCGCCGGCUGGUGGAUGCUGCGCGCUUGAAUUCCGGUCGAUCCCAAGAGGCGCAGUUCAAUAAAGCUGUGGGAAACUGCAGAGAGAGGCCACCAG    112071.3
..((((((((.((((.((.((((((((((((.(((((((((((.(((((....))))))))).((((.((((...((.....))...))))..))))))))))).)))))))))))).)).)))).))).))))).... 
...................UUCCACAGCUUUCUUGAACUG...................................................................................................    74743.45919
..................................................................................................GUUCAAUAAAGCUGUGGGAAA....................    12512.225567
..................CUUCCACAGCUUUCUUGAACU....................................................................................................    296.078421
..................CUUCCACAGCUUUCUUGAACUG...................................................................................................    111.726646
..................CUUCCACAGCUUUCUUGAACC....................................................................................................    6.55772
..................CUUCCACAGCUUUCUUGAACUU...................................................................................................    33.295479
...................UUCCACAGCUUUCUUGAACUGCA.................................................................................................    12.01656
...................UUCCACAGCUUUCUUGAACU....................................................................................................    7484.1465
...................UUCCACAGCUUUCUUGAA......................................................................................................    1536.222334
...................UUCCACAGCUUUCUUGAACUGC..................................................................................................    1290.529518
...................UUCCACAGCUUUCUUGAAC.....................................................................................................    1845.36578
...................UUCCACAGCUUUCUUGAACGG...................................................................................................    45.14813
...................UUCCACAGCUCUCUUGAACUG...................................................................................................    71.267815
...................UUCCACUGCUUUCUUGAACUG...................................................................................................    22.721367
...................CUCCACAGCUUUCUUGAACUG...................................................................................................    116.84043
...................UUCCACAGCUUUCUUGAAAUG...................................................................................................    13.11437
...................UCCCACAGCUUUCUUGAACUG...................................................................................................    98.071923
...................UUCGACAGCUUUCUUGAACUG...................................................................................................    9.948958
...................UUCCACAGCUUUUUUGAACUG...................................................................................................    21.684294
...................UUCCCCAGCUUUCUUGAACUG...................................................................................................    31.16441
...................UUCCACAGCUUUCUUGAAUUG...................................................................................................    34.256721
...................UUCCACAGCUUUCUUGAACUC...................................................................................................    726.341129
...................UUCCACAGAUUUCUUGAACUG...................................................................................................    24.131914
...................UUCCACAGCUUUCUUGAACG....................................................................................................    64.33308
...................UUCUACAGCUUUCUUGAACUG...................................................................................................    136.51992
...................UUCCACAGUUUUCUUGAACUG...................................................................................................    290.57116
...................UUCCACAGCUUUCUUGACCUG...................................................................................................    32.384536
...................UUCCACAGCUUUCUUGUACUG...................................................................................................    16.335462
...................UUCCACAUCUUUCUUGAACUG...................................................................................................    188.36403
...................UUCCACAGCUUUCUUGAACCG...................................................................................................    133.936643
...................UUACACAGCUUUCUUGAACUG...................................................................................................    39.711813
...................UUCAACAGCUUUCUUGAACUG...................................................................................................    33.993125
...................UUCCUCAGCUUUCUUGAACUG...................................................................................................    15.889733
...................UUCCACAGCUUUCUUAAACUG...................................................................................................    23.551716
...................UGCCACAGCUUUCUUGAACUG...................................................................................................    22.746791
...................UUCCACAACUUUCUUGAACUG...................................................................................................    14.286128
...................UUCCACAGCUUUCUUGCACU....................................................................................................    8.52046
...................UUCCACAGCUUUCUUGAACC....................................................................................................    428.469029
...................UUCCACAGCUUUCUUGAACA....................................................................................................    175.022076
...................UUCCACAGCUUUCUCGAACUG...................................................................................................    95.45931
...................UUCCAAAGCUUUCUUGAACUG...................................................................................................    11.05051
...................GUCCACAGCUUUCUUGAACUG...................................................................................................    24.889016
...................UUCCACAGCUUUCUUGAGCU....................................................................................................    4.179448
...................UUCCACAGCUUUCUUUAACUG...................................................................................................    18.636552
...................UUCCAUAGCUUUCUUGAACUG...................................................................................................    193.553628
...................UUCCACAGCUUUCUUGAACUGA..................................................................................................    264.82662
...................UUCCACAGCUUUCUUGAACUGU..................................................................................................    70.763185
...................UUCCGCAGCUUUCUUGAACUG...................................................................................................    99.429891
...................UUCCACAGCUUUCCUGAACUG...................................................................................................    79.417
...................UUGCACAGCUUUCUUGAACUG...................................................................................................    12.839496
...................UUUCACAGCUUUCUUGAACUG...................................................................................................    271.336737
...................UUCCACAGCUUCCUUGAACUG...................................................................................................    82.865643
...................UUCCACAGCUUUCUUGGACUG...................................................................................................    120.059769
...................UUCCACAGCUUUCUUGAAU.....................................................................................................    142.705877
...................UUCCACAGCUUUCUUGAACUA...................................................................................................    1175.545569
...................UUCCACAGCUUUCUUGCACUG...................................................................................................    103.983
...................UUCCACACCUUUCUUGAACUG...................................................................................................    42.828565
...................UUCCACAGCUUUCUUGAAUU....................................................................................................    6.823218
...................UACCACAGCUUUCUUGAACUG...................................................................................................    19.270959
...................UUCCACAGCUUUCUUGAACAG...................................................................................................    22.641026
...................UUCCACCGCUUUCUUGAACUG...................................................................................................    101.133833
...................UUCCACAGCUUUCUUGAGCUG...................................................................................................    112.066966
...................UUCCACAGCCUUCUUGAACUG...................................................................................................    144.815832
...................AUCCACAGCUUUCUUGAACUG...................................................................................................    40.543942
...................UUCCACGGCUUUCUUGAACUG...................................................................................................    158.493348
....................UCCACAGCUUUCUUGAACUG...................................................................................................    618.422082
....................UCCACAGCUUUCUUGAACU....................................................................................................    24.903312
....................UCCACAGGUUUCUUGAACUG...................................................................................................    41.529324
.....................CCACAGCUUUCUUGAACUG...................................................................................................    191.457259
......................CACAGCUUUCUUGAACUG...................................................................................................    245.59471
.................................................................................................AGUUCAAUAAAGCUGUGGGAAA....................    96.363444
..................................................................................................GUUCAAUAAAGCUGUGGGA......................    1036.443251
..................................................................................................GUUCAAUAAAGCUGUGGGAA.....................    875.087814
..................................................................................................GUUCAAUAAAGCUGUGGG.......................    262.34772
..................................................................................................GUUCAAUAAUGCUGUGGGAAA....................    4.85089
..................................................................................................GUUCAAUAAAGCUGUGGGGAA....................    8.067039
..................................................................................................GUUCAAUAAAGCUGUGGGAG.....................    7.25601
..................................................................................................GUUUAAUAAAGCUGUGGGAAA....................    5.92887
..................................................................................................GUUCAAUAGAGCUGUGGGAAA....................    3.99758
..................................................................................................GUUCAAUAAAGCUGUGGA.......................    12.77581
..................................................................................................GUUCGAUAAAGCUGUGGGAAA....................    8.696344
..................................................................................................GUCCAAUAAAGCUGUGGGAAA....................    6.168374
..................................................................................................GUUCAAUAAAGCUGUGGGAGA....................    12.991687
..................................................................................................GUUCAAUAAAGCUGUGGGAAG....................    85.457169
..................................................................................................GUUCAAUAAAGCUGUGGGAAC....................    11.093914
..................................................................................................GUUCAAUAAAGCUGUGGGAAU....................    46.179648
..................................................................................................GUUCAGUAAAGCUGUGGGAAA....................    7.524051
..................................................................................................GUUCAAUAACGCUGUGGGAAA....................    5.38988
..................................................................................................GUUCAAUAAGGCUGUGGGAAA....................    10.112766
..................................................................................................GUUCAAUAAAGUUGUGGGAAA....................    3.874983
..................................................................................................GCUCAAUAAAGCUGUGGGAAA....................    7.691979
..................................................................................................UUUCAAUAAAGCUGUGGGAAA....................    20.334054
...................................................................................................UUCAAUAAAGCUGUGGGAAAC...................    988.961245
...................................................................................................UUCAAUAAAGCUGUGGGA......................    41.799376
...................................................................................................UUCAAUAAAGCUGUGGGAAA....................    1085.687658
...................................................................................................UUCAAUAAAGCUGUGGGAA.....................    25.814825
....................................................................................................UCAAUAAAGCUGUGGGAAAC...................    5.460647
....................................................................................................UCAAUAAAGCUGUGGGAAA....................    23.555856
```

zma-MIR396b

```
Chromosome:     chr9
Start:          15122740
End:            15122873
Strand:         -
Strand bias:    1.0
Abundance bias: 1.0
MFE:            -67.90
```

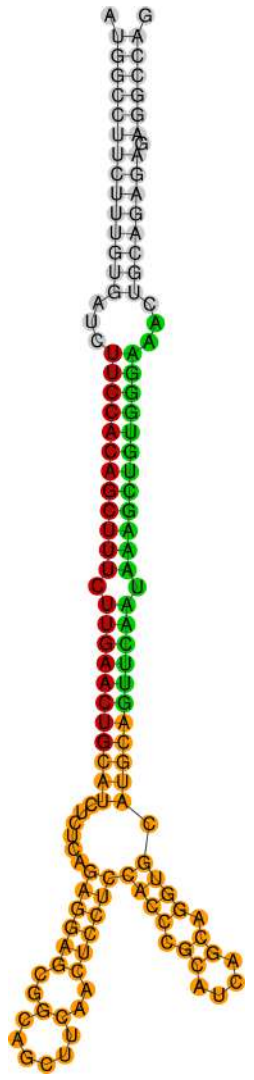

```
                  Mature                                                                    Star
AUGGCCUUCUUUGUGAUCUUCCACAGCUUUCUUGAACUGCAUCUCUCAGAGGAGCGGCAGCUUCAACUCCUCCACCCGCAUCAGCAGGUGCAUGCAGUUCAAUAAAGCUGUGGGAAACUGCAGAGAGAGGCCAG   112092.1
.((((((((((((((...((((((((((((.(((((((((((......((((((..(......)..))))))((((.((....)).)))).))))))))))).))))))))))))...)))))))).)))))).
..................UUCCACAGCUUUCUUGAACUG...............................................................................................   74743.45919
................................................................................................GUUCAAUAAAGCUGUGGGAAA.................   12512.225567
...............AUCUUCCACAGCUUUCUUGAACUG...............................................................................................   33.79804
.................CUUCCACAGCUUUCUUGAACU................................................................................................   296.078421
.................CUUCCACAGCUUUCUUGAACUG...............................................................................................   111.726646
.................CUUCCACAGCUUUCUUGAACC................................................................................................   6.55772
.................CUUCCACAGCUUUCUUGAACUU...............................................................................................   33.295479
..................UUCCACAGCUUUCUUGAACUGCA.............................................................................................   12.01656
..................UUCCACAGCUUUCUUGAACU................................................................................................   7484.1465
..................UUCCACAGCUUUCUUGAA..................................................................................................   1536.222334
..................UUCCACAGCUUUCUUGAACUGC..............................................................................................   1290.529518
..................UUCCACAGCUUUCUUGAAC.................................................................................................   1845.36578
..................UUCCACAGCUUUCUUGAACGG...............................................................................................   45.14813
..................UUCCACAGCUCUCUUGAACUG...............................................................................................   71.267815
..................UUCCACUGCUUUCUUGAACUG...............................................................................................   22.721367
..................CUCCACAGCUUUCUUGAACUG...............................................................................................   116.84043
..................UUCCACAGCUUUCUUGAAAUG...............................................................................................   13.11437
..................UCCCACAGCUUUCUUGAACUG...............................................................................................   98.071923
..................UUCGACAGCUUUCUUGAACUG...............................................................................................   9.948958
..................UUCCACAGCUUUUUUGAACUG...............................................................................................   21.684294
..................UUCCCCAGCUUUCUUGAACUG...............................................................................................   31.16441
..................UUCCACAGCUUUCUUGAAUUG...............................................................................................   34.256721
..................UUCCACAGCUUUCUUGAACUC...............................................................................................   726.341129
..................UUCCACAGAUUUCUUGAACUG...............................................................................................   24.131914
..................UUCCACAGCUUUCUUGAACG................................................................................................   64.33308
..................UUCUACAGCUUUCUUGAACUG...............................................................................................   136.51992
..................UUCCACAGUUUUCUUGAACUG...............................................................................................   290.57116
..................UUCCACAGCUUUCUUGACCUG...............................................................................................   32.384536
..................UUCCACAGCUUUCUUGUACUG...............................................................................................   16.335462
..................UUCCACAUCUUUCUUGAACUG...............................................................................................   188.36403
..................UUCCACAGCUUUCUUGAACCG...............................................................................................   133.936643
..................UUACACAGCUUUCUUGAACUG...............................................................................................   39.711813
..................UUCAACAGCUUUCUUGAACUG...............................................................................................   33.993125
..................UUCCUCAGCUUUCUUGAACUG...............................................................................................   15.889733
..................UUCCACAGCUUUCUUAAACUG...............................................................................................   23.551716
..................UGCCACAGCUUUCUUGAACUG...............................................................................................   22.746791
..................UUCCACAACUUUCUUGAACUG...............................................................................................   14.286128
..................UUCCACAGCUUUCUUGCACU................................................................................................   8.52046
..................UUCCACAGCUUUCUUGAACC................................................................................................   428.469029
..................UUCCACAGCUUUCUUGAACA................................................................................................   175.022076
..................UUCCACAGCUUUCUCGAACUG...............................................................................................   95.45931
..................UUCCAAAGCUUUCUUGAACUG...............................................................................................   11.05051
..................GUCCACAGCUUUCUUGAACUG...............................................................................................   24.889016
..................UUCCACAGCUUUCUUGAGCU................................................................................................   4.179448
..................UUCCACAGCUUUCUUUAACUG...............................................................................................   18.636552
..................UUCCAUAGCUUUCUUGAACUG...............................................................................................   193.553628
..................UUCCACAGCUUUCUUGAACUGA..............................................................................................   264.82662
..................UUCCACAGCUUUCUUGAACUGU..............................................................................................   70.763185
..................UUCCGCAGCUUUCUUGAACUG...............................................................................................   99.429891
..................UUCCACAGCUUUCCUGAACUG...............................................................................................   79.417
..................UUGCACAGCUUUCUUGAACUG...............................................................................................   12.839496
..................UUUCACAGCUUUCUUGAACUG...............................................................................................   271.336737
..................UUCCACAGCUUCCUUGAACUG...............................................................................................   82.865643
..................UUCCACAGCUUUCUUGGACUG...............................................................................................   120.059769
..................UUCCACAGCUUUCUUGAAU.................................................................................................   142.705877
..................UUCCACAGCUUUCUUGAACUA...............................................................................................   1175.545569
..................UUCCACAGCUUUCUUGCACUG...............................................................................................   103.983
..................UUCCACACCUUUCUUGAACUG...............................................................................................   42.828565
..................UUCCACAGCUUUCUUGAAUU................................................................................................   6.823218
..................UACCACAGCUUUCUUGAACUG...............................................................................................   19.270959
..................UUCCACAGCUUUCUUGAACAG...............................................................................................   22.641026
..................UUCCACCGCUUUCUUGAACUG...............................................................................................   101.133833
..................UUCCACAGCUUUCUUGAGCUG...............................................................................................   112.066966
..................UUCCACAGCCUUCUUGAACUG...............................................................................................   144.815832
..................AUCCACAGCUUUCUUGAACUG...............................................................................................   40.543942
..................UUCCACGGCUUUCUUGAACUG...............................................................................................   158.493348
...................UCCACAGCUUUCUUGAACUG...............................................................................................   618.422082
...................UCCACAGCUUUCUUGAACU................................................................................................   24.903312
...................UCCACAGGUUUCUUGAACUG...............................................................................................   41.529324
....................CCACAGCUUUCUUGAACUG...............................................................................................   191.457259
.....................CACAGCUUUCUUGAACUG...............................................................................................   245.59471
...............................................................................................AGUUCAAUAAAGCUGUGGGAAA.................   96.363444
................................................................................................GUUCAAUAAAGCUGUGGGA...................   1036.443251
................................................................................................GUUCAAUAAAGCUGUGGGAA..................   875.087814
................................................................................................GUUCAAUAAAGCUGUGGG....................   262.34772
................................................................................................GUUCAAUAAUGCUGUGGGAAA.................   4.85089
................................................................................................GUUCAAUAAAGCUGUGGGGAA.................   8.067039
................................................................................................GUUCAAUAAAGCUGUGGGAG..................   7.25601
................................................................................................GUUUAAUAAAGCUGUGGGAAA.................   5.92887
................................................................................................GUUCAAUAGAGCUGUGGGAAA.................   3.99758
................................................................................................GUUCAAUAAAGCUGUGGA....................   12.77581
................................................................................................GUUCGAUAAAGCUGUGGGAAA.................   8.696344
................................................................................................GUCCAAUAAAGCUGUGGGAAA.................   6.168374
................................................................................................GUUCAAUAAAGCUGUGGGAGA.................   12.991687
................................................................................................GUUCAAUAAAGCUGUGGGAAG.................   85.457169
................................................................................................GUUCAAUAAAGCUGUGGGAAC.................   11.093914
................................................................................................GUUCAAUAAAGCUGUGGGAAU.................   46.179648
................................................................................................GUUCAGUAAAGCUGUGGGAAA.................   7.524051
................................................................................................GUUCAAUAACGCUGUGGGAAA.................   5.38988
................................................................................................GUUCAAUAAGGCUGUGGGAAA.................   10.112766
................................................................................................GCUCAAUAAAGCUGUGGGAAA.................   7.691979
................................................................................................UUUCAAUAAAGCUGUGGGAAA.................   20.334054
.................................................................................................UUCAAUAAAGCUGUGGGAAAC................   988.961245
.................................................................................................UUCAAUAAAGCUGUGGGA...................   41.799376
.................................................................................................UUCAAUAAAGCUGUGGGAAA.................   1085.687658
.................................................................................................UUCAAUAAAGCUGUGGGAA..................   25.814825
..................................................................................................UCAAUAAAGCUGUGGGAAAC................   5.460647
..................................................................................................UCAAUAAAGCUGUGGGAAA.................   23.555856
```

zma-MIR396c

Chromosome: chr2
Start: 1951562
End: 1951804
Strand: -
Strand bias: 1.0
Abundance bias:0.996
MFE: -113.90

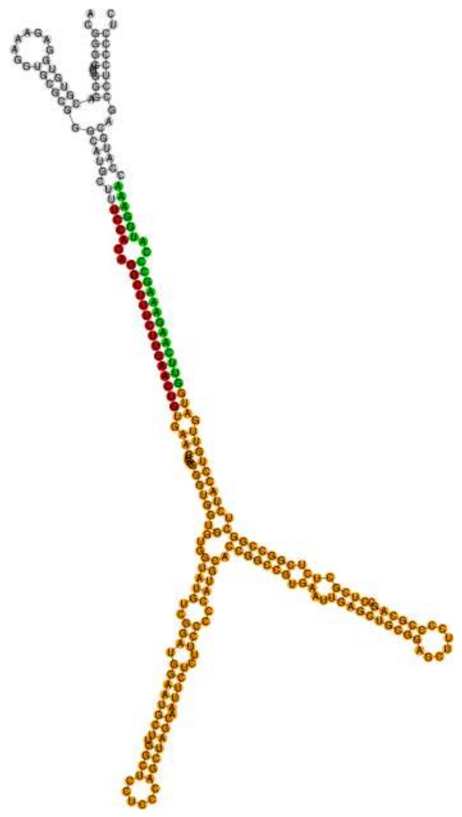

Mature Star

```
ACGGGGUGUGGGACGUGUGGAGAAAGGUGCGCGGGCAUGCUUUCCACAGGCUUUCUUGAACUGUGAACUCGUGGGUGGUGGUGGUAUGUCGGAUGGAAUGCUUGGCUCCUCCCAGCUAGCAAUUCUCUUCCCCCAUGCACCGGCCGUGAAUUGAGCUGCGGAGCUUCCCCGCAGCCUCGCUCUCGGCCGGCUCUACCUGUUGAUGGUUCAAGAAAGCCCAUGGAAACCAUGCAGCCUCCCCUC    840053.8
..((((...(((.((((((........)))))).(((((.((((((..((((((((((((((((.(((....((((((.(...(((((..(((.((((((((.((((......))))))).)))))..)))..))))).(((((((.((..((((((((((.......)))))).)))).)).)))))))).))))))))).))))))))))))))))..)))))).)))))..))))))).. 
..........................................UCCACAGGCUUUCUUGAACUG....................................................................................................................................................................................    718887.31059
.............................................................................................................................................................................................................GUUCAAGAAAGCCCAUGGAAA.................    2355.112633
.......................................CUUUCCACAGGCUUUCUUGAA.......................................................................................................................................................................................    2896.113552
..........................................UCCACAGGCUUUCUUGAACUGU...................................................................................................................................................................................    1156.715765
..........................................UCCACAGGCUUUCUUGAACU.....................................................................................................................................................................................    36521.804453
..........................................UCCACAGACUUUCUUGAACUG....................................................................................................................................................................................    307.704861
..........................................UCCACAGGCUUUCUUGAACCG....................................................................................................................................................................................    1438.083916
..........................................UCCACAGGAUUUCUUGAACUG....................................................................................................................................................................................    428.305037
..........................................UCCACAGGCUUUUUUGAACUG....................................................................................................................................................................................    468.328238
..........................................UUCACAGGCUUUCUUGAACUG....................................................................................................................................................................................    1589.05865
..........................................UCCACAGGCUUUCUCGAACUG....................................................................................................................................................................................    1026.442829
..........................................UCCACAGGUUUUCUUGAACUG....................................................................................................................................................................................    716.741899
..........................................UCCUCAGGCUUUCUUGAACUG....................................................................................................................................................................................    264.290133
..........................................UCCACUGGCUUUCUUGAACUG....................................................................................................................................................................................    251.431646
..........................................ACCACAGGCUUUCUUGAACUG....................................................................................................................................................................................    404.531885
..........................................CCCACAGGCUUUCUUGAACUG....................................................................................................................................................................................    1120.396047
..........................................UCCACAGGCUUUCUUGAACUGC...................................................................................................................................................................................    1146.263692
..........................................UCCACAGGCUUUCUUGAAUU.....................................................................................................................................................................................    371.824141
..........................................UCCACAGGCUUUCUUUAACUG....................................................................................................................................................................................    1463.348522
..........................................UCCACAGGCUUUCCUGAACUG....................................................................................................................................................................................    940.64492
..........................................UCUACAGGCUUUCUUGAACUG....................................................................................................................................................................................    1041.179848
..........................................UCCACAGUCUUUCUUGAACUG....................................................................................................................................................................................    481.7404
..........................................UCCACAGGCUUUCUUGGACUG....................................................................................................................................................................................    1241.286017
..........................................UCCGCAGGCUUUCUUGAACUG....................................................................................................................................................................................    953.077192
..........................................UCCACCGGCUUUCUUGAACUG....................................................................................................................................................................................    697.096726
..........................................UCCAUAGGCUUUCUUGAACUG....................................................................................................................................................................................    737.943239
..........................................UCCACAGGCUUCCUUGAACUG....................................................................................................................................................................................    946.35867
..........................................UCCACAGGCUUUCUUGCACUG....................................................................................................................................................................................    3130.768522
..........................................UCCACAAGCUUUCUUGAACUG....................................................................................................................................................................................    432.07935
..........................................UCCACAGGCUUUCUUGAACUGA...................................................................................................................................................................................    3192.296666
..........................................UCCACAGGCUUUCUUGAGCUG....................................................................................................................................................................................    1129.855393
..........................................UCCACAGGCUUUCUUGAAU......................................................................................................................................................................................    629.521759
..........................................UCCCCAGGCUUUCUUGAACUG....................................................................................................................................................................................    361.032161
..........................................GCCACAGGCUUUCUUGAACUG....................................................................................................................................................................................    261.388925
..........................................UCCACAGGCUUUCUUGAAAUG....................................................................................................................................................................................    1819.600816
..........................................UCCACAGGCUUUCUUGAACUA....................................................................................................................................................................................    11091.575494
..........................................UCCACAGGCUUUCUUGAACUU....................................................................................................................................................................................    5136.836528
..........................................UCCACAGGCUUUCUUGACCUG....................................................................................................................................................................................    784.78331
..........................................UCCACGGGCUUUCUUGAACUG....................................................................................................................................................................................    1158.515602
..........................................UCCACAGGCUUUCUUGAACA.....................................................................................................................................................................................    1550.501843
..........................................UCCACAGGCUUUCUUGAACC.....................................................................................................................................................................................    3220.153343
..........................................UCCACAGGCUUUCUUGAACGG....................................................................................................................................................................................    658.928353
..........................................UCCACACGCUUUCUUGAACUG....................................................................................................................................................................................    375.435355
..........................................UCCACAUGCUUUCUUGAACUG....................................................................................................................................................................................    541.885965
..........................................UCCACAGGCUUUCUUGAAUUG....................................................................................................................................................................................    540.214783
..........................................UCCACAGGCUUUCUUGAACUC....................................................................................................................................................................................    1212.463033
..........................................UCCACAGGCUUUCUUGAACUUU...................................................................................................................................................................................    3122.989501
..........................................UCCACAGGCUUUCUUGUACUG....................................................................................................................................................................................    270.455451
..........................................UCCACAGGCUUUCUUAAACUG....................................................................................................................................................................................    446.260474
..........................................UCCACAGGCUCUCUUGAACUG....................................................................................................................................................................................    735.836224
..........................................UCAACAGGCUUUCUUGAACUG....................................................................................................................................................................................    449.031367
..........................................UCCACAGGCCUUCUUGAACUG....................................................................................................................................................................................    1188.761652
...........................................CCACAGGCUUUCUUGAACUG....................................................................................................................................................................................    1080.000516
............................................CACAGGCUUUCUUGAACUG....................................................................................................................................................................................    1644.059362
.............................................ACAGGCUUUCUUGAACUGUGA.................................................................................................................................................................................    442.155791
.............................................ACAGGCUUUCUUGAACUG....................................................................................................................................................................................    4101.17094
..........................................................................................................................................................................................................AUGGUUCAAGAAAGCCCAUGGAAA.................    1607.57863
..............................................................................................................................................................................................................UUCAAGAAAGCCCAUGGAAA.................    294.581777
```

zma-MIR396e

Chromosome: chr4
Start: 176331822
End: 176332046
Strand: -
Strand bias: 1.0
Abundance bias: 0.997
MFE: -65.40

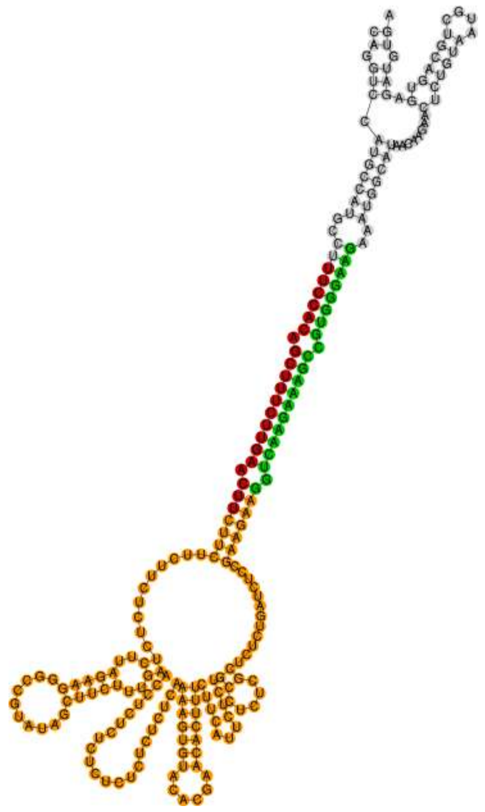

```
                  Mature                                                                                                                                                       Star
CAGGUCCAUGCCAUGCCUUUCCACAGCUUUCUUGAACUUCUUCUUCUUCUCUCUCUUAGAAGGGCCGUAUAGCUUCUUUGAACCUCUCUCUCUCUCUCUCUCAAAAGUGUACACGAACACUUUCUUCAUUCUCUGCCUCUCGCUCUCUGAUCUCCGAAGAAGGUCAAGAAAGCCGUGGGAAGAAAUGGCAUAACAAGAACUCUGUAAUGCUGCAGUGAGAUGUGA    78975.0
((.(((.(((((((..((((((((.((((((((((.(((((((..........((..(((((..........)))))..))......................(((((((......)))))))...........((.....))...........))))))).)))))))))).))))))))..))))))).......(.(((((....))))).).))).)).
..................UUCCACAGCUUUCUUGAACUU..........................................................................................................................................................................................    61048.799606
.................................................................................................................................................................GGUCAAGAAAGCCGUGGGAAG...........................................    16.793603
.................UUUCCACAGCUUUCUUGAACUU..........................................................................................................................................................................................    110.679514
.................UUUCCACAGCUUUCUUGAACU...........................................................................................................................................................................................    172.154969
..................UUCCACAGCUUUCUUGAACUUCUU.......................................................................................................................................................................................    44.104037
..................UUCCACAGCUUUCUUGAACUUC.........................................................................................................................................................................................    77.273151
..................UUCCACAGCUUUCUUGAACU...........................................................................................................................................................................................    7484.1465
..................UUCCACAGCUUUCUUGAA.............................................................................................................................................................................................    1536.222334
..................UUCCACAGCUUUCUUGAAC............................................................................................................................................................................................    1845.36578
..................UUUCACAGCUUUCUUGAACUU..........................................................................................................................................................................................    76.084759
..................UUCCACAGCUCUCUUGAACUU..........................................................................................................................................................................................    61.326549
..................UUCCACUGCUUUCUUGAACUU..........................................................................................................................................................................................    22.369356
..................CUCCACAGCUUUCUUGAACUU..........................................................................................................................................................................................    122.932443
..................UUACACAGCUUUCUUGAACUU..........................................................................................................................................................................................    44.107294
..................UCCCACAGCUUUCUUGAACUU..........................................................................................................................................................................................    101.871484
..................UUCCACAGCUUUAUUGAACUU..........................................................................................................................................................................................    13.378211
..................UUCCACAGCUUUUUUGAACUU..........................................................................................................................................................................................    31.574656
..................UUCCCCAGCUUUCUUGAACUU..........................................................................................................................................................................................    15.6393
..................UUCCACAGCUUUCUUGAACUC..........................................................................................................................................................................................    726.341129
..................UUCCACAGAUUUCUUGAACUU..........................................................................................................................................................................................    21.933326
..................UUCCACAGCUUUCUUGAACG...........................................................................................................................................................................................    64.33308
..................UUCUACAGCUUUCUUGAACUU..........................................................................................................................................................................................    80.760736
..................UUCCACAGUUUUCUUGAACUU..........................................................................................................................................................................................    63.057274
..................UUCCACAGCUUUCUUGACCUU..........................................................................................................................................................................................    18.891378
..................UUCCACAUCUUUCUUGAACUU..........................................................................................................................................................................................    59.120892
..................UUCCACAGCUUUCUUGAACCU..........................................................................................................................................................................................    127.624423
..................UUCAACAGCUUUCUUGAACUU..........................................................................................................................................................................................    26.779914
..................UUCCACAGCUUUCUUAAACUU..........................................................................................................................................................................................    26.872129
..................UUCCACAGCUUUCUUGAACUUU.........................................................................................................................................................................................    1037.436115
..................UUCCACAGCUUUCUUGAACUUA.........................................................................................................................................................................................    62.899522
..................UGCCACAGCUUUCUUGAACUU..........................................................................................................................................................................................    12.880743
..................UUCCACAACUUUCUUGAACUU..........................................................................................................................................................................................    19.971546
..................UACCACAGCUUUCUUGAACUU..........................................................................................................................................................................................    19.827435
..................UUCCACAGCUUUCUUGAACC...........................................................................................................................................................................................    428.469029
..................UUCCACAGCUUUCUUGAACA...........................................................................................................................................................................................    175.022076
..................UUCCACAGCUUUCUCGAACUU..........................................................................................................................................................................................    100.939939
..................GUCCACAGCUUUCUUGAACUU..........................................................................................................................................................................................    29.354608
..................UUCCACAGCUUUCUUUAACUU..........................................................................................................................................................................................    23.922236
..................UUCCAUAGCUUUCUUGAACUU..........................................................................................................................................................................................    66.917989
..................UUCCGCAGCUUUCUUGAACUU..........................................................................................................................................................................................    93.516844
..................UUCCACAGCUUUCCUGAACUU..........................................................................................................................................................................................    81.00885
..................UUGCACAGCUUUCUUGAACUU..........................................................................................................................................................................................    14.685554
..................AUCCACAGCUUUCUUGAACUU..........................................................................................................................................................................................    43.026632
..................UUCCUCAGCUUUCUUGAACUU..........................................................................................................................................................................................    13.786286
..................UUCCACAGCUUCCUUGAACUU..........................................................................................................................................................................................    88.76967
..................UUCCACAGCUUUCUUGGACUU..........................................................................................................................................................................................    121.345236
..................UUCCACAGCCUUCUUGAACUU..........................................................................................................................................................................................    129.064155
..................UUCCACAGCUUUCUUGAAU............................................................................................................................................................................................    142.705877
..................UUCCACAGCUUUCUUGAAUUU..........................................................................................................................................................................................    38.315873
..................UUCCACAGCUUUCUUGAACUA..........................................................................................................................................................................................    1175.545569
..................UUCCACAGCUUUCUUGCACUU..........................................................................................................................................................................................    73.7117
..................UUCCACAGCUUUCUUGAACAU..........................................................................................................................................................................................    34.645359
..................UUCCACCGCUUUCUUGAACUU..........................................................................................................................................................................................    119.02558
..................UUCCACAGCUUUCUUGAGCUU..........................................................................................................................................................................................    122.85581
..................UUCCACGGCUUUCUUGAACUU..........................................................................................................................................................................................    136.213677
...................UCCACAGCUUUCUUGAACUU..........................................................................................................................................................................................    86.73899
...................UCCACAGCUUUCUUGAACU...........................................................................................................................................................................................    24.903312
.....................CACAGCUUUCUUGAACUU..........................................................................................................................................................................................    33.348719
```

zma-MIR396f

Chromosome: chr5
Start: 220211637
End: 220211788
Strand: +
Strand bias: 1.0
Abundance bias: 0.977
MFE: -58.10

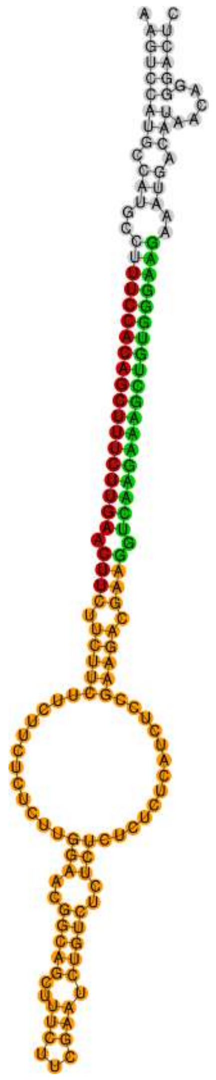

**Mature:** UUCCACAGCUUUCUUGAACUU; **Star:** GGUCAAGAAAGCUGUGGGAAG	
AAGUCCAUGCCAUGCCUUUCCACAGCUUUCUUGAACUUCUUCUUCUUCUUCUCUCUUGGAACGGCAGCUUUCUUCGAAUCUGUCUCUCUCUCUCUCAUCUCCGAAGACGAAGGUCAAGAAAGCUGUGGGAAGAAAUGACAUAACAGGGACUC	91217.4
.((((((((.(((..((((((((((((((((((.((((.(((((...........(((..(((((..(((...))).)))))..)))............))))).)))).))))))))))))))))))..))).))).....))))).	
UUCCACAGCUUUCUUGAACUU	61048.799606
GGUCAAGAAAGCUGUGGGAAG	9010.653472
UUUCCACAGCUUUCUUGAACUU	110.679514
UUUCCACAGCUUUCUUGAACU	172.154969
UUCCACAGCUUUCUUGAACUUCUU	44.104037
UUCCACAGCUUUCUUGAACUUC	77.273151
UUCCACAGCUUUCUUGAACU	7484.1465
UUCCACAGCUUUCUUGAA	1536.222334
UUCCACAGCUUUCUUGAAC	1845.36578
UUUCACAGCUUUCUUGAACUU	76.084759
UUCCACAGCUCUCUUGAACUU	61.326549
UUCCACUGCUUUCUUGAACUU	22.369356
CUCCACAGCUUUCUUGAACUU	122.932443
UUACACAGCUUUCUUGAACUU	44.107294
UUCCACAGCUUUCUUGAAAUU	10.138191
UCCCACAGCUUUCUUGAACUU	101.871484
UUCCACAGCUUUAUUGAACUU	13.378211
UUCCACAGCUUUUUUGAACUU	31.574656
UUCCCCAGCUUUCUUGAACUU	15.6393
UUCCACAGCUUUCUUGAACUC	726.341129
UUCCACAGAUUUCUUGAACUU	21.933326
UUCCACAGCUUUCUUGAACG	64.33308
UUCUACAGCUUUCUUGAACUU	80.760736
UUCCACAGUUUUCUUGAACUU	63.057274
UUCCACAGCUUUCUUGGACU	3.715919
UUCCACAGCUUUCUUGACCUU	18.891378
UUCCACAGCUUUCUUGUACUU	7.744787
UUCCACAUCUUUCUUGAACUU	59.120892
UUCCACAGCUUUCUUGAACCU	127.624423
UUCAACAGCUUUCUUGAACUU	26.779914
UUCCACAGCCUUCUUGAACU	3.63555
UUCCACAGCUUUCUUAAACUU	26.872129
UUCCACAGCUUUCUUGAACUUU	1037.436115
UUCCACAGCUUUCUUGAACUUA	62.899522
UGCCACAGCUUUCUUGAACUU	12.880743
UUCCACAACUUUCUUGAACUU	19.971546
UUCCACAGCUUUCUUGCACU	8.52046
UACCACAGCUUUCUUGAACUU	19.827435
UUCCACAGCUUUCUUGAACC	428.469029
UUCCACAGCUUUCUUGAACA	175.022076
UUCCACAGCUUUCUCGAACUU	100.939939
UUCCAAAGCUUUCUUGAACUU	10.27561
GUCCACAGCUUUCUUGAACUU	29.354608
UUCCACAGCUUUCUUGAGCU	4.179448
UUCCACAGCUUUCUUUAACUU	23.922236
UUCCAUAGCUUUCUUGAACUU	66.917989
UUCCGCAGCUUUCUUGAACUU	93.516844
UUCCACAGCUUUCCUGAACUU	81.00885
UUGCACAGCUUUCUUGAACUU	14.685554
AUCCACAGCUUUCUUGAACUU	43.026632
UUCCUCAGCUUUCUUGAACUU	13.786286
UUCCACAGCUUCCUUGAACUU	88.76967
UUCCACAGCUUUCUUGGACUU	121.345236
UUCCACAGCCUUCUUGAACUU	129.064155
UUCCACAGCUUUCUUGAAU	142.705877
UUCCACAGCUUUCUUGAAUUU	38.315873
UUCCACAGCUUUCUUGAACUA	1175.545569
UUCCACAGCUUUCUUGCACUU	73.7117
UUCCACACCUUUCUUGAACUU	10.368434
UUCCACAGCAUUCUUGAACUU	3.759279
UUCCACAGCUUUCUUGAAUU	6.823218
UUCCACAGCUUUCUUGAACAU	34.645359
UUCCACCGCUUUCUUGAACUU	119.02558
UUCCACAGCUUUCUUGAGCUU	122.85581
UUCCACGGCUUUCUUGAACUU	136.213677
UCCACAGCUUUCUUGAACUU	86.73899
UCCACAGCUUUCUUGAACU	24.903312
CCACAGCUUUCUUGAACUU	7.287021
CACAGCUUUCUUGAACUU	33.348719
GAAGGUCAAGAAAGCUGUGGGAAG	262.367784
GAAGGUCAAGAAAGCUGUGGGAAA	9.533804
AAGGUCAAGAAAGCUGUGGGAAG	9.490044
AAGGUCAAGAAAGCUGUGGGAAGA	9.52154
GGUCAAGAAAGCUGUGGGA	109.684355
GGUCAAGAAAGCUGUGGGAA	122.3278
GGUCAAGAAAGCUGUGGGAAGA	85.950717
GGUCAAGAAGGCUGUGGGAAG	15.822129
GGUCAAGAAAGCUGUGGGAGG	9.342115
GAUCAAGAAAGCUGUGGGAAG	9.810675
GGUCAAGAAAGCUGUGGGGAG	17.477163
GGUCAGGAAAGCUGUGGGAAG	11.807723
GGCCAAGAAAGCUGUGGGAAG	10.829284
GGGCAAGAAAGCUGUGGGAAG	3.991506
GGUCAAGAAAGCUGCGGGAAG	4.363284
GGUCAAGAAAGCUGUGGGAAGC	10.061156
GGUCAAGAAAGCUGUGGGAAGU	37.327448
GGUCGAGAAAGCUGUGGGAAG	12.37438
UGUCAAGAAAGCUGUGGGAAG	7.16714
GGUCAAGAGAGCUGUGGGAAG	8.823979
GGUCAAGAAAGCUGUGGGAAU	5.980317
GGUCAAGAAAGCUGUGGGAAA	116.384421
AGUCAAGAAAGCUGUGGGAAG	6.677991
GUUCAAGAAAGCUGUGGGAAG	7.868507
GGUCAAGGAAGCUGUGGGAAG	11.974558
GUCAAGAAAGCUGUGGGAAGA	465.772424
GUCAAGAAAGCUGUGGGA	4.372449
GUCAAGAAAGCUGUGGGAAG	35.422528
UCAAGAAAGCUGUGGGAAG	5.004979

zma-MIR396d

```
Chromosome:    chr10
Start:         148727697
End:           148727964
Strand:        +
Strand bias:   1.0
Abundance bias:0.996
MFE:           -123.00
```

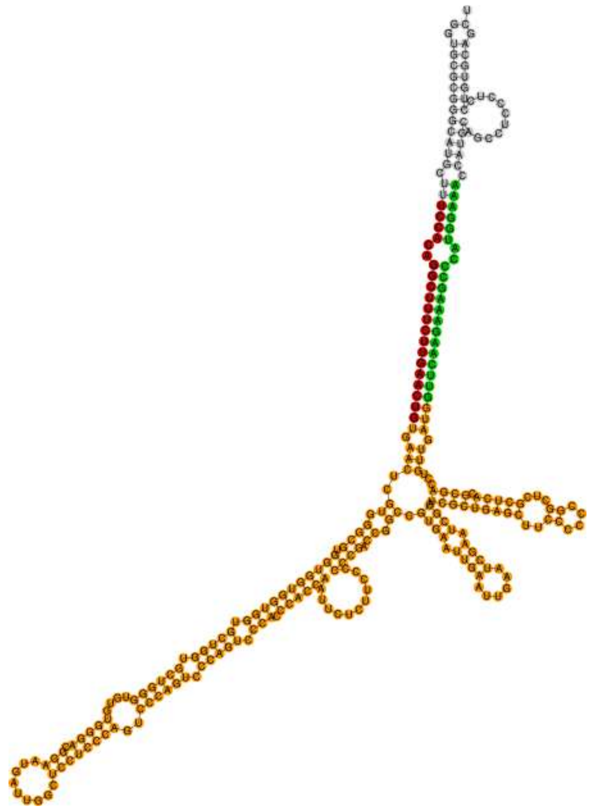

```
                Mature                                                                                                                                                                                                                   Star
GGUGCGCGGGCAUGCUUUCCACAGGCUUUCUUGAACUGUGAACUCGUGGGCGUGGUGGUGGUGGUGCUGGUGCUGGGUGUGUGGGACGGAAUGAUUGGCUCCUCCCAGUCCCAGUCCCAGUCCCACCCACCAAUUCUCUUCCCCCCGACCGGCCGUGAAUUGAAUUGAAUCGAAUCGCAUCGCUGAGCUUCCCCCCGGCUCGCUCACGCGACCUGUUGAUGGUUCAAGAAAGCCCAUGGAAACCAUGCAGCCUCCCUCCUGUGCAGCU    840053.0
(.((((((((((((.((((((..((((((((((((((((.(((..((.((((.((((((((((((.(((((.((((((....(((((.(((.........))))))))..)))))).))))).))).))))))..........)))).)).)).((((.((((......)))).)))).(((((((((..((....))...))))).))))...))).)))))))))))))))..)))))).))))).........))))))).).
.................UCCACAGGCUUUCUUGAACUG......................................................................................................................................................................................................................................    718887.31059
.............................................................................................................................................................................................................................GUUCAAGAAAGCCCAUGGAAA..........................    2355.112633
..............CUUUCCACAGGCUUUCUUGAA.........................................................................................................................................................................................................................................    2896.113552
.................UCCACAGGCUUUCUUGAACUGU.....................................................................................................................................................................................................................................    1156.715765
.................UCCACAGGCUUUCUUGAACU.......................................................................................................................................................................................................................................    36521.804453
.................UCCACAGACUUUCUUGAACUG......................................................................................................................................................................................................................................    307.704861
.................UCCACAGGCUUUCUUGAACCG......................................................................................................................................................................................................................................    1438.083916
.................UCCACAGGAUUUCUUGAACUG......................................................................................................................................................................................................................................    428.305037
.................UCCACAGGCUUUUUUGAACUG......................................................................................................................................................................................................................................    468.328238
.................UUCACAGGCUUUCUUGAACUG......................................................................................................................................................................................................................................    1589.05865
.................UCCACAGGCUUUCUCGAACUG......................................................................................................................................................................................................................................    1026.442829
.................UCCACAGGUUUUCUUGAACUG......................................................................................................................................................................................................................................    716.741899
.................UCCUCAGGCUUUCUUGAACUG......................................................................................................................................................................................................................................    264.290133
.................UCCACUGGCUUUCUUGAACUG......................................................................................................................................................................................................................................    251.431646
.................ACCACAGGCUUUCUUGAACUG......................................................................................................................................................................................................................................    404.531885
.................CCCACAGGCUUUCUUGAACUG......................................................................................................................................................................................................................................    1120.396047
.................UCCACAGGCUUUCUUGAACUGC.....................................................................................................................................................................................................................................    1146.263692
.................UCCACAGGCUUUCUUGAAUU.......................................................................................................................................................................................................................................    371.824141
.................UCCACAGGCUUUCUUUAACUG......................................................................................................................................................................................................................................    1463.348522
.................UCCACAGGCUUUCCUGAACUG......................................................................................................................................................................................................................................    940.64492
.................UCUACAGGCUUUCUUGAACUG......................................................................................................................................................................................................................................    1041.179848
.................UCCACAGUCUUUCUUGAACUG......................................................................................................................................................................................................................................    481.7404
.................UCCACAGGCUUUCUUGGACUG......................................................................................................................................................................................................................................    1241.286017
.................UCCGCAGGCUUUCUUGAACUG......................................................................................................................................................................................................................................    953.077192
.................UCCACCGGCUUUCUUGAACUG......................................................................................................................................................................................................................................    697.096726
.................UCCAUAGGCUUUCUUGAACUG......................................................................................................................................................................................................................................    737.943239
.................UCCACAGGCUUCCUUGAACUG......................................................................................................................................................................................................................................    946.35867
.................UCCACAGGCUUUCUUGCACUG......................................................................................................................................................................................................................................    3130.768522
.................UCCACAAGCUUUCUUGAACUG......................................................................................................................................................................................................................................    432.07935
.................UCCACAGGCUUUCUUGAACUGA.....................................................................................................................................................................................................................................    3192.296666
.................UCCACAGGCUUUCUUGAGCUG......................................................................................................................................................................................................................................    1129.855393
.................UCCACAGGCUUUCUUGAAU........................................................................................................................................................................................................................................    629.521759
.................UCCCCAGGCUUUCUUGAACUG......................................................................................................................................................................................................................................    361.032161
.................GCCACAGGCUUUCUUGAACUG......................................................................................................................................................................................................................................    261.388925
.................UCCACAGGCUUUCUUGAAAUG......................................................................................................................................................................................................................................    1819.600816
.................UCCACAGGCUUUCUUGAACUA......................................................................................................................................................................................................................................    11091.575494
.................UCCACAGGCUUUCUUGAACUU......................................................................................................................................................................................................................................    5136.836528
.................UCCACAGGCUUUCUUGACCUG......................................................................................................................................................................................................................................    784.78331
.................UCCACGGGCUUUCUUGAACUG......................................................................................................................................................................................................................................    1158.515602
.................UCCACAGGCUUUCUUGAACA.......................................................................................................................................................................................................................................    1550.501843
.................UCCACAGGCUUUCUUGAACC.......................................................................................................................................................................................................................................    3220.153343
.................UCCACAGGCUUUCUUGAACGG......................................................................................................................................................................................................................................    658.928353
.................UCCACACGCUUUCUUGAACUG......................................................................................................................................................................................................................................    375.435355
.................UCCACAUGCUUUCUUGAACUG......................................................................................................................................................................................................................................    541.885965
.................UCCACAGGCUUUCUUGAAUUG......................................................................................................................................................................................................................................    540.214783
.................UCCACAGGCUUUCUUGAACUC......................................................................................................................................................................................................................................    1212.463033
.................UCCACAGGCUUUCUUGAACUUU.....................................................................................................................................................................................................................................    3122.989501
.................UCCACAGGCUUUCUUGUACUG......................................................................................................................................................................................................................................    270.455451
.................UCCACAGGCUUUCUUAAACUG......................................................................................................................................................................................................................................    446.260474
.................UCCACAGGCUCUCUUGAACUG......................................................................................................................................................................................................................................    735.836224
.................UCAACAGGCUUUCUUGAACUG......................................................................................................................................................................................................................................    449.031367
.................UCCACAGGCCUUCUUGAACUG......................................................................................................................................................................................................................................    1188.761652
..................CCACAGGCUUUCUUGAACUG......................................................................................................................................................................................................................................    1080.000516
...................CACAGGCUUUCUUGAACUG......................................................................................................................................................................................................................................    1644.059362
....................ACAGGCUUUCUUGAACUGUGA...................................................................................................................................................................................................................................    442.155791
....................ACAGGCUUUCUUGAACUG......................................................................................................................................................................................................................................    4101.17094
..........................................................................................................................................................................................................................AUGGUUCAAGAAAGCCCAUGGAAA..........................    1607.57863
..............................................................................................................................................................................................................................UUCAAGAAAGCCCAUGGAAA..........................    294.581777
```

zma-MIR396_N1

Chromosome: chr5
Start: 220222130
End: 220222277
Strand: -
Strand bias: 1.0
Abundance bias:1.0
MFE: -59.60

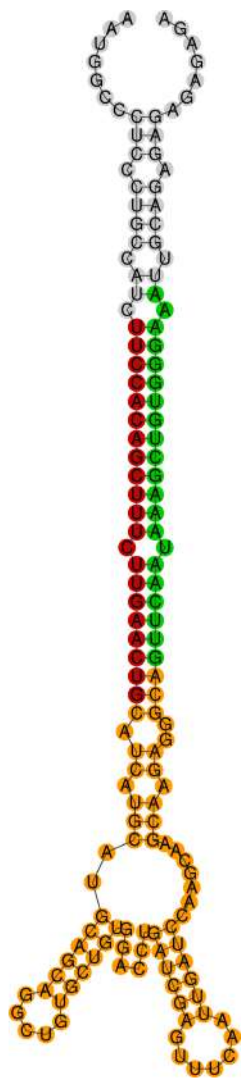

```
                   Mature                                                                                    Star
AAUGGCCCUCCCUGCCAUCUUCCACAGCUUUCUUGAACUGCAUCAUGCAUGCAGCAGGCUGUGCUGUGGACCUGAUCGAGUUUCAAUUGAUCCAAGCAAGCAAGAGGGCAGUUCAAUAAAGCUGUGGGAAAUUGCAGAGAGAGAGAGA   111136.2
.......(((.((((.((.((((((((((((.(((((((((.((.(((..((((((.....))))))......((((((.......)))))).......))).))..))))))))).)))))))))))).)).)))).))).......
...................UUCCACAGCUUUCUUGAACUG............................................................................................................   74743.45919
..............................................................................................................GUUCAAUAAAGCUGUGGGAAA.................   12512.225567
................AUCUUCCACAGCUUUCUUGAACUG............................................................................................................   33.79804
..................CUUCCACAGCUUUCUUGAACU.............................................................................................................   296.078421
..................CUUCCACAGCUUUCUUGAACUG............................................................................................................   111.726646
..................CUUCCACAGCUUUCUUGAACC.............................................................................................................   6.55772
..................CUUCCACAGCUUUCUUGAACUU............................................................................................................   33.295479
...................UUCCACAGCUUUCUUGAACUGCA..........................................................................................................   12.01656
...................UUCCACAGCUUUCUUGAACU.............................................................................................................   7484.1465
...................UUCCACAGCUUUCUUGAA...............................................................................................................   1536.222334
...................UUCCACAGCUUUCUUGAACUGC...........................................................................................................   1290.529518
...................UUCCACAGCUUUCUUGAAC..............................................................................................................   1845.36578
...................UUCCACAGCUUUCUUGAACGG............................................................................................................   45.14813
...................UUCCACAGCUCUCUUGAACUG............................................................................................................   71.267815
...................UUCCACUGCUUUCUUGAACUG............................................................................................................   22.721367
...................CUCCACAGCUUUCUUGAACUG............................................................................................................   116.84043
...................UUCCACAGCUUUCUUGAAAUG............................................................................................................   13.11437
...................UCCCACAGCUUUCUUGAACUG............................................................................................................   98.071923
...................UUCGACAGCUUUCUUGAACUG............................................................................................................   9.948958
...................UUCCACAGCUUUUUUGAACUG............................................................................................................   21.684294
...................UUCCCCAGCUUUCUUGAACUG............................................................................................................   31.16441
...................UUCCACAGCUUUCUUGAAUUG............................................................................................................   34.256721
...................UUCCACAGCUUUCUUGAACUC............................................................................................................   726.341129
...................UUCCACAGAUUUCUUGAACUG............................................................................................................   24.131914
...................UUCCACAGCUUUCUUGAACG.............................................................................................................   64.33308
...................UUCUACAGCUUUCUUGAACUG............................................................................................................   136.51992
...................UUCCACAGUUUUCUUGAACUG............................................................................................................   290.57116
...................UUCCACAGCUUUCUUGACCUG............................................................................................................   32.384536
...................UUCCACAGCUUUCUUGUACUG............................................................................................................   16.335462
...................UUCCACAUCUUUCUUGAACUG............................................................................................................   188.36403
...................UUCCACAGCUUUCUUGAACCG............................................................................................................   133.936643
...................UUACACAGCUUUCUUGAACUG............................................................................................................   39.711813
...................UUCAACAGCUUUCUUGAACUG............................................................................................................   33.993125
...................UUCCUCAGCUUUCUUGAACUG............................................................................................................   15.889733
...................UUCCACAGCUUUCUUAAACUG............................................................................................................   23.551716
...................UGCCACAGCUUUCUUGAACUG............................................................................................................   22.746791
...................UUCCACAACUUUCUUGAACUG............................................................................................................   14.286128
...................UUCCACAGCUUUCUUGCACU.............................................................................................................   8.52046
...................UUCCACAGCUUUCUUGAACC.............................................................................................................   428.469029
...................UUCCACAGCUUUCUUGAACA.............................................................................................................   175.022076
...................UUCCACAGCUUUCUCGAACUG............................................................................................................   95.45931
...................UUCCAAAGCUUUCUUGAACUG............................................................................................................   11.05051
...................GUCCACAGCUUUCUUGAACUG............................................................................................................   24.889016
...................UUCCACAGCUUUCUUGAGCU.............................................................................................................   4.179448
...................UUCCACAGCUUUCUUUAACUG............................................................................................................   18.636552
...................UUCCAUAGCUUUCUUGAACUG............................................................................................................   193.553628
...................UUCCACAGCUUUCUUGAACUGA...........................................................................................................   264.82662
...................UUCCACAGCUUUCUUGAACUGU...........................................................................................................   70.763185
...................UUCCGCAGCUUUCUUGAACUG............................................................................................................   99.429891
...................UUCCACAGCUUUCCUGAACUG............................................................................................................   79.417
...................UUGCACAGCUUUCUUGAACUG............................................................................................................   12.839496
...................UUUCACAGCUUUCUUGAACUG............................................................................................................   271.336737
...................UUCCACAGCUUCCUUGAACUG............................................................................................................   82.865643
...................UUCCACAGCUUUCUUGGACUG............................................................................................................   120.059769
...................UUCCACAGCUUUCUUGAAU..............................................................................................................   142.705877
...................UUCCACAGCUUUCUUGAACUA............................................................................................................   1175.545569
...................UUCCACAGCUUUCUUGCACUG............................................................................................................   103.983
...................UUCCACACCUUUCUUGAACUG............................................................................................................   42.828565
...................UUCCACAGCUUUCUUGAAUU.............................................................................................................   6.823218
...................UACCACAGCUUUCUUGAACUG............................................................................................................   19.270959
...................UUCCACAGCUUUCUUGAACAG............................................................................................................   22.641026
...................UUCCACCGCUUUCUUGAACUG............................................................................................................   101.133833
...................UUCCACAGCUUUCUUGAGCUG............................................................................................................   112.066966
...................UUCCACAGCCUUCUUGAACUG............................................................................................................   144.815832
...................AUCCACAGCUUUCUUGAACUG............................................................................................................   40.543942
...................UUCCACGGCUUUCUUGAACUG............................................................................................................   158.493348
....................UCCACAGCUUUCUUGAACUG............................................................................................................   618.422082
....................UCCACAGCUUUCUUGAACU.............................................................................................................   24.903312
....................UCCACAGGUUUCUUGAACUG............................................................................................................   41.529324
.....................CCACAGCUUUCUUGAACUG............................................................................................................   191.457259
......................CACAGCUUUCUUGAACUG............................................................................................................   245.59471
.............................................................................................................AGUUCAAUAAAGCUGUGGGAAA.................   96.363444
..............................................................................................................GUUCAAUAAAGCUGUGGGA...................   1036.443251
..............................................................................................................GUUCAAUAAAGCUGUGGGAA..................   875.087814
..............................................................................................................GUUCAAUAAAGCUGUGGG....................   262.34772
..............................................................................................................GUUCAAUAAUGCUGUGGGAAA.................   4.85089
..............................................................................................................GUUCAAUAAAGCUGUGGGGAA.................   8.067039
..............................................................................................................GUUCAAUAAAGCUGUGGGAG..................   7.25601
..............................................................................................................GUUUAAUAAAGCUGUGGGAAA.................   5.92887
..............................................................................................................GUUCAAUAGAGCUGUGGGAAA.................   3.99758
..............................................................................................................GUUCAAUAAAGCUGUGGA....................   12.77581
..............................................................................................................GUUCGAUAAAGCUGUGGGAAA.................   8.696344
..............................................................................................................GUCCAAUAAAGCUGUGGGAAA.................   6.168374
..............................................................................................................GUUCAAUAAAGCUGUGGGAGA.................   12.991687
..............................................................................................................GUUCAAUAAAGCUGUGGGAAG.................   85.457169
..............................................................................................................GUUCAAUAAAGCUGUGGGAAC.................   11.093914
..............................................................................................................GUUCAAUAAAGCUGUGGGAAU.................   46.179648
..............................................................................................................GUUCAGUAAAGCUGUGGGAAA.................   7.524051
..............................................................................................................GUUCAAUAACGCUGUGGGAAA.................   5.38988
..............................................................................................................GUUCAAUAAGGCUGUGGGAAA.................   10.112766
..............................................................................................................GUUCAAUAAAGUUGUGGGAAA.................   3.874983
..............................................................................................................GCUCAAUAAAGCUGUGGGAAA.................   7.691979
..............................................................................................................UUUCAAUAAAGCUGUGGGAAA.................   20.334054
...............................................................................................................UUCAAUAAAGCUGUGGGAAAU................   44.6883
...............................................................................................................UUCAAUAAAGCUGUGGGA...................   41.799376
...............................................................................................................UUCAAUAAAGCUGUGGGAAA.................   1085.687658
...............................................................................................................UUCAAUAAAGCUGUGGGAA..................   25.814825
................................................................................................................UCAAUAAAGCUGUGGGAAA.................   23.555856
```

zma-MIR397b

Chromosome: chr5
Start: 84610578
End: 84610688
Strand: -
Strand bias: 1.0
Abundance bias:0.996
MFE: -70.70

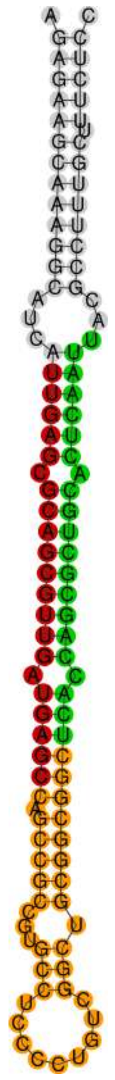

```
                  Mature                                                       Star
AGAGAAGCAAAGGCAUCAUUGAGCGCAGCGUUGAUGAGCCAGCCGCCGUGCCUCCCCUGUCGGCUGCGGCGGCUCACCAGCGCUGCACUCAAUUACGCCUUUGCUUUCUCC    177209.6
.(((((((((((((...((((((.(((((((((.((((((.(((((...(((.........))).))))))))))).))))))))).))))))...)))))))).))))).
..................UUGAGCGCAGCGUUGAUGAGC........................................................................    147781.505867
.........................................................................UCACCAGCGCUGCACUCAAUU.................    11563.724456
...............UCAUUGAGCGCAGCGUUGAUG...........................................................................    317.383196
...............UCAUUGAGCGCAGCGUUGAUGU..........................................................................    12.994256
................CAUUGAGCGCAGCGUUGAUGAGC........................................................................    8.13224
.................AUUGAGCGCAGCGUUGAUGAGC........................................................................    65.30739
.................AUUGAGCGCAGCGUUGAUGAG.........................................................................    83.266761
..................UUGAGCGCAGCGUUGAUGA..........................................................................    26.083858
..................UUGAGCGCAGCGUUGAUG...........................................................................    2649.234932
..................UUGAGCGCAGCGUUGAUGAGCC.......................................................................    9.75869
..................UUGAGCGCAGCGUUGAUGAGCCAG.....................................................................    9.266208
..................UUGAGCGCAGCGUUGAUGAG.........................................................................    208.95331
..................UUGAGCGCAGCGUUGAUGAGCCA......................................................................    58.450677
..................UUGAGAGCAGCGUUGAUGAGC........................................................................    13.287025
..................UUGAGCGCAGCGUUGAUGU..........................................................................    4.004592
..................UUGAGCGCAGCGUUGAUA...........................................................................    236.14096
..................UUGAGCGCAGCGUUGAUU...........................................................................    9.234064
..................AUGAGCGCAGCGUUGAUGAGC........................................................................    24.992187
..................UCGAGCGCAGCGUUGAUGAGC........................................................................    791.453159
..................UUGAACGCAGCGUUGAUGAGC........................................................................    28.867366
..................UUGAGUGCAGCGUUGAUGAGC........................................................................    74.451942
..................UUGAGCGCAGCGUUGAUGAGCA.......................................................................    191.082363
..................UUGAGCGCAGCGUUGAUGAGCU.......................................................................    53.155292
..................UUGAGCGCAGCGUUGUUGAGC........................................................................    22.470139
..................UUGCGCGCAGCGUUGAUGAGC........................................................................    403.72184
..................UUGAGCGCAGCAUUGAUGAGC........................................................................    41.99411
..................UUUAGCGCAGCGUUGAUGAGC........................................................................    224.314461
..................CUGAGCGCAGCGUUGAUGAGC........................................................................    121.551372
..................UUGAUCGCAGCGUUGAUGAGC........................................................................    41.049176
..................UUGAGCGCAGCGUGGAUGAGC........................................................................    9.30654
..................UUGAGCGCAGCGUUGAUGAC.........................................................................    3.632209
..................UUGAGCGCAGCGUUGAUGGGC........................................................................    157.15564
..................UUGAGCGCAGAGUUGAUGAGC........................................................................    13.673143
..................UUGAGCGCAGCGCUGAUGAGC........................................................................    63.942802
..................UUGAGCGCAGCGUUAAUGAGC........................................................................    16.264771
..................UUGAGCGCAGGGUUGAUGAGC........................................................................    10.466251
..................UUCAGCGCAGCGUUGAUGAGC........................................................................    50.426625
..................UUGACCGCAGCGUUGAUGAGC........................................................................    3.48139
..................UUGAGCGCAGCGUUGAUGAAC........................................................................    74.691267
..................UUGUGCGCAGCGUUGAUGAGC........................................................................    19.745611
..................UUGAGCGCUGCGUUGAUGAGC........................................................................    5.909427
..................UGGAGCGCAGCGUUGAUGAGC........................................................................    1306.535843
..................UUGAGCGCAGCGUUGAUGAUC........................................................................    58.969338
..................UUGAGCGCAGCGGUGAUGAGC........................................................................    9.06887
..................UUGAGCGCAGCGUUGAUGAGG........................................................................    70.829449
..................UUGAGCGCAGCGUUGAUUAGC........................................................................    55.318467
..................UUGAGCGCAGCGUUGACGAGC........................................................................    47.49515
..................UUAAGCGCAGCGUUGAUGAGC........................................................................    21.005673
..................UUGAGCGCAGCGUUGAUGUGC........................................................................    16.102219
..................UUGAGCGCAGCGUUUAUGAGC........................................................................    41.97903
..................UUGAGCGCAGCGUUGAUAAGC........................................................................    45.599089
..................UUGAGCGAAGCGUUGAUGAGC........................................................................    8.523193
..................UUGAGCGCAGCGUUGAUGAGA........................................................................    1249.219203
..................UUGAGCGCAGCGUUGAUGAGU........................................................................    5302.727898
..................UUGAGCCCAGCGUUGAUGAGC........................................................................    10.289389
..................UUGAGCUCAGCGUUGAUGAGC........................................................................    27.488072
..................UUGAGCGCAGUGUUGAUGAGC........................................................................    64.822326
..................UAGAGCGCAGCGUUGAUGAGC........................................................................    517.054383
..................UUGAGCGCAGCGUUGAGGAGC........................................................................    7.093272
..................UUGAGCGUAGCGUUGAUGAGC........................................................................    68.127689
..................UUGAGCGCAGCUUUGAUGAGC........................................................................    53.861784
..................UUGAGCGCAUCGUUGAUGAGC........................................................................    19.987424
..................UUGAGCACAGCGUUGAUGAGC........................................................................    42.054626
..................GUGAGCGCAGCGUUGAUGAGC........................................................................    41.168635
..................UUGAGCGCAGCGUCGAUGAGC........................................................................    54.247581
..................UUGAGCGCAGCGUUGGUGAGC........................................................................    92.854656
..................UUGAGCGCAGCGUUGAUGACC........................................................................    32.923563
..................UUGAGCGCAGCGUAGAUGAGC........................................................................    4.412935
..................UUGAGCGCGGCGUUGAUGAGC........................................................................    68.41716
..................UUGGGCGCAGCGUUGAUGAGC........................................................................    128.051103
...................UGAGCGCAGCGUUGAUGAGC........................................................................    165.71172
....................GAGCGCAGCGUUGAUGAGC........................................................................    58.383567
.....................AGCGCAGCGUUGAUGAGC........................................................................    12.751519
.........................................................................UCACCAGCGCUGCACUCAAU..................    132.898215
.........................................................................UCACCAGCGCUGCACUCAA...................    6.16913
.........................................................................UCACCAGCGCUGCACUCA....................    118.845727
.........................................................................UCACCAGCGCUGCACUCAAUUA................    4.55406
.........................................................................UCACCAGCGCCGCACUCAAUU.................    3.92953
.........................................................................UCACCAGCGCUGCAUUCAAUU.................    5.662893
.........................................................................UCACCAGCGCUGCACUCAAUA.................    33.48441
.........................................................................UCACCAGCGCUGCACUCAAUG.................    17.694159
.........................................................................CCACCAGCGCUGCACUCAAUU.................    6.893113
.........................................................................UUACCAGCGCUGCACUCAAUU.................    104.277275
.........................................................................UCACCAGCGCUGCACUCAAUUU................    11.03323
.........................................................................UCACCAGCGCUGCACUCAAUUC................    7.352113
.........................................................................UCACCAGCGCUGCCCUCAAUU.................    3.16219
.........................................................................UCAACAGCGCUGCACUCAAUU.................    3.04621
.........................................................................UCACCAGCGCUGCACUCAAUC.................    410.514609
.........................................................................UCACCAGCGCUGCGCUCAAUU.................    3.904845
.........................................................................UCGCCAGCGCUGCACUCAAUU.................    5.573094
.........................................................................UCACCAGCGCUGCACUCGAUU.................    4.108782
..........................................................................CACCAGCGCUGCACUCAAUU.................    9.80901
...........................................................................ACCAGCGCUGCACUCAAUU.................    8.7913
............................................................................CCAGCGCUGCACUCAAUU.................    179.440426
............................................................................CCAGCGCUGCACUCAAUUAC...............    6.7919
............................................................................CCAGCGCUGCACUCAAUUACG..............    225.949544
............................................................................CCAGCGCUGCACUCAAUC.................    10.70296
............................................................................CCAGCGCUGCACUCAAUUU................    8.129893
```

zma-MIR398a

Chromosome: chr2
Start: 174319882
End: 174320052
Strand: +
Strand bias: 1.0
Abundance bias: 0.991
MFE: -87.60

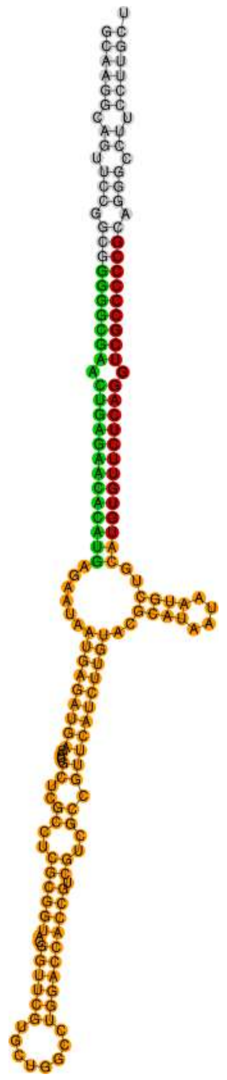

```
                 Star                                                                                                                    Mature
GCAAGGCAGUUCCGGCGGGGGCGAACUGAGAACACAUGAGAAUAAUGAGAUGAGAUUGCUCGCCUCGCGGUACGGUUCGUGCUGGCCUGGACCACCGUCGUCGCCGUUCAUCUUGUACGCAUAAUAAUGCUGCAUGUGUUCUCAGGUCGCCCCCGCAGGGCCUUCCUUGCU    93591.7
((((((.((.(((.((((((((((.(((((((((((((......(((((((((....((..((..((((((..((((((........)))))))))).))..)).)))))))))))..((((....))))..))))))))))))).)))))))))).))).)).)))))).
......................................................................................................................................UGUGUUCUCAGGUCGCCCCCG................    64848.416265
.................GGGGCGAACUGAGAACACAUG.....................................................................................................................................    5631.21748
........................................................................................................................................UGUUCUCAGGUCGCCCCCG................    178.585101
.......................................................................................................................................GUGUUCUCAGGUCGCCCCCG................    77.473169
.......................................................................................................................................GUGUUCUCAGGUCGCCCCCGC...............    28.420179
......................................................................................................................................UGUGUUCUCAGGUCGCCCC..................    1082.644756
......................................................................................................................................UGUGUUCUCAGGUCGCCC...................    1382.817255
......................................................................................................................................UGUGUUCUCAGGUCGCCCCCGCA..............    10.30473
......................................................................................................................................UGUGUUCUCAGGUCGCCCCC.................    812.765397
......................................................................................................................................UGUGUUCUCAGGUCGCCCU..................    150.916632
......................................................................................................................................UGUGUUCUCAGGUCGCUCCCG................    96.120976
......................................................................................................................................UGUGUUCUCAGGUCGCCCA..................    15.227486
......................................................................................................................................UGUGUUCUCACGUCGCCCCCG................    5.81126
......................................................................................................................................UGUGUUCUCAGGCCGCCCCCG................    99.188521
......................................................................................................................................UGUGUUCUUAGGUCGCCCCCG................    33.525559
......................................................................................................................................UGUUUUCUCAGGUCGCCCCCG................    95.874553
......................................................................................................................................UCUGUUCUCAGGUCGCCCCCG................    33.420215
......................................................................................................................................UGUGUUCUCAGGUAGCCCCCG................    13.463201
......................................................................................................................................GGUGUUCUCAGGUCGCCCCCG................    25.635286
......................................................................................................................................UGUGUUCUCAGGUCGCCCCCC................    185.87269
......................................................................................................................................UGUGUUCUCAGGUCGCCCCCA................    1695.747318
......................................................................................................................................UGUGUUCUCAGGUCGCCCCCU................    375.952567
......................................................................................................................................UGUGUUCUCAGUUCGCCCCCG................    26.115092
......................................................................................................................................UGUGUUCUCAGGUCGCCCCCGA...............    108.531259
......................................................................................................................................UGUAUUCUCAGGUCGCCCCCG................    22.182733
......................................................................................................................................UGUGUUCUCAGGUUGCCCCCG................    24.561499
......................................................................................................................................UGUGUUCUCAGGUCGCCU...................    102.402775
......................................................................................................................................UGUGUUGUCAGGUCGCCCCCG................    19.807949
......................................................................................................................................UGUGUUCUCUGGUCGCCCCCG................    7.158499
......................................................................................................................................UGUGUUUUCAGGUCGCCCCCG................    154.86953
......................................................................................................................................UGUGUUAUCAGGUCGCCCCCG................    33.53148
......................................................................................................................................UGUGUUCACAGGUCGCCCCCG................    9.729536
......................................................................................................................................UGUGUUCUCAGGGCGCCCCCG................    14.384443
......................................................................................................................................UGUGCUCUCAGGUCGCCCCCG................    70.496887
......................................................................................................................................UGUGUUCUCAGGUCGCCUCCG................    51.589414
......................................................................................................................................UGUGUUCUCAGGUCGCCA...................    14.419622
......................................................................................................................................UGUGUUCUCAGGUCGCCG...................    11.288294
......................................................................................................................................UGUGUUCUCAGGUCGUCCCCG................    306.587243
......................................................................................................................................UGUGUUCUCAGGUCGCCCCCGU...............    137.794322
......................................................................................................................................UGUGUUCUCAGGUCCCCCCCG................    44.736593
......................................................................................................................................CGUGUUCUCAGGUCGCCCCCG................    81.334755
......................................................................................................................................UGGGUUCUCAGGUCGCCCCCG................    51.192874
......................................................................................................................................UGUGUUCUCAGGUCGCCCCAG................    14.442211
......................................................................................................................................UUUGUUCUCAGGUCGCCCCCG................    65.589914
......................................................................................................................................UAUGUUCUCAGGUCGCCCCCG................    22.662611
......................................................................................................................................UGUGUUCUCAUGUCGCCCCCG................    67.664144
......................................................................................................................................UGUGUUCUCAGGUCGCCCCUG................    102.817921
......................................................................................................................................UGUGUCCUCAGGUCGCCCCCG................    77.971113
......................................................................................................................................UGUGUUCUCAGGUCGCCCUCG................    36.735595
......................................................................................................................................UGUGUUCUCAGGUCGCCGCCG................    8.257687
......................................................................................................................................UGUGUUCUCAGGACGCCCCCG................    19.312322
......................................................................................................................................UGUGUUCUCAGGUCACCCCCG................    43.327672
......................................................................................................................................UGUGUUCUCAGGUCGCCCCA.................    54.689125
......................................................................................................................................UGCGUUCUCAGGUCGCCCCCG................    112.163695
......................................................................................................................................UGUGUUCUAAGGUCGCCCCCG................    5.257365
......................................................................................................................................AGUGUUCUCAGGUCGCCCCCG................    31.034047
......................................................................................................................................UGAGUUCUCAGGUCGCCCCCG................    29.327977
......................................................................................................................................UGUGUUCCCAGGUCGCCCCCG................    87.151201
......................................................................................................................................UGUGUUCUCGGGUCGCCCCCG................    85.580781
......................................................................................................................................UGUGUUCUCAAGUCGCCCCCG................    16.984015
......................................................................................................................................UGUGUUCUCAGAUCGCCCCCG................    8.422973
......................................................................................................................................UGUGUUCUCAGGUCGCCCCU.................    137.89214
......................................................................................................................................UGUGUUCUCAGGUCGCCCCG.................    82.191632
.....................................................................................................................................AUGUGUUCUCAGGUCGCCCCC.................    6678.775948
.....................................................................................................................................AUGUGUUCUCAGGUCGCCC...................    42.563396
.....................................................................................................................................AUGUGUUCUCAGGUCGCCCCCG................    399.263314
.....................................................................................................................................AUGUGUUCUCAGGUCGCCCC..................    75.551733
.....................................................................................................................................AUGUGUUCUCAGGUCGCCCCA.................    181.09146
.....................................................................................................................................AUGUGUUCUCAGGUCGCCCCU.................    488.295385
.....................................................................................................................................AUGUGUUCUCAGGUCUCCCCC.................    7.430253
.....................................................................................................................................AUGUGUUCUCAGGUCGCCCCCA................    39.336594
.....................................................................................................................................AUGUGUUCUCAGGUCGCCCCCU................    82.64385
....................................................................................................................................CAUGUGUUCUCAGGUCGCCCC..................    2093.704775
....................................................................................................................................CAUGUGUUCUCAGGUCGCCCA..................    7.60258
....................................................................................................................................CAUGUGUUCUCAGGUCGCCCU..................    73.531251
....................................................................................................................................CAUGUGUUCUCAGGUCGCCCCA.................    6.09649
....................................................................................................................................CAUGUGUUCUCAGGUCGCCCCU.................    24.47679
.................GGGGCGAACUGAGAACACAUGAG...................................................................................................................................    6.220818
.................GGGGCGAACUGAGAACACAU......................................................................................................................................    523.550415
.................GGGGCGAACUGAGAACACA.......................................................................................................................................    411.589501
.................GGGGCGAACUGAGAACAC........................................................................................................................................    68.059453
.................GGGGCGAACUGAGAACACAUGA....................................................................................................................................    338.626343
.................GGGGCGAACUGAGAACACAUGAGA..................................................................................................................................    23.372125
.................GGGGCGAACUGAGAACACAUU.....................................................................................................................................    7.03432
.................GGGGCGAACUGAGAACACAUA.....................................................................................................................................    853.35155
.................GGGGCGAACUGAGAACGCAUG.....................................................................................................................................    8.35267
.................GGGGCGAACUGAGAACACAC......................................................................................................................................    10.36546
.................GGGGCGGACUGAGAACACAUG.....................................................................................................................................    9.568386
.................GGGGCGAACUGAGAACACAUAAG...................................................................................................................................    7.55817
.................GGGGCGAACUGAGAACACAUGU....................................................................................................................................    39.793525
.................GGGGCGAACUGAGAACAU........................................................................................................................................    6.78429
.................GGGGCGAACUGAGAACACGUG.....................................................................................................................................    5.78866
.................GUGGCGAACUGAGAACACAUG.....................................................................................................................................    6.5276
................GGGGGCGAACUGAGAACACA.......................................................................................................................................    30.39143
................GGGGGCGAACUGAGAACAC........................................................................................................................................    16.18712
................GGGGGCGAACUGAGAACACAU......................................................................................................................................    779.078025
................GGGGGCGAACUGAGAACACAC......................................................................................................................................    11.80851
................GGGGGCGAACUGAGAACACAUU.....................................................................................................................................    5.464751
```

zma-MIR398b

```
Chromosome:    chr7
Start:         39887922
End:           39888067
Strand:        +
Strand bias:   1.0
Abundance bias:0.982
MFE:           -72.10
```

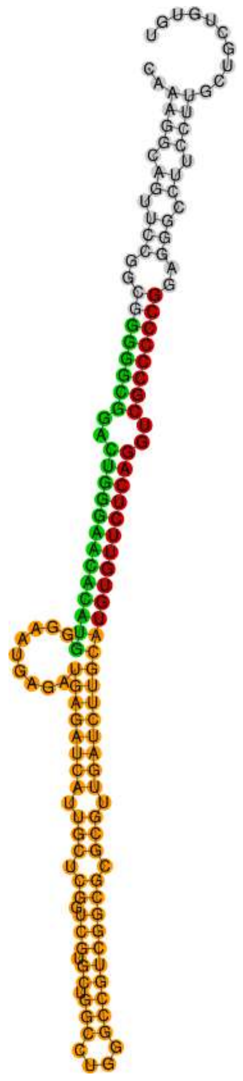

```
                 Star                                                                  Mature
CAAAGGCAGUUCCGGCGGGGGCGGACUGGGAACACAUGGGAAUGAGAUGAGAUCAUUGCUCGGUCGUGCUGGCCUGGGCCGUCGGCGCGCGUUGAUCUUGCAUGUGUUCUCAGGUCGCCCCCGGAGGGCCUUCCUUGCUGCUGUGU    121709.3
..((((.((.(((..((((((((..(((((((((((((.........((((((((.(((.((.(((.((.(((....)))))))))).))).)))))))))))))))))))))..))))))))..))).)).))))..........
......................................................................................................UGUGUUCUCAGGUCGCCCCCG.......................    64848.416265
.................GGGGCGGACUGGGAACACAUG............................................................................................................    18772.740569
........................................................................................................UGUUCUCAGGUCGCCCCCG.......................    178.585101
.......................................................................................................GUGUUCUCAGGUCGCCCCCG.......................    77.473169
......................................................................................................UGUGUUCUCAGGUCGCCCC.........................    1082.644756
......................................................................................................UGUGUUCUCAGGUCGCCC..........................    1382.817255
......................................................................................................UGUGUUCUCAGGUCGCCCCC........................    812.765397
......................................................................................................UGUGUUCUCAGGUCGCCCU.........................    150.916632
......................................................................................................UGUGUUCUCAGGUCGCUCCCG.......................    96.120976
......................................................................................................UGUGUUCUCAGGUCGCCCA.........................    15.227486
......................................................................................................UGUGUUCUCAGGCCGCCCCCG.......................    99.188521
......................................................................................................UGUGUUCUUAGGUCGCCCCCG.......................    33.525559
......................................................................................................UGUUUUCUCAGGUCGCCCCCG.......................    95.874553
......................................................................................................UCUGUUCUCAGGUCGCCCCCG.......................    33.420215
......................................................................................................UGUGUUCUCAGGUAGCCCCCG.......................    13.463201
......................................................................................................GGUGUUCUCAGGUCGCCCCCG.......................    25.635286
......................................................................................................UGUGUUCUCAGGUCGCCCCCC.......................    185.87269
......................................................................................................UGUGUUCUCAGGUCGCCCCCA.......................    1695.747318
......................................................................................................UGUGUUCUCAGGUCGCCCCCU.......................    375.952567
......................................................................................................UGUGUUCUCAGUUCGCCCCCG.......................    26.115092
......................................................................................................UGUGUUCUCAGGUCGCCCCCGA......................    108.531259
......................................................................................................UGUAUUCUCAGGUCGCCCCCG.......................    22.182733
......................................................................................................UGUGUUCUCAGGUUGCCCCCG.......................    24.561499
......................................................................................................UGUGUUCUCAGGUCGCCU..........................    102.402775
......................................................................................................UGUGUUGUCAGGUCGCCCCCG.......................    19.807949
......................................................................................................UGUGUUUUCAGGUCGCCCCCG.......................    154.86953
......................................................................................................UGUGUUAUCAGGUCGCCCCCG.......................    33.53148
......................................................................................................UGUGUUCUCAGGGCGCCCCCG.......................    14.384443
......................................................................................................UGUGCUCUCAGGUCGCCCCCG.......................    70.496887
......................................................................................................UGUGUUCUCAGGUCGCCUCCG.......................    51.589414
......................................................................................................UGUGUUCUCAGGUCGCCA..........................    14.419622
......................................................................................................UGUGUUCUCAGGUCGCCG..........................    11.288294
......................................................................................................UGUGUUCUCAGGUCGUCCCCG.......................    306.587243
......................................................................................................UGUGUUCUCAGGUCGCCCCCGU......................    137.794322
......................................................................................................UGUGUUCUCAGGUCGCCCCCCG......................    63.058175
......................................................................................................UGUGUUCUCAGGUCCCCCCCG.......................    44.736593
......................................................................................................CGUGUUCUCAGGUCGCCCCCG.......................    81.334755
......................................................................................................UGGGUUCUCAGGUCGCCCCCG.......................    51.192874
......................................................................................................UGUGUUCUCAGGUCGCCCCAG.......................    14.442211
......................................................................................................UUUGUUCUCAGGUCGCCCCCG.......................    65.589914
......................................................................................................UAUGUUCUCAGGUCGCCCCCG.......................    22.662611
......................................................................................................UGUGUUCUCAUGUCGCCCCCG.......................    67.664144
......................................................................................................UGUGUUCUCAGGUCGCCCCUG.......................    102.817921
......................................................................................................UGUGUCCUCAGGUCGCCCCCG.......................    77.971113
......................................................................................................UGUGUUCUCAGGUCGCCCUCG.......................    36.735595
......................................................................................................UGUGUUCUCAGGACGCCCCCG.......................    19.312322
......................................................................................................UGUGUUCUCAGGUCACCCCCG.......................    43.327672
......................................................................................................UGUGUUCUCAGGUCGCCCCA........................    54.689125
......................................................................................................UGCGUUCUCAGGUCGCCCCCG.......................    112.163695
......................................................................................................AGUGUUCUCAGGUCGCCCCCG.......................    31.034047
......................................................................................................UGAGUUCUCAGGUCGCCCCCG.......................    29.327977
......................................................................................................UGUGUUCCCAGGUCGCCCCCG.......................    87.151201
......................................................................................................UGUGUUCUCGGGUCGCCCCCG.......................    85.580781
......................................................................................................UGUGUUCUCAAGUCGCCCCCG.......................    16.984015
......................................................................................................UGUGUUCUCAGGUCGCCCCU........................    137.89214
......................................................................................................UGUGUUCUCAGGUCGCCCCG........................    82.191632
.....................................................................................................AUGUGUUCUCAGGUCGCCCCC........................    6678.775948
.....................................................................................................AUGUGUUCUCAGGUCGCCC..........................    42.563396
.....................................................................................................AUGUGUUCUCAGGUCGCCCCCG.......................    399.263314
.....................................................................................................AUGUGUUCUCAGGUCGCCCC.........................    75.551733
.....................................................................................................AUGUGUUCUCAGGUCGCCCCA........................    181.09146
.....................................................................................................AUGUGUUCUCAGGUCGCCCCU........................    488.295385
.....................................................................................................AUGUGUUCUCAGGUCGCCCCCA.......................    39.336594
.....................................................................................................AUGUGUUCUCAGGUCGCCCCCU.......................    82.64385
....................................................................................................CAUGUGUUCUCAGGUCGCCCC.........................    2093.704775
....................................................................................................CAUGUGUUCUCAGGUCGCCCU.........................    73.531251
....................................................................................................CAUGUGUUCUCAGGUCGCCCCU........................    24.47679
...................GGCGGACUGGGAACACAUGGG..........................................................................................................    5965.290621
...................GGCGGACUGGGAACACAUGG...........................................................................................................    190.369356
...................GGCGGACUGGGAACACAUGGGA.........................................................................................................    80.299173
...................GGCGGACUGGGAACACAU.............................................................................................................    44.181869
...................GGCGGACUGGGAACACAUG............................................................................................................    471.396783
...................GGCGGACUGGGAACACAUA............................................................................................................    23.272975
..................GGGCGGACUGGGAACACA..............................................................................................................    63.868456
..................GGGCGGACUGGGAACACAUGG...........................................................................................................    4955.545792
..................GGGCGGACUGGGAACACAU.............................................................................................................    300.332284
..................GGGCGGACUGGGAACACAUG............................................................................................................    381.421346
..................GGGCGGACUGGGAACACAC.............................................................................................................    17.62284
..................GGGCGGACUGGGAACACAUGU...........................................................................................................    26.166016
..................GGGCGGACUGGGAACACAUGA...........................................................................................................    225.291612
.................GGGGCGGACUGGGAACACAUGGGA.........................................................................................................    61.011476
.................GGGGCGGACUGGGAACAC...............................................................................................................    60.186417
.................GGGGCGGACUGGGAACACAUGG...........................................................................................................    241.58641
.................GGGGCGGACUGGGAACACAUGGG..........................................................................................................    95.268499
.................GGGGCGGACUGGGAACACAU.............................................................................................................    1111.329892
.................GGGGCGGACUGGGAACACA..............................................................................................................    1468.891188
.................GUGGCGGACUGGGAACACAUG............................................................................................................    23.307003
.................GGUGCGGACUGGGAACACAUG............................................................................................................    19.71832
.................GGGGCGGACUGGGAACACAUGC...........................................................................................................    17.470041
.................GGGGCGGACUGGGAACGCAUG............................................................................................................    14.577812
.................GGGGCGGACUGGGAACACAUGU...........................................................................................................    102.890193
.................GGGGCGGACUUGGAACACAUG............................................................................................................    25.0466
.................GGGGCGGACUGGGAACACAUGA...........................................................................................................    119.637009
.................GGGGCGGACUGGGAACACGUG............................................................................................................    10.823168
.................GGGGCGGACUGGGGACACAUG............................................................................................................    15.288387
.................GGGGCGGACUGGGAACACAUA............................................................................................................    545.951537
.................GGGGCGGACUGGGAACACAUU............................................................................................................    51.809507
.................GGGGCGGACUGGGAACACC..............................................................................................................    25.47738
```

zma-MIR399a

```
Chromosome:    chr4
Start:         233617208
End:           233617324
Strand:        -
Strand bias:   1.0
Abundance bias:1.0
MFE:           -52.30
```

```
                                      Star                                                          Mature
AAGAGGGUGAGAAUCACAGUGCGGUUCUCCUCUGGCACGAAGGCUGGCCAGAGCAGCUUAUAUUGCCACCACCCUUCCUUGCCAAAGGAGAAUUGCCCUGCCAUUCACAACCCUGCA    3816.6
...(((((..((((..(((.(((((((((((.(((((.(((((.(((...(.((((......))))).))).)))))..))))).))))))))))).)))..))))...)))))...
...............................................................................UGCCAAAGGAGAAUUGCCCUG.................    2633.716647
..................GUGCGGUUCUCCUCUGGCACG..............................................................................    941.792312
................................................................................GCCAAAGGAGAAUUGCCCUG.................    2.89319
...............................................................................UGCCAAAGGAGAAUUGCCCU..................    33.025434
...............................................................................UGCCAAAGGAGAAUUGCC....................    7.494289
...............................................................................UGCCAAAGGAGAAUUGCCC...................    10.61725
...............................................................................UGCCAAAGGAGAUUUGCCCUG.................    15.91686
...............................................................................UGCCAAAGGAGAAUUGCCCUGA................    11.7104
...............................................................................UGCCAAAGGAGAAUUGCCCUA.................    7.1316
...............................................................................UGCCAAAGGAGAAUUGCCU...................    1.08407
..................GUGCGGUUCUCCUCUGGCACGA.............................................................................    59.0192
..................GUGCGGUUCUCCUCUGGCA................................................................................    6.05917
..................GUGCGGUUCUCCUCUGGCACA..............................................................................    78.05308
..................GUGCGGUUCUCCUCUGGCACCA.............................................................................    2.27703
..................GUGCGGUUCUCCUCUGGCACGC.............................................................................    3.2529
..................GUGCGGUUCUCCUCUGGCACGU.............................................................................    2.60232
```

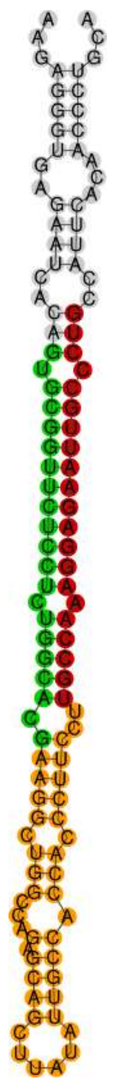

```
                  Star                                              Mature
UCAGGGUGCGAAUCACAGUGCAGCUCUCCUCUGGCAUGAAGGCUGUGAGAGAGGCAUGACAAUUUCUGGCCUUGCCCUGCCAAAGGAGAGCUGUCCUGCCAUUCAUUAGCCCUGC    4840.
.((((((..((((..(((.(((((((((((.(((((..(((((((.((((..(......)..)))))))))))....))))).))))))))))).)))..))))....)))))).
.............................................................................UGCCAAAGGAGAGCUGUCCUG.................    1661.78634
.................GUGCAGCUCUCCUCUGGCAUG.............................................................................    2880.215795
.............................................................................UGCCAAAGGAGAGCUGUCCU..................    135.101729
.............................................................................UGCCAAAGGAGAGCUGUCCUA.................    4.24032
.............................................................................UGCCAAAGGAGAGCUGUCCUC.................    2.16664
.............................................................................UGCCAAAGGAGAGCUGUCCUU.................    0.843162
.............................................................................UGCCAAAGGAGAGCUGUCCUGA................    8.13224
.............................................................................UGCCAAAGGAGAGUUGUCCUG.................    16.18777
..................UGCAGCUCUCCUCUGGCAUG.............................................................................    18.40787
.................GUGCAGCUCUCCUCUGGCAUGA............................................................................    76.53018
.................GUGCAGCUCUCCUCUGGCA...............................................................................    8.56093
.................GUGCAGCUCUCCUCUGGCAUGC............................................................................    1.95174
.................GUGCAGCUCUCCUCUGGCAUA.............................................................................    11.70076
.................GUGCAGCUCUUCUCUGGCAUG.............................................................................    4.01655
................AGUGCAGCUCUCCUCUGGCAUG.............................................................................    5.20464
```

zma-MIR399b

Chromosome: chr4
Start: 158285686
End: 158285800
Strand: +
Strand bias: 1.0
Abundance bias: 0.999
MFE: -56.80

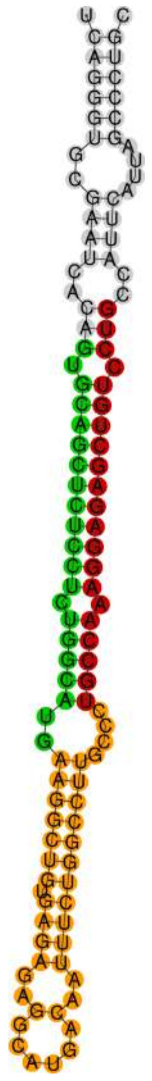

zma-MIR399c

Chromosome: chr6
Start: 163867937
End: 163868136
Strand: +
Strand bias: 1.0
Abundance bias: 0.999
MFE: -99.90

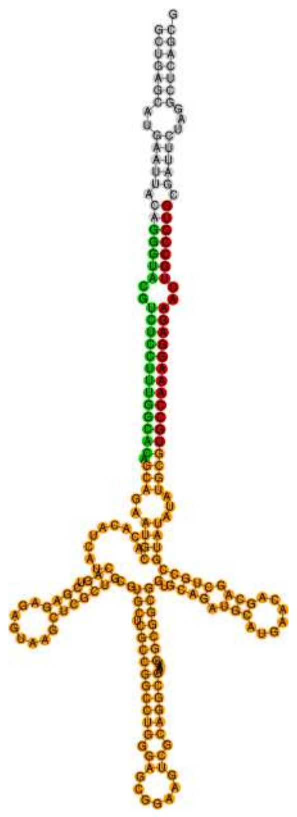

```
                 Star                                                                                                                                     Mature
GCUGAGCAUGAAUUACAGGGUACGUCUCCUUUGGCACAGCAGAAUGCACACAUCAUCAGUCGAGAGAGUAAGCUCGCUGCGUGCUCGCCGGCCUGGGAGCGGAAGUCGCAGGCCAAAUGGCGGCGGUGCAGAUGCAUGAACAGCAGCUGCCGUAUAUAUGCGUGCCAAAGGAGAAUUGCCCUGCGAUUCUAGGCUCAGCG    23453.1
(((((((..(((((.(((((((..(((((((((((((.(((..((((.........(((.((((........)))))))..(((.((((((((((.((.......)).))))))....)))))))(.((((.(((.......))).)))))))))...))))))))))))))))..))))))).)))))...))))))).
..................................................................................................................................................................UGCCAAAGGAGAAUUGCCCUG.................    2633.716647
.................GGGUACGUCUCCUUUGGCACA..................................................................................................................................................................    20415.427412
...................................................................................................................................................................GCCAAAGGAGAAUUGCCCUG.................    2.89319
..................................................................................................................................................................UGCCAAAGGAGAAUUGCCCU..................    33.025434
..................................................................................................................................................................UGCCAAAGGAGAAUUGCC....................    7.494289
..................................................................................................................................................................UGCCAAAGGAGAAUUGCCC...................    10.61725
..................................................................................................................................................................UGCCAAAGGAGAUUUGCCCUG.................    15.91686
..................................................................................................................................................................UGCCAAAGGAGAAUUGCCCUGA................    11.7104
..................................................................................................................................................................UGCCAAAGGAGAAUUGCCCUA.................    7.1316
..................................................................................................................................................................UGCCAAAGGAGAAUUGCCU...................    1.08407
....................UACGUCUCCUUUGGCACA..................................................................................................................................................................    5.30177
..................GGUACGUCUCCUUUGGCACA..................................................................................................................................................................    4.54438
.................GGGUACGUCUCCUUUGGC.....................................................................................................................................................................    4.89774
.................GGGUACGUCUCCUUUGGCA....................................................................................................................................................................    2.60232
.................GGGUACGUCUCCUUUGGCAC...................................................................................................................................................................    186.32083
.................GGGUACGUCUCCCUUGGCACA..................................................................................................................................................................    6.81657
.................GGGUACGCCUCCUUUGGCACA..................................................................................................................................................................    2.27703
.................GGGUACGUCCCCUUUGGCACA..................................................................................................................................................................    1.95174
.................GGGUACGUCUCCUCUGGCACA..................................................................................................................................................................    1.95174
.................GGGUACGUCUCCUUUGGUACA..................................................................................................................................................................    8.12257
.................GGGCACGUCUCCUUUGGCACA..................................................................................................................................................................    5.30177
.................GGGUACGUCUCCUUUGGCACAA.................................................................................................................................................................    5.20464
.................GGGUACGUCUCCUUUGGCACC..................................................................................................................................................................    2.27703
.................GGGUACGUCUCCUUUGGCACU..................................................................................................................................................................    13.68056
................AGGGUACGUCUCCUUUGGCACA..................................................................................................................................................................    45.9438
```

zma-MIR399d

Chromosome: chr3
Start: 192096065
End: 192096187
Strand: +
Strand bias: 1.0
Abundance bias: 1.0
MFE: -53.50

```
                 Star                                                                  Mature
UCAAGGUGAGAAUCACAGUGUGGCUCUCCUCUGGCAUGAAGGCGCGGCUGAGAGGCACGAACAGUUUCUGGCCUUCCCCUGCCAAUGCCAAAGGAGAGCUGCCCUGCCAUUCGUUAGCCCUGC    2402.0
.((.(((..((((..(((.(((((((((((.((((((...((((.((....(((((..(((....)))..)))))..)))))).)))))).))))))))))).)))..))))....))).)).
.....................................................................................UGCCAAAGGAGAGCUGCCCUG.................    398.566179
.................GUGUGGCUCUCCUCUGGCAUG.....................................................................................    1822.706719
.....................................................................................UGCCAAAGGAGAGCUGCCCU..................    102.773615
.....................................................................................UGCCAAAGGAGAGCUGCCCUU.................    2.16664
.................GUGUGGCUCUCCUCUGGCAUGA....................................................................................    31.40952
.................GUGUGGCUCUCCUCUGGCAU......................................................................................    4.69762
.................GUGUGGCUCUCCUCUGGCA.......................................................................................    4.68598
.................GUGUGGCUCUCCUCUGGCAUGC....................................................................................    8.43162
.................GUGUGGCUCUCCUCUGGCAUGU....................................................................................    9.39524
.................GUGUGGCUCUCUUCUGGCAUG.....................................................................................    7.36368
.................GUGUGGCUCUCCUCUGGCAUA.....................................................................................    9.89
```

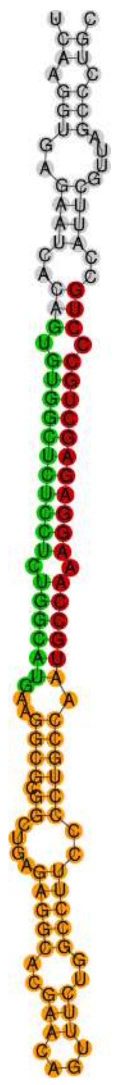

zma-MIR399e

Chromosome: chr2
Start: 16763311
End: 16763428
Strand: -
Strand bias: 1.0
Abundance bias: 1.0
MFE: -57.40

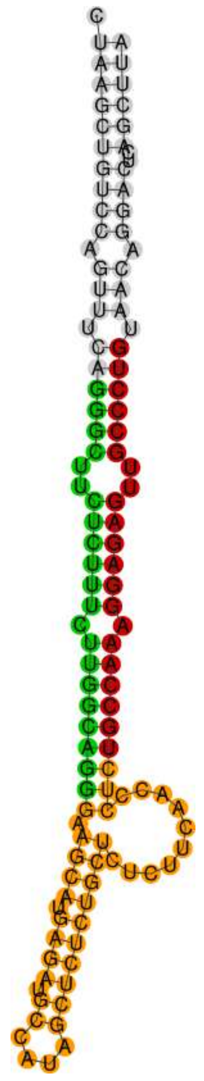

```
                   Star                                                      Mature
CUAAGCUGUCCAGUUUCAGGGCUUCUCUUUCUUGGCAGGGAAGCAUGAGAUGCCAUAGCUCUCUGCUCUCUUCAACCCUCUGCCAAAGGAGAGUUGCCCUGUAACAGGACUCAGCUUA    28387.9
.((((((((((.(((.((((((..((((((.(((((((((.((((.((((.((....))))))))))..........))))))))).))))))..)))))).))).)))).))))))
................................................................................UGCCAAAGGAGAGUUGCCCUG.................    22946.624303
..................GGGCUUCUCUUUCUUGGCAGG...............................................................................    487.095422
...................................................................................CAAAGGAGAGUUGCCCUG.................    15.93729
...................................................................................CAAAGGAGAGUUGCCCUU.................    8.1585
.................................................................................GCCAAAGGAGAGUUGCCCUG.................    17.87973
.................................................................................GCCAAAGGAGAGUUGCCCU..................    2.70031
................................................................................UGCCAAAGGAGAGUUGCCCU..................    3825.479119
................................................................................UGCCAAAGGAGAGUUGCCCUGU................    251.318698
................................................................................UGCCAAAGGAGAGUUGCC....................    57.15644
................................................................................UGCCAAAGGAGAGUUGCCC...................    93.159229
................................................................................UUCCAAAGGAGAGUUGCCCUG.................    5.65725
................................................................................UGCCAAAGGAGAGUUGCCCG..................    0.963614
................................................................................UGCCAAAGGAGAGUUUCCCUG.................    29.48094
................................................................................UGCCAAAGGAGAGUUGACCUG.................    1.08407
................................................................................UGCCAAAGGGGAGUUGCCCUG.................    0.963614
................................................................................UGCCAAAGGAGGGUUGCCCUG.................    1.32497
................................................................................UGCCAAAGGAGAGUUGCCA...................    1.80678
................................................................................UGCCAAAGGAGAGUUGCCCC..................    19.243644
................................................................................UGCCAAAGGAGAGUUGCCCA..................    10.730025
................................................................................UGCCAAAGGAGAGUUGCCCUGA................    13.655326
................................................................................UGCCAAAGGAGAGUUGGCCUG.................    0.72271
................................................................................UGCCAAAGGAGAGUUGCCCCG.................    2.719412
................................................................................UGCCAAAGGAGAGUUGCCUUG.................    5.26639
................................................................................UGCCAAAGGAGAUUUGCCCUG.................    15.91686
................................................................................CGCCAAAGGAGAGUUGCCCUG.................    1.32497
................................................................................UGCCAAAGGAGAGUUGCU....................    0.963614
................................................................................UGCCAAAGGAGAGUUGCCCUC.................    2.04768
................................................................................UGCCAAAGGAGAGUUGCCCUA.................    327.004254
................................................................................UGCCAAAGGAGAGUGGCCCUG.................    1.62498
................................................................................UGCCAAAGGAGAGUUGCCU...................    6.391255
................................................................................UGCCAAAGGAGAGUCGCCCUG.................    1.32497
................................................................................UGCCAAGGGAGAGUUGCCCUG.................    0.963614
................................................................................GGCCAAAGGAGAGUUGCCCUG.................    2.49113
................................................................................UGCCAAAUGAGAGUUGCCCUG.................    10.16091
................................................................................UGCCAAAGGAGUGUUGCCCUG.................    22.08632
................................................................................UGCCAAAGGAGAGUUGCUCUG.................    22.527872
................................................................................UGCCAAAGGAGAGUUGCCAUG.................    5.77123
................................................................................UGCCAAAGGAGAGUUGCCCGG.................    22.935655
................................................................................UGCCAAAGGAGAGUUGCCCUU.................    52.609515
................................................................................UGCCAAAGUAGAGUUGCCCUG.................    13.58705
................................................................................UGCCAAAGGAUAGUUGCCCUG.................    21.30165
................................................................................UGCCAAAGGUGAGUUGCCCUG.................    2.59037
................................................................................UGCAAAAGGAGAGUUGCCCUG.................    6.09243
................................................................................UACCAAAGGAGAGUUGCCCUG.................    0.963614
................................................................................UGCCAAAGGAGAGUUGUCCUG.................    16.18777
...............................................................................CUGCCAAAGGAGAGUUGCCCUG.................    4.12665
..............................................................................UCUGCCAAAGGAGAGUUGCCC...................    5.77973
..................GGGCUUCUCUUUCUUGGCAGGGA.............................................................................    4.837047
..................GGGCUUCUCUUUCUUGGCA.................................................................................    0.713614
..................GGGCUUCUCUUUCUUGGCAGGG..............................................................................    0.501437
..................GGGCUUCUCUUUCUUGGCAGGA..............................................................................    0.706844
..................GGGCUUCUCUUUCUUGGCAGA...............................................................................    1.95174
.................AGGGCUUCUCUUUCUUGGCAG................................................................................    4.01655
.................AGGGCUUCUCUUUCUUGGCA.................................................................................    1.87625
...............UCAGGGCUUCUCUUUCUUGGC..................................................................................    7.48921
```

zma-MIR399f

```
Chromosome:    chr6
Start:         163860157
End:           163860259
Strand:        -
Strand bias:   1.0
Abundance bias:0.981
MFE:           -47.90
```

```
                    Star                                       Mature
GGAUGUGCACCUGCAUUUCUGGGCAACUUCUCCUUUGGCAGAUGCGCGAGAAUGUGCCACUGCUGCCAAAGGAAAUUUGCCCCGGAAUUCAUCUGCACAAGCA    1993.2
(..((((((..((.(((((.((((((.((.((((((((((..((((......)))).....)))))))))))))).)))))).))))).))..))))))..).
...............................................................UGCCAAAGGAAAUUUGCCCCG...................    930.904852
....................GGGCAACUUCUCCUUUGGCAGA.............................................................    1013.104148
................................................................GCCAAAGGAAAUUUGCCCCG...................    1.15727
...............................................................UGCCAAAGGAAAUUUGCCCC....................    5.843215
...............................................................UGCCAAAGGAAAUUUGCCCCGA..................    2.92761
....................GGGCAACUUCUCCUUUGGCAGG.............................................................    0.963614
```

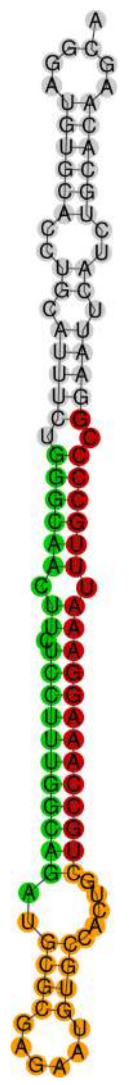

zma-MIR399g

Chromosome: chr4
Start: 233618387
End: 233618490
Strand: +
Strand bias: 1.0
Abundance bias: 1.0
MFE: -60.80

```
                    Star                                          Mature
GAGUGUGGAGGUCCAUUGCAGGGCAACCCCCGUUGGCAGGUGCGACGGCGACGUACCCCUACCCUGCCAAAGGGGAUUUGCCCGGCGAUGCGCCUGCACACAC    892.8
..(((((.((((.((((((.((((((..((((.((((((((((.((((....))).)...)).))))))))).))))..)))))).)))))).)))).)))))..
................................................................UGCCAAAGGGGAUUUGCCCGG..................    871.28864
....................GGGCAACCCCCGUUGGCAGG...............................................................    15.635626
.................................................................GCCAAAGGGGAUUUGCCCGGC.................    2.423406
................................................................UGCCAACGGGGAUUUGCCCGG..................    0.702532
................................................................UGCCAAAGGGGGUUUGCCCGG..................    0.702532
................................................................UGCCAAAGGAGAUUUGCCCGG..................    2.066517
```

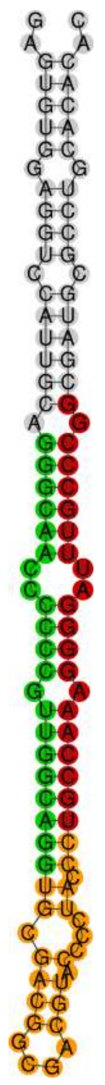

zma-MIR399h

Chromosome: chr5
Start: 150363079
End: 150363203
Strand: +
Strand bias: 1.0
Abundance bias:1.0
MFE: -54.50

```
                 Star                                                              Mature
CUAGGGUGAGAAUCACAGUGCAGUUCUCCUCUGGCACGAACCAAGGCUGAGCAGCUUGCCCCAUGGCUAGCCCACGACGGCCCUUCCUUGCCAAAGGAGAAUUGCCCUGCCAUUCACAACCCUAC    2769.9
.((((((..((((..(((.(((((((((((.(((((.(((....(((((....(((.(((....))).)))......))))).)))..))))).))))))))))).)))..))))...)))))).
........................................................................................UGCCAAAGGAGAAUUGCCCUG................    2633.716647
.................GUGCAGUUCUCCUCUGGCACG.......................................................................................    46.35298
.........................................................................................GCCAAAGGAGAAUUGCCCUG................    2.89319
........................................................................................UGCCAAAGGAGAAUUGCCCU.................    33.025434
........................................................................................UGCCAAAGGAGAAUUGCC...................    7.494289
........................................................................................UGCCAAAGGAGAAUUGCCC..................    10.61725
........................................................................................UGCCAAAGGAGAUUUGCCCUG................    15.91686
........................................................................................UGCCAAAGGAGAAUUGCCCUGA...............    11.7104
........................................................................................UGCCAAAGGAGAAUUGCCCUA................    7.1316
........................................................................................UGCCAAAGGAGAAUUGCCU..................    1.08407
```

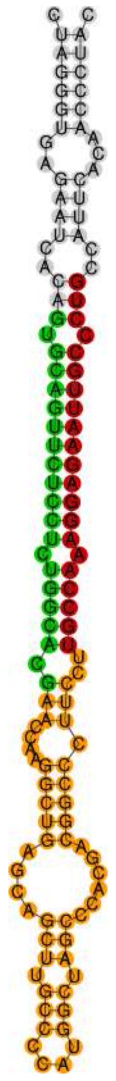

zma-MIR399j

Chromosome: chr9
Start: 24309462
End: 24309670
Strand: -
Strand bias: 1.0
Abundance bias: 0.97
MFE: -123.00

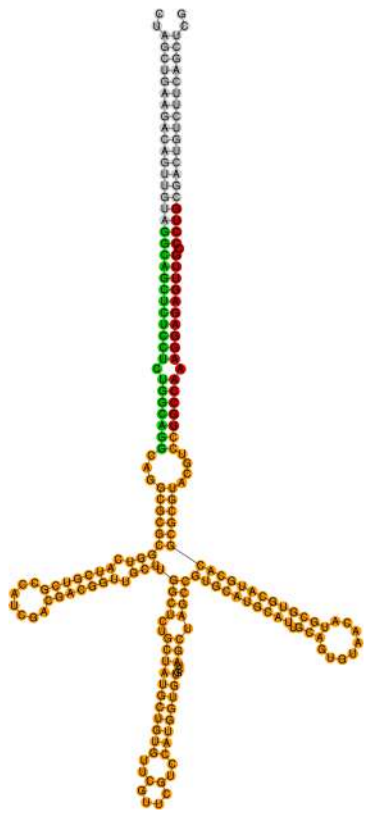

```
                 Star                                                                                                                                                                       Mature
CUAGCUGAAGACAGUUGUAGGCAGCUCUCCUCUGGCAGGCAGGCGCGCGGUCAUCGUCGCCAUCGACGACGGUUGCUUGGCUCUGCUAUGCUGUGUUCGUUCGUCCAUGGUGUGCUAGCUAGCCGUGCAUGCAUUGCAGUGUAACAUGCGUGCAUGCACGCGCGUACGUCCUGCCAAAGGAGAGUUGCCCUGCGACUGUCUUCAGCUCG    29846.0
..(((((((((((((((((((((((((((.(((((((...((((((((.(((((((.......))))))).))).((((..((((((((((..(....)..))))))))...))).))))(((((((((.(((........))))))))))))))))))....))))))).))))))))).)))))))))))))))))..
...........................................................................................................................................................................UGCCAAAGGAGAGUUGCCCUG.................    22946.624303
...................GGCAGCUCUCCUCUGGCAGG..........................................................................................................................................................................    1203.337455
..............................................................................................................................................................................CAAAGGAGAGUUGCCCUG.................    15.93729
..............................................................................................................................................................................CAAAGGAGAGUUGCCCUU.................    8.1585
............................................................................................................................................................................GCCAAAGGAGAGUUGCCCUG.................    17.87973
............................................................................................................................................................................GCCAAAGGAGAGUUGCCCU..................    2.70031
...........................................................................................................................................................................UGCCAAAGGAGAGUUGCCCU..................    3825.479119
...........................................................................................................................................................................UGCCAAAGGAGAGUUGCC....................    57.15644
...........................................................................................................................................................................UGCCAAAGGAGAGUUGCCC...................    93.159229
...........................................................................................................................................................................UUCCAAAGGAGAGUUGCCCUG.................    5.65725
...........................................................................................................................................................................UGCCAAAGGAGAGUUGCCCG..................    0.963614
...........................................................................................................................................................................UGCCAAAGGAGAGUUUCCCUG.................    29.48094
...........................................................................................................................................................................UGCCAAAGGAGAGUUGACCUG.................    1.08407
...........................................................................................................................................................................UGCCAAAGGGGAGUUGCCCUG.................    0.963614
...........................................................................................................................................................................UGCCAAAGGAGGGUUGCCCUG.................    1.32497
...........................................................................................................................................................................UGCCAAAGGAGAGUUGCCA...................    1.80678
...........................................................................................................................................................................UGCCAAAGGAGAGUUGCCCC..................    19.243644
...........................................................................................................................................................................UGCCAAAGGAGAGUUGCCCA..................    10.730025
...........................................................................................................................................................................UGCCAAAGGAGAGUUGCCCUGA................    13.655326
...........................................................................................................................................................................UGCCAAAGGAGAGUUGGCCUG.................    0.72271
...........................................................................................................................................................................UGCCAAAGGAGAGUUGCCCCG.................    2.719412
...........................................................................................................................................................................UGCCAAAGGAGAGUUGCCUUG.................    5.26639
...........................................................................................................................................................................UGCCAAAGGAGAUUUGCCCUG.................    15.91686
...........................................................................................................................................................................CGCCAAAGGAGAGUUGCCCUG.................    1.32497
...........................................................................................................................................................................UGCCAAAGGAGAGUUGCU....................    0.963614
...........................................................................................................................................................................UGCCAAAGGAGAGUUGCCCUC.................    2.04768
...........................................................................................................................................................................UGCCAAAGGAGAGUUGCCCUA.................    327.004254
...........................................................................................................................................................................UGCCAAAGGAGAGUGGCCCUG.................    1.62498
...........................................................................................................................................................................UGCCAAAGGAGAGUUGCCU...................    6.391255
...........................................................................................................................................................................UGCCAAAGGAGAGUCGCCCUG.................    1.32497
...........................................................................................................................................................................UGCCAAGGGAGAGUUGCCCUG.................    0.963614
...........................................................................................................................................................................GGCCAAAGGAGAGUUGCCCUG.................    2.49113
...........................................................................................................................................................................UGCCAAAUGAGAGUUGCCCUG.................    10.16091
...........................................................................................................................................................................UGCCAAAGGAGUGUUGCCCUG.................    22.08632
...........................................................................................................................................................................UGCCAAAGGAGAGUUGCUCUG.................    22.527872
...........................................................................................................................................................................UGCCAAAGGAGAGUUGCCAUG.................    5.77123
...........................................................................................................................................................................UGCCAAAGGAGAGUUGCCCGG.................    22.935655
...........................................................................................................................................................................UGCCAAAGGAGAGUUGCCCUU.................    52.609515
...........................................................................................................................................................................UGCCAAAGUAGAGUUGCCCUG.................    13.58705
...........................................................................................................................................................................UGCCAAAGGAUAGUUGCCCUG.................    21.30165
...........................................................................................................................................................................UGCCAAAGGUGAGUUGCCCUG.................    2.59037
...........................................................................................................................................................................UGCAAAAGGAGAGUUGCCCUG.................    6.09243
...........................................................................................................................................................................UACCAAAGGAGAGUUGCCCUG.................    0.963614
...........................................................................................................................................................................UGCCAAAGGAGAGUUGUCCUG.................    16.18777
..........................................................................................................................................................................CUGCCAAAGGAGAGUUGCCCUG.................    4.12665
...................GGCAGCUCUCCUCUGGCAGGCA........................................................................................................................................................................    3.2529
...................GGCAGCUCUCCUCUGGCAGGC.........................................................................................................................................................................    11.02751
...................GGCAGCUCUCCUCUGGCA............................................................................................................................................................................    46.862507
...................GGCAGCUCUCCUCUGGCAGGA.........................................................................................................................................................................    14.294173
...................GGCAGCUCUCCUCUGGCU............................................................................................................................................................................    2.49113
...................GGCAGCUCUCCUCUGGCAGC..........................................................................................................................................................................    5.52993
...................GGCAGCUCUCCUCUGGCAGA..........................................................................................................................................................................    6.3969
...................GUCAGCUCUCCUCUGGCAGG..........................................................................................................................................................................    2.61104
.................UAGGCAGCUCUCCUCUGGC.............................................................................................................................................................................    12.63742
.................UAGGCAGCUCUCCUCUGGCAGG..........................................................................................................................................................................    6.14304
```

zma-MIR408a

Chromosome: chr3
Start: 58127892
End: 58128093
Strand: -
Strand bias: 1.0
Abundance bias: 0.992
MFE: -88.20

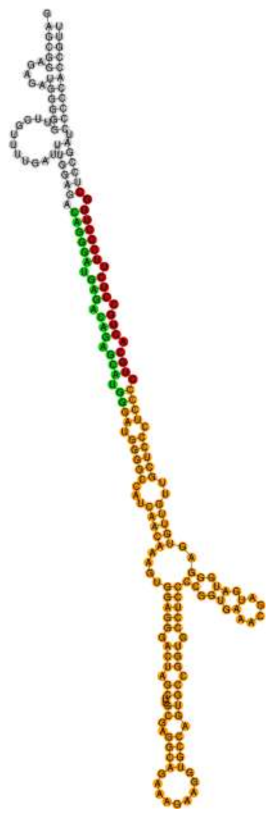

```
                                   Star                                                                                                                                   Mature
GAGCGGAGAGAUGGGGGUUGGUUUUGAUUUGGAGACAGGGAUGAGACAGAGCAUGGGAUGGGGCCAUCAACAAAGUGGAGGGACUAGCUUGCGAGGCAGAAAGAAGGUGCCAGUGCCGGUGCCUCCCCGGUGAAACGAUGAUGGGAGUGUUGUUGCUCCCUCCCCUGCACUGCCUCUUCCCUGGCUCCGAUCCCCACCGUU 413406.8
.(((((.....((((((..........((((((.((((((.(((.(((.(((.((((.(((((...(((((....(((((.(((.((..((..((((.........)))).)))).))).)))))((.((.(.....).)).))..)))))..))))).)))).))).))).))).)))))).)))))).)))))))))))
....................................................................................................................................................................CUGCACUGCCUCUUCCCUGGC................ 169678.0911
...................................CAGGGAUGAGACAGAGCAUGG................................................................................................................................................. 27958.976812
.......................................................................................................................................................................CACUGCCUCUUCCCUGGCUCC............. 482.279129
.....................................................................................................................................................................UGCACUGCCUCUUCCCUGGCU............... 9987.765641
.....................................................................................................................................................................UGCACUGCCUCUUCCCUG.................. 527.593617
.....................................................................................................................................................................UGCACUGCCUCUUCCCUGGC................ 71030.142012
.....................................................................................................................................................................UGCACUGCCUCUUCCCUGG................. 867.055173
.....................................................................................................................................................................UGCACUGCCUCUUCCCUGGCUC.............. 1925.325842
.....................................................................................................................................................................UGCACUGCCUCUUCCCUGGCA............... 617.037772
.....................................................................................................................................................................UGCACUGCCUCUUCCCUU.................. 673.94389
.....................................................................................................................................................................UGCACUGCCUCUUCCCUGGCC............... 533.455662
.....................................................................................................................................................................UGCACUGCCUCUUCCCUGAC................ 315.393769
.....................................................................................................................................................................UGCACUGCUUCUUCCCUGGC................ 224.503785
.....................................................................................................................................................................UGCACUGCCUCUUCCCUGCC................ 292.61188
.....................................................................................................................................................................UGCACUGCCUCUUCCCUGGU................ 11162.523051
.....................................................................................................................................................................UGCACUGCCUCUUCCCUGGA................ 3110.365334
....................................................................................................................................................................CUGCACUGCCUCUUCCCU................... 1128.277885
....................................................................................................................................................................CUGCACUGCCUCUUCCCUG.................. 232.796091
....................................................................................................................................................................CUGCACUGCCUCUUCCCUGGCU............... 1074.519951
....................................................................................................................................................................CUGCACUGCCUCUUCCCUGG................. 786.307117
....................................................................................................................................................................CUGCACUGCCUCUUCCCUGGCUCC............. 1129.329395
....................................................................................................................................................................CUGCACUGCCUCUUCCCUGGU................ 29656.508409
....................................................................................................................................................................CUGCACUGCCUCUUCCCUGGG................ 591.948426
....................................................................................................................................................................CUGCACUGCCUCUUCCCUGGA................ 8284.40701
....................................................................................................................................................................CUGCACUGCCUCUUCCCUGGCA............... 245.985806
....................................................................................................................................................................CUGCACUGCCUCUUCCCUGCC................ 784.853752
....................................................................................................................................................................CUGCACUGCCUCUCCCCUGGC................ 268.583571
....................................................................................................................................................................CUGCACUGCUUCUUCCCUGGC................ 399.054841
....................................................................................................................................................................CGGCACUGCCUCUUCCCUGGC................ 476.24152
....................................................................................................................................................................CUGCACUGCCUCUUCUCUGGC................ 290.852554
....................................................................................................................................................................CUGCACUGCCUCUUCCCCGGC................ 618.705272
....................................................................................................................................................................CUGCACUGUCUCUUCCCUGGC................ 456.035209
....................................................................................................................................................................CAGCACUGCCUCUUCCCUGGC................ 334.866756
....................................................................................................................................................................CUGCGCUGCCUCUUCCCUGGC................ 369.978326
....................................................................................................................................................................CUGCACCGCCUCUUCCCUGGC................ 293.504876
....................................................................................................................................................................CUGCACUGCCUCCUCCCUGGC................ 391.546641
....................................................................................................................................................................CUGCACUGCCCCUUCCCUGGC................ 395.703637
....................................................................................................................................................................CCGCACUGCCUCUUCCCUGGC................ 757.331807
....................................................................................................................................................................CUGCACUGCCUCUUCCCUGAC................ 686.197061
...................................CAGGGAUGAGACAGAGCAU................................................................................................................................................... 2647.588095
...................................CAGGGAUGAGACAGAGCAUGGG................................................................................................................................................ 267.798633
...................................CAGGGAUGAGACAGAGCA.................................................................................................................................................... 345.891499
...................................CAGGGAUGAGACAGAGCAUG.................................................................................................................................................. 15364.03935
...................................CAGGGAUGAGACAGAGCAUA.................................................................................................................................................. 333.634992
...................................CAGGGAUGAGACAGAGCAUGGU................................................................................................................................................ 3005.233305
...................................CAGGGAUGAGACAGAGCAUGGA................................................................................................................................................ 677.917255
...................................CAGGGAUGAGACAGAGCAUGGC................................................................................................................................................ 270.834316
...................................CAGGGAUGAGACAGAGCAUGU................................................................................................................................................. 398.092379
...................................CAGGGAUGAGACAGAGCAUGA................................................................................................................................................. 1196.063623
...................................CAGGGAUGAGGCAGAGCAUGG................................................................................................................................................. 482.681549
..................................ACAGGGAUGAGACAGAGCAUGG................................................................................................................................................. 578.84835
..................................ACAGGGAUGAGACAGAGCAU................................................................................................................................................... 315.578133
..................................ACAGGGAUGAGACAGAGCAUG.................................................................................................................................................. 22539.047116
..................................ACAGGGAUGAGACAGAGCAUA.................................................................................................................................................. 246.159281
.................................GACAGGGAUGAGACAGAGCAU................................................................................................................................................... 1422.301906
.................................GACAGGGAUGAGACAGAGCAUG.................................................................................................................................................. 427.393242
................................AGACAGGGAUGAGACAGAGCA.................................................................................................................................................... 1986.219466
................................AGACAGGGAUGAGACAGAGCAUGG................................................................................................................................................. 467.209323
```

zma-MIR408b

Chromosome: chr8
Start: 39583923
End: 39584105
Strand: -
Strand bias: 1.0
Abundance bias: 0.997
MFE: -77.30

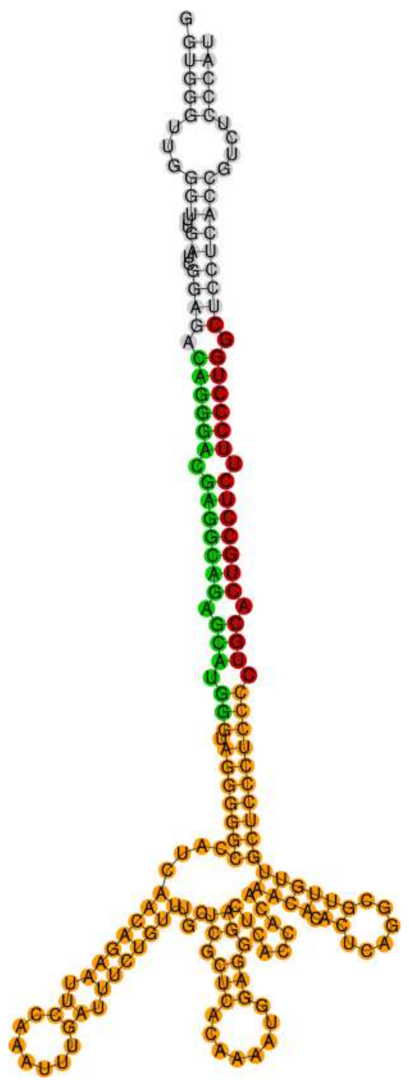

```
                                Star                                                                                                                        Mature
GGUGGGUUGGGUUUGAUCGGAGACAGGGACGAGGCAGAGCAUGGGUAGGGGGCCAUCAACAGAAUUCCAAAUUUGAUUUCUGUUUGCUCGCUCACAAAAUGGAGGGACUCACCACAAACACACUCAGGCGUUGUUGCUCCCUCCCCUGCACUGCCUCUUCCCUGGCUCCUCACCGUCUCCCAU 343207.8
.(((((...(((..((..((((.((((((.(((((((.(((.(((.(((((((....(((((((.((.......)).)))))))...((.((((........))).)).........((((.((......)))))))))))))))).))).))))))).)))))).)))))))))....)))))
.................................................................................................................................................CUGCACUGCCUCUUCCCUGGC................. 169678.0911
.......................CAGGGACGAGGCAGAGCAUGG........................................................................................................................................... 4308.876387
....................................................................................................................................................CACUGCCUCUUCCCUGGCUCC.............. 482.279129
...................................................................................................................................................GCACUGCCUCUUCCCUGGCUC............... 79.183633
...................................................................................................................................................GCACUGCCUCUUCCCUGGCU................ 73.307284
...................................................................................................................................................GCACUGCCUCUUCCCUGGC................. 145.820016
..................................................................................................................................................UGCACUGCCUCUUCCCUGGCU................ 9987.765641
..................................................................................................................................................UGCACUGCCUCUUCCCUG................... 527.593617
..................................................................................................................................................UGCACUGCCUCUUCCCUGGC................. 71030.142012
..................................................................................................................................................UGCACUGCCUCUUCCCUGG.................. 867.055173
..................................................................................................................................................UGCACUGCCUCUUCCCUGGCUC............... 1925.325842
..................................................................................................................................................UGCACUGCCUCUUCCCUGGCA................ 617.037772
..................................................................................................................................................UGCACUGCCUCUUCCCUGGCG................ 148.307967
..................................................................................................................................................UGCACUGCCUCUUCCCUC................... 78.078172
..................................................................................................................................................UGCACUGCCUCUUCCCUA................... 169.27748
..................................................................................................................................................UGCACUGCCUCUUCCCUU................... 673.94389
..................................................................................................................................................UGCACUGCCUCCUCCCUGGC................. 105.374163
..................................................................................................................................................UGCACUGCCUCUUCCCUGGCC................ 533.455662
..................................................................................................................................................UGCACUGCCCCUUCCCUGGC................. 112.199024
..................................................................................................................................................UGCACUGCCUCUUCCCUGGUU................ 65.31663
..................................................................................................................................................UGCACUGCCUCUUCCCUGAC................. 315.393769
..................................................................................................................................................UGCACUGCUUCUUCCCUGGC................. 224.503785
..................................................................................................................................................UGCACUGCCUCUUCCCUGU.................. 164.453111
..................................................................................................................................................UGCACUGCCUCUUCCCUGA.................. 69.566665
..................................................................................................................................................UGCACUGCCUCUUCCCCGGC................. 197.116075
..................................................................................................................................................UGCACUGCCUCUUCCCUGCC................. 292.61188
..................................................................................................................................................UGCACUGCCUCUUCCCGGGC................. 53.80286
..................................................................................................................................................UGCACUGUCUCUUCCCUGGC................. 196.527627
..................................................................................................................................................UGCACUGCCUCUCCCCUGGC................. 93.671207
..................................................................................................................................................UGCACUGCCUCUUCUCUGGC................. 120.24067
..................................................................................................................................................UGCACUGCCUCUUCCCUGUC................. 65.731916
..................................................................................................................................................UGCACUGCCUCUUCCCUGGU................. 11162.523051
..................................................................................................................................................UGCACUGCCUCUUCCCUGGA................. 3110.365334
..................................................................................................................................................UGCACUGCCUCUUCCCUGGG................. 211.87166
.................................................................................................................................................CUGCACUGCCUCUUCCCU.................... 1128.277885
.................................................................................................................................................CUGCACUGCCUCUUCCCUG................... 232.796091
.................................................................................................................................................CUGCACUGCCUCUUCCCUGGCU................ 1074.519951
.................................................................................................................................................CUGCACUGCCUCUUCCCUGG.................. 786.307117
.................................................................................................................................................CUGCACUGCCUCUUCCCUGGCUCC.............. 1129.329395
.................................................................................................................................................CUGUACUGCCUCUUCCCUGGC................. 206.412125
.................................................................................................................................................CUGCACUUCCUCUUCCCUGGC................. 79.758229
.................................................................................................................................................CUGCACUGCCUCUUCCCA.................... 91.793771
.................................................................................................................................................CUGCACUGCCUCUUCCCC.................... 135.460125
.................................................................................................................................................CUGCACUGCCUCUUCCCUGGU................. 29656.508409
.................................................................................................................................................CUGCACUGCCUCUUCCCUGGG................. 591.948426
.................................................................................................................................................CUGCACUGCCUCUUCCCUGGA................. 8284.40701
.................................................................................................................................................CUGCACUGCCUCUUCCCUGGCC................ 131.102688
.................................................................................................................................................CUGCACUGCCUCUUCCUUGGC................. 112.31915
.................................................................................................................................................CUGCACUGCCUCUUCCCUGGCA................ 245.985806
.................................................................................................................................................CUGCACUGCCUCUUUCCUGGC................. 85.337999
.................................................................................................................................................AUGCACUGCCUCUUCCCUGGC................. 223.623851
.................................................................................................................................................CUGCACUGCCUCUUCCCUGCC................. 784.853752
.................................................................................................................................................CUGCACUGCCUUUUCCCUGGC................. 102.188385
.................................................................................................................................................CUGCACAGCCUCUUCCCUGGC................. 62.75306
.................................................................................................................................................CUGCACUGCCUCUCCCCUGGC................. 268.583571
.................................................................................................................................................CUGAACUGCCUCUUCCCUGGC................. 71.580071
.................................................................................................................................................CUGCACUGCUUCUUCCCUGGC................. 399.054841
.................................................................................................................................................CUGCACUGCCUCUUCCCUUGC................. 86.52449
.................................................................................................................................................CGGCACUGCCUCUUCCCUGGC................. 476.24152
.................................................................................................................................................CUGCACUGCCUCUUCUCUGGC................. 290.852554
.................................................................................................................................................CUACACUGCCUCUUCCCUGGC................. 105.545352
.................................................................................................................................................CUGCACUGCCUCUUCCCUAGC................. 141.861239
.................................................................................................................................................CUUCACUGCCUCUUCCCUGGC................. 104.342202
.................................................................................................................................................CUGCACUGCCUCUUCCCAGGC................. 92.728404
.................................................................................................................................................UUGCACUGCCUCUUCCCUGGC................. 145.834666
.................................................................................................................................................CUGCACUGCCUCUUCCAUGGC................. 93.563332
.................................................................................................................................................CUGCACUGCCUCUUCCCCGGC................. 618.705272
.................................................................................................................................................CUGCACUGCCACUUCCCUGGC................. 45.007513
.................................................................................................................................................CUGCACUGUCUCUUCCCUGGC................. 456.035209
.................................................................................................................................................CAGCACUGCCUCUUCCCUGGC................. 334.866756
.................................................................................................................................................CUGCACUGCCUCUUCCCGGGC................. 185.578987
.................................................................................................................................................CUGCAUUGCCUCUUCCCUGGC................. 88.541219
.................................................................................................................................................CUGCGCUGCCUCUUCCCUGGC................. 369.978326
.................................................................................................................................................CUGCACCGCCUCUUCCCUGGC................. 293.504876
.................................................................................................................................................CUGCUCUGCCUCUUCCCUGGC................. 63.189343
.................................................................................................................................................CUGCACUACCUCUUCCCUGGC................. 82.034833
.................................................................................................................................................CUGCACUGCCUCCUCCCUGGC................. 391.546641
.................................................................................................................................................CUGCACUGCCCCUUCCCUGGC................. 395.703637
.................................................................................................................................................CUGCACUGCCUCUUCCCUGUC................. 129.41212
.................................................................................................................................................CCGCACUGCCUCUUCCCUGGC................. 757.331807
.................................................................................................................................................CUGCACUGCCUCUUCCCUGAC................. 686.197061
.................................................................................................................................................CUGCACUGCCUCUUCCCUCGC................. 63.0847
........................AGGGACGAGGCAGAGCAUG............................................................................................................................................ 47.203272
.......................CAGGGACGAGGCAGAGCAU............................................................................................................................................. 307.218386
.......................CAGGGACGAGGCAGAGCAUG............................................................................................................................................ 5820.274748
.......................CAGGGACGAGGCAGAGCAUGGG.......................................................................................................................................... 150.118054
.......................CAGGGACGAGGCAGAGCAUGC........................................................................................................................................... 43.387158
.......................CAGGGACGAGGCAGAGCAUGA........................................................................................................................................... 201.635434
.......................CAGGGAUGAGGCAGAGCAUGG........................................................................................................................................... 482.681549
.......................CAGGGACGAGGCAGAGCAUA............................................................................................................................................ 96.759063
.......................CAGGGACGAGGCAGAGCAUGGC.......................................................................................................................................... 88.281036
.......................CAGGGACGAGGCAGAGCAUGGA.......................................................................................................................................... 118.19076
.......................CAGGGACGAGGCAGAGCAUGGU.......................................................................................................................................... 1266.285031
......................ACAGGGACGAGGCAGAGCAUGG........................................................................................................................................... 1466.148352
......................ACAGGGACGAGGCAGAGCAUG............................................................................................................................................ 379.224584
......................ACAGGGACGAGGCAGAGCAU............................................................................................................................................. 72.354535
......................ACAGGGACGAGGCAGAGCAUGGU.......................................................................................................................................... 72.458759
....................AGACAGGGACGAGGCAGAGCA.............................................................................................................................................. 45.25441
```

zma-MIR528b

Chromosome:	chr9
Start:	156643637
End:	156643750
Strand:	-
Strand bias:	1.0
Abundance bias:	0.991
MFE:	-66.70

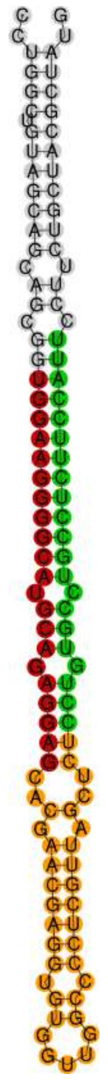

```
                    Mature                                                        Star
CCUGGCUGUAGCAGCAGCGGUGGAAGGGGCAUGCAGAGGAGCACGAACGAGGUGUGGUUGGCCCCUCGUUAGCUCUCCUGUGCCUGCCUCUUCCAUUCCUUCUGCUACGCUAUG   517164.8
..((((.(((((((.((.(((((((((((((.(((.(((((..(.(((((((.((.....)).))))))).)..))))).))).))))))))))))).)).)))))))))))..
....................UGGAAGGGGCAUGCAGAGGAG.........................................................................   444475.58448
............................................................................CCUGUGCCUGCCUCUUCCAUU.................   9703.948492
....................UGGAAGGGGCAUGCAGAGGAGCAC......................................................................   127.876387
....................UGGAAGGGGCAUGCAGAGGA..........................................................................   8891.252005
....................UGGAAGGGGCAUGCAGAGGAGC........................................................................   12283.911698
....................UGGAAGGGGCAUGCAGAG............................................................................   1212.575106
....................UGGAAGGGGCAUGCAGAGGAGCA.......................................................................   529.235887
....................UGGAAGGGGCAUGCAGAGG...........................................................................   6854.795063
....................UGGAAGAGGCAUGCAGAGGAG.........................................................................   95.774232
....................UGGAAGGGGCAUGCAAAGGAG.........................................................................   33.90379
....................UGGAAGGGGCAUGCAGAUGAG.........................................................................   103.030449
....................UGGAAGGGGCCUGCAGAGGAG.........................................................................   94.685734
....................UGGAAGGGGCAUGUAGAGGAG.........................................................................   316.578905
....................UGGAAGGGGUAUGCAGAGGAG.........................................................................   108.214018
....................UGGAAGGGGCAUGCAGCGGAG.........................................................................   276.718031
....................UGGAAGGGGCAUGCGGAGGAG.........................................................................   235.281641
....................UGGAAGGGGCAUGCAGACGAG.........................................................................   30.347512
....................AGGAAGGGGCAUGCAGAGGAG.........................................................................   88.790897
....................UGGAAGGGGCAUGAAGAGGAG.........................................................................   218.347355
....................UGGAAGGUGCAUGCAGAGGAG.........................................................................   123.540633
....................UGGAAGGGGCAUCCAGAGGAG.........................................................................   44.191886
....................UGGAAGGGGCAUGCAGAGGG..........................................................................   41.834357
....................UGGAAGGGGCAUGCAGAGGU..........................................................................   74.299742
....................UGGAAGGGGCAUGCACAGGAG.........................................................................   37.065188
....................UGGAAGGGGCAUGCAGAGGAGU........................................................................   4175.187617
....................UGGAAGGGGCAUGCAGAGGAGA........................................................................   2814.14717
....................UGGAGGGGGCAUGCAGAGGAG.........................................................................   212.713524
....................UGGAAGGGGAAUGCAGAGGAG.........................................................................   194.05132
....................UGGAAGGGGCAUGCAGAGGAU.........................................................................   541.276475
....................UGGAAGGGGCAUGCAGAGGAC.........................................................................   1776.018256
....................UGGAAGGGGCAUGCAGAGGAA.........................................................................   3649.205068
....................UGGAAGGGGCAUGCAGAGUAG.........................................................................   103.027393
....................UGGAAGGGGCAUGCAGAA............................................................................   59.200373
....................UGGAAGGGGCAUGCAGAU............................................................................   239.532116
....................UGGACGGGGCAUGCAGAGGAG.........................................................................   49.892997
....................UGGAAGCGGCAUGCAGAGGAG.........................................................................   85.621982
....................UAGAAGGGGCAUGCAGAGGAG.........................................................................   97.735816
....................UGGAAGGGGCAUGCAGAGGGG.........................................................................   302.607282
....................UGGAAGGGGCACGCAGAGGAG.........................................................................   178.384525
....................UGGAAGGGGCAUGCCGAGGAG.........................................................................   164.847311
....................UGUAAGGGGCAUGCAGAGGAG.........................................................................   198.268742
....................UGGAAGUGGCAUGCAGAGGAG.........................................................................   206.502189
....................UGGAUGGGGCAUGCAGAGGAG.........................................................................   22.445461
....................UGAAAGGGGCAUGCAGAGGAG.........................................................................   83.567613
....................UCGAAGGGGCAUGCAGAGGAG.........................................................................   39.699773
....................UGGAAGGGGCAUACAGAGGAG.........................................................................   94.626718
....................UGGAACGGGCAUGCAGAGGAG.........................................................................   37.370599
....................UGGAAGGGACAUGCAGAGGAG.........................................................................   97.915401
....................UGGAAGGGGCAUUCAGAGGAG.........................................................................   150.013448
....................UGGAAGGGGCAUGCAGAGGUG.........................................................................   70.417763
....................UUGAAGGGGCAUGCAGAGGAG.........................................................................   196.608133
....................GGGAAGGGGCAUGCAGAGGAG.........................................................................   82.289585
....................UGGAAGGGGCAUGCAGAGAAG.........................................................................   34.706149
....................UGGGAGGGGCAUGCAGAGGAG.........................................................................   228.034397
....................UGGAAGGGGCAUGCAGAAGAG.........................................................................   62.361397
....................UGGUAGGGGCAUGCAGAGGAG.........................................................................   24.764429
....................UGGAAGGGGCAUGCAGUGGAG.........................................................................   30.286618
....................UGGCAGGGGCAUGCAGAGGAG.........................................................................   115.438176
....................UGGAAGGGGCAUGCAGAGGCG.........................................................................   205.103984
....................UGGAAGGGGCGUGCAGAGGAG.........................................................................   197.044634
....................UGGAAGGAGCAUGCAGAGGAG.........................................................................   71.337
....................UGGAAGGGGCAUGCAGAGCAG.........................................................................   29.480647
....................UGGAAGGGGCAUGCUGAGGAG.........................................................................   49.738711
....................UGCAAGGGGCAUGCAGAGGAG.........................................................................   68.041754
....................UGGAAGGCGCAUGCAGAGGAG.........................................................................   28.686717
....................UGGAAGGGGCAUGCAUAGGAG.........................................................................   119.789667
....................UGGAAGGGUCAUGCAGAGGAG.........................................................................   128.139705
....................UGGAAGGGGCAUGCAGGGGAG.........................................................................   223.677407
....................CGGAAGGGGCAUGCAGAGGAG.........................................................................   222.062676
....................UGGAAGGGCCAUGCAGAGGAG.........................................................................   54.885136
....................UGGAAUGGGCAUGCAGAGGAG.........................................................................   210.011242
....................UGGAAAGGGCAUGCAGAGGAG.........................................................................   87.348991
....................UGGAAGGGGCAUGCAGAGA...........................................................................   659.94566
....................UGGAAGGGGCAUGCAGAGC...........................................................................   40.93751
....................UGGAAGGGGCAUGCAGAGU...........................................................................   194.109243
.....................GGAAGGGGCAUGCAGAGGAG.........................................................................   2159.707917
.....................GGAAGGGGCAUGCAGAGGAGC........................................................................   529.345046
.....................GGAAGGGGCAUGCAGAGGA..........................................................................   63.700737
.....................GGAAGGGGCAUGCAGAGGAGU........................................................................   78.518991
......................GAAGGGGCAUGCAGAGGAG.........................................................................   39.777886
.......................AAGGGGCAUGCAGAGGAG.........................................................................   154.216753
.........................................................................UCUCCUGUGCCUGCCUCUUCC....................   49.291202
.........................................................................UCUCCUGUGCCUGCCUCUUCCAUU.................   429.669541
..........................................................................CUCCUGUGCCUGCCUCUUCCAUU.................   97.512889
...........................................................................UCCUGUGCCUGCCUCUUCCAU..................   180.56575
...........................................................................UCCUGUGCCUGCCUCUUCC....................   26.3228
...........................................................................UCCUGUGCCUGCCUCUUCCAUU.................   1615.313929
...........................................................................UCCUGUGCCUGCCUCUUCCAUC.................   75.21625
............................................................................CCUGUGCCUGCCUCUUCCAU..................   119.542183
............................................................................CCUGUGCCUGCCUCUUCC....................   102.704852
............................................................................CCUGUGCCUGCCUCUUCCAUA................   46.89992
............................................................................CCUGUGCCUGCCUCUUCCAUC................   329.590347
............................................................................CCUGUGCCUGCCUCUUCU....................   28.887118
............................................................................CUUGUGCCUGCCUCUUCCAUU.................   62.665
............................................................................CCUGUGCCUGCCUCUUCCAUUU................   37.583296
.............................................................................CUGUGCCUGCCUCUUCCAUU.................   158.931815
..............................................................................UGUGCCUGCCUCUUCCAUUCCU..............   64.990294
..............................................................................UGUGCCUGCCUCUUCCAUU.................   242.858163
```

zma-MIR529

Chromosome: chr5
Start: 164370890
End: 164371047
Strand: +
Strand bias: 1.0
Abundance bias: 1.0
MFE: -67.20

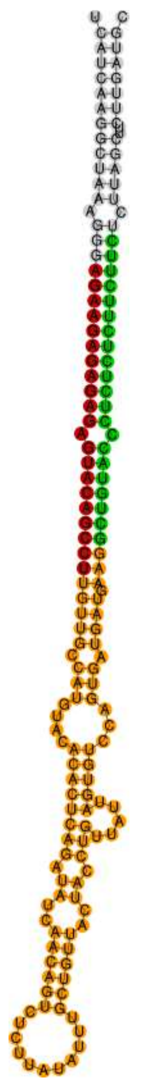

```
                 Mature                                                                                              Star
UCAUCAAGGCUAAAGGGAGAAGAGAGAGAGUACAGCCUUGUUGCCAUGUACACACUCAGAUAUCAACAGUCUCUUAUAUUUGCUGUUACUACCUGUUAUUAGUGUCCAGUGAUGAUGAAGGCUGUACCCUCUCUCUUCUUCUCUUAGCUUCUUGAUGC 25505.4
.((((((((((((.((((((((((((((.((((((((((((((.(((....((((((((.((..((((((...........))))))..)).))).....)))))...))).)))).)))))))))).)))))))))))))).))))).))))))).
.................AGAAGAGAGAGAGUACAGCCU........................................................................................................................ 20833.388454
........................................................................................................................GCUGUACCCUCUCUCUUCUUC................. 824.012429
.................AGAAGAGAGAGAGUACAGCC......................................................................................................................... 468.048952
.................AGAAGAGAGAGAGUACAGC.......................................................................................................................... 213.391896
.................AGAAGAGAGAGAGUACAGCCUU....................................................................................................................... 16.06262
.................AGAAGAGAGAGAGUACAG........................................................................................................................... 7.80415
.................AGAAUAGAGAGAGUACAGCCU........................................................................................................................ 22.9103
.................AGAAGAGAGAGAGGACAGCCU........................................................................................................................ 84.013196
.................AGACGAGAGAGAGUACAGCCU........................................................................................................................ 8.67442
.................AGAAGAGAGAGAGUACAACCU........................................................................................................................ 5.92275
.................AGAAGAGAGAAAGUACAGCCU........................................................................................................................ 2.898403
.................AUAAGAGAGAGAGUACAGCCU........................................................................................................................ 20.98829
.................AGAAGAGAGAGACUACAGCCU........................................................................................................................ 4.93275
.................AGAGGAGAGAGAGUACAGCCU........................................................................................................................ 8.009437
.................AGAAGAGAGAGAGUACACCCU........................................................................................................................ 23.897107
.................AGAAGAGAGUGAGUACAGCCU........................................................................................................................ 2.313741
.................AGAAGAGAGAGAGUACGGCCU........................................................................................................................ 4.63993
.................AGAAGAGAGAGGGUACAGCCU........................................................................................................................ 5.706879
.................UGAAGAGAGAGAGUACAGCCU........................................................................................................................ 20.7489
.................AGAAGCGAGAGAGUACAGCCU........................................................................................................................ 12.327482
.................AGUAGAGAGAGAGUACAGCCU........................................................................................................................ 2.16135
.................AGAAGAGAGAGAGUACAUCCU........................................................................................................................ 47.829771
.................AGAAGAGAGAGAGUACAGCAU........................................................................................................................ 29.100955
.................AGAAGAGAGAGAGAACAGCCU........................................................................................................................ 18.770345
.................AGAAGAGAGGGAGUACAGCCU........................................................................................................................ 11.081164
.................AGAAGAGAGAGAGUACCGCCU........................................................................................................................ 9.22059
.................AGAAGAGAGAGAGUACAGACU........................................................................................................................ 69.352729
.................AGAAGACAGAGAGUACAGCCU........................................................................................................................ 4.71662
.................AGAAGAGAUAGAGUACAGCCU........................................................................................................................ 25.46559
.................AGAAGAGAAAGAGUACAGCCU........................................................................................................................ 3.63594
.................AGAUGAGAGAGAGUACAGCCU........................................................................................................................ 2.16135
.................AGAAGAGAGAGAGUACAGCCG........................................................................................................................ 341.03474
.................AGAAGAGAGAGAGUACAGCCC........................................................................................................................ 871.584725
.................AGAAGAGAGAGAGUACAGCCA........................................................................................................................ 620.522412
.................AGAAGAGAGAGAGUAUAGCCU........................................................................................................................ 25.620001
.................AGAAGAGAGAGAUUACAGCCU........................................................................................................................ 18.892383
.................AGAAGAGACAGAGUACAGCCU........................................................................................................................ 5.18723
.................AGAAGAGAGAGAGUACAGCUU........................................................................................................................ 21.966839
.................AGAAGAUAGAGAGUACAGCCU........................................................................................................................ 21.170834
.................AGAAGAGAGAGCGUACAGCCU........................................................................................................................ 5.135938
.................AGAAGAGGGAGAGUACAGCCU........................................................................................................................ 16.634764
.................AGAAGAGAGAGAGUACAGU.......................................................................................................................... 8.39512
.................GGAAGAGAGAGAGUACAGCCU........................................................................................................................ 15.788503
.................AAAAGAGAGAGAGUACAGCCU........................................................................................................................ 3.661338
.................AGAAGAGAGAGAGUCCAGCCU........................................................................................................................ 5.606558
.................AGAAGAGAGAUAGUACAGCCU........................................................................................................................ 8.21312
.................AGAAGGGAGAGAGUACAGCCU........................................................................................................................ 10.577688
.................AGAAGAGAGAGAGUACAGCGU........................................................................................................................ 4.32269
.................AGAACAGAGAGAGUACAGCCU........................................................................................................................ 4.10656
.................AGAAGAGAGAGAGUAGAGCCU........................................................................................................................ 4.983547
.................AGAAGAGAGAGAGUACAGCA......................................................................................................................... 55.35908
.................AGAAGAGAGAGAGUACAGCU......................................................................................................................... 114.77182
.................AGAAGAGAGACAGUACAGCCU........................................................................................................................ 5.250484
.................AGAAGAGAGAGAAUACAGCCU........................................................................................................................ 4.512937
.................CGAAGAGAGAGAGUACAGCCU........................................................................................................................ 33.220991
.................AGGAGAGAGAGAGUACAGCCU........................................................................................................................ 10.807804
.................AGAAGAGAGAGAGUAAAGCCU........................................................................................................................ 30.44317
.................ACAAGAGAGAGAGUACAGCCU........................................................................................................................ 6.331154
.................AGAAGUGAGAGAGUACAGCCU........................................................................................................................ 8.5997
.................AGAAGAGAGAGAGUACAGGCU........................................................................................................................ 14.73228
.................AGAAGAGAGAGAGUGCAGCCU........................................................................................................................ 3.686737
.................AGAAGAGAGAGUGUACAGCCU........................................................................................................................ 4.576676
.................AGAAGAGAGCGAGUACAGCCU........................................................................................................................ 6.36901
.................AGAAGAGAGAGAGCACAGCCU........................................................................................................................ 45.968148
.................AGAAAAGAGAGAGUACAGCCU........................................................................................................................ 4.475081
.................AGAAGAGUGAGAGUACAGCCU........................................................................................................................ 5.20076
.................AGAAGAGAGAGAGUUCAGCCU........................................................................................................................ 4.907351
.................AGCAGAGAGAGAGUACAGCCU........................................................................................................................ 3.496006
.................AGAAGAGAGAGAGUACUGCCU........................................................................................................................ 5.339127
.................AGAAGAGCGAGAGUACAGCCU........................................................................................................................ 11.349001
.................AGAAGAGAGAGAGUACAGUCU........................................................................................................................ 16.654787
.................AGAAGAAAGAGAGUACAGCCU........................................................................................................................ 2.376985
.................AGAAGAGAGAGAGUACAGCCUC....................................................................................................................... 1.51294
.................AGAAGAGAGAGAGUACAGCCUA....................................................................................................................... 2.37748
.................AGAAGAGAGAGAGUACAGCCUG....................................................................................................................... 2.53144
..................GAAGAGAGAGAGUACAGCC......................................................................................................................... 8.21164
..................GAAGAGAGAGAGUACAGC.......................................................................................................................... 4.10656
..................GAAGAGAGAGAGUACAGCCUU....................................................................................................................... 1.94521
..................GAAGAGAGAGAGUACAGCCU........................................................................................................................ 119.812087
..................GAAGAGAGAGAGGACAGCCU........................................................................................................................ 0.17779
...................AAGAGAGAGAGUACAGCC......................................................................................................................... 2.37748
...................AAGAGAGAGAGUACAGCCU........................................................................................................................ 5.157144
...................AAGAGAGAGAGUACAGCCUUG...................................................................................................................... 1.94521
....................AGAGAGAGAGUACAGCCU........................................................................................................................ 19.896887
.....................................................................................................................AAGGCUGUACCCUCUCUCUUC.................... 16.25061
.......................................................................................................................GGCUGUACCCUCUCUCUUCUU.................. 0.304783
........................................................................................................................GCUGUACCCUCUCUCUUCUU.................. 67.199166
........................................................................................................................GCUGUACCCUCUCUCUUCU................... 4.512572
........................................................................................................................GCUGUACCCUCUCUCUUC.................... 7.68888
........................................................................................................................GCUGUACCCUCUCUCUUCUUA................. 0.253986
........................................................................................................................GCUGUACCCUCUCUCUUCUUU................. 11.422236
........................................................................................................................GCUGUACUCUCUCUCUUCUUC................. 1.72908
```

zma-MIR827

Chromosome: chr5
Start: 171268562
End: 171268781
Strand: -
Strand bias: 1.0
Abundance bias:1.0
MFE: -93.90

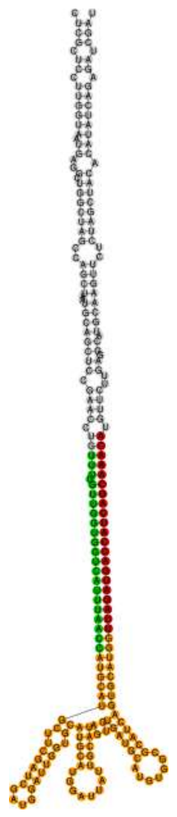

```
                                                    Star                                                                                Mature
CUCGCUCCUUGGUAAUGAGGCUGGCUAGCCAGCUAAUGCAGCUCCGAACCUGUUUUGUUGGUGGUCAUUUAACCAUGCAUGCUUCGAUCGAUGGAUUGGUGCAUGCAUGGAUUAUUGCAUAGUGUGAUGCAUGUGGCGCAUCAGUGCAUGGUUAGAUGACCAUCAGCAAACAUGUUCUUGAGGCAUGCAAGUUCUCUAGCUACACAUAUCAGAGAUCGAU    302282.2
.(((.((.((((((.((..(.(((((((..((((..((((((((.((((.(((((.((((((((((((((((((((((((((.((((((....)))))).))(((((........)))))....((((((.......))))))))))))))))))))))))))))))))))).))))..)).)).))))))))..)))))))).)))))))).)).))).
.......................................................................................................................................................UUAGAUGACCAUCAGCAAACA................................................    252542.0055
....................................................UUUUGUUGGUGGUCAUUUAACC..................................................................................................................................................    30955.94196
..........................................................................................................................................................GAUGACCAUCAGCAAACA................................................    180.41215
.........................................................................................................................................................AGAUGACCAUCAGCAAACA................................................    218.295115
........................................................................................................................................................UAGAUGACCAUCAGCAAACA................................................    2264.615566
.......................................................................................................................................................UUAGAUGACCAUCAGCAAAC.................................................    2368.981629
.......................................................................................................................................................UUAGAUGACCAUCAGCAAACAU...............................................    228.939371
.......................................................................................................................................................UUAGAUGACCAUCAGCAAA..................................................    205.86654
.......................................................................................................................................................UUAGAUGACCAUCAGCAA...................................................    143.78625
.......................................................................................................................................................UUAGAUGACCAUCAGCGAACA................................................    94.548445
.......................................................................................................................................................UUAGAUUACCAUCAGCAAACA................................................    164.720445
.......................................................................................................................................................UUAGAUGACCGUCAGCAAACA................................................    73.643284
.......................................................................................................................................................UUAGAUGACCAUCAGCAAGCA................................................    89.741063
.......................................................................................................................................................CUAGAUGACCAUCAGCAAACA................................................    134.820655
.......................................................................................................................................................UUAGAUGACCAUCAGCAAAU.................................................    133.944605
.......................................................................................................................................................UUAGAUGACCAUCAGCAAACUU...............................................    63.01406
.......................................................................................................................................................UUAGAUGACCAUCAGCAAAUA................................................    44.841383
.......................................................................................................................................................UUAGAUGACCAUCAGCAAACAG...............................................    212.461248
.......................................................................................................................................................UUAGAUGACCAUCAGCAAACAC...............................................    207.187202
.......................................................................................................................................................UUAGAUGACCAUCAGCAAACAA...............................................    649.367835
.......................................................................................................................................................UUAGAUGACCAUCAGCAAACU................................................    727.270007
.......................................................................................................................................................UUAGAUGACCAUCAGCAAACG................................................    540.552308
.......................................................................................................................................................UUAGAUGACCAUCAGCAAACC................................................    82.17429
.......................................................................................................................................................UUAGAUAACCAUCAGCAAACA................................................    44.135224
.......................................................................................................................................................UUAGACGACCAUCAGCAAACA................................................    91.576636
.......................................................................................................................................................UUAGAUGACCAUCAUCAAACA................................................    85.381642
.......................................................................................................................................................UUAUAUGACCAUCAGCAAACA................................................    40.816887
.......................................................................................................................................................UUAGAUGACCAUCGGCAAACA................................................    106.22658
.......................................................................................................................................................UUAGAUGACCAUUAGCAAACA................................................    75.750855
.......................................................................................................................................................UUAGAUGACCAUCAACAAACA................................................    54.482677
.......................................................................................................................................................UUGGAUGACCAUCAGCAAACA................................................    79.949718
.......................................................................................................................................................UCAGAUGACCAUCAGCAAACA................................................    88.875527
.......................................................................................................................................................UGAGAUGACCAUCAGCAAACA................................................    37.03316
.......................................................................................................................................................UUAGGUGACCAUCAGCAAACA................................................    107.292094
.......................................................................................................................................................UUAGAUCACCAUCAGCAAACA................................................    58.499126
.......................................................................................................................................................GUAGAUGACCAUCAGCAAACA................................................    127.313147
.......................................................................................................................................................UUAGAUGACCACCAGCAAACA................................................    64.762995
.......................................................................................................................................................UUAGAUGACUAUCAGCAAACA................................................    94.737378
.......................................................................................................................................................UUAGAUGACCAUCAGCAACCA................................................    268.262674
.......................................................................................................................................................UUAGAUGACCAUCAGCAGACA................................................    77.053376
.......................................................................................................................................................UUAGAUGAUCAUCAGCAAACA................................................    123.303028
.......................................................................................................................................................UUAGAUGGCCAUCAGCAAACA................................................    135.477945
.......................................................................................................................................................UUAGAUGACCAUCAGCAAAAA................................................    48.00729
......................................................................................................................................................GUUAGAUGACCAUCAGCAAACA................................................    52.264737
......................................................................................................................................................GUUAGAUGACCAUCAGCAAAC.................................................    1006.361235
......................................................UUGUUGGUGGUCAUUUAACC..................................................................................................................................................    61.365583
.....................................................UUUGUUGGUGGUCAUUUAACCA.................................................................................................................................................    2095.422565
.....................................................UUUGUUGGUGGUCAUUUAACC..................................................................................................................................................    1965.244939
....................................................UUUUGUUGGUGGUCAUUUAAC...................................................................................................................................................    351.221623
....................................................UUUUGUUGGUGGUCAUUUAA....................................................................................................................................................    47.212869
....................................................UUUUGUUGGUGGUCAUUUAACCA.................................................................................................................................................    238.867607
....................................................CUUUGUUGGUGGUCAUUUAACC..................................................................................................................................................    41.218521
....................................................UUUUGUUGGUUGUCAUUUAACC..................................................................................................................................................    57.608499
....................................................UUUUGUUGGUGGUCAUUUAACA..................................................................................................................................................    227.769233
....................................................UUUUGUUGGUGGUCAUUUAACU..................................................................................................................................................    765.363776
....................................................UUUUGUUGUUGGUCAUUUAACC..................................................................................................................................................    64.288342
....................................................UUUUGUUGGUGGUCAUUUAAAC..................................................................................................................................................    52.80973
....................................................UUUUGUUGGUGGUCCUUUAACC..................................................................................................................................................    45.26313
```

```
                  Star                                                          Mature
AGUUGGGUAUGCUAGAGUUGAAACCCAGCAAUCGAACAUAUAUUUUGUUGUAUAUCUGGUAAUACUUCAGAAUAUAUGUUCAAUUGUUGGGUUUCAACUUAUCCUGCUAGCCAACA    210.8
.(((((.((.((..(((((((((((((((((.((((((((((((((..((((........))))..)))))))))))))).))))))))))))))))).....)))).))))).
..............................................................................UUCAAUUGUUGGGUUUCAACU.................    124.677054
.................UUGAAACCCAGCAAUCGAACA..............................................................................    84.086107
...............................................................................UCAAUUGUUGGGUUUCAACUU................    1.900747
.................UUGAAACCCAGCAAUCGAAC...............................................................................    0.228587
```

zma-MIR_N195a

```
Chromosome:    chr9
Start:         139046314
End:           139046429
Strand:        -
Strand bias:   1.0
Abundance bias:1.0
MFE:           -58.70
```

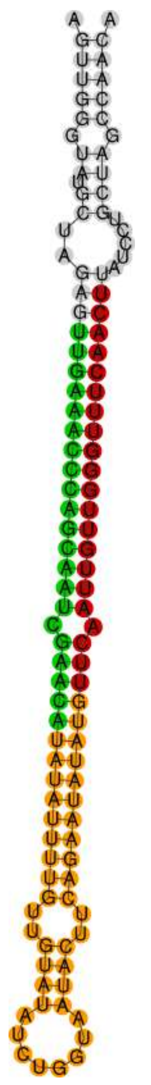

Mature Star

```
UAUGCACAAGUAGGGCCUGUGAGAUAGACAAAUUUGUUCAUAUUCUAGUUAUAUAGGACAUGUGAGAAUCAUUUAAUGUAUCGGACGAAUUGGUUCUGACAGGCCCUAAACACAUUGUAAGUGGUGAACUAGAACAUGAACAAAUUUGUCUACCUCACAGGUCCUAAUUGCACAUAU    43.6
((((..(((.(((((((((((((.(((((((((((((((((.((((((((...((((.(.(((.(((((((.((..(((.....))))).))))))).))).).))))..(((.......)))...)))))))).))))))))))))))))).))))))))))))).)))..)))).
.................UGUGAGAUAGACAAAUUUGUU..............................................................................................................................................    29.63035
................................................................................................................................................CAAAUUUGUCUACCUCACAGG................    3.885276
................CUGUGAGAUAGACAAAUUUGU...............................................................................................................................................    0.203188
```

zma-MIR_N48a

Chromosome: chr1
Start: 102722309
End: 102722485
Strand: +
Strand bias: 1.0
Abundance bias: 0.772
MFE: -76.50

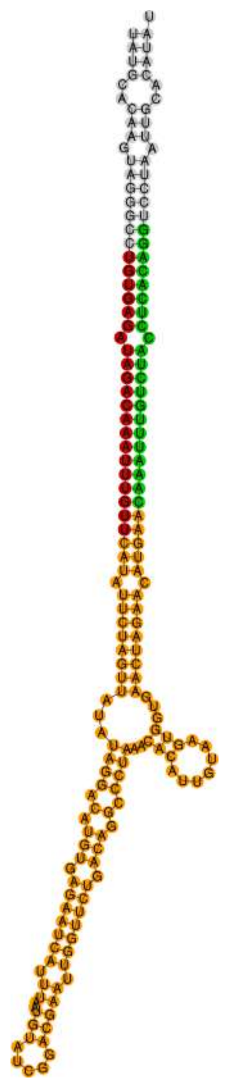

```
                                Mature                                                                                          Star
AGAAUACGACCUGGUUAGCUGACGUGGUCUUGCCACCGUGUCGUCCACGUCGCACGACAUGAUUGGAGCAGGACGCGGCAAGGACCAGGCGGCGGCCAUGUGGCAAAGCCGUGCCACGUGGACAACACGGAGGCAAGGCCACGUCAGCUACCCAAGUCUUGUUC    566.6
.(((((.(((.(((.(((((((((((((((((((.((((((.((((((((.(((((......(((..(((((.(((.((.........)).))).)).)))..)))...))))).)))))))).)))))).))))))))))))))))))).))).))).)))))
........................UGGUCUUGCCACCGUGUCGUCC......................................................................................................................    267.484633
.........................................................................................................................ACAACACGGAGGCAAGGCCACG.....................    19.864578
........................UGGUCUUGCCACCGUGUCGUC.......................................................................................................................    9.825788
........................UGGUCUUGCCACCGUGUCGUCA.....................................................................................................................    1.44062
........................UGGUCUUGCCACCGUGUCGUCU.....................................................................................................................    5.52599
```

zma-MIR_N196a

```
Chromosome:    chr9
Start:         145888822
End:           145888985
Strand:        -
Strand bias:   1.0
Abundance bias:0.955
MFE:           -98.50
```

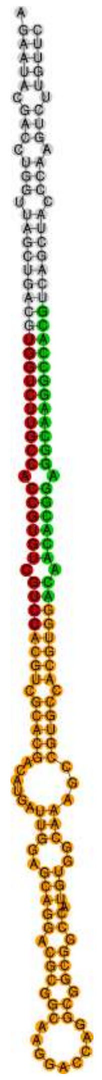

zma-MIR2275_N2

```
Chromosome:    chr1
Start:         237845499
End:           237845747
Strand:        +
Strand bias:   1.0
Abundance bias:0.785
MFE:           -103.50
```

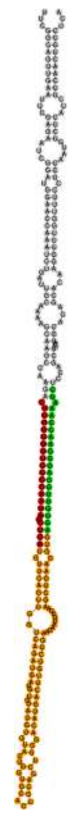

```
                                                                 Mature                                                                          Star
UUCGGCAGUUGAAUUCGAGAAUCGGAUGGAACCAAAUCUGAUUGCAAAAGCAAGCCAAGACUUGGUUUUCUCCAAUAUCUCAUGUUCAGUUGGUCAGAUGAUGUGUAUGUGUACUGGUGCACUGCAGCUUGGACAUGACUUGUCUGAAGAAUUUGAAGCUGAAGGUGAGUGUUGGAGGGAACCAAAUCUCCAGCUGCAUGCACAGCACAAGGUUUGGUUUCCUCCAAUGUCUCAUCUUCAACUGUCUCA   1556.8
...((((((((((...((((...(((.((((((((((((...(((....((((((..(((.((((((((((((((((.(((((.(((((((..((((((((.((.(((((.((..(.(((...))).).)).))))))))))))))).........))))))).))))))))))))))))))))).)))...)))...)))...)))..)))))))))))).)))....))))...))))))))))...
............................................................CUUGGUUUUCUCCAAUAUCUCA.......................................................................................................................................................................   0.304783
.......................................................................................................................................................................AGUGUUGGAGGGAACCAAAUC.............................................................   1153.497514
.....................................................................................................................................................................UGAGUGUUGGAGGGAACCAAA...............................................................   0.584167
.....................................................................................................................................................................UGAGUGUUGGAGGGAACCAAAUCU............................................................   0.380978
.......................................................................................................................................................................AGUGUUGGAGGGAACCAAAUCUCC..........................................................   0.939746
.......................................................................................................................................................................AGUGUUGGAGGGAACCAAA...............................................................   0.406377
.......................................................................................................................................................................AGUGUUGGAGGGAACCAAAUCU............................................................   20.16479
.......................................................................................................................................................................AGUGUUGGAGGGAACCAAAU..............................................................   22.81517
.......................................................................................................................................................................AGUGUUGGAGGGAACCGAAUC.............................................................   0.279384
.......................................................................................................................................................................CGUGUUGGAGGGAACCAAAUC.............................................................   0.152391
.......................................................................................................................................................................AGUGCUGGAGGGAACCAAAUC.............................................................   0.482573
.......................................................................................................................................................................AGUGUGGGAGGGAACCAAAUC.............................................................   0.253986
.......................................................................................................................................................................AGUGUUGGGGGGAACCAAAUC.............................................................   0.330181
.......................................................................................................................................................................AGUGUUGGAGUGAACCAAAUC.............................................................   0.203188
.......................................................................................................................................................................AGUGUUGGAGGGAACCCAAUC.............................................................   0.431775
.......................................................................................................................................................................AGUGUCGGAGGGAACCAAAUC.............................................................   0.17779
.......................................................................................................................................................................AGUGUUGGAGGGAACCAAAUCA............................................................   0.35558
.......................................................................................................................................................................AGUGUUGGAGGGAACCAAAUCC............................................................   0.634964
.......................................................................................................................................................................AGUGUUGGAGGGAUCCAAAUC.............................................................   0.17779
.......................................................................................................................................................................AGUGUUUGAGGGAACCAAAUC.............................................................   0.228587
.......................................................................................................................................................................AGUGUUGGAGGGAAACAAAUC.............................................................   0.330181
.......................................................................................................................................................................AGUGUUGGAGGGAACCAAAUA.............................................................   0.990544
.......................................................................................................................................................................AGUGUUGGAGGGAACCAAAUG.............................................................   0.35558
.......................................................................................................................................................................AGUGUUGGAGGGAACCAAAUU.............................................................   3.724738
.......................................................................................................................................................................AGUGGUGGAGGGAACCAAAUC.............................................................   0.761957
.......................................................................................................................................................................AGUGUUGGAGGCAACCAAAUC.............................................................   0.228587
.......................................................................................................................................................................AGCGUUGGAGGGAACCAAAUC.............................................................   0.888949
.......................................................................................................................................................................AGUGUUGGAGGGAACUAAAUC.............................................................   0.228587
.......................................................................................................................................................................AGUGUUGAAGGGAACCAAAUC.............................................................   0.228587
.......................................................................................................................................................................AGUGUUGGAGGGAGCCAAAUC.............................................................   0.17779
.......................................................................................................................................................................AGUGUUGGAGGGAACCACAUC.............................................................   0.152391
.......................................................................................................................................................................AAUGUUGGAGGGAACCAAAUC.............................................................   0.152391
.......................................................................................................................................................................AGGGUUGGAGGGAACCAAAUC.............................................................   5.2603
.......................................................................................................................................................................AGUGUUGGAGGGAACCAAAA..............................................................   0.17779
.......................................................................................................................................................................AGUGUUAGAGGGAACCAAAUC.............................................................   0.203188
.......................................................................................................................................................................AGUGUUGGAGGGGACCAAAUC.............................................................   0.406377
.......................................................................................................................................................................AGUGUUGGAGGGAACAAAAUC.............................................................   0.431775
.......................................................................................................................................................................AGUGUUGGAGGGAACCAAUU..............................................................   0.152391
.......................................................................................................................................................................AGUGUUGGAGGGAACCAAACC.............................................................   0.228587
.......................................................................................................................................................................AGUGUUGGAGGGUACCAAAUC.............................................................   0.253986
.......................................................................................................................................................................AGUGUUGGAGGAAACCAAAUC.............................................................   0.380978
.......................................................................................................................................................................AGUGUUGGAGGGAAUCAAAUC.............................................................   0.228587
.......................................................................................................................................................................AGAGUUGGAGGGAACCAAAUC.............................................................   0.330181
.......................................................................................................................................................................AGUGUUGGAGGGCACCAAAUC.............................................................   0.253986
.......................................................................................................................................................................AGUGUUGGAGGGAACCUAAUC.............................................................   0.152391
........................................................................................................................................................................GUGUUGGAGGGAACCAAAUC.............................................................   1.78955
........................................................................................................................................................................GUGUUGGAGGGAACCAAAUCU............................................................   0.253986
........................................................................................................................................................................GUGUUGGAGGGAACCAAAUCUCC..........................................................   0.152391
```

zma-MIR_N51a

Chromosome: chr1
Start: 180011505
End: 180011638
Strand: -
Strand bias: 0.986
Abundance bias:0.821
MFE: -34.40

```
                  Mature                                                                       Star
AUUUAAGGCAGAGGCGAAUCAAGACUAGGUGGGCAAGCUGCAAACUCGAUUAUCAAGCAACUGAGAGACUGGGUGGUAUAUUUUAGAGAAACAUGCUAUUCAUCUACUUCUUGGUGAAGCCGUUGACCGGAGAA    325.1
.(((..(((((.(((..(((((((.((((((((..(((......(((.........((.(((.((...)).))).)).........)))......))).))))))))..)))))))...))).))).))..))).
..................UCAAGACUAGGUGGGCAAGCU...............................................................................................    176.99865
...............................................................................................CUAUUCAUCUACUUCUUGGUG..................    0.152391
..................UCAAGACUAGGUGGGCAAGCUGCA...........................................................................................    1.119402
..................UCAAGACUAGGUGGGCAAGCUGC............................................................................................    0.427815
..................UCAAGACUAGGUGGGCAAGC................................................................................................    5.622578
..................UCAAGACUAGGGGGGCAAGCU...............................................................................................    2.69598
..................UCAAGACUAGGUGGGCAAGCC...............................................................................................    4.21907
..................UCAAGACUAGGUGGGCAAGCG...............................................................................................    0.855631
```

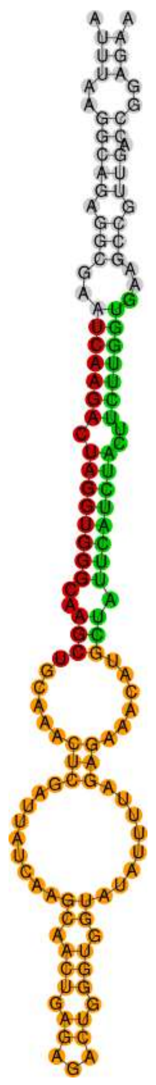

```
                   Star                                                              Mature
UUGGAUACUCUAGUAUUCCCCCUAAUUCACAUGUGUUGAGAUAUAUUGGGGUGUAAAUUAGUUUAAUUUAUACUCUAAUAAACCUCAACACACGUGGAUUGCGGUGAAUACUAGAGUAUCCA    169.1
.(((((((((((((((((.((.((((((((.(((((((((...(((((((((((((((((...)))))))))))))))))...))))))))).)))))))).)).)))))))))))))))))
....................................................................................UCAACACACGUGGAUUGCGGU.................    152.244811
....................CCUAAUUCACAUGUGUUGAGA.................................................................................    2.22954
......................................................................................AACACACGUGGAUUGCGGUGAA..............    6.068185
....................................................................................UCAACACACGUGGAUUGCGGUA................    1.27549
....................................................................................UCAACACACGUGGAUUGCGGUU................    1.769312
```

zma-MIR_N54a

```
Chromosome:    chr1
Start:         243210368
End:           243210489
Strand:        -
Strand bias:   1.0
Abundance bias:0.967
MFE:           -68.90
```

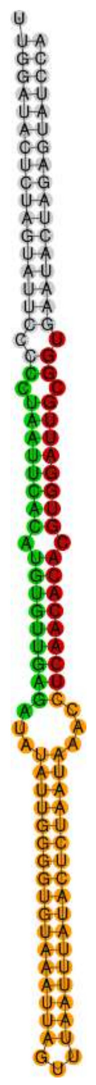

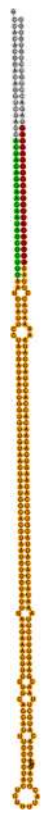

zma-MIR_N59a

```
Chromosome:     chr1
Start:          302661735
End:            302661992
Strand:         -
Strand bias:    1.0
Abundance bias:0.847
MFE:            -183.70
```

```
                         Star                                                                                                                                                                                                     Mature
GCCACACGGACAGGUCUACCACUACAGGAACAAUAUCUCUUUAAUGUAUUCCUAUGAUAAGUGGUGUUGUAACUUAUGGCACGUAAUGUGGUAUUGAAGAAACUUCCUUCAGCAUUUCCUCUACUCUUUUACGUAGGUCAGGACAUGCUUAACGAAGUUUCUUAAAUACCACAUUACGUGUCAUAAGUUACAACACCACUUAUCAUAAAAAUACCUUAAAGAGAUAUUGUUCCUGUAGUGGUAGACCUGUCUGUGUGG    4141.6
.(((((((((((((((((((((((((((((((((((((((((((.(((((..(((((((((((((((((((((((((((((((((((((((((((.((((((((((.((.(((((.((((((((........))))...)))).))))).)).)))))))))).))))))))))))))))))))))))))))))))))))))))))))..))))).)))))))))))))))))))))))))))))))))))))))))))
...........................................................................................................................................................................................................................AGAGAUAUUGUUCCUGUAGUGG.................    729.299703
....................ACUACAGGAACAAUAUCUCUUU........................................................................................................................................................................................................................    2.9854
..............................................................................................................................................................................................................................GAUAUUGUUCCUGUAGUGGUA...............    251.333155
..............................................................................................................................................................................................................................GAUAUUGUUCCUGUAGUGGUAG..............    60.14835
..............................................................................................................................................................................................................................GAUAUUGUUCCUGUAGUGGU................    4.6879
.............................................................................................................................................................................................................................AGAUAUUGUUCCUGUAGUGGUAG..............    3.15946
.............................................................................................................................................................................................................................AGAUAUUGUUCCUGUAGUGGUA...............    146.806764
.............................................................................................................................................................................................................................AGAUAUUGUUCCUGUAGUGGU................    312.770015
.............................................................................................................................................................................................................................AGAUAUUGUUCCUGUAGUGGC................    17.99628
............................................................................................................................................................................................................................GAGAUAUUGUUCCUGUAGUGG.................    24.425839
............................................................................................................................................................................................................................GAGAUAUUGUUCCUGUAGUGGUAG..............    81.084358
............................................................................................................................................................................................................................GAGAUAUUGUUCCUGUAGUGGUA...............    76.312404
............................................................................................................................................................................................................................GAGAUAUUGUUCCUGUAGUGGU................    466.626202
............................................................................................................................................................................................................................GAGAUAUUGUUCCUGUAGUGGC................    21.687943
...........................................................................................................................................................................................................................AGAGAUAUUGUUCCUGUAG....................    1.221129
...........................................................................................................................................................................................................................AGAGAUAUUGUUCCUGUAGUG..................    151.07376
...........................................................................................................................................................................................................................AGAGAUAUUGUUCCUGUAGU...................    45.077718
...........................................................................................................................................................................................................................AGAGAUAUUGUUCCUGUAGUGGU................    0.152391
...........................................................................................................................................................................................................................AGAGAUAUUGUUCCUGUAGUGGUA...............    457.829581
...........................................................................................................................................................................................................................AGAGAUAUUGUUCCUGUAGUA..................    1.28532
...........................................................................................................................................................................................................................AGAGAUAUUGUUCCUGUAGUGA.................    49.28452
...........................................................................................................................................................................................................................AGAGAUAUUGUUCCUGUAGUGU.................    3.559002
..........................................................................................................................................................................................................................AAGAGAUAUUGUUCCUGUAGUGGU................    130.072156
..........................................................................................................................................................................................................................AAGAGAUAUUGUUCCUGUAGUGG.................    2.987029
..........................................................................................................................................................................................................................AAGAGAUAUUGUUCCUGUAGUGGUA...............    1.11754
..........................................................................................................................................................................................................................AAGAGAUAUUGUUCCUGUAGUG..................    211.972886
..........................................................................................................................................................................................................................AAGAGAUAUUGUUCCUGUAGU...................    11.175764
..........................................................................................................................................................................................................................AAGAGAUAUUGUUCCUGUAGUGGC................    4.991998
..........................................................................................................................................................................................................................AAGAGAUAUUGUUCCUGUAGUGGG................    0.17779
..........................................................................................................................................................................................................................AAGAGAUAUUGUUCCUGUAGUGA.................    0.279384
..........................................................................................................................................................................................................................AAGAGAUAUUGUUCCUGUAGUGGA................    2.59065
..........................................................................................................................................................................................................................AAGAGAUAUUGUUCCUGUAGUA..................    1.10171
.........................................................................................................................................................................................................................AAAGAGAUAUUGUUCCUGUAG....................    0.228587
.........................................................................................................................................................................................................................AAAGAGAUAUUGUUCCUGUAGUGG.................    124.478205
.........................................................................................................................................................................................................................AAAGAGAUAUUGUUCCUGUAGU...................    14.801094
.........................................................................................................................................................................................................................AAAGAGAUAUUGUUCCUGUAGUGGA................    0.253986
.........................................................................................................................................................................................................................AAAGAGAUAUUGUUCCUGUAGUGA.................    5.083829
........................................................................................................................................................................................................................UAAAGAGAUAUUGUUCCUGUAGU...................    7.72157
........................................................................................................................................................................................................................UAAAGAGAUAUUGUUCCUGUAGUG..................    21.214615
......................UACAGGAACAAUAUCUCUUUA.......................................................................................................................................................................................................................    8.095781
.....................CUACAGGAACAAUAUCUCUUUA.......................................................................................................................................................................................................................    8.829209
.....................CUACAGGAACAAUAUCUCUUU........................................................................................................................................................................................................................    14.224363
....................ACUACAGGAACAAUAUCUCUUUAA......................................................................................................................................................................................................................    1.08032
...................CACUACAGGAACAAUAUCUCUU.........................................................................................................................................................................................................................    23.096967
..................CCACUACAGGAACAAUAUCUC...........................................................................................................................................................................................................................    2.356332
```

zma-MIR_N65a

```
Chromosome:    chr2
Start:         70460061
End:           70460180
Strand:        +
Strand bias:   1.0
Abundance bias:1.0
MFE:           -71.20
```

```
                 Mature                                                            Star
AGUUUGGAAGGCUAAAACCCGGAGAGGAUUGAAGGGUUAAAAUCCCUUUCUUAUUCAAUUUUGAAUAAGGAGGGGAUUUUAGCCCCUCUAUCCCCUUCCGGGUUUAAGGUUCCCAAACUA    221.1
(((((((.((.((.((((((((((.((((.((.((((((((((((((((((((((((....)))))))))))))))))))))))).)).)))).).))))))))).)).)).))))))).
.................CCCGGAGAGGAUUGAAGGGUU..................................................................................    146.345276
..................................................................................CCCCUCUAUCCCCUUCCGGGUU................    21.754702
.................CCCGGAGAGGAUUGAAGGGUUA.................................................................................    0.182396
.................CCCGGAGAGGAUUGAAGGGU...................................................................................    37.578723
..................CCGGAGAGGAUUGAAGGGUUA.................................................................................    4.168404
..................CCGGAGAGGAUUGAAGGGUU..................................................................................    2.854539
...................CGGAGAGGAUUGAAGGGUUAA................................................................................    7.828307
....................GGAGAGGAUUGAAGGGUUAAA...............................................................................    0.43814
```

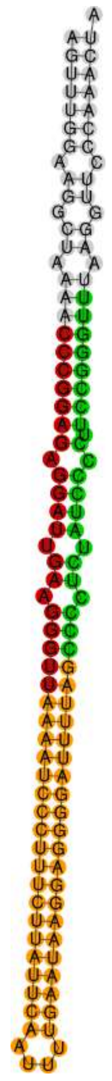

zma-MIR_N66a

Chromosome: chr2
Start: 92156503
End: 92156639
Strand: +
Strand bias: 1.0
Abundance bias:0.906
MFE: -56.00

```
                 Mature                                                                     Star
AGAACAAUAGCAUUCUUAUGCAGAACAAUUUACAGACGGAAAUGAUUUGUACUAUUUCUCAGGUGCAAACCAACCAUUUCUCAGGUGCAAACCAUUUCGAUCUGUAAGUUGUUGUGCACAAGAAUACUACUGUUUUA   1413.4
((((((.(((.((((((.((((.(((((((((((((..((((((.((((((((........(((........)))........)))))))).))))))..))))))))))))).)))).)))))).))).)))))).
.................AUGCAGAACAAUUUACAGACG...................................................................................................   910.30856
....................................................................................................UCUGUAAGUUGUUGUGCACAA................   64.942148
...............UUAUGCAGAACAAUUUACAGA.....................................................................................................   2.974932
...............UUAUGCAGAACAAUUUACAGACG...................................................................................................   1.54332
...............UUAUGCAGAACAAUUUACAGACGG..................................................................................................   22.83989
................UAUGCAGAACAAUUUACAGACGGA.................................................................................................   1.42512
................UAUGCAGAACAAUUUACAGACGG..................................................................................................   16.35346
................UAUGCAGAACAAUUUACAGACG...................................................................................................   1.44922
.................AUGCAGAACAAUUUACAGACGG..................................................................................................   6.42602
.................AUGCAGAACAAUUUACAGA.....................................................................................................   4.61838
.................AUGCAGAACAAUUUACAGAC....................................................................................................   9.31235
.................AUGCAGAACAAUUUACAGACGA..................................................................................................   0.956615
.................AUGCAGAACAAUUUACAGACGU..................................................................................................   19.06756
.................AUGCAGAACAAUUUACAGACA...................................................................................................   46.57129
..................UGCAGAACAAUUUACAGACGG..................................................................................................   147.944247
...................................................................................................AUCUGUAAGUUGUUGUGCACAAGA..............   2.947832
....................................................................................................UCUGUAAGUUGUUGUGCAC..................   2.16082
....................................................................................................UCUGUAAGUUGUUGUGCACA.................   19.1958
```

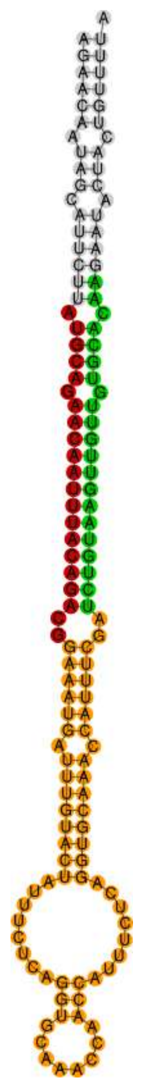

zma-MIR_N16b

Chromosome: chr1
Start: 273081714
End: 273081832
Strand: -
Strand bias: 1.0
Abundance bias: 0.997
MFE: -48.70

Star Mature

```
GAAGGUUAGGAGAGUAAAAUAUCAUUUUGGAACCACAAAUAAGCUAAAAUAAGCUACCCAAUGUUAACUUAUUUCAGUUUAUUUGUGGUCUCAAAGUGAUAUUUUACCCUCAUACACUU     543.4
.(((..((.(((.(((((((((((((((((.((((((((((((((.(((((((.((........)).))))))).)))))))))))))).))))))))))))))))).))).))..)))
.................................................................................UUUGUGGUCUCAAAGUGAUAU.................     458.370216
....................AUCAUUUUGGAACCACAAAUA..............................................................................     3.272475
.................................................................................UUUGUGGUCUCAAAGUGAUA..................     8.901715
.................................................................................UUUGUGGUCUCAAAGUGAUAUU................     32.422154
.................................................................................UUUGUGGUCUCAAAGUGAUAUG................     1.45641
................................................................................AUUUGUGGUCUCAAAGUGAUA..................     19.834531
...............................................................................UAUUUGUGGUCUCAAAGUGAU...................     17.603827
```

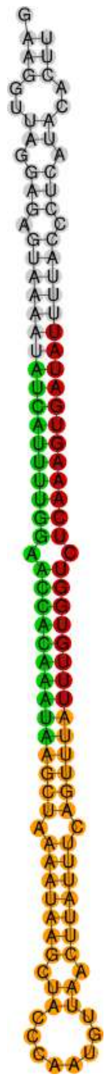

zma-MIR_N82a

Chromosome: chr2
Start: 231204074
End: 231204211
Strand: +
Strand bias: 1.0
Abundance bias: 1.0
MFE: -79.80

```
                         Mature                                                                         Star
UGGCAUGGCUCUCUUGGUCCCUCUCUCCCUUGAAGGCUCUCUCUGGUAAAUAUGGUUGCCUUUCACAAUGAACUUCCAUCAGAUCUUUGUGAGAGAGCCUUCAGGAGGGGGGGGGAUCCAGGUGAGCCAUAGUGCCU   791.4
.(((((((((.(((((((((((((((((((((((((((((((.(((((......)))))....((((.((.((......)).)).))))))))))))))))))).))))))))))).))))).))))))...)))).
.................UCCCUCUCUCCCUUGAAGGC....................................................................................................   198.010163
..................................................................................................CUUCAGGAGGGGGGGGGAUC...................   304.812519
.................UCCCUCUCUCCCUUGAAGG.....................................................................................................   1.11468
.................UCCCUCUCUCCCUUGAAGGCU...................................................................................................   9.27662
.................UCCCUCUCUCCCUUGAAGGCUC..................................................................................................   69.68177
.................UCCCUCUCUCCCUUGAAGGU....................................................................................................   4.16731
.................UCCCUCUCUCCCUUGAAGGCA...................................................................................................   0.306198
.................UCUCUCUCUCCCUUGAAGGC....................................................................................................   4.85314
..................CCCUCUCUCCCUUGAAGGC....................................................................................................   4.43622
..................................................................................................CUUCAGGAGGGGGGGGGAUCC..................   180.212967
...................................................................................................UUCAGGAGGGGGGGGGAUC...................   0.306198
...................................................................................................UUCAGGAGGGGGGGGGAUCC..................   14.3144
```

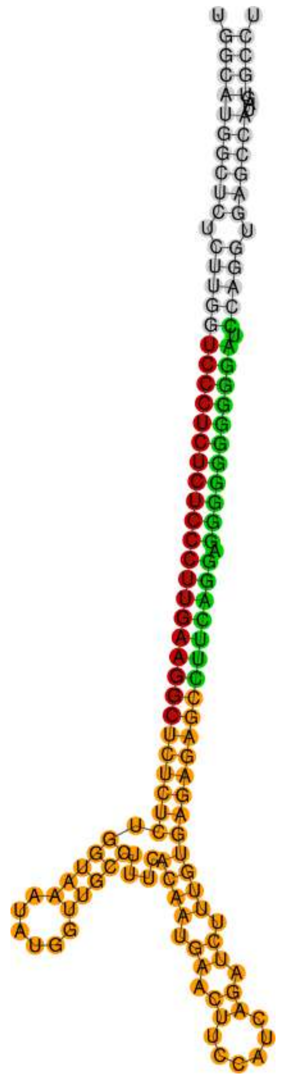

zma-MIR_N62a

Chromosome: chr2
Start: 32718058
End: 32718252
Strand: -
Strand bias: 1.0
Abundance bias: 0.931
MFE: -96.80

```
                      Mature                                                                                                                                             Star
AAAAAUCAGGUUUGCUCAAACCGGAUGCUAACGUGGUGUGCCAUGUUGGAACACACUAAUGAAAGUUCGGGGACCCAAAUGACAUAUUAUACCAACUUAAGAGACUCAAGUGACACAGCAAUGCCAAGUUCUAGGACCCAAGUGAGUCUAACGUGGCACGCCACGUCGGUAUCCGGUUUGAGCAAACCUGGUUUU   400.8
.((((((((((((((((((((((((((((.((((((((((((((((((((.(.((((..((...((((.(((((.....((.(((.........((((.((...)).)))).........))).)).))))).)))).)))))).))))))))))))))))))))))).))))))))))))))))))))))))))))
....................CCGGAUGCUAACGUGGUGUGC..........................................................................................................................................................   291.690191
.............................................................................................................................................................ACGCCACGUCGGUAUCCGGUU.................   57.687516
.................AAACCGGAUGCUAACGUGGUGUGC..........................................................................................................................................................   2.537851
.................AAACCGGAUGCUAAUGUGGUGUGC..........................................................................................................................................................   2.81519
..................ACCGGAUGCUAAUGUGGUGUGCCA.........................................................................................................................................................   0.749285
....................CCGGAUGCUAACGUGGUGUGU..........................................................................................................................................................   4.70642
.....................CGGAUGCUAACGUGGUGUGCC.........................................................................................................................................................   1.874038
............................................................................................................................................................CACGCCACGUCGGUAUCCGGU..................   1.847265
..............................................................................................................................................................CGCCACGUCGGUAUCCGGUUU................   1.974365
..............................................................................................................................................................CGCCACGUCGGCAUCCGGUUU................   1.95085
................................................................................................................................................................CCACGUCGGCAUCCGGUUUGA..............   5.558593
```

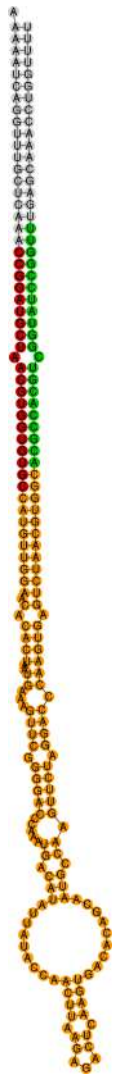

zma-MIR_N17c

Chromosome: chr2
Start: 32967259
End: 32967376
Strand: -
Strand bias: 1.0
Abundance bias: 0.961
MFE: -66.40

Mature / Star

```
GGAUAGUCUAGUAUUUAUCCUAAUACACAUGGGUUGAGCGAGAUUGGGUGUAAAUUAGUUUAAUUUACAUCACAAUCUCCUUCAACCCAUGUGUAUUGAGAUGAAUACUAUAGUAUCA    1109.5
.((((.(.((((((((((.(((((((((((((((((.((((((((((((((((((...)))))))))))).))))))).)))))))))))))))))).))))))))))).).)))).
....................UAAUACACAUGGGUUGAGCGA.............................................................................    275.7817
...............................................................................CUUCAACCCAUGUGUAUUGAG..................    22.17699
...................CUAAUACACAUGGGUUGAGC...............................................................................    4.41886
...................CUAAUACACAUGGGUUGAGCGA.............................................................................    15.52662
...................CUAAUACACAUGGGUUGAGCG..............................................................................    23.57475
...................CUAAUACACAUGGGUUGAGCGAG............................................................................    2.43422
....................UAAUACACAUGGGUUGAGC...............................................................................    1.5596
....................UAAUACACAUGGGUUGAGCG..............................................................................    56.35813
....................UAAUACACAUGGGUUGAGCGAG............................................................................    38.0569
....................UAAUACACAUGGGUUGAGCGAU............................................................................    7.72771
....................UAAUACACAUGGGUUGAGCGAA............................................................................    11.00602
.....................AAUACACAUGGGUUGAGCGA.............................................................................    30.98355
.....................AAUACACAUGGGUUGAGCG..............................................................................    4.25375
.....................AAUACACAUGGGUUGAGCGAG............................................................................    155.04811
......................AUACACAUGGGUUGAGCGAG............................................................................    122.57954
......................AUACACAUGGGUUGAGCGA.............................................................................    12.76212
......................AUACACAUGGGUUGAGCGAGA...........................................................................    2.43422
.......................UACACAUGGGUUGAGCGAG............................................................................    3.23393
..............................................................................CCUUCAACCCAUGUGUAUUGA...................    93.63659
...............................................................................CUUCAACCCAUGUGUAUUGAGA.................    6.27671
...............................................................................CUUCAACCCAUGUGUAUUGA...................    61.22355
................................................................................UUCAACCCAUGUGUAUUGAGA.................    110.9848
.................................................................................UCAACCCAUGUGUAUUGAGAU................    4.56417
```

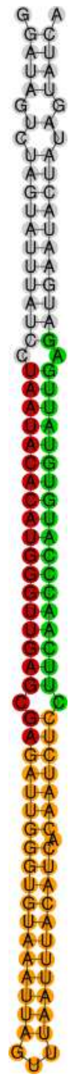

zma-MIR_N63a

Chromosome: chr2
Start: 59486219
End: 59486518
Strand: -
Strand bias: 1.0
Abundance bias: 0.755
MFE: -220.80

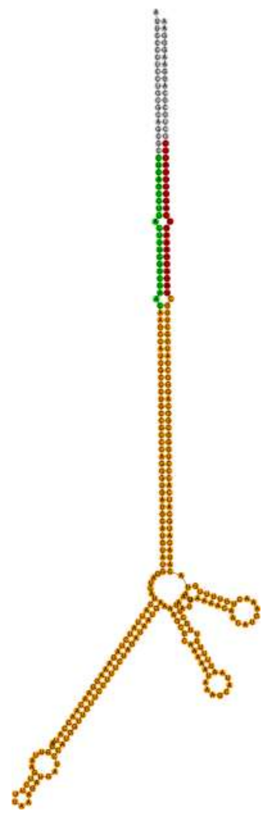

```
                    Star                                                                                                                                                                                                                                                      Mature
AUUCCCUUCCUGCGCAGCGCCUCAGCCUGACUUUCGUCUAACAAGCCAUCGCCCUGCCGCCCAGGGUGAUGCAUGUAUGGAUCAUCAUUAUCUAGAAAGGAACAACCAUAGGUUAAGUCUUUAAAGAUGAUCCAUGGUUGUUCCUUUCUAGAUAAUGAAAAGCGCAAAAAUAAAGCAUAAUUUUUUUGCUUAAUUAAGAAAACAACAUGAAACUGUUUUGUACAUACAUGCAUCACCCUGGGCGGCAGGGCGAUGGCUUGCUAGACGAAAGCCAGGCUGAGGCGCUGCGCAGGAAGGGAA   3940.7
.((((((((((((((((((((((((((((.((((((((((.((((((((((((((((((((((((((((((((((((((.....(((((((((((((((((((((((((.((....((((....))))...))))))))))))))))))))))))))))).(((((.((((((........)))))).))))).....(.((((((.........)))))).).)).)))))))))))))))))))))))))))))))))))))).)))))))))).))))))))))))))))))))))))))))
.....................................................................................................................................................................................................................................................................UAGACGAAAGCCAGGCUGAGGC.................   203.116252
....................CUCAGCCUGACUUUCGUCUAAC..................................................................................................................................................................................................................................................................   27.203158
........................................................................................................................................................................................................................................................................ACGAAAGCCAGGCUGAGGCGCU..............   23.968266
........................................................................................................................................................................................................................................................................ACGAAAGUCAGGCUGAGGCGCU..............   211.340549
........................................................................................................................................................................................................................................................................ACGAAAGUCAGGCUGAGGCGC...............   14.101956
........................................................................................................................................................................................................................................................................ACGAAAACCAGGCUGAGGCGCU..............   12.29151
.......................................................................................................................................................................................................................................................................GACGAAAGCCAGGCUGAGGCGC...............   113.19996
.......................................................................................................................................................................................................................................................................GACGAAAGCCAGGCUGAGGCG................   0.875122
.......................................................................................................................................................................................................................................................................GACGAAAGUCAGGCUGAGGCG................   2.141796
.......................................................................................................................................................................................................................................................................GACGAAAACCAGGCUGAGGCGC...............   8.59001
.......................................................................................................................................................................................................................................................................GACGAAAGUCAGGCUGAGGCGC...............   1045.886756
......................................................................................................................................................................................................................................................................AGACGAAAGCCAGGCUGAGGCG................   57.217669
......................................................................................................................................................................................................................................................................AGACGAAAGCCAGGCUGAG...................   0.318481
......................................................................................................................................................................................................................................................................AGACGAAAGCCAGGCUGAGGCGCU..............   1.280433
......................................................................................................................................................................................................................................................................AGACGAAAGUCAGGCUGAGGCGCU..............   0.695744
......................................................................................................................................................................................................................................................................AGACGAAAGUCAGGCUGAGGCG................   4.686197
......................................................................................................................................................................................................................................................................AGACGAAAACCAGGCUGAGGCG................   10.4435
......................................................................................................................................................................................................................................................................AGACGAAAGUCAGGCUGAGGC.................   0.203188
.....................................................................................................................................................................................................................................................................UAGACGAAAGCCAGGCUGAGGCGC...............   1.668176
.....................................................................................................................................................................................................................................................................UAGACGAAAGCCAGGCUGAGG..................   1.08057
.....................................................................................................................................................................................................................................................................UAGACGAAAGUCAGGCUGAGGC.................   279.831178
.....................................................................................................................................................................................................................................................................UAGACGAAAGUCAGGCUGAGGCGC...............   4.890267
.....................................................................................................................................................................................................................................................................UAGACGAAAACCAGGCUGAGGC.................   78.09139
.....................................................................................................................................................................................................................................................................UAGACGAAAGCCAGGCCGAGGC.................   12.2865
....................................................................................................................................................................................................................................................................CUAGACGAAAGCCAGGCUGAGGCG................   1.48578
....................................................................................................................................................................................................................................................................CUAGACGAAAGCCAGGCUGAGG..................   67.630235
....................................................................................................................................................................................................................................................................CUAGACGAAAGUCAGGCUGAGG..................   286.083869
....................................................................................................................................................................................................................................................................CUAGACGAAAACCAGGCUGAGG..................   10.7507
....................................................................................................................................................................................................................................................................CUAGACGAAAACCAGGCUGAGGCG................   2.15014
...................................................................................................................................................................................................................................................................GCUAGACGAAAGCCAGGCUGAG...................   2.02607
..................................................................................................................................................................................................................................................................UGCUAGACGAAAGCCAGGCUGA....................   66.044143
..................................................................................................................................................................................................................................................................UGCUAGACGAAAACCAGGCUGA....................   11.48776
......................CAGCCUGACUUUCGUCUAACAA................................................................................................................................................................................................................................................................   18.150125
.....................UCAGCCUGACUUUCGUCUAACA.................................................................................................................................................................................................................................................................   17.369849
.....................UCAGCCUGACUUUCGUCUAAC..................................................................................................................................................................................................................................................................   9.389615
...................CCUCAGCCUGACUUUCGUCUA....................................................................................................................................................................................................................................................................   2.568635
...................CCUCAGCCUGACUUUCGUCUAA...................................................................................................................................................................................................................................................................   40.731397
..................GCCUCAGCCUGACUUUCGUCUA....................................................................................................................................................................................................................................................................   2.720564
.................CGCCUCAGCCUGACUUUCGUCU.....................................................................................................................................................................................................................................................................   0.713025
```

zma-MIR_N68a

```
Chromosome:    chr2
Start:         111713754
End:           111713986
Strand:        -
Strand bias:   1.0
Abundance bias:0.922
MFE:           -170.00
```

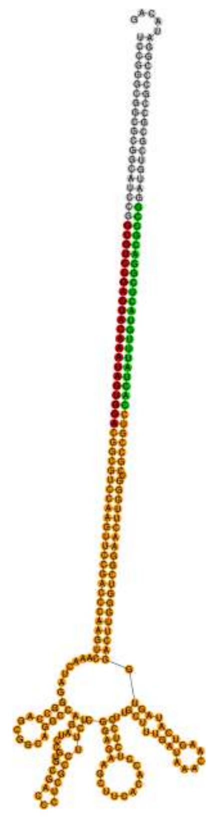

```
                    Mature                                                                                                                                                                           Star
UCCGGGCGGCGCGGCAUCCGGCGUCCGAGUACAAAUAGUGGACGGCGUCCAAGUUCCGACCCAAGUCAAACUAGGGCCAGCGGCAGGCCAGAUCCGUGCGACCCUCGCCUUCUCGGAGAAGCUUCACACCUCUCCUUGCUUUGAUAAACAAGUCAUAGUGGACUUGGGUCGGAACUUGGGUCGCCGUCCACUAUUUGUACUCGGACGCCGGAUGUCGCGCCGCCCGGAUACAG    846.
(((((((((((((((((((((((((((((((((((((((((((((((((((((((((((((((((((.......((((.......))))(((...(.((((...))))).))).(((((...........)))))..(((.((((......)))).))).)))))))))))))))))))).))))))))))))))))))))))))))))))))))))))))))))))).....
....................GCGUCCGAGUACAAAUAGUGGA...............................................................................................................................................................................................    264.92188
............................................................................................................................................................................................CACUAUUUGUACUCGGACGCCG.......................    145.76546
..................CGGCGUCCGAGUACAAAUAGUGGA...............................................................................................................................................................................................    0.644434
..................CGGCGUCCGAGUACAAAUAGUG.................................................................................................................................................................................................    2.59933
.....................CGUCCGAGUACAAAUAGUGGA...............................................................................................................................................................................................    16.586317
.......................UCCGAGUACAAAUAGUGGACGG............................................................................................................................................................................................    11.236578
..........................................................................................................................................................................................UCCACUAUUUGUACUCGGACGCCG.......................    0.273594
..........................................................................................................................................................................................UCCACUAUUUGUACUCGGACGC.........................    50.540084
...........................................................................................................................................................................................CCACUAUUUGUACUCGGACGCCGG......................    2.70511
...........................................................................................................................................................................................CCACUAUUUGUACUCGGACGCC........................    85.054072
............................................................................................................................................................................................CACUAUUUGUACUCGGACGCCGG......................    0.243195
............................................................................................................................................................................................CACUAUUUGUACUCGGACGCC........................    24.638735
............................................................................................................................................................................................CACUAUUUGUACUCGGACGCCGGA.....................    1.203368
............................................................................................................................................................................................CACUAUUUGUACUCGGACGCCA.......................    0.634079
.............................................................................................................................................................................................ACUAUUUGUACUCGGACGCCGGA.....................    0.304783
.............................................................................................................................................................................................ACUAUUUGUACUCGGACGCCG.......................    7.675648
.............................................................................................................................................................................................ACUAUUUGUACUCGGACGCCGG......................    24.632645
..............................................................................................................................................................................................CUAUUUGUACUCGGACGCCGGA.....................    47.384194
...............................................................................................................................................................................................UAUUUGUACUCGGACGCCGGAU....................    93.493778
...............................................................................................................................................................................................UAUUUGUACUCGGACGCCGGA.....................    0.365186
```

zma-MIR_N77a

Chromosome: chr2
Start: 174363167
End: 174363285
Strand: -
Strand bias: 1.0
Abundance bias: 0.977
MFE: -68.80

```
                  Mature                                                      Star
AGUUUGGGAGCCACAAAACCGGAGGGGUUGAAGGUGCUAAAAUCCUCUUUUAUUCAAUUUUGAAUAAAAAGGGGAUUUUAGCUCCUCCAAUCUCCUCCGGUAUUUUAGCUCUCAAACUA    843.
(((((((((((......(((((((((((((.(((.(((((((((((((((((((((....))))).))))))))))))))))).))).))))).))))))))......))))))))))).
..................CCGGAGGGGUUGAAGGUGCUA................................................................................    296.758756
................................................................................GCUCCUCCAAUCUCCUCCGGUA.................    37.886021
................AACCGGAGGGGUUGAAGGUGC..................................................................................    42.236469
.................ACCGGAGGGGUUGAAGGUGC..................................................................................    5.803248
.................ACCGGAGGGGUUGAAGGUGCU.................................................................................    126.844126
..................CCGGAGGGGUUGAAGGUGCU.................................................................................    5.616248
...................CGGAGGGGUUGAAGGUGCUAA...............................................................................    3.128166
...................CGGAGGGGUUGAAGGUGCUA................................................................................    255.900087
...................CGGAGGGGUUGAAGGUGCU.................................................................................    4.780313
...................CGGAGGGGUUGAAGGUGCUU................................................................................    0.273594
....................GGAGGGGUUGAAGGUGCUAA...............................................................................    0.956615
....................GGAGGGGUUGAAGGUGCUA................................................................................    1.43866
...............................................................................AGCUCCUCCAAUCUCCUCCGGU..................    0.563734
................................................................................GCUCCUCCAAUCUCCUCCGGU..................    30.740179
.................................................................................CUCCUCCAAUCUCCUCCGGUAU................    10.078875
..................................................................................UCCUCCAAUCUCCUCCGGUAUU...............    1.031506
```

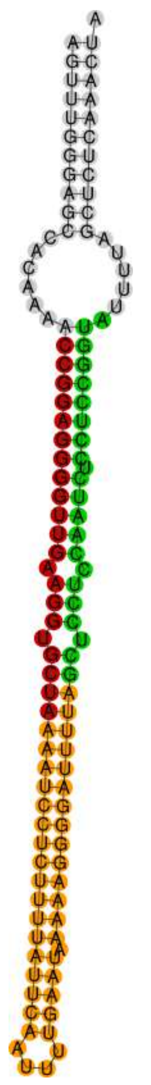

zma-MIR_N83a

Chromosome: chr2
Start: 238612185
End: 238612302
Strand: -
Strand bias: 1.0
Abundance bias: 0.999
MFE: -55.20

```
                  Mature                                                                 Star
GAUGUAAUUUUUCUAUCCUUUCAUGCUAUCGUCAGUGGCACAAAAGGAUAAGUACAUGUGAUUUUCUUACCCUUUUACGCCACUGACGAGGGCAUGAAAAAGGUAAGAAAUUGCAUCC   1673.1
((((((((((((.((((.(((((((((.(((((((((((..((((((.((((.............)))).))))))..))))))))))).)))))))))..)))))))))))))))).
..................UUUCAUGCUAUCGUCAGUGGC...............................................................................   1084.74138
................................................................................CACUGACGAGGGCAUGAAAAA.................   12.460494
.................CUUUCAUGCUAUCGUCAGUGG................................................................................   1.250585
.................CUUUCAUGCUAUCGUCAGUG.................................................................................   0.956615
.................CUUUCAUGCUAUCGUCAGUGGC...............................................................................   21.556688
.................CUUUCAUGCUAUCGUCAGU..................................................................................   1.11605
..................UUUCAUGCUAUCGUCAGUGG................................................................................   0.318481
..................UUUCAUGCUAUCGUCAGUG.................................................................................   0.956615
..................UUUCAUGCUAUCGUCAGU..................................................................................   1.82635
..................UUUCAUGCUAUCGUCAGUGGCA..............................................................................   2.96808
..................UUUCAUGCUAUCGUCAGUGGCU..............................................................................   0.371561
..................UUUCACGCUAUCGUCAGUGGC...............................................................................   4.70928
..................UUUCAUGCUAUCGUCAUUGGC...............................................................................   3.13952
..................UUUCAUGCUAUCGUCAGUGGU...............................................................................   24.44375
...................UUCAUGCUAUCGUCAGUGGCA..............................................................................   490.26026
...................UUCAUGCUAUCGUCAGUGGC...............................................................................   5.131216
...................UUCAUGCUAUCGUCAGCGGCA..............................................................................   0.318481
...............................................................................CCACUGACGAGGGCAUGAAAA..................   0.762188
................................................................................CACUGACGAGGGCAUGAAAAAGG...............   8.928128
................................................................................CACUGACGAGGGCAUGAAAAAG................   0.530802
................................................................................CACUGACGAGGGCAUGAAAAAGGU..............   0.459297
.................................................................................ACUGACGAGGGCAUGAAAAAGG...............   3.51561
```

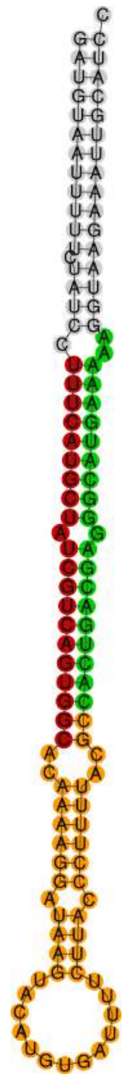

Star Mature

```
UUUCUAAUUGUGAUUUUGCCCUCACUUUUUUCGGAUGAAGUUGAAAGUUUUCCCUACUCUGGAAGAAAAGUGAGGAUAAAAUCACAAAAUAGGGUAACUUUUCAACUUCAUCCGAGAAAAGUGGGGGCAAAAUCACAAAGUUUGA    298.3
.......((((((((((((((((((((((((((((((((((((((((((..(((((.(((...)))...((((........))))....))))).))).))))))))))))))))))))))))))))))))))))))).......
..........................................................................................................UUCAUCCGAGAAAAGUGGGGG..................    276.347553
....................CUCACUUUUUUCGGAUGAAGU........................................................................................................    10.483567
...........................................................................................................UCAUCCGAGAAAAGUGGGGGC.................    0.689122
...........................................................................................................UCAUCCGAGAAAAGUGGGGG..................    1.226975
...................CCUCACUUUUUUCGGAUGAAG.........................................................................................................    0.660362
```

zma-MIR_N91a

```
Chromosome:    chr3
Start:         94788024
End:           94788168
Strand:        +
Strand bias:   1.0
Abundance bias:0.97
MFE:           -74.70
```

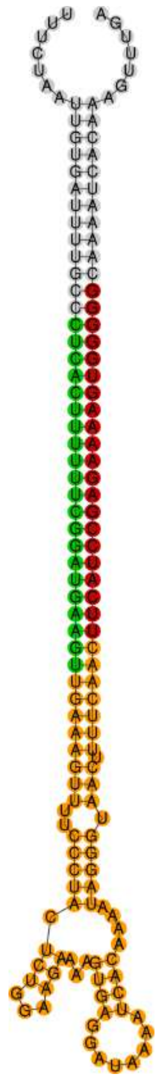

zma-MIR_N103a

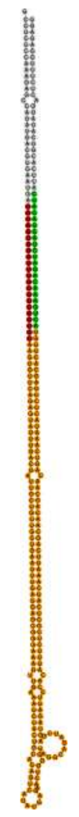

```
Chromosome:    chr3
Start:         233860433
End:           233860700
Strand:        +
Strand bias:   1.0
Abundance bias:0.994
MFE:           -196.20
```

```
                                Mature                                                                                                                                                                                                 Star
GCCUCUCCAAAGAGCCAUAUGACGUGAAUGAUGUAGUAGAUCUGUUUCAGUACAAGUGUUCAGAGUAUUUGACUAUUGCUGUUGCUGAAUUUGAUGAGCGGGAAAUUUGAAAUCUACUGCACAGUCAUAUGAGAACUGGGUAGGCAGAUCGAGUAGAUUCCACAUUUCUCGCUCAUCAAAUUCAGCAACAGCAACAGUCAAAUACUCUGAGCACUUGUACUGAAACAGAUCUACUACAUCAUUCACGUCAUAUGACUCUUUGGAGAGG 9999.0
.(((((((((((((.(((((((((((((((((((((((((((((((((((((((((((((((((((((((((((.(((((((((((((((((((((((((((((((.((.((((((((((.(((((......).)))).))..........)))))))).)).))))))))))))))))))))))))))))))).))))))))))))))))))))))))))))))))))))))))))))))))))))))))))).)))))))))))))
...............................UGUAGUAGAUCUGUUUCAGUAC....................................................................................................................................................................................................................... 3064.639486
..........................................................................................................................................................................................................................ACUGAAACAGAUCUACUACAUC............................ 7.92169
............................UGAUGUAGUAGAUCUGUUUCAG.......................................................................................................................................................................................................................... 147.557092
............................UGAUGUAGUAGAUCUGUUUCAGUA........................................................................................................................................................................................................................ 3.637417
.............................GAUGUAGUAGAUCUGUUUCAGUAC....................................................................................................................................................................................................................... 8.195656
.............................GAUGUAGUAGAUCUGUUUCAGU......................................................................................................................................................................................................................... 19.501707
..............................AUGUAGUAGAUCUGUUUCAGU......................................................................................................................................................................................................................... 96.088871
..............................AUGUAGUAGAUCUGUUUCAGUA........................................................................................................................................................................................................................ 242.38528
..............................AUGUAGUAGAUCUGUUUCAGUAC....................................................................................................................................................................................................................... 0.863864
..............................AUGUAGUAGAUCUGUUUCAGUACA...................................................................................................................................................................................................................... 1.30192
...............................UGUAGUAGAUCUGUUUCAGU......................................................................................................................................................................................................................... 1.7777
...............................UGUAGUAGAUCUGUUUCAGUA........................................................................................................................................................................................................................ 65.030699
...............................UGUAGUAGAUCUGUUUCAGUAU....................................................................................................................................................................................................................... 434.13322
...............................UGUAGUAGAUCUGUUUCAGUAA....................................................................................................................................................................................................................... 38.655655
................................GUAGUAGAUCUGUUUCAGUACAAG.................................................................................................................................................................................................................... 3.83152
................................GUAGUAGAUCUGUUUCAGUAC....................................................................................................................................................................................................................... 87.797462
................................GUAGUAGAUCUGUUUCAGUAU....................................................................................................................................................................................................................... 10.088638
.................................UAGUAGAUCUGUUUCAGUACAAG.................................................................................................................................................................................................................... 24.971202
.................................UAGUAGAUCUGUUUCAGUAC....................................................................................................................................................................................................................... 161.110948
.................................UAGUAGAUCUGUUUCAGUACAA..................................................................................................................................................................................................................... 35.217128
.................................UAGUAGAUCUGUUUCAGUACA...................................................................................................................................................................................................................... 69.372933
.................................UAGUAGAUCUGUUUCAGUAU....................................................................................................................................................................................................................... 7.998254
..................................AGUAGAUCUGUUUCAGUACAAG.................................................................................................................................................................................................................... 13.099494
.......................................................................................................................................................................................................................UGUACUGAAACAGAUCUACUAC............................... 223.878252
.......................................................................................................................................................................................................................UGUACUGAAACAGAUCUACUACAU............................. 5.831941
.......................................................................................................................................................................................................................UGUACUGAAACAGAUCUACUACA.............................. 38.697385
.......................................................................................................................................................................................................................UGUACUGAAACAGAUCUACUAU............................... 0.842035
.........................................................................................................................................................................................................................UACUGAAACAGAUCUACUACAU............................. 175.074094
.........................................................................................................................................................................................................................UACUGAAACAGAUCUACUACA.............................. 17.851296
..........................................................................................................................................................................................................................ACUGAAACAGAUCUACUACAU............................. 1.151826
...........................................................................................................................................................................................................................CUGAAACAGAUCUACUACAUCA........................... 2.43422
............................................................................................................................................................................................................................UGAAACAGAUCUACUACAUCAU.......................... 2.683969
.............................................................................................................................................................................................................................GAAACAGAUCUACUACAUCAUU......................... 1.811774
```

	Mature		Star		
AUUUGUUGAUCGGGGAUC	CCGAGGGGAUCCAUGGGAGAA	AAAUCGUCUUGCUAUUCAAAAUUGUAUUGUAAGGGGAUUCCU	CGCCUAUGGAUCCUCUCGAGA	UCCCUAACCACCAAAUC	254.8

```
AUUUGUUGAUCGGGGAUCCCGAGGGGAUCCAUGGGAGAAAAAUCGUCUUGCUAUUCAAAAUUGUAUUGUAAGGGGAUUCCUCGCCUAUGGAUCCUCUCGAGAUCCCUAACCACCAAAUC	254.8
(((((.((...(((((((.((((((((((((((((.((..((((.((((((....((....))....)))))).))))..)).)))))))))))))))).)))))))...)).))))).
..................CCGAGGGGAUCCAUGGGAGAA................................................................................	145.191649
.................................................................................CGCCUAUGGAUCCUCUCGAGA.................	96.941042
................................................................................UCGCCUAUGGAUCCUCUCGAG..................	7.752684
.................................................................................CGCCUAUGGAUCCUCUCGAG..................	4.210282
.................................................................................CGCCUAUGGAUCCUCUCGAGAA................	0.152391
```

zma-MIR_N92a

Chromosome: chr3
Start: 123760327
End: 123760445
Strand: -
Strand bias: 1.0
Abundance bias: 0.998
MFE: -59.70

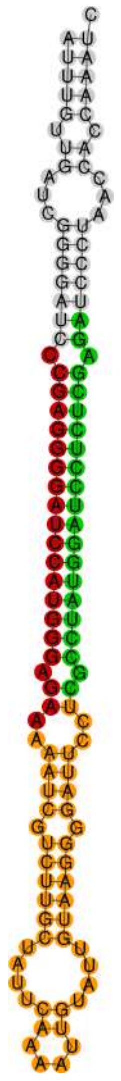

zma-MIR_N96a

Chromosome: chr3
Start: 174892282
End: 174892472
Strand: -
Strand bias: 1.0
Abundance bias: 0.885
MFE: -95.40

```
              Mature                                                                                                                                          Star
UCCCACGGAUUUGCUCAAUCCGGACGCCGAUGUGGCGUGCCACGUUGGAGCAUACUAAUGAAAGUUCCGAGACCCAGUGACACAACAUGUCAAGUUUGGGACCCGAGUGACACAACAUGCCAAGUUCGAGGACCCAAGUGACUCUAACGUGGUACGCCACGUCGGUAUCCGGUUUGAGCAAAACCGGUUUU    633.6
.....(((.(((((((((.(((((.(((((((((((((((((((((((((((((((......))).............(((((.....)))))...(((((.((((((((.((.....)).))..)))).)).)))))))).))))))))))))))))))))))))))).))))).))))))))).))).....
..............UCAAUCCGGACGCCGAUGUGG............................................................................................................................................................    523.255393
.............................................................................................................................................................ACGUCGGUAUCCGGUUUGAGC.............    22.943524
.............CUCAAUCCGGACGCCGAUGUGG............................................................................................................................................................    0.279384
.............CUCAAUCCGGACGCCGAUGUG.............................................................................................................................................................    0.816266
..............UCAAUCCGGACGCCGAUGUGGC...........................................................................................................................................................    0.17779
..............UCAAUCCGGACGCCGAUG...............................................................................................................................................................    0.152391
..............UCAAUCCGGACGCCGAUGU..............................................................................................................................................................    2.338328
..............UCAAUCCGGACGCCGAUGUGA............................................................................................................................................................    3.046476
...............CAAUCCGGACGCCGAUGUGGC...........................................................................................................................................................    3.007442
................................................................................................................................................................UCGGUAUCCGGUUUGAGCA............    3.31562
................................................................................................................................................................UCGGUAUCCGGUUUGAGCAAA..........    1.236604
```

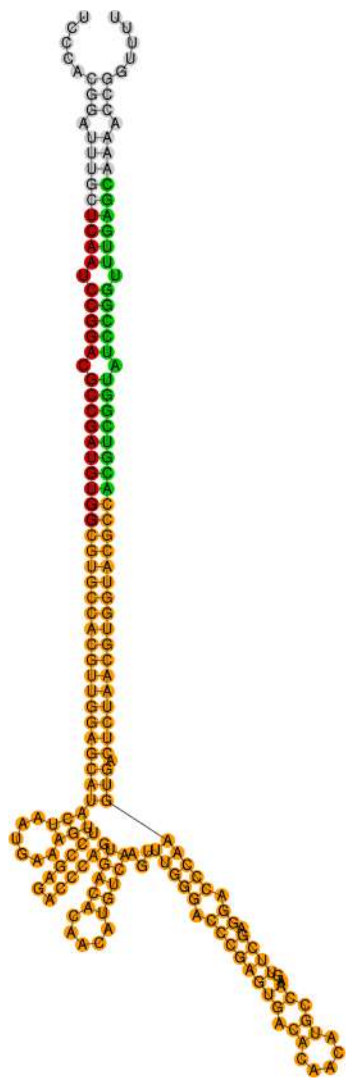

zma-MIR_N98a

Chromosome: chr3
Start: 190102398
End: 190102535
Strand: -
Strand bias: 0.98
Abundance bias: 0.894
MFE: -71.80

```
                   Star                                                                Mature
UGGAAACCCAUAGAAAAUCCAGGGGUUCAGCUUGUUAAGUCAGCAACAUACACAAUGACAAAUUUACAGGUAGCUUCAAUAUCACUGUGUAUGCUGCUGCCUUAAUAAUCUGAAUCCGCGGGUUUUCUAUGGGUUUCC    600.2
.(((((((((((((((((((..((((((((.((((((((.(((((.((((((((.(((...(((...((....))..))).))).)))))))).))))).)))))))).))))))))..)))))))))))))))))))
...................................................................................................CCUUAAUAAUCUGAAUCCGCGG.................    294.23878
....................AGGGGUUCAGCUUGUUAAGUCA................................................................................................    79.638038
......................................................................................................UAAUAAUCUGAAUCCGCGGGU...............    56.121964
...................................................................................................CCUUAAUAAUCUGAAUCCGC...................    3.017264
...................................................................................................CCUUAAUAAUCUGAAUCCGCG..................    19.115255
..................................................................................................GCCUUAAUAAUCUGAAUCCGC...................    32.880939
.................................................................................................UGCCUUAAUAAUCUGAAUCCG....................    49.781393
.......................GGUUCAGCUUGUUAAGUCAGC..............................................................................................    1.57832
```

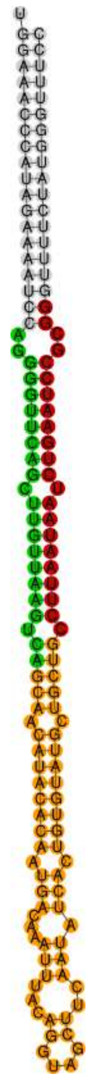

zma-MIR_N99a

```
Chromosome:    chr3
Start:         197787832
End:           197787948
Strand:        -
Strand bias:   1.0
Abundance bias:0.984
MFE:           -53.60
```

```
                       Star                                        Mature
UGAUUCUGGUGGAACGGUAGAAGGGUAAAAUAUCAUAAAUAAACUGAAAUAAGCUACCUAAAUAGCUUAUUUCAGAUUAUUUAUGAUAUGUUACUCUACUACUCUUUUAUCAUAAUC    1183.6
.((((.((((((((.(((((.(((((((.(((((((((((((.(((((((((((((......))))))))))))).))))))))))))).))))))).))))).)))))))).))))
...............................................................................UUUAUGAUAUGUUACUCUACU.................    1008.155689
....................AAGGGUAAAAUAUCAUAAAUA............................................................................    10.443227
.................................................................................UAUGAUAUGUUACUCUACUACU..............    1.604696
...............................................................................UUUAUGAUAUGUUACUCU....................    0.888855
...............................................................................UUUAUGAUAUGUUACUCUACUA................    1.10171
...............................................................................UUUAUGAUAUGUUACUCUAC..................    22.20337
...............................................................................UUUAUGAUAUGUUACUCUACA.................    20.328213
...............................................................................UUUAUGAUAUGUUACUCUACC.................    66.59298
...............................................................................UUUAUGAUAUGUUACUCUACG.................    5.43726
...............................................................................UUUAUGAUAUGUUACUCUACUU................    24.325364
...............................................................................UUUAUGAUAUGUUACUCUACUC................    0.842035
.............................................................................UAUUUAUGAUAUGUUACUCUA...................    2.189054
............................................................................UUAUUUAUGAUAUGUUACUCUACU.................    0.424642
.................UAGAAGGGUAAAAUAUCAUAA...............................................................................    0.273594
```

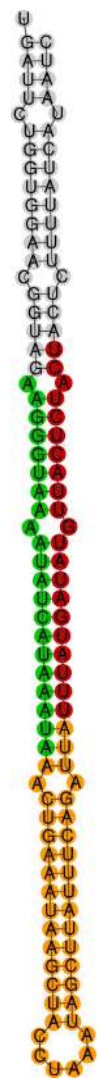

zma-MIR_N109a

```
Chromosome:    chr4
Start:         28394975
End:           28395095
Strand:        +
Strand bias:   1.0
Abundance bias:0.999
MFE:           -60.60
```

```
                 Mature                                                      Star
AGUUUGAGAGUUCAAAAUCCGGAGAGGAUUAGAGGGACUAAAAUCCCCGUUUUAUUUAAAAUUGAAUAAGGAGGAGAUUUUAGUCUCUCUAAUUCCCUCGGGUUUUGUGUCUCCCAAACUA    293.6
((((((.((((.(((((((((.((.((((((((((((((((((((.((.(((((((((....))))))))).)).))))))))))))))))))))).))))))))))).).))).)))))).
.................UCCGGAGAGGAUUAGAGGGACU..................................................................................    269.151863
...................................................................................UCUCUCUAAUUCCCUCGGGUU.................    2.75392
.................UCCGGAGAGGAUUAGAGGGAC...................................................................................    3.55014
.................UCCGGAGAGGAUUAGAGGGA....................................................................................    10.377
.................UCCGGAGAGGAUUAGAGGG.....................................................................................    6.20131
...................CGGAGAGGAUUAGAGGGACUA.................................................................................    0.760754
.................................................................................AGUCUCUCUAAUUCCCUCGGG...................    0.568493
```

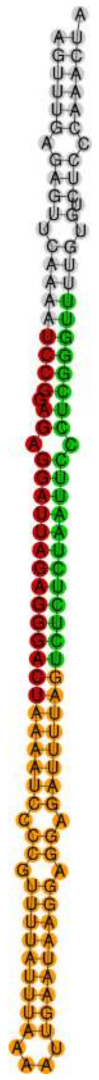

zma-MIR_N113a

Chromosome:	chr4
Start:	92593607
End:	92593808
Strand:	+
Strand bias:	1.0
Abundance bias:	0.999
MFE:	-81.70

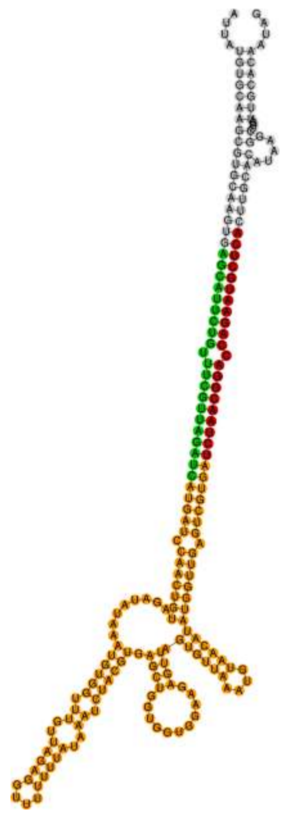

```
                     Star                                                                                                                                  Mature
AUUAUGUGCAAGCGUGCAAGUGAGCAUUCUGUUUCGUUAGAUCAUGAUCCAACUGUAGAUAUAAAUGUGGUUUGUUAGAGGUUUUUUUAUAAAUCUACGUGAGCUGGUGGUGGAAGAGUUAGUGUUAAAUGUAACAUAUGGUUGAGUCGUGAUCUAACGGACCAGAAUGCUCACUUGCACAUAAGGCCUGAUUGCACAAUAG    702.1
....(((((((((((((((((((((((((((.((((((((((((((((.(((((((........((((((.((..(((((....)))))..)).)))))).((((...........)))).((((((....))))))))))))).)))))))))))))))).)))))))))))))))))).....))....)))))))....
........................................................................................................................................................UCUAACGGACCAGAAUGCUCA.............................    289.746315
......................AGCAUUCUGUUUCGUUAGAUC...............................................................................................................................................................    5.142426
...........................................................................................................................................................AACGGACCAGAAUGCUCACU...........................    0.17779
..........................................................................................................................................................UAACGGACCAGAAUGCUCACU...........................    105.17736
........................................................................................................................................................UCUAACGGACCAGAAUGCUCACUU..........................    5.375128
........................................................................................................................................................UCUAACGGACCAGAAUGCUC..............................    0.965145
........................................................................................................................................................UCUAACGGACCAGAAUGCUCACU...........................    0.253986
...................GUGAGCAUUCUGUUUCGUUAGAUC...............................................................................................................................................................    8.684629
```

zma-MIR_N14a

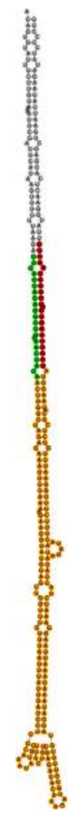

```
Chromosome:    chr6
Start:         103648322
End:           103648614
Strand:        -
Strand bias:   1.0
Abundance bias:0.815
MFE:           -109.20
```

```
                                           Star                                                                                                                                                                                                   Mature
ACAGUGUAUUUCUUGUACAAUUCGACUCAUAAGUUUUUUAAGAUUCAUCACAGGCUUCAACUACAACUUGGCUAGAGAAAUUUUAAAAGAAGGUAUGUAGCUGGUUUGUUUAAUUUAUUUAUCCUCCCAAAUAUGGUUACUGUCGAGACACAAAGAUGUGUUUUGUAGGAUAAAUAAAUUAAGGAAAUCUACUAUAUAUAUAACUUUCUUUGGAAUUUGUCUCGCAUAAGUUAUAGUUAAGCCGUGUGAUAAAUCUUAUGGAACUCAUGAGCUGAAUUAUAAGAUAUCCCCUG 1824.8
.(((.(.((.(((((((..(((((.(((((.((((((.(((((((.(((((((((((.(((((.((((((((.(((.((((((((((.((((((((((((..(((((.(((((((((((((((((.(((....))).......(((((((((....))))))))).))))))))))))))))).))))).)).))))).....)))))))))))))))).))).)).)))))).)))))))))).)))))).))))))).)))))).))))).)))))))))))).)).).)))
.........................................................................................................................................................................................................................................UAGUUAAGCCGUGUGAUAAAUC...................................... 1217.63071
...........................................UUCAUCACAGGCUUCAACUACA.................................................................................................................................................................................................................................... 108.791627
..........................................................................................................................................................................................................................................AGUUAAGCCGUGUGAUAAAUC...................................... 0.457174
.........................................................................................................................................................................................................................................UAGUUAAGCCGUGUGAUAA......................................... 1.68796
.........................................................................................................................................................................................................................................UAGUUAAGCCGUGUGAUAAAUCUU.................................... 0.330181
.........................................................................................................................................................................................................................................UAGUUAAGCCGUGUGAUAAAU....................................... 38.740928
.........................................................................................................................................................................................................................................UAGUUAAGCCGUGUGAUAAAUCU..................................... 3.97663
.........................................................................................................................................................................................................................................UAGUUAAGCCGUGUGAUAAA........................................ 5.93418
.........................................................................................................................................................................................................................................UAGUUAAGCCGUGGGAUAAAUC...................................... 0.482573
.........................................................................................................................................................................................................................................UAGUUAAGCCGUGUGAUAAAGC...................................... 0.203188
.........................................................................................................................................................................................................................................UGGUUAAGCCGUGUGAUAAAUC...................................... 0.17779
.........................................................................................................................................................................................................................................UAGUUAAGCCGUGUGAUAAUU....................................... 0.330181
.........................................................................................................................................................................................................................................UAGUUAAGCCGGGUGAUAAAUC...................................... 2.622813
.........................................................................................................................................................................................................................................UAGCUAAGCCGUGUGAUAAAUC...................................... 0.17779
.........................................................................................................................................................................................................................................UAGUUAAGCCGUGUGAUAAAUCA..................................... 0.380978
.........................................................................................................................................................................................................................................UAGUUAAGCCGUGCGAUAAAUC...................................... 0.431775
.........................................................................................................................................................................................................................................UAGUUAAGCCGCGUGAUAAAUC...................................... 0.431775
.........................................................................................................................................................................................................................................UAGGUAAGCCGUGUGAUAAAUC...................................... 0.304783
.........................................................................................................................................................................................................................................UAGUUAAGCCGUGUGAGAAAUC...................................... 0.253986
.........................................................................................................................................................................................................................................UAGUUAAGCCGUGUGAUAAAUA...................................... 4.93325
.........................................................................................................................................................................................................................................UAGUUAAGCCGUGUGAUAAAUG...................................... 0.558768
.........................................................................................................................................................................................................................................UAGUUAAGCCGUGUGAUAAAUU...................................... 22.55357
.........................................................................................................................................................................................................................................UAGUUAAGUCGUGUGAUAAAUC...................................... 0.17779
.........................................................................................................................................................................................................................................UAGUUAAGCCGUGUGAUAAAUUUU.................................... 0.304783
.........................................................................................................................................................................................................................................UAGUUAAGCCGUGUGAUAAU........................................ 0.228587
.........................................................................................................................................................................................................................................UAGUUAAGCCGUGUGAUAAAUUU..................................... 1.918414
.........................................................................................................................................................................................................................................UAGUUAAGCCGAGUGAUAAAUC...................................... 0.203188
.........................................................................................................................................................................................................................................UAGUUAAGCCGUGUGAUAAACC...................................... 0.228587
........................................................................................................................................................................................................................................AUAGUUAAGCCGUGUGAUAAA........................................ 0.406377
........................................................................................................................................................................................................................................AUAGUUAAGCCGUGUGAUAA......................................... 0.279384
........................................................................................................................................................................................................................................AUAGUUAAGCCGUGUGAUAAAU....................................... 1.525779
.......................................................................................................................................................................................................................................UAUAGUUAAGCCGUGUGAUAAA........................................ 3.065301
.......................................................................................................................................................................................................................................UAUAGUUAAGCCGUGUGAUAA......................................... 3.25382
.......................................................................................................................................................................................................................................UAUAGUUAAGCCGUGUGAUAAU........................................ 0.304783
.......................................................................................................................................................................................................................................UAUAGUUAAGCCGUGUGAUAAAUU...................................... 0.431775
......................................................................................................................................................................................................................................UUAUAGUUAAGCCGUGUGAUAA......................................... 0.787355
......................................................................................................................................................................................................................................UUAUAGUUAAGCCGUGUGAUAAAUC...................................... 0.406377
.............................................CAUCACAGGCUUCAACUACA.................................................................................................................................................................................................................................... 0.799007
.............................................CAUCACAGGCUUCAACUACAAC.................................................................................................................................................................................................................................. 4.088
.............................................CAUCACAGGCUUCAACUACAA................................................................................................................................................................................................................................... 0.761957
.............................................CAUCACAGGCUUCAACUACAACU................................................................................................................................................................................................................................. 1.857825
.............................................CAUCACAGGCUUCAACUACU.................................................................................................................................................................................................................................... 0.605605
............................................UCAUCACAGGCUUCAACUAC..................................................................................................................................................................................................................................... 1.347989
............................................UCAUCACAGGCUUCAACUACAA................................................................................................................................................................................................................................... 2.115776
............................................UCAUCACAGGCUUCAACU....................................................................................................................................................................................................................................... 0.921107
............................................UCAUCACAGGCUUCAACUACA.................................................................................................................................................................................................................................... 2.560134
............................................UCAUCACAGGCUUCAACUG...................................................................................................................................................................................................................................... 0.17779
............................................UCAUCACAGGCUUCAACUACU.................................................................................................................................................................................................................................... 2.208509
...........................................UUCAUCACAGGCUUCAACU....................................................................................................................................................................................................................................... 2.794547
...........................................UUCAUCACAGGCUUCAACUAC..................................................................................................................................................................................................................................... 18.402987
...........................................UUCAUCACAGGCUUCAACUACAACU................................................................................................................................................................................................................................. 0.631003
...........................................UUCAUCACAGGCUUCAACUA...................................................................................................................................................................................................................................... 0.783395
...........................................UUCAUCACAGGCUUCAACUACAA................................................................................................................................................................................................................................... 2.28377
...........................................UUCAUCACAGGCUUCAACUACU.................................................................................................................................................................................................................................... 15.99696
...........................................UUCAUCACAGGCUUCAACUACAU................................................................................................................................................................................................................................... 1.414399
..........................................AUUCAUCACAGGCUUCAACU....................................................................................................................................................................................................................................... 0.784328
..........................................AUUCAUCACAGGCUUCAACUAC..................................................................................................................................................................................................................................... 0.53337
..........................................AUUCAUCACAGGCUUCAACUA...................................................................................................................................................................................................................................... 0.580206
........................................AGAUUCAUCACAGGCUUCAACUAC..................................................................................................................................................................................................................................... 0.330181
```

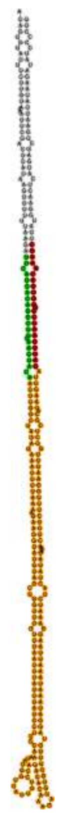

zma-MIR_N14b

```
Chromosome:    chr6
Start:         103722543
End:           103722833
Strand:        -
Strand bias:   1.0
Abundance bias:0.813
MFE:           -106.90
```

Star / Mature

```
ACAGUGUAUUUCUUGUACAAUUCGACUCAUAAGUUUUUUAAGAUUCAUCACAGGCUUCAACUACAACUUGGCUAGAGAAAUUUUAAAAGAAGGUAUGUAGCUGGUUUGUUUAAUUUAUUUAUCCUCCCAAAUAUGGUUACUGUCCAGACACAAAGAUGUGUUUUGUAGGAUAAAUAAAUUAAGGAAAUCUACUAUAUAUAACUUUCUUUGGAAUUUGUCUCGCAUAAGUUAUAGUUAAGCCGUGUGAUAAAUCUUAUGGAACUCAUGAGCUGAAUUAUAAGAUAUCCCCUG 1828.4
.(((.(.((.(((((((..(((((.(((((.((((((.(((((((.(((((((((((.(((((.((((((((.(((.((((((((((.(((((((((((..(((((.(((((((((((((((((..(((...(((.......)))((((((....)))))))).)))))))))))))))))).)))))..))))))...)))))))))))))))).))).)).)))))).))))))))))).)))))).))))))).)))))).))))).))))))))))))).)).).)))
.......................................................................................................................................................................................................................................UAGUUAAGCCGUGUGAUAAAUC...................................... 1217.63071
...........................................UUCAUCACAGGCUUCAACUACA.................................................................................................................................................................................................................................. 108.791627
........................................................................................................................................................................................................................................AGUUAAGCCGUGUGAUAAAUC...................................... 0.457174
.......................................................................................................................................................................................................................................UAGUUAAGCCGUGUGAUAA......................................... 1.68796
.......................................................................................................................................................................................................................................UAGUUAAGCCGUGUGAUAAAUCUU.................................... 0.330181
.......................................................................................................................................................................................................................................UAGUUAAGCCGUGUGAUAAAU....................................... 38.740928
.......................................................................................................................................................................................................................................UAGUUAAGCCGUGUGAUAAAUCU..................................... 3.97663
.......................................................................................................................................................................................................................................UAGUUAAGCCGUGUGAUAAA........................................ 5.93418
.......................................................................................................................................................................................................................................UAGUUAAGCCGUGGGAUAAAUC...................................... 0.482573
.......................................................................................................................................................................................................................................UAGUUAAGCCGUGUGAUAAAGC...................................... 0.203188
.......................................................................................................................................................................................................................................UGGUUAAGCCGUGUGAUAAAUC...................................... 0.17779
.......................................................................................................................................................................................................................................UAGUUAAGCCGUGUGAUAAUU....................................... 0.330181
.......................................................................................................................................................................................................................................UAGUUAAGCCGGGUGAUAAAUC...................................... 2.622813
.......................................................................................................................................................................................................................................UAGCUAAGCCGUGUGAUAAAUC...................................... 0.17779
.......................................................................................................................................................................................................................................UAGUUAAGCCGUGUGAUAAAUCA..................................... 0.380978
.......................................................................................................................................................................................................................................UAGUUAAGCCGUGCGAUAAAUC...................................... 0.431775
.......................................................................................................................................................................................................................................UAGUUAAGCCGCGUGAUAAAUC...................................... 0.431775
.......................................................................................................................................................................................................................................UAGGUAAGCCGUGUGAUAAAUC...................................... 0.304783
.......................................................................................................................................................................................................................................UAGUUAAGCCGUGUGAGAAAUC...................................... 0.253986
.......................................................................................................................................................................................................................................UAGUUAAGCCGUGUGAUAAAUA...................................... 4.93325
.......................................................................................................................................................................................................................................UAGUUAAGCCGUGUGAUAAAUG...................................... 0.558768
.......................................................................................................................................................................................................................................UAGUUAAGCCGUGUGAUAAAUU...................................... 22.55357
.......................................................................................................................................................................................................................................UAGUUAAGUCGUGUGAUAAAUC...................................... 0.17779
.......................................................................................................................................................................................................................................UAGUUAAGCCGUGUGAUAAAUUUU.................................... 0.304783
.......................................................................................................................................................................................................................................UAGUUAAGCCGUGUGAUAAU........................................ 0.228587
.......................................................................................................................................................................................................................................UAGUUAAGCCGUGUGAUAAAUUU..................................... 1.918414
.......................................................................................................................................................................................................................................UAGUUAAGCCGAGUGAUAAAUC...................................... 0.203188
.......................................................................................................................................................................................................................................UAGUUAAGCCGUGUGAUAAACC...................................... 0.228587
......................................................................................................................................................................................................................................AUAGUUAAGCCGUGUGAUAAA........................................ 0.406377
......................................................................................................................................................................................................................................AUAGUUAAGCCGUGUGAUAA......................................... 0.279384
......................................................................................................................................................................................................................................AUAGUUAAGCCGUGUGAUAAAU....................................... 1.525779
.....................................................................................................................................................................................................................................UAUAGUUAAGCCGUGUGAUAAA........................................ 3.065301
.....................................................................................................................................................................................................................................UAUAGUUAAGCCGUGUGAUAA......................................... 3.25382
.....................................................................................................................................................................................................................................UAUAGUUAAGCCGUGUGAUAAU........................................ 0.304783
.....................................................................................................................................................................................................................................UAUAGUUAAGCCGUGUGAUAAAUU...................................... 0.431775
....................................................................................................................................................................................................................................UUAUAGUUAAGCCGUGUGAUAA......................................... 0.787355
....................................................................................................................................................................................................................................UUAUAGUUAAGCCGUGUGAUAAAUC...................................... 0.406377
.............................................CAUCACAGGCUUCAACUACA.................................................................................................................................................................................................................................. 0.799007
.............................................CAUCACAGGCUUCAACUACAAC................................................................................................................................................................................................................................ 4.088
.............................................CAUCACAGGCUUCAACUACAA................................................................................................................................................................................................................................. 0.761957
.............................................CAUCACAGGCUUCAACUACAACU............................................................................................................................................................................................................................... 1.857825
.............................................CAUCACAGGCUUCAACUACU.................................................................................................................................................................................................................................. 0.605605
............................................UCAUCACAGGCUUCAACUAC................................................................................................................................................................................................................................... 1.347989
............................................UCAUCACAGGCUUCAACUACAA................................................................................................................................................................................................................................. 2.115776
............................................UCAUCACAGGCUUCAACU..................................................................................................................................................................................................................................... 0.921107
............................................UCAUCACAGGCUUCAACUACA.................................................................................................................................................................................................................................. 2.560134
............................................UCAUCACAGGCUUCAACUG.................................................................................................................................................................................................................................... 0.17779
............................................UCAUCACAGGCUUCAACUACU.................................................................................................................................................................................................................................. 2.208509
...........................................UUCAUCACAGGCUUCAACU..................................................................................................................................................................................................................................... 2.794547
...........................................UUCAUCACAGGCUUCAACUAC................................................................................................................................................................................................................................... 18.402987
...........................................UUCAUCACAGGCUUCAACUACAACU............................................................................................................................................................................................................................... 0.631003
...........................................UUCAUCACAGGCUUCAACUA.................................................................................................................................................................................................................................... 0.783395
...........................................UUCAUCACAGGCUUCAACUACAA................................................................................................................................................................................................................................. 2.28377
...........................................UUCAUCACAGGCUUCAACUACU.................................................................................................................................................................................................................................. 15.99696
...........................................UUCAUCACAGGCUUCAACUACAU................................................................................................................................................................................................................................. 1.414399
..........................................AUUCAUCACAGGCUUCAACU..................................................................................................................................................................................................................................... 0.784328
..........................................AUUCAUCACAGGCUUCAACUAC................................................................................................................................................................................................................................... 0.53337
..........................................AUUCAUCACAGGCUUCAACUA.................................................................................................................................................................................................................................... 0.580206
........................................AGAUUCAUCACAGGCUUCAACUAC................................................................................................................................................................................................................................... 0.330181
```

zma-MIR_N14c

Chromosome: chr6
Start: 103793742
End: 103794036
Strand: -
Strand bias: 1.0
Abundance bias: 0.814
MFE: -106.60

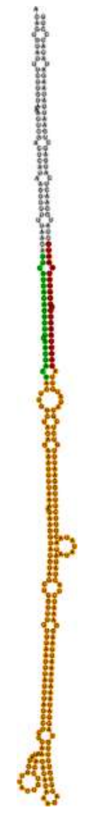

Star / Mature

```
ACAGUGUAUUUCUUGUACAAUUCGACUCAUAAGUUUUUUAAGAUUCAUCACAGGCUUCAACUACAACUUGGCUAGAGAAAUUUUAAAAGAAGGUAUGUAGCUGGUUUGUUUAAUUUAUUUAUCCUCCCAAAUAUGGUUACUGUCCAGACACAAAGAUGUGUUUUGUAGGAUAAAUAAAUUAAGGAAAUCUACUAUAUAUAUAUAACUUUCUUUGGAAUUUGUCUCGCAUAUGUUAUAGUUAAGCCGUGUGAUAAAUCUUAUGGAACUCAUGAGCUGAAUUAUAAGAUAUACCCUG 1827.8
.(((.((((.(((((((..(((((.(((((.((((((.(((((((.((((((((((.(((((.(((...((.(((.((((((((((.(((((((((((..(((((.((((((((((((((((..(((...(((.......)))(((((....))))))))).))))))))))))))))).)))))..))))))).......))))))))))))))))).))).))....))).))))))))).)))))).)))))))).))))))).)))))).))))))))))))).)))).)))
```

Read	Abundance
UAGUUAAGCCGUGUGAUAAAUC	1217.63071
UUCAUCACAGGCUUCAACUACA	108.791627
AGUUAAGCCGUGUGAUAAAUC	0.457174
UAGUUAAGCCGUGUGAUAA	1.68796
UAGUUAAGCCGUGUGAUAAAUCUU	0.330181
UAGUUAAGCCGUGUGAUAAAU	38.740928
UAGUUAAGCCGUGUGAUAAAUCU	3.97663
UAGUUAAGCCGUGUGAUAAA	5.93418
UAGUUAAGCCGUGGGAUAAAUC	0.482573
UAGUUAAGCCGUGUGAUAAAGC	0.203188
UGGUUAAGCCGUGUGAUAAAUC	0.17779
UAGUUAAGCCGUGUGAUAAUU	0.330181
UAGUUAAGCCGGGUGAUAAAUC	2.622813
UAGCUAAGCCGUGUGAUAAAUC	0.17779
UAGUUAAGCCGUGUGAUAAAUCA	0.380978
UAGUUAAGCCGUGCGAUAAAUC	0.431775
UAGUUAAGCCGCGUGAUAAAUC	0.431775
UAGGUAAGCCGUGUGAUAAAUC	0.304783
UAGUUAAGCCGUGUGAGAAAUC	0.253986
UAGUUAAGCCGUGUGAUAAAUA	4.93325
UAGUUAAGCCGUGUGAUAAAUG	0.558768
UAGUUAAGCCGUGUGAUAAAUU	22.55357
UAGUUAAGUCGUGUGAUAAAUC	0.17779
UAGUUAAGCCGUGUGAUAAAUUUU	0.304783
UAGUUAAGCCGUGUGAUAAU	0.228587
UAGUUAAGCCGUGUGAUAAAUUU	1.918414
UAGUUAAGCCGAGUGAUAAAUC	0.203188
UAGUUAAGCCGUGUGAUAAACC	0.228587
AUAGUUAAGCCGUGUGAUAAA	0.406377
AUAGUUAAGCCGUGUGAUAA	0.279384
AUAGUUAAGCCGUGUGAUAAAU	1.525779
UAUAGUUAAGCCGUGUGAUAAA	3.065301
UAUAGUUAAGCCGUGUGAUAA	3.25382
UAUAGUUAAGCCGUGUGAUAAU	0.304783
UAUAGUUAAGCCGUGUGAUAAAUU	0.431775
UUAUAGUUAAGCCGUGUGAUAA	0.787355
UUAUAGUUAAGCCGUGUGAUAAAUC	0.406377
CAUCACAGGCUUCAACUACA	0.799007
CAUCACAGGCUUCAACUACAAC	4.088
CAUCACAGGCUUCAACUACAA	0.761957
CAUCACAGGCUUCAACUACAACU	1.857825
CAUCACAGGCUUCAACUACU	0.605605
UCAUCACAGGCUUCAACUAC	1.347989
UCAUCACAGGCUUCAACUACAA	2.115776
UCAUCACAGGCUUCAACU	0.921107
UCAUCACAGGCUUCAACUACA	2.560134
UCAUCACAGGCUUCAACUG	0.17779
UCAUCACAGGCUUCAACUACU	2.208509
UUCAUCACAGGCUUCAACU	2.794547
UUCAUCACAGGCUUCAACUAC	18.402987
UUCAUCACAGGCUUCAACUACAACU	0.631003
UUCAUCACAGGCUUCAACUA	0.783395
UUCAUCACAGGCUUCAACUACAA	2.28377
UUCAUCACAGGCUUCAACUACU	15.99696
UUCAUCACAGGCUUCAACUACAU	1.414399
AUUCAUCACAGGCUUCAACU	0.784328
AUUCAUCACAGGCUUCAACUAC	0.53337
AUUCAUCACAGGCUUCAACUA	0.580206
AGAUUCAUCACAGGCUUCAACUAC	0.330181

zma-MIR_N23b

Chromosome: chr10
Start: 83684807
End: 83685055
Strand: +
Strand bias: 1.0
Abundance bias:0.921
MFE: -178.20

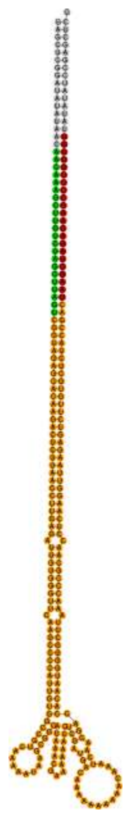

Star   Mature

```
GAGCUCGGAUAUAUAACAACAAAGCUUGUCUAGCUUAGUCGGUAGAGCACAAGACUCUUAACCUUGAGAUUGUGGGUUCAAGCCCCAUUGUGGCCGUCAAAAAUAAGCGGCAUAAACGAAAAAAUCUCGAUGACAAAAAAAAAACAAAUCAACGACCACAAUGGGGCUUAAACCCGCAACCUCAAGGUUAAGAGUCUUGUGCUCUACCGACUAAGCUAGACAAGCUUUGUUGUUAUAUAUCCGAGCUCG    119.4
((((((((((((((((((((((((((((((((((((((((((((((((((((((((((((((((.(((((((((.((((((((((((((((((.........))))))...............(((.(((...............))).))).)))))))))))))).))))))))).)))))))))))))))))))))))))))))))))))))))))))))))))))))))))))))))))).
...................................................................................................................................................................................................................UAAGCUAGACAAGCUUUGUUGU................    78.03025
.................AACAAAGCUUGUCUAGCUUAGU..................................................................................................................................................................................................................    11.697
....................................................................................................................................................................................................................AAGCUAGACAAGCUUUGUUGU................    8.05791
....................................................................................................................................................................................................................AAGCUAGACAAGCUUUGUUGUU...............    3.04278
..................................................................................................................................................................................................................CUAAGCUAGACAAGCUUUGUUGU................    3.37912
.................AACAAAGCUUGUCUAGCUUAGUCGG................................................................................................................................................................................................................    3.360212
.................AACAAAGCUUGUCUAGCUUAGUCG.................................................................................................................................................................................................................    2.46931
```

zma-MIR_N23a

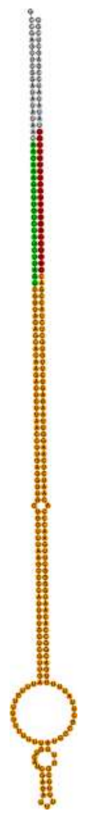

```
Chromosome:    chr10
Start:         83684805
End:           83685057
Strand:        -
Strand bias:   1.0
Abundance bias:0.921
MFE:           -193.00
```

```
                     Star                                                                                                                                                                                             Mature
GCCGAGCUCGGAUAUAUAACAACAAAGCUUGUCUAGCUUAGUCGGUAGAGCACAAGACUCUUAACCUUGAGGUUGCGGGUUUAAGCCCCAUUGUGGUCGUUGAUUUGUUUUUUUUUUGUCAUCGAGAUUUUUCGUUUAUGCCGCUUAUUUUUGACGGCCACAAUGGGGCUUGAACCCACAAUCUCAAGGUUAAGAGUCUUGUGCUCUACCGACUAAGCUAGACAAGCUUUGUUGUUAUAUAUCCGAGCUCGG   119.4
.((((((((((((((((((((((((((((((((((((((((((((((((((((((((((((((((((((((((((.(((((((((((((((((((((((((((.............((....(((((...)))))..))............))))))))))))))))))))))))))).))))))))))))))))))))))))))))))))))))))))))))))))))))))))))))))))))))))))
.....................................................................................................................................................................................................................UAAGCUAGACAAGCUUUGUUGU.................   78.03025
....................AACAAAGCUUGUCUAGCUUAGU..................................................................................................................................................................................................................   11.697
......................................................................................................................................................................................................................AAGCUAGACAAGCUUUGUUGU.................   8.05791
......................................................................................................................................................................................................................AAGCUAGACAAGCUUUGUUGUU................   3.04278
....................................................................................................................................................................................................................CUAAGCUAGACAAGCUUUGUUGU.................   3.37912
....................AACAAAGCUUGUCUAGCUUAGUCGG...............................................................................................................................................................................................................   3.360212
....................AACAAAGCUUGUCUAGCUUAGUCG................................................................................................................................................................................................................   2.46931
```

zma-MIR_N121a

Chromosome: chr4
Start: 200045011
End: 200045151
Strand: -
Strand bias: 1.0
Abundance bias: 0.998
MFE: -60.70

```
                                    Mature                                                                    Star
UGUGUACGCUAUAAGGGCUAGUUUGGGAGCCACAAAACCGGAGGAGGUUAGAGGAGCUAAAAUCUCAUCUUUAUUCAAUUUUGAAUAAGAAGGAAAUUUUAGCCCCUCUAAUCUCCUCCGAUUUUUAGAUGUAGUUGCGCG    5951.9
.(((((.((((((..((((........))))..((((.((((((((((((((((.((((((((.((.((((.(((((....)))))))))..)).)))))))).)))))))))))))))).))))....))))))))))).
....................................ACCGGAGGAGGUUAGAGGAGC....................................................................................    4297.950543
.......................................................................................................CCCUCUAAUCUCCUCCGAUUU.................    218.771446
.................................AAAACCGGAGGAGGUUAGAGG.......................................................................................    0.621726
....................................ACCGGAGGAGGUUAGAGG.......................................................................................    13.75889
....................................ACCGGAGGAGGUUAGAGGA......................................................................................    1.69751
....................................ACCGGAGGAGGUUAGAGGAG.....................................................................................    20.812157
....................................ACCGGAGGAGGUUAGAGGAGCA...................................................................................    1.776756
....................................ACCGGAGGAGGUUAGAGGAGA....................................................................................    1.479654
....................................ACCGGAGGAGGUUAGAGGAGU....................................................................................    68.870341
....................................ACCGGAGGGGGUUAGAGGAGC....................................................................................    0.605866
....................................ACCGGAGGAGGUUAGAGGCGC....................................................................................    1.59436
....................................ACCUGAGGAGGUUAGAGGAGC....................................................................................    5.06285
....................................AUCGGAGGAGGUUAGAGGAGC....................................................................................    6.96277
.....................................CCGGAGGAGGUUAGAGGAGCU...................................................................................    585.049
.....................................CCGGAGGAGGUUAGAGGAGCUA..................................................................................    73.729293
.....................................CCGGAGGAGGUUAGAGGAG.....................................................................................    37.655817
.....................................CCGGAGGAGGUUAGAGGA......................................................................................    15.83038
.....................................CCGGAGGAGGUUAGAGGAGC....................................................................................    75.512431
......................................CGGAGGAGGUUAGAGGAGC....................................................................................    0.558717
......................................CGGAGGAGGUUAGAGGAGCU...................................................................................    40.487622
......................................CGGAGGAGGUUAGAGGAGCUA..................................................................................    302.11679
......................................CGGAGGAGGUUAGAGGAG.....................................................................................    5.70789
.......................................GGAGGAGGUUAGAGGAGCUAA.................................................................................    18.340246
.......................................GGAGGAGGUUAGAGGAGCUA..................................................................................    2.003708
.......................................GGAGGAGGUUAGAGGAGCU...................................................................................    1.199053
....................................................................................................AGCCCCUCUAAUCUCCUCCGA....................    101.456689
.....................................................................................................GCCCCUCUAAUCUCCUCCGAU...................    28.964446
.......................................................................................................CCCUCUAAUCUCCUCCGAUU..................    10.090784
........................................................................................................CCUCUAAUCUCCUCCGAUUU.................    1.035669
```

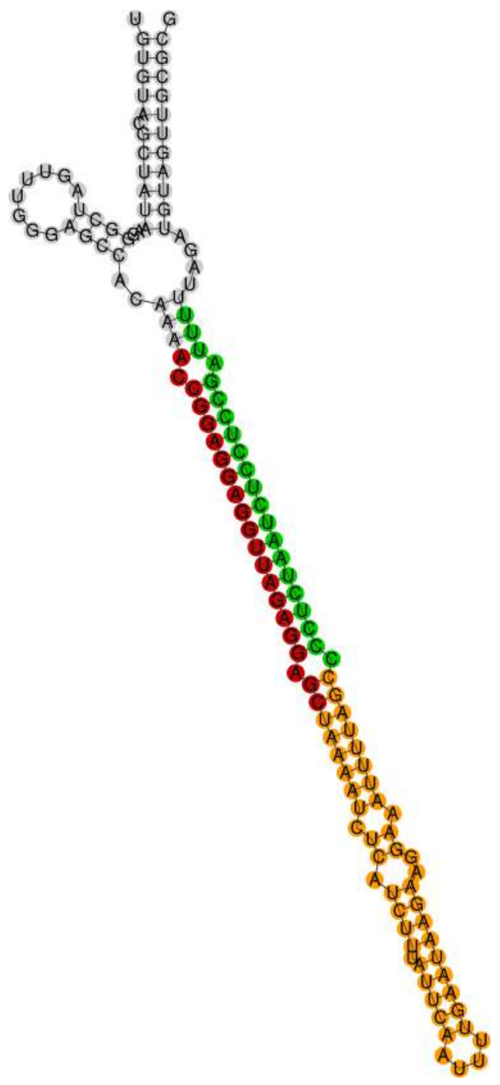

zma-MIR_N124a

Chromosome: chr4
Start: 236485062
End: 236485241
Strand: -
Strand bias: 1.0
Abundance bias: 0.999
MFE: -71.80

```
                    Star                                                                                                                    Mature
UGGUCCAUGAACUUUAAAAAUGCAUUUUUAGGUCCUUGAACUUGUUAAAUGAUGUAUCACAGAUCCCUAAACUUGAAAAAAUGGUGCAUCACAGGUCUUUUUUUAGACCAUGCCAAUCACUGUUAUGUGCCACUUAACAAAUUCAAGGGCCUAAAAAUGUAUUUUCAAAGUCCGUGGAUC   231.4
.((((((((.(((((.((((((((((((((((((((((((.(((((((.((((((((((......................))))))))))..(((((......)))))........(((......)))....))))))).)))))))))))))))))))))))).))))).))))))))
.............................................................................................................................................UCAAGGGCCUAAAAAUGUAUU..................   89.417753
....................UGCAUUUUUAGGUCCUUGAAC...........................................................................................................................................   8.311455
............................................................................................................................................UUCAAGGGCCUAAAAAUGUAU...................   17.39747
.....................GCAUUUUUAGGUCCUUGAACU..........................................................................................................................................   17.341668
....................UGCAUUUAUAGGUCCUUGAAC...........................................................................................................................................   0.279384
...................AUGCAUUUUUAGGUCCUUGAA............................................................................................................................................   5.538366
..................AAUGCAUUUUUAGGUCCUUGA.............................................................................................................................................   15.128088
```

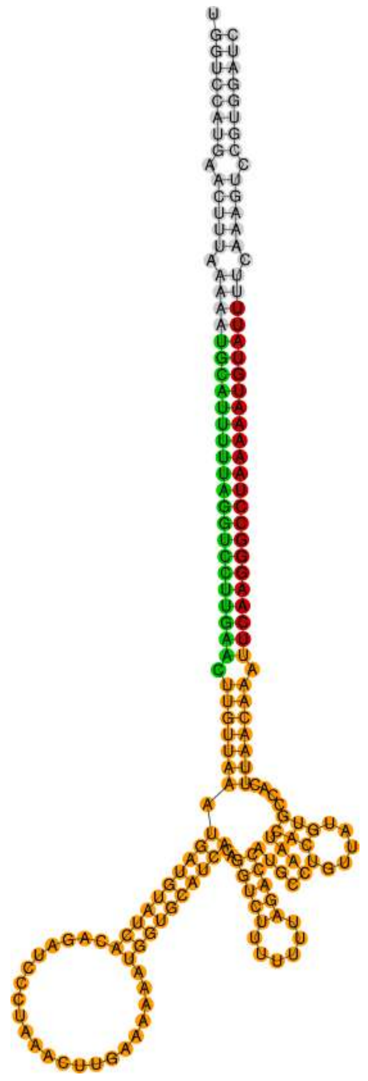

zma-MIR_N129a

Chromosome: chr5
Start: 22201754
End: 22201956
Strand: +
Strand bias: 1.0
Abundance bias: 0.931
MFE: -110.60

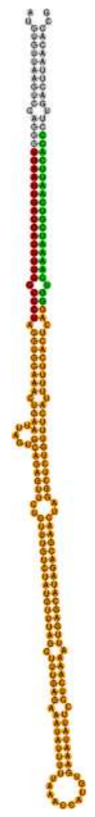

Mature Star

```
AUGUGUUAAGUCCAGGGUGAAUUGACGAUUUUGCCCCUAGGUGGAAACUGAAUUAGUGCAGAGUUCUGUUUGUCUAUGUUUAGCUUUGAGAAAUAUUUUUAAACCAUGUGAAAUAUUCCUCAAAAUUGAGCAUAGACGAACUAGGCUCUGUUUCAUUUUUCACUCAGGGUCAAAAUCGUCAAUUCACCCUUGACUUAACACCG    222.3
..(((((((((.(((((((((((((((((((.((((.((((((((.((((.....((((((((..(((((((((((((((.((((((.((((((((.........)))))))).)))))).))))))))))))))))..)))))))))))).)))))))).)))).))))))))))))))))))).))))))))))..
.................UGAAUUGACGAUUUUGCCCCU.....................................................................................................................................................................    183.115679
.......................................................................................................................................................................GGUCAAAAUCGUCAAUUCACC...............    11.697834
.................UGAAUUGACGAUUUUGCCCCA.....................................................................................................................................................................    2.22954
........................................................................................................................................................................GUCAAAAUCGUCAAUUCACCCU.............    0.152391
.........................................................................................................................................................................UCAAAAUCGUCAAUUCACCCU.............    9.805657
```

zma-MIR_N22a

```
Chromosome:    chr10
Start:         37093471
End:           37093708
Strand:        +
Strand bias:   1.0
Abundance bias:0.998
MFE:           -109.70
```

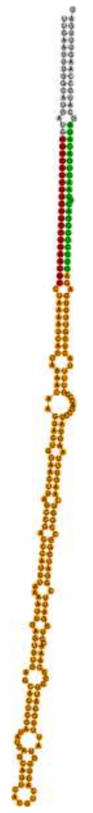

```
                 Mature                                                                                                                                                                       Star
UUGAAUUUGGCAUGAUGUGUCACUUGGUUCCUGAACUCCUAAAAUCAGGAAAACAAGUACUGGAACUUAGCCGACCCAUUUAAAAUUGAGUUUGAGCAUCCGAUAUGAGCAUUCACGUCAGUGGUAUUUGGAUGGGCCAAACCUGGAUUCGGAUGGGCCGGUCAAGUUUAAGUACCCGACCCGUUUUACUGGUUUUGAGAGUUCGGGGACGCAAGUGACAUAGCAUGCCAAGUUUGAG 254.5
(((((((((((((.((((((((((((((((((((((.(((((((((..((((..(((((.((((((.((((.(((((((..((((.((((((..(((((((....(.((((......)))).)..)))))))...)))).)).)))).))))))).)))).)))))).))))).......))))..)))))))))).))))))))))))).))))))))))).))))))))))))))).
.................UGUCACUUGGUUCCUGAACUC........................................................................................................................................................................................................ 228.517289
........................................................................................................................................................................................................GUUCGGGGACGCAAGUGACAUA................ 25.274491
..................GUCACUUGGUUCCUGAACUC........................................................................................................................................................................................................ 0.318481
```

zma-MIR_N22b

Chromosome:	chr10
Start:	93020181
End:	93020400
Strand:	+
Strand bias:	1.0
Abundance bias:	0.998
MFE:	-108.70

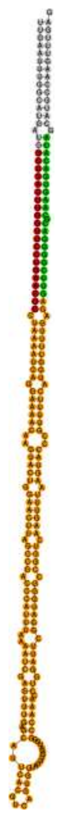

Mature Star

```
UUGAAUUUGGCAUGAUGUGUCACUUGGUUCCUGAACUCCUAAAAUCAGGAAAACAAGUACUGGAACUUAGCCGACCCAUUUAAAAUCGAGUUUGAGCAUUCACGUCAGUGGUAUUUGGAUGGGCCAAACCUGGAUUCGGAUGGGCCGGUCAAGUUUAAGUACCCGUUUUCAUGGUUUUGAGAGUUCGGGGACGCAAGUGACAUAGCAUGCCAAGUUUGAG    254.5
(((((((((((((.(((((((((((((((((((((((.((((((((.((((((..(((((.((((((.((((.(((((((..((((.((((((.((..((((....))))..........)))))).)).)))).))))))).)))).)))))).)))))..)))))).)))))))).)))))))))))).))))))))))).))))))))))))))).
.................UGUCACUUGGUUCCUGAACUC......................................................................................................................................................................................    228.517289
......................................................................................................................................................................................GUUCGGGGACGCAAGUGACAUA................    25.274491
..................GUCACUUGGUUCCUGAACUC......................................................................................................................................................................................    0.318481
```

```
                    Star                                                                                                                                    Mature
GCUAUGGUAAAUGUAUUGAUCUCUUCUACACCACUCAGAUGCAAUUUCUUAUCCUUUUAUGCAUCUUCAGUGGCACAGAAAGAAUUAGGACAUAUAAUUUUCUUAUGUUUCUAUGCCACUGACGAUGACAUGGAAAAGUAAUAAAUUGUAUCCGAAUGGUGUAGAAGGAAUUAGGAUAUUUACGAUGG   574.7
.((((.((((((((.(((((.(((((((((((.((.((((((((((.((((..((((((((((.(((((((((.(((((....(((((.(......).)))))..))))).))))))))).)))).)))))))..)))).))))))))))).)).))))))))))))).))))).)))))))).))))
......................................................................................................................................................UCCGAAUGGUGUAGAAGGAAU.................   477.409598
....................CUCUUCUACACCACUCAGAUG...................................................................................................................................................   0.583882
......................................................................................................................................................UCCGAAUGGUGUAGAAGG....................   1.23482
......................................................................................................................................................UCCGAAUGGUGUAGAAGGAA..................   16.20248
......................................................................................................................................................UCCGAAUGGUGUAGAAGGAAUU................   3.060134
......................................................................................................................................................UCCGAAUGGGGUAGAAGGAAU.................   0.182396
.....................................................................................................................................................AUCCGAAUGGUGUAGAAGGAAUUA................   0.427815
```

zma-MIR_N130a

```
Chromosome:    chr5
Start:         22412122
End:           22412309
Strand:        -
Strand bias:   1.0
Abundance bias:0.868
MFE:           -81.30
```

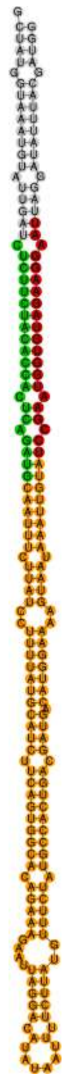

zma-MIR_N21b

Chromosome: chr10
Start: 149277471
End: 149277649
Strand: +
Strand bias: 0.994
Abundance bias:0.926
MFE: -109.60

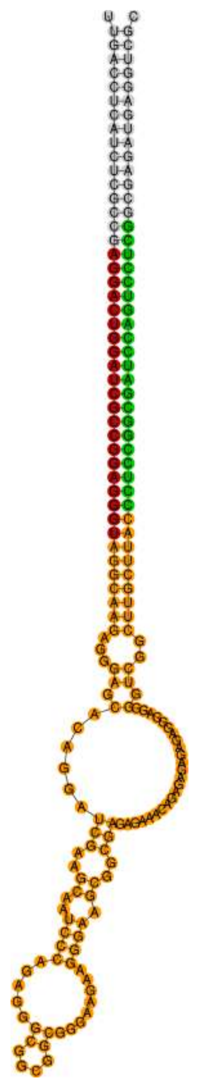

```
                        Mature                                                                                                                   Star
UUGACCUCAUCUCGCCGAGGACUGGAUCGCCGGAGGGUAGGCAAGAGGGAGCACAGGAUCGAAGCAAUCCCAGAGGGCGGCGGCGGGAAGAAGGGAAGCGGCGAGAGAAACAGAGAGAGAGAGGGAGGGGUCGGCUUGCUUACCCUCCGGCGAUCCAGUCCUCGGCGAGAUGAGGUCGC    42425.8
.((((((((((((((((((((((((((((((((((((((((((((...((.(......(((..((..((((.....((....))........)))).))..)))........................).))..)))))))))))))))))))))))))))))))))))))))))))).
.................AGGACUGGAUCGCCGGAGGGU.............................................................................................................................................    31174.25228
...............................................................................................................................................CCUCCGGCGAUCCAGUCCUCG...............    392.937842
..............CCGAGGACUGGAUCGCCGGAG................................................................................................................................................    328.971058
...............CGAGGACUGGAUCGCCGGAG................................................................................................................................................    0.182396
...............CGAGGACUGGAUCGCCGGAGG...............................................................................................................................................    124.866448
................GAGGACUGGAUCGCCGGAG................................................................................................................................................    37.63092
................GAGGACUGGAUCGCCGGAGG...............................................................................................................................................    11.92437
................GAGGACUGGAUCGCCGGAGGG..............................................................................................................................................    1161.644963
................GAGGACUGGAUCGCCGGAGGA..............................................................................................................................................    29.960603
.................AGGACUGGAUCGCCGGAGGG..............................................................................................................................................    526.705536
.................AGGACUGGAUCGCCGGAGGGUA............................................................................................................................................    33.435878
.................AGGACUGGAUCGCCGGAG................................................................................................................................................    253.240886
.................AGGACUGGAUCGCCGGAGG...............................................................................................................................................    302.343306
.................UGGACUGGAUCGCCGGAGGGU.............................................................................................................................................    10.405459
.................AGGCCUGGAUCGCCGGAGGGU.............................................................................................................................................    4.396758
.................AGGACAGGAUCGCCGGAGGGU.............................................................................................................................................    2.07492
.................AGGACUGGAUCGCCGGAA................................................................................................................................................    5.69337
.................AGGACUGGAUCGCCGGAGGAU.............................................................................................................................................    15.335697
.................AGGACUGGAUCGCCGGAGAGU.............................................................................................................................................    2.811205
.................AGGACUGGAUCGCCGGCGGGU.............................................................................................................................................    63.06515
.................AGGACUGGAUCGCCGGAGGUU.............................................................................................................................................    3.154857
.................AGGUCUGGAUCGCCGGAGGGU.............................................................................................................................................    7.08833
.................AGGACUGGAUCGACGGAGGGU.............................................................................................................................................    1.27549
.................AGGACUGGAUCGCCGGGGGGU.............................................................................................................................................    57.282965
.................AGGACUGGGUCGCCGGAGGGU.............................................................................................................................................    20.346278
.................AGGACUGGAUCGCCGGUGGGU.............................................................................................................................................    2.44225
.................AGGACCGGAUCGCCGGAGGGU.............................................................................................................................................    12.723176
.................AGGACUGGAUCGCCGGAGUGU.............................................................................................................................................    11.949515
.................AGGACUGGAUCGCCUGAGGGU.............................................................................................................................................    9.94447
.................AGGACUUGAUCGCCGGAGGGU.............................................................................................................................................    22.745755
.................AGGACUGGAUCGCCAGAGGGU.............................................................................................................................................    4.01178
.................GGGACUGGAUCGCCGGAGGGU.............................................................................................................................................    20.1455
.................AGUACUGGAUCGCCGGAGGGU.............................................................................................................................................    19.754754
.................AGGACUGGAUCGUCGGAGGGU.............................................................................................................................................    1.570363
.................AGGACUGGAUCGCCGGAUGGU.............................................................................................................................................    17.91123
.................AGGACUGGAUCGCUGGAGGGU.............................................................................................................................................    3.3581
.................AGGACUGGAUCGCCGGAGGCU.............................................................................................................................................    83.52165
.................AGGACUGGCUCGCCGGAGGGU.............................................................................................................................................    0.756944
.................AUGACUGGAUCGCCGGAGGGU.............................................................................................................................................    26.297585
.................AGGACUGGAUCACCGGAGGGU.............................................................................................................................................    7.151747
.................AGGACUGGAUCCCCGGAGGGU.............................................................................................................................................    0.182396
.................AGGACUGGAUCGCAGGAGGGU.............................................................................................................................................    7.3882
.................AGGACUGGACCGCCGGAGGGU.............................................................................................................................................    11.266456
.................AGGACUGGAUCGCCGGAAGGU.............................................................................................................................................    1.11605
.................AGGACUGGAACGCCGGAGGGU.............................................................................................................................................    2.349765
.................AGGACUGGAUCGCCGGAGGGUG............................................................................................................................................    1.27549
.................AGGACUGGAUCGCCGGAGGGUC............................................................................................................................................    1.83169
.................AGGACUGGAUCGCCGGAGGGUU............................................................................................................................................    55.434532
.................AGGACUGGAUCGCCGGAGGA..............................................................................................................................................    3.944302
.................AGGACUGGAUCGCCGGAGGU..............................................................................................................................................    1.01167
.................AGGACUGGAUCGCCGGAU................................................................................................................................................    0.243195
.................CGGACUGGAUCGCCGGAGGGU.............................................................................................................................................    6.65835
.................AGGACUGGAUCGCCGGAGGGA.............................................................................................................................................    594.0461
.................AGGACUGGAUCGCCGGAGGGC.............................................................................................................................................    2445.880786
.................AGGACUGGAUCGCCGGAGGGG.............................................................................................................................................    632.532622
.................AGGGCUGGAUCGCCGGAGGGU.............................................................................................................................................    21.574496
.................AGGACUGAAUCGCCGGAGGGU.............................................................................................................................................    3.84925
.................AGGACUGUAUCGCCGGAGGGU.............................................................................................................................................    10.651315
.................AGGACUGGAUCUCCGGAGGGU.............................................................................................................................................    2.44225
.................AGGACUGGAUUGCCGGAGGGU.............................................................................................................................................    20.406458
.................AGGACUGGAUAGCCGGAGGGU.............................................................................................................................................    11.40427
.................AGGACUGGAUCGCCGUAGGGU.............................................................................................................................................    10.20742
.................AGGACUGGAUCGCCGGAGA...............................................................................................................................................    1.03139
..................GGACUGGAUCGCCGGAGGG..............................................................................................................................................    15.3671
..................GGACUGGAUCGCCGGAGGGU.............................................................................................................................................    19.44848
..................GGACUGGAUCGCCGGAGGGUA............................................................................................................................................    73.284754
..................GGACUGGAUCGCCGGAGG...............................................................................................................................................    9.99967
..................GGACUGGAUCGCCGGAGGGUU............................................................................................................................................    1.43492
...................GACUGGAUCGCCGGAGGGU.............................................................................................................................................    100.771777
...................GACUGGAUCGCCGGAGGG..............................................................................................................................................    0.558768
...................GACUGGAUCGCCGGAGGGUA............................................................................................................................................    32.607595
............................................................................................................................................UACCCUCCGGCGAUCCAGUCC..................    133.172441
.............................................................................................................................................ACCCUCCGGCGAUCCAGUCCU.................    16.613174
..............................................................................................................................................CCCUCCGGCGAUCCAGUCCUC................    4.338255
...............................................................................................................................................CCUCCGGCGAUCCAGUCCUC................    40.501837
...............................................................................................................................................CCUCCGGCGAUCCAGUCCU.................    24.139018
...............................................................................................................................................CCUCCGGCGAUCCAGUCCUCU...............    1.971151
...............................................................................................................................................CCUCCGGCGAUCCAGUCCUCA...............    24.61424
................................................................................................................................................CUCCGGCGAUCCAGUCCUCG...............    3.23393
................................................................................................................................................CUCCGGCGAUCCAGUCCUCGG..............    25.930033
.................................................................................................................................................UCCGGCGAUCCAGUCCUCGGC.............    84.635194
..................................................................................................................................................CCGGCGAUCCAGUCCUCGGC.............    0.75184
..................................................................................................................................................CCGGCGAUCCAGUCCUCGGCG............    4.441972
```

zma-MIR_N21a

Chromosome:	chr10
Start:	149277469
End:	149277651
Strand:	-
Strand bias:	1.0
Abundance bias:	0.977
MFE:	-100.60

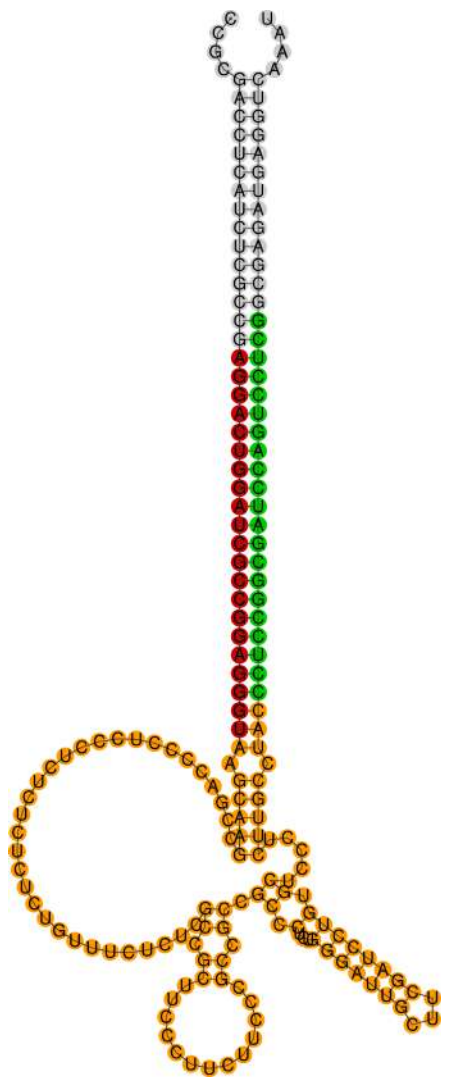

```
                                    Mature                                                                                                                                          Star
CCGCGACCUCAUCUCGCCGAGGACUGGAUCGCCGGAGGGUAAGCAAGCCGACCCCUCCCUCUCUCUCUCUGUUUCUCUCGCCGCUUCCCUUCUUCCCGCCGCCGCCCUCUGGGAUUGCUUCGAUCCUGUGCUCCCUCUUGCCUACCCUCCGGCGAUCCAGUCCUCGGCGAGAUGAGGUCAAAU   40314.6
....((((((((((((((((((((((((((((((((((((.(((((.................................((.((.............)).)).((.(...((((((((...)))))))).)).....))))).))))))))))))))))))))))))))))))))))))....
...................AGGACUGGAUCGCCGGAGGGU...............................................................................................................................................   31174.25228
.................................................................................................................................................CCUCCGGCGAUCCAGUCCUCG.................   392.937842
................CCGAGGACUGGAUCGCCGGAG..................................................................................................................................................   328.971058
.................CGAGGACUGGAUCGCCGGAG..................................................................................................................................................   0.182396
.................CGAGGACUGGAUCGCCGGAGG.................................................................................................................................................   124.866448
..................GAGGACUGGAUCGCCGGAG..................................................................................................................................................   37.63092
..................GAGGACUGGAUCGCCGGAGG.................................................................................................................................................   11.92437
..................GAGGACUGGAUCGCCGGAGGG................................................................................................................................................   1161.644963
..................GAGGACUGGAUCGCCGGAGGA................................................................................................................................................   29.960603
...................AGGACUGGAUCGCCGGAGGG................................................................................................................................................   526.705536
...................AGGACUGGAUCGCCGGAGGGUA..............................................................................................................................................   33.435878
...................AGGACUGGAUCGCCGGAGGGUAAG............................................................................................................................................   25.459967
...................AGGACUGGAUCGCCGGAG..................................................................................................................................................   253.240886
...................AGGACUGGAUCGCCGGAGG.................................................................................................................................................   302.343306
...................UGGACUGGAUCGCCGGAGGGU...............................................................................................................................................   10.405459
...................AGGCCUGGAUCGCCGGAGGGU...............................................................................................................................................   4.396758
...................AGGACAGGAUCGCCGGAGGGU...............................................................................................................................................   2.07492
...................AGGACUGGAUCGCCGGAA..................................................................................................................................................   5.69337
...................AGGACUGGAUCGCCGGAGGAU...............................................................................................................................................   15.335697
...................AGGACUGGAUCGCCGGAGAGU...............................................................................................................................................   2.811205
...................AGGACUGGAUCGCCGGCGGGU...............................................................................................................................................   63.06515
...................AGGACUGGAUCGCCGGAGGUU...............................................................................................................................................   3.154857
...................AGGUCUGGAUCGCCGGAGGGU...............................................................................................................................................   7.08833
...................AGGACUGGAUCGACGGAGGGU...............................................................................................................................................   1.27549
...................AGGACUGGAUCGCCGGGGGGU...............................................................................................................................................   57.282965
...................AGGACUGGGUCGCCGGAGGGU...............................................................................................................................................   20.346278
...................AGGACUGGAUCGCCGGUGGGU...............................................................................................................................................   2.44225
...................AGGACCGGAUCGCCGGAGGGU...............................................................................................................................................   12.723176
...................AGGACUGGAUCGCCGGAGUGU...............................................................................................................................................   11.949515
...................AGGACUGGAUCGCCUGAGGGU...............................................................................................................................................   9.94447
...................AGGACUUGAUCGCCGGAGGGU...............................................................................................................................................   22.745755
...................AGGACUGGAUCGCCAGAGGGU...............................................................................................................................................   4.01178
...................GGGACUGGAUCGCCGGAGGGU...............................................................................................................................................   20.1455
...................AGUACUGGAUCGCCGGAGGGU...............................................................................................................................................   19.754754
...................AGGACUGGAUCGUCGGAGGGU...............................................................................................................................................   1.570363
...................AGGACUGGAUCGCCGGAUGGU...............................................................................................................................................   17.91123
...................AGGACUGGAUCGCUGGAGGGU...............................................................................................................................................   3.3581
...................AGGACUGGAUCGCCGGAGGCU...............................................................................................................................................   83.52165
...................AGGACUGGCUCGCCGGAGGGU...............................................................................................................................................   0.756944
...................AUGACUGGAUCGCCGGAGGGU...............................................................................................................................................   26.297585
...................AGGACUGGAUCACCGGAGGGU...............................................................................................................................................   7.151747
...................AGGACUGGAUCCCCGGAGGGU...............................................................................................................................................   0.182396
...................AGGACUGGAUCGCAGGAGGGU...............................................................................................................................................   7.3882
...................AGGACUGGACCGCCGGAGGGU...............................................................................................................................................   11.266456
...................AGGACUGGAUCGCCGGAAGGU...............................................................................................................................................   1.11605
...................AGGACUGGAACGCCGGAGGGU...............................................................................................................................................   2.349765
...................AGGACUGGAUCGCCGGAGGGUG..............................................................................................................................................   1.27549
...................AGGACUGGAUCGCCGGAGGGUC..............................................................................................................................................   1.83169
...................AGGACUGGAUCGCCGGAGGGUU..............................................................................................................................................   55.434532
...................AGGACUGGAUCGCCGGAGGA................................................................................................................................................   3.944302
...................AGGACUGGAUCGCCGGAGGU................................................................................................................................................   1.01167
...................AGGACUGGAUCGCCGGAU..................................................................................................................................................   0.243195
...................CGGACUGGAUCGCCGGAGGGU...............................................................................................................................................   6.65835
...................AGGACUGGAUCGCCGGAGGGA...............................................................................................................................................   594.0461
...................AGGACUGGAUCGCCGGAGGGC...............................................................................................................................................   2445.880786
...................AGGACUGGAUCGCCGGAGGGG...............................................................................................................................................   632.532622
...................AGGGCUGGAUCGCCGGAGGGU...............................................................................................................................................   21.574496
...................AGGACUGAAUCGCCGGAGGGU...............................................................................................................................................   3.84925
...................AGGACUGUAUCGCCGGAGGGU...............................................................................................................................................   10.651315
...................AGGACUGGAUCUCCGGAGGGU...............................................................................................................................................   2.44225
...................AGGACUGGAUUGCCGGAGGGU...............................................................................................................................................   20.406458
...................AGGACUGGAUAGCCGGAGGGU...............................................................................................................................................   11.40427
...................AGGACUGGAUCGCCGUAGGGU...............................................................................................................................................   10.20742
...................AGGACUGGAUCGCCGGAGA.................................................................................................................................................   1.03139
....................GGACUGGAUCGCCGGAGGG................................................................................................................................................   15.3671
....................GGACUGGAUCGCCGGAGGGU...............................................................................................................................................   19.44848
....................GGACUGGAUCGCCGGAGGGUA..............................................................................................................................................   73.284754
....................GGACUGGAUCGCCGGAGG.................................................................................................................................................   9.99967
....................GGACUGGAUCGCCGGAGGGUU..............................................................................................................................................   1.43492
.....................GACUGGAUCGCCGGAGGGUAA.............................................................................................................................................   72.098061
.....................GACUGGAUCGCCGGAGGGU...............................................................................................................................................   100.771777
.....................GACUGGAUCGCCGGAGGG................................................................................................................................................   0.558768
.....................GACUGGAUCGCCGGAGGGUA..............................................................................................................................................   32.607595
.....................GACUGGAUCGCCGGAGGGGAA.............................................................................................................................................   0.243195
......................ACUGGAUCGCCGGAGGGUAAG............................................................................................................................................   16.219043
..............................................................................................................................................UACCCUCCGGCGAUCCAGUCC....................   133.172441
...............................................................................................................................................ACCCUCCGGCGAUCCAGUCCU...................   16.613174
................................................................................................................................................CCCUCCGGCGAUCCAGUCCUC..................   4.338255
.................................................................................................................................................CCUCCGGCGAUCCAGUCCUC..................   40.501837
.................................................................................................................................................CCUCCGGCGAUCCAGUCCU...................   24.139018
.................................................................................................................................................CCUCCGGCGAUCCAGUCCUCU.................   1.971151
.................................................................................................................................................CCUCCGGCGAUCCAGUCCUCA.................   24.61424
..................................................................................................................................................CUCCGGCGAUCCAGUCCUCG.................   3.23393
..................................................................................................................................................CUCCGGCGAUCCAGUCCUCGG................   25.930033
...................................................................................................................................................UCCGGCGAUCCAGUCCUCGGC...............   84.635194
....................................................................................................................................................CCGGCGAUCCAGUCCUCGGC...............   0.75184
....................................................................................................................................................CCGGCGAUCCAGUCCUCGGCG..............   4.441972
```

zma-MIR2275_N4

Chromosome: chr6
Start: 6798198
End: 6798296
Strand: +
Strand bias: 1.0
Abundance bias: 0.995
MFE: -40.60

```
                Star                                         Mature
UUUGGCAACCGAAUACGAGAAUUGGAUGGAACCAAAUCUGACGGCAAGAGCAAGCCAAGACUUGGUUUCCUCCAAUUUCUCAUGUUCAGUUGAUCAGAU    612.1
(((((((((.(((((.((((((((((.((((((((.(((...(((........))).))).)))))))).))))).))))).))))).)))).))))).
............................................................CUUGGUUUCCUCCAAUUUCUCA.................    392.460843
.................AGAAUUGGAUGGAACCAAAUC..............................................................    164.86206
............................................................CUUGGUUUCCUCCAAUUUCUC..................    25.38622
............................................................CUUGGUUUCCUCCAAUUUC....................    0.427815
............................................................CUUGGUUUCCUCCAAUUUCU...................    3.298552
............................................................CUUGGUUUCCUCCAAUUUCUCAU................    0.203188
............................................................CUUGGGUUCCUCCAAUUUCUCA.................    0.609565
............................................................CUUGGUUUCCUCCAAUUUCCCA.................    0.228587
............................................................CUUGGUUUCCUCCAAUUUCUCU.................    4.02928
............................................................CUUGGUUUCCUCCAAUUUCUAA.................    0.152391
.................AGAAUUGGAUGGAACCAAAUCU.............................................................    2.69225
.................AGAAUUGGAUGGAACCAAA...............................................................    0.406377
.................AGAAUUGGAUGGAACCAAAU..............................................................    13.09354
.................AGAAUUGGAUGGAACCAAAUCA.............................................................    0.431775
.................AGAAUUGGAUGGAACCAAAUU..............................................................    1.062779
```

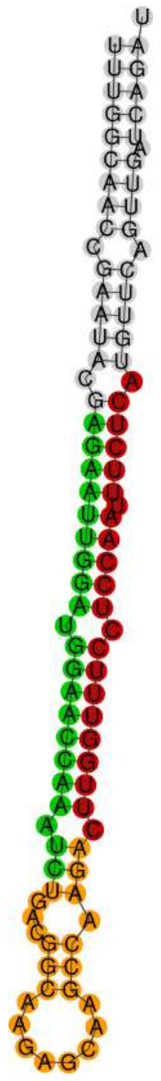

zma-MIR_N146a

Chromosome: chr6
Start: 7404497
End: 7404608
Strand: +
Strand bias: 1.0
Abundance bias: 0.872
MFE: -57.50

```
                 Mature                                                    Star
CAACUCCUUGUUUAAUUUGGCACGACAAAAAACUCGCACCGGGUGCUCUAAUCGGCGGUCGAUUACAGCGUCCAGUGCGAGUUCUUUGCCGUGCCAAAAUUGAAAGGAGGUC    1225.2
...((((((..(((((((((((((.((((.(((((((((.(((((((.((((((.....)))))).))))))).))))))))).)))).)))))).)))))))))))))...
.................UGGCACGACAAAAAACUCGCA..........................................................................    784.720345
.............................................................................CGAGUUCUUUGCCGUGCCAAAA.............    2.69488
...............UUUGGCACGACAAAAAACUCG............................................................................    1.367902
...............UUUGGCACGACAAAAAACUCGCAC.........................................................................    2.730105
................UUGGCACGACAAAAAACUCGCACC........................................................................    0.636962
.................UGGCACGACAAAAAACUCGC...........................................................................    8.254351
.................UGGCACGACAACAAACUCGCA..........................................................................    1.40644
.................UGGCACGACAAAAAGCUCGCA..........................................................................    4.804584
.................UGGCACGACAAACAACUCGCA..........................................................................    0.883101
.................UGGCACGACAAAACACUCGCA..........................................................................    1.13542
..................GGCACGACAAAAAACUCGCA..........................................................................    58.115113
..................GGCACGACAAAAAACUCGCACC........................................................................    0.424642
..................GGCACGACAAAAAACUCGCAC.........................................................................    141.731584
..................GGCACGACAAAAAACUCGC...........................................................................    1.00222
..................GGCACGACAAAACACUCGCAC.........................................................................    1.17204
...........................................................................UGCGAGUUCUUUGCCGUGCCAAAA.............    2.55977
............................................................................GCGAGUUCUUUGCCGUGCCA................    6.34627
............................................................................GCGAGUUCUUUGCCGUGCCAA...............    13.501026
.............................................................................CGAGUUCUUUGCCGUGCCAAA..............    34.317092
```

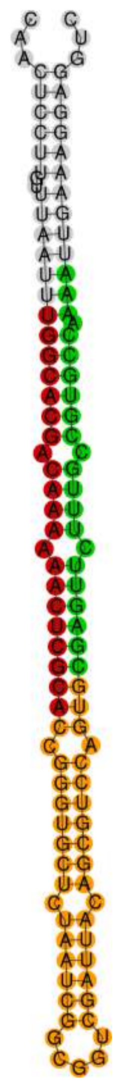

zma-MIR_N148a

Chromosome: chr6
Start: 91184726
End: 91184905
Strand: +
Strand bias: 1.0
Abundance bias: 0.998
MFE: -85.20

```
                  Mature                                                                                                      Star
GUGCACAUAUGCACAUAUUAAAUCUGGACCCUUCAUCUAAUAAUCAGCGGCUGAACUUGCACCUGAGUGUGGGGGAGUGGAGUGCCAAAGGGUUUUUCUGUAAAUAUACCAGCCAUUGAUUGUUGGACGAAGGGUCUAGAUUUAAUAUGUGCAUAUAUACACGGAUGGGUCAUGUGCAUA    1192.9
((((((((((((((((((((((((((((((((((.(((((((((((.(((((.((((.(.((.......))).))))((((.(((....))).))))...........))))).)))))))))))).)))))))))))))))))))))))))))................))))))).
.................UUAAAUCUGGACCCUUCAUCU.............................................................................................................................................    1024.390187
..............................................................................................................................ACGAAGGGUCUAGAUUUAAUA.................................    13.404433
..............AUAUUAAAUCUGGACCCUUCAUCU.............................................................................................................................................    0.371561
.................UUAAAUCUGGACCCUUCAUC..............................................................................................................................................    105.089343
.................UUAAAUCUGGACCCUUCAU...............................................................................................................................................    0.228587
.................UUAAAUCUGGACCCUUCU................................................................................................................................................    0.203188
.................UUAAAUCUGGACCCUUCAUCA.............................................................................................................................................    9.9873
.................UUAAAUCUGGACCCUUCAUCC.............................................................................................................................................    17.6514
.................UUAAAUCUGGACCCUUCAUCUU............................................................................................................................................    0.253986
..................UAAAUCUGGACCCUUCAUCUA............................................................................................................................................    8.35705
...................AAAUCUGGACCCUUCAUCU.............................................................................................................................................    7.022994
..............................................................................................................................ACGAAGGGUCUAGAUUUAAU..................................    3.7292
```

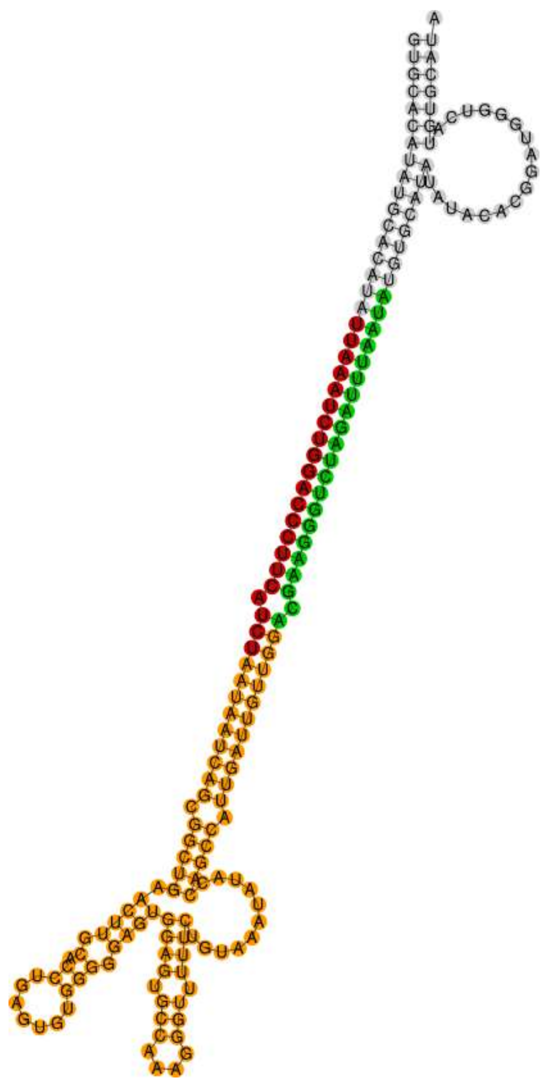

zma-MIR_N24a

Chromosome: chr7
Start: 170347732
End: 170347862
Strand: +
Strand bias: 0.951
Abundance bias: 0.958
MFE: -129.60

```
                 Mature                                                                        Star
GGGCUUGUUCGUUUUGGAGUGGAUUGAAGGGGGAUUGGAGGGGAUUAAAUCCCCUCCUAUACAAAUUUGUAUAGGAGGGGAUUUAAUCCCCUCCAAUCCCCCUUCAAUCCACUCCAAAACGAACAAGCCCU   662.
(((((((((((((((((((((((((((((((((((((((((((((((((((((((((((((((....))))))))))))))))))))))))))))))))))))))))))))))))))))))))))))))).
.................AGUGGAUUGAAGGGGGAUUGG.............................................................................................   144.441876
..............................................................................................AAUCCCCCUUCAAUCCACUCC................   23.050547
..............UGGAGUGGAUUGAAGGGGGAU................................................................................................   0.408264
...............GGAGUGGAUUGAAGGGGGAUUGG.............................................................................................   1.649277
...............GGAGUGGAUUGAAGGGGGAUU...............................................................................................   0.306198
...............GGAGUGGAUUGAAGGGGGAUUGGA............................................................................................   39.734658
................GAGUGGAUUGAAGGGGGAUUG..............................................................................................   2.804869
................GAGUGGAUUGAAGGGGGAUUGGAG...........................................................................................   11.55648
................GAGUGGAUUGAAGGGGGAUUGGA............................................................................................   0.916389
.................AGUGGAUUGAAGGGGGAUU...............................................................................................   10.25541
.................AGUGGAUUGAAGGGGGAUUGGAGG..........................................................................................   13.550062
.................AGUGGAUUGAAGGGGGAU................................................................................................   1.55331
.................AGUGGAUUGAAGGGGGAUUGGA............................................................................................   55.581969
.................AGUGGAUUGAAGGGGGAUUGGAG...........................................................................................   0.182396
..................GUGGAUUGAAGGGGGAUUGGAG...........................................................................................   4.027196
..................GUGGAUUGAAGGGGGAUUGGAGG..........................................................................................   0.600426
..................GUGGAUUGAAGGGGGAUUGGA............................................................................................   39.36907
...................UGGAUUGAAGGGGGAUUGGAGG..........................................................................................   0.306198
...................UGGAUUGAAGGGGGAUUGGAG...........................................................................................   18.372805
....................GGAUUGAAGGGGGAUUGGAGG..........................................................................................   29.578719
...........................................................................................UCCAAUCCCCCUUCAAUCCAC...................   16.9877
.............................................................................................CAAUCCCCCUUCAAUCCACUC.................   0.636962
..............................................................................................AAUCCCCCUUCAAUCCACUCCAAA.............   1.289627
...............................................................................................AUCCCCCUUCAAUCCACUCCA...............   1.67216
```

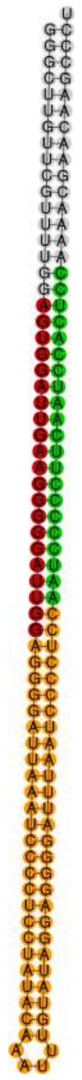

zma-MIR_N24b

Chromosome: chr7
Start: 170347733
End: 170347861
Strand: -
Strand bias: 0.951
Abundance bias: 0.958
MFE: -125.10

```
                 Mature                                                                           Star
GGGCUUGUUCGUUUUGGAGUGGAUUGAAGGGGGAUUGGAGGGGAUUAAAUCCCCUCCUAUACAAAUUUGUAUAGGAGGGGAUUUAAUCCCCUCCAAUCCCCCUUCAAUCCACUCCAAAACGAACAAGCC    662.
.((((((((((((((((((((((((((((((((((((((((((((((((((((((((((((((....))))))))))))))))))))))))))))))))))))))))))))))))))))))))))))))
.................AGUGGAUUGAAGGGGGAUUGG...........................................................................................    144.441876
..............................................................................................AAUCCCCCUUCAAUCCACUCC..............    23.050547
..............UGGAGUGGAUUGAAGGGGGAU..............................................................................................    0.408264
...............GGAGUGGAUUGAAGGGGGAUUGG...........................................................................................    1.649277
...............GGAGUGGAUUGAAGGGGGAUU.............................................................................................    0.306198
...............GGAGUGGAUUGAAGGGGGAUUGGA..........................................................................................    39.734658
................GAGUGGAUUGAAGGGGGAUUG............................................................................................    2.804869
................GAGUGGAUUGAAGGGGGAUUGGAG.........................................................................................    11.55648
................GAGUGGAUUGAAGGGGGAUUGGA..........................................................................................    0.916389
.................AGUGGAUUGAAGGGGGAUU.............................................................................................    10.25541
.................AGUGGAUUGAAGGGGGAUUGGAGG........................................................................................    13.550062
.................AGUGGAUUGAAGGGGGAU..............................................................................................    1.55331
.................AGUGGAUUGAAGGGGGAUUGGA..........................................................................................    55.581969
.................AGUGGAUUGAAGGGGGAUUGGAG.........................................................................................    0.182396
..................GUGGAUUGAAGGGGGAUUGGAG.........................................................................................    4.027196
..................GUGGAUUGAAGGGGGAUUGGAGG........................................................................................    0.600426
..................GUGGAUUGAAGGGGGAUUGGA..........................................................................................    39.36907
...................UGGAUUGAAGGGGGAUUGGAGG........................................................................................    0.306198
...................UGGAUUGAAGGGGGAUUGGAG.........................................................................................    18.372805
....................GGAUUGAAGGGGGAUUGGAGG........................................................................................    29.578719
...........................................................................................UCCAAUCCCCCUUCAAUCCAC.................    16.9877
.............................................................................................CAAUCCCCCUUCAAUCCACUC...............    0.636962
..............................................................................................AAUCCCCCUUCAAUCCACUCCAAA...........    1.289627
...............................................................................................AUCCCCCUUCAAUCCACUCCA.............    1.67216
```

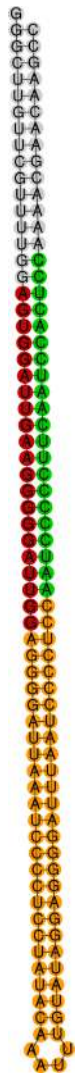

```
                 Mature                                               Star
UGUUCUGUGCUAUAAAUAAAUCCUAUAAAUUUACCAGAGUGUUCUGUAGACAUGUUCAAUUUAUUGAACACUCUUGUAAAUUUAUGGGAUUUAAAUUUAUGGGAUUUAUUAACAACACAUUACAA    195.3
(((..(((((((((((((((((((((((((((((.(((((((((.(((((.........))))).))))))))).))))))))))))))))))..))))))).............))))..))).
.................AAAUCCUAUAAAUUUACCAGA.......................................................................................    174.25124
.........................................................................UUGUAAAUUUAUGGGAUUUAA...............................    16.380027
..................AAUCCUAUAAAUUUACCAGA.......................................................................................    4.716427
```

zma-MIR_N154a

```
Chromosome:    chr6
Start:         145096611
End:           145096735
Strand:        +
Strand bias:   1.0
Abundance bias:1.0
MFE:           -43.60
```

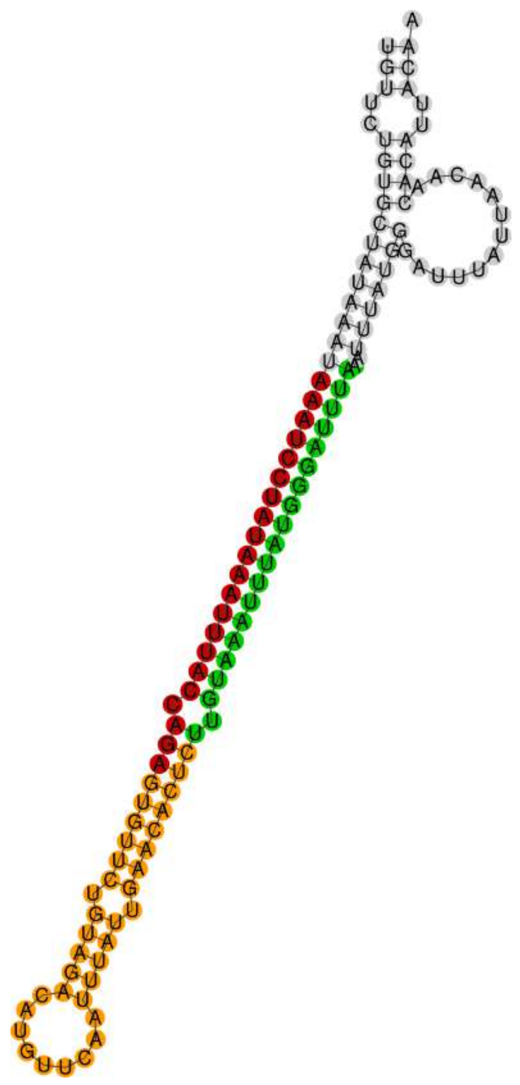

zma-MIR_N147a

Chromosome: chr6
Start: 36081889
End: 36082084
Strand: -
Strand bias: 0.974
Abundance bias: 0.805
MFE: -149.10

```
                   Star                                                                                                                                             Mature
GCCGUCGGCCUUAUAAAUGGAUUGUAACCGGACCUUUGAAGCCAAGCAAUAAUCUCUCACCUUUCUACCUCUGUUAAGUCUACGGUUGGGAGGCAACCGGAGAUUAUGCCUCCCAACCGCAGGCUUAACAGAGGUAGAAAGAAGAUUAUUGCUUGCCUUCAAAGGUCCGGUUACAAUCCAUUUAUAAGGCCUGCGG 1456.9
.((((.(((((((((((((((((((((((((((((((((((.(((((((((((((.....(((((((((((((((((((((.(((((((((((((...........))))))))))))).))))))))))))))))))))).))))))))))))).))))))))))))))))))))))))))))))))))).))))
..............................................................................................................................................................UCAAAGGUCCGGUUACAAUCC................. 501.555343
....................AUUGUAACCGGACCUUUGAAG........................................................................................................................................................... 237.772383
..............................................................................................................................................................UCAAAGGUCCGGUUACAAUC.................. 1.32118
.............................................................................................................................................................UUCAAAGGUCCGGUUACAAUC.................. 11.94056
.......................GUAACCGGACCUUUGAAGCC......................................................................................................................................................... 0.68091
......................UGUAACCGGACCUUUGAAGCC......................................................................................................................................................... 2.179437
.....................UUGUAACCGGACCUUUGAAGCC......................................................................................................................................................... 1.597228
.....................UUGUAACCGGACCUUUGAAGC.......................................................................................................................................................... 120.926156
.....................UUGUAACCGAACCUUUGAAG........................................................................................................................................................... 2.681514
.....................UUGUAACCGAACCUUUGAA............................................................................................................................................................ 64.51048
.....................UUGUAACCGAACCUUUGA............................................................................................................................................................. 11.08715
....................AUUGUAACCGGACCUUUGAAGC.......................................................................................................................................................... 5.251402
....................AUUGUAACCGAACCUUUGAAG........................................................................................................................................................... 19.175115
.................UGGAUUGUAACCGGACCUUUG.............................................................................................................................................................. 2.20164
```

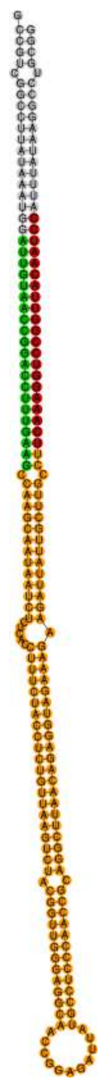

```
                    Star                                                        Mature
GAUACUCUAGUAUUGACCCCAAUACACAUGGGUUGAGGAGGAUUGGGUGUAAAUUAAACUAAUUUACAUCUCAAUCCACCUCAACUCAUGUGUAUUGGGGUCAAUACUAGAGCACC    385.7
....((((((((((((((((((((((((((((((((((.(((((((((((((((((...))))))))))).)))))).))))))))))))))))))))))))))))))))))....
.............................................................................ACCUCAACUCAUGUGUAUUGG..................    108.102979
....................AAUACACAUGGGUUGAGGAGG...........................................................................    131.093442
..............................................................................CCUCAACUCAUGUGUAUUGGG.................    116.326858
.....................AUACACAUGGGUUGAGGAGGA..........................................................................    4.211418
...................CAAUACACAUGGGUUGAGGAG............................................................................    26.015233
```

zma-MIR_N17b

Chromosome:	chr6
Start:	46131458
End:	46131573
Strand:	-
Strand bias:	1.0
Abundance bias:	1.0
MFE:	-83.10

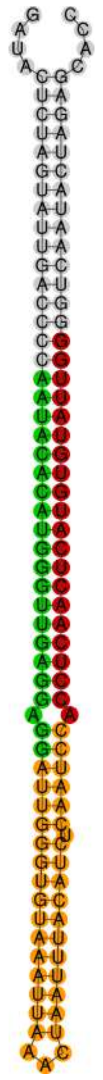

Mature Star

```
CUCUUAGGGCUCAUUUGGAAGCAAGGAUAAUGGAGGGGAUUGAGGGGGCUAGAAUCCCACUAUUCUAGCCCCCUCAAUCCCCUCCAUUAUCCUUGCUUUCAAACAAGCCCUUAGGU    161.4
.(((.((((((..((((((((((((((((((((((((((((((((((((((((((......))))))))))))))))))))))))))))))))))))))))))..)))))).))).
..................AAGCAAGGAUAAUGGAGGGGA.............................................................................    100.029161
...............................................................................CCCUCCAUUAUCCUUGCUUUC................    2.016043
...............UGGAAGCAAGGAUAAUGGAGG................................................................................    25.81819
...................AGCAAGGAUAAUGGAGGGGAU............................................................................    19.201384
............................................................................AUCCCCUCCAUUAUCCUUGCU...................    6.543003
```

zma-MIR_N32a

Chromosome: chr1
Start: 220123752
End: 220123867
Strand: -
Strand bias: 1.0
Abundance bias: 0.952
MFE: -94.50

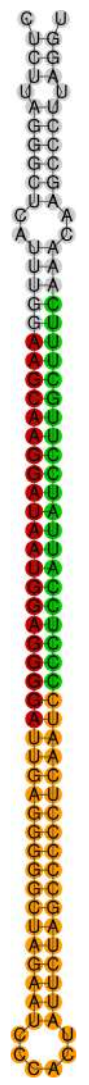

zma-MIR2275_N1

Chromosome: chr1
Start: 237845322
End: 237845446
Strand: +
Strand bias: 1.0
Abundance bias: 0.997
MFE: -47.70

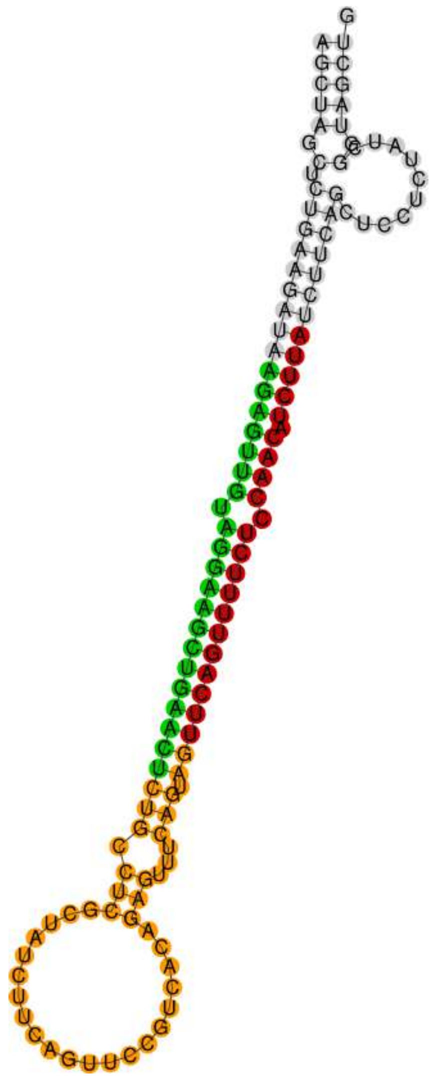

```
                 Star                                                        Mature
AGCUAGCUCUGAAGAUAAGAGUUGUAGGAAGCUGAACUCUGCCUCGCUAUCUUCAGUUCCGUCACAGAGUUUCAGUAGUUCAGUUUUCUCCAACAUCUUAUCUUCAGCUCCUCUAUCGGUAGCUG   1762.3
(((((.(.((((((((((((((((.((((((((((((((((.(((.....................)))...))).))))))))))))).)))).))))))))))))..........).))))).
..............................................................................UUCAGUUUUCUCCAACAUCUUA.........................   1645.097643
.................AGAGUUGUAGGAAGCUGAACU.......................................................................................   23.22133
................................................................................CAGUUUUCUCCAACAUCUUA.........................   0.17779
...............................................................................UCAGUUUUCUCCAACAUCUU..........................   1.35475
...............................................................................UCAGUUUUCUCCAACAUCUUA.........................   3.28201
..............................................................................UUCAGUUUUCUCCAACAUCUUAU........................   2.483008
..............................................................................UUCAGUUUUCUCCAACAUCU...........................   8.06324
..............................................................................UUCAGUUUUCUCCAACAUCUU..........................   31.22842
..............................................................................UUCAGUUUUCUCCAACAUC............................   6.77714
..............................................................................UUCGGUUUUCUCCAACAUCUUA.........................   0.35558
..............................................................................UUCAGUUUUCUCCAACAUCUUUUC.......................   0.57042
..............................................................................UUCAGAUUUCUCCAACAUCUUA.........................   0.203188
..............................................................................UUCAGUUUUCUCCAACAUCUUAA........................   1.063712
..............................................................................UUAAGUUUUCUCCAACAUCUUA.........................   0.152391
..............................................................................UUCAGUUUUCUCCAACAUCUUUU........................   4.25881
..............................................................................UUCAGUUUUCUACAACAUCUUA.........................   0.457174
..............................................................................UCCAGUUUUCUCCAACAUCUUA.........................   0.203188
..............................................................................UUCAGUGUUCUCCAACAUCUUA.........................   0.936719
..............................................................................UUCAGUUUUCUCCAAAAUCUUA.........................   0.967011
..............................................................................UUCAGUCUUCUCCAACAUCUUA.........................   0.203188
..............................................................................UUCAGUUUUCUCCAACAUAUUA.........................   0.757996
..............................................................................UUCAGUUUUCUCCAACGUCUUA.........................   0.253986
..............................................................................UUCAGUUUUCUCAAACAUCUUA.........................   0.17779
..............................................................................UUCAGUUUUCUCCAACAUCUUCU........................   0.152391
..............................................................................UUCAGGUUUCUCCAACAUCUUA.........................   5.75429
..............................................................................UUCAGUUUUUUCCAACAUCUUA.........................   0.17779
..............................................................................UUCAGUUUUCUCCAACAUCUGA.........................   0.304783
..............................................................................UUCAGUUCUCUCCAACAUCUUA.........................   0.203188
..............................................................................UUCAGUUUUCCCCAACAUCUUA.........................   0.457174
..............................................................................UUCAGUUUUCUCCAACAUUUUA.........................   0.17779
..............................................................................UUCAGCUUUCUCCAACAUCUUA.........................   1.246395
..............................................................................UUCAGUUUUCUCCAACAUCUUU.........................   12.2109
..............................................................................UUCAGUUUUCUCCAACUUCUUA.........................   0.228587
..............................................................................UGCAGUUUUCUCCAACAUCUUA.........................   0.152391
..............................................................................UUCAGUUUUCUCCAACAUCUCA.........................   0.152391
..............................................................................UUCAGUUUUCUCCAACCUCUUA.........................   0.900601
..............................................................................UUCAGUUUUCUCCAAUAUCUUA.........................   0.799007
..............................................................................UUCCGUUUUCUCCAACAUCUUA.........................   0.228587
..............................................................................UUCAGUUUUCUCCAACAUCCUA.........................   0.17779
..............................................................................UUCAGUUUUAUCCAACAUCUUA.........................   0.631003
....................GUUGUAGGAAGCUGAACUCU.....................................................................................   0.17779
.................AGAGUUGUAGGAAGCUGAAC........................................................................................   0.380978
.................AGAGUUGUAGGAAGCUGA..........................................................................................   0.17779
```

zma-MIR2275_N3

Chromosome: chr6
Start: 6798007
End: 6798133
Strand: +
Strand bias: 1.0
Abundance bias: 0.993
MFE: -50.40

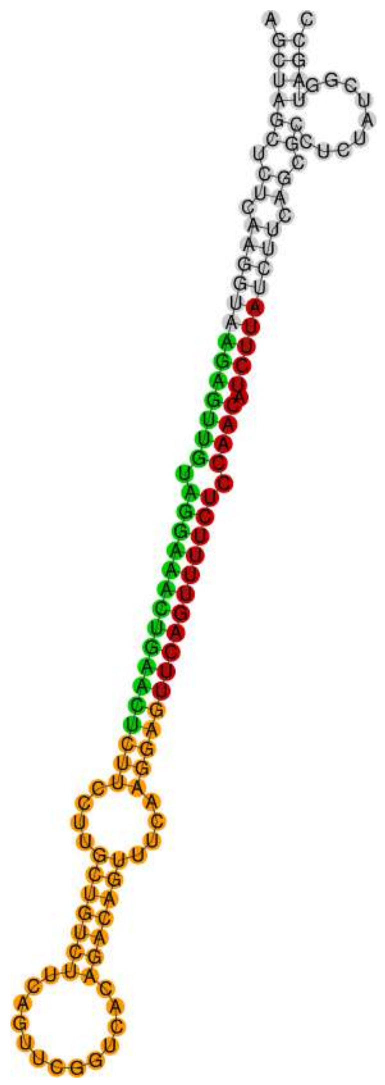

Star Mature

AGCUAGCUCUCAAGGUAAGAGUUGUAGGAAACUGAACUCUUCCUUGCUGUCUUCAGUUCGGUCACAGACAGUUUCAAGGAGUUCAGUUUUCUCCAACAUCUUAUCUUCAGCGCCUCUAUCGGUAGCC	2785.5
.((((((.((.(((((((((((((.((((((((((((((((....(((((((.............)))))))....)))))))))))))))).)))).))))))))).)).)).........)))).	
....UUCAGUUUUCUCCAACAUCUUA........................	1645.097643
.................AGAGUUGUAGGAAACUGAACU...	1005.608176
....CAGUUUUCUCCAACAUCUUA........................	0.17779
....UCAGUUUUCUCCAACAUCUU.........................	1.35475
....UCAGUUUUCUCCAACAUCUUA........................	3.28201
....UUCAGUUUUCUCCAACAUCUUAU.......................	2.483008
....UUCAGUUUUCUCCAACAUCU..........................	8.06324
....UUCAGUUUUCUCCAACAUCUU.........................	31.22842
....UUCAGUUUUCUCCAACAUC...........................	6.77714
....UUCGGUUUUCUCCAACAUCUUA........................	0.35558
....UUCAGUUUUCUCCAACAUCUUUUC......................	0.57042
....UUCAGAUUUCUCCAACAUCUUA........................	0.203188
....UUCAGUUUUCUCCAACAUCUUAA.......................	1.063712
....UUAAGUUUUCUCCAACAUCUUA........................	0.152391
....UUCAGUUUUCUCCAACAUCUUUU.......................	4.25881
....UUCAGUUUUCUACAACAUCUUA........................	0.457174
....UCCAGUUUUCUCCAACAUCUUA........................	0.203188
....UUCAGUGUUCUCCAACAUCUUA........................	0.936719
....UUCAGUUUUCUCCAAAAUCUUA........................	0.967011
....UUCAGUCUUCUCCAACAUCUUA........................	0.203188
....UUCAGUUUUCUCCAACAUAUUA........................	0.757996
....UUCAGUUUUCUCCAACGUCUUA........................	0.253986
....UUCAGUUUUCUCAAACAUCUUA........................	0.17779
....UUCAGUUUUCUCCAACAUCUUCU.......................	0.152391
....UUCAGGUUUCUCCAACAUCUUA........................	5.75429
....UUCAGUUUUUUCCAACAUCUUA........................	0.17779
....UUCAGUUUUCUCCAACAUCUGA........................	0.304783
....UUCAGUUCUCUCCAACAUCUUA........................	0.203188
....UUCAGUUUUCCCCAACAUCUUA........................	0.457174
....UUCAGUUUUCUCCAACAUUUUA........................	0.17779
....UUCAGCUUUCUCCAACAUCUUA........................	1.246395
....UUCAGUUUUCUCCAACAUCUUU........................	12.2109
....UUCAGUUUUCUCCAACUUCUUA........................	0.228587
....UGCAGUUUUCUCCAACAUCUUA........................	0.152391
....UUCAGUUUUCUCCAACAUCUCA........................	0.152391
....UUCAGUUUUCUCCAACCUCUUA........................	0.900601
....UUCAGUUUUCUCCAAUAUCUUA........................	0.799007
....UUCCGUUUUCUCCAACAUCUUA........................	0.228587
....UUCAGUUUUCUCCAACAUCCUA........................	0.17779
....UUCAGUUUUAUCCAACAUCUUA........................	0.631003
....................GUUGUAGGAAACUGAACUCU...	0.152391
...................AGUUGUAGGAAACUGAACU...	0.152391
..................GAGUUGUAGGAAACUGAACU...	1.796307
.................AGAGUUGUAGGAAACUGAACUC..	2.76844
.................AGAGUUGUAGGAAACUGAA...	0.457174
.................AGAGUUGUAGGAAACUGAAC..	7.93298
.................AGAGUUGUAGGAAACUGA..	0.457174
.................AGAGUUGUAGGAAACUGAACUCU...	0.304783
.................AGAGUUGUAGGAAACUGAACUU..	3.4542
.................AGAGUUGUAGGAAACUGAACUA..	0.431775
.................AGAGUUGUAGGAAACUGAAUU...	0.584167
.................AGAGUUGUAGGAAACUAAACU...	0.457174
.................AGAGUUGUAGGGAACUGAACU...	0.406377
.................AGAGUUAUAGGAAACUGAACU...	0.152391
.................AGAGUUGUAGGAAACUGGACU...	0.330181
.................AGGGUUGUAGGAAACUGAACU...	0.17779
.................AGAGCUGUAGGAAACUGAACU...	0.304783
.................AAAGUUGUAGGAAACUGAACU...	0.152391
.................AGAGUUGUAGGAAAAUGAACU...	0.53337
.................AGAGUUGUAGGACACUGAACU...	0.152391
.................AGAGUUGGAGGAAACUGAACU...	1.04134
.................AGAGUUGUAGGAAACUGAAU..	0.203188
.................AGAGUUGUAGGAAACUGAAGU...	0.152391
.................AGAGAUGUAGGAAACUGAACU...	0.228587
.................AGAGUUGUAGGAAACUGAGCU...	0.253986
.................AGAGUGGUAGGAAACUGAACU...	0.228587
.................AGAGUUGUAGGAAAUUGAACU...	0.330181
.................AGAAUUGUAGGAAACUGAACU...	0.17779
.................AGAGUUGUAGGAAACUGCACU...	0.152391
.................GGAGUUGUAGGAAACUGAACU...	0.203188
.................AGAGUUGUAGGAAACUGAACG...	0.330181
.................AGAGUUGAAGGAAACUGAACU...	0.17779
.................AGAGUUGCAGGAAACUGAACU...	0.609565
.................AGAGUUGUAGGAAACUGAACC...	0.203188
.................AGAGUUGUAGGAAACUGAACA...	0.253986
.................AGAGUUGUAGGAAACCGAACU...	0.253986
.................AGAGUUGUGGGAAACUGAACU...	0.152391
.................AGAGUCGUAGGAAACUGAACU...	0.17779
.................AGAGUUGUAGGAAACUGUACU...	0.152391
.................AGAGUUUUAGGAAACUGAACU...	0.152391
.................AGAGUUGUAAGAAACUGAACU...	0.228587
.................AGAGUUGUUGGAAACUGAACU...	0.152391
.................AGAGUUGUAGGAAACUCAACU...	0.17779
.................ACAGUUGUAGGAAACUGAACU...	0.228587
.................AGAGUUGUAGGAAACUUAACU...	0.152391
.................AGAGGUGUAGGAAACUGAACU...	0.609565
.................AGAGUUGUAGGAAACUGAAAU...	0.507971

zma-MIR_N39a

```
Chromosome:    chr4
Start:         109027559
End:           109027800
Strand:        +
Strand bias:   1.0
Abundance bias:0.896
MFE:           -141.00
```

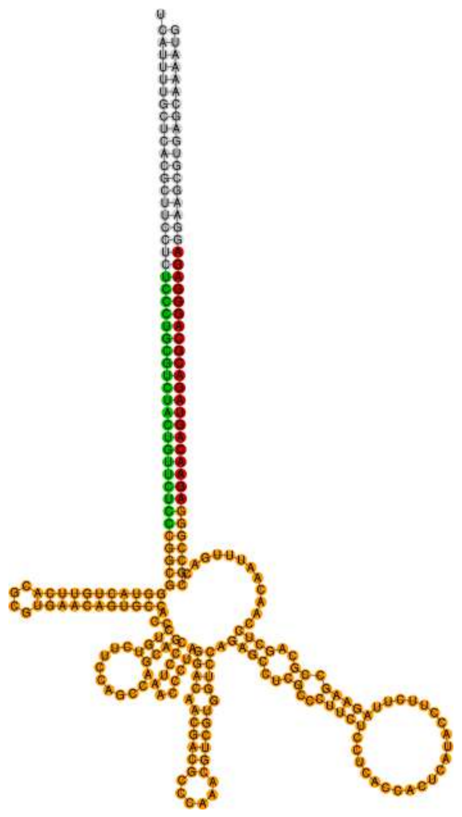

```
                    Star                                                                                                                                                                                                             Mature
UCAUUUUGCUCACGCUUCCUCUCCCUGCGUCUACUGUUCUCCCGGCGGGUACUGUUCACGCGUGAACAGUGCCACCCUGUCUUCCAGCCAGCAGCCCUAACCCUAGGACAACGACGCCCAAACGUCGUGGUCCAGAGCCUCGCCCUUCUCCUCACCACUCAUACCUUCUUAGAAGCCGCAGCUCCAACAAUUUGACCGCCGGGAGAACAGUAGACGCAGGGAGAGGAAGCGUGAGCAAAAUG   181.0
.(((((((((((((((((((((((((((((((((((((((((((((((((((((((((....)))))))))))...((((..........))))...........((((.((((((......)))))).)))).((((..((..(((((......................))))).))..))))...........))))))))))))))))))))))))))))))))))))))))))))))
...........................................................................................................................................................................................................AGAACAGUAGACGCAGGGAGA..................   91.941904
.....................UCCCUGCGUCUACUGUUCUCC........................................................................................................................................................................................................   27.178808
............................................................................................................................................................................................................GAACAGUAGACGCAGGGAGAG.................   3.28228
...........................................................................................................................................................................................................AGAACAGUAGACGCAGGGAGAGGA...............   6.590149
...........................................................................................................................................................................................................AGAACAGUAGACGCAGGGAGAG.................   1.44278
...........................................................................................................................................................................................................AGAACAGUAGACGCAGGGAGAGG................   0.995617
..........................................................................................................................................................................................................GAGAACAGUAGACGCAGGGAGAGG................   1.585359
..........................................................................................................................................................................................................GAGAACAGUAGACGCAGGGAG...................   25.820101
........................................................................................................................................................................................................GGGAGAACAGUAGACGCAGGGA...................   1.902077
........................................................................................................................................................................................................GGGAGAACAGUAGACGCAGGGAGA.................   1.267371
......................CCCUGCGUCUACUGUUCUCCC.......................................................................................................................................................................................................   0.152391
```

zma-MIR_N39b

```
Chromosome:    chr4
Start:         109027561
End:           109027800
Strand:        -
Strand bias:   1.0
Abundance bias:0.896
MFE:           -149.50
```

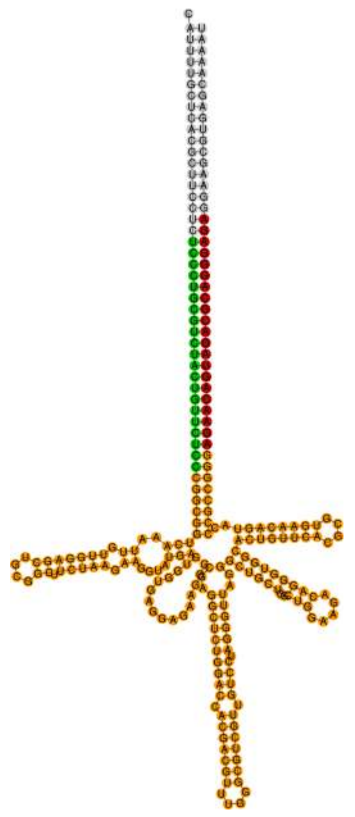

```
                    Star                                                                                                                                                                                      Mature
CAUUUUGCUCACGCUUCCUCUCCCUGCGUCUACUGUUCUCCCGGCGGUCAAAUUGUUGGAGCUGCGGCUUCUAAGAAGGUAUGAGUGGUGAGGAGAAGGGCGAGGCUCUGGACCACGACGUUUGGGCGUCGUUGUCCUAGGGUUAGGGCUGCUGGCUGGAAGACAGGGUGGCACUGUUCACGCGUGAACAGUACCCGCCGGGAGAACAGUAGACGCAGGGAGAGGAAGCGUGAGCAAAAU    181.0
.(((((((((((((((((((((((((((((((((((((((((((((((((..((.(((((((....)).))))).))....)))................(..((((((((((.(((((((....))))))).)))).))))))..)((((((..(((.....)))))))))((((((((....))))))))..))))))))))))))))))))))))))))))))))))))))))))))
..........................................................................................................................................................................................................AGAACAGUAGACGCAGGGAGA.................    91.941904
....................UCCCUGCGUCUACUGUUCUCC.......................................................................................................................................................................................................    27.178808
...........................................................................................................................................................................................................GAACAGUAGACGCAGGGAGAG................    3.28228
..........................................................................................................................................................................................................AGAACAGUAGACGCAGGGAGAGGA..............    6.590149
..........................................................................................................................................................................................................AGAACAGUAGACGCAGGGAGAG................    1.44278
..........................................................................................................................................................................................................AGAACAGUAGACGCAGGGAGAGG...............    0.995617
.........................................................................................................................................................................................................GAGAACAGUAGACGCAGGGAGAGG...............    1.585359
.........................................................................................................................................................................................................GAGAACAGUAGACGCAGGGAG..................    25.820101
.......................................................................................................................................................................................................GGGAGAACAGUAGACGCAGGGA...................    1.902077
.......................................................................................................................................................................................................GGGAGAACAGUAGACGCAGGGAGA.................    1.267371
.....................CCCUGCGUCUACUGUUCUCCC......................................................................................................................................................................................................    0.152391
```

```
                  Mature                                                            Star
GUUUGGGACCAAGUGAAAUGAAGAGAAUUGAGGGGGCUAUAAUCCCUUGUUAUUCAAAAUUGAAUAGCAAGGGAUUAUAGCCCCCUCAAUUCUCUUUGUUCCACCUACUCCCAAACA    461.4
((((((((....(((.((((((((((((((((((((((((((((((((((((((((....)))))))))))))))))))))))))))))))))))))))).)))....)))))))).
..................UGAAGAGAAUUGAGGGGGCUA..............................................................................    367.603094
...............................................................................GCCCCCUCAAUUCUCUUUGUU.................    0.737033
...............AAAUGAAGAGAAUUGAGGGGGC................................................................................    1.795135
...............AAAUGAAGAGAAUUAAGGGGGCUA..............................................................................    1.927074
...............AAAUGAAGAGAAUUAAGGGGGC................................................................................    0.319396
.................AUGAAGAGAAUUGAGGGGGCU...............................................................................    18.756317
.................AUGAAGAGAAUUAAGGGGGCUAUA............................................................................    0.365563
..................UGAAGAGAAUUGAGGGGGCU...............................................................................    14.509826
..................UGAAGAGAAUUGAGGGGGCUU..............................................................................    0.182396
............................................................................AUAGCCCCCUCAAUUCUCUUUGUU.................    4.070754
```

zma-MIR_N1a

```
Chromosome:    chr4
Start:         235905513
End:           235905629
Strand:        -
Strand bias:   1.0
Abundance bias:0.889
MFE:           -83.90
```

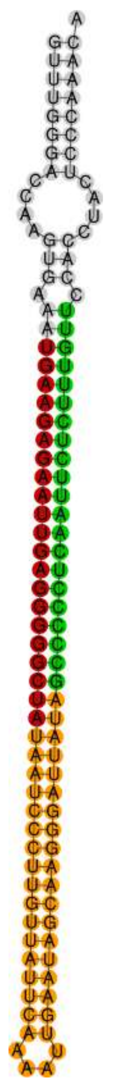

zma-MIR_N1d

Chromosome: chr4
Start: 242220925
End: 242221041
Strand: -
Strand bias: 1.0
Abundance bias: 0.885
MFE: -79.00

```
                  Mature                                                             Star
GUUUGGGACCAAGUGAAAUGAAGAGAAUUGAGGGGGCUAUAAUCCCUUGUUAUUCAAAAUUGAAUAGUAAGGGAUUAUAGCCCCCUCAAUUCUCUUUGUUCCACCUACUCCCAAGCA    463.7
((((((((....(((.((((((((((((((((((((((((((((((((((((((((....)))))))))))))))))))))))))))))))))))))))).)))....)))))))).
..................UGAAGAGAAUUGAGGGGGCUA..............................................................................    367.603094
...............................................................................GCCCCCUCAAUUCUCUUUGUU.................    0.737033
...............AAAUGAAGAGAAUUGAGGGGGC................................................................................    1.795135
...............AAAUGAAGAGAAUUAAGGGGGCUA..............................................................................    1.927074
...............AAAUGAAGAGAAUUAAGGGGGC................................................................................    0.319396
.................AUGAAGAGAAUUGAGGGGGCU...............................................................................    18.756317
.................AUGAAGAGAAUUAAGGGGGCUAUA............................................................................    0.365563
..................UGAAGAGAAUUGAGGGGGCU...............................................................................    14.509826
..................UGAAGAGAAUUGAGGGGGCUU..............................................................................    0.182396
............................................................................AUAGCCCCCUCAAUUCUCUUUGUU.................    4.070754
```

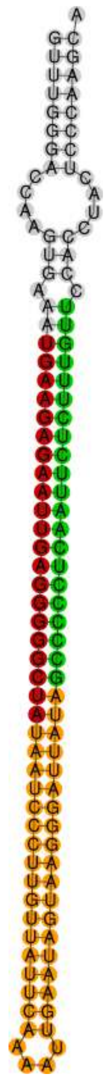

zma-MIR_N1e

Chromosome: chr6
Start: 128049160
End: 128049276
Strand: -
Strand bias: 1.0
Abundance bias: 0.885
MFE: -79.00

```
                  Mature                                                     Star
GUUUGGGACCAAGUGAAAUGAAGAGAAUUGAGGGGGCUAUAAUCCCUUGUUAUUCAAAAUUGAAUAGUAAGGGAUUAUAGCCCCCUCAAUUCUCUUUGUUCCACCUACUCCCAAACA    463.7
(((((((....(((.(((((((((((((((((((((((((((((((((((((((((....))))))))))))))))))))))))))))))))))))))))).)))....))))))).
..................UGAAGAGAAUUGAGGGGGCUA..............................................................................    367.603094
...............................................................................GCCCCCUCAAUUCUCUUUGUU.................    0.737033
...............AAAUGAAGAGAAUUGAGGGGGC................................................................................    1.795135
...............AAAUGAAGAGAAUUAAGGGGGCUA..............................................................................    1.927074
...............AAAUGAAGAGAAUUAAGGGGGC................................................................................    0.319396
.................AUGAAGAGAAUUGAGGGGGCU...............................................................................    18.756317
.................AUGAAGAGAAUUAAGGGGGCUAUA............................................................................    0.365563
..................UGAAGAGAAUUGAGGGGGCU...............................................................................    14.509826
..................UGAAGAGAAUUGAGGGGGCUU..............................................................................    0.182396
............................................................................AUAGCCCCCUCAAUUCUCUUUGUU.................    4.070754
```

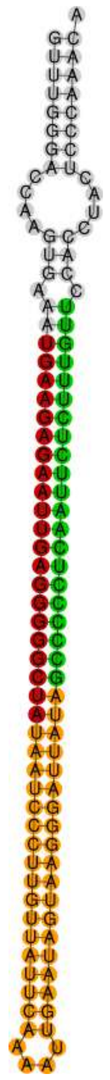

	Mature	Star	

```
ACAUCAAAUCUUGUCCAUCGGAUCCACCAUCUAUGGCUAAGUGCAAUCUCGCCACCUCUCAGACUAUUUUCUAAAGAACCCCUAUGUUUUCACAUAAUAUGGCGUGCUAUCCCCAGGUGGGAUUGCACUCAGCCGUAGAUGAUGGAUUUGAUGGACAAGAUUUGAUGUU    113.7
((((((((((((((((((((((((((.(((((((((((.((((((((((((((...(((.(((......)))..))).....(((((....)))))...(((.(......).))))))))))))))))).))))))))))).)))))))))))))))))))))))))).
.................UCGGAUCCACCAUCUAUGGCU...................................................................................................................................    89.268419
....................................................................................................................................CCGUAGAUGAUGGAUUUGAUG................    7.44596
...................................................................................................................................GCCGUAGAUGAUGGAUUUGAU.................    6.391451
....................................................................................................................................CCGUAGAUGAUGGAUUUGAUGGAC.............    2.507644
```

zma-MIR_N125a

Chromosome: chr5
Start: 10259222
End: 10259390
Strand: +
Strand bias: 1.0
Abundance bias: 0.928
MFE: -89.60

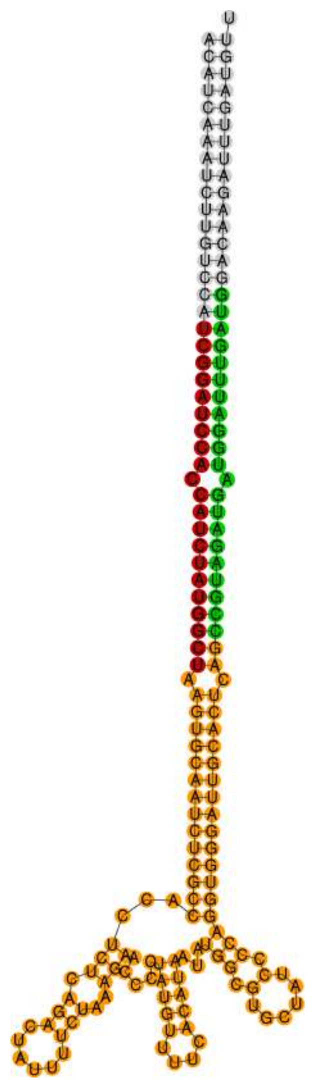

zma-MIR_N34b

```
Chromosome:    chr5
Start:         11380490
End:           11380691
Strand:        +
Strand bias:   1.0
Abundance bias:0.998
MFE:           -83.30
```

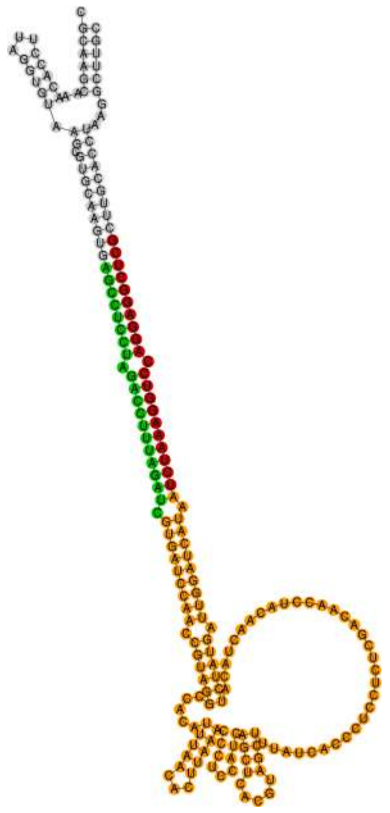

```
                                   Star                                                                                                                                    Mature
CGCAAGCAAACACCUUAGGUGUAAGCGUGCAAGUGAGCCUCCUAGACCUUUAGAUCGUGAUCCAACCGUAGGCCACAUUUAACACUUAAACCCUCCAUCAGCUCACGUAGCUUUAUCACCCUCCUCUCGACAACCUACAACUACAUCUAUGAUUGGAUCAUAAUCUAAAGGUCCAGGAGGCUCGCUUGCACCUAAGGCUUGC   276.
.((((((..(((((...))))).((.((((((((((((((((.((((((((((.(((((((((.((((((.....(((((...)))))........((((.....))))....................................)))))).))))))))).))))))))))).))))))))))))))))))...))))))
...................................................................................................................................................................UCUAAAGGUCCAGGAGGCUCG..................   193.167864
...................................AGCCUCCUAGACCUUUAGAUC..................................................................................................................................................   22.04335
...................................................................................................................................................................UCUAAAGGUCCAGGAGGC.....................   1.64419
...................................................................................................................................................................UCUAAAGGUCCAGGAGGCU....................   10.233154
...................................................................................................................................................................UCUAAAGGUCCAGGAGGCUC...................   1.511467
..................................................................................................................................................................AUCUAAAGGUCCAGGAGGCUC...................   44.332901
....................................GCCUCCUAGACCUUUAGAUCG.................................................................................................................................................   1.54137
...............................GUGAGCCUCCUAGACCUUUAGAUC..................................................................................................................................................   1.89491
```

zma-MIR_N26b

Chromosome: chr6
Start: 145789872
End: 145789987
Strand: +
Strand bias: 1.0
Abundance bias: 0.794
MFE: -85.00

	Mature / Star	
	GGUUGAUUUGGUGAGAAGUGAUCACGGGAGAUUGGAGAAGAUUGAGGGGAAAAUUAGUUUAUUUUCUCCUCAAUUCUCUCCAAUCUUCCGUGAUCCACUUGUCACCAAAUCAGCCC	625.2
	((((((((((((((.(((((((((((((((((((((((.(((((((((((((((......))))))))))))))).)))))))))))))))))).))))).)))))))))))))).	
	.................GUGAUCACGGGAGAUUGGAGA..	283.8489
	....UCCAAUCUUCCGUGAUCCACUU................	80.390689
	..............GAAGUGAUCACGGGAGAUUGGA..	0.42559
	..............GAAGUGAUCACGGGAGAUUGGAGA..	2.408382
	...............AAGUGAUCACGGGAGAUUGGA..	0.243195
	................AGUGAUCACGGGAGAUUGGAGA..	0.212795
	................AGUGAUCACGGGAGAUUGGA..	3.593512
	................AGUGAUCACGGGAGAUUGGAG...	94.820001
	.................GUGAUCACGGGAGAUUGG...	10.109
	..................UGAUCACGGGAGAUUGGAGAAG..	5.3554
	....UCCAAUCUUCCGUGAUCCACU.................	15.091301

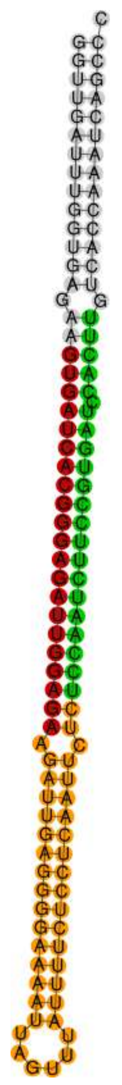

zma-MIR_N29a

Chromosome: chr6
Start: 92810434
End: 92810583
Strand: -
Strand bias: 1.0
Abundance bias: 0.932
MFE: -75.40

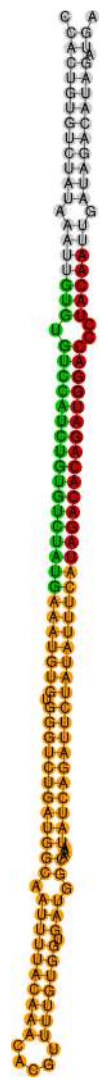

```
                  Star                                                                                             Mature
CCACUGUGUCUAUAAAUUGUGUGUCCAUCUGUGUCUAUGAAAUGUGUGGGUCUGAUGGCAAUUUUACAAACACGUUUUGUGGUGAUGGCAAAUAUCAGAUUCUAUAUUUCAUAGACACAGAUGGACCCUACAAUUGAUAGACAUAGAUGA    1764.6
.((((((((((((.(((((((.((((((((((((((((((((((((.((((((((((((.((((((((((.....))))))).))).))...))))))))))))))))))))))))))))))))))..))))))).)))))))))).)).
...............................................................................................................UAGACACAGAUGGACCCUACAA.................    678.692831
..................GUGUGUCCAUCUGUGUCUAUG...............................................................................................................    80.249838
................................................................................................................AGACACAGAUGGACCCUACAAU................    34.070274
................................................................................................................AGACACAGAUGGACCCUACAA.................    2.31455
...............................................................................................................UAGACACAGAUGGACCCUA....................    3.432574
...............................................................................................................UAGACACAGAUGGACCCUAC...................    51.646268
...............................................................................................................UAGACACAGAUGGACCCUACAAU................    28.63654
...............................................................................................................UAGACACAGAUGGACCCUACA..................    115.641432
...............................................................................................................UAGACACAGAUGGACCCUACAAA................    1.837555
...............................................................................................................UAGACACAGAUGGACCCUACAU.................    7.132832
...............................................................................................................UAGACACAGAUGGACCCUACAUU................    3.89362
...............................................................................................................UAGACACAGAUGGACCCUACAUUU...............    14.301431
...............................................................................................................UAGACACAGAUGGACCCUACU..................    11.297538
..............................................................................................................AUAGACACAGAUGGACCCUAC...................    14.451719
..............................................................................................................AUAGACACAGAUGGACCCUACA..................    134.780448
..............................................................................................................AUAGACACAGAUGGACCCUA....................    0.243195
.................UGUGUGUCCAUCUGUGUCUAU................................................................................................................    100.961905
................UUGUGUGUCCAUCUGUGUCUA.................................................................................................................    154.757874
................UUGUGUGUCCAUCUGUGUCU..................................................................................................................    16.84003
................UUGUGUGUCCAUAUGUGUCUA.................................................................................................................    188.858273
```

zma-MIR_N29b

Chromosome: chr6
Start: 92898528
End: 92898678
Strand: -
Strand bias: 1.0
Abundance bias: 0.932
MFE: -76.00

```
                   Star                                                                     Mature
ACCACUGUGUCUAUAAAUUGUGUGUCCAUCUGUGUCUAUGAAAUGUGUGGGUCUGAUGGCAAUUUUACAAACGUGUUUCAUGGUGAUGGCAAAUAUCAGAUUCUAUAUUUCAUAGACACAGAUGGACCCUACAAUUGAUAGACAUAGAUGG    1764.6
.(((((((((((((.(((((((.((((((((((((((((((((((((.((((((((((((....((((...((((...))))))))..))...))))))))))))))))))))))))))))))))))..))))))).)))))))))).)))
................................................................................................................UAGACACAGAUGGACCCUACAA.................    678.692831
...................GUGUGUCCAUCUGUGUCUAUG...............................................................................................................    80.249838
.................................................................................................................AGACACAGAUGGACCCUACAAU.................    34.070274
.................................................................................................................AGACACAGAUGGACCCUACAA..................    2.31455
................................................................................................................UAGACACAGAUGGACCCUA....................    3.432574
................................................................................................................UAGACACAGAUGGACCCUAC...................    51.646268
................................................................................................................UAGACACAGAUGGACCCUACAAU................    28.63654
................................................................................................................UAGACACAGAUGGACCCUACA..................    115.641432
................................................................................................................UAGACACAGAUGGACCCUACAAA................    1.837555
................................................................................................................UAGACACAGAUGGACCCUACAU.................    7.132832
................................................................................................................UAGACACAGAUGGACCCUACAUU................    3.89362
................................................................................................................UAGACACAGAUGGACCCUACAUUU...............    14.301431
................................................................................................................UAGACACAGAUGGACCCUACU..................    11.297538
...............................................................................................................AUAGACACAGAUGGACCCUAC...................    14.451719
...............................................................................................................AUAGACACAGAUGGACCCUACA..................    134.780448
...............................................................................................................AUAGACACAGAUGGACCCUA....................    0.243195
..................UGUGUGUCCAUCUGUGUCUAU................................................................................................................    100.961905
.................UUGUGUGUCCAUCUGUGUCUA.................................................................................................................    154.757874
.................UUGUGUGUCCAUCUGUGUCU..................................................................................................................    16.84003
.................UUGUGUGUCCAUAUGUGUCUA.................................................................................................................    188.858273
```

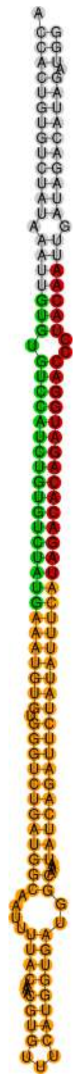

Mature Star

```
UUAAGGACACAUAAAGGACUAGUUCGGAAGCAAGAGAAUUAGAGGAGAGAUGGCUAAAAUCCCCUUCCUAAUUAAAUAGGAAGGGAAUUUGAGCACCCUCAAUCCCCUCUAAUUCCCUUGCUUUCAAACUAGCCCAAAGAGUGUCACUGCA     187.4
...(((((((.....((.((((((.(((((((((.((((((((((.(((...(((.((((.(((((((((......))))))))).)))).)))...))).....)))))))))).))))))))).)))))).)).....))))).))...
.................ACUAGUUCGGAAGCAAGAGAA.................................................................................................................     171.550281
..................................................................................................................CCCUUGCUUUCAAACUAGCCC................     0.645157
................GACUAGUUCGGAAGCAAGAGA..................................................................................................................     5.294801
.................ACUAGUUCGGAAGCAAGAGAU.................................................................................................................     1.43492
```

zma-MIR_N200a

Chromosome: chr10
Start: 2751854
End: 2752004
Strand: +
Strand bias: 1.0
Abundance bias: 0.954
MFE: -67.10

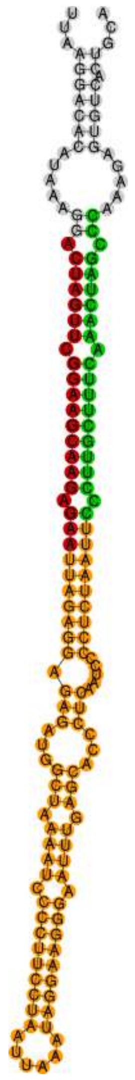

```
                     Mature                                                      Star
AGUUUGGGAGCCACAAAACCGGAUGAGUUGAAGGGACUACAAUCUCCUUCUUAUUUAAAUUUAAAUAAGAAGGGAAUUCUAGUCCCUCCAAUCUCCUUUGGUUUUGUGACUCUCAAACUA    126.4
((((((((((.((((((((((((.((((((.((((((((.(((.(((((((((((((....))))))))))))).))).)))))))).))).))).))))))))))))).)))))))))).
.................ACCGGAUGAGUUGAAGGGACU..................................................................................    88.14631
..................................................................................UCCCUCCAAUCUCCUUUGGUUU................    0.304783
..............AAAACCGGAUGAGUUGAAGGGACU..................................................................................    5.952874
...............AAACCGGAUGAGUUGAAGGGACU..................................................................................    0.457174
................AACCGGAUGAGUUGAAGGGACUAC................................................................................    0.702768
.................ACCGGAUGAGUUGAAGGGAC...................................................................................    1.16833
.................ACCGGAUGAGUUGAAGGGACUA.................................................................................    1.14293
.................ACCGGAUGAGUUGAAGGGAAU..................................................................................    0.152391
..................CCGGAUGAGUUGAAGGGACUA.................................................................................    0.228587
..................CCGGAUGAGUUGAAGGGACU..................................................................................    2.56525
...................CGGAUGAGUUGAAGGGACUA.................................................................................    0.203188
...................CGGAUGAGUUGAAGGGACU..................................................................................    0.253986
....................GGAUGAGUUGAAGGGACUA.................................................................................    0.203188
....................GGAUGAGUUGAAGGGACU..................................................................................    0.253986
```

zma-MIR_N205a

```
Chromosome:    chr10
Start:         53775196
End:           53775315
Strand:        +
Strand bias:   1.0
Abundance bias:0.805
MFE:           -65.80
```

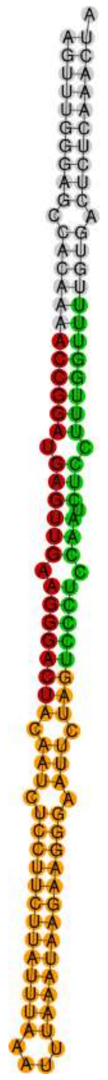

zma-MIR_N208a

```
Chromosome:    chr10
Start:         106806016
End:           106806235
Strand:        -
Strand bias:   1.0
Abundance bias:0.978
MFE:           -106.40
```

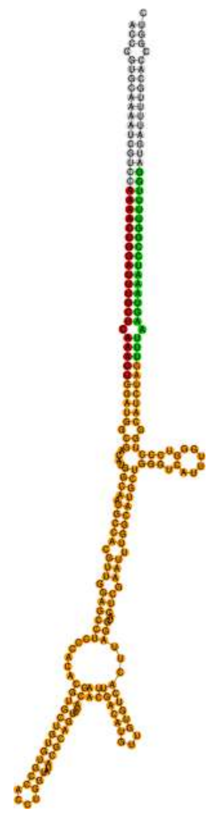

```
                  Mature                                                                                                                                                               Star
ACCCGUGCAAAAUCGUCCAAAACCGGAUUUGCUCAAACUGGAUGGCGACAUGGCAAUGCCACGUUGGAGCCUCCCACACGUGCUGUGUGCCACCUGGUAUACGCAGUCUACGAACUUGACAUGUUGUGUCACUUAGGUACUCGAAUUUGGCAUGCUGGGUCAUUUGGGUCCCUGGCAUCUAGUUUAAGUAAAUCCGGUUUUGGAUGAUUUUGCACCGGUC    193.0
(((.((((((((((((((((((((((((((((.((((((((((.((....((((.(((((.(((.((((((......((((((((((((....)))..)))))))..)))....((((((...))))))...)))..))).))).)))))))))(((.(......).))))).))))))))))).))))))))))))))))))))))))))))).))).
..................AAAACCGGAUUUGCUCAAACU.....................................................................................................................................................................................    80.817304
...................................................................................................................................................................UUUAAGUAAAUCCGGUUUUGG....................................    19.334009
...............UCCAAAACCGGAUUUGCUCAAAC......................................................................................................................................................................................    0.203188
...............UCCAAAACCGGAUUUGCUCAAACU.....................................................................................................................................................................................    1.211211
..................AAAACCGGAUUUGCUCAAACUGGA.................................................................................................................................................................................    0.304783
```

zma-MIR_N209a

```
Chromosome:    chr10
Start:         135044926
End:           135045148
Strand:        -
Strand bias:   1.0
Abundance bias:0.927
MFE:           -135.70
```

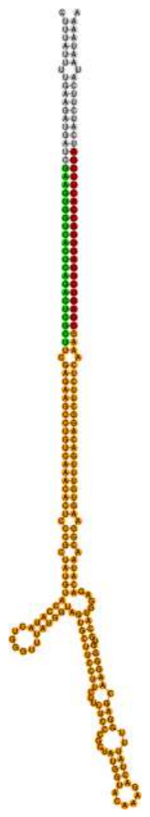

```
                    Star                                                                                                                                                                            Mature
CUUUAUUUUGAAGAUGAUCGAAGUGGUCACUCAGACUCGCUUCGAGAAGCCUGUCAAACACUCCCGCUAUGUACCAUACUGGGUUUAUGGUAGUGCUUCCUUCUUCUCCCGCUAUGGUACAAAGACUAUUUGGAGCAAGGGGUGCAUGGAGACAUAACGGAAGUGUUUGACAGGCUUCUCAAAGCGAGUCUGAGUGACCACUUCGAUCAUCUUCAUAAUAAAA   336.8
.(((((.((((((((((((((((((((((((((((((((((.(((((((((((((((((((.(((.((((((((((((.......)))))).((((((((((...((((....(((((......)))))..)))).)))))).))))....))))).))).))))))))))))))))))).))))))))))))))))))))))))))))))))))).)))))).
........................................................................................................................................................................................CGAGUCUGAGUGACCACUUCGA.................   115.24433
...................GAAGUGGUCACUCAGACUCGCU......................................................................................................................................................................................   3.961714
........................................................................................................................................................................................CGAGUCUGAGUGACCACUUCG..................   1.95085
........................................................................................................................................................................................CGAGUCUGAGUGACCACUUCGAUC...............   3.63037
.......................................................................................................................................................................................GCGAGUCUGAGUGACCACUUCG..................   10.19699
......................................................................................................................................................................................AGCGAGUCUGAGUGACCACUUCGA.................   44.443738
......................................................................................................................................................................................AGCGAGUCUGAGUGACCACUU....................   14.35553
....................AAGUGGUCACUCAGACUCGCU......................................................................................................................................................................................   17.208187
...................GAAGUGGUCACUCAGACUCGC.......................................................................................................................................................................................   5.88628
..................CGAAGUGGUCACUCAGACUCGC.......................................................................................................................................................................................   72.771653
.................UCGAAGUGGUCACUCAGACUCG........................................................................................................................................................................................   16.895616
.................UCGAAGUGGUCACUCAGACUCGCU......................................................................................................................................................................................   5.75313
```

Star   Mature

```
GGCCGCGGGGCCCAUGCAUUACCGGGUUGGGUCUCCUCUGGCAGUAACUUGCCUGGUCGUCGAUGACCUAGCUAGCGUUGCGUGCAGAGACACUGCCAAAGGAGAUUUGCCCAGCAAUGCAGUCUUGGGUG   157.3
.(((.((((((...((((((.(.((((..((((((((.(((((((..(((((..((((......))))..))..(((.....))).)))..))))))).)))))))).)))).).)))))))))))))))).
.............................................................................................UGCCAAAGGAGAUUUGCCCAG.................   55.718399
.......................GGGUUGGGUCUCCUCUGGCAGU......................................................................................   72.958956
.............................................................................................UGCCAAAGGAGAGUUGCCCA..................   10.730025
.............................................................................................UGCCAAAGGAGAUUUGCCCUG.................   15.91686
.............................................................................................UGCCAAAGGAGAUUUGCCCGG.................   2.066517
```

zma-MIR399_N3

Chromosome:	chr6
Start:	163865477
End:	163865607
Strand:	-
Strand bias:	1.0
Abundance bias:	1.0
MFE:	-55.10

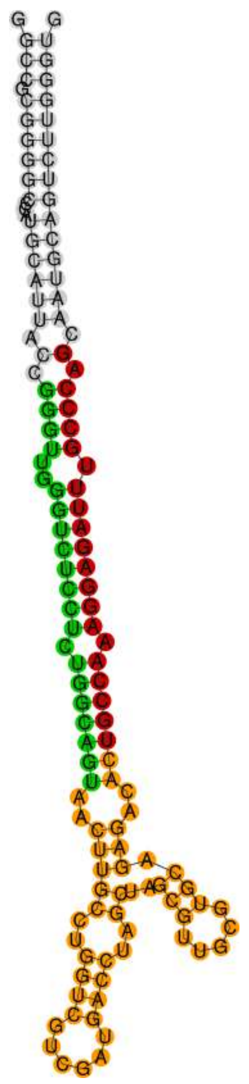

zma-MIR_N157a

Chromosome: chr6
Start: 171893487
End: 171893605
Strand: -
Strand bias: 1.0
Abundance bias: 0.928
MFE: -58.40

	Mature / Star	
	UCAAGUGAUCAAGUGCAUCCAAUCAAAAUCCAACGGUGAUUGCUAUUUUAGGUGGAAGAGGUUAAAUGUAAAUUAGCGUUCACCAUUGGAUUUUGAUUGAAUGGACCAGAUCGCUUGAU	394.2
	(((((((((..((.(((.((((((((((((((.(((((.(((((.((((.((..((....))..)).)))).))))).))))).)))))))))))))).))).))..))))))))).	
	..................CCAAUCAAAAUCCAACGGUGA..	301.065171
	....ACCAUUGGAUUUUGAUUGAAU.................	35.786144
	.................UCCAAUCAAAAUCCAACGGUG...	1.852487
	....CACCAUUGGAUUUUGAUUGAA..................	0.956615
	....ACCAUUGGAUUUUGAUUGAAUU................	1.893351
	....CCAUUGGAUUUUGAUUGAAUG................	0.318481
	....AUUGGAUUUUGAUUGAAUGGA..............	24.192722

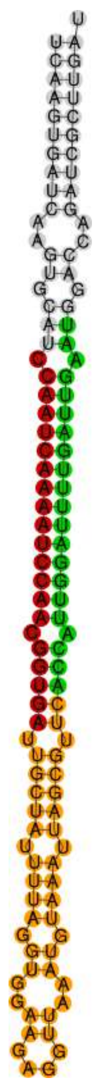

zma-MIR_N158a

```
Chromosome:    chr7
Start:         22702041
End:           22702224
Strand:        -
Strand bias:   1.0
Abundance bias:0.785
MFE:           -122.60
```

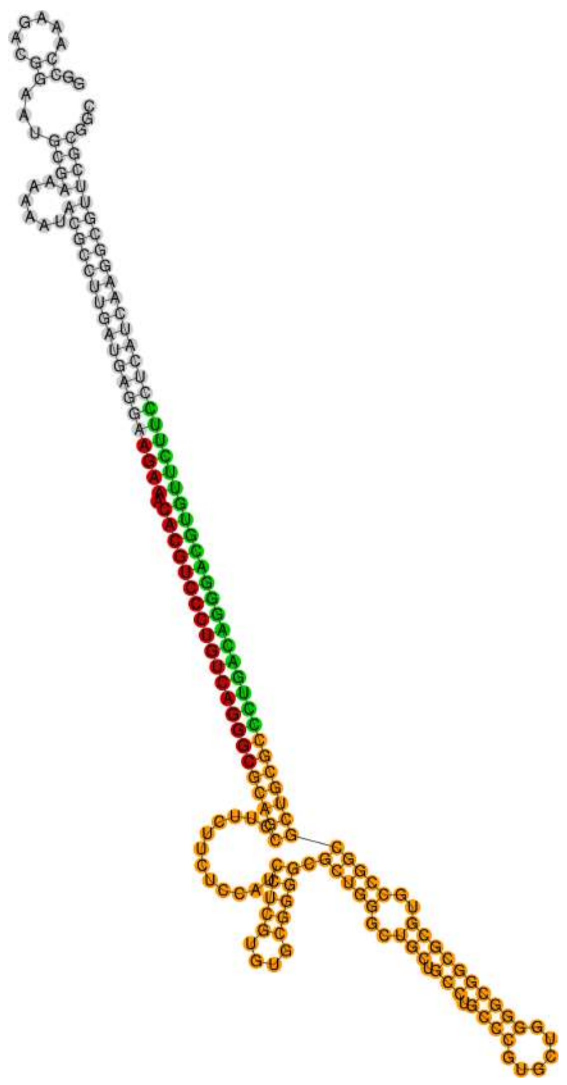

```
                                              Mature                                                                                           Star
GGCCAAAGACGGAAUGCGAAAAAAUACGCCUUGAUGAGGAAGAAACACGUCCCUGUCAGGGCGCAGCCUUCUUCUCCAUCCUCGUGUGCGGGGCGCUGGGCUGCUGCCUGCCCGUGCUGGGGCGGCGCGUGCCGGCGCUGCGCCCUGACAGGGACGUGUUCUUCCUCAUCAAGGCGUUCGCGGC    8640.9
..((......))...((((......(((((((((((((((((((.((((((((((((((((((((((............(((((....))))).(((((..(((.(((.((((......))))))))))..))))))))))))))))))))))))))))))))))))))))))))))))))...
........................................AGAAACACGUCCCUGUCAGGGC..........................................................................................................................    2309.446661
...............................................................................................................................................CCUGACAGGGACGUGUUCUUC....................    44.763658
.....................................GGAAGAAACACGUCCCUGUCAGGGC..........................................................................................................................    11.58167
.......................................AAGAAACACGUCCCUGUCAGGGC..........................................................................................................................    12.602163
.......................................AAGAAACACGUCCCUGUCAGGG...........................................................................................................................    1220.483713
.......................................AAGAAACACGUCCCUGUCAG.............................................................................................................................    16.942641
.......................................AAGAAACACGUCCCUGUCAGG............................................................................................................................    33.588128
.......................................AAGAAACACGUCUCUGUCAGG............................................................................................................................    18.79467
.......................................AAGAAACACGUCCCUGUCAGGA...........................................................................................................................    1.143577
.......................................AAGAAACACGUCUCUGUCAGGG...........................................................................................................................    152.517373
.......................................AAGAAACACGUCCCUGUCAGGGU..........................................................................................................................    11.350169
.......................................AAGAAACACGUCCCUGUCAGGGA..........................................................................................................................    8.50233
.......................................AAGAAACACGUCCCUGUCAGGGCU.........................................................................................................................    1.63442
.......................................AAGAAACACGUCCCUGUCAGGU...........................................................................................................................    2.14028
.......................................AAGAAACACGUCUCUGUCAG.............................................................................................................................    2.79247
........................................AGAAACACGUCCCUGUCAGGG...........................................................................................................................    90.719586
........................................AGAAACACUUCCCUGUCAGGGC..........................................................................................................................    1.15457
........................................AGAAACACGUCUCUGUCAGGG...........................................................................................................................    1.65256
........................................AGAAACACGUCUCUGUCAGGGC..........................................................................................................................    26.83332
........................................AGAAACACGUCCCUGUCAGGGU..........................................................................................................................    0.883101
........................................AGAAACACGUCCCUGUCAGGGG..........................................................................................................................    3.44287
........................................AGAAACACGUCCCUGUCAGGGA..........................................................................................................................    4.44317
.........................................GAAACACGUCCCUGUCAGGGC..........................................................................................................................    13.967182
.........................................GAAACACGUCCCUGUCAGGGCG.........................................................................................................................    11.679002
..........................................AAACACGUCCCUGUCAGGG...........................................................................................................................    60.951838
..........................................AAACACGUCCCUGUCAGGGCGC........................................................................................................................    438.236256
..........................................AAACACGUCCCUGUCAGGGCG.........................................................................................................................    63.055644
..........................................AAACACGUCCCUGUCAGGGC..........................................................................................................................    201.55589
..........................................AAACACGUCCCUGUCAGG............................................................................................................................    3.68569
..........................................AAACACGUCUCUGUCAGGG...........................................................................................................................    5.81736
..........................................AAACACGUCUCUGUCAGGGC..........................................................................................................................    1.93229
..........................................AAACACGUCUCUGUCAGG............................................................................................................................    10.78093
..........................................AAACACGUCCCUGUCAGGGCU.........................................................................................................................    1.44198
..........................................AAACACGGCCCUGUCAGGGCGC........................................................................................................................    1.29681
..........................................AAACACGUCCCUGUCAGGGA..........................................................................................................................    0.75184
..........................................AAACACGUCUCUGUCAGGGCGC........................................................................................................................    69.98312
...........................................AACACGUCCCUGUCAGGGCG.........................................................................................................................    1.367692
...........................................AACACGUCCCUGUCAGGGCGCA.......................................................................................................................    6.091407
...........................................AACACGUCCCUGUCAGGGCGC........................................................................................................................    108.622919
...........................................AACACGUCCCUGUCAGGGC..........................................................................................................................    12.647964
...........................................AACACGUCCCUGUCAGGG...........................................................................................................................    3.842412
...........................................AACACGUCUCUGUCAGGGC..........................................................................................................................    9.64821
...........................................AACACGUCCCUGUCAGGA...........................................................................................................................    0.318481
...........................................AACACGUCUCUGUCAGGG...........................................................................................................................    9.33108
...........................................AACACGUCUCUGUCAGGGCGC........................................................................................................................    52.83705
............................................................................................................................................CGCCCUGACAGGGACGUGUUC.......................    1.81977
.............................................................................................................................................GCCCUGACAGGGACGUGUUCUU.....................    0.17779
.............................................................................................................................................GCCCUGACAGGGACGUGUUC.......................    32.815458
.............................................................................................................................................GCCCUGACAGGGACGUGUUCU......................    591.159713
.............................................................................................................................................GCCCUGACAGGGCCGUGUUCU......................    2.749404
.............................................................................................................................................GCCCUGACAGGGACGUGUUCC......................    1.55963
..............................................................................................................................................CCCUGACAGGGACGUGUUCU......................    0.651837
..............................................................................................................................................CCCUGACAGGGACGUGUUCUU.....................    4.788762
...............................................................................................................................................CCUGACAGGGACGUGUUCU......................    1.45641
................................................................................................................................................CUGACAGGGACGUGUUCUUCC...................    851.341765
................................................................................................................................................CUGACAGGGACGUGUUCU......................    45.121237
................................................................................................................................................CUGACAGGGACGUGUUCUUC....................    36.621546
................................................................................................................................................CUGACAGGGACGUGUUCUUCA...................    23.342188
................................................................................................................................................CUGACAGGGACGUGUUCUUCU...................    86.789849
.................................................................................................................................................UGACAGGGACGUGUUCUUCC...................    26.242469
.................................................................................................................................................UGACAGGGACGUGUUCUUCCU..................    2.805443
.................................................................................................................................................UGACAGGGACGUGUUCUUCU...................    1.23482
..................................................................................................................................................GACAGGGACGUGUUCUUCCUC.................    9.32841
```

```
                                Mature                                                          Star
GAGAGAUUUUGAUAUUUAGGACUCAAUUUGCAUGCCUUUCGAGAUUUGACACCCCUAUCACAGUGACAUGUGGGGUCAUUGACAUGUGGCCAGAGUGUCAAAUCUUGAAAGGCAUGCGAAUUGGGUCCCAAAAUCUUAAAUUUCU    698.4
.((((((((.(((.(((.((((((((((((((((((((((((((((((((((.....(((((......)))))((((((......))))))...)))))))))))))))))))))))))))))))))).)))))).))))))))
................................UGCCUUUCGAGAUUUGACACC............................................................................................    278.713982
...............................................................................................UGUCAAAUCUUGAAAGGCAUG.............................    3.319785
................................................................................................GUCAAAUCUUGAAAGGCAUGCG...........................    0.9455
.................................................................................................UCAAAUCUUGAAAGGCAUGCG...........................    1.15179
```

zma-MIR_N159a

```
Chromosome:    chr7
Start:         25824495
End:           25824639
Strand:        -
Strand bias:   1.0
Abundance bias:0.826
MFE:           -76.10
```

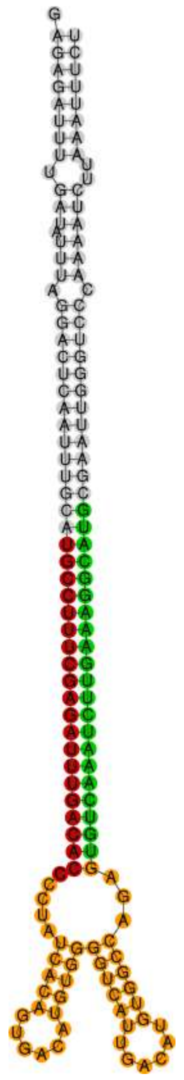

zma-MIR_N169a

```
Chromosome:    chr8
Start:         16008016
End:           16008159
Strand:        +
Strand bias:   1.0
Abundance bias:0.991
MFE:           -82.90
```

```
                    Star                                                                             Mature
CGUCGUCGUGGUAUUUGGCGCUGACCUCCUUUCCUAGAAACGGCUGCUCGGCCUCAUCCUUCAUUGACACACACACACACACCACUCAUUGAGGCCGAGCGACCGUUCUUAGGAAAAGAGGUCGGCACCAAUGACCACGACGCC    410.
.(.(((((((((..((((.(((((((((.((((((((.(((((.((((((((((((.................................)))))))))))).))))).)))))))).))))))))).))))..)))))))))).
.........................................................................................................UUCUUAGGAAAAGAGGUCGGC..................    389.973188
.....................UGACCUCCUUUCCUAGAAACG......................................................................................................    8.80656
..........................................................................................................UCUUAGGAAAAGAGGUCGGC..................    0.53258
..........................................................................................................UCUUAGGAAAAGAGGUCGGCA.................    1.077049
.........................................................................................................UUCUUAGGAAAAGAGGUCGGCA.................    0.212795
.........................................................................................................UUCUUAGGAAAAGAGGUCGGCACC...............    2.114843
.....................UGACCUCCUUUCCUAGAAAC.......................................................................................................    3.6694
.....................UGACCUCCUUUCCUAGAAACU......................................................................................................    0.427815
```

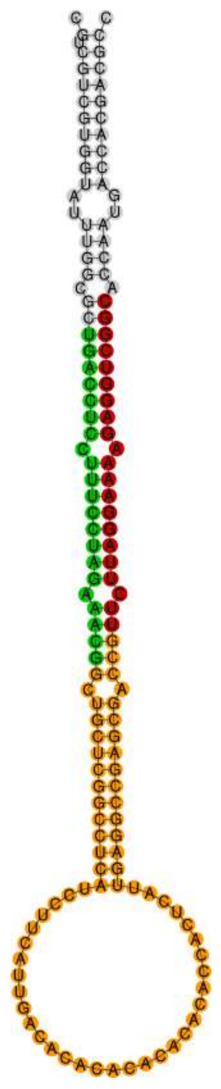

Star Mature

```
AAGGGCCUGUUUGGAUUUUCUCCUCUAAGCUUUAUAGCUCUAAACUUUAGAGAUAAAGCUUUAAGCAAGUGUUCUGAGGUUACCUCUCUAAACUCUAGACCCACCUCAUAUUAGAGCUCAAAAGUGUGCUAAAGUGCUCUAAAGCUUUUAGAGCUCUAAAGUUUGGAGGGGAAAACUGAAAGGGAAAUGUGCCCUUG 63.9
(((((((.(((.(.(((((((((((((((((((.((((((((((((((((.....((((((.(((...(((((((((......((((.....))))...)))))).....)))))).........))))))))).))))))))..))))))))).))))))))))))))))))).).))).))......)))))).
..........................................................................................................................................................UCUAAAGUUUGGAGGGGAAAA...................... 33.18755
.................UUCUCCUCUAAGCUUUAUAGC............................................................................................................................................................... 30.80298
```

zma-MIR_N180a

Chromosome: chr8
Start: 177143419
End: 177143615
Strand: +
Strand bias: 1.0
Abundance bias: 1.0
MFE: -72.30

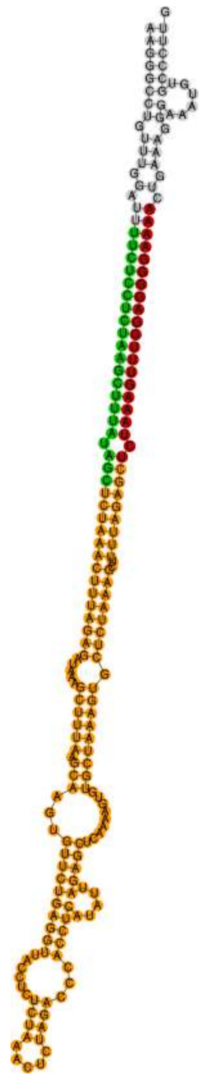

zma-MIR_N173a

Chromosome: chr8
Start: 105396473
End: 105396587
Strand: -
Strand bias: 0.998
Abundance bias: 0.999
MFE: -69.20

```
                  Mature                                                              Star
ACCAUUGCUGCUACUAUGUCCAAGUCAAUGCAUCAUUGCCCCUAGUUUUGCCAUCAAAGAAGGCGAAGGCAGGGGCAAUGGUGCAUUGACUUGGUCAAAUCAGGGGGGCAAUGAU    591.2
..(((((((.((....((.((((((((((((((((((((((((..(((((((.((...)).)))))))..)))))))))))))))))))))))).))......)).)))))))..
..................UCCAAGUCAAUGCAUCAUUGC............................................................................    249.587616
............................................................................AAUGGUGCAUUGACUUGGUCA..................    74.101873
................UGUCCAAGUCAAUGCAUCAUU..............................................................................    7.986942
.................GUCCAAGUCAAUGCAUCAUUG.............................................................................    8.226494
..................UCCAAGUCAAUGCAUCAUUG.............................................................................    1.260953
....................CAAGUCAAUGCAUCAUUGC............................................................................    0.709268
............................................................................AAUGGUGCAUUGACUUGGUC...................    66.489915
............................................................................AAUGGUGCAUUGACUUGGCCA..................    5.59742
.............................................................................AUGGUGCAUUGACUUGGUCAA.................    124.546019
.............................................................................AUGGUGCAUUGACUUGGUCA..................    39.228425
.............................................................................AUGGUGCAUUGACUUGGUC...................    12.916865
```

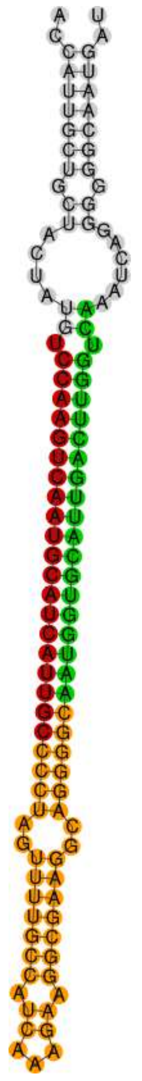

```
                                               Mature                                                                                                                          Star
AUUUGGAAUAAAGUUUUUUUUCCUGAAUCUGCGUUUUUCCUGUGUUUUUUAACUAAUUCCUGUAAGGGCUUCUAUGAAACACAAGAAUUUUAUAGAAAUGGUAUAGUAAUUUAACAGGAAUUAGUUCAAAAACACAUGAAAAACACAGGAUUCAGAAAAUUUUCCUUGU    490.9
....((((...((((((.....(((((((((.(((((((.(((((((((.((((((((((((((((.((((((((((((........)))))))))........)))..))).))))))))))))).))))))))).))))))).)).)))))))))))))))))....
...........................UCUGCGUUUUUCCUGUGUUUU.........................................................................................................................    250.666382
..................................................................................................................................AACACAUGAAAAACACAGGAUU.................    128.350237
.................................................................................................................................AAACACAUGAAAAACACAGGA...................    0.883101
..................................................................................................................................AACACAUGAAAAACACAGGAU..................    109.584497
..................................................................................................................................AACACAUGAAACACACAGGAUU.................    0.703221
..................................................................................................................................AACACAUGAAAACCACAGGAUU.................    0.756944
```

zma-MIR_N174a

Chromosome:	chr8
Start:	111488442
End:	111488610
Strand:	-
Strand bias:	1.0
Abundance bias:	1.0
MFE:	-55.30

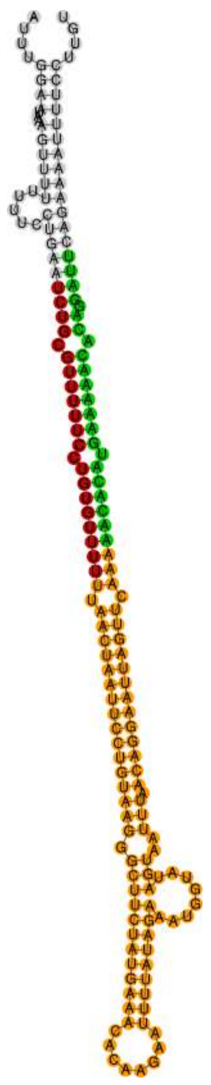

zma-MIR_N8b

Chromosome: chr10
Start: 60227109
End: 60227254
Strand: -
Strand bias: 1.0
Abundance bias: 0.967
MFE: -99.40

```
                             Mature                                                                                                                           Star
CGACUUUAAGGGCUUGUUUGGGACCAAGUGAAAUGGAGAGAAUUGAGGGGGCUAUAAUCCCUUGUUAUUCAAAAUUGAAUAGUAAGGGAUUAUAGCCCCCUCAAUUCUCUUUGUUCCACUUACUCCCAAACAAGCUCUAAGGGUUU    1362.5
.((((((.(((((((((((((((..(((((.((((((((((((((((((((((((((((((((((((((((....)))))))))))))))))))))))))))))))))))))))).)))))..))))))))))))))).)))))).
..................UGGGACCAAGUGAAAUGGAGA..................................................................................................................    1207.691177
..........................................................................................................UUUGUUCCACUUACUCCCAAA................    13.593481
...............GUUUGGGACCAAGUGAAAUGGAGA...........................................................................................................    0.492907
.................UUGGGACCAAGUGAAAUGGAG............................................................................................................    2.19298
..................UGGGACCAAGUGAAAUGG..............................................................................................................    37.68304
..................UGGGACCAAGUGAAAUGGAG............................................................................................................    23.92118
..................UGGGACCAAGUGAAAUGGAGG...........................................................................................................    14.88467
..................UGGGACCAAGUGAAAUGGAGU...........................................................................................................    7.583595
..................UGGGACCAAGUGAAAUAGAGA...........................................................................................................    9.89436
```

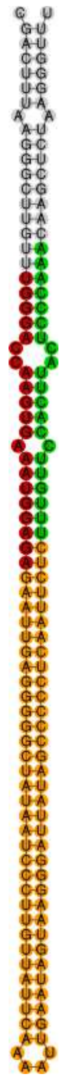

zma-MIR_N8c

Chromosome: chr10
Start: 60808576
End: 60808723
Strand: -
Strand bias: 1.0
Abundance bias: 0.973
MFE: -92.70

```
                         Mature                                                                                              Star
AAACGUGAAGGGCUUGUUUGGGACCAAGUGAAAUGGAGAGAAUUGAGGGGGCUAUAAUCCCUUGUUAUUCAAAAUUGAAUAGCAAAGGAUUAUAGCCCCUUCAAUUCUCUUUGUUCCACUUACUCCCAAAUAAGCCCGAAAGGGUUUU    1354.9
((((.(...((((((((((((((..(((((.((((((((((((((((((((((((((((.((((((((((....)))))))))).))))))))))))))))))))))))))))).))))).)))))..))))))))))))))....).)))).
..................UGGGACCAAGUGAAAUGGAGA.................................................................................................................    1207.691177
..............................................................................................................UUUGUUCCACUUACUCCCAAA.................    13.593481
...............GUUUGGGACCAAGUGAAAUGGAGA.................................................................................................................    0.492907
.................UUGGGACCAAGUGAAAUGGAG..................................................................................................................    2.19298
..................UGGGACCAAGUGAAAUGG....................................................................................................................    37.68304
..................UGGGACCAAGUGAAAUGGAG..................................................................................................................    23.92118
..................UGGGACCAAGUGAAAUGGAGG.................................................................................................................    14.88467
..................UGGGACCAAGUGAAAUGGAGU.................................................................................................................    7.583595
..................UGGGACCAAGUGAAAUAGAGA.................................................................................................................    9.89436
```

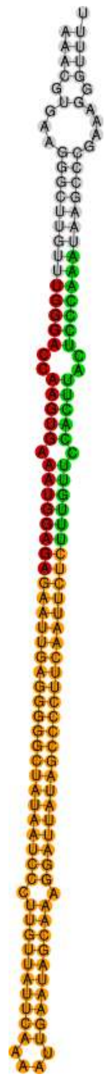

zma-MIR_N8d

Chromosome: chr2
Start: 192385197
End: 192385340
Strand: +
Strand bias: 1.0
Abundance bias: 0.968
MFE: -94.50

Mature Star

```
UACGCUAAGGGCUUGUUUGGGACCAAGUGAAAUGGAGAGAAUUGAGGGGACUAUAAUCCCUUGUUAUUUAAAAUUGAAUAGCAAGGGAUUAUAGCCCCUCAAUUCUCUUUGUUCCACUUACUCCCAAACAAGCCCUAAGACUA    1361.8
....((.(((((((((((((((..(((((.(((((((((((((((((((.((((((((((((((((((((....)))))))))))))))))))).))))))))))))))))))).)))))..))))))))))))))).))....
.................UGGGACCAAGUGAAAUGGAGA...................................................................................................    1207.691177
.........................................................................................................UUUGUUCCACUUACUCCCAAA..............    13.593481
..............GUUUGGGACCAAGUGAAAUGGAGA...................................................................................................    0.492907
................UUGGGACCAAGUGAAAUGGAG....................................................................................................    2.19298
.................UGGGACCAAGUGAAAUGG......................................................................................................    37.68304
.................UGGGACCAAGUGAAAUGGAG....................................................................................................    23.92118
.................UGGGACCAAGUGAAAUGGAGG...................................................................................................    14.88467
.................UGGGACCAAGUGAAAUGGAGU...................................................................................................    7.583595
.................UGGGACCAAGUGAAAUAGAGA...................................................................................................    9.89436
```

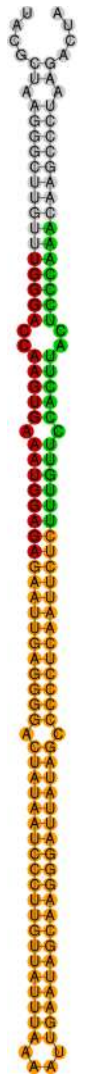

```
                  Mature                                                                                Star
GACACUUAGGGCUUGUUUGGGACCAAGUGAAAUGGAGAGAAUUGAGGGGGCUAUUAUCCCUUGUUAUUCAAAAUUGAAUAGCAAGGGAUUAUAGCCCCCUCAAUUCUCUUUGUUCCACUUACUCCCAAACAAGCUCUUAGGGUA     1359.8
.((.((((((((((((((((((..(((((.((((((((((((((((((((((((.(((((((((((((((....))))))))))))))).)))))))))))))))))))))))).)))))..)))))))))))).)))))))).
.................UGGGACCAAGUGAAAUGGAGA..........................................................................................................     1207.691177
............................................................................................................UUUGUUCCACUUACUCCCAAA...............     13.593481
..............GUUUGGGACCAAGUGAAAUGGAGA..........................................................................................................     0.492907
...............UUGGGACCAAGUGAAAUGGAG............................................................................................................     2.19298
.................UGGGACCAAGUGAAAUGG.............................................................................................................     37.68304
.................UGGGACCAAGUGAAAUGGAG...........................................................................................................     23.92118
.................UGGGACCAAGUGAAAUGGAGG..........................................................................................................     14.88467
.................UGGGACCAAGUGAAAUGGAGU..........................................................................................................     7.583595
.................UGGGACCAAGUGAAAUAGAGA..........................................................................................................     9.89436
```

zma-MIR_N8a

```
Chromosome:    chr6
Start:         135561318
End:           135561461
Strand:        +
Strand bias:   1.0
Abundance bias:0.969
MFE:           -98.30
```

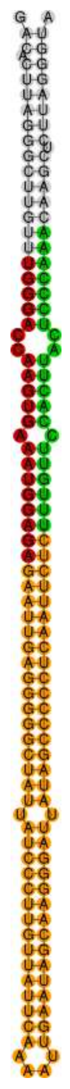

```
                                   Star                                                                                                                                            Mature
GCUCUUGUGCUCAUAUGAGUACUUUAAAUCUGGUGCUUUUAUUUCUCAUCCAGUGGCUUAAGGCAGCAGUGGCACACAUAAAAGACAGGUGACGCAACAAGGGUAGUACUAAGAAUAAACUAGGAAAUGUCUAUUUACAUUUAGAUGAGAAAUGAAUACACCAGAUUUAAAGUGCUCAUAUGAGCAUAAGAG   60.
.((((((((((((((((((((((((((((((((..(((((((((((((..((((((...(....)...))).)))..........((((((.(.....).))..)))))..............((((((......)))))).))))))))))))..))))))))))))))))))))))))))))))))
..........................................................................................................................................................AAUACACCAGAUUUAAAGUGC.................   32.745634
....................ACUUUAAAUCUGGUGCUUUUA.......................................................................................................................................................   14.3107
```

zma-MIR_N176a

Chromosome: chr8
Start: 128419328
End: 128419519
Strand: -
Strand bias: 1.0
Abundance bias:0.775
MFE: -83.30

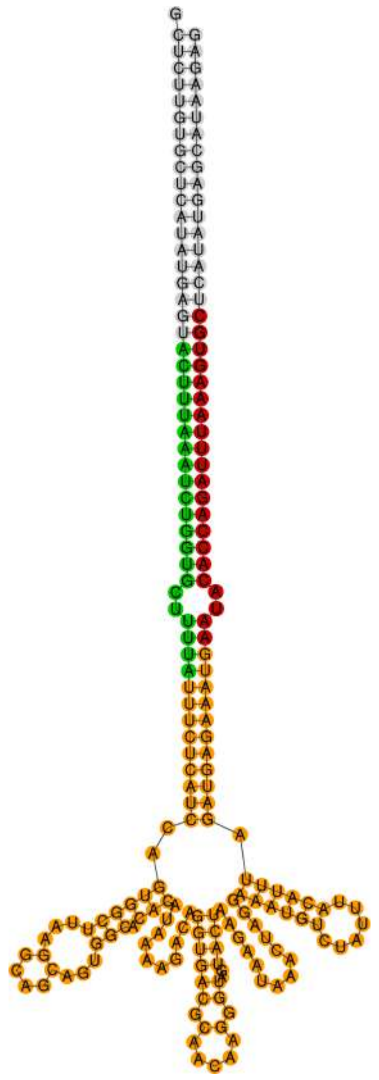

```
                                                                      Star                                    Mature
UGCCAUGAUGUCGUCCCUUUCACCUCCCACUAAGUACCCCCUCUUAAGAAGAGAGGGUACAUGGUCAUUAGAAGUAUUUUCUGGUCUUCAAAUGACCAUGUACCCUCUCUUCUUAAGUUAUAAAAUGGU    66.0
.((((((((...)))...................................(((((((((((((((((((((((.((((.(((....))))))).))))))))))))))))))))))).......)))))
...........................................................................................AUGACCAUGUACCCUCUCUUC.................    60.82251
.................................................AGAGAGGGUACAUGGUCAUUA...........................................................    5.19498
```

zma-MIR_N178a

Chromosome:	chr8
Start:	154460923
End:	154461051
Strand:	-
Strand bias:	1.0
Abundance bias:	1.0
MFE:	-53.40

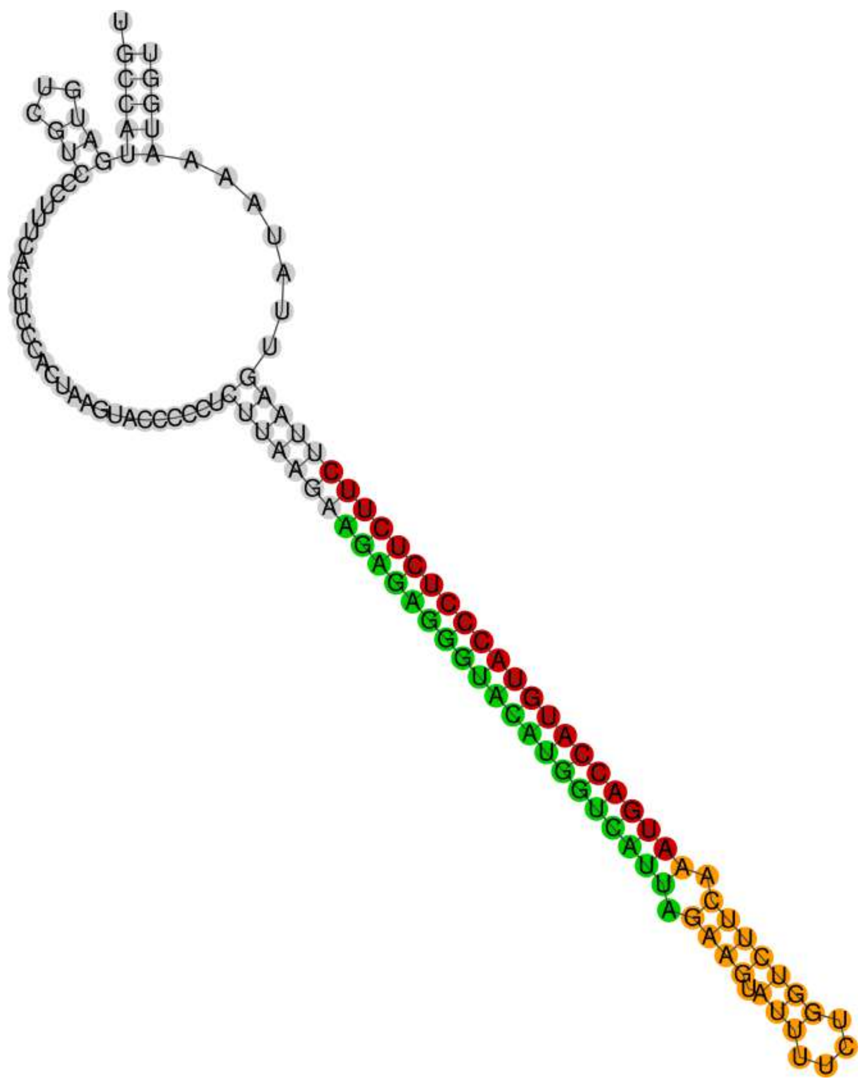

zma-MIR_N9c

```
Chromosome:    chr7
Start:         151540385
End:           151540527
Strand:        +
Strand bias:   1.0
Abundance bias:0.947
MFE:           -84.90
```

```
                      Star                                                         Mature
AGAUGGCCGGCCUGAGCCGCCUCGCAUCCCCAUCCCGCCGACGUGCGCGUCCCUUCGGGUGGGCGCGCCGUCGUCGGAUGGGUGCUGCGCGGUGGUCGAGGCCCAGUCCGACGAGUUAAGAACUGCGGCACUGUUAGCCGUCG    16974.4
.((((((.(((((..((((((.((((.((((((((.(.(((((.((((((((((...)).))))))))))))).)))))))).).)))).))))))..)))))(((((((.(.((((...)))))))).))))...)))))).
..........................................................................CGGAUGGGUGCUGCGCGGUGGU...............................................    12878.34773
.................CGCCUCGCAUCCCCAUCCCGCC........................................................................................................    717.822586
.............................................................................AUGGGUGCUGCGCGGUGGU...............................................    6.654518
.............................................................................AUGGGUGCUGCGCGGUGGUA..............................................    3.185643
............................................................................GAUGGGUGCUGCGCGGUGGU...............................................    8.632153
...........................................................................GGAUGGGUGCUGCGCGGUGG................................................    2.43913
...........................................................................GGAUGGGUGCUGCGCGGUGGU...............................................    184.261289
...........................................................................GGAUGGGUGCUGCGCGGUGGUA..............................................    0.357231
..........................................................................CGGAUGGGUGCUGCGCGGUGG................................................    660.005286
..........................................................................CGGAUGGGUGCUGCGCGGUG.................................................    108.337808
..........................................................................CGGAUGGGUGCUGCGCGGU..................................................    113.650154
..........................................................................CGGAUGGGUGCUGCGCGGUGGUC..............................................    4.677659
..........................................................................CGGAUGGGUUCUGCGCGGUGGU...............................................    4.277825
..........................................................................CGGAUGGGUGCUGUGCGGUGGU...............................................    0.364792
..........................................................................CGGAUGGGUGCUGCGAGGUGGU...............................................    2.408794
..........................................................................CGGAUGCGUGCUGCGCGGUGGU...............................................    0.61377
..........................................................................CGGAUGGGUGCUGCGCGGCGGU...............................................    6.841739
..........................................................................CGGAUGGGUGCUGCGCGGGGGU...............................................    7.761097
..........................................................................CUGAUGGGUGCUGCGCGGUGGU...............................................    0.925992
..........................................................................CGGCUGGGUGCUGCGCGGUGGU...............................................    6.0609
..........................................................................CGGAUGGGCGCUGCGCGGUGGU...............................................    7.194178
..........................................................................CGGGUGGGUGCUGCGCGGUGGU...............................................    2.696226
..........................................................................CGGAUGGGUGCUGCGCGGUGGG...............................................    34.229152
..........................................................................CGGAUGGGUGCUGCGCGGUGGC...............................................    242.879807
..........................................................................CGGAUGGGUGCUGCGCGGUGGA...............................................    73.943594
..........................................................................CGGAUGGGUGCUGCGCGGUGGUA..............................................    143.189313
..........................................................................CGGAUGGGUGCUGCGCGGUGGUG..............................................    19.756879
..........................................................................CGGAUGGGAGCUGCGCGGUGGU...............................................    0.182396
..........................................................................CGGAUGGGGGCUGCGCGGUGGU...............................................    0.518993
..........................................................................CGGAGGGGUGCUGCGCGGUGGU...............................................    0.212795
..........................................................................CGGAUGGUUGCUGCGCGGUGGU...............................................    3.961628
..........................................................................CGGAUGGGUGCUGCGCUGUGGU...............................................    2.774629
..........................................................................CGGAUGGGUGCUGCGCGGUGCU...............................................    2.88453
..........................................................................CGGAUGGGUGCUGCGCGGAGGU...............................................    0.273594
..........................................................................CGGACGGGUGCUGCGCGGUGGU...............................................    1.35015
..........................................................................CGGAUGGGUGUUGCGCGGUGGU...............................................    1.54303
..........................................................................AGGAUGGGUGCUGCGCGGUGGU...............................................    1.99826
..........................................................................CGGAUGGGUGCUGCGCGGC..................................................    2.86013
..........................................................................CGGAUGUGUGCUGCGCGGUGGU...............................................    2.235245
..........................................................................CGGAUGGGUACUGCGCGGUGGU...............................................    2.88453
..........................................................................CGGAUGGGUGCUGCGUGGUGGU...............................................    2.663121
..........................................................................CGGAUGGGUGCUGCGCGGUGUU...............................................    5.706205
..........................................................................CGGAUGGGUGCUUCGCGGUGGU...............................................    0.243195
..........................................................................CGGAUGGGUCCUGCGCGGUGGU...............................................    3.448356
..........................................................................CGGAUGAGUGCUGCGCGGUGGU...............................................    2.335495
..........................................................................CGGAUGGGUGCUGCGCGGUGAU...............................................    5.192466
..........................................................................UGGAUGGGUGCUGCGCGGUGGU...............................................    1.15727
..........................................................................CGUAUGGGUGCUGCGCGGUGGU...............................................    1.01025
.........................................................................UCGGAUGGGUGCUGCGCGGU..................................................    3.246275
.........................................................................UCGGAUGGGUGCUGCGCGGUGGU...............................................    212.815899
.........................................................................UCGGAUGGGUGCUGCGCGGUGG................................................    236.790315
.........................................................................UCGGAUGGGUGCUGCGCGGUGGC...............................................    0.884046
.........................................................................UCGGAUGGGUGCUGCGCGGUGGA...............................................    1.382145
.........................................................................UCGGAUGGGUGCUGCGCGGUGGUA..............................................    1.128082
........................................................................GUCGGAUGGGUGCUGCGCGGUGGUA..............................................    0.364792
..................GCCUCGCAUCCCCAUCCCGC.........................................................................................................    0.371561
..................GCCUCGCAUCCCCAUCCC...........................................................................................................    0.318481
..................GCCUCGCAUCCCCAUCCCGCC........................................................................................................    16.580858
.................CGCCUCGCAUCCCCAUCCCGC.........................................................................................................    270.28578
.................CGCCUCGCAUCCCCAUCCCG..........................................................................................................    11.664853
.................CGCCUCGCAUCCCCAUCCC...........................................................................................................    0.477722
.................CGCCUCGCAUCCCCAUCCCGCU........................................................................................................    37.647572
```

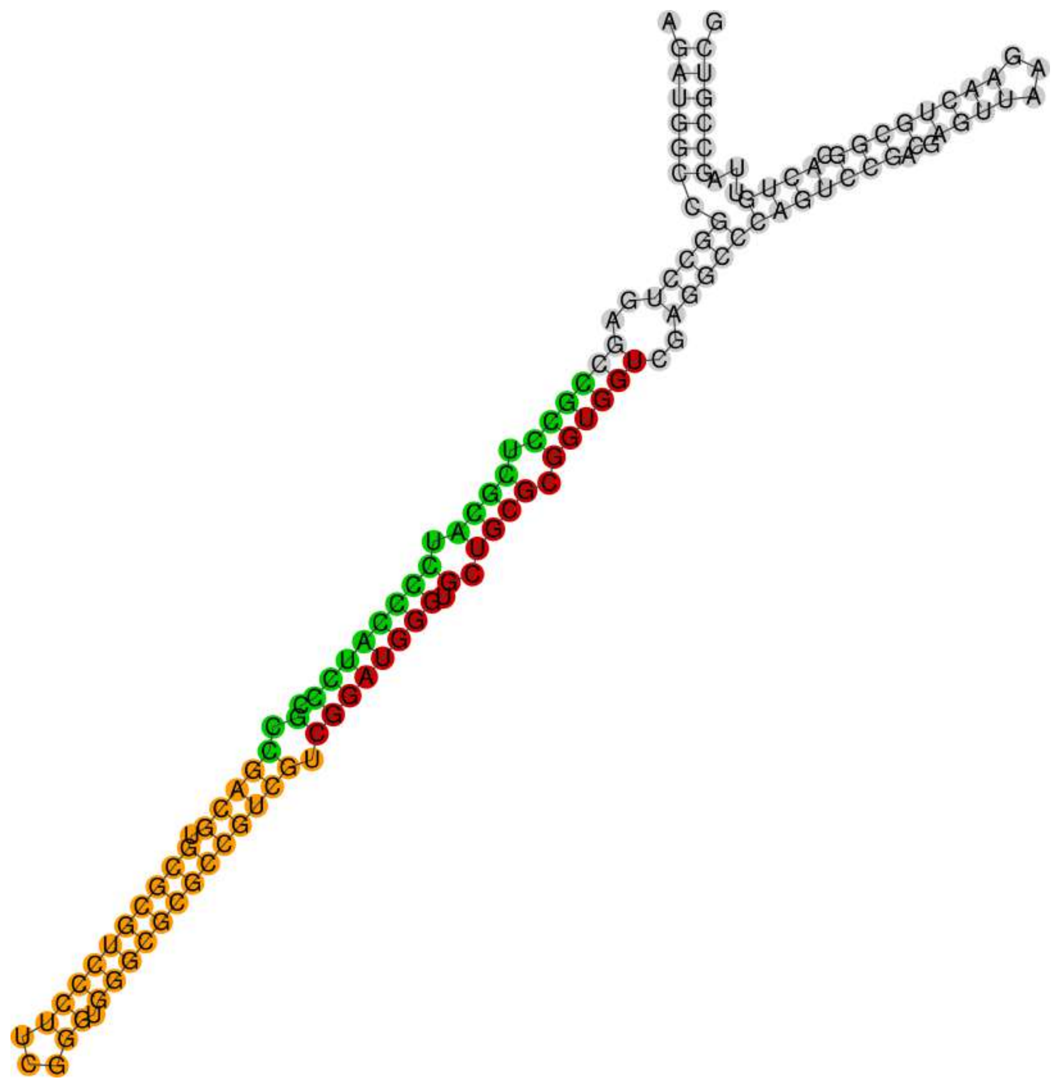

zma-MIR_N9d

Chromosome: chr1
Start: 274817468
End: 274817610
Strand: +
Strand bias: 1.0
Abundance bias: 0.958
MFE: -86.90

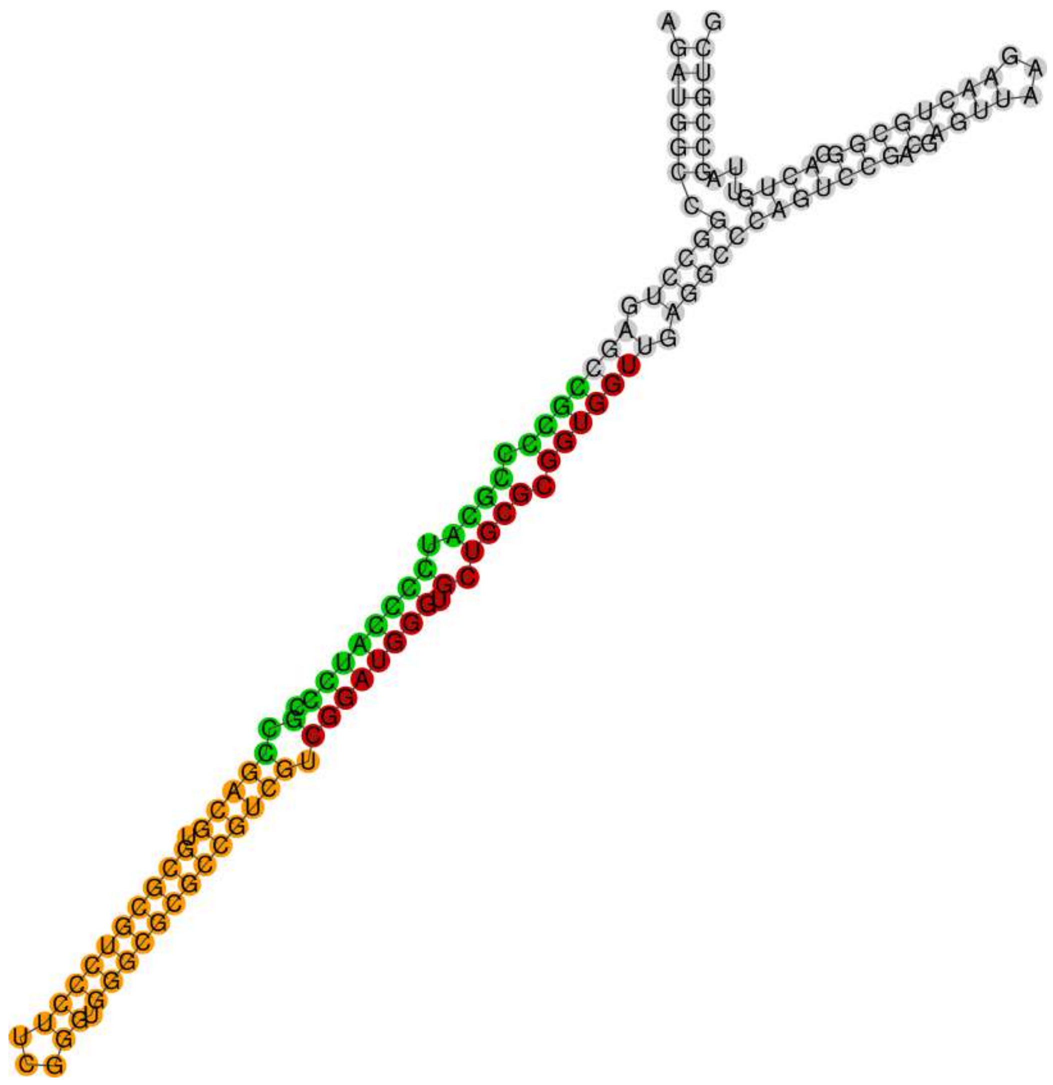

```
                           Star                                                   Mature
AGAUGGCCGGCCUGAGCCGCCCCGCAUCCCCAUCCCGCCGACGUGCGCGUCCCUUCGGGUGGGCGCGCCGUCGUCGGAUGGGUGCUGCGCGGUGGUUGAGGCCCAGUCCGACGAGUUAAGAACUGCGGCACUGUUAGCCGUCG    21501.0
.((((((.(((((.(((((((.((((.((((((((.(.(((((.((((((((((...)).)))))))))))).)))))))).).)))).))))))).)))))((((((.(.((((...)))))))).))))...)))))).
.........................................................................CGGAUGGGUGCUGCGCGGUGGU................................................    12878.34773
.................CGCCCCGCAUCCCCAUCCCGCC........................................................................................................    2717.053301
............................................................................AUGGGUGCUGCGCGGUGGU................................................    6.654518
............................................................................AUGGGUGCUGCGCGGUGGUU...............................................    1.562945
............................................................................AUGGGUGCUGCGCGGUGGUUG..............................................    3.542156
............................................................................AUGGGUGCUGCGCGGUGGUUGA.............................................    0.759983
............................................................................AUGGGUGCUGCGCGGUGGUA...............................................    3.185643
...........................................................................GAUGGGUGCUGCGCGGUGGU................................................    8.632153
...........................................................................GAUGGGUGCUGCGCGGUGGUU...............................................    0.306198
..........................................................................GGAUGGGUGCUGCGCGGUGG.................................................    2.43913
..........................................................................GGAUGGGUGCUGCGCGGUGGU................................................    184.261289
..........................................................................GGAUGGGUGCUGCGCGGUGGUA...............................................    0.357231
.........................................................................CGGAUGGGUGCUGCGCGGUGG.................................................    660.005286
.........................................................................CGGAUGGGUGCUGCGCGGUGGUU...............................................    39.78851
.........................................................................CGGAUGGGUGCUGCGCGGUG..................................................    108.337808
.........................................................................CGGAUGGGUGCUGCGCGGU...................................................    113.650154
.........................................................................CGGAUGGGUUCUGCGCGGUGGU................................................    4.277825
.........................................................................CGGAUGGGUGCUGUGCGGUGGU................................................    0.364792
.........................................................................CGGAUGGGUGCUGCGAGGUGGU................................................    2.408794
.........................................................................CGGAUGCGUGCUGCGCGGUGGU................................................    0.61377
.........................................................................CGGAUGGGUGCUGCGCGGCGGU................................................    6.841739
.........................................................................CGGAUGGGUGCUGCGCGGGGGU................................................    7.761097
.........................................................................CUGAUGGGUGCUGCGCGGUGGU................................................    0.925992
.........................................................................CGGCUGGGUGCUGCGCGGUGGU................................................    6.0609
.........................................................................CGGAUGGGCGCUGCGCGGUGGU................................................    7.194178
.........................................................................CGGGUGGGUGCUGCGCGGUGGU................................................    2.696226
.........................................................................CGGAUGGGUGCUGCGCGGUGGG................................................    34.229152
.........................................................................CGGAUGGGUGCUGCGCGGUGGC................................................    242.879807
.........................................................................CGGAUGGGUGCUGCGCGGUGGA................................................    73.943594
.........................................................................CGGAUGGGUGCUGCGCGGUGGUA...............................................    143.189313
.........................................................................CGGAUGGGUGCUGCGCGGUGGUG...............................................    19.756879
.........................................................................CGGAUGGGAGCUGCGCGGUGGU................................................    0.182396
.........................................................................CGGAUGGGGGCUGCGCGGUGGU................................................    0.518993
.........................................................................CGGAGGGGUGCUGCGCGGUGGU................................................    0.212795
.........................................................................CGGAUGGUUGCUGCGCGGUGGU................................................    3.961628
.........................................................................CGGAUGGGUGCUGCGCUGUGGU................................................    2.774629
.........................................................................CGGAUGGGUGCUGCGCGGUGCU................................................    2.88453
.........................................................................CGGAUGGGUGCUGCGCGGAGGU................................................    0.273594
.........................................................................CGGACGGGUGCUGCGCGGUGGU................................................    1.35015
.........................................................................CGGAUGGGUGUUGCGCGGUGGU................................................    1.54303
.........................................................................AGGAUGGGUGCUGCGCGGUGGU................................................    1.99826
.........................................................................CGGAUGGGUGCUGCGCGGC...................................................    2.86013
.........................................................................CGGAUGUGUGCUGCGCGGUGGU................................................    2.235245
.........................................................................CGGAUGGGUACUGCGCGGUGGU................................................    2.88453
.........................................................................CGGAUGGGUGCUGCGUGGUGGU................................................    2.663121
.........................................................................CGGAUGGGUGCUGCGCGGUGUU................................................    5.706205
.........................................................................CGGAUGGGUGCUUCGCGGUGGU................................................    0.243195
.........................................................................CGGAUGGGUCCUGCGCGGUGGU................................................    3.448356
.........................................................................CGGAUGAGUGCUGCGCGGUGGU................................................    2.335495
.........................................................................CGGAUGGGUGCUGCGCGGUGAU................................................    5.192466
.........................................................................UGGAUGGGUGCUGCGCGGUGGU................................................    1.15727
.........................................................................CGUAUGGGUGCUGCGCGGUGGU................................................    1.01025
........................................................................UCGGAUGGGUGCUGCGCGGU...................................................    3.246275
........................................................................UCGGAUGGGUGCUGCGCGGUGGU................................................    212.815899
........................................................................UCGGAUGGGUGCUGCGCGGUGGUU...............................................    1.932477
........................................................................UCGGAUGGGUGCUGCGCGGUGG.................................................    236.790315
........................................................................UCGGAUGGGUGCUGCGCGGUGGC................................................    0.884046
........................................................................UCGGAUGGGUGCUGCGCGGUGGA................................................    1.382145
........................................................................UCGGAUGGGUGCUGCGCGGUGGUA...............................................    1.128082
.......................................................................GUCGGAUGGGUGCUGCGCGGUGGUA...............................................    0.364792
..................GCCCCGCAUCCCCAUCCCGC.........................................................................................................    3.658304
..................GCCCCGCAUCCCCAUCCCGCC........................................................................................................    5.679577
.................CGCCCCGCAUCCCCAUCCCGC.........................................................................................................    1863.93701
.................CGCCCCGCAUCCCCAUCCCGCCG.......................................................................................................    0.319396
.................CGCCCCGCAUCCCCAUCCCG..........................................................................................................    180.272163
.................CGCCCCGCAUCCCCAUCCC...........................................................................................................    12.439371
.................CGCCCCGCAUCCCCAUCCCGCCU.......................................................................................................    7.473374
.................CGCCCCGCAUCCCCAUCCCU..........................................................................................................    13.098666
.................CGCCCCGCAUCCCCAUCCCGA.........................................................................................................    1.579001
.................CGCCCCGCAUCCCCAUCCCGU.........................................................................................................    14.289954
.................CGCCCCGCAUCCCCAUCCCGCU........................................................................................................    718.774882
```

zma-MIR_N9a

Chromosome: chr4
Start: 237151393
End: 237151535
Strand: +
Strand bias: 1.0
Abundance bias: 0.947
MFE: -84.90

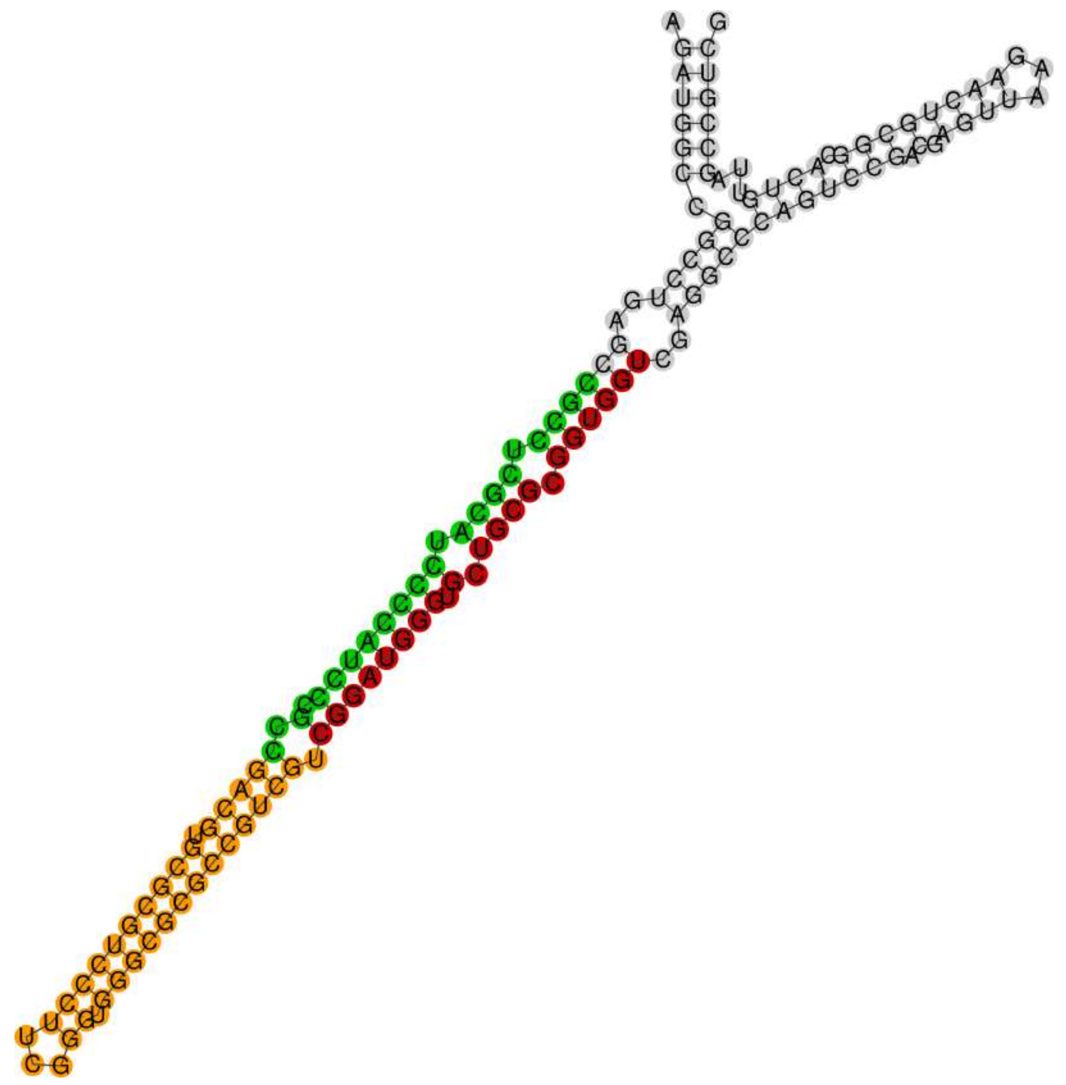

Star / Mature

```
AGAUGGCCGGCCUGAGCCGCCUCGCAUCCCCAUCCCGCCGACGUGCGCGUCCCUUCGGGUGGGCGCGCCGUCGUCGGAUGGGUGCUGCGCGGUGGUCGAGGCCCAGUCCGACGAGUUAAGAACUGCGGCACUGUUAGCCGUCG    16974.4
.((((((.(((((..((((((.((((.((((((((.(.(((((.((((((((((...)).))))))))))))).)))))))).).)))).))))))..)))))(((((((.(.((((...)))))))).))))...)))))).
..........................................................................CGGAUGGGUGCUGCGCGGUGGU...............................................    12878.34773
.................CGCCUCGCAUCCCCAUCCCGCC........................................................................................................    717.822586
.............................................................................AUGGGUGCUGCGCGGUGGU...............................................    6.654518
.............................................................................AUGGGUGCUGCGCGGUGGUA..............................................    3.185643
............................................................................GAUGGGUGCUGCGCGGUGGU...............................................    8.632153
...........................................................................GGAUGGGUGCUGCGCGGUGG................................................    2.43913
...........................................................................GGAUGGGUGCUGCGCGGUGGU...............................................    184.261289
...........................................................................GGAUGGGUGCUGCGCGGUGGUA..............................................    0.357231
..........................................................................CGGAUGGGUGCUGCGCGGUGG................................................    660.005286
..........................................................................CGGAUGGGUGCUGCGCGGUG.................................................    108.337808
..........................................................................CGGAUGGGUGCUGCGCGGU..................................................    113.650154
..........................................................................CGGAUGGGUGCUGCGCGGUGGUC..............................................    4.677659
..........................................................................CGGAUGGGUUCUGCGCGGUGGU...............................................    4.277825
..........................................................................CGGAUGGGUGCUGUGCGGUGGU...............................................    0.364792
..........................................................................CGGAUGGGUGCUGCGAGGUGGU...............................................    2.408794
..........................................................................CGGAUGCGUGCUGCGCGGUGGU...............................................    0.61377
..........................................................................CGGAUGGGUGCUGCGCGGCGGU...............................................    6.841739
..........................................................................CGGAUGGGUGCUGCGCGGGGGU...............................................    7.761097
..........................................................................CUGAUGGGUGCUGCGCGGUGGU...............................................    0.925992
..........................................................................CGGCUGGGUGCUGCGCGGUGGU...............................................    6.0609
..........................................................................CGGAUGGGCGCUGCGCGGUGGU...............................................    7.194178
..........................................................................CGGGUGGGUGCUGCGCGGUGGU...............................................    2.696226
..........................................................................CGGAUGGGUGCUGCGCGGUGGG...............................................    34.229152
..........................................................................CGGAUGGGUGCUGCGCGGUGGC...............................................    242.879807
..........................................................................CGGAUGGGUGCUGCGCGGUGGA...............................................    73.943594
..........................................................................CGGAUGGGUGCUGCGCGGUGGUA..............................................    143.189313
..........................................................................CGGAUGGGUGCUGCGCGGUGGUG..............................................    19.756879
..........................................................................CGGAUGGGAGCUGCGCGGUGGU...............................................    0.182396
..........................................................................CGGAUGGGGGCUGCGCGGUGGU...............................................    0.518993
..........................................................................CGGAGGGGUGCUGCGCGGUGGU...............................................    0.212795
..........................................................................CGGAUGGUUGCUGCGCGGUGGU...............................................    3.961628
..........................................................................CGGAUGGGUGCUGCGCUGUGGU...............................................    2.774629
..........................................................................CGGAUGGGUGCUGCGCGGUGCU...............................................    2.88453
..........................................................................CGGAUGGGUGCUGCGCGGAGGU...............................................    0.273594
..........................................................................CGGACGGGUGCUGCGCGGUGGU...............................................    1.35015
..........................................................................CGGAUGGGUGUUGCGCGGUGGU...............................................    1.54303
..........................................................................AGGAUGGGUGCUGCGCGGUGGU...............................................    1.99826
..........................................................................CGGAUGGGUGCUGCGCGGC..................................................    2.86013
..........................................................................CGGAUGUGUGCUGCGCGGUGGU...............................................    2.235245
..........................................................................CGGAUGGGUACUGCGCGGUGGU...............................................    2.88453
..........................................................................CGGAUGGGUGCUGCGUGGUGGU...............................................    2.663121
..........................................................................CGGAUGGGUGCUGCGCGGUGUU...............................................    5.706205
..........................................................................CGGAUGGGUGCUUCGCGGUGGU...............................................    0.243195
..........................................................................CGGAUGGGUCCUGCGCGGUGGU...............................................    3.448356
..........................................................................CGGAUGAGUGCUGCGCGGUGGU...............................................    2.335495
..........................................................................CGGAUGGGUGCUGCGCGGUGAU...............................................    5.192466
..........................................................................UGGAUGGGUGCUGCGCGGUGGU...............................................    1.15727
..........................................................................CGUAUGGGUGCUGCGCGGUGGU...............................................    1.01025
.........................................................................UCGGAUGGGUGCUGCGCGGU..................................................    3.246275
.........................................................................UCGGAUGGGUGCUGCGCGGUGGU...............................................    212.815899
.........................................................................UCGGAUGGGUGCUGCGCGGUGG................................................    236.790315
.........................................................................UCGGAUGGGUGCUGCGCGGUGGC...............................................    0.884046
.........................................................................UCGGAUGGGUGCUGCGCGGUGGA...............................................    1.382145
.........................................................................UCGGAUGGGUGCUGCGCGGUGGUA..............................................    1.128082
........................................................................GUCGGAUGGGUGCUGCGCGGUGGUA..............................................    0.364792
..................GCCUCGCAUCCCCAUCCCGC.........................................................................................................    0.371561
..................GCCUCGCAUCCCCAUCCC...........................................................................................................    0.318481
..................GCCUCGCAUCCCCAUCCCGCC........................................................................................................    16.580858
.................CGCCUCGCAUCCCCAUCCCGC.........................................................................................................    270.28578
.................CGCCUCGCAUCCCCAUCCCG..........................................................................................................    11.664853
.................CGCCUCGCAUCCCCAUCCC...........................................................................................................    0.477722
.................CGCCUCGCAUCCCCAUCCCGCU........................................................................................................    37.647572
```

zma-MIR_N9b

Chromosome: chr5
Start: 15586389
End: 15586531
Strand: +
Strand bias: 1.0
Abundance bias: 0.958
MFE: -86.90

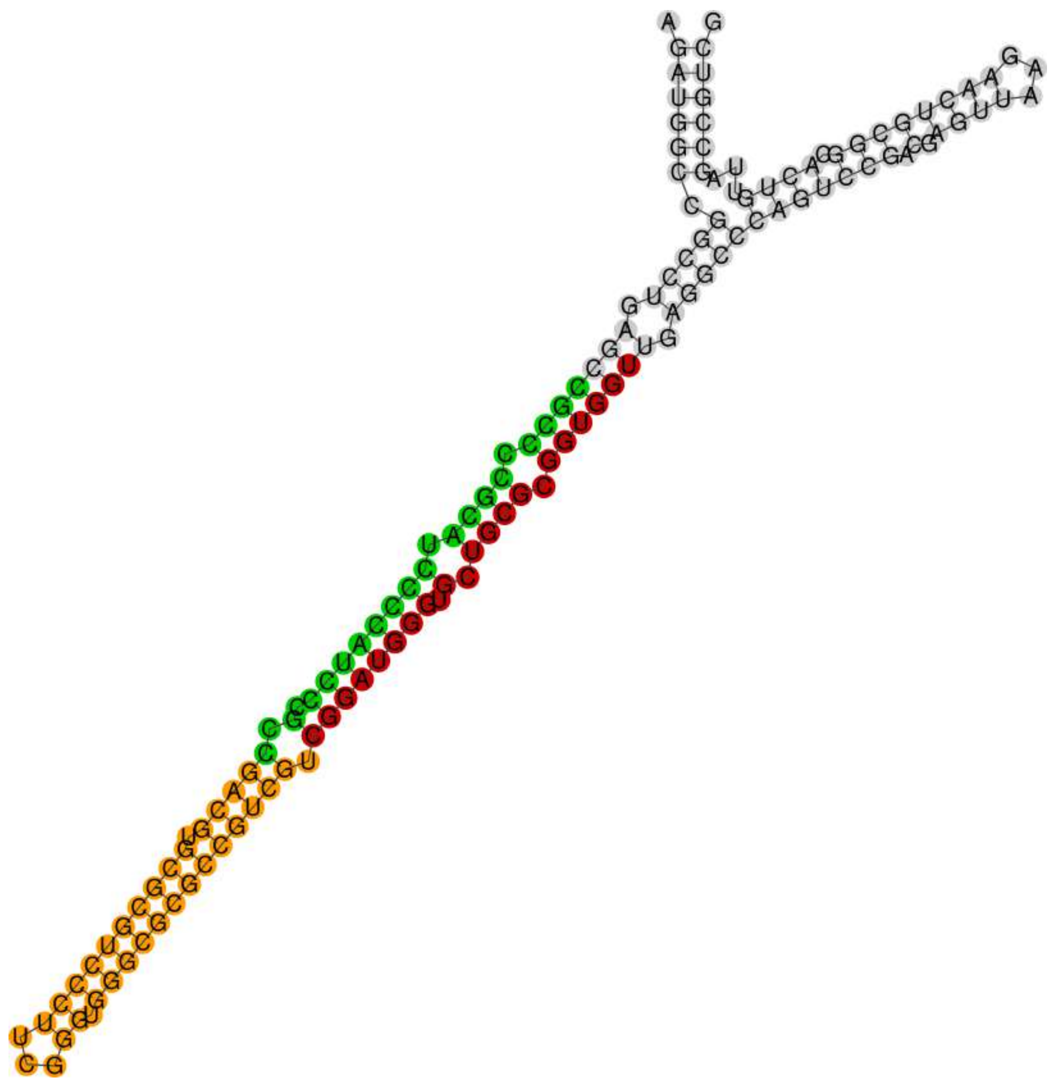

```
                 Star                                                  Mature
AGAUGGCCGGCCUGAGCCGCCCCGCAUCCCCAUCCCGCCGACGUGCGCGUCCCUUCGGGUGGGCGCGCCGUCGUCGGAUGGGUGCUGCGCGGUGGUUGAGGCCCAGUCCGACGAGUUAAGAACUGCGGCACUGUUAGCCGUCG   21501.0
.((((((.(((((.(((((((.((((.((((((((.(.(((((.((((((((((...)).))))))))))))).)))))))).).)))).))))))).))))).(((((((.(.((((...)))))))).))))...)))))).
..........................................................................CGGAUGGGUGCUGCGCGGUGGU...............................................   12878.34773
.................CGCCCCGCAUCCCCAUCCCGCC........................................................................................................   2717.053301
.............................................................................AUGGGUGCUGCGCGGUGGU...............................................   6.654518
.............................................................................AUGGGUGCUGCGCGGUGGUU..............................................   1.562945
.............................................................................AUGGGUGCUGCGCGGUGGUUG.............................................   3.542156
.............................................................................AUGGGUGCUGCGCGGUGGUUGA............................................   0.759983
.............................................................................AUGGGUGCUGCGCGGUGGUA..............................................   3.185643
............................................................................GAUGGGUGCUGCGCGGUGGU...............................................   8.632153
............................................................................GAUGGGUGCUGCGCGGUGGUU..............................................   0.306198
...........................................................................GGAUGGGUGCUGCGCGGUGG................................................   2.43913
...........................................................................GGAUGGGUGCUGCGCGGUGGU...............................................   184.261289
...........................................................................GGAUGGGUGCUGCGCGGUGGUA..............................................   0.357231
..........................................................................CGGAUGGGUGCUGCGCGGUGG................................................   660.005286
..........................................................................CGGAUGGGUGCUGCGCGGUGGUU..............................................   39.78851
..........................................................................CGGAUGGGUGCUGCGCGGUG.................................................   108.337808
..........................................................................CGGAUGGGUGCUGCGCGGU..................................................   113.650154
..........................................................................CGGAUGGGUUCUGCGCGGUGGU...............................................   4.277825
..........................................................................CGGAUGGGUGCUGUGCGGUGGU...............................................   0.364792
..........................................................................CGGAUGGGUGCUGCGAGGUGGU...............................................   2.408794
..........................................................................CGGAUGCGUGCUGCGCGGUGGU...............................................   0.61377
..........................................................................CGGAUGGGUGCUGCGCGGCGGU...............................................   6.841739
..........................................................................CGGAUGGGUGCUGCGCGGGGGU...............................................   7.761097
..........................................................................CUGAUGGGUGCUGCGCGGUGGU...............................................   0.925992
..........................................................................CGGCUGGGUGCUGCGCGGUGGU...............................................   6.0609
..........................................................................CGGAUGGGCGCUGCGCGGUGGU...............................................   7.194178
..........................................................................CGGGUGGGUGCUGCGCGGUGGU...............................................   2.696226
..........................................................................CGGAUGGGUGCUGCGCGGUGGG...............................................   34.229152
..........................................................................CGGAUGGGUGCUGCGCGGUGGC...............................................   242.879807
..........................................................................CGGAUGGGUGCUGCGCGGUGGA...............................................   73.943594
..........................................................................CGGAUGGGUGCUGCGCGGUGGUA..............................................   143.189313
..........................................................................CGGAUGGGUGCUGCGCGGUGGUG..............................................   19.756879
..........................................................................CGGAUGGGAGCUGCGCGGUGGU...............................................   0.182396
..........................................................................CGGAUGGGGGCUGCGCGGUGGU...............................................   0.518993
..........................................................................CGGAGGGGUGCUGCGCGGUGGU...............................................   0.212795
..........................................................................CGGAUGGUUGCUGCGCGGUGGU...............................................   3.961628
..........................................................................CGGAUGGGUGCUGCGCUGUGGU...............................................   2.774629
..........................................................................CGGAUGGGUGCUGCGCGGUGCU...............................................   2.88453
..........................................................................CGGAUGGGUGCUGCGCGGAGGU...............................................   0.273594
..........................................................................CGGACGGGUGCUGCGCGGUGGU...............................................   1.35015
..........................................................................CGGAUGGGUGUUGCGCGGUGGU...............................................   1.54303
..........................................................................AGGAUGGGUGCUGCGCGGUGGU...............................................   1.99826
..........................................................................CGGAUGGGUGCUGCGCGGC..................................................   2.86013
..........................................................................CGGAUGUGUGCUGCGCGGUGGU...............................................   2.235245
..........................................................................CGGAUGGGUACUGCGCGGUGGU...............................................   2.88453
..........................................................................CGGAUGGGUGCUGCGUGGUGGU...............................................   2.663121
..........................................................................CGGAUGGGUGCUGCGCGGUGUU...............................................   5.706205
..........................................................................CGGAUGGGUGCUUCGCGGUGGU...............................................   0.243195
..........................................................................CGGAUGGGUCCUGCGCGGUGGU...............................................   3.448356
..........................................................................CGGAUGAGUGCUGCGCGGUGGU...............................................   2.335495
..........................................................................CGGAUGGGUGCUGCGCGGUGAU...............................................   5.192466
..........................................................................UGGAUGGGUGCUGCGCGGUGGU...............................................   1.15727
..........................................................................CGUAUGGGUGCUGCGCGGUGGU...............................................   1.01025
.........................................................................UCGGAUGGGUGCUGCGCGGU..................................................   3.246275
.........................................................................UCGGAUGGGUGCUGCGCGGUGGU...............................................   212.815899
.........................................................................UCGGAUGGGUGCUGCGCGGUGGUU..............................................   1.932477
.........................................................................UCGGAUGGGUGCUGCGCGGUGG................................................   236.790315
.........................................................................UCGGAUGGGUGCUGCGCGGUGGC...............................................   0.884046
.........................................................................UCGGAUGGGUGCUGCGCGGUGGA...............................................   1.382145
.........................................................................UCGGAUGGGUGCUGCGCGGUGGUA..............................................   1.128082
........................................................................GUCGGAUGGGUGCUGCGCGGUGGUA..............................................   0.364792
..................GCCCCGCAUCCCCAUCCCGC.........................................................................................................   3.658304
..................GCCCCGCAUCCCCAUCCCGCC........................................................................................................   5.679577
.................CGCCCCGCAUCCCCAUCCCGC.........................................................................................................   1863.93701
.................CGCCCCGCAUCCCCAUCCCGCCG.......................................................................................................   0.319396
.................CGCCCCGCAUCCCCAUCCCG..........................................................................................................   180.272163
.................CGCCCCGCAUCCCCAUCCC...........................................................................................................   12.439371
.................CGCCCCGCAUCCCCAUCCCGCCU.......................................................................................................   7.473374
.................CGCCCCGCAUCCCCAUCCCU..........................................................................................................   13.098666
.................CGCCCCGCAUCCCCAUCCCGA.........................................................................................................   1.579001
.................CGCCCCGCAUCCCCAUCCCGU.........................................................................................................   14.289954
.................CGCCCCGCAUCCCCAUCCCGCU........................................................................................................   718.774882
```

zma-MIR156a

Chromosome: chr4
Start: 87069146
End: 87069319
Strand: -
Strand bias: 1.0
Abundance bias: 0.997
MFE: -88.80

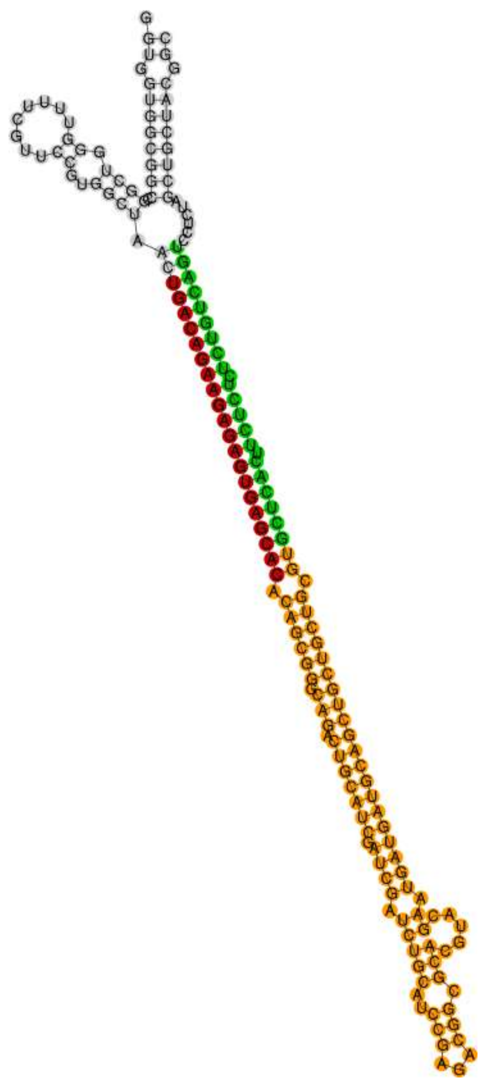

Mature / Star

```
GGUGGUGGCGGCCGGCUGGGUUUUCGUUCCGUGGCUAACUGACAGAAGAGAGUGAGCACACAGCGGGCAGACUGCAUCGAUCGAUCUGCAUCCGAGACGGCGCACGUACGAAUGAUGAUGCAGCUGCUGCUGCGUGCUCACUUCUCUCUCUGUCAGUCCUCUAGCUGCUACGGC    103423.6
.((.((((((((.((((.((........))..)))).((((((((((((((((((((.((((((.(((.(((((((.((((.(((((..(((...))).)))).....)).)))))))))))))))))))).)))))))).))))).)))))))))......)))))))).))
```

GCUCACUUCUCUCUCUGUCAGU	19939.069519
AACUGACAGAAGAGAGUGAGCAC	164.75987
CUGACAGAAGAGAGUGAGC	102.579584
CUGACAGAAGAGAGUGAGCA	383.254841
CUGACAGAAGAGAGUGAGCAC	9425.736403
CUGACAGAAGAGAGCGAGCAC	60.425123
CUGACAGAAGAGAGUGAGCAA	51.77735
CUGACAGAAGAGAGUGAGCAU	45.738507
UGACGGAAGAGAGUGAGCAC	1035.822926
UGACAGAAGAGAGUGAGCUC	281.048229
UGACAGAAGAGAGUGAGCACC	1828.279371
UGACAGAAGAGAGUGAGCACU	6098.791209
UGACAGAAGAGAGUGACCAC	646.770901
UAACAGAAGAGAGUGAGCAC	417.580681
UGACAGAAGAGAGUAAGCAC	488.193738
UGACAGAAGAGAGUGGGC	77.23421
UGACAGAAGAGAUUGAGCAC	1505.804512
UGCCAGAAGAGAGUGAGCAC	501.894875
UGAAAGAAGAGAGUGAGCAC	640.159167
UGACAGAAGAGAGUGAGA	554.015096
UGACAGAAGAGAGUGAGU	2232.969562
UUACAGAAGAGAGUGAGCAC	1035.372591
UGACCGAAGAGAGUGAGCAC	264.671683
UGACAGAAGAGCGUGAGCAC	706.951071
UGACAGAAGAGAGUGAGCGC	1109.259318
UGACAGAAGGGAGUGAGCAC	1001.809665
UGACAGAAGAGAGUGAGAAC	1871.723337
UGACAAAAGAGAGUGAGCAC	256.326531
AGACAGAAGAGAGUGAGCAC	528.84129
UGACAGAAGAGACUGAGCAC	428.37133
UGACAGAAGAGUGUGAGCAC	165.023433
UGUCAGAAGAGAGUGAGCAC	247.60008
UGACAGAUGAGAGUGAGCAC	186.63299
UGACAGGAGAGAGUGAGCAC	894.554726
UGACAGAAGUGAGUGAGCAC	199.47868
UGACAGAAGAGAGUGAACAC	1044.717943
UGACAGAACAGAGUGAGCAC	202.496003
CGACAGAAGAGAGUGAGCAC	1214.211113
UGACAGCAGAGAGUGAGCAC	722.109101
UGACAGAAGCGAGUGAGCAC	495.361686
UCACAGAAGAGAGUGAGCAC	222.85712
GGACAGAAGAGAGUGAGCAC	348.463879
UGAGAGAAGAGAGUGAGCAC	241.705638
UGACUGAAGAGAGUGAGCAC	278.48864
UGACAGAAGAGAAUGAGCAC	260.689562
UGACAGAAGACAGUGAGCAC	173.019221
UGACAGAAUAGAGUGAGCAC	480.6204
UGACAGAAGAGAGUGUGCAC	252.70159
UGACAGUAGAGAGUGAGCAC	174.72889
UGACAGAAGAGAGUGAGCAA	10411.15771
UGACAGAAGAGAGUGAGCAU	9796.070943
UGACAGAAGAGAGUGAUCAC	585.074974
UGACAGAAGAGAGUGAGCAG	765.609921
UGGCAGAAGAGAGUGAGCAC	947.950764
UGACACAAGAGAGUGAGCAC	157.041902
UGACAGAAGAGAGUGCGCAC	1154.036353
UGACAGAAGAGAGUGGGCAC	1099.312511
UGACAGAAGAGAGUGAGUAC	588.143268
UGACAGAAGAGAGUUAGCAC	541.985546
UGACAGAAGAGAGUCAGCAC	235.215704
UGACAGAAGAAAGUGAGCAC	246.464492
UGACAGAAGAUAGUGAGCAC	611.235981
UGACAGACGAGAGUGAGCAC	352.374538
UGACAGAAAAGAGUGAGCAC	408.181761
UGACAGAAGAGAGUGACC	39.26405
UGACAUAAGAGAGUGAGCAC	619.31861
UGACAGAGGAGAGUGAGCAC	903.11262
UGACAGAAGAGAGUGAGCC	63.345774
UGACAGAAGAGAGUGAGCG	212.845116
UGACAGAAGAGAGUGAGCU	189.011259
UGACAGAAGAGGGUGAGCAC	940.452296
UGACAGAAGAGAGUGAGCCC	3924.353668
UGACAGAAGAGAGUGAGGAC	190.055323
GACAGAAGAGAGUGAGCACU	110.958352
ACAGAAGAGAGUGAGCACU	100.871647
UGCUCACUUCUCUCUCUGUCAGU	116.19639
UGCUCACUUCUCUUUCUGUCAGU	467.182479
GCUCACUUCUCUCUCUGUCAGUCC	86.259605
GCUCACUUCUCUCUCUGUC	152.336886
GCUCACUUCUCUCUCUGUCAG	365.46719
GCUCACUUCUCUCUCUGUCA	292.0869
GCCCACUUCUCUCUCUGUCAGU	37.9093
GCUCACUUCUCUCUCUGUCAGG	39.928552
GCUCACUUCUCUCUCUGUCAGA	98.191904
GCUCACUUCUCUUUCUGUCAGU	3699.902946
GCUCACUUCUCCCUCUGUCAGU	34.184388
GCUCACUUCUUUCUCUGUCAGU	42.622179
GCUCACUUCUCUCUCCGUCAGU	33.792879
GCUCACUUCCCUCUCUGUCAGU	38.781508
GCUCACUUCUCUCUCUGCCAGU	34.838924
GCUCACUUCUCUCUCUGUCAGUU	32.643158
GCUCACUCCUCUCUCUGUCAGU	44.875908
GCUCACUUCUCUCUCUGUCGGU	53.633905
ACUCACUUCUCUCUCUGUCAGU	32.538694
GCUCACCUCUCUCUCUGUCAGU	49.728872
GCUCGCUUCUCUCUCUGUCAGU	33.072911
GCUCACUUCUCUCCCUGUCAGU	34.486387

zma-MIR156e

Chromosome: chr2
Start: 33005027
End: 33005183
Strand: +
Strand bias: 1.0
Abundance bias: 1.0
MFE: -99.10

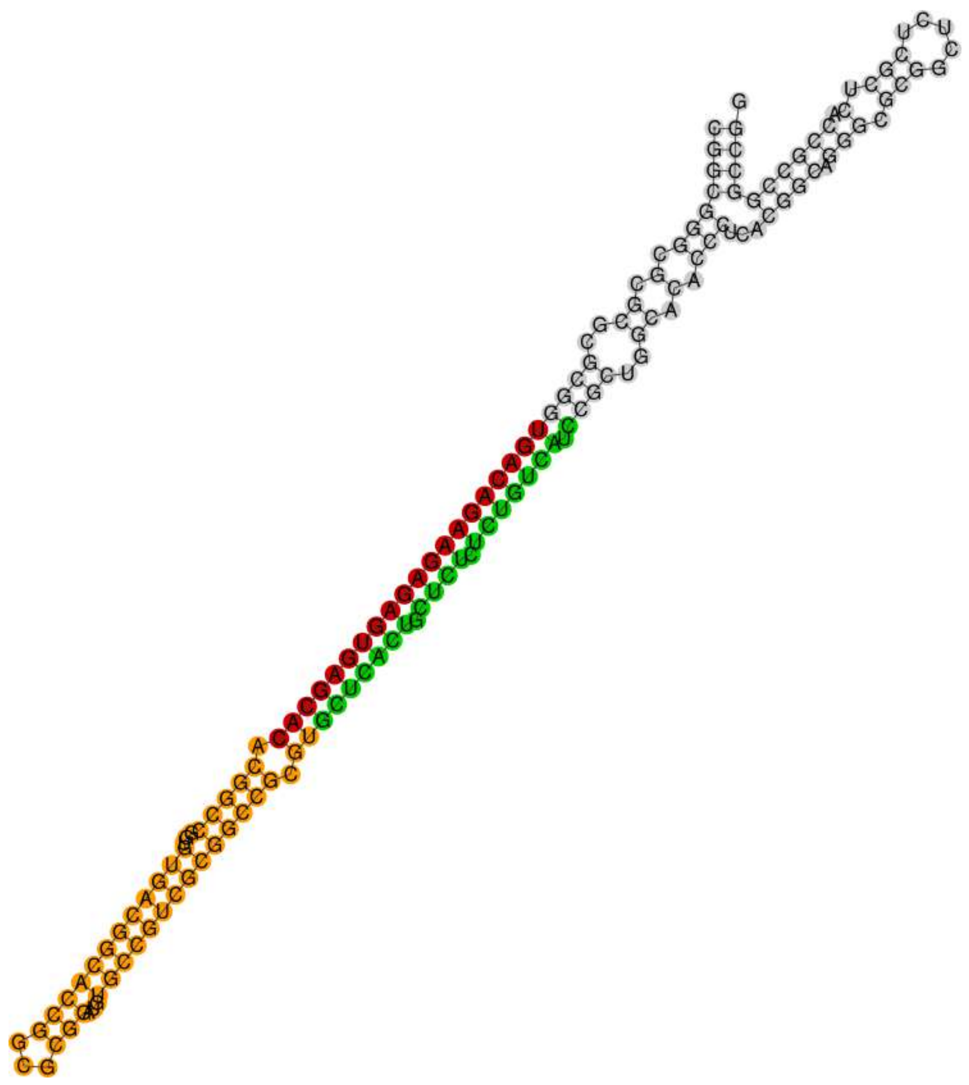

```
                  Mature                                                         Star
CGGCGGGCGCGCGCGCGGUGACAGAAGAGAGUGAGCACACGGCCGGUGUGACGGCACCGGCGCGGAUGUGCCGUCGCGGCCGCGUGCUCACUGCUCUCUCUGUCAUCCGCUGGCACACCCUCACGGCAGGGCGCGGCUCUCGCUCACCGCCGGCCGG    77717.9
((((((.(.((..(((((((((((((((((((((((.(((((...(((((((((((...)))...)))))))))))))).))))))))).)))).))))))).))))..)).).)))...((((.(((.(((.....))).).)))))))))).

.....................................................................................GCUCACUGCUCUCUCUGUCAUC..................................................    6808.310455
...............CGGUGACAGAAGAGAGUGAGCAC.......................................................................................................................    19.411009
................GGUGACAGAAGAGAGUGAGCAC.......................................................................................................................    26.933198
.................GUGACAGAAGAGAGUGAGCAC.......................................................................................................................    116.752429
..................UGACAGAAGGGAGUGAGC.........................................................................................................................    8.85506
..................UGACGGAAGAGAGUGAGCAC.......................................................................................................................    1035.822926
..................UGACAGAAGAGAGUGAGCUC.......................................................................................................................    281.048229
..................UGACAGAAGAGAGUGAGCACC......................................................................................................................    1828.279371
..................UGACAGAAGAGAGUGAGCACU......................................................................................................................    6098.791209
..................UGACAGAAGAGAGUGACCAC.......................................................................................................................    646.770901
..................UAACAGAAGAGAGUGAGCAC.......................................................................................................................    417.580681
..................UGACAGAAGAGAGUAAGCAC.......................................................................................................................    488.193738
..................UGACAGAAGAGAGUGGGC.........................................................................................................................    77.23421
..................UGACAGAAGAGAUUGAGCAC.......................................................................................................................    1505.804512
..................UGCCAGAAGAGAGUGAGCAC.......................................................................................................................    501.894875
..................UGAAAGAAGAGAGUGAGCAC.......................................................................................................................    640.159167
..................UGACAGAAGAGAGUGAGG.........................................................................................................................    26.55614
..................UGACAGAAGAGAGUGAGA.........................................................................................................................    554.015096
..................UGACAGAAGAGAGUGAGU.........................................................................................................................    2232.969562
..................UUACAGAAGAGAGUGAGCAC.......................................................................................................................    1035.372591
..................UGACCGAAGAGAGUGAGCAC.......................................................................................................................    264.671683
..................UGACAGAAGGGAGUGAGCA........................................................................................................................    13.717518
..................UGACAGAAGAGCGUGAGCAC.......................................................................................................................    706.951071
..................UGACAGAAGAGAGUGAGCGC.......................................................................................................................    1109.259318
..................UGACAGAAGAGAGUGAAC.........................................................................................................................    8.95173
..................UGACAGAAGGGAGUGAGCAC.......................................................................................................................    1001.809665
..................UGACAGAAGAGAGUGAGAA........................................................................................................................    6.907639
..................UGACAGAAGAGAGUGAGAAC.......................................................................................................................    1871.723337
..................UGACAAAAGAGAGUGAGCAC.......................................................................................................................    256.326531
..................AGACAGAAGAGAGUGAGCAC.......................................................................................................................    528.84129
..................UGACAGAAGAGACUGAGCAC.......................................................................................................................    428.37133
..................UGACAGAAGAGUGUGAGCAC.......................................................................................................................    165.023433
..................UGUCAGAAGAGAGUGAGCAC.......................................................................................................................    247.60008
..................UGACAGAAGAGAGUGGGCA........................................................................................................................    26.859814
..................UGACAGAUGAGAGUGAGCAC.......................................................................................................................    186.63299
..................UGACAGGAGAGAGUGAGCAC.......................................................................................................................    894.554726
..................UGACAGAAGUGAGUGAGCAC.......................................................................................................................    199.47868
..................UGACAGAAGAGAGUGAACAC.......................................................................................................................    1044.717943
..................UGACAGAACAGAGUGAGCAC.......................................................................................................................    202.496003
..................CGACAGAAGAGAGUGAGCAC.......................................................................................................................    1214.211113
..................UGACAGCAGAGAGUGAGCAC.......................................................................................................................    722.109101
..................UGACAGAAGCGAGUGAGCAC.......................................................................................................................    495.361686
..................UCACAGAAGAGAGUGAGCAC.......................................................................................................................    222.85712
..................GGACAGAAGAGAGUGAGCAC.......................................................................................................................    348.463879
..................UGAGAGAAGAGAGUGAGCAC.......................................................................................................................    241.705638
..................UGACUGAAGAGAGUGAGCAC.......................................................................................................................    278.48864
..................UGACAGAAGAGAGUGAGCCCA......................................................................................................................    4.8288
..................UGACAGAAGAGAAUGAGCAC.......................................................................................................................    260.689562
..................UGACAGAAGACAGUGAGCAC.......................................................................................................................    173.019221
..................UGACAGAAUAGAGUGAGCAC.......................................................................................................................    480.6204
..................UGACAGAAGAGAGUGUGCAC.......................................................................................................................    252.70159
..................UGACAGUAGAGAGUGAGCAC.......................................................................................................................    174.72889
..................UGACAGAAGAGGGUGAGCA........................................................................................................................    14.015897
..................UGACAGAAGAGAGUGAGCAA.......................................................................................................................    10411.15771
..................UGACAGAAGAGAGUGAGCAU.......................................................................................................................    9796.070943
..................UGACAGAAGAGAGUGAUCAC.......................................................................................................................    585.074974
..................UGACAGAAGAGAGUGAGCAG.......................................................................................................................    765.609921
..................UGACAGAAGAGAUUGAGCA........................................................................................................................    7.261237
..................UGACAGAAGAGGGUGAGC.........................................................................................................................    12.5658
..................UGGCAGAAGAGAGUGAGCAC.......................................................................................................................    947.950764
..................UGACAGAAGAGAGUGAGCAAA......................................................................................................................    22.295708
..................UGACACAAGAGAGUGAGCAC.......................................................................................................................    157.041902
..................UGACAGAAGAGAGUGCGCAC.......................................................................................................................    1154.036353
..................UGACAGAAGAGAGUGGGCAC.......................................................................................................................    1099.312511
..................UGACAGAAGAGAGUGAGUAC.......................................................................................................................    588.143268
..................UGACAGAAGAGAGUUAGCAC.......................................................................................................................    541.985546
..................UGACAGAAGAGAGUCAGCAC.......................................................................................................................    235.215704
..................UGACAGAAGAAAGUGAGCAC.......................................................................................................................    246.464492
..................UGACAGAAGAGAGUGUGCA........................................................................................................................    10.05945
..................UGACAGAAGAUAGUGAGCAC.......................................................................................................................    611.235981
..................UGACAGACGAGAGUGAGCAC.......................................................................................................................    352.374538
..................UGACAGAAAAGAGUGAGCAC.......................................................................................................................    408.181761
..................UGACAGAAGAGAGUGACC.........................................................................................................................    39.26405
..................UGACAUAAGAGAGUGAGCAC.......................................................................................................................    619.31861
..................UGACAGAGGAGAGUGAGCAC.......................................................................................................................    903.11262
..................UGACAGAAGAGAGUGAGCC........................................................................................................................    63.345774
..................UGACAGAAGAGAGUGAGCG........................................................................................................................    212.845116
..................UGACAGAAGAGAGUGAGCU........................................................................................................................    189.011259
..................UGACAGAAGAGGGUGAGCAC.......................................................................................................................    940.452296
..................UGACAGAAGAGAGUGAGCCC.......................................................................................................................    3924.353668
..................UGACAGAAGAGAGUUAGCA........................................................................................................................    4.904918
..................UGACAGAAGAGAGUGAGGAC.......................................................................................................................    190.055323
...................GACAGAAGAGAGUGAGCAA.......................................................................................................................    5.75943
...................GACAGAAGAGAGUGAGCACU......................................................................................................................    110.958352
....................ACAGAAGAGAGUGAGCACU......................................................................................................................    100.871647
.....................CAGAAGAGAGUGAGCACACG....................................................................................................................    5.132734
....................................................................................UGCUCACUGCUCUCUCUGUCAUC..................................................    3144.182058
....................................................................................UGCUCACUGCUCUCUCUGUCAU...................................................    138.855787
....................................................................................UGCUCACUGCUCUCUCUGUC.....................................................    25.47496
....................................................................................UGCUCACUGCUCUCUCUGUCAUU.................................................    71.097695
.....................................................................................GCUCACUGCUCUCUCUGUCA....................................................    23.277504
.....................................................................................GCUCACUGCUCUCUCUGUCAU...................................................    371.044321
.....................................................................................GCUCACUGCUCUCUCUGUC.....................................................    21.30662
.....................................................................................GCUCACUGCUCUCUCUGUCAUA.................................................    21.596048
.....................................................................................GCUCACUGCUCUCUCUGUCAUU.................................................    138.82144
.....................................................................................GCUCACUGCUCUCUCUGUCACC.................................................    12.542391
.....................................................................................GCUCACCGCUCUCUCUGUCAUC..................................................    5.739701
```

zma-MIR156f

Chromosome: chr2
Start: 185613976
End: 185614167
Strand: -
Strand bias: 1.0
Abundance bias: 0.998
MFE: -91.20

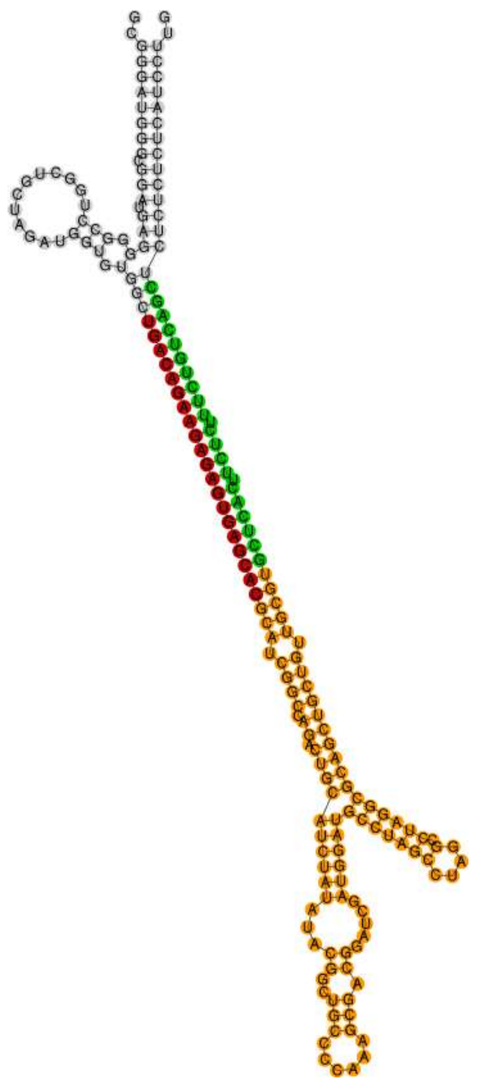

Mature / Star

GCGGGAUGGGCGGAUGAGGGGCCUGGCUGCUAGAUGGUGUGGCUGACAGAAGAGAGUGAGCACGCAUCGGCCAGACUGCAUCUAUAUACGGCUGCCCCAAAGCGACGGAUCGAUGGAUGCCUAGCCUAGGCCUAGGCGCAGCUGCUGUUGCGUGCUCACUUCUCUUUCUGUCAGCUCUCUCUCUCAUCCUUG 528075.2

..((((((((.(((.((((.(((............))).)((((((((((((((((((((((((.((((.((.((((((((((...((..(((......))).)).....))))))((((((....).)))))))))))))))).))))))))))).)))).))))))))))))))))))))))))..

Read	Abundance
GCUCACUUCUCUUUCUGUCAGC	364945.120691
CUGACAGAAGAGAGUGAGCA	383.254841
CUGACAGAAGAGAGUGAGCAC	9425.736403
UGACAGAAGAGAGUGAGCACG	2387.522239
UGACGGAAGAGAGUGAGCAC	1035.822926
UGACAGAAGAGAGUGAGCUC	281.048229
UGACAGAAGAGAGUGAGCACC	1828.279371
UGACAGAAGAGAGUGAGCACU	6098.791209
UGACAGAAGAGAGUGACCAC	646.770901
UAACAGAAGAGAGUGAGCAC	417.580681
UGACAGAAGAGAGUAAGCAC	488.193738
UGACAGAAGAGAUUGAGCAC	1505.804512
UGCCAGAAGAGAGUGAGCAC	501.894875
UGAAAGAAGAGAGUGAGCAC	640.159167
UGACAGAAGAGAGUGAGA	554.015096
UGACAGAAGAGAGUGAGU	2232.969562
UUACAGAAGAGAGUGAGCAC	1035.372591
UGACCGAAGAGAGUGAGCAC	264.671683
UGACAGAAGAGCGUGAGCAC	706.951071
UGACAGAAGAGAGUGAGCGC	1109.259318
UGACAGAAGGGAGUGAGCAC	1001.809665
UGACAGAAGAGAGUGAGAAC	1871.723337
UGACAAAAGAGAGUGAGCAC	256.326531
AGACAGAAGAGAGUGAGCAC	528.84129
UGACAGAAGAGACUGAGCAC	428.37133
UGUCAGAAGAGAGUGAGCAC	247.60008
UGACAGGAGAGAGUGAGCAC	894.554726
UGACAGAAGUGAGUGAGCAC	199.47868
UGACAGAAGAGAGUGAACAC	1044.717943
UGACAGAACAGAGUGAGCAC	202.496003
CGACAGAAGAGAGUGAGCAC	1214.211113
UGACAGCAGAGAGUGAGCAC	722.109101
UGACAGAAGCGAGUGAGCAC	495.361686
UCACAGAAGAGAGUGAGCAC	222.85712
GGACAGAAGAGAGUGAGCAC	348.463879
UGAGAGAAGAGAGUGAGCAC	241.705638
UGACUGAAGAGAGUGAGCAC	278.48864
UGACAGAAGAGAAUGAGCAC	260.689562
UGACAGAAUAGAGUGAGCAC	480.6204
UGACAGAAGAGAGUGUGCAC	252.70159
UGACAGAAGAGAGUGAGCAA	10411.15771
UGACAGAAGAGAGUGAGCAU	9796.070943
UGACAGAAGAGAGUGAUCAC	585.074974
UGACAGAAGAGAGUGAGCAG	765.609921
UGGCAGAAGAGAGUGAGCAC	947.950764
UGACAGAAGAGAGUGCGCAC	1154.036353
UGACAGAAGAGAGUGGGCAC	1099.312511
UGACAGAAGAGAGUGAGUAC	588.143268
UGACAGAAGAGAGUUAGCAC	541.985546
UGACAGAAGAGAGUCAGCAC	235.215704
UGACAGAAGAAAGUGAGCAC	246.464492
UGACAGAAGAUAGUGAGCAC	611.235981
UGACAGACGAGAGUGAGCAC	352.374538
UGACAGAAAAGAGUGAGCAC	408.181761
UGACAUAAGAGAGUGAGCAC	619.31861
UGACAGAGGAGAGUGAGCAC	903.11262
UGACAGAAGAGAGUGAGCG	212.845116
UGACAGAAGAGGGUGAGCAC	940.452296
UGACAGAAGAGAGUGAGCCC	3924.353668
UGCUCACUUCUCUUUCUGUCAGC	46602.614945
UGCUCACUUCUCUUUCUGUCAG	947.328945
UGCUCACUUCUCUUUCUGUCAGU	467.182479
UGCUCACUUCUCUUUCUGUCAGA	329.83814
GCUCACUUCUCUUUCUGUCA	1394.92418
GCUCACUUCUCUUUCUGUCAG	5692.0141
GCUCACUUCUCUUUCUGUCAGCU	2092.03289
GCUCACUUCUCCUUCUGUCAGC	807.484845
GUUCACUUCUCUUUCUGUCAGC	603.259066
GCUCCCUUCUCUUUCUGUCAGC	262.617552
GCUUACUUCUCUUUCUGUCAGC	451.949223
GCUCACCUCUCUUUCUGUCAGC	809.68754
GCUCACUCCUCUUUCUGUCAGC	889.91802
GCUCACUUCUCUUUCCGUCAGC	689.69269
GCUCACUUCUCUUCCUGUCAGC	611.91629
GCUCACUUCCCUUUCUGUCAGC	709.862347
GCUCACUUCUCUUUCUGUCAGU	3699.902946
GCUCACUUCUCUUUCUGUCAGA	1703.20216
GCCCACUUCUCUUUCUGUCAGC	738.665243
GCUCACUUCUCUUUUUGUCAGC	228.99606
GCUCAUUUCUCUUUCUGUCAGC	473.76077
GCUCACUUCUUUUUCUGUCAGC	401.250028
GCUCACUUCUCUUUCUGUUAGC	297.73002
GCACACUUCUCUUUCUGUCAGC	211.64703
GAUCACUUCUCUUUCUGUCAGC	258.35862
GCUCACUUCUCUUUCUGUCAAC	267.94471
GCUCACUUUUCUUUCUGUCAGC	321.30935
GCUCACUUCUCUUUCUGUCAUC	317.18406
GCUCACUUCUCUUUCUGCCAGC	709.20568
ACUCACUUCUCUUUCUGUCAGC	619.701424
GCUCACUUCUCUUUCUGUCAGCA	400.173986
GCUCACUUCUCUUUCUGUCGGC	815.711125
GCUCACUUCUCUUUCUGUCACC	311.0367
GCUCACUUCUCUUUCUUUCAGC	282.18636
UCUCACUUCUCUUUCUGUCAGC	396.302
CUCACUUCUCUUUCUGUCAGC	1172.328604
UCACUUCUCUUUCUGUCAGC	251.08668
CACUUCUCUUUCUGUCAGC	478.815288

zma-MIR156g

Chromosome: chr7
Start: 119411269
End: 119411441
Strand: +
Strand bias: 1.0
Abundance bias: 0.998
MFE: -81.40

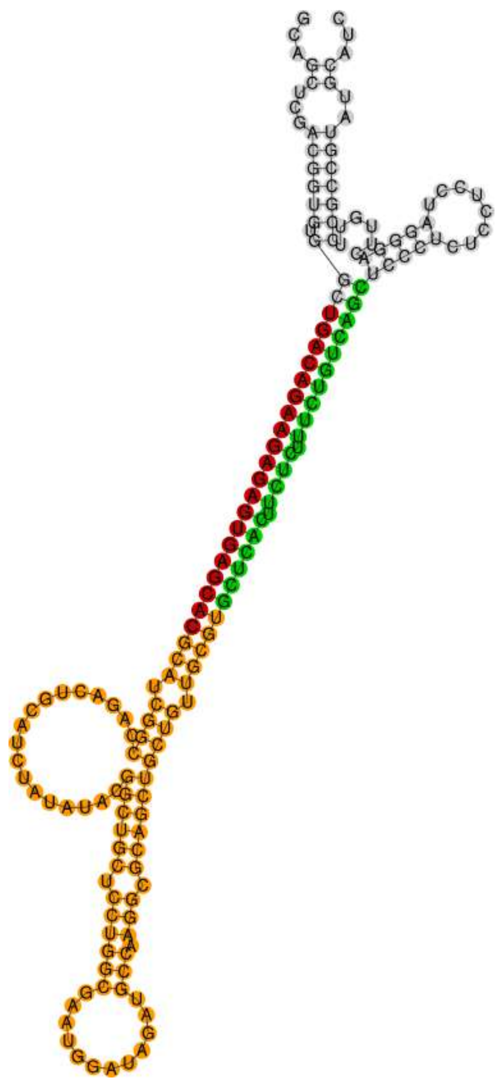

```
                  Mature                                                                                          Star
GCAGCUCGACGGUGUGGCUGACAGAAGAGAGUGAGCACGCAUCGGCCAGACUGCAUCUAUAUACGGCUGCUCCUGGCGAAUGGAUAGAUGCCAAGGCGCAGCUGCUGUUGCGUGCUCACUUCUCUUUCUGUCAGCUCCCUCUCCUCCUAGGGACUUGUUCCGCCGUAUGCAUC    528073.5
...((...(((((.((((((((((((((((((((((((.((((.................((((((.((((((...........))).))).))))))))))).))))))))))).)))).))))))))))(((((........)))))......)))))))..))...

.................................................................................................................GCUCACUUCUCUUUCUGUCAGC......................................    364945.120691
.................CUGACAGAAGAGAGUGAGCA........................................................................................................................................    383.254841
.................CUGACAGAAGAGAGUGAGCAC.......................................................................................................................................    9425.736403
..................UGACAGAAGAGAGUGAGCACG......................................................................................................................................    2387.522239
..................UGACGGAAGAGAGUGAGCAC.......................................................................................................................................    1035.822926
..................UGACAGAAGAGAGUGAGCUC.......................................................................................................................................    281.048229
..................UGACAGAAGAGAGUGAGCACC......................................................................................................................................    1828.279371
..................UGACAGAAGAGAGUGAGCACU......................................................................................................................................    6098.791209
..................UGACAGAAGAGAGUGACCAC.......................................................................................................................................    646.770901
..................UAACAGAAGAGAGUGAGCAC.......................................................................................................................................    417.580681
..................UGACAGAAGAGAGUAAGCAC.......................................................................................................................................    488.193738
..................UGACAGAAGAGAUUGAGCAC.......................................................................................................................................    1505.804512
..................UGCCAGAAGAGAGUGAGCAC.......................................................................................................................................    501.894875
..................UGAAAGAAGAGAGUGAGCAC.......................................................................................................................................    640.159167
..................UGACAGAAGAGAGUGAGA.........................................................................................................................................    554.015096
..................UGACAGAAGAGAGUGAGU.........................................................................................................................................    2232.969562
..................UUACAGAAGAGAGUGAGCAC.......................................................................................................................................    1035.372591
..................UGACCGAAGAGAGUGAGCAC.......................................................................................................................................    264.671683
..................UGACAGAAGAGCGUGAGCAC.......................................................................................................................................    706.951071
..................UGACAGAAGAGAGUGAGCGC.......................................................................................................................................    1109.259318
..................UGACAGAAGGGAGUGAGCAC.......................................................................................................................................    1001.809665
..................UGACAGAAGAGAGUGAGAAC.......................................................................................................................................    1871.723337
..................UGACAAAAGAGAGUGAGCAC.......................................................................................................................................    256.326531
..................AGACAGAAGAGAGUGAGCAC.......................................................................................................................................    528.84129
..................UGACAGAAGAGACUGAGCAC.......................................................................................................................................    428.37133
..................UGUCAGAAGAGAGUGAGCAC.......................................................................................................................................    247.60008
..................UGACAGGAGAGAGUGAGCAC.......................................................................................................................................    894.554726
..................UGACAGAAGUGAGUGAGCAC.......................................................................................................................................    199.47868
..................UGACAGAAGAGAGUGAACAC.......................................................................................................................................    1044.717943
..................UGACAGAACAGAGUGAGCAC.......................................................................................................................................    202.496003
..................CGACAGAAGAGAGUGAGCAC.......................................................................................................................................    1214.211113
..................UGACAGCAGAGAGUGAGCAC.......................................................................................................................................    722.109101
..................UGACAGAAGCGAGUGAGCAC.......................................................................................................................................    495.361686
..................UCACAGAAGAGAGUGAGCAC.......................................................................................................................................    222.85712
..................GGACAGAAGAGAGUGAGCAC.......................................................................................................................................    348.463879
..................UGAGAGAAGAGAGUGAGCAC.......................................................................................................................................    241.705638
..................UGACUGAAGAGAGUGAGCAC.......................................................................................................................................    278.48864
..................UGACAGAAGAGAAUGAGCAC.......................................................................................................................................    260.689562
..................UGACAGAAUAGAGUGAGCAC.......................................................................................................................................    480.6204
..................UGACAGAAGAGAGUGUGCAC.......................................................................................................................................    252.70159
..................UGACAGAAGAGAGUGAGCAA.......................................................................................................................................    10411.15771
..................UGACAGAAGAGAGUGAGCAU.......................................................................................................................................    9796.070943
..................UGACAGAAGAGAGUGAUCAC.......................................................................................................................................    585.074974
..................UGACAGAAGAGAGUGAGCAG.......................................................................................................................................    765.609921
..................UGGCAGAAGAGAGUGAGCAC.......................................................................................................................................    947.950764
..................UGACAGAAGAGAGUGCGCAC.......................................................................................................................................    1154.036353
..................UGACAGAAGAGAGUGGGCAC.......................................................................................................................................    1099.312511
..................UGACAGAAGAGAGUGAGUAC.......................................................................................................................................    588.143268
..................UGACAGAAGAGAGUUAGCAC.......................................................................................................................................    541.985546
..................UGACAGAAGAGAGUCAGCAC.......................................................................................................................................    235.215704
..................UGACAGAAGAAAGUGAGCAC.......................................................................................................................................    246.464492
..................UGACAGAAGAUAGUGAGCAC.......................................................................................................................................    611.235981
..................UGACAGACGAGAGUGAGCAC.......................................................................................................................................    352.374538
..................UGACAGAAAAGAGUGAGCAC.......................................................................................................................................    408.181761
..................UGACAUAAGAGAGUGAGCAC.......................................................................................................................................    619.31861
..................UGACAGAGGAGAGUGAGCAC.......................................................................................................................................    903.11262
..................UGACAGAAGAGAGUGAGCG........................................................................................................................................    212.845116
..................UGACAGAAGAGGGUGAGCAC.......................................................................................................................................    940.452296
..................UGACAGAAGAGAGUGAGCCC.......................................................................................................................................    3924.353668
................................................................................................................UGCUCACUUCUCUUUCUGUCAGC......................................    46602.614945
................................................................................................................UGCUCACUUCUCUUUCUGUCAG.......................................    947.328945
................................................................................................................UGCUCACUUCUCUUUCUGUCAGU.....................................    467.182479
................................................................................................................UGCUCACUUCUCUUUCUGUCAGA.....................................    329.83814
.................................................................................................................GCUCACUUCUCUUUCUGUCA........................................    1394.92418
.................................................................................................................GCUCACUUCUCUUUCUGUCAG.......................................    5692.0141
.................................................................................................................GCUCACUUCUCUUUCUGUCAGCU.....................................    2092.03289
.................................................................................................................GCUCACUUCUCCUUCUGUCAGC......................................    807.484845
.................................................................................................................GUUCACUUCUCUUUCUGUCAGC......................................    603.259066
.................................................................................................................GCUCCCUUCUCUUUCUGUCAGC......................................    262.617552
.................................................................................................................GCUUACUUCUCUUUCUGUCAGC......................................    451.949223
.................................................................................................................GCUCACCUCUCUUUCUGUCAGC......................................    809.68754
.................................................................................................................GCUCACUCCUCUUUCUGUCAGC......................................    889.91802
.................................................................................................................GCUCACUUCUCUUUCCGUCAGC......................................    689.69269
.................................................................................................................GCUCACUUCUCUUCCUGUCAGC......................................    611.91629
.................................................................................................................GCUCACUUCCCUUUCUGUCAGC......................................    709.862347
.................................................................................................................GCUCACUUCUCUUUCUGUCAGU......................................    3699.902946
.................................................................................................................GCUCACUUCUCUUUCUGUCAGA......................................    1703.20216
.................................................................................................................GCCCACUUCUCUUUCUGUCAGC......................................    738.665243
.................................................................................................................GCUCACUUCUCUUUUUGUCAGC......................................    228.99606
.................................................................................................................GCUCAUUUCUCUUUCUGUCAGC......................................    473.76077
.................................................................................................................GCUCACUUCUUUUUCUGUCAGC......................................    401.250028
.................................................................................................................GCUCACUUCUCUUUCUGUUAGC......................................    297.73002
.................................................................................................................GCACACUUCUCUUUCUGUCAGC......................................    211.64703
.................................................................................................................GAUCACUUCUCUUUCUGUCAGC......................................    258.35862
.................................................................................................................GCUCACUUCUCUUUCUGUCAAC......................................    267.94471
.................................................................................................................GCUCACUUUUCUUUCUGUCAGC......................................    321.30935
.................................................................................................................GCUCACUUCUCUUUCUGUCAUC......................................    317.18406
.................................................................................................................GCUCACUUCUCUUUCUGCCAGC......................................    709.20568
.................................................................................................................ACUCACUUCUCUUUCUGUCAGC......................................    619.701424
.................................................................................................................GCUCACUUCUCUUUCUGUCAGCA.....................................    400.173986
.................................................................................................................GCUCACUUCUCUUUCUGUCGGC......................................    815.711125
.................................................................................................................GCUCACUUCUCUUUCUGUCACC......................................    311.0367
.................................................................................................................GCUCACUUCUCUUUCUUUCAGC......................................    282.18636
.................................................................................................................UCUCACUUCUCUUUCUGUCAGC......................................    396.302
..................................................................................................................CUCACUUCUCUUUCUGUCAGC......................................    1172.328604
...................................................................................................................UCACUUCUCUUUCUGUCAGC......................................    251.08668
....................................................................................................................CACUUCUCUUUCUGUCAGC......................................    478.815288
```

zma-MIR156h

Chromosome: chr10
Start: 129642869
End: 129643013
Strand: -
Strand bias: 1.0
Abundance bias: 0.999
MFE: -99.40

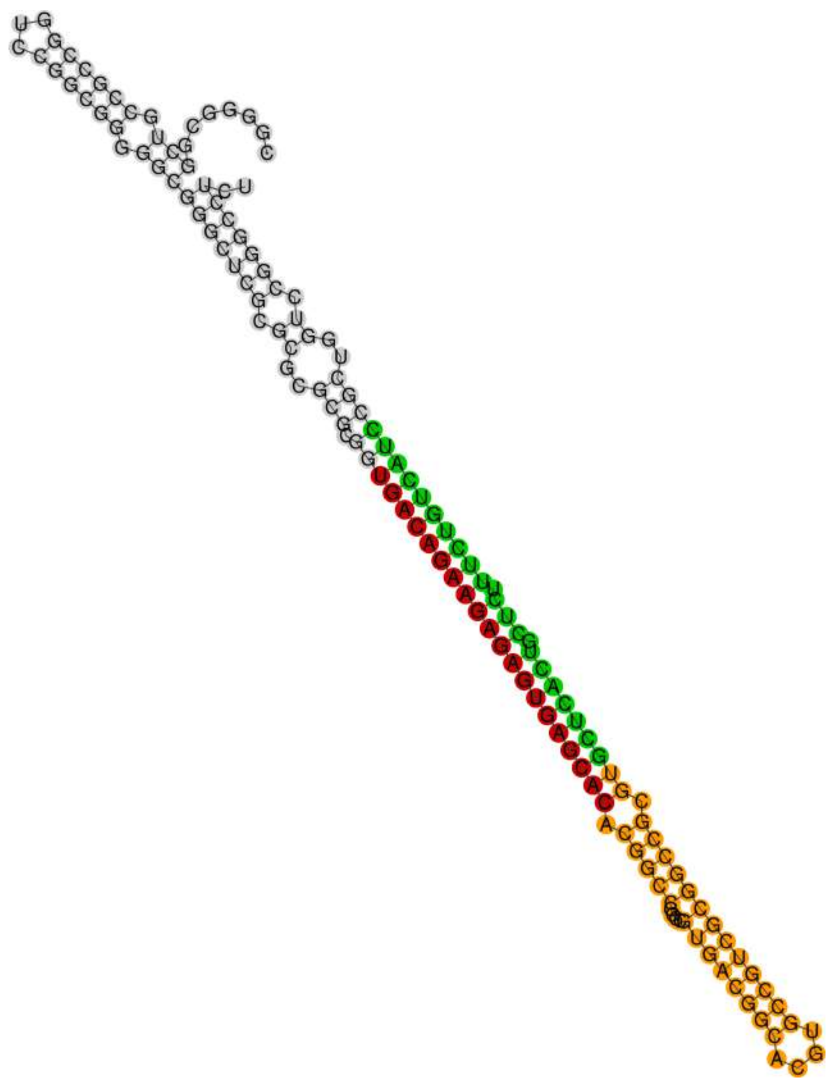

```
                                                Mature                                                Star
CGGGGCGGCUGCCGCCGGUCCGGCGGGGGCGGGCUCGCGCGCGCGCGGUGACAGAAGAGAGUGAGCACACGGCCGGGCGUGACGGCACGUGCCGUCGCGGCCGCGUGCUCACUGCUCUUUCUGUCAUCCGCUGGUCCGGGCCUCU    68319.4
.......(((.((((((...)))))).)))(((((((.((..(((.((((((((((((((((((((((.(((((....((((((((....))))))))))))).))))))))).))).)))))))))))))..)).)))))))..

..........................................................................................................GCUCACUGCUCUUUCUGUCAUC.................    888.625808
.............................................CGGUGACAGAAGAGAGUGAGCAC.............................................................................    19.411009
..............................................GGUGACAGAAGAGAGUGAGCAC.............................................................................    26.933198
...............................................GUGACAGAAGAGAGUGAGCAC.............................................................................    116.752429
................................................UGACAGAAGGGAGUGAGC...............................................................................    8.85506
................................................UGACGGAAGAGAGUGAGCAC.............................................................................    1035.822926
................................................UGACAGAAGAGAGUGAGCUC.............................................................................    281.048229
................................................UGACAGAAGAGAGUGAGCACC............................................................................    1828.279371
................................................UGACAGAAGAGAGUGAGCACU............................................................................    6098.791209
................................................UGACAGAAGAGAGUGACCAC.............................................................................    646.770901
................................................UAACAGAAGAGAGUGAGCAC.............................................................................    417.580681
................................................UGACAGAAGAGAGUAAGCAC.............................................................................    488.193738
................................................UGACAGAAGAGAGUGGGC...............................................................................    77.23421
................................................UGACAGAAGAGAUUGAGCAC.............................................................................    1505.804512
................................................UGCCAGAAGAGAGUGAGCAC.............................................................................    501.894875
................................................UGAAAGAAGAGAGUGAGCAC.............................................................................    640.159167
................................................UGACAGAAGAGAGUGAGG...............................................................................    26.55614
................................................UGACAGAAGAGAGUGAGA...............................................................................    554.015096
................................................UGACAGAAGAGAGUGAGU...............................................................................    2232.969562
................................................UUACAGAAGAGAGUGAGCAC.............................................................................    1035.372591
................................................UGACCGAAGAGAGUGAGCAC.............................................................................    264.671683
................................................UGACAGAAGGGAGUGAGCA..............................................................................    13.717518
................................................UGACAGAAGAGCGUGAGCAC.............................................................................    706.951071
................................................UGACAGAAGAGAGUGAGCGC.............................................................................    1109.259318
................................................UGACAGAAGAGAGUGAAC...............................................................................    8.95173
................................................UGACAGAAGGGAGUGAGCAC.............................................................................    1001.809665
................................................UGACAGAAGAGAGUGAGAA..............................................................................    6.907639
................................................UGACAGAAGAGAGUGAGAAC.............................................................................    1871.723337
................................................UGACAGAAGAGAGUGACCA..............................................................................    3.092819
................................................UGACAAAAGAGAGUGAGCAC.............................................................................    256.326531
................................................AGACAGAAGAGAGUGAGCAC.............................................................................    528.84129
................................................UGACAGAAGAGCGUGAGCA..............................................................................    4.24841
................................................UGACAGAAGAGACUGAGCAC.............................................................................    428.37133
................................................UGACAGAAGAGUGUGAGCAC.............................................................................    165.023433
................................................UGUCAGAAGAGAGUGAGCAC.............................................................................    247.60008
................................................UGACAGAAGAGAGUGGGCA..............................................................................    26.859814
................................................UGACAGAUGAGAGUGAGCAC.............................................................................    186.63299
................................................UGACAGGAGAGAGUGAGCAC.............................................................................    894.554726
................................................UGACAGAAGUGAGUGAGCAC.............................................................................    199.47868
................................................UGACAGAAGAGAGUGAACAC.............................................................................    1044.717943
................................................UGACAGAACAGAGUGAGCAC.............................................................................    202.496003
................................................CGACAGAAGAGAGUGAGCAC.............................................................................    1214.211113
................................................UGACAGCAGAGAGUGAGCAC.............................................................................    722.109101
................................................UGACAGAAGCGAGUGAGCAC.............................................................................    495.361686
................................................UCACAGAAGAGAGUGAGCAC.............................................................................    222.85712
................................................GGACAGAAGAGAGUGAGCAC.............................................................................    348.463879
................................................UGAGAGAAGAGAGUGAGCAC.............................................................................    241.705638
................................................UGACUGAAGAGAGUGAGCAC.............................................................................    278.48864
................................................UGACAGAAGAGAGUGAGCCCA............................................................................    4.8288
................................................UGACAGAAGAGAAUGAGCAC.............................................................................    260.689562
................................................UGACAGAAGACAGUGAGCAC.............................................................................    173.019221
................................................UGACAGAAUAGAGUGAGCAC.............................................................................    480.6204
................................................UGACAGAAGAGAGUGUGCAC.............................................................................    252.70159
................................................UGACAGUAGAGAGUGAGCAC.............................................................................    174.72889
................................................UGACAGAAGAGGGUGAGCA..............................................................................    14.015897
................................................UGACAGAAGAGAGUGCGCA..............................................................................    4.75095
................................................UGACAGAAGAGAGUGAGCAA.............................................................................    10411.15771
................................................UGACAGAAGAGAGUGAGCAU.............................................................................    9796.070943
................................................UGACAGAAGAGAGUGAUCAC.............................................................................    585.074974
................................................UGACAGAAGAGAGUGAGCAG.............................................................................    765.609921
................................................UGACAGAAGAGAUUGAGCA..............................................................................    7.261237
................................................UGACAGAAGAGGGUGAGC...............................................................................    12.5658
................................................UGGCAGAAGAGAGUGAGCAC.............................................................................    947.950764
................................................UGACAGAAGAGAGUGAGCAAA............................................................................    22.295708
................................................UGACACAAGAGAGUGAGCAC.............................................................................    157.041902
................................................UGACAGAAGAGAGUGCGCAC.............................................................................    1154.036353
................................................UGACAGAAGAGAGUGGGCAC.............................................................................    1099.312511
................................................UGACAGAAGAGAGUGAGUAC.............................................................................    588.143268
................................................UGACAGAAGAGAGUUAGCAC.............................................................................    541.985546
................................................UGACAGAAGAGAGUCAGCAC.............................................................................    235.215704
................................................UGACAGAAGAAAGUGAGCAC.............................................................................    246.464492
................................................UGACAGAAGAGAGUGAGUA..............................................................................    3.064174
................................................UGACAGAAGAGAGUGUGCA..............................................................................    10.05945
................................................UGACAGAAGAUAGUGAGCAC.............................................................................    611.235981
................................................UGACAGACGAGAGUGAGCAC.............................................................................    352.374538
................................................UGACAGAAAAGAGUGAGCAC.............................................................................    408.181761
................................................UGACAGAAGAGAGUGACC...............................................................................    39.26405
................................................UGACAUAAGAGAGUGAGCAC.............................................................................    619.31861
................................................UGACAGAGGAGAGUGAGCAC.............................................................................    903.11262
................................................UGACAGAAGAGAGUGAGCC..............................................................................    63.345774
................................................UGACAGAAGAGAGUGAGCG..............................................................................    212.845116
................................................UGACAGAAGAGAGUGAGCU..............................................................................    189.011259
................................................UGACAGAAGAGGGUGAGCAC.............................................................................    940.452296
................................................UGACAGAAGAGAGUGAGCCC.............................................................................    3924.353668
................................................UGACAGAAGAGAGUGAGAACA............................................................................    3.3907
................................................UGACAGAAGAGAGUUAGCA..............................................................................    4.904918
................................................UGACAGAAGAGAGUGAGGAC.............................................................................    190.055323
.................................................GACAGAAGAGAGUGAGCAA.............................................................................    5.75943
.................................................GACAGAAGAGAGUGAGCACU............................................................................    110.958352
..................................................ACAGAAGAGAGUGAGCACU............................................................................    100.871647
...................................................CAGAAGAGAGUGAGCACACG..........................................................................    5.132734
.........................................................................................................UGCUCACUGCUCUUUCUGUCAUC.................    61.289348
..........................................................................................................GCUCACUGCUCUUUCUGUCAU..................    63.85967
..........................................................................................................GCUCACUGCUCUUUCUGUCA...................    7.231668
..........................................................................................................GCUCACUUCUCUUUCUGUCAUC.................    317.18406
..........................................................................................................GCUCACUGCUCUUUCUGUCAUU.................    8.0654
..........................................................................................................GCUCACUGCUCUUUCUGUCAGC.................    27.263392
```

zma-MIR156i

Chromosome: chr4
Start: 140280586
End: 140280739
Strand: -
Strand bias: 1.0
Abundance bias: 1.0
MFE: -92.60

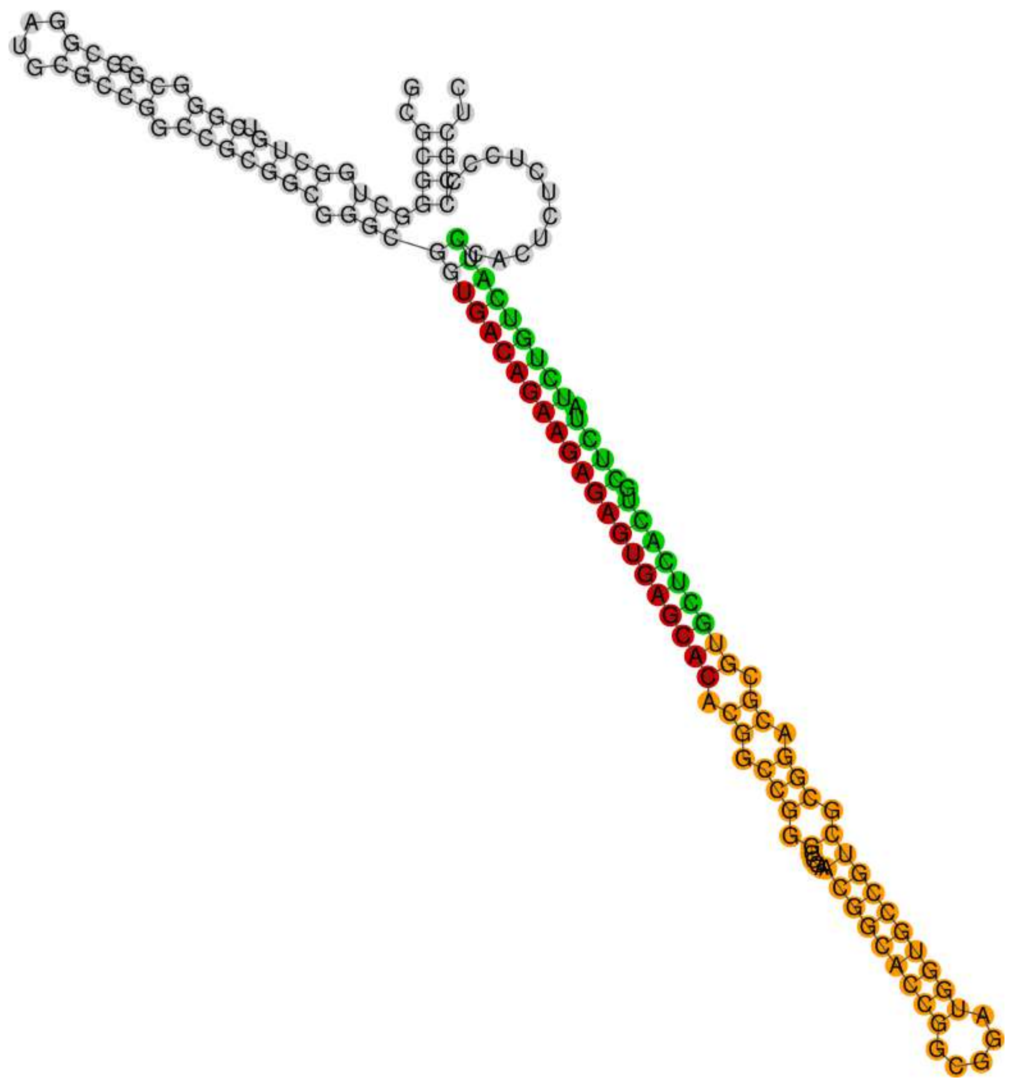

Mature — Star

GCGCGGGCUGGCUGUCGGGCGCGCGGAUGCGCCGGCCGCGGCGGGCGGUGACAGAAGAGAGUGAGCACACGGCCGGGCGGAACGGCACCGGCGGAUGGUGCCGUCGCGGACGCGUGCUCACUGCUCUAUCUGUCAUCCACUCUCUCCCCGCUC	69861.5
..(((((((.((((.(((.((.(((....))))).))))))).)))(((((((((((((((((((((.((.(((.(....((((((((((.....)))))))))).))).)).)))))))))).)))).)))))))))...........))))..	
GCUCACUGCUCUAUCUGUCAUC	2685.47272
CGGUGACAGAAGAGAGUGAGCAC	19.411009
GGUGACAGAAGAGAGUGAGCAC	26.933198
GUGACAGAAGAGAGUGAGCAC	116.752429
UGACAGAAGGGAGUGAGC	8.85506
UGACGGAAGAGAGUGAGCAC	1035.822926
UGACAGAAGAGAGUGAGCUC	281.048229
UGACAGAAGAGAGUGAGCACC	1828.279371
UGACAGAAGAGAGUGAGCACU	6098.791209
UGACAGAAGAGAGUGACCAC	646.770901
UAACAGAAGAGAGUGAGCAC	417.580681
UGACAGAAGAGAGUAAGCAC	488.193738
UGACAGAAGAGAGUGGGC	77.23421
UGACAGAAGAGAUUGAGCAC	1505.804512
UGCCAGAAGAGAGUGAGCAC	501.894875
UGAAAGAAGAGAGUGAGCAC	640.159167
UGACAGAAGAGAGUGAGG	26.55614
UGACAGAAGAGAGUGAGA	554.015096
UGACAGAAGAGAGUGAGU	2232.969562
UUACAGAAGAGAGUGAGCAC	1035.372591
UGACCGAAGAGAGUGAGCAC	264.671683
UGACAGAAGGGAGUGAGCA	13.717518
UGACAGAAGAGCGUGAGCAC	706.951071
UGACAGAAGAGAGUGAGCGC	1109.259318
UGACAGAAGAGAGUGAAC	8.95173
UGACAGAAGGGAGUGAGCAC	1001.809665
UGACAGAAGAGAGUGAGAA	6.907639
UGACAGAAGAGAGUGAGAAC	1871.723337
UGACAAAAGAGAGUGAGCAC	256.326531
AGACAGAAGAGAGUGAGCAC	528.84129
UGACAGAAGAGCGUGAGCA	4.24841
UGACAGAAGAGACUGAGCAC	428.37133
UGACAGAAGAGUGUGAGCAC	165.023433
UGUCAGAAGAGAGUGAGCAC	247.60008
UGACAGAAGAGAGUGGGCA	26.859814
UGACAGAUGAGAGUGAGCAC	186.63299
UGACAGGAGAGAGUGAGCAC	894.554726
UGACAGAAGUGAGUGAGCAC	199.47868
UGACAGAAGAGAGUGAACAC	1044.717943
UGACAGAACAGAGUGAGCAC	202.496003
CGACAGAAGAGAGUGAGCAC	1214.211113
UGACAGCAGAGAGUGAGCAC	722.109101
UGACAGAAGCGAGUGAGCAC	495.361686
UCACAGAAGAGAGUGAGCAC	222.85712
GGACAGAAGAGAGUGAGCAC	348.463879
UGAGAGAAGAGAGUGAGCAC	241.705638
UGACUGAAGAGAGUGAGCAC	278.48864
UGACAGAAGAGAGUGAGCCCA	4.8288
UGACAGAAGAGAAUGAGCAC	260.689562
UGACAGAAGACAGUGAGCAC	173.019221
UGACAGAAUAGAGUGAGCAC	480.6204
UGACAGAAGAGAGUGUGCAC	252.70159
UGACAGUAGAGAGUGAGCAC	174.72889
UGACAGAAGAGGGUGAGCA	14.015897
UGACAGAAGAGAGUGCGCA	4.75095
UGACAGAAGAGAGUGAGCAA	10411.15771
UGACAGAAGAGAGUGAGCAU	9796.070943
UGACAGAAGAGAGUGAUCAC	585.074974
UGACAGAAGAGAGUGAGCAG	765.609921
UGACAGAAGAGAUUGAGCA	7.261237
UGACAGAAGAGGGUGAGC	12.5658
UGGCAGAAGAGAGUGAGCAC	947.950764
UGACAGAAGAGAGUGAGCAAA	22.295708
UGACACAAGAGAGUGAGCAC	157.041902
UGACAGAAGAGAGUGCGCAC	1154.036353
UGACAGAAGAGAGUGGGCAC	1099.312511
UGACAGAAGAGAGUGAGUAC	588.143268
UGACAGAAGAGAGUUAGCAC	541.985546
UGACAGAAGAGAGUCAGCAC	235.215704
UGACAGAAGAAAGUGAGCAC	246.464492
UGACAGAAGAGAGUGUGCA	10.05945
UGACAGAAGAUAGUGAGCAC	611.235981
UGACAGACGAGAGUGAGCAC	352.374538
UGACAGAAAAGAGUGAGCAC	408.181761
UGACAGAAGAGAGUGACC	39.26405
UGACAUAAGAGAGUGAGCAC	619.31861
UGACAGAGGAGAGUGAGCAC	903.11262
UGACAGAAGAGAGUGAGCC	63.345774
UGACAGAAGAGAGUGAGCG	212.845116
UGACAGAAGAGAGUGAGCU	189.011259
UGACAGAAGAGGGUGAGCAC	940.452296
UGACAGAAGAGAGUGAGCCC	3924.353668
UGACAGAAGAGAGUGAGAACA	3.3907
UGACAGAAGAGAGUUAGCA	4.904918
UGACAGAAGAGAGUGAGGAC	190.055323
GACAGAAGAGAGUGAGCAA	5.75943
GACAGAAGAGAGUGAGCACU	110.958352
ACAGAAGAGAGUGAGCACU	100.871647
CAGAAGAGAGUGAGCACACG	5.132734
GCUCACUGCUCUAUCUGUCA	67.024856
GCUCACUGCUCUAUCUGUC	12.88541
GCUCACUGCUCUAUCUGUCAUCC	5.713684
GCUCACUGCUCUAUCUGUCAU	141.525434
UCUCACUGCUCUAUCUGUCAUC	3.17727
GCUCACUGCUCUAUCUGUCAGC	5.76619
GCUCACUGCUCUAUCUGUCAAC	11.13941
GCUCACUGCUCUAUCUGUCAUU	13.024123

zma-MIR156l

Chromosome: chr5
Start: 188595316
End: 188595441
Strand: -
Strand bias: 1.0
Abundance bias: 1.0
MFE: -83.50

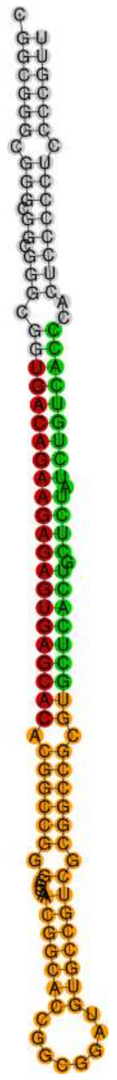

```
                    Mature                                                               Star
CGGCGGGCGGGCGGCGGGCGGUGACAGAAGAGAGUGAGCACACGGCCGGGCGGAACGGCACCGGCGGAUGUGCCGUCGCGGCCGCGUGCUCACUGCUCUAUCUGUCACCCACUCCCCCUCCCCGUU    80715.1
.((((((.(((.((.(((.((((((((((((((((((((((.((((((.(....(((((((........)))))))).)))))).))))))))).)))).)))))))))..)))))))).))))))

.......................................................................................GCUCACUGCUCUAUCUGUCACC.................    10511.76968
..................CGGUGACAGAAGAGAGUGAGCAC.....................................................................................    19.411009
...................GGUGACAGAAGAGAGUGAGCAC.....................................................................................    26.933198
....................GUGACAGAAGAGAGUGAGCAC.....................................................................................    116.752429
.....................UGACGGAAGAGAGUGAGCAC.....................................................................................    1035.822926
.....................UGACAGAAGAGAGUGAGCUC.....................................................................................    281.048229
.....................UGACAGAAGAGAGUGAGCACC....................................................................................    1828.279371
.....................UGACAGAAGAGAGUGAGCACU....................................................................................    6098.791209
.....................UGACAGAAGAGAGUGACCAC.....................................................................................    646.770901
.....................UAACAGAAGAGAGUGAGCAC.....................................................................................    417.580681
.....................UGACAGAAGAGAGUAAGCAC.....................................................................................    488.193738
.....................UGACAGAAGAGAGUGGGC.......................................................................................    77.23421
.....................UGACAGAAGAGAUUGAGCAC.....................................................................................    1505.804512
.....................UGCCAGAAGAGAGUGAGCAC.....................................................................................    501.894875
.....................UGAAAGAAGAGAGUGAGCAC.....................................................................................    640.159167
.....................UGACAGAAGAGAGUGAGG.......................................................................................    26.55614
.....................UGACAGAAGAGAGUGAGA.......................................................................................    554.015096
.....................UGACAGAAGAGAGUGAGU.......................................................................................    2232.969562
.....................UUACAGAAGAGAGUGAGCAC.....................................................................................    1035.372591
.....................UGACCGAAGAGAGUGAGCAC.....................................................................................    264.671683
.....................UGACAGAAGGGAGUGAGCA......................................................................................    13.717518
.....................UGACAGAAGAGCGUGAGCAC.....................................................................................    706.951071
.....................UGACAGAAGAGAGUGAGCGC.....................................................................................    1109.259318
.....................UGACAGAAGGGAGUGAGCAC.....................................................................................    1001.809665
.....................UGACAGAAGAGAGUGAGAAC.....................................................................................    1871.723337
.....................UGACAAAAGAGAGUGAGCAC.....................................................................................    256.326531
.....................AGACAGAAGAGAGUGAGCAC.....................................................................................    528.84129
.....................UGACAGAAGAGACUGAGCAC.....................................................................................    428.37133
.....................UGACAGAAGAGUGUGAGCAC.....................................................................................    165.023433
.....................UGUCAGAAGAGAGUGAGCAC.....................................................................................    247.60008
.....................UGACAGAAGAGAGUGGGCA......................................................................................    26.859814
.....................UGACAGAUGAGAGUGAGCAC.....................................................................................    186.63299
.....................UGACAGGAGAGAGUGAGCAC.....................................................................................    894.554726
.....................UGACAGAAGUGAGUGAGCAC.....................................................................................    199.47868
.....................UGACAGAAGAGAGUGAACAC.....................................................................................    1044.717943
.....................UGACAGAACAGAGUGAGCAC.....................................................................................    202.496003
.....................CGACAGAAGAGAGUGAGCAC.....................................................................................    1214.211113
.....................UGACAGCAGAGAGUGAGCAC.....................................................................................    722.109101
.....................UGACAGAAGCGAGUGAGCAC.....................................................................................    495.361686
.....................UCACAGAAGAGAGUGAGCAC.....................................................................................    222.85712
.....................GGACAGAAGAGAGUGAGCAC.....................................................................................    348.463879
.....................UGAGAGAAGAGAGUGAGCAC.....................................................................................    241.705638
.....................UGACUGAAGAGAGUGAGCAC.....................................................................................    278.48864
.....................UGACAGAAGAGAAUGAGCAC.....................................................................................    260.689562
.....................UGACAGAAGACAGUGAGCAC.....................................................................................    173.019221
.....................UGACAGAAUAGAGUGAGCAC.....................................................................................    480.6204
.....................UGACAGAAGAGAGUGUGCAC.....................................................................................    252.70159
.....................UGACAGUAGAGAGUGAGCAC.....................................................................................    174.72889
.....................UGACAGAAGAGGGUGAGCA......................................................................................    14.015897
.....................UGACAGAAGAGAGUGAGCAA.....................................................................................    10411.15771
.....................UGACAGAAGAGAGUGAGCAU.....................................................................................    9796.070943
.....................UGACAGAAGAGAGUGAUCAC.....................................................................................    585.074974
.....................UGACAGAAGAGAGUGAGCAG.....................................................................................    765.609921
.....................UGACAGAAGAGGGUGAGC.......................................................................................    12.5658
.....................UGGCAGAAGAGAGUGAGCAC.....................................................................................    947.950764
.....................UGACAGAAGAGAGUGAGCAAA....................................................................................    22.295708
.....................UGACACAAGAGAGUGAGCAC.....................................................................................    157.041902
.....................UGACAGAAGAGAGUGCGCAC.....................................................................................    1154.036353
.....................UGACAGAAGAGAGUGGGCAC.....................................................................................    1099.312511
.....................UGACAGAAGAGAGUGAGUAC.....................................................................................    588.143268
.....................UGACAGAAGAGAGUUAGCAC.....................................................................................    541.985546
.....................UGACAGAAGAGAGUCAGCAC.....................................................................................    235.215704
.....................UGACAGAAGAAAGUGAGCAC.....................................................................................    246.464492
.....................UGACAGAAGAGAGUGUGCA......................................................................................    10.05945
.....................UGACAGAAGAUAGUGAGCAC.....................................................................................    611.235981
.....................UGACAGACGAGAGUGAGCAC.....................................................................................    352.374538
.....................UGACAGAAAAGAGUGAGCAC.....................................................................................    408.181761
.....................UGACAGAAGAGAGUGACC.......................................................................................    39.26405
.....................UGACAUAAGAGAGUGAGCAC.....................................................................................    619.31861
.....................UGACAGAGGAGAGUGAGCAC.....................................................................................    903.11262
.....................UGACAGAAGAGAGUGAGCC......................................................................................    63.345774
.....................UGACAGAAGAGAGUGAGCG......................................................................................    212.845116
.....................UGACAGAAGAGAGUGAGCU......................................................................................    189.011259
.....................UGACAGAAGAGGGUGAGCAC.....................................................................................    940.452296
.....................UGACAGAAGAGAGUGAGCCC.....................................................................................    3924.353668
.....................UGACAGAAGAGAGUGAGGAC.....................................................................................    190.055323
......................GACAGAAGAGAGUGAGCACU....................................................................................    110.958352
.......................ACAGAAGAGAGUGAGCACU....................................................................................    100.871647
......................................................................................UGCUCACUGCUCUAUCUGUCACC.................    2132.075868
......................................................................................UGCUCACUGCUCUAUCUGUCAC..................    84.79067
......................................................................................UGCUCACUGCUCUAUCUGUCACU.................    46.411752
......................................................................................UGCUCACUGCUCUAUCUGUCACA.................    11.59303
.......................................................................................GCUCACUGCUCUAUCUGUCA...................    67.024856
.......................................................................................GCUCACUGCUCUAUCUGUCAC..................    301.633468
.......................................................................................GCUCACUGCUCUAUCUGUC....................    12.88541
.......................................................................................GCUCACUGCUCUAUCUGUCACA.................    76.187575
.......................................................................................GCUCACUGCUCUAUCUGUCACU.................    274.889151
.......................................................................................GCUCACUGCUCUCUCUGUCACC.................    12.542391
.......................................................................................GCUCAUUGCUCUAUCUGUCACC.................    19.665548
.......................................................................................GCUCACUGCUCUAUCCGUCACC.................    9.787331
.......................................................................................GCUCACUGCUUUAUCUGUCACC.................    10.16636
.......................................................................................GCUUACUGCUCUAUCUGUCACC.................    13.877461
.......................................................................................GCUCACUGCUCUAUCUGUCAAC.................    11.13941
.......................................................................................GCUCACUGCCCUAUCUGUCACC.................    18.615972
.......................................................................................GCUCACUGCUCUAUCUGCCACC.................    12.292809
.......................................................................................GUUCACUGCUCUAUCUGUCACC.................    18.036891
........................................................................................CUCACUGCUCUAUCUGUCACC.................    18.683889
```

zma-MIR156_N1

```
Chromosome:    chr3
Start:         6928343
End:           6928623
Strand:        -
Strand bias:   1.0
Abundance bias:1.0
MFE:           -80.20
```

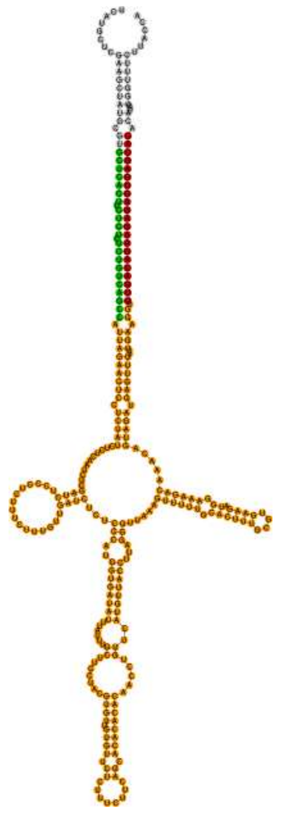

```
                    Star                                                                                                                                                                                                                                Mature
UCAUGCUCGAAGCUAUGCGUGCUCACUUCUCUCUCUGUCAGCCAUUAGAACUCCUCUAUCUCUCAAUCUCGAUCUCCCUCUUUCUUUGUUGAUCUCUCCCAUGGUGAUAUUUAUUUGCUUCCUACGUGUUGUGUUCUCUUUCUUCAGCACACACACAACCUGUUCAUGUUACCUUAGGGUUAAAGUUUUUGCACUUUGCGUGAAGAUGGAAAGACAAACAGUAGAUGAGUUUUUUGAAGGUUUGACAGAAGAGAGUGAGCACACACGGUGGUUUCUUACCA    606267.6
........((((((((.((((((((.((((.(((((((((.((((((((.(((((...........((((................))))...(((..((((((((......((.......((((.((((.((........)).))))))))).....))..))))))))...))).....((((((.(((((....)))).)).))))))).....))))).))))))..))).)).)))))))))))))))))))))).))...)))))))......
.................................................................................................................................................................................................................................................UUGACAGAAGAGAGUGAGCAC...................    454754.154608
..................................................................................................................................................................................................................................................UGACAGAAGAGAGUGAGCACC..................    1828.279371
..................................................................................................................................................................................................................................................UGACAGAAGAGAGUGAGCACU..................    6098.791209
..................................................................................................................................................................................................................................................UGACAGAAGAGAGUGACCAC...................    646.770901
..................................................................................................................................................................................................................................................UGACAGAAGAGAGUAAGCAC...................    488.193738
..................................................................................................................................................................................................................................................UGACAGAAGAGAUUGAGCAC...................    1505.804512
..................................................................................................................................................................................................................................................UGACAGAAGAGAGUGAGA.....................    554.015096
..................................................................................................................................................................................................................................................UGACAGAAGAGAGUGAGU.....................    2232.969562
..................................................................................................................................................................................................................................................UGACAGAAGAGCGUGAGCAC...................    706.951071
..................................................................................................................................................................................................................................................UGACAGAAGAGAGUGAGCGC...................    1109.259318
..................................................................................................................................................................................................................................................UGACAGAAGGGAGUGAGCAC...................    1001.809665
..................................................................................................................................................................................................................................................UGACAGAAGAGAGUGAGAAC...................    1871.723337
..................................................................................................................................................................................................................................................UGACAGAAGAGACUGAGCAC...................    428.37133
..................................................................................................................................................................................................................................................UGACAGAAGAGAGUGAACAC...................    1044.717943
..................................................................................................................................................................................................................................................UGACAGAAGCGAGUGAGCAC...................    495.361686
..................................................................................................................................................................................................................................................UGACAGAAUAGAGUGAGCAC...................    480.6204
..................................................................................................................................................................................................................................................UGACAGAAGAGAGUGAGCAA...................    10411.15771
..................................................................................................................................................................................................................................................UGACAGAAGAGAGUGAGCAU...................    9796.070943
..................................................................................................................................................................................................................................................UGACAGAAGAGAGUGAUCAC...................    585.074974
..................................................................................................................................................................................................................................................UGACAGAAGAGAGUGAGCAG...................    765.609921
..................................................................................................................................................................................................................................................UGACAGAAGAGAGUGCGCAC...................    1154.036353
..................................................................................................................................................................................................................................................UGACAGAAGAGAGUGGGCAC...................    1099.312511
..................................................................................................................................................................................................................................................UGACAGAAGAGAGUGAGUAC...................    588.143268
..................................................................................................................................................................................................................................................UGACAGAAGAGAGUUAGCAC...................    541.985546
..................................................................................................................................................................................................................................................UGACAGAAGAUAGUGAGCAC...................    611.235981
..................................................................................................................................................................................................................................................UGACAGACGAGAGUGAGCAC...................    352.374538
..................................................................................................................................................................................................................................................UGACAGAAAAGAGUGAGCAC...................    408.181761
..................................................................................................................................................................................................................................................UGACAGAGGAGAGUGAGCAC...................    903.11262
..................................................................................................................................................................................................................................................UGACAGAAGAGGGUGAGCAC...................    940.452296
..................................................................................................................................................................................................................................................UGACAGAAGAGAGUGAGCCC...................    3924.353668
.................................................................................................................................................................................................................................................UUGACAGAAGAGAGUGAGCA....................    5663.212102
.................................................................................................................................................................................................................................................UUGACAGAAGAGAGUGAGC.....................    7053.501975
.................................................................................................................................................................................................................................................UUGACAGAAGAGAGUGAGCACA..................    585.977629
.................................................................................................................................................................................................................................................UUGACAGAAGAGAGCGAGCAC...................    49762.477181
.................................................................................................................................................................................................................................................UUGACGGAAGAGAGUGAGCAC...................    383.686177
.................................................................................................................................................................................................................................................UUGACAGAAGAGAGUGAGCCC...................    1011.740781
.................................................................................................................................................................................................................................................UUGACAGAAGAGAGUGAGU.....................    1342.258838
.................................................................................................................................................................................................................................................UUGACAGAAGAGAGUGAGA.....................    337.190553
.................................................................................................................................................................................................................................................UUGACAGAAGAGAUUGAGCAC...................    521.73085
.................................................................................................................................................................................................................................................UUGACAGAAGAGAGUGAGCACC..................    551.421093
.................................................................................................................................................................................................................................................UUGACAGAAGAGAGUGAGCACU..................    1633.850316
.................................................................................................................................................................................................................................................UUGACAGAAGGGAGUGAGCAC...................    368.058968
.................................................................................................................................................................................................................................................UUGACAGAAGAGAGCGAGC.....................    625.846718
.................................................................................................................................................................................................................................................UUGACAGAGGAGAGUGAGCAC...................    350.73692
.................................................................................................................................................................................................................................................UCGACAGAAGAGAGUGAGCAC...................    474.923344
.................................................................................................................................................................................................................................................UUGACAGAAGAGAGUGAGAAC...................    624.690479
.................................................................................................................................................................................................................................................UUGGCAGAAGAGAGUGAGCAC...................    424.222591
.................................................................................................................................................................................................................................................UUGACAGAAGAGGGUGAGCAC...................    392.089028
.................................................................................................................................................................................................................................................UUGACAGAAGAGAGUGGGCAC...................    525.521175
.................................................................................................................................................................................................................................................UUGACAGAAGAGAGUGCGCAC...................    497.568994
.................................................................................................................................................................................................................................................UUGACAGAAGAGAGGGAGCAC...................    433.26253
.................................................................................................................................................................................................................................................UUGACAGAAGAGAGCGAGCA....................    319.290445
.................................................................................................................................................................................................................................................UUGACAGAAGAGAGUGAGCAA...................    1999.230613
.................................................................................................................................................................................................................................................UUGACAGAAGAGAGUGAGCAU...................    5226.120249
.................................................................................................................................................................................................................................................UUGACAGAAGAGAGUGAGCGC...................    526.33982
....................GCUCACUUCUCUCUCUGUCAGC...............................................................................................................................................................................................................................    6091.05944
....................GCUCACUUCUCUCUCUGUCAG................................................................................................................................................................................................................................    365.46719
```

zma-MIR160f

```
Chromosome:    chr2
Start:         241093417
End:           241093549
Strand:        -
Strand bias:   1.0
Abundance bias:0.983
MFE:           -63.40
```

```
                  Mature                                                    Star
GGUGAAAGAUAGGGAAUAUGCCUGGCUCCCUGUAUGCCGCUCGCAUGGCUACCACCGCCUGUGGUCGUUGCGGCUGGCGUGCGAGGUGCCAGGCAUGUUUUAAUAAUUAUCUCCCUCCCCCCUCUCUCUCAUA    4679.6
.((((.(((.((((((((((((((((.((.((((((((((.(((((((((((........))))))).)))))).)))))))).)).))))))))))))....................)))).))).)))).
..................UGCCUGGCUCCCUGUAUGCC...............................................................................................    1682.996037
.................AUGCCUGGCUCCCUGUAUGC................................................................................................    7.57396
.................AUGCCUGGCUCCCUGUAUGCC...............................................................................................    312.148988
..................UGCCUGGCUCCCUGUAUG.................................................................................................    240.304981
..................UGCCUGGCUCCCUGUAUGC................................................................................................    715.659827
..................UGCCUGGCUCCCUGUAUGCCG..............................................................................................    223.631741
..................UGCCUGGCUCCCUGUAUUC................................................................................................    13.3184
..................UGCCUGGCUCCCUGUAUGCA...............................................................................................    164.345598
..................UGCCUGGCUCCCUGUAUA.................................................................................................    4.73897
..................UGCCUGGCUCCCUGUAUC.................................................................................................    1.69914
..................UGCCUGGCUCCCUGUAUU.................................................................................................    63.701248
..................UGCCUGGCUCCCUGUAUGCG...............................................................................................    0.72271
..................UGCCUGGCUCCCUGUAUGCU...............................................................................................    605.924741
..................UGCCUGGCUCCCUGUAUGA................................................................................................    14.94996
..................UGCCUGGCUCCCUGUAUGU................................................................................................    32.487343
..................UGCCUGGCUCCCUGUAUGCCC..............................................................................................    56.852869
..................UGCCUGGCUCCCUGUAUGCCU..............................................................................................    460.805045
```

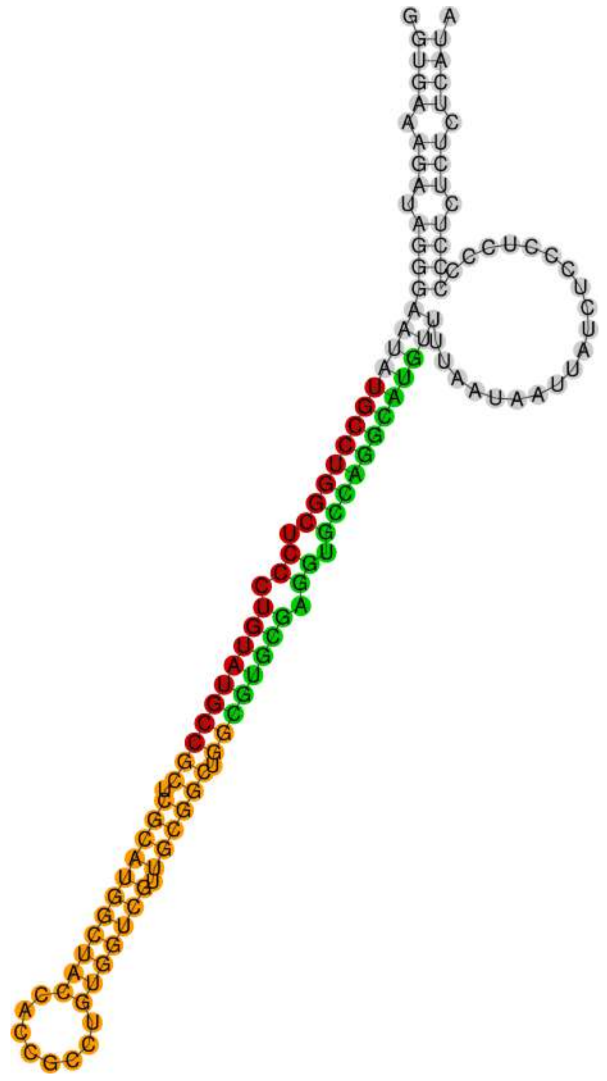

zma-MIR164f

```
Chromosome:    chr4
Start:         179535492
End:           179535629
Strand:        +
Strand bias:   1.0
Abundance bias:1.0
MFE:           -80.40
```

```
                 Mature                                                                       Star
GGGCGAGCAAACCGUGCUGGAGAAGCAGGGCACGUGCUCGCCGACAAGUCGAAGCCUUAGGGCAGCUUGGCCUGUGGCUGCACGCGUACGUGGUCGUCGCAGCACGUGCGCUCCUUCUCCAACAUGGCUUCUCGCCCC    6049.2
(((((((.((.(((((.((((((((.((.(((((((((((.((((.......((((.((((.((...)).)))).)))).((((....)))))))).)).))))))))).)).)))))))).))))).))))))))).
.................UGGAGAAGCAGGGCACGUGC.....................................................................................................    3089.04901
..............UGCUGGAGAAGCAGGGCACGU.......................................................................................................    20.190755
................CUGGAGAAGCAGGGCACGUGC.....................................................................................................    21.374255
.................UGGAGAAGCAGGGCACGU.......................................................................................................    164.379322
.................UGGAGAAGCAGGGCACGUGCU....................................................................................................    1299.31139
.................UGGAGAAGCAGGGCACGUG......................................................................................................    489.534144
.................UGGAGAAGCAGGGCACGUGA.....................................................................................................    175.819208
.................UGGAGAAGCAGGGCACGUGG.....................................................................................................    3.6078
.................UGGAGAAGCAGGGCACGUGCG....................................................................................................    368.762493
.................UGGAGAAGCAGGGCACGCGC.....................................................................................................    1.55963
.................UGGAGAAGCAGGGAACGUGCU....................................................................................................    1.91323
.................UGGAGAAGCAGGGCACGUGCC....................................................................................................    212.266876
.................UGGAGAAGCAGGGCACGUU......................................................................................................    9.3391
.................UGGAGAAGCAGGGCACGUA......................................................................................................    3.62316
.................UGGAGAAGCAGGGCACGC.......................................................................................................    3.80678
.................UGGAGAAGCAGGGCACGUGUU....................................................................................................    14.11516
.................UGGAGAAGCAGGGCCCGUGC.....................................................................................................    3.250584
.................UGGAGAAGUAGGGCACGUGCU....................................................................................................    0.182396
.................UGGAGAAGCAGGGCACGUGUUC...................................................................................................    1.69076
.................UGGAGAAGCAGGGCCCGUGCU....................................................................................................    1.992155
.................UGGAGAAGCAGGGCACGUCC.....................................................................................................    1.83618
.................UGGAGAAGCAGGGCACGUUC.....................................................................................................    1.651336
..................GGAGAAGCAGGGCACGUG......................................................................................................    6.55789
..................GGAGAAGCAGGGCACGUGC.....................................................................................................    135.908292
..................GGAGAAGCAGGGCACGUGCU....................................................................................................    6.36501
..................GGAGAAGCAGGGCACGUGCG....................................................................................................    2.70031
......................................................................................................CACGUGCGCUCCUUCUCCAGC...............    8.43597
```

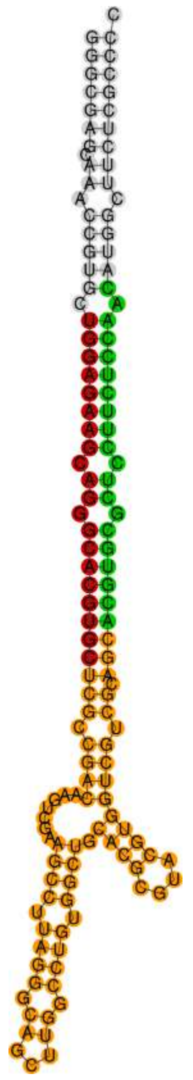

zma-MIR166f

Chromosome: chr5
Start: 8289172
End: 8289279
Strand: +
Strand bias: 1.0
Abundance bias:1.0
MFE: -62.40

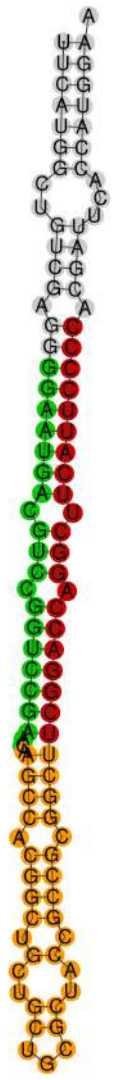

```
                 Star                                                          Mature
UUCAUGGCUGUCGAGGGGAAUGACGUCCGGUCCGAACAAGCCACGGCUGCUGCUGCGCUACCGCCGCGGCUUCGGACCAGGCUUCAUUCCCCACGAUUCACCAUGGAA    3499832.
((((((..((((.(((((((((.(((.(((((((...((((.((((.(..((...))..).)))).)))))))))))).))).))))))))).))))...))))))).
.......................................................................UCGGACCAGGCUUCAUUCCCC................    2961896.0766
.........................................................................GGACCAGGCUUCAUUCCCC................    850.718575
........................................................................CGGACCAGGCUUCAUUCCCC................    1981.517186
.......................................................................UCGGACCAGGCUUCAUUCCC.................    25276.65239
.......................................................................UCGGACCAGGCUUCAUUCCCCA...............    15301.964877
.......................................................................UUGGACCAGGCUUCAUUCCCC................    6166.826765
.......................................................................UCGGACGAGGCUUCAUUCCCC................    170.447553
.......................................................................UCGGACCAGGCUUUAUUCCCC................    3533.163648
.......................................................................UCGGACCAGGCUUCAGUCCCC................    1007.658931
.......................................................................UCGGACCAGGCGUCAUUCCCC................    834.884155
.......................................................................UCGGACCAGGCUUCACUCCCC................    1291.80307
.......................................................................ACGGACCAGGCUUCAUUCCCC................    995.77943
.......................................................................UCGGACCAGGCUUCAUUCCGC................    519.213536
.......................................................................UCGGACCAGGCUUCCUUCCCC................    8766.353245
.......................................................................UCAGACCAGGCUUCAUUCCCC................    1351.88353
.......................................................................UCGGACCAGGCUUCAUUUCCC................    4299.307935
.......................................................................UCGGACCAGGCUUCAUUU...................    896.57008
.......................................................................UCGUACCAGGCUUCAUUCCCC................    3689.419818
.......................................................................UCGGACCAGGCCUCAUUCCCC................    1796.88093
.......................................................................CCGGACCAGGCUUCAUUCCCC................    1566.461868
.......................................................................UCGCACCAGGCUUCAUUCCCC................    563.824914
.......................................................................UCGGACCAGGUUUCAUUCCCC................    1530.66488
.......................................................................UCGGACCAGGCUUCAUUCCA.................    882.275967
.......................................................................UCGGACCAGGCUUCAUUCCCA................    19371.06785
.......................................................................UCGGACCAGGCUUCAUUCCCG................    1866.084944
.......................................................................UCGGACCAGGCUUCAUCC...................    213.397153
.......................................................................UCGGACCAGGCUUCGUUCCCC................    2381.191261
.......................................................................UCGGACCAGGCUUCAUUCACC................    1299.806243
.......................................................................UGGGACCAGGCUUCAUUCCCC................    548.708034
.......................................................................UCGGACCGGGCUUCAUUCCCC................    1473.378566
.......................................................................UCGGACAAGGCUUCAUUCCCC................    871.121313
.......................................................................UCGGACCUGGCUUCAUUCCCC................    644.270222
.......................................................................UCGGACCCGGCUUCAUUCCCC................    464.050619
.......................................................................UCGGACCAGACUUCAUUCCCC................    742.625098
.......................................................................UCGGACCAGGCUCCAUUCCCC................    1557.683496
.......................................................................UCUGACCAGGCUUCAUUCCCC................    3855.713456
.......................................................................UCGGACCAGGCUUCAAUCCCC................    6228.669769
.......................................................................UCGGACCAGGCUUCAUGCCCC................    742.940595
.......................................................................UCGGACCAGGCUUGAUUCCCC................    318.168133
.......................................................................UCGGACCAGGCUUCAUUCUC.................    223.097641
.......................................................................UCGGACCACGCUUCAUUCCCC................    177.12601
.......................................................................UCGGGCCAGGCUUCAUUCCCC................    2017.66487
.......................................................................UCGGACCAGGCUUCAUCCCC.................    201.411416
.......................................................................UCGGACCAGGCUUCAUUUC..................    791.817105
.......................................................................UCGGACCAGGCUUCAUUGCCC................    325.308415
.......................................................................UCGGAACAGGCUUCAUUCCCC................    305.306124
.......................................................................UAGGACCAGGCUUCAUUCCCC................    1004.401772
.......................................................................UCGGACCAGGCUUCAUUCCAC................    1433.934665
.......................................................................UCGGACCAGGCUUCAUUCGCC................    168.967216
.......................................................................UCGGACCAGGCUUCAUUACCC................    1394.886589
.......................................................................UCGGAUCAGGCUUCAUUCCCC................    1152.339525
.......................................................................GCGGACCAGGCUUCAUUCCCC................    1373.489964
.......................................................................UCGGACCAGGCUUCUUUCCCC................    1025.081502
.......................................................................UCGGACCAGCCUUCAUUCCCC................    249.973803
.......................................................................UCGGACCAGGCUUAAUUCCCC................    2595.787516
.......................................................................UCGGACCAGGCUUCAUACCCC................    615.957197
.......................................................................UCGGACCAGGCAUCAUUCCCC................    304.313191
.......................................................................UCGGACCAGUCUUCAUUCCCC................    1698.045668
.......................................................................UCGGACCAGGAUUCAUUCCCC................    3361.421404
.......................................................................UCGAACCAGGCUUCAUUCCCC................    1222.225559
.......................................................................UCGGACCAUGCUUCAUUCCCC................    2915.09744
.......................................................................UCGGACCAGGCUGCAUUCCCC................    494.068714
.......................................................................UCGGACCAGGCUUCAUCCCCC................    3088.3769
.......................................................................UCGGACCAGGGUUCAUUCCCC................    1443.742425
.......................................................................UCGGACUAGGCUUCAUUCCCC................    1395.703481
.......................................................................UCGGACCAAGCUUCAUUCCCC................    754.854373
.......................................................................UCGGACCAGGCUUCAUUCCCU................    54756.65173
.......................................................................UCGGACCAGGCUACAUUCCCC................    282.049971
.......................................................................UCGGACCAGGCUUCAUUCA..................    774.926224
.......................................................................UCCGACCAGGCUUCAUUCCCC................    642.164282
.......................................................................UCGGACCAGGCUUCAUUCUCC................    3115.46708
.......................................................................UCGGCCCAGGCUUCAUUCCCC................    380.166369
.......................................................................UCGGACCAGGCUUCAUUCU..................    5070.280857
.......................................................................UCGGUCCAGGCUUCAUUCCCC................    488.345507
.......................................................................UCGGACCAGGCUUCAUCCC..................    590.988535
......................................................................UUCGGACCAGGCUUCAUUC...................    549.988645
......................................................................UUCGGACCAGGCUUCAUUCCCC................    194.236285
......................................................................UUCGGACCAGGCUUCAUUCC..................    1196.755557
......................................................................UUCGGACCAGGCUUCAUUCCC.................    292333.56287
......................................................................UUCGGACCAGGCUUCCUUCCC.................    260.79545
......................................................................UUCGGACCAGGCUUCAUUCCCA................    325.754909
......................................................................UUCGGACCAGUCUUCAUUCCC.................    654.632519
......................................................................UUCGGACCAGGCUUCAUUCU..................    188.659332
......................................................................UUCGGACCAUGCUUCAUUCCC.................    204.87594
......................................................................UUCGGACCAGGCUUCAUUUCC.................    233.314464
......................................................................UUCGGACCAGGCUUCAUUCUC.................    451.13295
......................................................................UUCGGACCAGGCUUAAUUCCC.................    166.52754
......................................................................UUCGGACCAGGCUUCAUUCCG.................    185.760058
......................................................................UUCGGACCAGGCUUCAUUCCA.................    2347.486747
......................................................................UUCGGACCAGGCUUCAUUCCU.................    6167.484099
......................................................................UUCGGACCAGGCUUUAUUCCC.................    206.792225
......................................................................UUCGGACCAGGAUUCAUUCCC.................    143.759502
......................................................................UUCGGACCAGGCUUCAUCCCC.................    159.448734
.....................................................................CUUCGGACCAGGCUUCAUUCCC.................    1000.402142
.................GAAUGACGUCCGGUCCGAAC.......................................................................    193.252362
.................GAAUGACGUCCGGUCCGAACA......................................................................    2173.452102
.................GAAUGACGUCCGGUCCGAACAAG....................................................................    280.488818
```

zma-MIR166h

```
Chromosome:    chr1
Start:         279585895
End:           279586022
Strand:        -
Strand bias:   1.0
Abundance bias:1.0
MFE:           -67.90
```

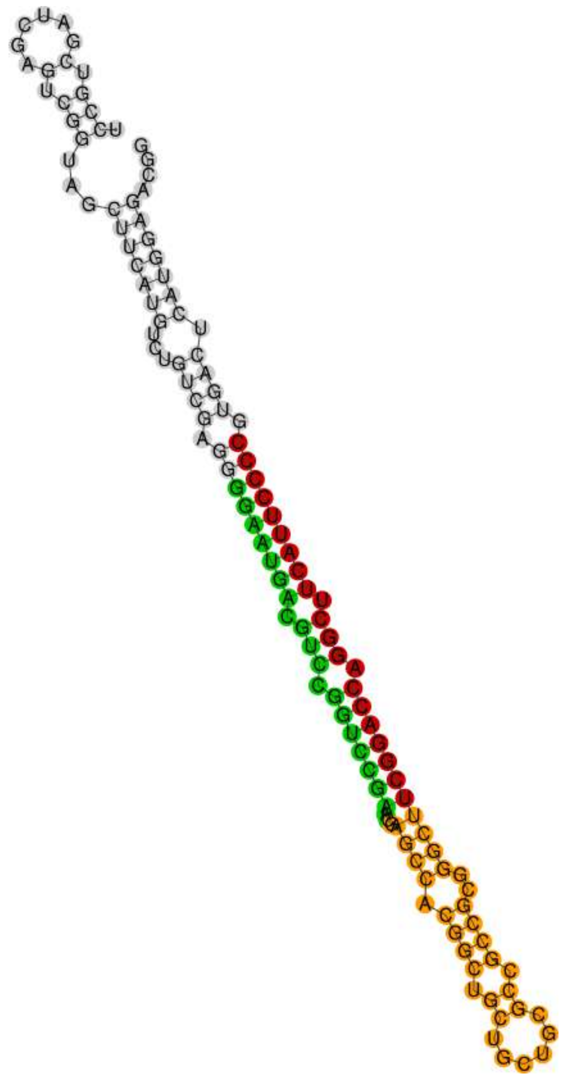

```
                                     Star                                                 Mature
UCCGUCGAUCGAGUCGGUAGCUUCAUGUCUGUCGAGGGGAAUGACGUCCGGUCCGAACGAGCCACGGCUGCUGCUGCGCCGCCGCGGGCUUCGGACCAGGCUUCAUUCCCCGUGACUCAUGGAGACGG    3484454.
.(((.(......).)))...(((((((...((((.(((((((((.(((.(((((((...((((.((((.((......)).))))..))))))))))).))).))))))))).)))).)))))))....
..........................................................................................UCGGACCAGGCUUCAUUCCCC.................    2961896.0766
............................................................................................GGACCAGGCUUCAUUCCCC.................    850.718575
...........................................................................................CGGACCAGGCUUCAUUCCCC.................    1981.517186
..........................................................................................UCGGACCAGGCUUCAUUCCC..................    25276.65239
..........................................................................................UCGGACCAGGCUUCAUUCCCCGU...............    188.838458
..........................................................................................UCGGACCAGGCUUCAUUCCCCG................    1298.842431
..........................................................................................UUGGACCAGGCUUCAUUCCCC.................    6166.826765
..........................................................................................UCGGACGAGGCUUCAUUCCCC.................    170.447553
..........................................................................................UCGGACCAGGCUUUAUUCCCC.................    3533.163648
..........................................................................................UCGGACCAGGCUUCAGUCCCC.................    1007.658931
..........................................................................................UCGGACCAGGCGUCAUUCCCC.................    834.884155
..........................................................................................UCGGACCAGGCUUCACUCCCC.................    1291.80307
..........................................................................................ACGGACCAGGCUUCAUUCCCC.................    995.77943
..........................................................................................UCGGACCAGGCUUCAUUCCGC.................    519.213536
..........................................................................................UCGGACCAGGCUUCCUUCCCC.................    8766.353245
..........................................................................................UCAGACCAGGCUUCAUUCCCC.................    1351.88353
..........................................................................................UCGGACCAGGCUUCAUUUCCC.................    4299.307935
..........................................................................................UCGGACCAGGCUUCAUUU....................    896.57008
..........................................................................................UCGUACCAGGCUUCAUUCCCC.................    3689.419818
..........................................................................................UCGGACCAGGCCUCAUUCCCC.................    1796.88093
..........................................................................................CCGGACCAGGCUUCAUUCCCC.................    1566.461868
..........................................................................................UCGCACCAGGCUUCAUUCCCC.................    563.824914
..........................................................................................UCGGACCAGGUUUCAUUCCCC.................    1530.66488
..........................................................................................UCGGACCAGGCUUCAUUCCA..................    882.275967
..........................................................................................UCGGACCAGGCUUCAUUCCCA.................    19371.06785
..........................................................................................UCGGACCAGGCUUCAUUCCCG.................    1866.084944
..........................................................................................UCGGACCAGGCUUCAUCC....................    213.397153
..........................................................................................UCGGACCAGGCUUCGUUCCCC.................    2381.191261
..........................................................................................UCGGACCAGGCUUCAUUCACC.................    1299.806243
..........................................................................................UGGGACCAGGCUUCAUUCCCC.................    548.708034
..........................................................................................UCGGACCGGGCUUCAUUCCCC.................    1473.378566
..........................................................................................UCGGACAAGGCUUCAUUCCCC.................    871.121313
..........................................................................................UCGGACCUGGCUUCAUUCCCC.................    644.270222
..........................................................................................UCGGACCCGGCUUCAUUCCCC.................    464.050619
..........................................................................................UCGGACCAGACUUCAUUCCCC.................    742.625098
..........................................................................................UCGGACCAGGCUCCAUUCCCC.................    1557.683496
..........................................................................................UCUGACCAGGCUUCAUUCCCC.................    3855.713456
..........................................................................................UCGGACCAGGCUUCAAUCCCC.................    6228.669769
..........................................................................................UCGGACCAGGCUUCAUGCCCC.................    742.940595
..........................................................................................UCGGACCAGGCUUGAUUCCCC.................    318.168133
..........................................................................................UCGGACCAGGCUUCAUUCUC..................    223.097641
..........................................................................................UCGGACCACGCUUCAUUCCCC.................    177.12601
..........................................................................................UCGGGCCAGGCUUCAUUCCCC.................    2017.66487
..........................................................................................UCGGACCAGGCUUCAUCCCC..................    201.411416
..........................................................................................UCGGACCAGGCUUCAUUUC...................    791.817105
..........................................................................................UCGGACCAGGCUUCAUUGCCC.................    325.308415
..........................................................................................UCGGAACAGGCUUCAUUCCCC.................    305.306124
..........................................................................................UAGGACCAGGCUUCAUUCCCC.................    1004.401772
..........................................................................................UCGGACCAGGCUUCAUUCCAC.................    1433.934665
..........................................................................................UCGGACCAGGCUUCAUUCGCC.................    168.967216
..........................................................................................UCGGACCAGGCUUCAUUACCC.................    1394.886589
..........................................................................................UCGGAUCAGGCUUCAUUCCCC.................    1152.339525
..........................................................................................GCGGACCAGGCUUCAUUCCCC.................    1373.489964
..........................................................................................UCGGACCAGGCUUCUUUCCCC.................    1025.081502
..........................................................................................UCGGACCAGCCUUCAUUCCCC.................    249.973803
..........................................................................................UCGGACCAGGCUUAAUUCCCC.................    2595.787516
..........................................................................................UCGGACCAGGCUUCAUACCCC.................    615.957197
..........................................................................................UCGGACCAGGCAUCAUUCCCC.................    304.313191
..........................................................................................UCGGACCAGUCUUCAUUCCCC.................    1698.045668
..........................................................................................UCGGACCAGGAUUCAUUCCCC.................    3361.421404
..........................................................................................UCGAACCAGGCUUCAUUCCCC.................    1222.225559
..........................................................................................UCGGACCAUGCUUCAUUCCCC.................    2915.09744
..........................................................................................UCGGACCAGGCUGCAUUCCCC.................    494.068714
..........................................................................................UCGGACCAGGCUUCAUCCCCC.................    3088.3769
..........................................................................................UCGGACCAGGGUUCAUUCCCC.................    1443.742425
..........................................................................................UCGGACUAGGCUUCAUUCCCC.................    1395.703481
..........................................................................................UCGGACCAAGCUUCAUUCCCC.................    754.854373
..........................................................................................UCGGACCAGGCUUCAUUCCCU.................    54756.65173
..........................................................................................UCGGACCAGGCUACAUUCCCC.................    282.049971
..........................................................................................UCGGACCAGGCUUCAUUCA...................    774.926224
..........................................................................................UCCGACCAGGCUUCAUUCCCC.................    642.164282
..........................................................................................UCGGACCAGGCUUCAUUCUCC.................    3115.46708
..........................................................................................UCGGCCCAGGCUUCAUUCCCC.................    380.166369
..........................................................................................UCGGACCAGGCUUCAUUCU...................    5070.280857
..........................................................................................UCGGUCCAGGCUUCAUUCCCC.................    488.345507
..........................................................................................UCGGACCAGGCUUCAUCCC...................    590.988535
.........................................................................................UUCGGACCAGGCUUCAUUC....................    549.988645
.........................................................................................UUCGGACCAGGCUUCAUUCCCC.................    194.236285
.........................................................................................UUCGGACCAGGCUUCAUUCC...................    1196.755557
.........................................................................................UUCGGACCAGGCUUCAUUCCC..................    292333.56287
.........................................................................................UUCGGACCAGGCUUCCUUCCC..................    260.79545
.........................................................................................UUCGGACCAGGCUUCAUUCCCA.................    325.754909
.........................................................................................UUCGGACCAGUCUUCAUUCCC..................    654.632519
.........................................................................................UUCGGACCAGGCUUCAUUCU...................    188.659332
.........................................................................................UUCGGACCAUGCUUCAUUCCC..................    204.87594
.........................................................................................UUCGGACCAGGCUUCAUUUCC..................    233.314464
.........................................................................................UUCGGACCAGGCUUCAUUCUC..................    451.13295
.........................................................................................UUCGGACCAGGCUUAAUUCCC..................    166.52754
.........................................................................................UUCGGACCAGGCUUCAUUCCG..................    185.760058
.........................................................................................UUCGGACCAGGCUUCAUUCCA..................    2347.486747
.........................................................................................UUCGGACCAGGCUUCAUUCCU..................    6167.484099
.........................................................................................UUCGGACCAGGCUUUAUUCCC..................    206.792225
........................................................................................CUUCGGACCAGGCUUCAUUCCC..................    1000.402142
......................................GAAUGACGUCCGGUCCGAAC......................................................................    193.252362
......................................GAAUGACGUCCGGUCCGAACG.....................................................................    713.269194
......................................GAAUGACGUCCGGUCCGAACGA....................................................................    995.558636
......................................GAAUGACGUCCGGUCCGAACGAG...................................................................    166.700603
```

zma-MIR166l

Chromosome: chr3
Start: 91435338
End: 91435470
Strand: +
Strand bias: 1.0
Abundance bias: 1.0
MFE: -61.90

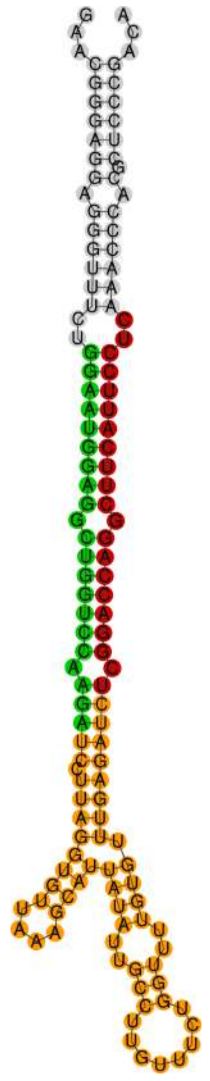

```
                       Star                                                                        Mature
GAACGGGAGGAGGGUUUCUGGAAUGGAGGCUGGUCCAAGAUCCUUAGGUGUUAAAGCAUUAUAUUGCCUUGUUUCUGGUUUUGUGUUUGAGAUCUCGGACCAGGCUUCAUUCCUCAAACCCACGCUCCCGACA    194858.3
...(((((((.((((((..(((((((((.(((((((.(((((.((((((((....))))((((..(((........)))..)))).))))))))).))))))).)))))))))..)))))).).))))))...
..............................................................................................UCGGACCAGGCUUCAUUCCUC..................    104300.9535
................................................................................................GGACCAGGCUUCAUUCCUC..................    110.36317
...............................................................................................CGGACCAGGCUUCAUUCCUC..................    65.633058
..............................................................................................UCGGACCAGGCUUCAUUCCUCAA................    26.97976
..............................................................................................UCGGACCAGGCUUCAUUCCUCA.................    68.106523
..............................................................................................UCGGACCAGACUUCAUUCCUC..................    10.479599
..............................................................................................UCGGACCAGGCUUCAUUCCCCAA................    103.452956
..............................................................................................UCGGACCAGGCUUCAUUCCGC..................    519.213536
..............................................................................................UCGGACCAGGCUUCAUUCCACA.................    13.48746
..............................................................................................UCGGACCAGGCUUCAUUU.....................    896.57008
..............................................................................................UCGGACCUGGCUUCAUUCCUC..................    15.238995
..............................................................................................UCGGACCAGGCUUUAUUCCUC..................    197.60323
..............................................................................................UCGGACCAGUCUUCAUUCCUC..................    21.194499
..............................................................................................UCGGACCAGGCUUCACUCCUC..................    79.64383
..............................................................................................UCGGACCAGGCUUAAUUCC....................    15.917613
..............................................................................................UCGGACCAGGCUUCAAUCCU...................    220.025166
..............................................................................................UCGGACCAUGCUUCAUUCCUC..................    32.016302
..............................................................................................UCGGACCAGGCUUCAUUUCUC..................    185.599463
..............................................................................................UCGGCCCAGGCUUCAUUCCUC..................    11.430085
..............................................................................................UCGGACCAGGCUUCCUUCC....................    50.891188
..............................................................................................ACGGACCAGGCUUCAUUCCUC..................    19.988167
..............................................................................................UCGGACCAGGCUUCAUUCCA...................    882.275967
..............................................................................................UCGGACCAGGCUUCAUUCCG...................    70.47754
..............................................................................................UCGGACCAGGCUUCCUUC.....................    17.30695
..............................................................................................UCGGACCAGGCUGCAUUCCU...................    56.370578
..............................................................................................UCGGACCAGGCUUCAUCC.....................    213.397153
..............................................................................................UCUGACCAGGCUUCAUUCCUC..................    52.897801
..............................................................................................UCGGACCAGGCCUCAUUCCUC..................    102.500851
..............................................................................................UCGGACCAGGCUUAAUUCCUC..................    81.513964
..............................................................................................UAGGACCAGGCUUCAUUCCUC..................    14.805695
..............................................................................................UCGGAUCAGGCUUCAUUCCUC..................    73.732414
..............................................................................................UCGGACCAGGCUUCAUUCUUC..................    657.246487
..............................................................................................UCGGACCAGGCUUCAUUCCUUA.................    52.381056
..............................................................................................UCGGACCAGGCUUCAUACCUC..................    10.639305
..............................................................................................UCGGACCAGGCUUCCUUCCUC..................    670.884554
..............................................................................................UCGGACCAGGCUUCAUUCAUC..................    40.399994
..............................................................................................UCGGACAAGGCUUCAUUCCUC..................    33.843552
..............................................................................................UCGGACCCGGCUUCAUUCCUC..................    19.533267
..............................................................................................UCGGACCAGGCUUCAUUCUU...................    588.635931
..............................................................................................UCGGACCAGGCUUCAUCCCU...................    21.31369
..............................................................................................UCGUACCAGGCUUCAUUCCUC..................    49.710609
..............................................................................................UCGGACCAGGCUUCGUUCCUC..................    123.853541
..............................................................................................UCGGACCAGGCUUCAUUUC....................    791.817105
..............................................................................................UCAGACCAGGCUUCAUUCCUC..................    50.213245
..............................................................................................UCGGACCAGGGUUCAUUCCUC..................    25.18478
..............................................................................................UCGGGCCAGGCUUCAUUCCUC..................    93.281483
..............................................................................................UCGGACCAGGCUUCUUUCCUC..................    33.4535
..............................................................................................UCGGACCAGGCUUCAUUUCU...................    316.908588
..............................................................................................UCGGACCAGGCUUCAUUCCUG..................    64.577884
..............................................................................................UCGGACCAGGCUUCAUUCCUA..................    1443.928467
..............................................................................................UCGGACCAGGCUUCAUUCCUU..................    4536.935147
..............................................................................................UCGGACCAGGCUUCAUUCCUCC.................    35.755782
..............................................................................................UCGGACCAGGCUUCAUUCCAC..................    1433.934665
..............................................................................................UCGGACCAGGUUUCAUUCCUC..................    38.046818
..............................................................................................UCGGACCAGGCUUCAGUCCUC..................    7.960367
..............................................................................................UCGGACCGGGCUUCAUUCCUC..................    85.2778
..............................................................................................UCGGACUAGGCUUCAUUCCUC..................    88.53342
..............................................................................................UCGGACCAGGCUUCAUCCCUC..................    108.432302
..............................................................................................UCCGACCAGGCUUCAUUCCUC..................    12.123586
..............................................................................................UCGGACCAGGCUUCAUUACUC..................    42.638267
..............................................................................................UCGAACCAGGCUUCAUUCCUC..................    16.600841
..............................................................................................UCGGACCAGGCUCCAUUCCUC..................    98.228261
..............................................................................................UCGGACCAGGCUUCAUUA.....................    37.313501
..............................................................................................CCGGACCAGGCUUCAUUCCUC..................    86.828145
..............................................................................................UCGGACGAGGCUUCAUUCCUC..................    11.07633
..............................................................................................UCGGACCAAGCUUCAUUCCUC..................    7.552203
..............................................................................................UCGGACCAGGCUUUAUUCC....................    23.344176
..............................................................................................UCGGACCAGGCUUCAGUCC....................    8.138179
..............................................................................................UCGGACCAGGAUUCAUUCCUC..................    101.181944
..............................................................................................UUGGACCAGGCUUCAUUCCUC..................    129.840899
..............................................................................................UCGGACCAGGCUUCAUUCCUCU.................    186.355772
..............................................................................................UCGGACCAGGCUUCAUUCA....................    774.926224
..............................................................................................UCGGACCAGGCUUCAGUC.....................    13.5645
..............................................................................................UCGGACCAGGCUUCAUUAC....................    12.309468
..............................................................................................UCGGACCAGGCUUCAUACC....................    7.82134
..............................................................................................UCGGACCAGGCUUCAUUCU....................    5070.280857
..............................................................................................UCGGACCAGGCUUCAUUCG....................    53.77499
..............................................................................................UCGGACCAGGCUUCAUCCC....................    590.988535
.............................................................................................CUCGGACCAGGCUUCAUUCCUC..................    26.172029
.............................................................................................CUCGGACCAGGCUUCAUUCCU...................    52.001263
............................................................................................UCUCGGACCAGGCUUCAUUCC....................    66118.43634
............................................................................................UCUCGGACCAGGCUUCAUUC.....................    86.021335
............................................................................................UCUCGGACCAGGCUUCUUUCC....................    16.051824
............................................................................................UCUCGGACCAGGCUUCAUUAC....................    12.87172
............................................................................................UCUCGGACCAGGCUUCAUUUC....................    24.98694
............................................................................................UCUCGGACCAGGCUUCAGUCC....................    8.73091
............................................................................................UCUCGGACCAGGCGUCAUUCC....................    17.09904
............................................................................................UCUCGGACCAGGAUUCAUUCC....................    9.707923
............................................................................................UCUCGGACCAGUCUUCAUUCC....................    88.42751
............................................................................................UCUCGGACCAGGCUUCAUUCU....................    500.66045
............................................................................................UCUCGGACCAGGCUUCAUUCA....................    169.326233
............................................................................................UCUCGGACCAGGCUUAAUUCC....................    24.190477
............................................................................................UCUCGGACCAGGCUUCCUUCC....................    18.069449
............................................................................................UCUCGGACCAGGCUUCAUUU.....................    8.03684
............................................................................................UCUCGGACCAGGCUUUAUUCC....................    12.666037
............................................................................................UCUCGGACCAUGCUUCAUUCC....................    87.88165
...........................................................................................AUCUCGGACCAGGCUUCAUUC.....................    170.31007
```

zma-MIR167e

Chromosome: chr7
Start: 10115799
End: 10115916
Strand: -
Strand bias: 1.0
Abundance bias: 0.997
MFE: -60.60

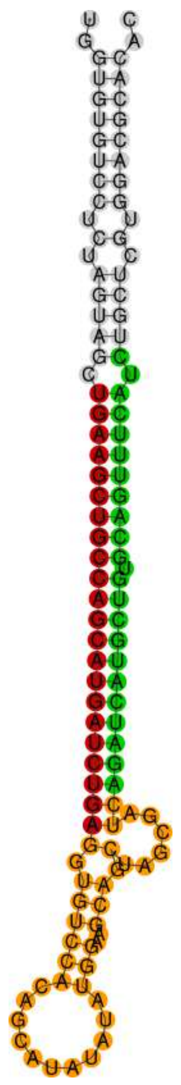

```
                   Mature                                                       Star
UGGUGUGUCCUCUAGUAGCUGAAGCUGCCAGCAUGAUCUGAGGUGUCCACAGCAUAUAUAUGGAAGCAGCUAGCGAUCAGAUCAUGCUGUGCAGUUUCAUCUGCUCGUGGACGCACAC 319021.5
..((((((((.(.(((((.(((((((((((((((((((((((.((((((...........)))..))).)......))))))))))))).))))))))).))))).).))))))))..
...................UGAAGCUGCCAGCAUGAUCUGA............................................................................. 190321.638405
..................CUGAAGCUGCCAGCAUGAUCUGA............................................................................. 120.675966
..................CUGAAGCUGCCAGCAUGAUCUGG............................................................................. 151.729718
..................CUGAAGCUGCCAGCAUGAUCUA.............................................................................. 136.909485
...................UGAAGCUGCCAGCAUGAUCUG.............................................................................. 87373.08142
...................UGAAGCUGCCAGCAUGAUCU............................................................................... 10812.468169
...................UGAAGCUGCCAGCAUGAUCUGAG............................................................................ 93.456948
...................UGAAGCUGCCAGCAUGAUC................................................................................ 7600.3824
...................UGAAGCUGCCAGCACGAUCUG.............................................................................. 57.250546
...................UGAAGCUGCCAGCAUUAUCUG.............................................................................. 54.428535
...................UGCAGCUGCCAGCAUGAUCUGA............................................................................. 191.23146
...................UGAAGCUGCCAGCCUGAUCU............................................................................... 56.97454
...................UGAAGCUGCCAGUAUGAUCUG.............................................................................. 46.20878
...................UGAAGCUGCCUGCAUGAUCUGA............................................................................. 65.733528
...................UGAAGCUGCCAGCAUGCUCUGA............................................................................. 329.205711
...................UGAAGCUCCCAGCAUGAUCUGA............................................................................. 72.558829
...................UUAAGCUGCCAGCAUGAUCUGA............................................................................. 139.981775
...................UGAAGCUGCCAGCAUUAUCUGA............................................................................. 106.403597
...................UGAAGCUGCUAGCAUGAUCUGA............................................................................. 217.573975
...................UAAAGCUGCCAGCAUGAUCUGA............................................................................. 101.522412
...................UGAAGCUGCCAGCAUGAUCUGU............................................................................. 2498.179587
...................UGAAGCUGCCAGCAUGAUCUGC............................................................................. 1106.906571
...................UGAAGCUGCCAGCAUGAUUU............................................................................... 303.296332
...................UGAAGCUGCCCGCAUGAUCUG.............................................................................. 90.249355
...................UGAAGCUGACAGCAUGAUCUGA............................................................................. 103.380295
...................UGACGCUGCCAGCAUGAUCUGA............................................................................. 90.181403
...................UGAACCUGCCAGCAUGAUCUGA............................................................................. 59.794643
...................UGAAGCUGCCAGCAUGGUCUG.............................................................................. 93.724288
...................UGAAGCUGCCAGCAUGGUCUGA............................................................................. 281.801836
...................UGAAGCUGCCAUCAUGAUCUGA............................................................................. 81.12687
...................UGAAGCUGCCAGCAUGAUCCG.............................................................................. 377.87098
...................UGAAGCUUCCAGCAUGAUCUGA............................................................................. 121.400555
...................UGAAACUGCCAGCAUGAUCUGA............................................................................. 55.567834
...................UGAAGCUGCCAGCAUGAUCGG.............................................................................. 221.064558
...................UGAAGCUACCAGCAUGAUCUGA............................................................................. 93.822657
...................UGAAGCUGCCAGCAUGAUCUGGG............................................................................ 58.964747
...................UGAAGCUGCCAGAAUGAUCUGA............................................................................. 50.958286
...................UGAAGCUGCCAGCAUGAUUUGA............................................................................. 149.630725
...................UGAAGCUGCCAGCAUGUUCUGA............................................................................. 40.698538
...................UGAAGAUGCCAGCAUGAUCUGA............................................................................. 85.782049
...................UGAAGCUGCCAGCAUGAUCUUA............................................................................. 84.496084
...................UGAAGCUGCCAGCAUGACC................................................................................ 89.30017
...................GGAAGCUGCCAGCAUGAUCUGA............................................................................. 68.19713
...................UGAAGCUGACAGCAUGAUCUG.............................................................................. 42.117292
...................UGAAGCUGCCAGCAUAAUCUGA............................................................................. 93.573188
...................UGAAGCUGCCGGCAUGAUCUG.............................................................................. 70.24741
...................UGAAGCUGCCCGCAUGAUCUGA............................................................................. 180.57913
...................UGAAGCUGCUAGCAUGAUCUG.............................................................................. 319.235784
...................UGAAGCUGCCAGCAUGAUCUU.............................................................................. 1312.566292
...................UGAAGCUGUCAGCAUGAUCUGA............................................................................. 222.347364
...................UGAAGCUGCCAACAUGAUCUGA............................................................................. 69.907627
...................UGAAGCUGCCAGCACGAUCUGA............................................................................. 192.4512
...................UGAAGCUGCCAGCAUGCUCUG.............................................................................. 170.22137
...................UGAAGCUGCCAGCAUGAUCUCA............................................................................. 51.863964
...................UGAAUCUGCCAGCAUGAUCUGA............................................................................. 166.209286
...................UGAAGCUGCCAGCGUGAUCUG.............................................................................. 57.90805
...................UGAAGCUGCCGGCAUGAUCUGA............................................................................. 249.39207
...................UGAAGCAGCCAGCAUGAUCUGA............................................................................. 56.747034
...................UGAAGCUGUCAGCAUGAUCUG.............................................................................. 262.231569
...................UGAAGCUGCCAUCAUGAUCUG.............................................................................. 84.182363
...................UGAAGCUGCCAGCCUGAUC................................................................................ 268.02408
...................CGAAGCUGCCAGCAUGAUCUGA............................................................................. 261.08718
...................UGGAGCUGCCAGCAUGAUCUGA............................................................................. 261.262175
...................UGAAGCUGCAAGCAUGAUCUGA............................................................................. 146.964335
...................UGAGGCUGCCAGCAUGAUCUGA............................................................................. 285.387524
...................UGAAGCUGCCAGCAUGAUCC............................................................................... 701.157019
...................UGAAGCUGCCAGCAUGAUCA............................................................................... 410.926106
...................UGAAGCUGCCAGCAUGAUCG............................................................................... 140.794267
...................UGAAGCUGCAAGCAUGAUCUG.............................................................................. 66.905988
...................UGAAGCUGCCAGCAUGAUAUGA............................................................................. 194.530758
...................UGAAGUUGCCAGCAUGAUCUGA............................................................................. 179.483606
...................UGAAGCUGCCAGCAUGACCUGA............................................................................. 193.93929
...................UGAAGCUGCCAGCAUGAUCUGAC............................................................................ 183.785896
...................UGAAGCUGCCAGCCUGAUCUG.............................................................................. 378.91246
...................UGAAGCUGCCAGUAUGAUCUGA............................................................................. 130.096887
...................UGAAGCUGCCAGCCUGAUCUGA............................................................................. 926.975749
...................UGAAGCUGCCAGCAUGAUAUG.............................................................................. 69.665306
...................UGAAGCUGCCAGCGUGAUCUGA............................................................................. 214.150885
...................UGAAGCUGCCAGCAUGAUCGGA............................................................................. 121.190122
...................UGAAGCUGCCAGCAUGACCUG.............................................................................. 82.41033
...................UGAAGCUGCCAGCAUGAUCUC.............................................................................. 366.816998
...................UGAAGCUGCCAGCAUGAUCAGA............................................................................. 54.066961
...................AGAAGCUGCCAGCAUGAUCUGA............................................................................. 76.736765
...................UGAAGCUGCCAGCAUGAUCCGA............................................................................. 209.23258
...................UGAAGCCGCCAGCAUGAUCUGA............................................................................. 245.45403
...................UGAAGCGGCCAGCAUGAUCUGA............................................................................. 48.117644
...................UGAAGCUGCCAGCAUGAUA................................................................................ 100.890336
...................UGAAGCUGCCAGCAUGAUU................................................................................ 496.456597
...................UGAAGCUGCCAGCAUGAUCAG.............................................................................. 48.591311
...................UGAAGCUGCCAGCAUGAUGUGA............................................................................. 104.729463
....................GAAGCUGCCAGCAUGAUCUG.............................................................................. 118.106332
....................GAAGCUGCCAGCAUGAUCUGA............................................................................. 310.842898
.....................AAGCUGCCAGCAUGAUCUGAG............................................................................ 48.844389
.....................AAGCUGCCAGCAUGAUCUGA............................................................................. 586.619841
...............................................................................GAUCAUGCUGUGCAGUUUCAU.................. 41.398435
...............................................................................GAUCAUGCUGUGCAGUUUCAUC................. 1173.246897
................................................................................AUCAUGCUGUGCAGUUUCAUC................. 106.503777
```

zma-MIR167f

```
Chromosome:    chr3
Start:         195044722
End:           195044834
Strand:        +
Strand bias:   1.0
Abundance bias:0.997
MFE:           -52.70
```

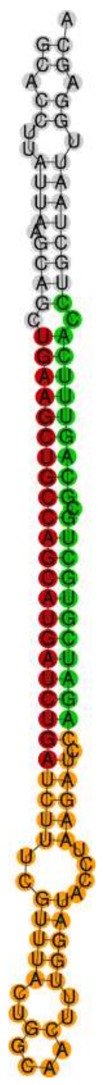

```
                Mature                                             Star
GCACCUUAUUAAGCAGCUGAAGCUGCCAGCAUGAUCUGAUCUUUCGUUUACUGGCAACUUUGGAUACCUAAGAUCCAGAUCGUGCUGCGCAGUUUCACCUGCUAAUUGGAGCA   317718.2
((.((..((((.((((.((((((((((((((((((((((((((..(((((..(....)..)))))....))))).)))))))))))).))))))))).)))))))).)).)).
.................UGAAGCUGCCAGCAUGAUCUGA..........................................................................   190321.638405
................CUGAAGCUGCCAGCAUGAUCUGA..........................................................................   120.675966
................CUGAAGCUGCCAGCAUGAUCUGG..........................................................................   151.729718
................CUGAAGCUGCCAGCAUGAUCUA...........................................................................   136.909485
.................UGAAGCUGCCAGCAUGAUCUGAU.........................................................................   202.181081
.................UGAAGCUGCCAGCAUGAUCUG...........................................................................   87373.08142
.................UGAAGCUGCCAGCAUGAUCU............................................................................   10812.468169
.................UGAAGCUGCCAGCAUGAUC.............................................................................   7600.3824
.................UGAAGCUGCCAGCACGAUCUG...........................................................................   57.250546
.................UGAAGCUGCCAGCAUUAUCUG...........................................................................   54.428535
.................UGCAGCUGCCAGCAUGAUCUGA..........................................................................   191.23146
.................UGAAGCUGCCAGCCUGAUCU............................................................................   56.97454
.................UGAAGCUGCCAGUAUGAUCUG...........................................................................   46.20878
.................UGAAGCUGCCUGCAUGAUCUGA..........................................................................   65.733528
.................UGAAGCUGCCAGCAUGCUCUGA..........................................................................   329.205711
.................UGAAGCUCCCAGCAUGAUCUGA..........................................................................   72.558829
.................UUAAGCUGCCAGCAUGAUCUGA..........................................................................   139.981775
.................UGAAGCUGCCAGCAUUAUCUGA..........................................................................   106.403597
.................UGAAGCUGCUAGCAUGAUCUGA..........................................................................   217.573975
.................UAAAGCUGCCAGCAUGAUCUGA..........................................................................   101.522412
.................UGAAGCUGCCAGCAUGAUCUGU..........................................................................   2498.179587
.................UGAAGCUGCCAGCAUGAUCUGC..........................................................................   1106.906571
.................UGAAGCUGCCAGCAUGAUUU............................................................................   303.296332
.................UGAAGCUGCCCGCAUGAUCUG...........................................................................   90.249355
.................UGAAGCUGACAGCAUGAUCUGA..........................................................................   103.380295
.................UGACGCUGCCAGCAUGAUCUGA..........................................................................   90.181403
.................UGAACCUGCCAGCAUGAUCUGA..........................................................................   59.794643
.................UGAAGCUGCCAGCAUGGUCUG...........................................................................   93.724288
.................UGAAGCUGCCAGCAUGGUCUGA..........................................................................   281.801836
.................UGAAGCUGCCAUCAUGAUCUGA..........................................................................   81.12687
.................UGAAGCUGCCAGCAUGAUCUGUU........................................................................   115.571651
.................UGAAGCUGCCAGCAUGAUCCG...........................................................................   377.87098
.................UGAAGCUGCCAGAAUGAUCUG...........................................................................   37.362435
.................UGAAGCUUCCAGCAUGAUCUGA..........................................................................   121.400555
.................UGUAGCUGCCAGCAUGAUCUGA..........................................................................   40.438589
.................UGAAACUGCCAGCAUGAUCUGA..........................................................................   55.567834
.................UGAAGCUGCCAGCAUGAUCGG...........................................................................   221.064558
.................UGAAGCUACCAGCAUGAUCUGA..........................................................................   93.822657
.................UGAAGCUGCCAGAAUGAUCUGA..........................................................................   50.958286
.................UGAAGCUGCCAGCAUGAUUUGA..........................................................................   149.630725
.................UGAAGCUGCCAGCAUGUUCUGA..........................................................................   40.698538
.................UGAAGAUGCCAGCAUGAUCUGA..........................................................................   85.782049
.................UGAAGCUGCCAGCAUGAUCUUA..........................................................................   84.496084
.................UGAAGCUGCCAGCAUGACC.............................................................................   89.30017
.................GGAAGCUGCCAGCAUGAUCUGA..........................................................................   68.19713
.................UGAAGCUGACAGCAUGAUCUG...........................................................................   42.117292
.................UGAAGCUGCCAGCAUAAUCUGA..........................................................................   93.573188
.................UGAAGCUGCCGGCAUGAUCUG...........................................................................   70.24741
.................UGAAGCUGCCCGCAUGAUCUGA..........................................................................   180.57913
.................UGAAGCUGCUAGCAUGAUCUG...........................................................................   319.235784
.................UGAAGCUGCCAGCAUGAUCUU...........................................................................   1312.566292
.................UGAAGCUGUCAGCAUGAUCUGA..........................................................................   222.347364
.................UGAAGCUGCCAACAUGAUCUGA..........................................................................   69.907627
.................UGAAGCUGCCAGCACGAUCUGA..........................................................................   192.4512
.................UGAAGCUGCCAGCAUGCUCUG...........................................................................   170.22137
.................UGAAGCUGCCAGCAUGAUCUCA..........................................................................   51.863964
.................UGAAUCUGCCAGCAUGAUCUGA..........................................................................   166.209286
.................UGAAGCUGCCAGCGUGAUCUG...........................................................................   57.90805
.................UGAAGCUGCCGGCAUGAUCUGA..........................................................................   249.39207
.................UGAAGCAGCCAGCAUGAUCUGA..........................................................................   56.747034
.................UGAAGCUGUCAGCAUGAUCUG...........................................................................   262.231569
.................UGAAGCUGCCAUCAUGAUCUG...........................................................................   84.182363
.................UGAAGCUGCCAGCCUGAUC.............................................................................   268.02408
.................CGAAGCUGCCAGCAUGAUCUGA..........................................................................   261.08718
.................UGGAGCUGCCAGCAUGAUCUGA..........................................................................   261.262175
.................UGAAGCUGCAAGCAUGAUCUGA..........................................................................   146.964335
.................UGAGGCUGCCAGCAUGAUCUGA..........................................................................   285.387524
.................UGAAGCUGCCAGCAUGAUCC............................................................................   701.157019
.................UGAAGCUGCCAGCAUGAUCA............................................................................   410.926106
.................UGAAGCUGCCAGCAUGAUCG............................................................................   140.794267
.................UGAAGCUGCAAGCAUGAUCUG...........................................................................   66.905988
.................UGAAGCUGCCAGCAUGAUAUGA..........................................................................   194.530758
.................UGAAGUUGCCAGCAUGAUCUGA..........................................................................   179.483606
.................UGAAGCUGCCAGCAUGACCUGA..........................................................................   193.93929
.................UGAAGCUGCCAGCAUGAUCUGAC.........................................................................   183.785896
.................UGAAGCUGCCAGCCUGAUCUG...........................................................................   378.91246
.................UGAAGCUGCCAGUAUGAUCUGA..........................................................................   130.096887
.................UGAAGCUGCCAGCCUGAUCUGA..........................................................................   926.975749
.................UGAAGCUGCCAGCAUGAUAUG...........................................................................   69.665306
.................UGAAGCUGCCAGCAUGAUCUGUUC........................................................................   38.32553
.................UGAAGCUGCCAGCGUGAUCUGA..........................................................................   214.150885
.................UGAAGCUGCCAGCAUGAUCGGA..........................................................................   121.190122
.................UGAAGCUGCCAGCAUGACCUG...........................................................................   82.41033
.................UGAAGCUGCCAGCAUGAUCUC...........................................................................   366.816998
.................UGAAGCUGCCAGCAUGAUCAGA..........................................................................   54.066961
.................AGAAGCUGCCAGCAUGAUCUGA..........................................................................   76.736765
.................UGAAGCUGCCAGCAUGAUCCGA..........................................................................   209.23258
.................UGAAGCCGCCAGCAUGAUCUGA..........................................................................   245.45403
.................UGAAGCGGCCAGCAUGAUCUGA..........................................................................   48.117644
.................UGAAGCUGCCAGCAUGAUA.............................................................................   100.890336
.................UGAAGCUGCCAGCAUGAUU.............................................................................   496.456597
.................UGAAGCUGCCAGCAUGAUCAG...........................................................................   48.591311
.................UGAAGCUGCCAGCAUGAUGUGA..........................................................................   104.729463
..................GAAGCUGCCAGCAUGAUCUG...........................................................................   118.106332
..................GAAGCUGCCAGCAUGAUCUGA..........................................................................   310.842898
...................AAGCUGCCAGCAUGAUCUGA..........................................................................   586.619841
.............................................................................GAUCGUGCUGCGCAGUUUCACC..............   133.839045
```

zma-MIR169f

Chromosome: chr5
Start: 168617413
End: 168617609
Strand: +
Strand bias: 1.0
Abundance bias: 0.974
MFE: -103.00

```
                                          Mature                                                                                       Star
GAGAGGAGGGCCUUGCAUGAGGACCAGAGCUGAUUCGUUCAGUAGCCAAGGAUGACUUGCCUAGGUAUAUAUGCAUGGGCUAUGGCUACAUGCCUGAGAGCCAGUCUCUUGUGACGCUGAGCAUGUAUAGUGUAGGCAUGUCUUCCUUGGCUACUCGGAGCGGCUCUAGUCAUCCAUGGAAGGCCUCAAUCUUCUUU   3277.8
(((((((((((((.((((..(((.(((((((.((((...(((((((((((.(((.((((((.((((((((((...((((..(((.....)))....)))).(((......))).....))))))))).).)))))).))).))))))))))))))))).))))))).)))...)))).)))))))...))))))).
..........................................UAGCCAAGGAUGACUUGCCUA......................................................................................................................................   1737.40401
..........................................UAGCCAAGGAUGACUUGCCU.......................................................................................................................................   1202.958549
..........................................UAGCCAAGGAUGACUUGC.........................................................................................................................................   50.854203
..........................................UAGCCAAGGAUGACUUGCC........................................................................................................................................   17.032323
..........................................UAGCCAAGGAUGACUUGUCUA......................................................................................................................................   1.5598
..........................................UAGCCAAGGAUGACUUGCCA.......................................................................................................................................   2.633131
..........................................UAGCCAAGGAUGACUCGCCUA......................................................................................................................................   2.59967
..........................................CAGCCAAGGAUGACUUGCCUA......................................................................................................................................   15.598
..........................................UAGCCAAGGAUGACUUGCCUU......................................................................................................................................   50.494015
..........................................UAGCCAAGGAUGACUUGCCUC......................................................................................................................................   6.51412
..........................................UAGCCAAGGAUGACUUGU.........................................................................................................................................   5.4679
..........................................UAGCCACGGAUGACUUGCCUA......................................................................................................................................   1.81977
..........................................UAGCCAAGGAUGAUUUGCCUA......................................................................................................................................   2.85964
..........................................UAGCCAAGGAUGAAUUGCCUA......................................................................................................................................   1.81977
..........................................UAGCCAAGGAUGACCUGCCUA......................................................................................................................................   3.37957
..........................................UAUCCAAGGAUGACUUGCCUA......................................................................................................................................   3.89951
..........................................UAGCUAAGGAUGACUUGCCUA......................................................................................................................................   2.07974
..........................................UAGCCAAGGAUGACUUGACUA......................................................................................................................................   2.3397
..........................................UAGCCAAGGAUCACUUGCCUA......................................................................................................................................   2.07974
..........................................UAGCCAAGGAUGACUUGCAUA......................................................................................................................................   2.3397
..........................................UAGCCAAGGAUGACUGGCCUA......................................................................................................................................   1.5598
..........................................UAGCCAAGGAUGACUUGCCGA......................................................................................................................................   18.729533
..........................................UAGCCAAGGAUUACUUGCCUA......................................................................................................................................   1.5598
..........................................UAGCCAAGGAUGACUUUCCUA......................................................................................................................................   5.19934
..........................................UAGCCAAGGAGGACUUGCCUA......................................................................................................................................   2.3397
..........................................UAGCCAAGGAUGACGUGCCUA......................................................................................................................................   2.07974
..........................................UAGCCAAGGAUGCCUUGCCUA......................................................................................................................................   3.096958
..........................................UAGACAAGGAUGACUUGCCUA......................................................................................................................................   1.81977
..........................................UAGCCAAGGAUGACUUGCU........................................................................................................................................   8.984663
..........................................UAGCAAAGGAUGACUUGCCUA......................................................................................................................................   2.59967
..........................................UAGCCAAGGAUGACUUCCCUA......................................................................................................................................   2.3397
...........................................AGCCAAGGAUGACUUGCCU.......................................................................................................................................   25.069596
............................................GCCAAGGAUGACUUGCCU.......................................................................................................................................   2.047482
```

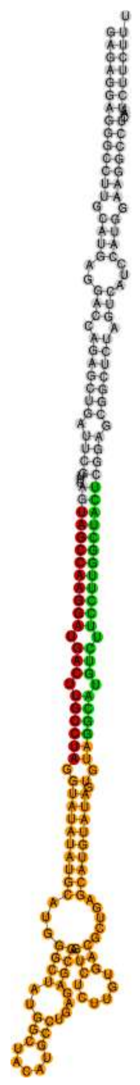

zma-MIR169l

Chromosome: chr1
Start: 303772009
End: 303772126
Strand: +
Strand bias: 1.0
Abundance bias: 0.981
MFE: -62.60

```
                         Mature                                                           Star
UAAGAGGCCAUCUUUGGUAGCCAGGGAUGAUUUGCCUGUGGUUCCUGUCGUUGCCAUGCCAAUGCCUGUACUGUUCGAUCUACGGGCAAAUCAUCCCUGCUACCGGAGUGUCUCUUAC    242.3
((((((((...(((((((((.((((((((((((((((((((.....((((..((..(((........)))..)).))))))))))))))))))))))))))))))))).)))))))).
.................UAGCCAGGGAUGAUUUGCCUGU...............................................................................    225.363825
.................UAGCCAGGGAUGAUUUGCC..................................................................................    1.22768
.................UAGCCAGGGAUGAUUUGCCUGUU..............................................................................    2.3397
.................UAGCCAGGGAUGAUUUUCCUGU...............................................................................    1.81977
.................UAGCAAGGGAUGAUUUGCCUGU...............................................................................    1.5598
..................AGCCAGGGAUGAUUUGCCUGU...............................................................................    5.45931
```

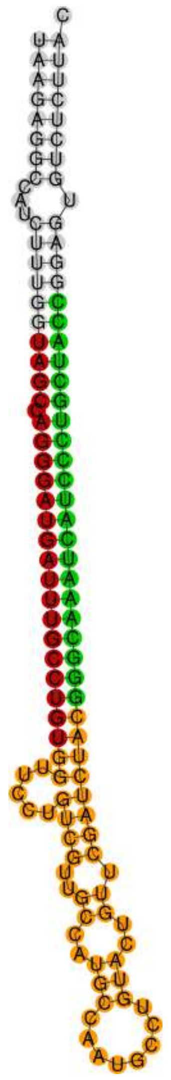

zma-MIR169p

```
Chromosome:    chr7
Start:         114375713
End:           114375808
Strand:        -
Strand bias:   1.0
Abundance bias:1.0
MFE:           -55.30
```

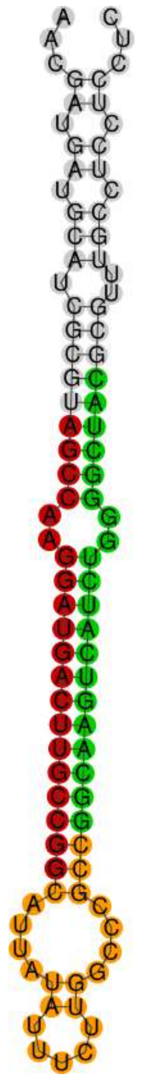

```
                      Mature                                      Star
AACGAUGAUGCAUCGCGUAGCCAAGGAUGACUUGCCGGCAUUAUAUUUCUUGGCCCGCCGGCAAGUCAUCUGGGGCUACGCGUUUGCCUCCUCCUC    5658.8
...((.((.(((.(((((((((..((((((((((((((....((.....))....))))))))))))))..)))))))))..))).)).))...
..................AGCCAAGGAUGACUUGCCGG..........................................................    3626.68943
.................UAGCCAAGGAUGACUUGCCG...........................................................    15.70003
.................UAGCCAAGGAUGACUUGC.............................................................    50.854203
.................UAGCCAAGGAUGACUUGCCGGC.........................................................    12.99016
.................UAGCCAAGGAUGACUUGCCGG..........................................................    1446.500292
.................UAGCCAAGGAUGACUUGCC............................................................    17.032323
.................UAGCCAAGGAUGACUUGCCA...........................................................    2.633131
.................UAGCCAAGGAUGACUUGU.............................................................    5.4679
.................UAGCCAAGGAUGACUUGUCGG..........................................................    1.51294
.................UAGCCAAGGAUGACUUGCCGA..........................................................    18.729533
.................UAGCCAAGGAUGAAUUGCCGG..........................................................    1.51294
.................UAGCCAAGGAUGACUUUCCGG..........................................................    1.94521
.................UAGCCAAGGAUGACUUGCU............................................................    8.984663
..................AGCCAAGGAUGACUUGCCGGC.........................................................    182.467562
..................AGCCAAGGAUGACUUGCCG...........................................................    73.961898
..................AGCCAAGGAUGACUUGCCGGU.........................................................    0.552991
..................AGCCAAGGAUGCCUUGCCGG..........................................................    1.81917
..................AGCCAAGGAUGACUUGCUGG..........................................................    0.627811
..................AGCCAAAGAUGACUUGCCGG..........................................................    0.627811
..................AGCCAGGGAUGACUUGCCGG..........................................................    0.737322
..................AGCCAAGGAUGACUUGCCGU..........................................................    1.5598
..................AGCCAAGGGUGACUUGCCGG..........................................................    0.61377
..................GGCCAAGGAUGACUUGCCGG..........................................................    1.460683
..................AGCCAAGGAUGAUUUGCCGG..........................................................    0.607944
...................GCCAAGGAUGACUUGCCGG..........................................................    76.694391
...................GCCAAGGAUGACUUGCCG...........................................................    3.39119
....................CCAAGGAUGACUUGCCGG..........................................................    95.567775
..........................................................CGGCAAGUCAUCUGGGGCUAC.................    7.61893
```

zma-MIR169q

Chromosome: chr8
Start: 130375268
End: 130375425
Strand: +
Strand bias: 1.0
Abundance bias: 1.0
MFE: -73.20

Mature Star

```
AUGAGGAGCCUAUCUCCCUUAGCCAAGAAUGGCUUGCCUAUCUCCAUUAUUUGGUCCAUCUUCUUGGGAGACCUCCCUCAUGGUGUGGAUGGAAUGUGGAUGGUGGCAGGCCUUCUGGCUAAGUGAGUGAUUCUCUUCCGCAAUGCUAGGCUGCUUGC    3605.2
..((((((..(..(((.((((((((.(((.((((((((.((((((((.((((.((((((((....((((....))))....)).)))))).))))))))).)))))))))))))))))))))))).)))..)..)))))).((((.((......))))))
.................CUUAGCCAAGAAUGGCUUGCCU.......................................................................................................................    3323.761894
................CCUUAGCCAAGAAUGGCUUGCCU.......................................................................................................................    0.477722
.................CUUAGCCAAGAAUGGCUUGCC........................................................................................................................    110.331863
.................CUUAGCCAAGAAUGGCUUGCCA.......................................................................................................................    88.001851
.................CCUAGCCAAGAAUGGCUUGCCU.......................................................................................................................    2.61104
.................CUUAGCCAAGAAUGGCUUGCCC.......................................................................................................................    2.74391
.................CUUAGCCAAGAAUGGCUUGCCG.......................................................................................................................    13.897776
.................CUUAGCCAAGAAUGGCUUUCCU.......................................................................................................................    16.852706
.................CUGAGCCAAGAAUGGCUUGCCU.......................................................................................................................    2.31465
.................CUUAGCCAAGAAUGGCUUGGCU.......................................................................................................................    1.0616
..................UUAGCCAAGAAUGGCUUGCCUA......................................................................................................................    2.519999
..................UUAGCCAAGAAUGGCUUGCCU.......................................................................................................................    19.976499
..................UUAGCCAAGAAUGACUUGCCU.......................................................................................................................    3.20072
...................UAGCCAAGAAUGGCUUGCCUAUC....................................................................................................................    17.31172
```

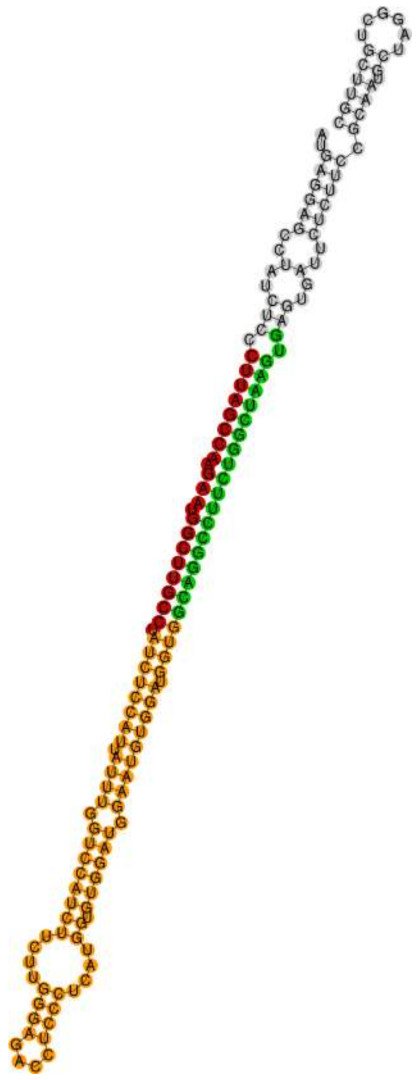

zma-MIR171b

Chromosome: chr10
Start: 143745123
End: 143745232
Strand: -
Strand bias: 1.0
Abundance bias: 1.0
MFE: -53.80

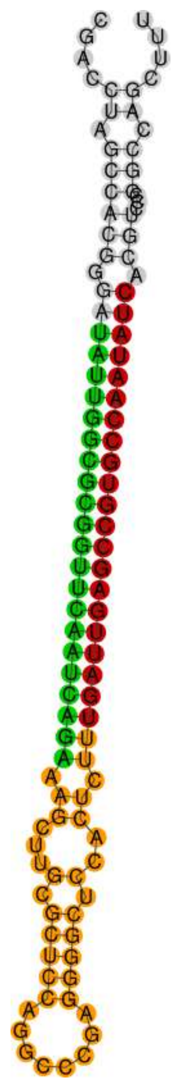

Star Mature

```
CGACCUAGCCACGGGAUAUUGGCGCGGUUCAAUCAGAAAGCUUGCGCUCCAGGCCCGAGGGGCUCCACUCUUUGAUUGAGCCGUGCCAAUAUCACGUCCCGGCCAGCUUU    146564.9
....((.((((((.(((((((((((((((((((((((.((...(.(((((........))))).)..)).))))))))))))))))))))))).)))...))).))....
........................................................................UGAUUGAGCCGUGCCAAUAUC.................    137861.56804
...........................................................................UUGAGCCGUGCCAAUAUCACG..............    525.45142
...........................................................................UUGAGCCGUGCCAAUAUCAC...............    7.81576
...........................................................................UUGAGCCGUGCCAAUAUC.................    47.74514
..........................................................................AUUGAGCCGUGCCAAUAUC.................    93.864466
.........................................................................GAUUGAGCCGUGCCAAUA...................    3.85758
.........................................................................GAUUGAGCCGUGCCAAUAUC.................    2898.392626
.........................................................................GAUUGAGCCGUGCCAAUAU..................    52.2702
.........................................................................GAUUGAGCCGUGCUAAUAUC.................    2.50743
.........................................................................GAUUGAGCCGUGUCAAUAUC.................    5.59349
.........................................................................GAUUGAGCCCUGCCAAUAUC.................    5.40062
.........................................................................GAUUGAGCCGUACCAAUAUC.................    2.50743
.........................................................................GAUUGAGCCGUGCCUAUAUC.................    3.27895
.........................................................................GAUUGAGUCGUGCCAAUAUC.................    5.78637
.........................................................................GAUUGAGCCGUGCCCAUAUC.................    3.47182
.........................................................................GAUUGAGCCUUGCCAAUAUC.................    4.24334
.........................................................................GAUUGAGCCGUGCCAAUAUU.................    4.82198
.........................................................................GAUUGAGCCGUGCCAAAAUC.................    3.85758
.........................................................................GAUUGAGCCAUGCCAAUAUC.................    7.90804
.........................................................................GAUUGAGCCGUGCCAACAUC.................    3.85758
.........................................................................GAUUGAGCCGUGCCAGUAUC.................    2.50743
.........................................................................GAUUGAGCUGUGCCAAUAUC.................    8.48668
.........................................................................GAUUGAGCCGUGCCGAUAUC.................    5.01486
.........................................................................GAUUGAGCCGUGCCAAUACC.................    2.70031
........................................................................UGAUUGAGCCGUGCCAAUAU..................    519.460044
........................................................................UGAUUGAGCCGUGCCAAU....................    80.757837
........................................................................UGAUUGAGCCGUGCCAAUAUCA................    131.412047
........................................................................UGAUUGAGCCGUGCCAAUA...................    253.099266
........................................................................UGAUUGAGCCGUGCCAGUAUC.................    52.269209
........................................................................UGAUUGCGCCGUGCCAAUAUC.................    45.54109
........................................................................UGAUUGAGCCGGGCCAAUAUC.................    104.768169
........................................................................UGAUUGAGCCGUGCUAAUAUC.................    61.711192
........................................................................UGAUGGAGCCGUGCCAAUAUC.................    16.57303
........................................................................UGAUUGAGCCGAGCCAAUAUC.................    32.82447
........................................................................UGAUUGAGGCGUGCCAAUAUC.................    19.10187
........................................................................UGAUUGAGCCGUGCCGAUAUC.................    56.489393
........................................................................UGAGUGAGCCGUGCCAAUAUC.................    43.316798
........................................................................UGAUUGAGCCGUGCGAAUAUC.................    7.43492
........................................................................UGAUUGAGCAGUGCCAAUAUC.................    76.172984
........................................................................UGAUUGAGCCGUGUCAAUAUC.................    97.237935
........................................................................UGAUUGAGCCGUACCAAUAUC.................    54.340798
........................................................................UGAUUGAGCCGUUCCAAUAUC.................    111.306088
........................................................................UGAUUGAUCCGUGCCAAUAUC.................    67.193462
........................................................................UGAUUGAGCCGUGCCAAUUU..................    32.03288
........................................................................UGAUUGAGCCGUGCCUAUAUC.................    24.070366
........................................................................UGAUUGAGCCGUGCCAAUUUC.................    29.55859
........................................................................UUAUUGAGCCGUGCCAAUAUC.................    111.300891
........................................................................UGAUUGAGCCGUGCCAAUAUG.................    22.035681
........................................................................UGAUUGAGCCCUGCCAAUAUC.................    30.08748
........................................................................UGAUUGAGUCGUGCCAAUAUC.................    178.071637
........................................................................UGAUUGAGCCGUGCCAAUACC.................    100.805743
........................................................................UGAUUGAGCCGUGGCAAUAUC.................    19.08152
........................................................................UGAUUGAGCCGUGCCAAUAUU.................    352.612951
........................................................................UGAUUGAGCCGUGCCAAUAUA.................    157.194939
........................................................................UGAUUGAGCCGUCCCAAUAUC.................    40.289629
........................................................................CGAUUGAGCCGUGCCAAUAUC.................    113.740769
........................................................................UGAUUGAGCCGUGCCAAGAUC.................    18.12679
........................................................................UGAUAGAGCCGUGCCAAUAUC.................    9.49441
........................................................................UGAUUGAGCCGUGCCAAUAUCG................    9.82041
........................................................................UGAUUGAGCCGUGCCAAUAUCC................    131.39027
........................................................................UGCUUGAGCCGUGCCAAUAUC.................    103.135059
........................................................................UGAUUGAGCGGUGCCAAUAUC.................    15.51776
........................................................................UGAUUGAACCGUGCCAAUAUC.................    50.866178
........................................................................AGAUUGAGCCGUGCCAAUAUC.................    32.918334
........................................................................GGAUUGAGCCGUGCCAAUAUC.................    126.493245
........................................................................UGAUUAAGCCGUGCCAAUAUC.................    25.060514
........................................................................UCAUUGAGCCGUGCCAAUAUC.................    18.4335
........................................................................UGAUUGAGCCGUGCCAACAUC.................    42.022085
........................................................................UGAUUGAGCUGUGCCAAUAUC.................    96.569206
........................................................................UGAUUGAGCCGUGCCAAUGUC.................    40.229137
........................................................................UGAUUGAGCCUUGCCAAUAUC.................    103.005721
........................................................................UGAUUGAGCCGUGCCAAAAUC.................    3.67141
........................................................................UGAUUGAGCCGUGCCAAUCUC.................    36.758104
........................................................................UGAUCGAGCCGUGCCAAUAUC.................    52.909318
........................................................................UGAUUGACCCGUGCCAAUAUC.................    65.044061
........................................................................UGAUUGAGCCGUGCCAAUAGC.................    34.9994
........................................................................UAAUUGAGCCGUGCCAAUAUC.................    30.047405
........................................................................UGAUUGAGCCGUGACAAUAUC.................    91.7386
........................................................................UGAAUGAGCCGUGCCAAUAUC.................    6.924
........................................................................UGAUUGAGCCGUGCCAAUC...................    19.98699
........................................................................UGAUUGAGCCGUGCAAAUAUC.................    53.1025
........................................................................UGAUUGAGACGUGCCAAUAUC.................    64.913135
........................................................................UGAUUGAGCCAUGCCAAUAUC.................    62.387571
........................................................................UGAUUGUGCCGUGCCAAUAUC.................    5.16365
........................................................................UGGUUGAGCCGUGCCAAUAUC.................    53.74719
........................................................................UGAUUGAGCCGUGCCAAUACCA................    22.06487
........................................................................UGAUUGGGCCGUGCCAAUAUC.................    79.264387
........................................................................UGAUUUAGCCGUGCCAAUAUC.................    75.421776
........................................................................UGAUUGAGCCGUGCCACUAUC.................    89.54748
........................................................................UGAUUCAGCCGUGCCAAUAUC.................    64.859455
........................................................................UGAUUGAGCCGUGCCCAUAUC.................    77.67629
........................................................................UGUUUGAGCCGUGCCAAUAUC.................    7.15019
........................................................................UGAUUGAGCCGUGCCAAUAAC.................    17.84542
........................................................................UGAUUGAGCCGUGCCAUUAUC.................    39.01943
.......................................................................UUGAUUGAGCCGUGCCAAUAUC.................    17.09109
................UAUUGGCGAGGUUCAAUCAGA.........................................................................    4.677886
.............GGAUAUUGGCGCGGUUCAAUC............................................................................    18.330614
```

zma-MIR171b1

```
Chromosome:    chr5
Start:         223360727
End:           223360836
Strand:        -
Strand bias:   1.0
Abundance bias:1.0
MFE:           -53.80
```

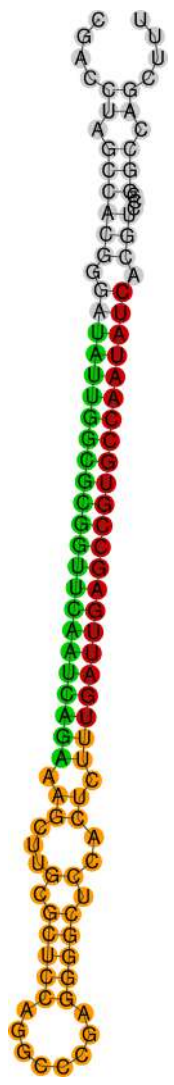

```
                Star                                                  Mature
CGACCUAGCCACGGGAUAUUGGCGCGGUUCAAUCAGAAAGCUUGCGCUCCAGGCCCGAGGGGCUCCACUCUUUGAUUGAGCCGUGCCAAUAUCACGUCCCGGCCAGCUUU    146564.9
....((.((((((.(((((((((((((((((((((((.((...(.(((((........))))).)..)).))))))))))))))))))))))).))).)))...))).))....
........................................................................UGAUUGAGCCGUGCCAAUAUC.................    137861.56804
...........................................................................UUGAGCCGUGCCAAUAUCACG..............    525.45142
...........................................................................UUGAGCCGUGCCAAUAUCAC...............    7.81576
...........................................................................UUGAGCCGUGCCAAUAUC.................    47.74514
..........................................................................AUUGAGCCGUGCCAAUAUC.................    93.864466
.........................................................................GAUUGAGCCGUGCCAAUA...................    3.85758
.........................................................................GAUUGAGCCGUGCCAAUAUC.................    2898.392626
.........................................................................GAUUGAGCCGUGCCAAUAU..................    52.2702
.........................................................................GAUUGAGCCGUGCUAAUAUC.................    2.50743
.........................................................................GAUUGAGCCGUGUCAAUAUC.................    5.59349
.........................................................................GAUUGAGCCCUGCCAAUAUC.................    5.40062
.........................................................................GAUUGAGCCGUACCAAUAUC.................    2.50743
.........................................................................GAUUGAGCCGUGCCUAUAUC.................    3.27895
.........................................................................GAUUGAGUCGUGCCAAUAUC.................    5.78637
.........................................................................GAUUGAGCCGUGCCCAUAUC.................    3.47182
.........................................................................GAUUGAGCCUUGCCAAUAUC.................    4.24334
.........................................................................GAUUGAGCCGUGCCAAUAUU.................    4.82198
.........................................................................GAUUGAGCCGUGCCAAAAUC.................    3.85758
.........................................................................GAUUGAGCCAUGCCAAUAUC.................    7.90804
.........................................................................GAUUGAGCCGUGCCAACAUC.................    3.85758
.........................................................................GAUUGAGCCGUGCCAGUAUC.................    2.50743
.........................................................................GAUUGAGCUGUGCCAAUAUC.................    8.48668
.........................................................................GAUUGAGCCGUGCCGAUAUC.................    5.01486
.........................................................................GAUUGAGCCGUGCCAAUACC.................    2.70031
........................................................................UGAUUGAGCCGUGCCAAUAU..................    519.460044
........................................................................UGAUUGAGCCGUGCCAAU....................    80.757837
........................................................................UGAUUGAGCCGUGCCAAUAUCA................    131.412047
........................................................................UGAUUGAGCCGUGCCAAUA...................    253.099266
........................................................................UGAUUGAGCCGUGCCAGUAUC.................    52.269209
........................................................................UGAUUGCGCCGUGCCAAUAUC.................    45.54109
........................................................................UGAUUGAGCCGGGCCAAUAUC.................    104.768169
........................................................................UGAUUGAGCCGUGCUAAUAUC.................    61.711192
........................................................................UGAUGGAGCCGUGCCAAUAUC.................    16.57303
........................................................................UGAUUGAGCCGAGCCAAUAUC.................    32.82447
........................................................................UGAUUGAGGCGUGCCAAUAUC.................    19.10187
........................................................................UGAUUGAGCCGUGCCGAUAUC.................    56.489393
........................................................................UGAGUGAGCCGUGCCAAUAUC.................    43.316798
........................................................................UGAUUGAGCCGUGCGAAUAUC.................    7.43492
........................................................................UGAUUGAGCAGUGCCAAUAUC.................    76.172984
........................................................................UGAUUGAGCCGUGUCAAUAUC.................    97.237935
........................................................................UGAUUGAGCCGUACCAAUAUC.................    54.340798
........................................................................UGAUUGAGCCGUUCCAAUAUC.................    111.306088
........................................................................UGAUUGAUCCGUGCCAAUAUC.................    67.193462
........................................................................UGAUUGAGCCGUGCCAAUUU..................    32.03288
........................................................................UGAUUGAGCCGUGCCUAUAUC.................    24.070366
........................................................................UGAUUGAGCCGUGCCAAUUUC.................    29.55859
........................................................................UUAUUGAGCCGUGCCAAUAUC.................    111.300891
........................................................................UGAUUGAGCCGUGCCAAUAUG.................    22.035681
........................................................................UGAUUGAGCCCUGCCAAUAUC.................    30.08748
........................................................................UGAUUGAGUCGUGCCAAUAUC.................    178.071637
........................................................................UGAUUGAGCCGUGCCAAUACC.................    100.805743
........................................................................UGAUUGAGCCGUGGCAAUAUC.................    19.08152
........................................................................UGAUUGAGCCGUGCCAAUAUU.................    352.612951
........................................................................UGAUUGAGCCGUGCCAAUAUA.................    157.194939
........................................................................UGAUUGAGCCGUCCCAAUAUC.................    40.289629
........................................................................CGAUUGAGCCGUGCCAAUAUC.................    113.740769
........................................................................UGAUUGAGCCGUGCCAAGAUC.................    18.12679
........................................................................UGAUAGAGCCGUGCCAAUAUC.................    9.49441
........................................................................UGAUUGAGCCGUGCCAAUAUCG................    9.82041
........................................................................UGAUUGAGCCGUGCCAAUAUCC................    131.39027
........................................................................UGCUUGAGCCGUGCCAAUAUC.................    103.135059
........................................................................UGAUUGAGCGGUGCCAAUAUC.................    15.51776
........................................................................UGAUUGAACCGUGCCAAUAUC.................    50.866178
........................................................................AGAUUGAGCCGUGCCAAUAUC.................    32.918334
........................................................................GGAUUGAGCCGUGCCAAUAUC.................    126.493245
........................................................................UGAUUAAGCCGUGCCAAUAUC.................    25.060514
........................................................................UCAUUGAGCCGUGCCAAUAUC.................    18.4335
........................................................................UGAUUGAGCCGUGCCAACAUC.................    42.022085
........................................................................UGAUUGAGCUGUGCCAAUAUC.................    96.569206
........................................................................UGAUUGAGCCGUGCCAAUGUC.................    40.229137
........................................................................UGAUUGAGCCUUGCCAAUAUC.................    103.005721
........................................................................UGAUUGAGCCGUGCCAAAAUC.................    3.67141
........................................................................UGAUUGAGCCGUGCCAAUCUC.................    36.758104
........................................................................UGAUCGAGCCGUGCCAAUAUC.................    52.909318
........................................................................UGAUUGACCCGUGCCAAUAUC.................    65.044061
........................................................................UGAUUGAGCCGUGCCAAUAGC.................    34.9994
........................................................................UAAUUGAGCCGUGCCAAUAUC.................    30.047405
........................................................................UGAUUGAGCCGUGACAAUAUC.................    91.7386
........................................................................UGAAUGAGCCGUGCCAAUAUC.................    6.924
........................................................................UGAUUGAGCCGUGCCAAUC...................    19.98699
........................................................................UGAUUGAGCCGUGCAAAUAUC.................    53.1025
........................................................................UGAUUGAGACGUGCCAAUAUC.................    64.913135
........................................................................UGAUUGAGCCAUGCCAAUAUC.................    62.387571
........................................................................UGAUUGUGCCGUGCCAAUAUC.................    5.16365
........................................................................UGGUUGAGCCGUGCCAAUAUC.................    53.74719
........................................................................UGAUUGAGCCGUGCCAAUACCA................    22.06487
........................................................................UGAUUGGGCCGUGCCAAUAUC.................    79.264387
........................................................................UGAUUUAGCCGUGCCAAUAUC.................    75.421776
........................................................................UGAUUGAGCCGUGCCACUAUC.................    89.54748
........................................................................UGAUUCAGCCGUGCCAAUAUC.................    64.859455
........................................................................UGAUUGAGCCGUGCCCAUAUC.................    77.67629
........................................................................UGUUUGAGCCGUGCCAAUAUC.................    7.15019
........................................................................UGAUUGAGCCGUGCCAAUAAC.................    17.84542
........................................................................UGAUUGAGCCGUGCCAUUAUC.................    39.01943
.......................................................................UUGAUUGAGCCGUGCCAAUAUC.................    17.09109
................UAUUGGCGAGGUUCAAUCAGA.........................................................................    4.677886
.............GGAUAUUGGCGCGGUUCAAUC............................................................................    18.330614
```

```
                                                         Star                                        Mature
GCGGUAAGAAUGCUGUGGCUGACCCAAUAUAAUAGAUCUCUGGGGAAUCGAAAACCUACGGGAUAUUGGUGCGGUUCAAUCAGAAAGCUUGCGCUCCAAAGCCCAGGGGCUCCACUCUUUGACUGAGCCGUGCCAAUAUCACGUCCUCGCUUUGCUUGCCCA    345.2
(.((((((((.((.(.((....((((..............)))).............(((.(((((((((((((((((.(((((.((...(.(((((........))))).)..)).))))).))))))))))))))))).))))).))).)).))))))).
..........................................................................................................................CUGAGCCGUGCCAAUAUCACGU..................    202.903562
...........................................................................................................................UGAGCCGUGCCAAUAUCACG...................    10.4155
..........................................................................................................................CUGAGCCGUGCCAAUAUCACG...................    71.187293
..........................................................................................................................CUGAGCCGUGCCAAUAUCACGC..................    0.895324
..........................................................................................................................CUGAGCCGUGCCAAUAUCACGA..................    1.5598
..........................................................................................................................CUGAGCCGUGCCAAUAUCACA...................    1.02323
..............................................................AUAUUGGUGCGGUUCAAUCA................................................................................    0.334392
.........................................................ACGGGAUAUUGGUGCGGUUCA....................................................................................    1.12477
```

zma-MIR171c

Chromosome: chr2
Start: 9079605
End: 9079766
Strand: +
Strand bias: 1.0
Abundance bias: 0.838
MFE: -55.60

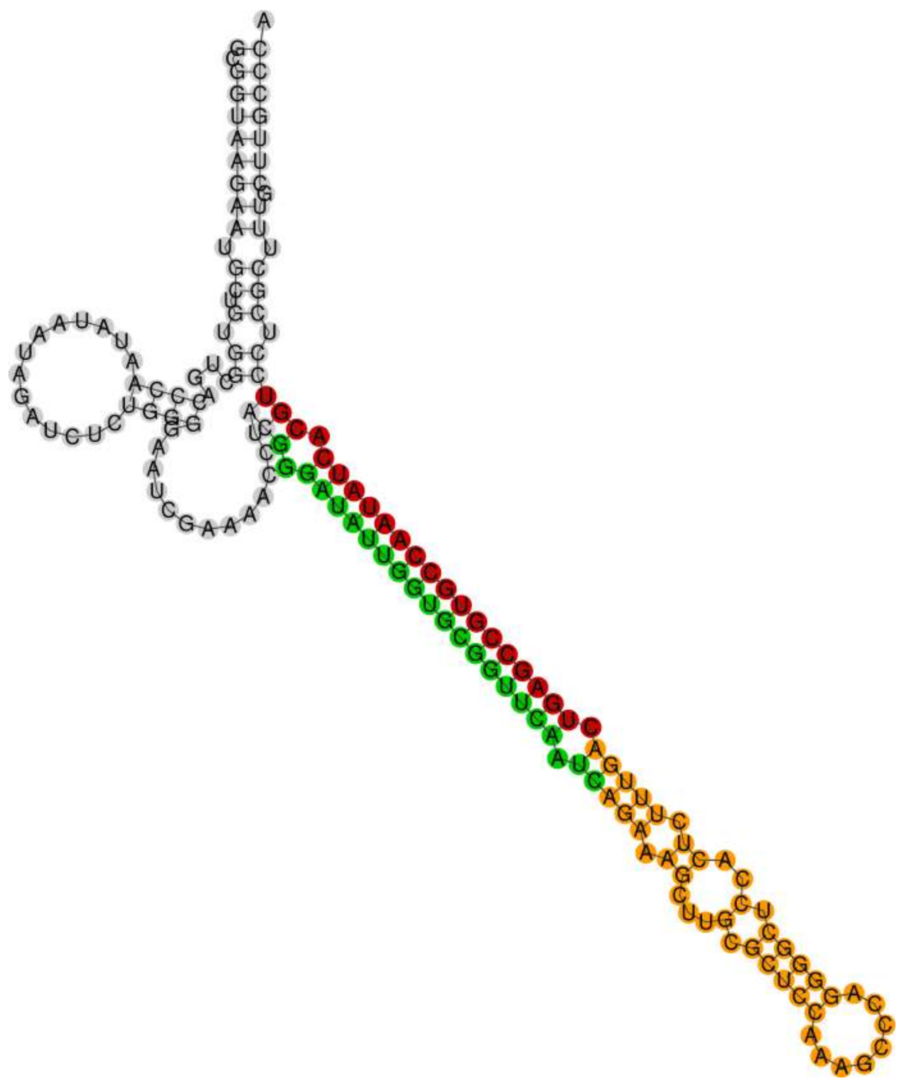

zma-MIR171h

Chromosome: chr2
Start: 18027101
End: 18027224
Strand: -
Strand bias: 1.0
Abundance bias: 0.998
MFE: -56.50

```
                      Star                                                                Mature
CGGGAGGAAGGAGACGACAUGGCGUGGUAUUGUUUCGGCUCAUGUCCUUCUUCGAUCGAGUCUUGCCGUUGGAUUUGGAUGUGAUGUGAGCCGAACCAAUAUCACUCAUGUACUCUUCAUUCCC    10612.9
.(((((..(((((...((((((.(((((((((.(((((((((((((.(((..((((.((........)).)))).))).).))))))))))))).)))))))))))))))).))))))..)))))

......................CGUGGUAUUGUUUCGGCUCAUG................................................................................    5703.478521
........................................................................................AGCCGAACCAAUAUCACUCA................    0.703221
........................................................................................AGCCGAACCAAUAUCACUC.................    2.16664
......................................................................................UGAGCCGAACCAAUAUCACUC.................    3944.691286
......................................................................................UGAGCCGAACCAAUAUCACU..................    58.627843
......................................................................................UGAGCCGAACCAAUAUCACUU.................    7.72458
......................................................................................UGAGCCGAACCAAUCUCACUC.................    1.64005
......................................................................................UGAGCCGAACCAAUAUCACUCU................    5.039599
......................................................................................UGAGCCGAACCCAUAUCACUC.................    4.811535
......................................................................................UGAGCCGAACCAAUAUCACUA.................    3.07342
......................................................................................UGAGCCGAACCACUAUCACUC.................    7.10694
......................CGUGGUAUUGUUUCGGCUCAUGUC..............................................................................    19.05264
......................CGUGGUAUUGUUUCGGCUC...................................................................................    13.46566
......................CGUGGUAUUGUUUCGGCUCA..................................................................................    63.046958
......................CGUGGUAUUGUUUCGGCUCAU.................................................................................    94.123982
......................CGUGGUAUUGUUUCGGCUCAUGU...............................................................................    16.491683
......................CGUGGCAUUGUUUCGGCUCAUG................................................................................    1.89236
......................CGUGGUAUUGUUUCGGCUCAC.................................................................................    3.44812
......................CGUGGUAUUGUUUCGGCUCCUG................................................................................    7.20537
......................CGUGGGAUUGUUUCGGCUCAUG................................................................................    0.228587
......................CGUGGUAUUGUUUCGGCUCAUC................................................................................    9.87312
......................CGUGGUAUUGUUUCGGCUCAUA................................................................................    494.76734
......................CGUGGUAUUGUUUCGGCUCAUU................................................................................    23.721338
......................CGUGGUAUUGUUUCGGCUCGUG................................................................................    7.45304
......................CGUGGUAUUGUUUCGGCUAAUG................................................................................    1.26157
......................CGUGGUAUUGUUUCGGCUCAGG................................................................................    30.22758
......................CGUGGUAUUGUUUCGGCUCAUGC...............................................................................    5.2801
......................CGUGGUAUUGUUUCGGCUCAUGA...............................................................................    10.24905
......................CGUGGUAUUGUUUCGGCUCACG................................................................................    47.27844
```

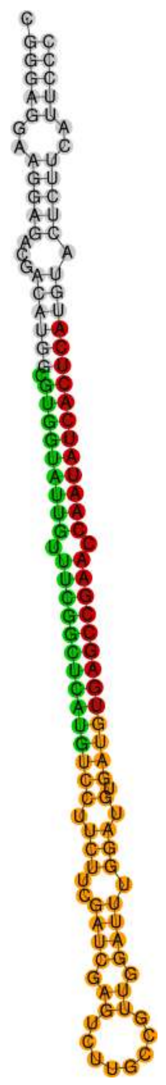

zma-MIR171j

```
Chromosome:    chr10
Start:         59006552
End:           59006679
Strand:        +
Strand bias:   1.0
Abundance bias:1.0
MFE:           -68.60
```

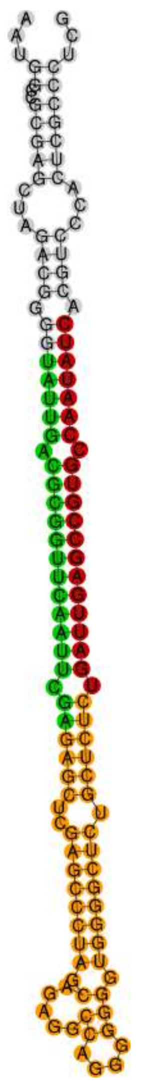

```
                         Star                                                        Mature
AAUGGCCGCGAGCUAGACGGGGUAUUGACGCGGUUCAAUUCGAGAGCUCGAGCCCUAGCAGAGGCCAGGGGGGGUGGGGCUCUGCUCUCUGAUUGAGCCGUGCCAAUAUCACGUCCCACUCGCCCUCG    147568.
...((..(((((...((((.(((((((.(((((((((((.((((((..((((((((.(.....((....))).)))))))).)))))).))))))))))).))))))).))))...)))))))...
.........................................................................................UGAUUGAGCCGUGCCAAUAUC..................    137861.56804
............................................................................................UUGAGCCGUGCCAAUAUCACG...............    525.45142
............................................................................................UUGAGCCGUGCCAAUAUCAC................    7.81576
............................................................................................UUGAGCCGUGCCAAUAUC..................    47.74514
...........................................................................................AUUGAGCCGUGCCAAUAUC..................    93.864466
..........................................................................................GAUUGAGCCGUGCCAAUA....................    3.85758
..........................................................................................GAUUGAGCCGUGCCAAUAUC..................    2898.392626
..........................................................................................GAUUGAGCCGUGCCAAUAU...................    52.2702
..........................................................................................GAUUGAGCCGUGCUAAUAUC..................    2.50743
..........................................................................................GAUUGAGCCGUGUCAAUAUC..................    5.59349
..........................................................................................GAUUGAGCCCUGCCAAUAUC..................    5.40062
..........................................................................................GAUUGAGCCGUACCAAUAUC..................    2.50743
..........................................................................................GAUUGAGCCGUGCCUAUAUC..................    3.27895
..........................................................................................GAUUGAGUCGUGCCAAUAUC..................    5.78637
..........................................................................................GAUUGAGCCGUGCCCAUAUC..................    3.47182
..........................................................................................GAUUGAGCCUUGCCAAUAUC..................    4.24334
..........................................................................................GAUUGAGCCGUGCCAAUAUU..................    4.82198
..........................................................................................GAUUGAGCCGUGCCAAAAUC..................    3.85758
..........................................................................................GAUUGAGCCAUGCCAAUAUC..................    7.90804
..........................................................................................GAUUGAGCCGUGCCAACAUC..................    3.85758
..........................................................................................GAUUGAGCCGUGCCAGUAUC..................    2.50743
..........................................................................................GAUUGAGCUGUGCCAAUAUC..................    8.48668
..........................................................................................GAUUGAGCCGUGCCGAUAUC..................    5.01486
..........................................................................................GAUUGAGCCGUGCCAAUACC..................    2.70031
.........................................................................................UGAUUGAGCCGUGCCAAUAU...................    519.460044
.........................................................................................UGAUUGAGCCGUGCCAAU.....................    80.757837
.........................................................................................UGAUUGAGCCGUGCCAAUAUCA.................    131.412047
.........................................................................................UGAUUGAGCCGUGCCAAUA....................    253.099266
.........................................................................................UGAUUGAGCCGUGCCAGUAUC..................    52.269209
.........................................................................................UGAUUGCGCCGUGCCAAUAUC..................    45.54109
.........................................................................................UGAUUGAGCCGGGCCAAUAUC..................    104.768169
.........................................................................................UGAUUGAGCCGUGCUAAUAUC..................    61.711192
.........................................................................................UGAUGGAGCCGUGCCAAUAUC..................    16.57303
.........................................................................................UGAUUGAGCCGAGCCAAUAUC..................    32.82447
.........................................................................................UGAUUGAGGCGUGCCAAUAUC..................    19.10187
.........................................................................................UGAUUGAGCCGUGCCGAUAUC..................    56.489393
.........................................................................................UGAGUGAGCCGUGCCAAUAUC..................    43.316798
.........................................................................................UGAUUGAGCCGUGCGAAUAUC..................    7.43492
.........................................................................................UGAUUGAGCAGUGCCAAUAUC..................    76.172984
.........................................................................................UGAUUGAGCCGUGUCAAUAUC..................    97.237935
.........................................................................................UGAUUGAGCCGUACCAAUAUC..................    54.340798
.........................................................................................UGAUUGAGCCGUUCCAAUAUC..................    111.306088
.........................................................................................UGAUUGAUCCGUGCCAAUAUC..................    67.193462
.........................................................................................UGAUUGAGCCGUGCCAAUUU...................    32.03288
.........................................................................................UGAUUGAGCCGUGCCUAUAUC..................    24.070366
.........................................................................................UGAUUGAGCCGUGCCAAUUUC..................    29.55859
.........................................................................................UUAUUGAGCCGUGCCAAUAUC..................    111.300891
.........................................................................................UGAUUGAGCCGUGCCAAUAUG..................    22.035681
.........................................................................................UGAUUGAGCCCUGCCAAUAUC..................    30.08748
.........................................................................................UGAUUGAGUCGUGCCAAUAUC..................    178.071637
.........................................................................................UGAUUGAGCCGUGCCAAUACC..................    100.805743
.........................................................................................UGAUUGAGCCGUGGCAAUAUC..................    19.08152
.........................................................................................UGAUUGAGCCGUGCCAAUAUU..................    352.612951
.........................................................................................UGAUUGAGCCGUGCCAAUAUA..................    157.194939
.........................................................................................UGAUUGAGCCGUCCCAAUAUC..................    40.289629
.........................................................................................CGAUUGAGCCGUGCCAAUAUC..................    113.740769
.........................................................................................UGAUUGAGCCGUGCCAAGAUC..................    18.12679
.........................................................................................UGAUAGAGCCGUGCCAAUAUC..................    9.49441
.........................................................................................UGAUUGAGCCGUGCCAAUAUCG.................    9.82041
.........................................................................................UGAUUGAGCCGUGCCAAUAUCC.................    131.39027
.........................................................................................UGCUUGAGCCGUGCCAAUAUC..................    103.135059
.........................................................................................UGAUUGAGCGGUGCCAAUAUC..................    15.51776
.........................................................................................UGAUUGAACCGUGCCAAUAUC..................    50.866178
.........................................................................................AGAUUGAGCCGUGCCAAUAUC..................    32.918334
.........................................................................................GGAUUGAGCCGUGCCAAUAUC..................    126.493245
.........................................................................................UGAUUAAGCCGUGCCAAUAUC..................    25.060514
.........................................................................................UCAUUGAGCCGUGCCAAUAUC..................    18.4335
.........................................................................................UGAUUGAGCCGUGCCAACAUC..................    42.022085
.........................................................................................UGAUUGAGCUGUGCCAAUAUC..................    96.569206
.........................................................................................UGAUUGAGCCGUGCCAAUGUC..................    40.229137
.........................................................................................UGAUUGAGCCUUGCCAAUAUC..................    103.005721
.........................................................................................UGAUUGAGCCGUGCCAAAAUC..................    3.67141
.........................................................................................UGAUUGAGCCGUGCCAAUCUC..................    36.758104
.........................................................................................UGAUCGAGCCGUGCCAAUAUC..................    52.909318
.........................................................................................UGAUUGACCCGUGCCAAUAUC..................    65.044061
.........................................................................................UGAUUGAGCCGUGCCAAUAGC..................    34.9994
.........................................................................................UAAUUGAGCCGUGCCAAUAUC..................    30.047405
.........................................................................................UGAUUGAGCCGUGACAAUAUC..................    91.7386
.........................................................................................UGAAUGAGCCGUGCCAAUAUC..................    6.924
.........................................................................................UGAUUGAGCCGUGCCAAUC....................    19.98699
.........................................................................................UGAUUGAGCCGUGCAAAUAUC..................    53.1025
.........................................................................................UGAUUGAGACGUGCCAAUAUC..................    64.913135
.........................................................................................UGAUUGAGCCAUGCCAAUAUC..................    62.387571
.........................................................................................UGAUUGUGCCGUGCCAAUAUC..................    5.16365
.........................................................................................UGGUUGAGCCGUGCCAAUAUC..................    53.74719
.........................................................................................UGAUUGAGCCGUGCCAAUACCA.................    22.06487
.........................................................................................UGAUUGGGCCGUGCCAAUAUC..................    79.264387
.........................................................................................UGAUUUAGCCGUGCCAAUAUC..................    75.421776
.........................................................................................UGAUUGAGCCGUGCCACUAUC..................    89.54748
.........................................................................................UGAUUCAGCCGUGCCAAUAUC..................    64.859455
.........................................................................................UGAUUGAGCCGUGCCCAUAUC..................    77.67629
.........................................................................................UGUUUGAGCCGUGCCAAUAUC..................    7.15019
.........................................................................................UGAUUGAGCCGUGCCAAUAAC..................    17.84542
.........................................................................................UGAUUGAGCCGUGCCAUUAUC..................    39.01943
........................................................................................CUGAUUGAGCCGUGCCAAUAUC..................    870.443047
........................................................................................CUGAUUGAGCCGUGCCAAUAU...................    30.064876
........................................................................................CUGAUUGAGCCGUGCCAAUAUU..................    4.697762
.......................................................................................UCUGAUUGAGCCGUGCCAAUA....................    7.625407
.......................................................................................UCUGAUUGAGCCGUGCCAAUAUC..................    132.37052
```

zma-MIR171k

Chromosome: chr10
Start: 138536839
End: 138536939
Strand: -
Strand bias: 1.0
Abundance bias: 0.998
MFE: -51.00

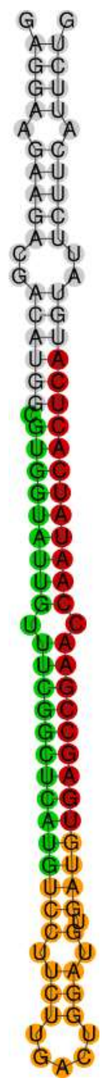

Star   Mature

```
GAGGAAGAAGACGACAUGGCGUGGUAUUGUUUCGGCUCAUGUCCUUCUUGACUGGAUGUGAUGUGAGCCGAACCAAUAUCACUCAUGUAUUCUUCAUUCUG    10612.9
.((((.(((((..((((((.(((((((((.((((((((((((((.(((.....))).).))))))))))))).))))))))))))))..))))).)))).

...................CGUGGUAUUGUUUCGGCUCAUG............................................................    5703.478521
.................................................................AGCCGAACCAAUAUCACUCA................    0.703221
.................................................................AGCCGAACCAAUAUCACUC.................    2.16664
...............................................................UGAGCCGAACCAAUAUCACUC.................    3944.691286
...............................................................UGAGCCGAACCAAUAUCACU..................    58.627843
...............................................................UGAGCCGAACCAAUAUCACUU.................    7.72458
...............................................................UGAGCCGAACCAAUCUCACUC.................    1.64005
...............................................................UGAGCCGAACCAAUAUCACUCU................    5.039599
...............................................................UGAGCCGAACCCAUAUCACUC.................    4.811535
...............................................................UGAGCCGAACCAAUAUCACUA.................    3.07342
...............................................................UGAGCCGAACCACUAUCACUC.................    7.10694
...................CGUGGUAUUGUUUCGGCUCAUGUC..........................................................    19.05264
...................CGUGGUAUUGUUUCGGCUC...............................................................    13.46566
...................CGUGGUAUUGUUUCGGCUCA..............................................................    63.046958
...................CGUGGUAUUGUUUCGGCUCAU.............................................................    94.123982
...................CGUGGUAUUGUUUCGGCUCAUGU...........................................................    16.491683
...................CGUGGCAUUGUUUCGGCUCAUG............................................................    1.89236
...................CGUGGUAUUGUUUCGGCUCAC.............................................................    3.44812
...................CGUGGUAUUGUUUCGGCUCCUG............................................................    7.20537
...................CGUGGGAUUGUUUCGGCUCAUG............................................................    0.228587
...................CGUGGUAUUGUUUCGGCUCAUC............................................................    9.87312
...................CGUGGUAUUGUUUCGGCUCAUA............................................................    494.76734
...................CGUGGUAUUGUUUCGGCUCAUU............................................................    23.721338
...................CGUGGUAUUGUUUCGGCUCGUG............................................................    7.45304
...................CGUGGUAUUGUUUCGGCUAAUG............................................................    1.26157
...................CGUGGUAUUGUUUCGGCUCAGG............................................................    30.22758
...................CGUGGUAUUGUUUCGGCUCAUGC...........................................................    5.2801
...................CGUGGUAUUGUUUCGGCUCAUGA...........................................................    10.24905
...................CGUGGUAUUGUUUCGGCUCACG............................................................    47.27844
```

zma-MIR2118c

Chromosome: chr10
Start: 118813624
End: 118813732
Strand: -
Strand bias: 1.0
Abundance bias: 0.992
MFE: -54.20

```
                   Star                                              Mature
AGAUGAUCUAAGAGCAGUGGGCAUGGGAACAUGGAGGAAGGCCUAUGAGGUUGAAACCUCAUUUUGUGUCUUCCUAAUGCCUCCCAUUCCUAUCGUUCUUAGGUGAUCC    128.1
.(((.((((((((((.(((((.((((((.(((..(((((((((.(((((((....)))))))...).)))))))).)))..)))))).))))).)))))))))).))).
......................................................................UUCCUAAUGCCUCCCAUUCCUA.................    97.395251
.......................................................................UCCUAAUGCCUCCCAUUCCU..................    0.784328
.......................................................................UCCUAAUGCCUCCCAUUCCUA.................    0.53337
......................................................................UUCCUAAUGCCUCCCAUUCC...................    1.114509
......................................................................UUCCUAAUGCCUCCCAUUCCU..................    25.99254
......................................................................UUCCUAAUGCCUCCCAUUCCUAA................    0.152391
......................................................................UUCCUAAUGCCUCCCAUUCCUUU................    0.152391
...................GGCAUGGGAACACGGAGGAAGG....................................................................    0.986844
```

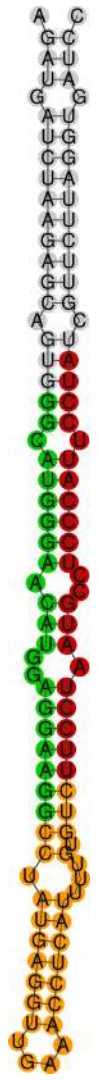

zma-MIR2118a

Chromosome: chr10
Start: 118790438
End: 118790645
Strand: -
Strand bias: 1.0
Abundance bias:0.866
MFE: -63.10

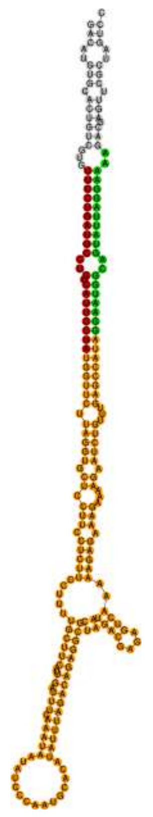

```
                  Mature                                                                                                                                                       Star
GACAUGUGCACUGUCGUGUUCCUGAUGCCUCUCAUUCCUAUGGUUCUUAGGUGCUCCUUCCUCUUCCUUUUGCUUCAUUGAUUUCAAAUAAUAUCCCAAUGCACAUAUUUAGACAGAGGCGCUAAUGACGAGAGUCAAAAAGAGAAAGAAAAGAAUCUGUUUGAGCCAUAGGAAUGGCAGUAUUAGGAAAAGACGAGUUCGCUAGUCC    215.0
(((..(((.((((((...((((((((((..(.((((((((((((((.(((((.((.(((.(((((.....((((((..((.(((.(((((..............))))))))))))))))....((((....))))..))))).)))...)).)))))...)))))))))))))))..))))))))))..))).))).)))..))).
..................UUCCUGAUGCCUCUCAUUCCUA........................................................................................................................................................................    100.7872
................UGUUCCUGAUGCCUCUCAUUC...........................................................................................................................................................................    0.57042
..................UUCCUGAUGCCUCUCAUUCCUAU.......................................................................................................................................................................    0.152391
..................UUCCUGAUGCCUCUCAUUCCU.........................................................................................................................................................................    75.02077
..................UUCCUGAUGCCUCUCAUUCC..........................................................................................................................................................................    5.381009
..................UUCCUGAUGCCUCUCAUUU...........................................................................................................................................................................    0.228587
..................UUCCUGAUGCCUUUCAUUCCU.........................................................................................................................................................................    1.06954
...................UCCUGAUGCCUCUCAUUCCU.........................................................................................................................................................................    0.427815
...................UCCUGAUGCCUCUCAUUCCUA........................................................................................................................................................................    0.330181
....................CCUGAUGCCUCUCAUUCCU.........................................................................................................................................................................    0.998236
.......................................................................................................................................................................AUAGGAAUGGCAGUAUUAGGA....................    1.164373
.......................................................................................................................................................................AUAGGAAUGGCAGCAUUAGGA....................    0.152391
```

zma-MIR2118g

Chromosome: chr2
Start: 51409119
End: 51409379
Strand: +
Strand bias: 1.0
Abundance bias: 0.852
MFE: -75.00

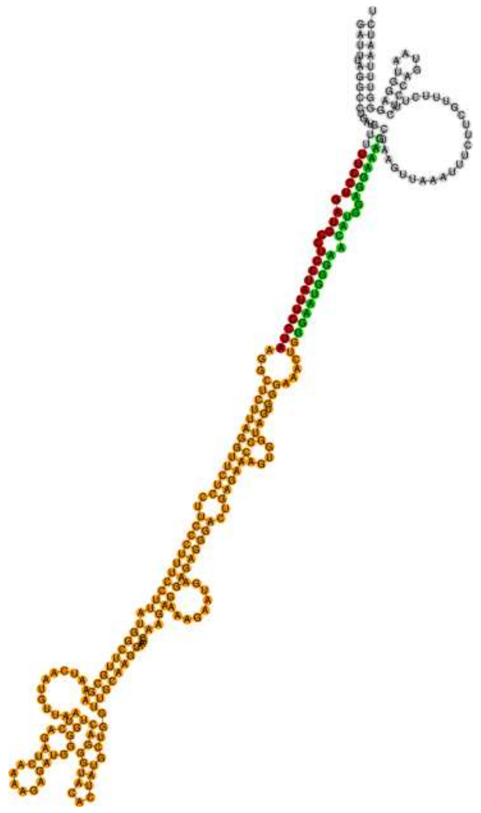

Mature   Star

```
GAUUUAGGCCUGAUGUUUUCCUGAUGCCUCCUAUUCCUAAGGCUCUUAGGUUCUCCUUCCCUUUCCUUAUGGCUUGCGAAAUCAAUGUUAAUCAGAUCAAAAGAGAUGGGGUCAGGGUACACUAUGCUGGUUGCAAGCAAGCAAGAGAAAGAAUGAGAGAGGGACUGAGAACAGUGGCUAGUGGGAAACUGGGAAUGGGAACAUGGAGGAAAGCUAAGUUAAAUUUCUUCGUUUCUUCUCCAGUAAUGGAGGGUUUAAUCU 484.5
((((.(((((....((((((((.(((..((((((((((...((((((((((((..((((((((((.(((((((((..........(((..(((......)))..)))(((.(((...))).))).))))))))...)).)))........))))))))..))))))).....)))).)))....))))))))))).)))..))))))))......................(((((....))))))))))))))).
.................UUCCUGAUGCCUCCUAUUCCUA...................................................................................................................................................................................................................................... 328.626585
...............UUUUCCUGAUGCCUCCUAUUC......................................................................................................................................................................................................................................... 0.203188
...............UUUUCCUGAUGCCUCCUAUUCC........................................................................................................................................................................................................................................ 2.319895
...............UUUUCCUGAUGCCUCCUAUUCCUA...................................................................................................................................................................................................................................... 0.656402
...............UUUUCCUGAUGCCUCCUAUUCCU....................................................................................................................................................................................................................................... 1.491524
...............UUUUCCUGAUGCCUCCUAUUCCUAU..................................................................................................................................................................................................................................... 0.228587
...............UUUUCCUGAUGCCUCCUAUUCCUU...................................................................................................................................................................................................................................... 8.87996
................UUUCCUGAUGCCUCCUAUUCCU....................................................................................................................................................................................................................................... 0.17779
................UUUCCUGAUGCCUCCUAUUCC........................................................................................................................................................................................................................................ 0.380978
................UUUCCUGAUGCCUCCUAUUCCUU...................................................................................................................................................................................................................................... 0.499118
.................UUCCUGAUGCCUCCUAUUCC........................................................................................................................................................................................................................................ 6.90003
.................UUCCUGAUGCCUCCUAUUC......................................................................................................................................................................................................................................... 0.279384
.................UUCCUGAUGCCUCCUAUUCCU....................................................................................................................................................................................................................................... 58.5968
.................UUCCUGAUGCCUCCUAUUCCUAA..................................................................................................................................................................................................................................... 0.228587
.................UUCCUGAUGCCUCCUAUUCU........................................................................................................................................................................................................................................ 0.253986
.................UUCCUGAUGCCUCCUAUUU......................................................................................................................................................................................................................................... 0.330181
.................UUCCCGAUGCCUCCUAUUCCUA...................................................................................................................................................................................................................................... 0.17779
.................UUCCUGAUGCCUCCUAUUCCUAU..................................................................................................................................................................................................................................... 1.04134
.................UUCCUGAUGCCUCCUAUUCCUU...................................................................................................................................................................................................................................... 1.246395
..................UCCUGAUGCCUCCUAUUCCUA...................................................................................................................................................................................................................................... 0.279384
```

zma-MIR395g

Chromosome: chr2
Start: 6277091
End: 6277191
Strand: -
Strand bias: 1.0
Abundance bias: 1.0
MFE: -44.80

```
                                          Star                                                  Mature
UAGGUUUGAUGUCCCUAGGAGUUCUAUGCAAGCACUUCACGAGGCAUUGUUUCAAGAGCUUUUGUGAAGUGUUUGGGGGAACUCUUGGUUUCUCAAAUCAU    6693.8
..(((((((.(...(((((((((((.(.(((((((((((((((((.((......)).)).))))))))))))))).))))))))))))...))))))))..
................................................................UGAAGUGUUUGGGGGAACUC.................    4586.749169
..................................................................AAGUGUUUGGGGGAACUC.................    4.22703
..................................................................AAGUGUUUGGGGGAACUCU................    3.15555
.................................................................GAAGUGUUUGGGGGAACU..................    43.736
.................................................................GAAGUGUUUGGGGGAACUC.................    5.1317
................................................................UGAAGUGUUUGGGGGAAC...................    117.318839
................................................................UGAAGUGUUUGGGGGAACUCU................    66.540551
................................................................UGAAGUGUUUGGGGGAACU..................    897.367501
................................................................UGAAGUGUUUGGGGGAAU...................    13.78406
................................................................UGGAGUGUUUGGGGGAACUC.................    3.28276
................................................................UGAAGUGUUUGGGGGAACG..................    9.43161
................................................................UGAAGUGUUUGGGGGAACA..................    48.00465
................................................................UGAAGUGUUUGGGGGAACC..................    111.29543
................................................................CGAAGUGUUUGGGGGAACUC.................    1.74419
................................................................UGAAGUGUUUGGGGGAACUCA................    21.45689
................................................................UGAAGUGUUUGGGGGAACUA.................    173.561085
................................................................UGAAGUGUUUGGGGGAACUU.................    494.126627
................................................................UGAAGUGUUUGGGGGCACUC.................    3.41142
................................................................UGAAGUGUUUGGGGGAACGC.................    7.60419
................................................................UGAAGUGUUUGGGGGGACUC.................    1.74419
................................................................UGAAGUGUUUGGGGGAACUUUU...............    5.28629
................................................................UGAAGUGUUUGGGGGAACCC.................    31.73744
................................................................UGAAGUGUUUGGGGGAACUCC................    17.66074
...............................................................GUGAAGUGUUUGGGGGAAC...................    1.94188
...............................................................GUGAAGUGUUUGGGGGAACUC.................    8.66896
...............................................................GUGAAGUGUUUGGGGGAACU..................    14.84634
```

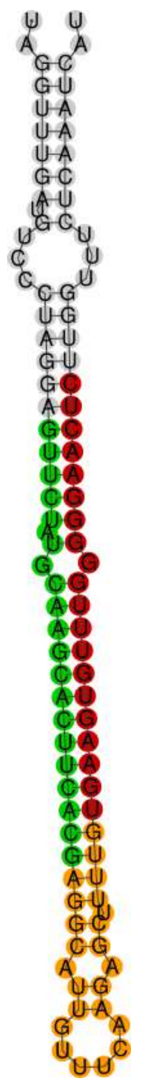

zma-MIR395k

Chromosome: chr10
Start: 78600282
End: 78600579
Strand: -
Strand bias: 1.0
Abundance bias: 0.999
MFE: -114.30

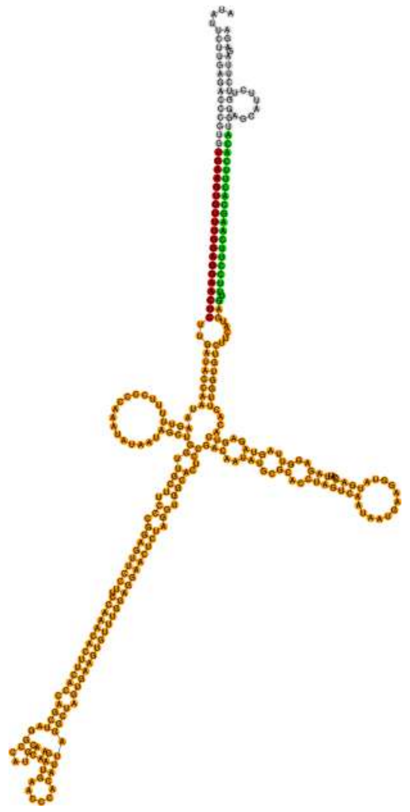

Mature
Star

```
AUAUUCUUGAGACCCGUGUGAAGUGUUUGGAGGAACUCUUGAUACCAAUAAGUUUUUCCCCAAAUAUAAUAGCUGGUUGCUUCCCGGAGUUCCUUCCAAACACUUCACCAGCUAGGCCAUGCACAAGAUGAACCCACAUUAGGCUAGUGAAGUGUUUGGAGGAACUCUAGGUGGCACUCCGACAAUAUGCGCACCUAGUCAAUAAUGAAGGUAUGACCAUUAGAGGUUAGUAGAGUCACACUUGGUGUCUUCAUGAGUUUCCUUCAAGCACUUCACAUGGAGCAUUCUGUCUUAGAGA 192.3
....((((((((((((((((((((((((((((((((((..((((((((..((((...............))))((.((((.((.(((((((((.((((((((((((.((((..((...))....((((......)))).)))).))))))))))))))))))))))).)).))))..))(((..(((..((..(((((((...........))))...)))..))..)))..)))....))))))))).....))).)))))))))))))))))))))).......)))))).)))
..................UGAAGUGUUUGGAGGAACUC.................................................................................................................................................................................................................................................................... 108.473745
..................UGAAGUGUUUGGAGGAAC...................................................................................................................................................................................................................................................................... 6.7799
..................UGAAGUGUUUGGAGGAACUCU.................................................................................................................................................................................................................................................................. 2.95892
..................UGAAGUGUUUGGAGGAACU..................................................................................................................................................................................................................................................................... 49.722191
..................UGAAGUGUUUGGAGGAACUA................................................................................................................................................................................................................................................................... 2.53622
..................UGAAGUGUUUGGAGGAACUU................................................................................................................................................................................................................................................................... 10.41321
..................UGAAGUGUUUGGAGGAACA.................................................................................................................................................................................................................................................................... 2.53622
..................UGAAGUGUUUGGAGGAACC.................................................................................................................................................................................................................................................................... 3.80433
..................UGAAGUGUUUGGAGGAACUUUU................................................................................................................................................................................................................................................................. 3.39828
..................UGAAGUGUUUGGAGGAACUUU.................................................................................................................................................................................................................................................................. 1.45641
```

zma-MIR395d

```
Chromosome:    chr2
Start:         6276136
End:           6276329
Strand:        -
Strand bias:   1.0
Abundance bias:1.0
MFE:           -55.60
```

```
                  Mature                                                                                                                      Star
UUGUUUCAAGAGCUUUUGUGAAGUGUUUGGGGGAACUCUUGUCUCAAAUCAUUUGCAUGUGCUAGAUGUUUGUCAUGUGUCAUGUGAUACGAGUUGAAGGCUAUACAACAUUUUGUGUUACUAGGAGUUCCCUACAAGCACGUCUCGAUACCUUGGCUUAAUAAAGGGUAUAAUUGUGAAGUGUUUGGGGGAGA    6693.8
((.(((((((.(((((((.((.(((((((((((((((((((...((((.(((((((....)).)))))))))...........((((((((((.((..(......)..)).)))))))))))))))))))))).))))))).)).))(((((((..........)))))))......))))).))))))).)).
..................UGAAGUGUUUGGGGGAACUC............................................................................................................................................................    4586.749169
.................GUGAAGUGUUUGGGGGAAC..............................................................................................................................................................    1.94188
.................GUGAAGUGUUUGGGGGAACUC............................................................................................................................................................    8.66896
.................GUGAAGUGUUUGGGGGAACU.............................................................................................................................................................    14.84634
..................UGAAGUGUUUGGGGGAAC..............................................................................................................................................................    117.318839
..................UGAAGUGUUUGGGGGAACUCU...........................................................................................................................................................    66.540551
..................UGAAGUGUUUGGGGGAACU.............................................................................................................................................................    897.367501
..................UGAAGUGUUUGGGGGAAU..............................................................................................................................................................    13.78406
..................UGGAGUGUUUGGGGGAACUC............................................................................................................................................................    3.28276
..................UGAAGUGUUUGGGGGAACG.............................................................................................................................................................    9.43161
..................UGAAGUGUUUGGGGGAACA.............................................................................................................................................................    48.00465
..................UGAAGUGUUUGGGGGAACC.............................................................................................................................................................    111.29543
..................CGAAGUGUUUGGGGGAACUC............................................................................................................................................................    1.74419
..................UGAAGUGUUUGGGGGAACUCA...........................................................................................................................................................    21.45689
..................UGAAGUGUUUGGGGGAACUA............................................................................................................................................................    173.561085
..................UGAAGUGUUUGGGGGAACUU............................................................................................................................................................    494.126627
..................UGAAGUGUUUGGGGGCACUC............................................................................................................................................................    3.41142
..................UGAAGUGUUUGGGGGAACGC............................................................................................................................................................    7.60419
..................UGAAGUGUUUGGGGGGACUC............................................................................................................................................................    1.74419
..................UGAAGUGUUUGGGGGAACUUUU.........................................................................................................................................................    5.28629
..................UGAAGUGUUUGGGGGAACCC............................................................................................................................................................    31.73744
..................UGAAGUGUUUGGGGGAACUCC...........................................................................................................................................................    17.66074
...................GAAGUGUUUGGGGGAACU.............................................................................................................................................................    43.736
...................GAAGUGUUUGGGGGAACUC............................................................................................................................................................    5.1317
....................AAGUGUUUGGGGGAACUC............................................................................................................................................................    4.22703
....................AAGUGUUUGGGGGAACUCU...........................................................................................................................................................    3.15555
```

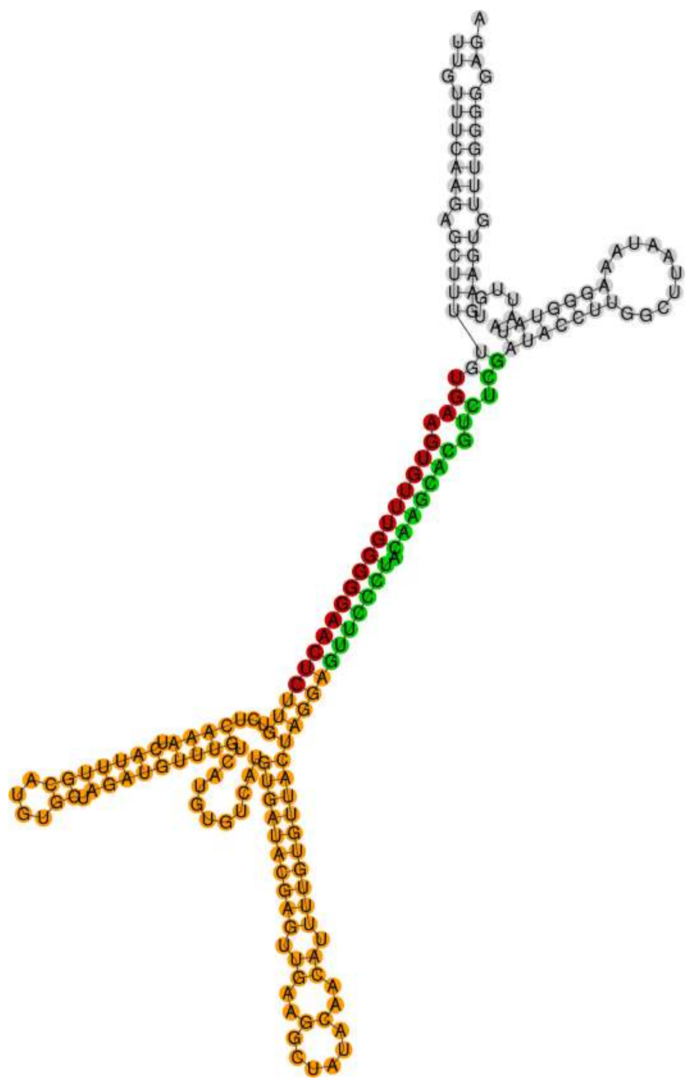

zma-MIR399i

Chromosome: chr5
Start: 150372024
End: 150372148
Strand: +
Strand bias: 1.0
Abundance bias: 1.0
MFE: -59.40

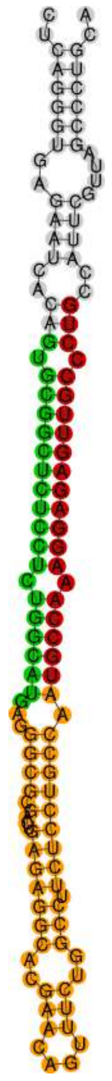

```
                  Star                                                        Mature
CUCAGGGUGAGAAUCACAGUGCGGCUCUCCUCUGGCAUGAGGGCGCGACUGAGAGGCACGAACAGUUUCUGGCCUUCUCCUGCCAAUGCCAAAGGAGAGUUGCCCUGCCAUUCGUUAGCCCUGCA    27617.5
..((((((..((((..(((.(((((((((((.((((((...((((.(...(((((((..(((....)))..))).))))))))).)))))).))))))))))).)))..))))....))))))..
......................................................................................UGCCAAAGGAGAGUUGCCCUG..................    22946.624303
.........................................................................................CAAAGGAGAGUUGCCCUG..................    15.93729
.........................................................................................CAAAGGAGAGUUGCCCUU..................    8.1585
.......................................................................................GCCAAAGGAGAGUUGCCCUG..................    17.87973
.......................................................................................GCCAAAGGAGAGUUGCCCU...................    2.70031
......................................................................................UGCCAAAGGAGAGUUGCCCU...................    3825.479119
......................................................................................UGCCAAAGGAGAGUUGCC.....................    57.15644
......................................................................................UGCCAAAGGAGAGUUGCCC....................    93.159229
......................................................................................UUCCAAAGGAGAGUUGCCCUG..................    5.65725
......................................................................................UGCCAAAGGAGAGUUGCCCG...................    0.963614
......................................................................................UGCCAAAGGAGAGUUUCCCUG..................    29.48094
......................................................................................UGCCAAAGGAGAGUUGACCUG..................    1.08407
......................................................................................UGCCAAAGGGGAGUUGCCCUG..................    0.963614
......................................................................................UGCCAAAGGAGGGUUGCCCUG..................    1.32497
......................................................................................UGCCAAAGGAGAGUUGCCA....................    1.80678
......................................................................................UGCCAAAGGAGAGUUGCCCC...................    19.243644
......................................................................................UGCCAAAGGAGAGUUGCCCA...................    10.730025
......................................................................................UGCCAAAGGAGAGUUGCCCUGA.................    13.655326
......................................................................................UGCCAAAGGAGAGUUGGCCUG..................    0.72271
......................................................................................UGCCAAAGGAGAGUUGCCCCG..................    2.719412
......................................................................................UGCCAAAGGAGAGUUGCCUUG..................    5.26639
......................................................................................UGCCAAAGGAGAUUUGCCCUG..................    15.91686
......................................................................................CGCCAAAGGAGAGUUGCCCUG..................    1.32497
......................................................................................UGCCAAAGGAGAGUUGCU.....................    0.963614
......................................................................................UGCCAAAGGAGAGUUGCCCUC..................    2.04768
......................................................................................UGCCAAAGGAGAGUUGCCCUA..................    327.004254
......................................................................................UGCCAAAGGAGAGUGGCCCUG..................    1.62498
......................................................................................UGCCAAAGGAGAGUUGCCU....................    6.391255
......................................................................................UGCCAAAGGAGAGUCGCCCUG..................    1.32497
......................................................................................UGCCAAGGGAGAGUUGCCCUG..................    0.963614
......................................................................................GGCCAAAGGAGAGUUGCCCUG..................    2.49113
......................................................................................UGCCAAAUGAGAGUUGCCCUG..................    10.16091
......................................................................................UGCCAAAGGAGUGUUGCCCUG..................    22.08632
......................................................................................UGCCAAAGGAGAGUUGCUCUG..................    22.527872
......................................................................................UGCCAAAGGAGAGUUGCCAUG..................    5.77123
......................................................................................UGCCAAAGGAGAGUUGCCCGG..................    22.935655
......................................................................................UGCCAAAGGAGAGUUGCCCUU..................    52.609515
......................................................................................UGCCAAAGUAGAGUUGCCCUG..................    13.58705
......................................................................................UGCCAAAGGAUAGUUGCCCUG..................    21.30165
......................................................................................UGCCAAAGGUGAGUUGCCCUG..................    2.59037
......................................................................................UGCAAAAGGAGAGUUGCCCUG..................    6.09243
......................................................................................UACCAAAGGAGAGUUGCCCUG..................    0.963614
......................................................................................UGCCAAAGGAGAGUUGUCCUG..................    16.18777
```

	Star / Mature	
	CUCAGGGUGAGAAUCACAGUGCGGCUCUCCUCUGGCAUGAAGGCGCGGCUGAGAGGCACGAACAGUUUCUGGCCUUCCCCUGCCAAUGCCAAAGGAGAGCUGCCCUGCCAUUCGUUAGCCCUGCA	503.5
	..((((((..((((..(((.(((((((((((.((((((...((((.((....(((((..(((....)))..)))))..)))))).)))))).))))))))))).)))..))))....))))))..	
	....UGCCAAAGGAGAGCUGCCCUG..................	398.566179
	....UGCCAAAGGAGAGCUGCCCU...................	102.773615
	....UGCCAAAGGAGAGCUGCCCUU..................	2.16664

zma-MIR399_N1

Chromosome: chr5
Start: 150426215
End: 150426339
Strand: +
Strand bias: 1.0
Abundance bias: 1.0
MFE: -62.70

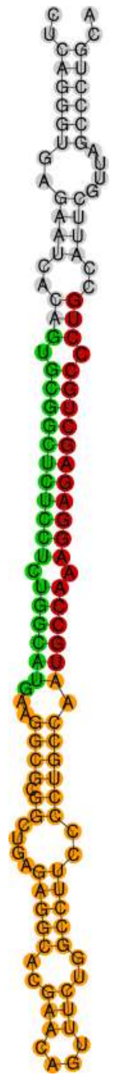

Star Mature

```
CUCAGGGUGAGAAUCACAGUGCGGCUCUCCUCUGGCAUGAAGGCGCGGCUGAGAGGCACGAACAGUUUCUGGCCUUCCCCUGCCAAUGCCAAAGGAGAGCUGCCCUGCCAUUCGUUAGCCCUGCA    503.5
..((((((..((((..(((.(((((((((((.((((((...((((.((....(((((..(((....)))..)))))..)))))).)))))).))))))))))).)))..))))....))))))..
......................................................................................UGCCAAAGGAGAGCUGCCCUG..................    398.566179
......................................................................................UGCCAAAGGAGAGCUGCCCU...................    102.773615
......................................................................................UGCCAAAGGAGAGCUGCCCUU..................    2.16664
```

zma-MIR399_N2

```
Chromosome:    chr5
Start:         150574423
End:           150574547
Strand:        +
Strand bias:   1.0
Abundance bias:1.0
MFE:           -62.70
```

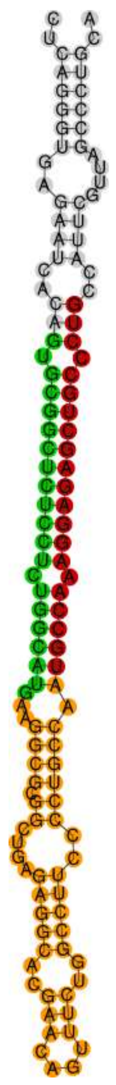

zma-MIR_N50a

```
Chromosome:    chr1
Start:         164971775
End:           164972073
Strand:        +
Strand bias:   0.949
Abundance bias:0.757
MFE:           -78.80
```

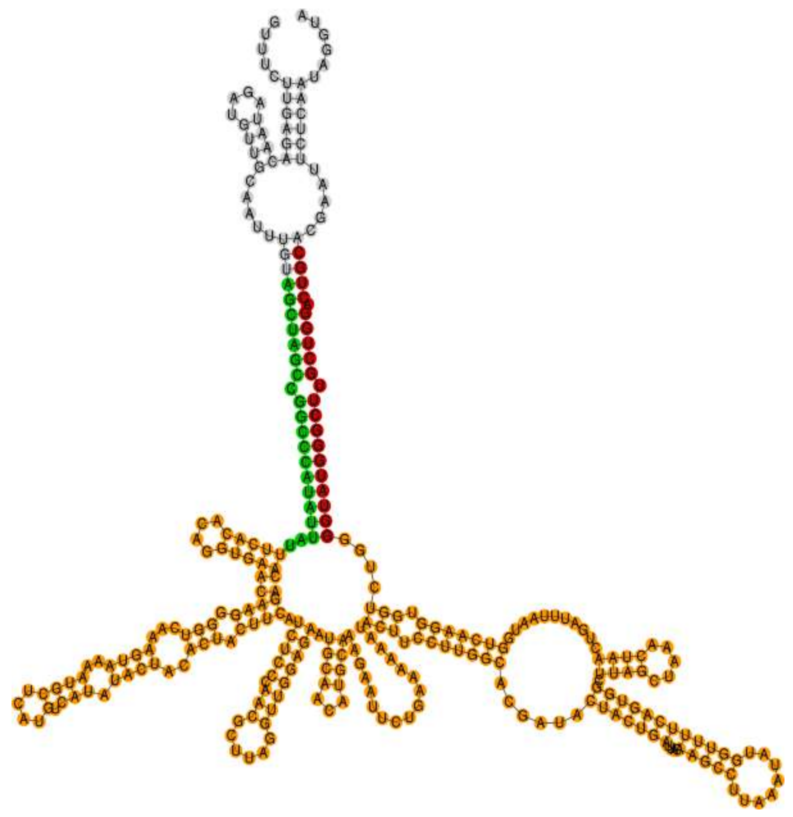

```
                                   Star                                                                                                                                                                                                                                                   Mature
GUUUCUUGAGACAAUAGAUGUUGCAAUUUGUAGCUAGCCGGCCCAUAUUAUUUCACACAGGUGAACACAGAAGGGGUCAAAGUAAAAUGCUCAUGUCAUAUACUACACUACUUCAUCUCCCAACGCUUAGGUUGGAGAAUGCAACAUGCAAAGAAUUCUGAAAAAAUACUUCCUUGGCACGAUACUACUGAUUACAAGCCUUAAAUAUGGUUUUCAGUGGCUUAGCUAAACUAACUGAUUUAAUGGUCAAGGUGGUCUGGGGUAUGGGCUUGCUGGACUGCACGAAUUCUCAAUAGGUA    265.6
.....((((((((((....)))).....((((((((((.((((((((((..(((((....)))))....((((.(((...((((..((((....).))).))))..))).)))).(((((.(((......)))))))).((((...))))................(((.(((((((......(((((((....(((((........)))))))))))).((((.....))))..........))))))).)))....)))))))))).))))).)))))).....))))))......
....................................................................................................................................................................................................................................................................GGUAUGGGCUUGCUGGACUGC..................    125.45193
......................................................................................................................................................................................................................................................................AUGGGCUUGCUGGACUGC..................    0.279384
.....................................................................................................................................................................................................................................................................UAUGGGCUUGCUGGACUGC..................    8.51063
....................................................................................................................................................................................................................................................................GGUAUGGGCUUGCUGGACU....................    52.902791
....................................................................................................................................................................................................................................................................GGUAUGGGCUUGCUGGACUG...................    3.78438
....................................................................................................................................................................................................................................................................GGUAUGGGCUUGCUGGAC......................    1.7525
....................................................................................................................................................................................................................................................................GGUAUGGGCUUGCUGGACG.....................    2.95553
....................................................................................................................................................................................................................................................................GGUAUGGACUUGCUGGACUGC..................    0.17779
....................................................................................................................................................................................................................................................................GGGAUGGGCUUGCUGGACUGC..................    0.660362
...................................................................................................................................................................................................................................................................GGGUAUGGGCUUGCUGGACUG...................    0.228587
...................................................................................................................................................................................................................................................................GGGUAUGGGCUUGCUGGACUGC..................    2.323853
.................................................................................................................................................................................................................................................................UGGGGUAUGGGCUUGCUGGACUGC..................    1.937982
```

Star Mature

```
GCUGCAGGCAAAACCGGCAAGCGAUUUGCAAGGACCUUGUUACUACACCACCAGCUACUGAUCCAUCACCAGGGGAGACGUUGAAGCCAGACUUGGUGGAGAAACAGGUCGGUCGCAAGCGCCGCGCCAAUGUCCGUAUU    156.1
..(((.((((.....(((..(((.(((((...(((((.(((.((...(((((((((((...(((.((....)))))...))...)))......)))))))).))))))))....))))))))...)))..)))).)))..
....................................................................................................GAAACAGGUCGGUCGCAAGCGC..................    12.6897
......................................................................................................AACAGGUCGGUCGUAAGCGCC.................    9.30582
...................................................................................................AGAAACAGGUCGGUCGCAAGCGCC.................    24.03132
..................................................................................................GAGAAACAGGUCGGUCGCAAGCGC..................    53.55627
..................................................................................................GAGAAACAGGUCGGUCGCAAGCGA..................    0.318481
.................................................................................................GGAGAAACAGGUCGGUCGCAAGCG...................    21.682637
.................................................................................................GGAGAAACAGGUCGGUCGCAAGC....................    4.896842
.................................................................................................GGAGAAACAGGUUGGUCGCAAGCG...................    1.569525
```

zma-MIR_N53a

```
Chromosome:    chr1
Start:         236120200
End:           236120339
Strand:        +
Strand bias:   1.0
Abundance bias:0.82
MFE:           -38.60
```

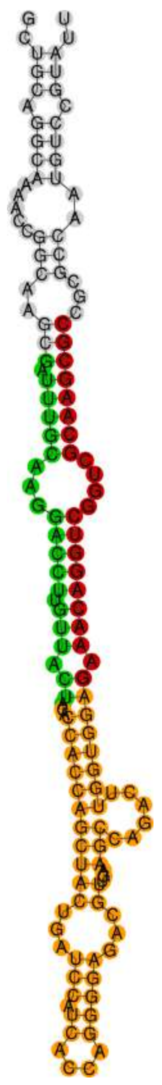

```
                                    Star                                                Mature
AACUUAUGAAGCACCACAUCAUGGAUAUGAACUAGAGCUAAAACAUCUCUUUUGUUUGCUCUGUUGUGCUGAAGACUAUAGCUGGUAAGAUCAACAAGCAGUGAGAUUGUUUUAGGGAAUUCGGCUCUUUAUCGAGU    199.1
.((((((((((..((...((((....)))).......((((((((((((..(((((((.(((.....((((.......)))).....)))....))))))).)))).))))))))).......))..)))))).))))
...................................................................................................CAGUGAGAUUGUUUUAGGGAA..................    187.63441
...................................................................................................CAGUGAGAUUGUUUUAGGGA...................    6.29536
...................................................................................................CAGGGAGAUUGUUUUAGGGAA..................    0.499118
...................................................................................................CAGUGAGAUUGUUUUAGGGAAA.................    0.380978
```

zma-MIR_N56a

```
Chromosome:    chr1
Start:         267235362
End:           267235498
Strand:        +
Strand bias:   0.981
Abundance bias:0.978
MFE:           -28.10
```

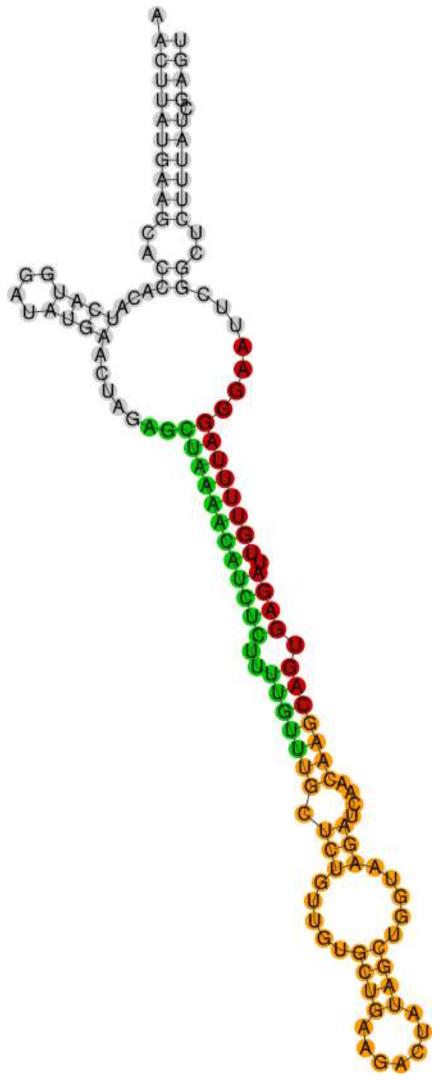

Star Mature

```
AAUGUCAUGGUUGUGGUAGCUUUUGCCACACUGAUGAGCUCCUUUGUUCGCAACCACCUAUAUAGUUUUGUUUGCUCAUCAGACAGGCUAAGCACAAGAACCAUAUUGAUAAG    71.7
..(((((((((.(.((.((((..(((...((((((((.....................................))))))))...))).)))))).).)))))...)))))..
..........................................................................CUCAUCAGACAGGCUAAGCAC..................    56.0622
...........................................................................CAUCAGACAGGCUAAGCACAA.................    0.427815
..........................................................................CUCAUCAGACAGGCUAAGCACA.................    0.799007
..........................................................................CUCAUCAGACAGGCUAAGCA...................    0.499118
..........................................................................CUCAUCAGACAGGCUAAGC....................    0.794114
```

zma-MIR_N58a

```
Chromosome:    chr1
Start:         289638589
End:           289638701
Strand:        +
Strand bias:   0.963
Abundance bias:0.816
MFE:           -32.37
```

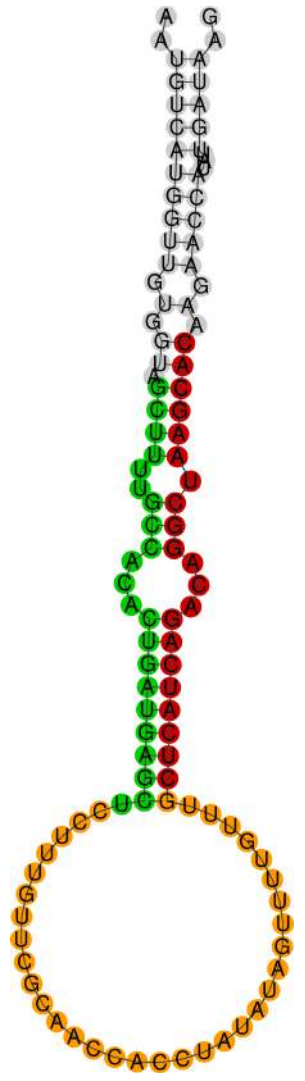

zma-MIR169_N3

Chromosome: chr1
Start: 303789461
End: 303789560
Strand: +
Strand bias: 1.0
Abundance bias: 0.997
MFE: -51.90

```
                 Mature                                           Star
AGGAGGCCCAUCUCUGAUAGCCAAGCAUGAUUUGCCCGUAGUCUGACUCUGAGUGUGCUCCUGCUACGGGCAAAUCAUCUUGGCCAUCUGAGUACCUCCC    2728.5
.(((((.....(((.(((.((((((.((((((((((((((((..(((.(.....).).))..))))))))))))))))))))))).))).))).))))).
.................UAGCCAAGCAUGAUUUGCCCGU.............................................................    1804.757707
.................UAGCCAAGCAUGAUUUGCCCGUAG...........................................................    1.81977
.................UAGCCAAGCAUGAUUUGCCCG..............................................................    727.803179
.................UAGCCAAGCAUGAUUUGCCCGUA............................................................    15.613972
.................UAGCCAAGCAUGAUUUGCCC...............................................................    10.87584
.................UAGCCAAGCAUGAUUUGCCCGA.............................................................    3.565406
.................UAGCCAAGCAUGAUUUGCCCGC.............................................................    15.32806
.................UAGCCAAGCAUGAUUUUCCCG..............................................................    1.5598
.................UAGCCAAGCAUGAUUUGCCCUU.............................................................    2.582895
.................UAGCCAAGCAUGAUUUGCCCGUU............................................................    14.753898
.................UAGCCAAGCCUGAUUUGCCCGU.............................................................    1.5598
.................UAGCCAAGCAUGAUUUGCCCC..............................................................    0.364792
.................UAGCCAAGUAUGAUUUGCCCGU.............................................................    1.5598
.................UAGCCAAGCAUGCUUUGCCCGU.............................................................    1.458206
.................UAGCCAAGCAUGAUUUGCCCA..............................................................    11.481954
.................UAGCCAAGCAUGAUUUGCCCU..............................................................    9.875135
.................UAGCCAAGCAUGAUUUUCCCGU.............................................................    2.59967
.................UAGCCAAGCAUGAUUUGCCUGU.............................................................    1.5598
..................AGCCAAGCAUGAUUUGCCCGU.............................................................    13.789481
..................AGCCAAGCAUGAUUUGCCCG..............................................................    15.2411
..................AGCCAAGCAUGAUUUGCCCGUA............................................................    62.024498
.................................................................ACGGGCAAAUCAUCUUGGCCAUC............    1.13918
```

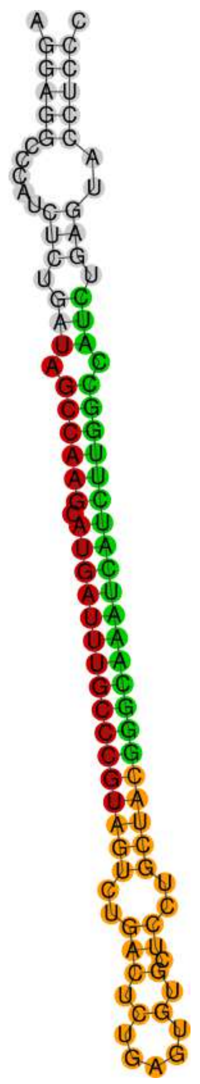

zma-MIR_N12b

Chromosome: chr8
Start: 105980009
End: 105980172
Strand: -
Strand bias: 1.0
Abundance bias: 0.832
MFE: -19.60

```
                        Star                                                                                                                          Mature
UUUGUGAAUAGAUAACACUAUAACAAUCUCUUAUUUUUGUUACAAGAAGUAUAAAGGUAUAAUUCUUUUUUAAUUUUUUUAUUUAGAAAAGUACACAUUACAACAAAUAUAAUACAAAUUAAUUUACAAAAACAAGUAGAUCGUUGCAACUAUCAAUUCAAACU    298.7
....(((((.((((......((((.(((((((.(((((((........((((....(((.....(((((((((.........))))))))).......)))..........))))..........))))))).))).)))).))))....)))).)))))....
..............................................................................................................................CAAAAACAAGUAGAUCGUUGC.................    49.3625
................................................................................................................................AAAACAAGUAGAUCGUUGCAA...............    22.8123
................................................................................................................................AAAACAAGUAGAUCGUUGCAAC..............    18.04873
...............................................................................................................................AAAAACAAGUAGAUCGUUGCA................    22.9553
...............................................................................................................................AAAAACAAGUAGAUCGUUGCAAC..............    2.35716
...............................................................................................................................AAAAACAAGUAGAUCGUUGCAA...............    33.903
..............................................................................................................................CAAAAACAAGUAGAUCGUUGCAAC..............    3.18071
..............................................................................................................................CAAAAACAAGUAGAUCGUUGCA................    42.291
.............................................................................................................................ACAAAAACAAGUAGAUCGUUGC.................    24.6024
.............................................................................................................................ACAAAAACAAGUAGAUCGUUGCAA...............    2.82568
............................................................................................................................UACAAAAACAAGUAGAUCGUUG..................    2.01198
............................................................................................................................UACAAAAACAAGUAGAUCGUUGCA................    1.20813
............................................................................................................................UACAAAAACAAGUAGAUCGUUGC.................    9.69503
...........................................................................................................................UUACAAAAACAAGUAGAUCGU....................    6.06549
...........................................................................................................................UUACAAAAACAAGUAGAUCGUUGC.................    7.19482
```

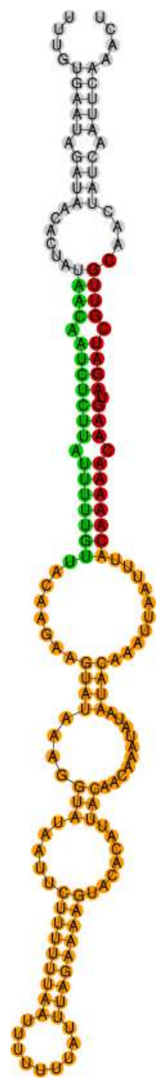

zma-MIR_N12c

```
Chromosome:    chr8
Start:         105986107
End:           105986269
Strand:        -
Strand bias:   1.0
Abundance bias:0.832
MFE:           -19.50
```

```
                    Star                                                                                           Mature
UUUGUGAAUAGAUAACACUAUAACAAUCUCUUAUUUUUGUUACAAGAAGUAUAAAGGUAUAAUUCUUUUUAAUUUUUUUAUUUAGAAAAGUACACAUUACAACAAAUAUAAUACAAAUUAAUUUACAAAAACAAGUAGAUCGUUGCAACUAUCAAUUCAAACU    298.7
....(((((.((((......((((.(((((((.(((((((...(((((.((((....)))).)))))....((((((((....))))))))..............................))))))).))).)))).))))....)))).))))).....
.............................................................................................................................CAAAAACAAGUAGAUCGUUGC.................    49.3625
...............................................................................................................................AAAACAAGUAGAUCGUUGCAA...............    22.8123
...............................................................................................................................AAAACAAGUAGAUCGUUGCAAC..............    18.04873
..............................................................................................................................AAAAACAAGUAGAUCGUUGCA................    22.9553
..............................................................................................................................AAAAACAAGUAGAUCGUUGCAAC..............    2.35716
..............................................................................................................................AAAAACAAGUAGAUCGUUGCAA...............    33.903
.............................................................................................................................CAAAAACAAGUAGAUCGUUGCAAC..............    3.18071
.............................................................................................................................CAAAAACAAGUAGAUCGUUGCA................    42.291
............................................................................................................................ACAAAAACAAGUAGAUCGUUGC.................    24.6024
............................................................................................................................ACAAAAACAAGUAGAUCGUUGCAA...............    2.82568
..........................................................................................................................UACAAAAACAAGUAGAUCGUUG...................    2.01198
..........................................................................................................................UACAAAAACAAGUAGAUCGUUGCA.................    1.20813
..........................................................................................................................UACAAAAACAAGUAGAUCGUUGC..................    9.69503
.........................................................................................................................UUACAAAAACAAGUAGAUCGU.....................    6.06549
.........................................................................................................................UUACAAAAACAAGUAGAUCGUUGC..................    7.19482
```

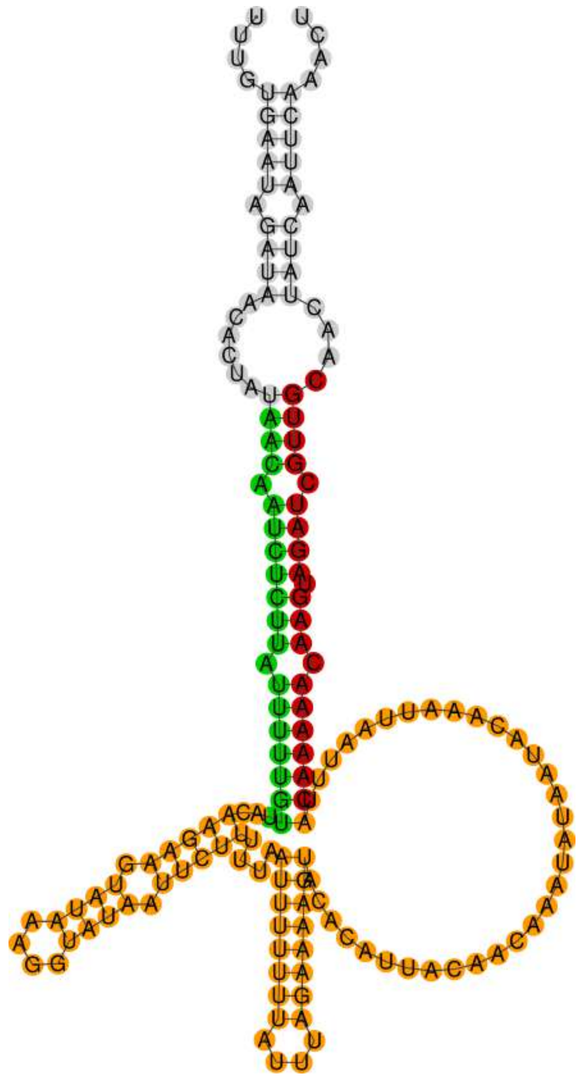

zma-MIR_N12a

```
Chromosome:    chr4
Start:         41808578
End:           41808744
Strand:        +
Strand bias:   1.0
Abundance bias:0.832
MFE:           -20.30
```

```
                                 Star                                                                                                   Mature
UUUUGUGAAUAGAUAACACUAUAACAAUCUCUUAUUUUUGUUACAAGAAGUAUAAAGGUAUAAUUCUUUUUAAUUUUUUUUUAUUUAGAAAAGUACACAUUACAACAAAUAUAAUACAAAUUAAUUUACAAAAACAAGUAGAUCGUUGCAACUAUCAAUUCAAACUG    298.7
.....(((((.((((......((((.(((((((.(((((((...(((((.((((....)))).)))))....((((((((......)))))))).................................))))))).))).)))).))))....)))).))))).....
................................................................................................................................CAAAAACAAGUAGAUCGUUGC..................    49.3625
..................................................................................................................................AAAACAAGUAGAUCGUUGCAA................    22.8123
..................................................................................................................................AAAACAAGUAGAUCGUUGCAAC...............    18.04873
.................................................................................................................................AAAAACAAGUAGAUCGUUGCA.................    22.9553
.................................................................................................................................AAAAACAAGUAGAUCGUUGCAAC...............    2.35716
.................................................................................................................................AAAAACAAGUAGAUCGUUGCAA................    33.903
................................................................................................................................CAAAAACAAGUAGAUCGUUGCAAC...............    3.18071
................................................................................................................................CAAAAACAAGUAGAUCGUUGCA.................    42.291
...............................................................................................................................ACAAAAACAAGUAGAUCGUUGC..................    24.6024
...............................................................................................................................ACAAAAACAAGUAGAUCGUUGCAA................    2.82568
..............................................................................................................................UACAAAAACAAGUAGAUCGUUG...................    2.01198
..............................................................................................................................UACAAAAACAAGUAGAUCGUUGCA.................    1.20813
..............................................................................................................................UACAAAAACAAGUAGAUCGUUGC..................    9.69503
.............................................................................................................................UUACAAAAACAAGUAGAUCGU.....................    6.06549
.............................................................................................................................UUACAAAAACAAGUAGAUCGUUGC..................    7.19482
```

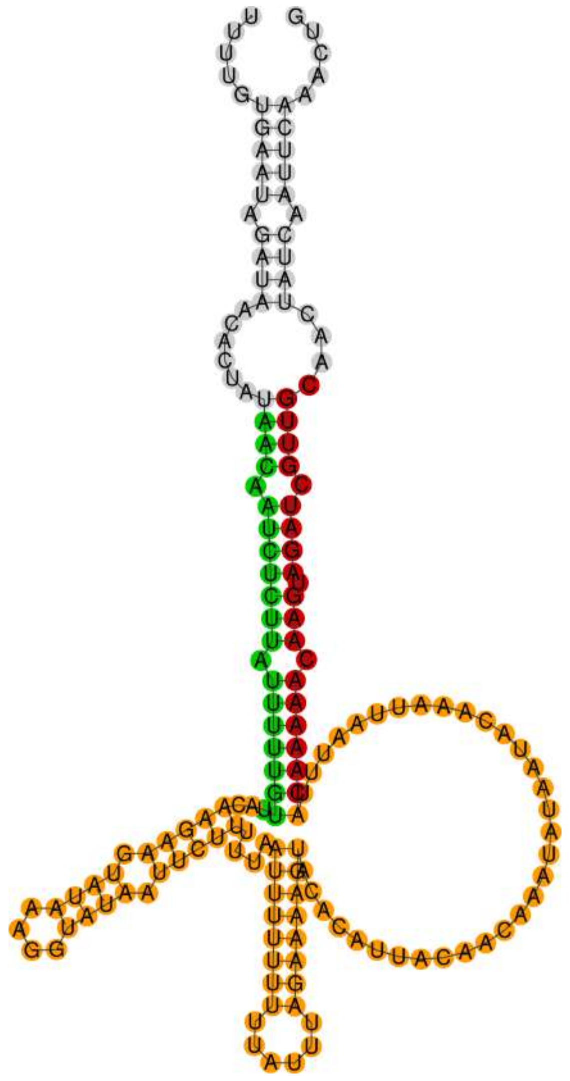

```
                  Mature                                               Star
AUUUAGGGCAUGUUUGGGAGCAAGAGGAUUGGAGGGGCUAAAAUCACCUUGCUAUUCAAAUAGCAAGGUGAUUUUAGCCCCUCCAAUCCCUUUGCUCCCAAACAAGGCCUUAGAUU    199.1
((((((((.(((((((((((((.((((((((((((((((((((((((((((((((....)))))))))))))))))))))))))))))))).))))))))))))))..)))))))).
..................AGCAAGAGGAUUGGAGGGGCU.............................................................................    160.37768
................GGAGCAAGAGGAUUGGAGGGG...............................................................................    0.357231
.................GAGCAAGAGGAUUGGAGGGGC..............................................................................    32.970344
..............................................................................CCCUCCAAUCCCUUUGCUCCC.................    0.152391
```

zma-MIR_N42a

```
Chromosome:    chr1
Start:         4143327
End:           4143442
Strand:        -
Strand bias:   1.0
Abundance bias:0.973
MFE:           -92.20
```

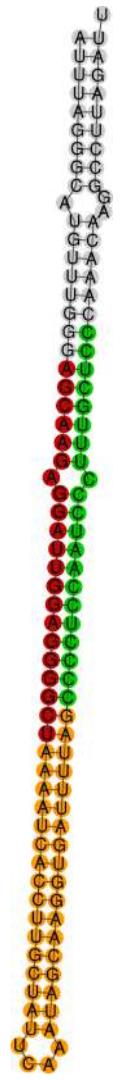

```
                                     Mature                                                              Star
CCUUGAGGCCUUGUUCGGUUGCGUCUGGAUAGAAGGGGAUUAAAUCCCCAUUCUAUUCAAUUUUGACUAGAGAGGGAUUUAAUCCCCUUCGAUCCAGACACAACUGAACAGAAUGGGUGUUCUUA    128.5
....((.((((((((((((((.((((((((.(((((((((((((((((..(((((.(((....))).))))).))))))))))))))))).)))))))).))))))))))....)))).))....
..................UUGCGUCUGGAUAGAAGGGGA......................................................................................    104.469667
.................GUUGCGUCUGGAUAGAAGGGGAUU.....................................................................................    0.942336
..................UUGCGUCUGGAUAGAAGGGGAU.....................................................................................    22.892498
...................UGCGUCUGGAUAGAAGGGGAU.....................................................................................    0.273594
```

zma-MIR_N46a

```
Chromosome:    chr1
Start:         63981221
End:           63981345
Strand:        -
Strand bias:   1.0
Abundance bias:1.0
MFE:           -70.20
```

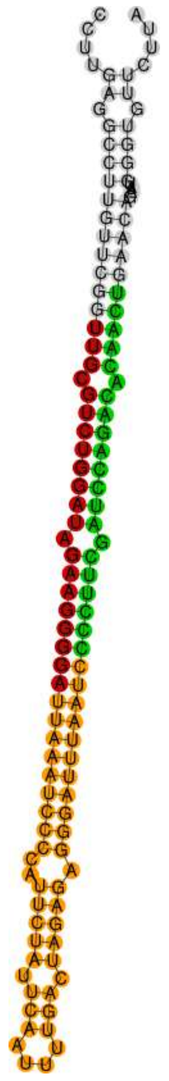

zma-MIR_N47a

```
Chromosome:    chr1
Start:         75696046
End:           75696145
Strand:        -
Strand bias:   1.0
Abundance bias:0.97
MFE:           -26.60
```

```
                          Star                                        Mature
UGUUGGUGCAUGUAAUCGUAUGGCAUCGUUACACACCAUGCAUCUCUGAUCUCUUUGCGCCAGUGUGUGUGACUAUGUCCCUGUAACGAUUGGCUCAGUG    945.6
..((((.((...((((((((((((((.((((((((((.((((.............))))...).))))))))).)))))...)).)))))))))))))..
..............................................................GUGUGUGUGACUAUGUCCCUG.................    61.08885
...............................................................UGUGUGUGACUAUGUCCC...................    14.60522
..............................................................GUGUGUGUGACUAUGUCCC...................    64.95976
..............................................................GUGUGUGUGACUAUGUCCCUGU................    26.6749
..............................................................GUGUGUGUGACUAUGUCCCUGUA...............    42.41207
..............................................................GUGUGUGUGACUAUGUCC....................    509.8306
..............................................................GUGUGUGUGACUAUGUCA....................    11.08127
..............................................................GUGUGUGUGACUAUGUCU....................    17.48617
.............................................................AGUGUGUGUGACUAUGUCCCUGUA...............    7.68354
.............................................................AGUGUGUGUGACUAUGUCC....................    78.76032
.............................................................AGUGUGUGUGACUAUGUCCCUG.................    29.08841
.............................................................AGUGUGUGUGACUAUGUCCC...................    10.2447
.............................................................AGUGUGUGUGACUAUGUCCCUGU................    5.18547
............................................................CAGUGUGUGUGACUAUGUCC....................    15.3671
...........................................................CCAGUGUGUGUGACUAUGUCC....................    8.96413
....................UGGCAUCGUUACACACCA..............................................................    14.0865
```

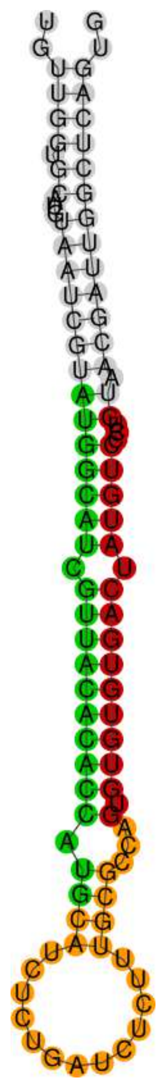

```
                         Mature                                                                                                                      Star
AUAUUCUAAUUAUAAAACUAAUUACAUAGGUUAGGACUAAACGAAAUCUAUUAAACCUAAUUAGUACAUGAUUCGCUCAUAUGACACUAUAGUAAACAUUUGCUAGUCAUGGAUUAAUUAGGUUUAAUAAAUUUUUCUCGUCGUUUAGUCUUCAUCUCUGUAAUUUGUUUUAUAAUUAGGAUAUA   238.7
(((((((((((((((((.(((((((.((((.((((((((((((((.(((((((((((((((((.(((((((.((.......((......))........)).))))))).)))))))))))))))))).))).......)))))))))))).)))).))))))).)))))))))))))))))).
..................UAAUUACAUAGGUUAGGACUA..................................................................................................................................................   160.270477
...................AAUUACAUAGGUUAGGACUA..................................................................................................................................................   0.784328
...................AAUUACAUAGGUUAGGACUAA.................................................................................................................................................   77.582645
```

zma-MIR_N49a

Chromosome: chr1
Start: 126665702
End: 126665886
Strand: -
Strand bias: 1.0
Abundance bias: 0.999
MFE: -71.60

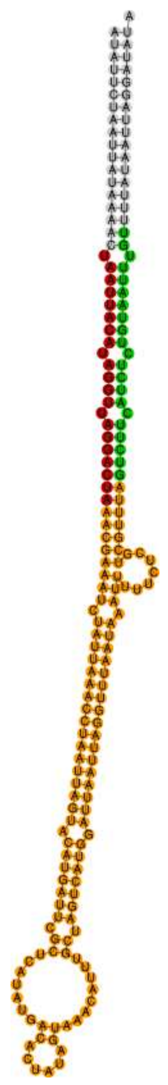

zma-MIR_N52a

Chromosome: chr1
Start: 183203563
End: 183203700
Strand: -
Strand bias: 1.0
Abundance bias:0.863
MFE: -23.40

```
                   Mature                                                                          Star
ACGUGACCCAAAUUAAAUUAAUUUUCUGUAUCUGCAAUGCAUUGUUAUUAUGGUCUGAUCUUUUCCUUUUCCAUUUCUAUUUCUAUAUGUGAUAUAUUAGUGCUGUAGAUAUAGUCAAAUGCAAUUUAUUCUCCACAU    956.9
..(((........(((((.((((.(((((((((((..((((((.(((((((....((........................))....)))))))...)))))))))))))))))..))))..)))))......)))..
..................UAAUUUUCUGUAUCUGCAAUGC..................................................................................................    350.830311
...............AAUUAAUUUUCUGUAUCUGCAAUG...................................................................................................    13.06211
................AUUAAUUUUCUGUAUCUGCAAUGC..................................................................................................    73.800215
................AUUAAUUUUCUGUAUCUGCAAUGU..................................................................................................    6.73939
.................UUAAUUUUCUGUAUCUGCAAUG...................................................................................................    291.976428
.................UUAAUUUUCUGUAUCUGCAAUA...................................................................................................    1.28532
..................UAAUUUUCUGUAUCUGCAAUG...................................................................................................    0.963578
..................UAAUUUUCUGUAUCUGCAAUGA..................................................................................................    2.53144
..................UAAUUUUCUGUAUCUGCAAUGU..................................................................................................    63.737663
...................AAUUUUCUGUAUCUGCAAUGC..................................................................................................    9.65917
..................................................................................................AGUGCUGUAGAUAUAGUCAAAUGC................    1.954496
...................................................................................................GUGCUGUAGAUAUAGUCAAAUGCA...............    0.228587
...................................................................................................GUGCUGUAGAUAUAGUCAAAUGC................    3.33706
......................................................................................................CUGUAGAUAUAGUCAAAUGCAAUU............    1.53572
.......................................................................................................UGUAGAUAUAGUCAAAUGCAAU.............    4.416
```

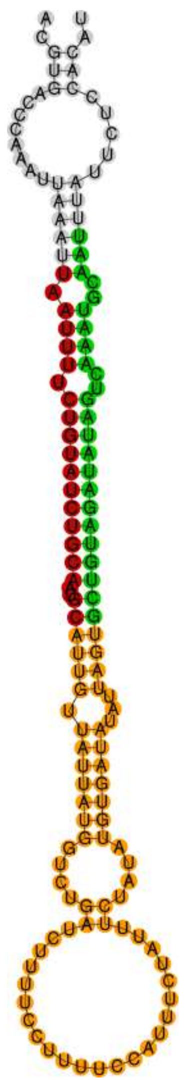

```
                    Star                                                                                                                                                   Mature
UGAACUAUUUUGGGUGUGUCAUCUAGGCGCAUGUACUAUUCUAAGUCACAAGAACCCCCAUUAACUAUUUAAACUUUUUAGGGUGUGUCAUCUAGGUCCAUGCACAACUGACGUGGCGUUCCACCUCAGUCCAACUGGUGACGUACUGACGCGGCCUUGAAUAGUACAUGGACCUAGAUGACACACCCGAAAUAGUUAA    92.4
..((((((((((((((((((((((((..((((((((((((...(((......((((......................))))((((((..((.((((..(....(((((.((((....)))).)))))....)..).))).)).)))))))))...))))))))))))..))))))))))))))))))))))))))..
.................................................................................................................................................................UAGUACAUGGACCUAGAUGAC.................    44.516186
....................................................................................................................................................................UACAUGGACCUAGAUGACACA..............    4.14919
.................................................................................................................................................................UAGUACAUGGACCUAGAUGACA................    25.745797
```

zma-MIR_N57a

```
Chromosome:    chr1
Start:         285664526
End:           285664724
Strand:        -
Strand bias:   1.0
Abundance bias:0.805
MFE:           -89.40
```

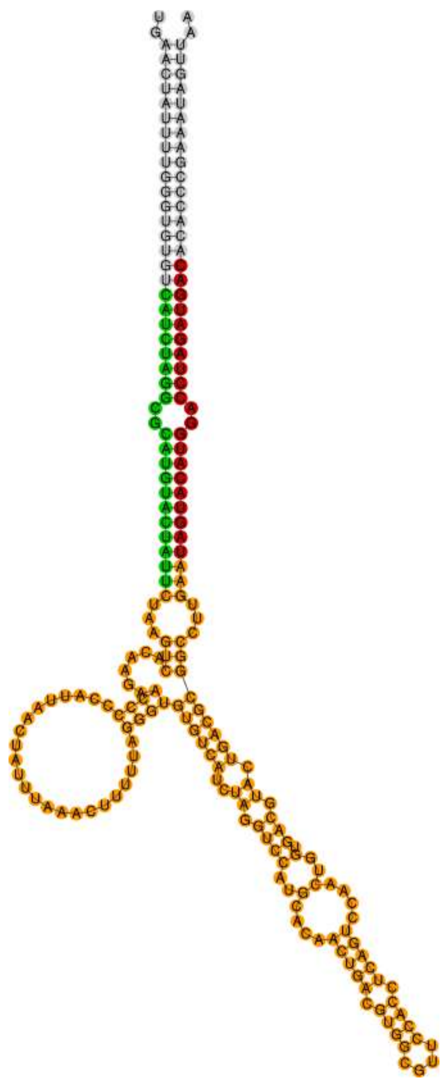

Mature Star

```
GGAUAAUGAAGUAAAAAUAAUUGGAACUGUGGUAGUUCUUUUCAAAGUUUCAAGACAGAAAGGAGUAUAAGGCAACUUUGAAAGGAACGACCACAGUUCCAUUUAUUUUUACUUCAUUAUAUAUCCA    133.5
((((((((((((((((((((.((((((((((((.(((((((((((((((((...((........))....)).))))))))))))))).)))))))))))).)))))))))))))))....))))).
.................UAAUUGGAACUGUGGUAGUUC.........................................................................................    126.101634
................AUAAUUGGAACUGUGGUAGUU..........................................................................................    6.679483
.................UAAUUGGAACUGUGGUAGUUU.........................................................................................    0.754985
```

zma-MIR_N60a

```
Chromosome:    chr2
Start:         2347248
End:           2347374
Strand:        +
Strand bias:   1.0
Abundance bias:1.0
MFE:           -62.80
```

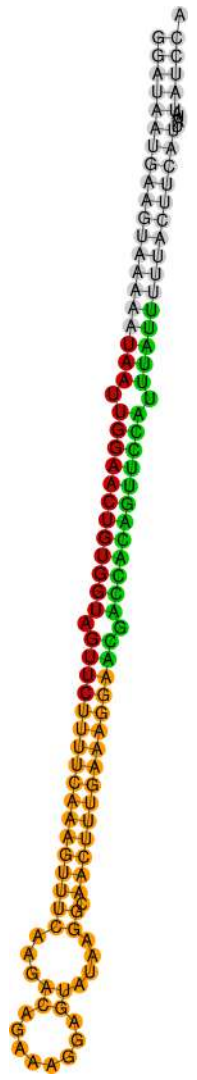

Star   Mature

```
AGAGGGCUGUUUGGUUUGUUGCCUUAGCUGCCACAAUAUGCCUAACUUUUAUGCCUAAGCUUAGUUCUUAAAUUUGAGCGCCUAAGGUUAGGCAAAUUGUGGCACCUUAGGUAGGAAACCAAACAAGCCCGAG    172.2
...(((((((((((((.((((((.((.(((((((((.((((((((((...((....((((((.........))))))))..)))))))))))).))))))))).)).)))))).))))))))).)))))...
...............................................................................................AAUUGUGGCACCUUAGGUAGG..................    141.961233
...................UGCCUUAGCUACCACAAUAUGCCU.............................................................................................    0.371561
```

zma-MIR_N13c

Chromosome: chr1
Start: 117095821
End: 117095953
Strand: +
Strand bias: 1.0
Abundance bias: 0.826
MFE: -68.60

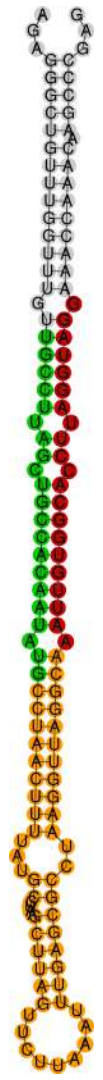

Star Mature

```
ACAGGGCUGUUUGGUUUGUUGCCUUAGCUGCCACAAAAUGCCUAACUUUUAUGCCUAAGCUUAGUUCUUAAAUUUGAGCGCCUAAGGUUAGGCAAAUUGUGGCACCUUAGGUAGGAAACCAAACAAGCCCUAC    171.8
..(((((((((((((((.((((((.((.((((((((..(((((((((((...((....((((((.........))))))))..)))))))))))..)))))))).)).)))))).))))))))).))))))..
..............................................................................................AAUUGUGGCACCUUAGGUAGG..................    141.961233
```

zma-MIR_N13a

```
Chromosome:    chr4
Start:         220897559
End:           220897691
Strand:        +
Strand bias:   1.0
Abundance bias:0.826
MFE:           -67.60
```

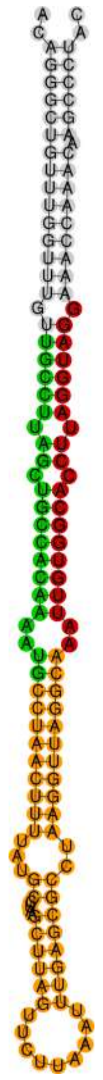

Star Mature

```
AAAGGGCUGUUUGGUUUGUUGCCUUAGCUGCCACAAUAUGCUUAACUUUUAUGCCUAGGCUUAGUUCUUAAAUUUGAGCGCCUAAGGUUAGGCAAAUUGUGGCACCUUAGGUAGGAAACCAAACAAGCCCUAA    171.8
..(((((((((((((((.((((((.((.(((((((((.((((((((((.......(((((...((((........))))))))))))))))))).))))))))).)).)))))).))))))))).))))))..
..............................................................................................AAUUGUGGCACCUUAGGUAGG..................    141.961233
```

zma-MIR_N13b

Chromosome:	chr6
Start:	166875420
End:	166875552
Strand:	+
Strand bias:	1.0
Abundance bias:	0.826
MFE:	-69.80

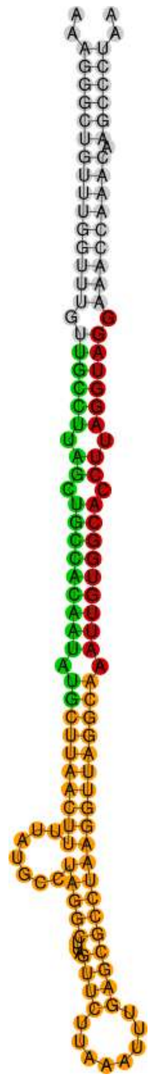

zma-MIR_N69a

Chromosome: chr2
Start: 111713776
End: 111713968
Strand: +
Strand bias: 1.0
Abundance bias:0.872
MFE: -128.80

```
                         Mature                                                                                                                                              Star
CCGGCGUCCGAGUACAAAUAGUGGACGGCGACCCAAGUUCCGACCCAAGUCCACUAUGACUUGUUUAUCAAAGCAAGGAGAGGUGUGAAGCUUCUCCGAGAAGGCGAGGGUCGCACGGAUCUGGCCUGCCGCUGGCCCUAGUUUGACUUGGGUCGGAACUUGGACGCCGUCCACUAUUUGUACUCGGACGCCG    1281.7
.(((((((((((((((((((((((((((((..(((((((((((((((((((.((((.(..((((((....))))))((((((((.....))))))))....((((..(((((.....))))).))))(((...)))).))))..))))))))))))))))))).)))))))))))))))))))))))))))))
..................UAGUGGACGGCGACCCAAGUUC.........................................................................................................................................................    341.081349
...............AAAUAGUGGACGGCGACCCAAG............................................................................................................................................................    4.932954
...............AAAUAGUGGACGGCGACCCAAGUUC.........................................................................................................................................................    0.212795
................AAUAGUGGACGGCGACCCAAGUU..........................................................................................................................................................    52.048435
.................AUAGUGGACGGCGACCCAAGUUC.........................................................................................................................................................    17.418049
.................AUAGUGGACGGCGACCCAAGUU..........................................................................................................................................................    4.950724
..................UAGUGGACGGCGACCCAAGUUCC........................................................................................................................................................    42.629244
..................UAGUGGACGGCGACCCAAGUUU.........................................................................................................................................................    5.5803
...................AGUGGACGGCGACCCAAGUUCCG.......................................................................................................................................................    3.146372
....................GUGGACGGCGACCCAAGUUCCGA......................................................................................................................................................    0.646081
.....................UGGACGGCGACCCAAGUUCCGA......................................................................................................................................................    13.7028
..........................................................................................................................................................GGAACUUGGACGCCGUCCACU..................    3.650176
...............................................................................................................................................................UUGGACGCCGUCCACUAUUUGU............    1.14908
```

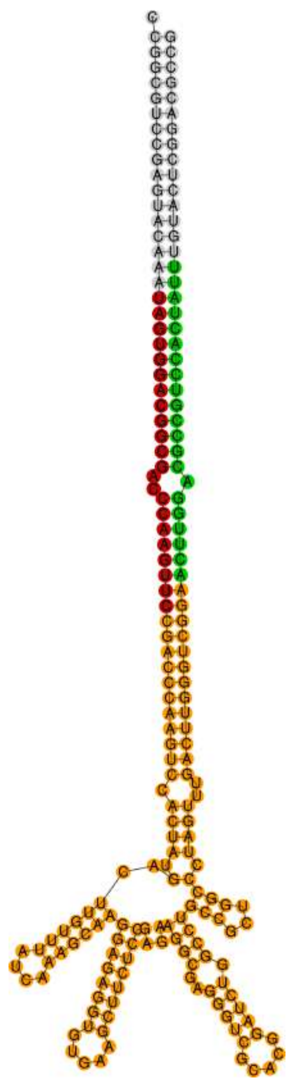

zma-MIR_N70a

Chromosome: chr2
Start: 121349754
End: 121349944
Strand: +
Strand bias: 1.0
Abundance bias: 0.958
MFE: -166.20

```
                 Mature                                                                                                           Star
GAGCUUCCCGCGGCCACAUGUAGAGCGGCGAGCAGGGUGAGGAGCAGCACCGCGGCGGCCGCCCUGCCGGCGCUUCCCAUGGCCACCAAGCCCGGCAGGGCGGCCGCCGCGGUGCUGCUCCUCACCCUGCUCGCCGCUUUACAUGUGGCCGCCCACAACAACAACACCGAGAACCGCAACCCGGGCGGCGG 230.3
.........(((((((((((((((((((((((((((((((((((((((((((((((((((((((((((((.(((.....(((...)))))))))))))))))))))))))))))))))))))))))))))))))))))))))))))))))))))..............(((....((((.......)))))))
.................AUGUAGAGCGGCGAGCAGGGU......................................................................................................................................................... 69.54874
..............CACAUGUAGAGCGGCGAGCAG............................................................................................................................................................ 5.16074
..............CACAUGUAGAGCGGCGAGCAGGGU......................................................................................................................................................... 0.212795
..............CACAUGUAGAGCGGCGAGCAGGG.......................................................................................................................................................... 1.64419
..............CACAUGUAGAGCGGCGAGCAGG........................................................................................................................................................... 1.61614
...............ACAUGUAGAGCGGCGAGCAGG........................................................................................................................................................... 10.64448
...............ACAUGUAGAGCGGCGAGCAGGG.......................................................................................................................................................... 2.6718
...............ACAUGUAGAGCGGCGAGCAGGGUG........................................................................................................................................................ 1.23314
................CAUGUAGAGCGGCGAGCAGGG.......................................................................................................................................................... 68.82181
.................AUGUAGAGCGGCGAGCAGGGC......................................................................................................................................................... 5.32449
..................UGUAGAGCGGCGAGCAGGGUGA....................................................................................................................................................... 2.05523
..................UGUAGAGCGGCGAGCAGGGUG........................................................................................................................................................ 48.79906
..................UGUAGAGCGGCGAGCAGGGUA........................................................................................................................................................ 2.92696
```

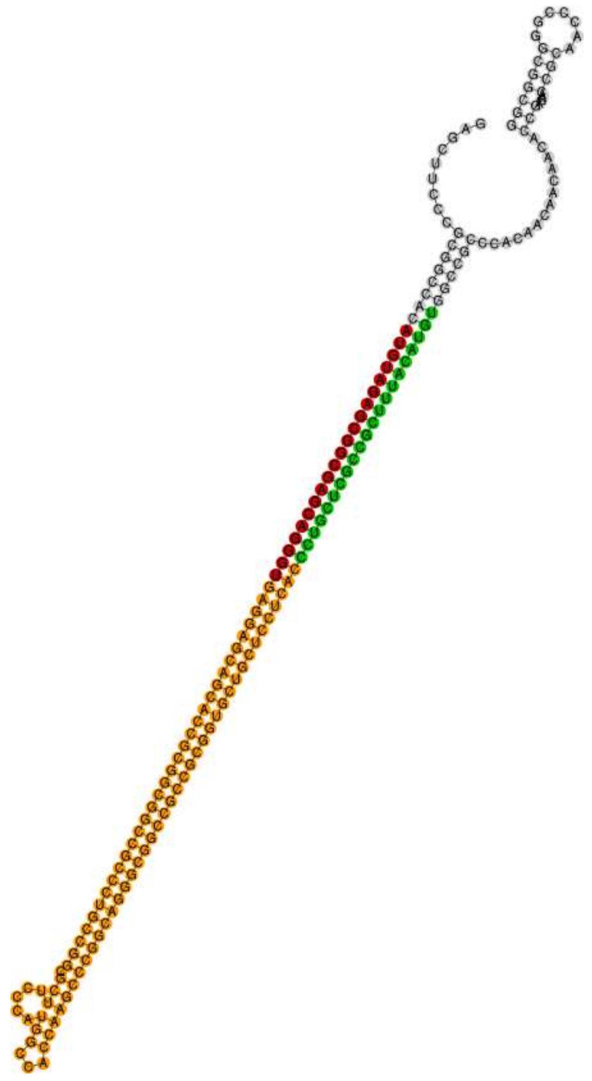

```
                  Mature                                                     Star
AAGGAAAAGCUAUAGUGUGGUUGUUGGGGGCCCUGUAUUGUCGAAAGUUCUCAAAACAUUAUAUUACAAAUCAUUUUUAAGCACGUUUCAGGUGUUCCAAAGCUACCAUAAAAUCAUUUUUUGUUC    42.2
((.((((((......((((((.(((.(((((((((...((((((((((........................))))))..).)))...)))).)))))..))).))))))......)))))).)).
.................UGGUUGUUGGGGGCCCUGUAU........................................................................................    30.3373
.................UGGUUGUUGGGGGCCCUGUA.........................................................................................    1.012915
.................UGGUUGUUGGGGGCCCUGUU.........................................................................................    0.380978
.................UGGUUGUUGGGGGCCCUGUUU........................................................................................    0.279384
```

zma-MIR_N73a

Chromosome: chr2
Start: 141864295
End: 141864420
Strand: +
Strand bias: 0.995
Abundance bias: 0.758
MFE: -22.50

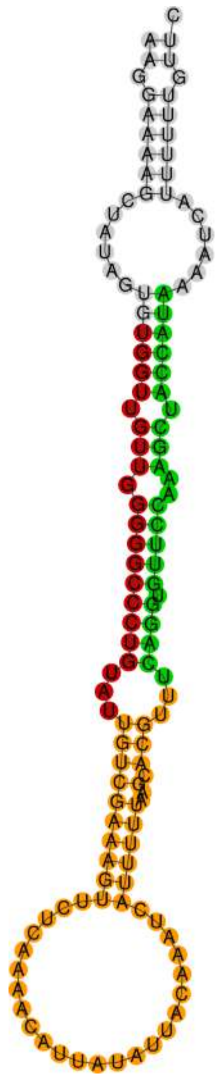

zma-MIR_N75a

Chromosome: chr2
Start: 158761267
End: 158761408
Strand: +
Strand bias: 0.964
Abundance bias: 0.952
MFE: -27.84

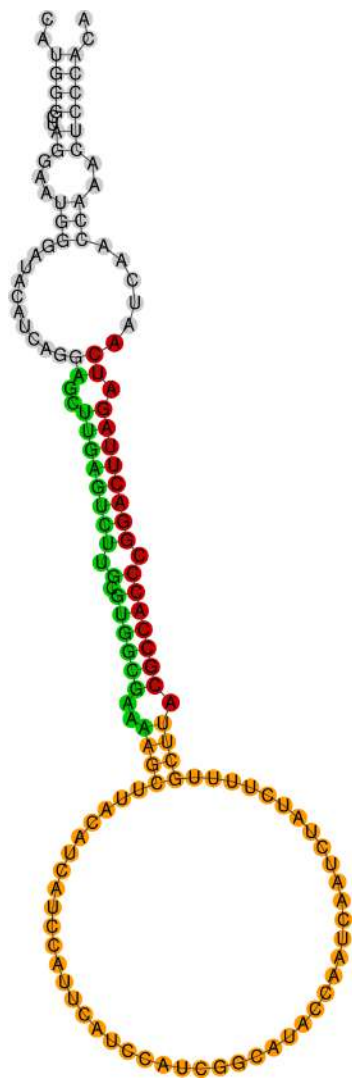

```
                                       Star                                                                     Mature
CAUGGGCUAGGAAUGGGAUACAUCAGGAGCUUGAGUCUUGCGUGGCGAAAAGCUUACAUCAUCCAUUCAUCCAUCGGCAUACCAAUCAAUCUAUCUUUUGCUUACGCCACCCGGACUUAGAUCAAUCAACCAAACUCCCACA    3084.0
..((((..((...(((..........((..((((((((.(.((((((..((((..............................................)))).))))))).)))))))).))......)))..))))))..
.......................................................................................................ACGCCACCCGGACUUAGAUCA..................    1435.036901
..........................................................................................................CCACCCGGACUUAGAUCAAUC...............    0.253986
..........................................................................................................CCACCCGGACUUAGAUCA..................    0.304783
.........................................................................................................GCCACCCGGACUUAGAUC...................    0.507971
........................................................................................................CGCCACCCGGACUUAGAU....................    0.406377
........................................................................................................CGCCACCCGGACUUAGAUCAA.................    15.58285
........................................................................................................CGCCACCCGGACUUAGAUCA..................    2.74677
........................................................................................................CGCCACCCGGACUUAGAUC...................    2.323853
.......................................................................................................ACGCCACCCGGACUUAGAUCAA.................    8.00009
.......................................................................................................ACGCCACCCGGACUUAGAUC...................    1360.760532
.......................................................................................................ACGCCACCCGGACUUAGAUCAAUC...............    54.4159
.......................................................................................................ACGCCACCCGGACUUAGAU....................    16.782398
.......................................................................................................ACGCCACCCGGACUUAGA.....................    11.68215
.......................................................................................................ACGCCACCCCGACUUAGAUC...................    0.330181
.......................................................................................................ACUCCACCCGGACUUAGAUCA..................    0.808793
.......................................................................................................ACGCCACCCGGAAUUAGAUC...................    0.457174
.......................................................................................................ACGCCACCCGGACCUAGAUCA..................    0.203188
.......................................................................................................ACGCCACCCGGACUGAGAUC...................    0.406377
.......................................................................................................ACGCCACCCGGACUUAGACC...................    0.253986
.......................................................................................................ACGCCACCCGGACUUGGAUCA..................    0.152391
.......................................................................................................ACGCCACCCGGACUGAGAUCA..................    0.431775
.......................................................................................................ACGCCACCCAGACUUAGAUC...................    0.152391
.......................................................................................................ACGCCACCCGGCCUUAGAUC...................    0.17779
.......................................................................................................AAGCCACCCGGACUUAGAUCA..................    0.279384
.......................................................................................................ACGCCACCCGUACUUAGAUC...................    0.152391
.......................................................................................................ACGCCACCCGGACUUAGAUA...................    0.457174
.......................................................................................................ACGCCACACGGACUUAGAUCA..................    0.279384
.......................................................................................................ACGUCACCCGGACUUAGAUCA..................    0.17779
.......................................................................................................ACGCCACCCGGACUUAAAUC...................    0.17779
.......................................................................................................ACGCCACCCCGACUUAGAUCA..................    0.152391
.......................................................................................................ACGCCACCCAGACUUAGAUCA..................    0.279384
.......................................................................................................ACGCCACCCGGACUUAGCUC...................    0.228587
.......................................................................................................ACGCCACCCGGGCUUAGAUC...................    0.457174
.......................................................................................................ACGCCACCCGAACUUAGAUC...................    0.253986
.......................................................................................................ACGCCACCCGGACUUAUAUC...................    0.228587
.......................................................................................................ACGCCACCCGGGCUUAGAUCA..................    0.253986
.......................................................................................................ACGCCACCCGGACUUAGAUAA..................    0.304783
.......................................................................................................ACACCACCCGGACUUAGAUCA..................    0.507971
.......................................................................................................ACGCCACCCGGACUUAAAUCA..................    0.228587
.......................................................................................................ACGCCACCCGGACUUAUAUCA..................    0.203188
.......................................................................................................ACGCCACCCGGAUUUAGAUC...................    0.253986
.......................................................................................................ACGCCAUCCGGACUUAGAUCA..................    0.152391
.......................................................................................................ACGCCACCUGGACUUAGAUC...................    0.304783
.......................................................................................................ACGCCACCCGGACUUAGAUU...................    0.660362
.......................................................................................................ACGCCACCCGGACUUAGAUCAU.................    0.228587
.......................................................................................................ACGCCACCCGGACUUAGAUCAC.................    1.179985
.......................................................................................................ACGCCACCCGGCCUUAGAUCA..................    0.152391
.......................................................................................................ACGCCACCCGGACUUAGGUC...................    0.152391
.......................................................................................................ACGCCACCCGGACUUCGAUCA..................    0.17779
.......................................................................................................ACCCCACCCGGACUUAGAUCA..................    0.228587
.......................................................................................................ACGCCACCCGGACUUCGAUC...................    0.203188
.......................................................................................................ACGCCACCCGGACUUAGAUCU..................    1.15948
.......................................................................................................ACGCCACCCGGACUUAGAUCG..................    0.992409
.......................................................................................................ACGCCACCCGGACUUAGAUCC..................    0.482573
.......................................................................................................ACGCCACCCGGACUUACAUC...................    0.203188
.......................................................................................................ACGCCACCCGGACUUAGGUCA..................    0.17779
.......................................................................................................ACGCCACCUGGACUUAGAUCA..................    0.17779
.......................................................................................................ACGCCACUCGGACUUAGAUCA..................    0.17779
.......................................................................................................AUGCCACCCGGACUUAGAUCA..................    0.279384
.......................................................................................................ACGACACCCGGACUUAGAUCA..................    0.203188
.......................................................................................................ACGCCACCCGGACUUAGAUUA..................    0.279384
.......................................................................................................ACGCCACCCGGACUUAGACCA..................    0.152391
......................................................................................................UACGCCACCCGGACUUAGAUCA..................    0.203188
......................................................................................................UACGCCACCCGGACUUAGAUC...................    8.64484
.....................................................................................................UUACGCCACCCGGACUUAGAUC...................    0.507971
.....................................................................................................UUACGCCACCCGGACUUAGAU....................    2.084545
```

zma-MIR_N79a

Chromosome: chr2
Start: 211183948
End: 211184116
Strand: +
Strand bias: 0.995
Abundance bias: 0.948
MFE: -63.80

```
                                  Star                                                                                                          Mature
GGUGGGUUGGGACUAGCCGACUGAGGGUCUGUUCGGUUACAUCGGAACGAGACCAGGAACUGUUCUAGAUGAUCAAUUACUAUAUAGAUUAAACUAUCAUUCCAAGUGGAAUUGUUCCAGAGGUUGAAUCCGAGAUAACCGAACAGACCCUGAGACUAGCAAUGAUAUA    1052.6
(((.(((((....))))).))).(((((((((((((((..(((((....((((.(((((.((((((((((((........................)))))).....)))))).)))))...))))...)))))..))))))))))))))).................. 
..................................................................................................................................CGAGAUAACCGAACAGACCCU..................    978.295
...................................................................................................................................GAGAUAACCGAACAGACCCU..................    0.855631
..................................................................................................................................CGAGAUAACCGAACAGACCC...................    1.485701
..................................................................................................................................CGAGAUAACCGAACAGACCCUGAG...............    0.370641
..................................................................................................................................CGAGAGAACCGAACAGACCCU..................    0.676908
..................................................................................................................................CGAGAUAACCGAACAGACCCG..................    8.8054
..................................................................................................................................CGAGAUAACCGAACAGACCCC..................    2.020938
..................................................................................................................................CGAGAUAACCGAACAUACCCU..................    0.427815
..................................................................................................................................CGAGAUAACCGAACAGACACU..................    0.499118
..................................................................................................................................CGAGAUAACCGAACAGACCAU..................    0.57042
..................................................................................................................................CGAGAUAACCAAACAGACCCU..................    0.228587
.................................................................................................................................CCGAGAUAACCGAACAGACCC...................    0.965145
.................................................................................................................................CCGAGAUAACCGAACAGACCCU..................    0.85959
.......................AGGGUCUGUUCGGUUACAUCGGAA..........................................................................................................................    1.094724
.......................AGGGUCUGUUCGGUUACAACGGAA..........................................................................................................................    0.319396
......................GAGGGUCUGUUCGGUUACAUCGGA...........................................................................................................................    0.408264
```

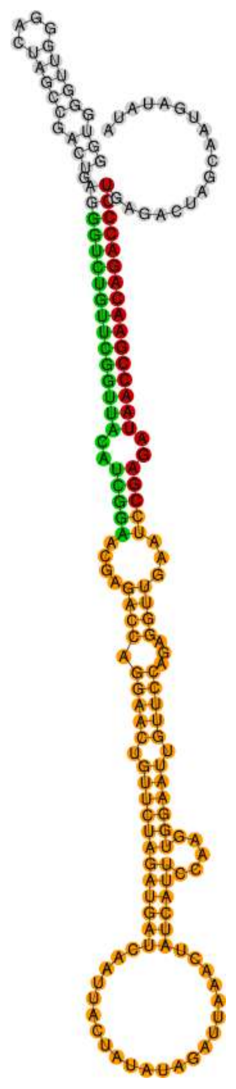

```
                 Mature                            Star
UAUAGAUGUACUUUGUUAUUUACUCUGUACAUUUUGGUAGUACAAAAAUGUGUGCAGUAAAUAGCGACAAGAAUGUGCAGAGUAAAUAACGAAGUACAUCUAUAA    122.6
(((((((((((((((((((((((((((((((((((.((.(((((......))))).((.....)).)).))))))))))))))))))))))))))))))))))).
.................AUUUACUCUGUACAUUUUGGU...................................................................    82.11751
...............UUAUUUACUCUGUACAUUUUGGUA..................................................................    0.580206
.................AUUUACUCUGUACAUUUU......................................................................    5.2812
.................AUUUACUCUGUACAUUUUGGUAGU................................................................    6.196615
.................AUUUACUCUGUACAUUUUGGUA.................................................................    19.31073
```

zma-MIR_N81a

Chromosome: chr2
Start: 220575213
End: 220575317
Strand: +
Strand bias: 1.0
Abundance bias: 0.925
MFE: -52.20

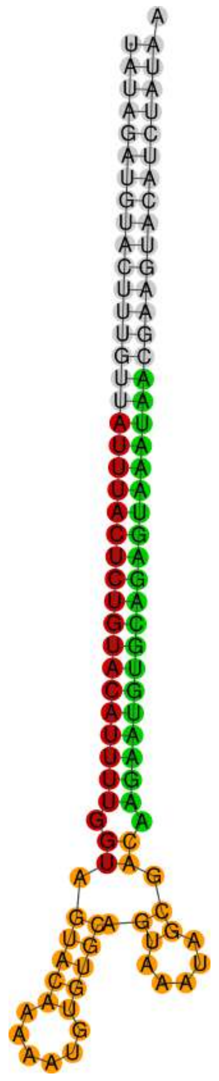

zma-MIR_N16a

```
Chromosome:    chr1
Start:         199074233
End:           199074352
Strand:        +
Strand bias:   1.0
Abundance bias:1.0
MFE:           -57.90
```

```
                                         Star                                                                        Mature
GAAAGAGUAGAAGGGUAAAAUAUCACAUUGAGAUCACAAAUAAACUAAAAUAAGCAACCAGUACUAACUUAUUUCAGUUUAUUUGUGGUCUCAAAGUGAUAUUUUACUCUCCUACCCUUU    538.5
.((((.((((.(((((((((((((((.(((((((((((((((((((.(((((((.............))))))).))))))))))))))))))).))))))))))))))).)))).))))
.................................................................................UUUGUGGUCUCAAAGUGAUAU..................    458.370216
.................................................................................UUUGUGGUCUCAAAGUGAUA...................    8.901715
.................................................................................UUUGUGGUCUCAAAGUGAUAUU.................    32.422154
.................................................................................UUUGUGGUCUCAAAGUGAUAUG.................    1.45641
................................................................................AUUUGUGGUCUCAAAGUGAUA...................    19.834531
...............................................................................UAUUUGUGGUCUCAAAGUGAU....................    17.603827
```

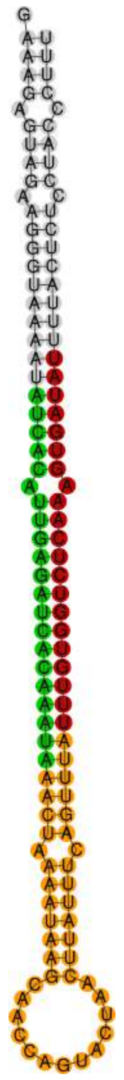

```
                                           Star                                                 Mature
GAAGGGUAAGAGGGUAACAUAUCACUUUGUGAUUACAAAUAAGCUAUCUAAUGUUGGCUUAUUUCAGCUUAUUUGUGGUCUCAAAGUGAUAUUUUAACUUCAUAUCAUUU    538.5
....((((.((((.(((.(((((((((((.(((((((((((((((....((((......))))..))))))))))))))))))).))))))))))).))).)))).))))....
.......................................................................UUUGUGGUCUCAAAGUGAUAU..................    458.370216
.......................................................................UUUGUGGUCUCAAAGUGAUA...................    8.901715
.......................................................................UUUGUGGUCUCAAAGUGAUAUU.................    32.422154
.......................................................................UUUGUGGUCUCAAAGUGAUAUG.................    1.45641
......................................................................AUUUGUGGUCUCAAAGUGAUA...................    19.834531
.....................................................................UAUUUGUGGUCUCAAAGUGAU....................    17.603827
```

zma-MIR_N16c

```
Chromosome:    chr2
Start:         2696576
End:           2696685
Strand:        +
Strand bias:   1.0
Abundance bias:1.0
MFE:           -41.80
```

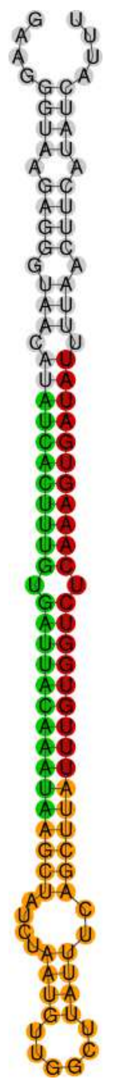

Star Mature

```
UGCUCCCUACAUUCCAAAUUAUAAGUCAUUCUCGCUUUUCUUGAUACAUAAUCUAGAUAUACUGUAAAGGUUGUGUAUCUAGAAAAAGCCAGAACGAUUUAUGAUUUCGAACGGAGGGAGU    353.1
.(((((((.(.(((.((((((((((((.((((.((((((((.((((((((((((..(((...)))..)))))))))))).)).)))))).)))).))))))))))))).))).).)))))))
..................................................................................AAAAAGCCAGAACGAUUUAUGA.................    343.717364
..................................................................................AAAAAGCCAGAACGCUUUAUGA.................    0.883101
..................................................................................AAAAAGCCAGACCGAUUUAUGA.................    0.703221
....................AUAAGUCAUUCUCGCUUUUCU................................................................................    7.838643
```

zma-MIR_N61a

```
Chromosome:    chr2
Start:         16789325
End:           16789445
Strand:        -
Strand bias:   1.0
Abundance bias:1.0
MFE:           -50.80
```

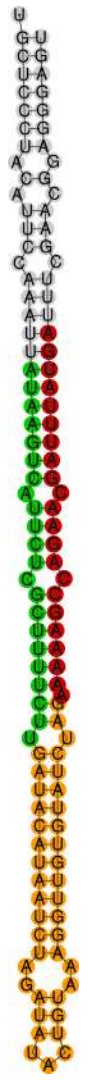

```
                         Mature                                                                                                                  Star
CAUAAGUCUAUCAAAUGCUUUGAAUGUGGAAAAAAUGUCAUUUUGCCCAUAACUGGAAAGUCAAACCACCAACUUGAGUGCUUUGGCAAGGGAGUAGGUGGUUUGACUUUGCAAUUGUGGACAAAACGACCUUUUUUCCACACUCAAAGCAUUUGAUAGGCUUAUGA    76.4
((((((((((((((((((((((.(((((((((((.(((.(((((.((((((.((.((((((((((((((.((((...(((....)))...)))).))))))))))))))).)).)))))).))))).))).))))))))))).))))))))))))))))))))))))).
..................UUUGAAUGUGGAAAAAAUGUCA.......................................................................................................................    48.692114
..................UUUGAAUGUGGAAAAAAUGUCAU......................................................................................................................    2.958426
...................UUGAAUGUGGAAAAAAUGUCAU......................................................................................................................    4.173204
....................UGAAUGUGGAAAAAAUGUCAUU.....................................................................................................................    10.48011
```

zma-MIR_N64a

Chromosome: chr2
Start: 64344194
End: 64344360
Strand: -
Strand bias: 1.0
Abundance bias: 0.867
MFE: -100.00

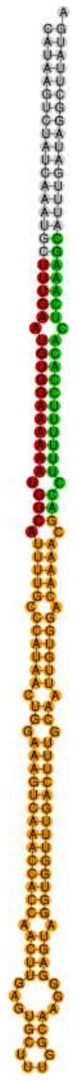

```
                  Mature                                                                          Star
CGGCCACAUGUAAAGCGGCGAGCAGGGUGAGGAGCAGCACCGCGGCGGCCGCCCUGCCGGGCUUGGUGGCCAUGGGAAGCGCCGGCAGGGCGGCCGCCGCGGUGCUGCUCCUCACCCUGCUCGCCGCUCUACAUGUGGCCGC    20.9
(((((((((((.((((((((((((((((((((((((((((((((((((((((((((((((((((............)))).))))))))))))))))))))))))))))))))))))))))))))))).)))))))))))).
..................CGAGCAGGGUGAGGAGCAGCA.......................................................................................................    11.5093
...............CGGCGAGCAGGGUGAGGAGCA..........................................................................................................    6.78227
................GGCGAGCAGGGUGAGGAGCAG.........................................................................................................    1.43866
```

zma-MIR_N71a

```
Chromosome:     chr2
Start:          121349763
End:            121349904
Strand:         -
Strand bias:    1.0
Abundance bias:0.941
MFE:            -151.90
```

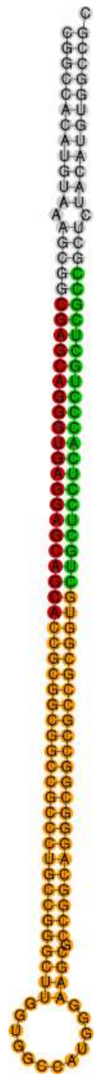

zma-MIR_N72a

```
Chromosome:    chr2
Start:         132233843
End:           132234057
Strand:        -
Strand bias:   1.0
Abundance bias:0.992
MFE:           -101.30
```

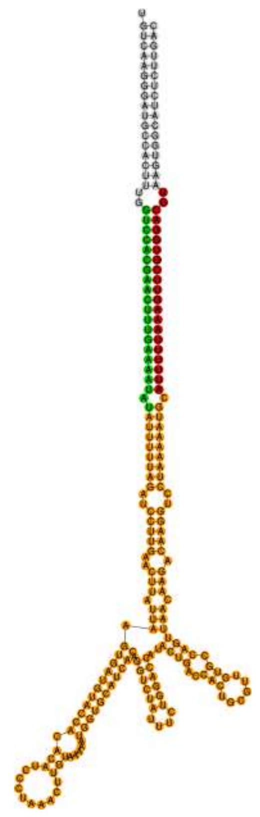

```
                    Star                                                                                                                                                                       Mature
UGUCAAGGGAUGCCACUUUGGUCCACGAACUUUGAAAAUAUAUUUUUAGAUCCUUGAACUUAUUAAGUGAUGUACCACACAUCCCUAAACUUGUAAAAAUGGUGCAUCACAGGUCUAUUUCUGGACCAUACUGACCACUGCGUUGUGCCAGUUAACAAGACAAGGUCCUAAAAAUGCAUUUUUAAAGUUCGUGGACGUAAGUGGCAUCUCUUGAC    491.7
.((((((((((((((((..((((((((((((((((((.(((((((((..(((((..(((.(((.((((((((((.(((..........))).....))))))))))).((((((....))))))..((((..(((......))).))))))).))).)))))..))))))))).)))))))))))))))))))..))))))))))))))))
.................................................................................................................................................................................AUUUUUAAAGUUCGUGGACGU.................    437.041877
.................................................................................................................................................................................AUUUUUAAAGUUCGUGGACGC.................    38.375726
.................................................................................................................................................................................AUUUUUAAAGUUCGUGGACGA.................    2.42074
..................UGGUCCACGAACUUUGAAAAU..........................................................................................................................................................................    9.880981
```

zma-MIR_N74a

Chromosome: chr2
Start: 152869331
End: 152869510
Strand: -
Strand bias: 1.0
Abundance bias: 0.789
MFE: -47.80

```
Star                                                                                                            Mature
AUAGGGGCUAAGUGUAAAUUAUGUACACUUUUGGACUAAAGUGCAAAAACCAAGGUGUGAGGGUACCACAAGUCAUAUGGUUCACAUAUUUGAAGAUUUGCAAGUCAAAAGAUGUGAAAUUUCAAUUGCGCUUAUAUUCAUCACUGUAGGACUUGGGUUCAAGCUUUCCCCAGCCCCUUU   493.4
..(((((((.((((((.......))))))((((.((....)).)))).....((.(.(((((((((.((((((.(((((((((((.(((((...........)))))..)))))).......................)))))).)))))))))....))))))))))))))))..
.............................................................................................................................................CACUGUAGGACUUGGGUUCAAG.................   163.514587
...............................................................................................................................................CUGUAGGACUUGGGUUCAAG.................   6.579509
.............................................................................................................................................CACUGUAGGACUUGGGUUCAAGCU...............   27.535876
.............................................................................................................................................CACUGUAGGACUUGGGUUCA...................   2.01957
.............................................................................................................................................CACUGUAGGACUUGGGUUCAAGC................   2.01957
.............................................................................................................................................CACUGUAGGACUUGGGUUC....................   5.161361
............................................................................................................................................UCACUGUAGGACUUGGGUUCAAG.................   0.371561
...........................................................................................................................................AUCACUGUAGGACUUGGGUUCAAGC................   14.3817
...........................................................................................................................................AUCACUGUAGGACUUGGGUUCAAGCU...............   0.626207
...........................................................................................................................................AUCACUGUAGGACUUGGGUUCAAG.................   9.671958
..........................................................................................................................................CAUCACUGUAGGACUUGGGUUCA...................   4.99461
..........................................................................................................................................CAUCACUGUAGGACUUGGGUUC....................   0.626207
..........................................................................................................................................CAUCACUGUAGGACUUGGGUUCAAG.................   151.60235
```

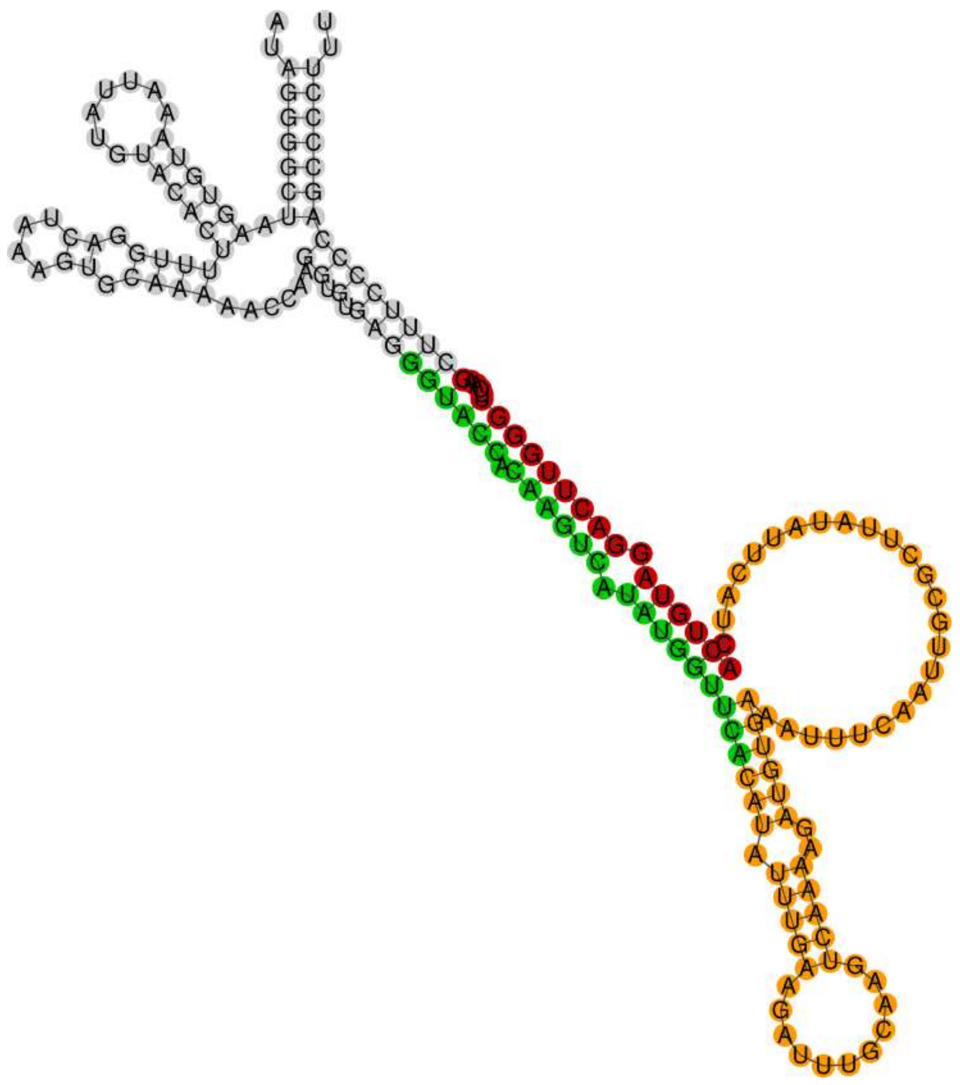

zma-MIR_N10b

Chromosome: chr2
Start: 79796889
End: 79797006
Strand: +
Strand bias: 1.0
Abundance bias: 1.0
MFE: -89.60

```
                 Mature                                                     Star
AGUUUGGGAGCAAGGGAAUUGGAGGGGAUUGAGGAGGCUAAAAUCCCUGGAUUGAGGAGGCUAAAAUCCCUGGAUUUUUAGCCUCCUCAAUCCCCUCCAAUUCCCUUGCUCCAAACUA    2648.0
(((((((.((((((((((((((((((((((((((((((((((((((..(((((.((....))..)))))..))).)))))))))))))))))))))))))))))))))))))))))))).
.................AUUGGAGGGGAUUGAGGAGGCU...............................................................................    2612.77163
.................AUUGGAGGGGAUUGAGGAGGCUA..............................................................................    5.89913
.................AGUGGAGGGGAUUGAGGAGGCU...............................................................................    2.61104
.................AUUGGAGGGGCUUGAGGAGGCU...............................................................................    0.956615
.................AUUGGAGGGGAUUGAGGAGGCUU..............................................................................    1.59436
.................AUUGGAGGGGAUUGAGGAGGCC...............................................................................    20.991323
.................AUUGGAGGGGAUUGAGGCGGCU...............................................................................    2.2321
.................AUUGGAGGGGAUUGGGGAGGCU...............................................................................    0.956615
```

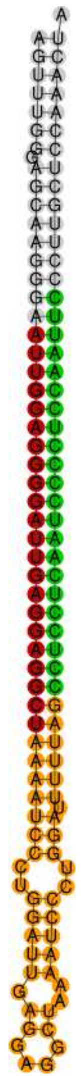

zma-MIR_N10a

```
Chromosome:    chr2
Start:         79796886
End:           79797007
Strand:        -
Strand bias:   1.0
Abundance bias:1.0
MFE:           -92.40
```

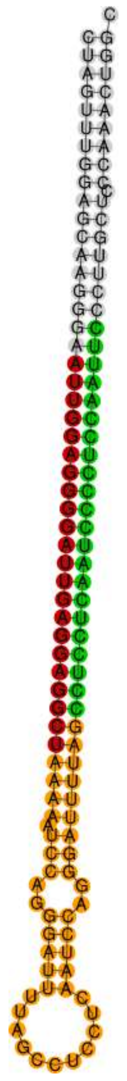

```
                  Mature                                                    Star
CUAGUUUGGAGCAAGGGAAUUGGAGGGGAUUGAGGAGGCUAAAAAUCCAGGGAUUUUAGCCUCCUCAAUCCAGGGAUUUUAGCCUCCUCAAUCCCCUCCAAUUCCCUUGCUCCCAAACUGGC    2648.0
((((((((((((((((((((((((((((((((((((((((((((.(((..(((((...........)))))..)))))))))))))))))))))))))))))))))))))))).))))))))).
..................AUUGGAGGGGAUUGAGGAGGCU..................................................................................    2612.77163
..................AUUGGAGGGGAUUGAGGAGGCUA.................................................................................    5.89913
..................AGUGGAGGGGAUUGAGGAGGCU..................................................................................    2.61104
..................AUUGGAGGGGCUUGAGGAGGCU..................................................................................    0.956615
..................AUUGGAGGGGAUUGAGGAGGCUU.................................................................................    1.59436
..................AUUGGAGGGGAUUGAGGAGGCC..................................................................................    20.991323
..................AUUGGAGGGGAUUGAGGCGGCU..................................................................................    2.2321
..................AUUGGAGGGGAUUGGGGAGGCU..................................................................................    0.956615
```

Mature Star

```
UUGUUUGGGAGACAGGGGAUUGGAGGGGAUUGAGGAGGCUAGAAUUCCUUACUGUUUAAUUUUGCAUAGCAAGGGAUUCUAGCCCCUCAAUCCCCUCUAAUCCCCUUACUCCCAAACAAG    2710.1
(((((((((((..((((((((((((((((((((((.((((((((((((((.((((.((....)).)))).))))))))))))))))))))))))))))))))))))..))))))))))).
..................AUUGGAGGGGAUUGAGGAGGCU................................................................................    2612.77163
..................AUUGGAGGGGAUUGAGGAGGCUA...............................................................................    5.89913
..................AGUGGAGGGGAUUGAGGAGGCU................................................................................    2.61104
..................AUUGGAGGGGCUUGAGGAGGCU................................................................................    0.956615
..................AUUGGAGGGGAUUGAGGAGGCUU...............................................................................    1.59436
..................AUUGGAGGGGAUUGAGGAGGCC................................................................................    20.991323
..................AUUGGAGGGGAUUGAGGCGGCU................................................................................    2.2321
..................AUUGGAGGGGAUUGGGGAGGCU................................................................................    0.956615
....................UGGAGGGGAUUGAGGAGGCUAG..............................................................................    3.02928
```

zma-MIR_N10c

```
Chromosome:    chr3
Start:         182293222
End:           182293341
Strand:        -
Strand bias:   1.0
Abundance bias:0.978
MFE:           -89.10
```

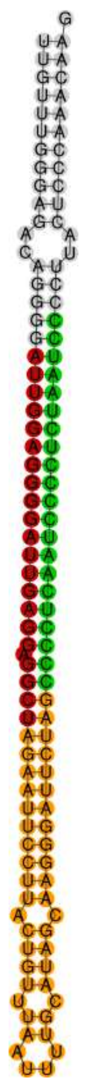

```
                                         Star                                                                              Mature
GGUGGCUAUUGAGUUGCAACAAAGCAUGUGAAUAUUGGCAUCGAACUAUCGGUUGAUAUAGAGUUAUACAUACACCACUAACUCAAACUCUUAACAUCAAUAAUGCCAAUGUUUGACUGCUAUGAGGACUUAUAGGAAUU    264.
.....((((.(((((....((.((((.(((((((((((((((((....)))((((((..((((((...................))))))....)))))).))))))))))))).))))))).))..))))))))).....
......................................................................................................AUGCCAAUGUUUGACUGCUAU.................    240.25559
.......................................................................................................UGCCAAUGUUUGACUGCUAU.................    0.406377
......................................................................................................AUGCCAAUGUUUGACUGCU...................    3.10818
......................................................................................................AUGCCAAUGUUUGACUGCUA..................    0.784328
......................................................................................................AUUCCAAUGUUUGACUGCUAU.................    0.279384
......................................................................................................AUGCCAAUGUUUGACUGCUAUU................    0.855631
.....................................................................................................AAUGCCAAUGUUUGACUGCU...................    0.203188
.....................................................................................................AAUGCCAAUGUUUGACUGCUAU.................    0.854698
.....................................................................................................AAUGCCAAUGUUUGACUGCUA..................    1.21214
....................................................................................................UAAUGCCAAUGUUUGACUGCU...................    1.14293
...................................................................................................AUAAUGCCAAUGUUUGACUGC....................    0.203188
...................................................................................................AUAAUGCCAAUGUUUGACUGCUAU.................    2.359038
```

zma-MIR_N76a

```
Chromosome:    chr2
Start:         163743815
End:           163743954
Strand:        -
Strand bias:   0.999
Abundance bias:0.953
MFE:           -33.30
```

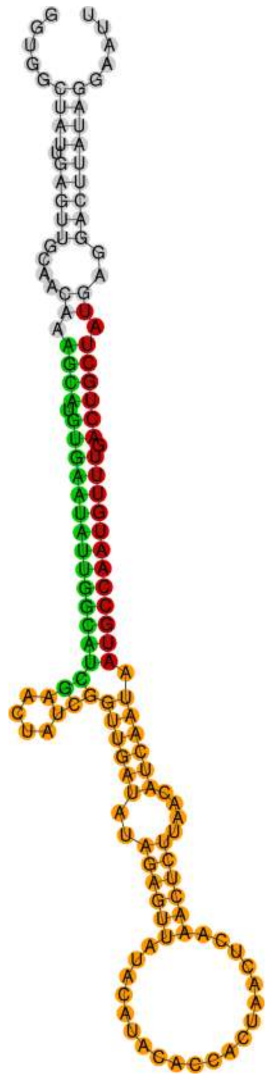

zma-MIR_N80a

Chromosome: chr2
Start: 219978116
End: 219978237
Strand: -
Strand bias: 1.0
Abundance bias: 0.948
MFE: -59.30

```
                                   Star                                                                             Mature
UUAGGGCUAGUUUAAAAACUUAGAUCUUCUUCGGGGUUUCCGGAGAUUGAAUGAAAAAUUAACUUUUUUCUCUCAAUCUCCGGAAAUACCGGAGUAGAUUUAAGUUUCCAAAAGAGCUUCCA    142.1
...(((((..(((..((((((((((((.((((((.(((((((((((((((..((((((......))))))..))))))))))))))).)))))).))))))))))))..)))..)))))...
...................................................................................AAUACCGGAGUAGAUUUAAGU.................    70.761375
....................................................................................AUACCGGAGUAGAUUUAAGUUUC.............    0.754013
...................................................................................AAUACCGGAGUAGAUUUAAGUUUC.............    2.690795
...................................................................................AAUACCGGAGUAGAUUUAAGC.................    1.81185
..................................................................................AAAUACCGGAGUAGAUUUAAGUUUC.............    0.432414
..................................................................................AAAUACCGGAGUAGAUUUAAGU.................    3.065334
..................................................................................AAAUACCGGAGUAGAUUUAAGUUU...............    6.669461
..................................................................................AAAUACCGGAGUAGAUUUAAG..................    2.64771
.................................................................................GAAAUACCGGAGUAGAUUUAAGUU................    0.319396
................................................................................GGAAAUACCGGAGUAGAUUUAAGU.................    1.237712
......................GAUCUUCUUCGGGGUUUCCGG.............................................................................    44.412964
```

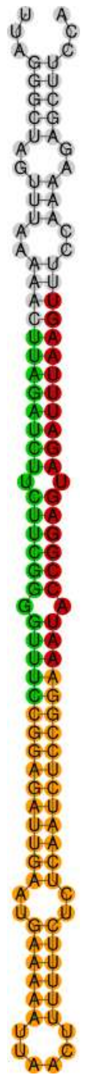

zma-MIR_N88a

Chromosome: chr3
Start: 41384359
End: 41384504
Strand: +
Strand bias: 1.0
Abundance bias: 0.774
MFE: -111.40

```
                 Mature                                                                   Star
UGCGAGCUGUUCUGGUAUUGGUCCGGAUGAGGCUAAGCUUACCCAUGAUGAGACAUGUGGCCAGUUAAAUCAUGGGUAUGCUUAGCCACAUGCCUCAUCAUGGGUAAGCUUAGCCUCGGCCGAACCAAUACCAGAACAGCUCGCAC    776.8
((((((((((((((((((((((.(((.(((((((((((((((((((((((((.((((((((.(((..............)))..)))))))).))))))))))))))))))))))))))))).))).)))))))))))))))))))))).
.................UUGGUCCGGAUGAGGCUAAGCU...........................................................................................................    400.78972
..............GUAUUGGUCCGGAUGAGGCUAAGC............................................................................................................    11.45997
...............UAUUGGUCCGGAUGAGGCUAA..............................................................................................................    5.47566
...............UAUUGGUCCGGAUGAGGCUAAGCU...........................................................................................................    14.01126
...............UAUUGGUCCGGAUGAGGCUAAG.............................................................................................................    32.411479
................AUUGGUCCGGAUGAGGCUAAGCU...........................................................................................................    1.490572
................AUUGGUCCGGAUGAGGCUAAGC............................................................................................................    55.110817
................AUUGGUCCGGAUGAGGCUAAGCUU..........................................................................................................    6.801013
.................UUGGUCCGGAUGAGGCUAAG.............................................................................................................    2.343018
.................UUGGUCCGGAUGAGGCUAAGC............................................................................................................    16.33786
.................UUGGUCCGGAUGAGGCUAAGCUU..........................................................................................................    3.11462
..................UGGUCCGGAUGAGGCUAAGCU...........................................................................................................    3.623716
..................UGGUCCGGAUGAGGCUAAGCUU..........................................................................................................    48.412872
```

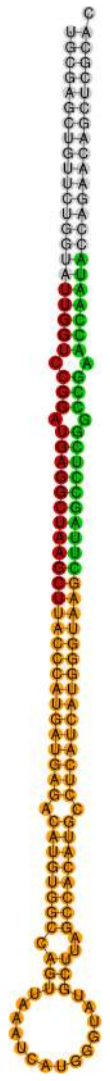

zma-MIR_N102a

```
Chromosome:    chr3
Start:         220051976
End:           220052177
Strand:        +
Strand bias:   0.948
Abundance bias:0.96
MFE:           -157.40
```

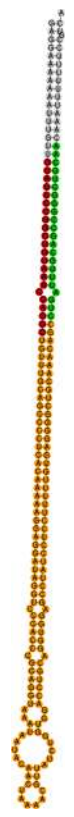

```
                 Mature                                                                                                                                                   Star
GAGGAAAAAAAUUUGUUGCAGUCCGGCUGUAAACCAGGCUGUUUGCAGCCCCUCACAAAAAGGAGGAUAGGUCCCACCCGCAGGUAAAUAAUACAACUAAAACGUUAUCUGGUGGACCUGCAGGUGGAGCCUAUCCUCCUUUUUGUGAGGGGCUGCAAACAGCCUGAUUUGCAGCCGGGCUGCAACAAAUUUUUUUCCGUCA     226.9
(((((((((((((((((((((((((((((((((.((((((((((((((((((((((((((((((((((((((.(((((.((((((..((.....(((......))).....))..)))))).))))).)))))))))))))))))))))))))))))))))))))).))))))))))))))))))))))))))))))).)).
.................GCAGUCCGGCUGUAAACCAGGC...................................................................................................................................................................     17.493841
..............GUUGCAGUCCGGCUGUAAACCAGG....................................................................................................................................................................     96.887062
...............UUGCAGUCCGGCUGUAAACCAGGC...................................................................................................................................................................     16.992802
.................GCAGUCCGGCUGUAAACCAGG....................................................................................................................................................................     0.152391
.................GCAGUCCGGCUGUAAACCAGGCUG................................................................................................................................................................     4.417284
.................GCAGUCCGGCUGUAAACCAGGAUG.................................................................................................................................................................     48.746967
..................CAGUCCGGCUGUAAACCAGGC...................................................................................................................................................................     0.660362
...................AGUCCGGCUGUAAACCAGGCU..................................................................................................................................................................     1.066201
..................................................................................................................................................................CCUGAUUUGCAGCCGGGCUGCAGC................     31.340555
```

zma-MIR_N84a

Chromosome: chr3
Start: 287458
End: 287566
Strand: -
Strand bias: 1.0
Abundance bias: 1.0
MFE: -30.10

```
                  Mature                                                Star
UGAUUCAGAUUCAGUUUCUGAUUUUGAAUCUGAUUCGGAUUCCAGAUCUGAUUUGGUUGAUUUUGAAUCUAAUUUGGGUUCUAAUUAGGAACGGAGAGUAGAGGAGUAC    172.5
..((((..((((.((((((((((..((((((((...((((((.(((((..........))))).))))))...)))))))).))))))))))...))))....))))..
..................UGAUUUUGAAUCUGAUUCGGAU.....................................................................    106.830742
...............UUCUGAUUUUGAAUCUGAUUCG........................................................................    1.72567
...............UUCUGAUUUUGAAUCUGAUUCGGA......................................................................    1.38899
................UCUGAUUUUGAAUCUGAUUCGGAU.....................................................................    0.499118
................UCUGAUUUUGAAUCUGAUUCGG.......................................................................    1.593905
.................CUGAUUUUGAAUCUGAUUCGGAUU....................................................................    10.138321
.................CUGAUUUUGAAUCUGAUUCGGAU.....................................................................    15.96925
.................CUGAUUUUGAAUCUGAUUCGGA......................................................................    5.42057
..................UGAUUUUGAAUCUGAUUCGGAUU....................................................................    11.998522
..................UGAUUUUGAAUCUGAUUCGGA......................................................................    1.35071
...................GAUUUUGAAUCUGAUUCGGAU.....................................................................    0.17779
...................GAUUUUGAAUCUGAUUCGGAUU....................................................................    0.84015
....................AUUUUGAAUCUGAUUCGGAUU....................................................................    7.80887
......................UUUGAAUCUGAUUCGGAUU....................................................................    6.816232
```

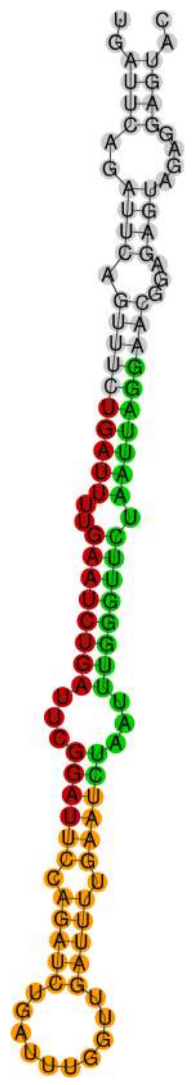

zma-MIR_N15b

Chromosome: chrB73V4_ctg134
Start: 32617
End: 32909
Strand: +
Strand bias: 1.0
Abundance bias:0.761
MFE: -56.80

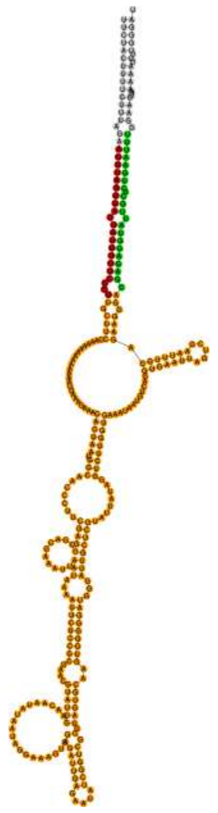

```
                                   Mature                                                                                                                                                                                                                                  Star
UUCUACUUUUGUUUAGAAUUUAGGCAUUAUUUUUCCCUUGCUUCCAAAAAAAAAAAAAUUAAAGAAACACUAAUGGCAACCCUUCGAGUCGACGAAAUUAAUAAAAUUCUCCGCGAACGUAUUGAACAAUAUAAUAGGAAAGUAGGGAUUGAGAAUAUCGGUCGCGUAGUGCAAGUGGGGAUGGGAUUGCUCGUAUUAUAGGUCUUGGUGAAAUAAUGUCAGGUGAAUUAGUCGAAUUUGCAGAAGGGACGAGAGGUAUUGCUCUGAAUUUGGAAUCUAAAAAUGUUGGGAU 9718.8
((((((((((((((.(((((((((((.((((((((..((.((((.......................((((((.(((.......(((((..........(((...(((((((((....((((((....................(.((((((.....)))))).).))))))..)))))))))...))))))))........)))))))))...........(((((((.....))))))).)))).)).)))))))).))).)))))))).))))...)))).).))))).
.................AUUUAGGCAUUAUUUUUCCCU............................................................................................................................................................................................................................................................... 3866.410815
..............AGAAUUUAGGCAUUAUUUUUCCCU............................................................................................................................................................................................................................................................... 0.796203
..............AGAAUUUAGGCAUUAUUUUUCCCUUG............................................................................................................................................................................................................................................................. 0.318481
..............AGAAUUUAGGCAUUAUUUUUCCCUU.............................................................................................................................................................................................................................................................. 0.318481
...............GAAUUUAGGCAUUAUUUUUCCCU............................................................................................................................................................................................................................................................... 10.791522
...............GAAUUUAGGCAUUAUUUUUCCCUUG............................................................................................................................................................................................................................................................. 4.48567
...............GAAUUUAGGCAUUAUUUUUCCCUU.............................................................................................................................................................................................................................................................. 43.9928
................AAUUUAGGCAUUAUUUUUCCCU............................................................................................................................................................................................................................................................... 68.734608
................AAUUUAGGCAUUAUUUUUCCCUUG............................................................................................................................................................................................................................................................. 2.31465
.................AUUUAGGCAUUAUUUUUCCCUUG............................................................................................................................................................................................................................................................. 231.317507
.................AUUUAGGCAUUAUUUUUCCCUU.............................................................................................................................................................................................................................................................. 556.954966
.................AUUUAGGCAUUAUUUUUCCC................................................................................................................................................................................................................................................................ 45.13044
.................AUUUAGGCAUUAUUUUUUCCU............................................................................................................................................................................................................................................................... 1.37654
.................AUUUAGGCAUUAUUUUUCUCU............................................................................................................................................................................................................................................................... 0.963578
.................AUUUAGGCAUUCUUUUUCCCU............................................................................................................................................................................................................................................................... 3.85431
.................AUUUAGGCAUUAUUUUUCCCC............................................................................................................................................................................................................................................................... 85.488197
.................AUUUAGGCAUUAUUUUUCCCA............................................................................................................................................................................................................................................................... 39.37336
.................AUUUAGGCAUUAUUUUUCCCG............................................................................................................................................................................................................................................................... 3.53534
.................CUUUAGGCAUUAUUUUUCCCU............................................................................................................................................................................................................................................................... 0.963578
..................UUUAGGCAUUAUUUUUCCCU............................................................................................................................................................................................................................................................... 469.603257
..................UUUAGGCAUUAUUUUUCCC................................................................................................................................................................................................................................................................ 3.2529
..................UUUAGGCAUUAUUUUUCCCUUG............................................................................................................................................................................................................................................................. 9.23024
..................UUUAGGCAUUAUUUUUCCCUU.............................................................................................................................................................................................................................................................. 61.31914
...................UUAGGCAUUAUUUUUCCCUUG............................................................................................................................................................................................................................................................. 6.73617
...................UUAGGCAUUAUUUUUCCCUU.............................................................................................................................................................................................................................................................. 8.253546
...................UUAGGCAUUAUUUUUCCCU............................................................................................................................................................................................................................................................... 257.689653
...................UUAGGCAUUAUUUUUCCC................................................................................................................................................................................................................................................................ 4.343382
....................UAGGCAUUAUUUUUCCCUUG............................................................................................................................................................................................................................................................. 112.822582
....................UAGGCAUUAUUUUUCCCU............................................................................................................................................................................................................................................................... 1187.251669
....................UAGGCAUUAUUUUUCCCUU.............................................................................................................................................................................................................................................................. 306.831434
```

zma-MIR_N15c

```
Chromosome:    chrPt
Start:         36502
End:           36793
Strand:        +
Strand bias:   1.0
Abundance bias:0.761
MFE:           -56.80
```

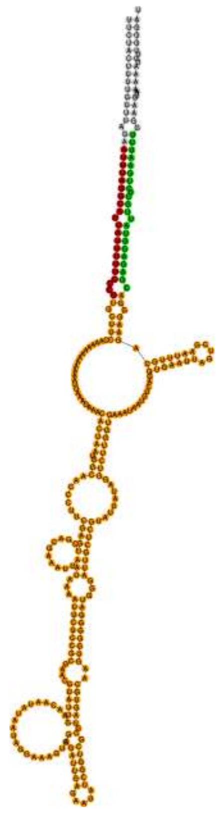

```
                 Mature                                                                                                                                                                                                                                                                      Star
UUCUACUUUUGUUUAGAAUUUAGGCAUUAUUUUUCCCUUGCUUCCAAAAAAAAAAAAAUUAAAGAAACACUAAUGGCAACCCUUCGAGUCGACGAAAUUAAUAAAAUUCUCCGCGAACGUAUUGAACAAUAUAAUAGGAAAGUAGGGAUUGAGAAUAUCGGUCGCGUAGUGCAAGUGGGGGAUGGGAUUGCUCGUAUUAUAGGUCUUGGUGAAAUAAUGUCAGGUGAAUUAGUCGAAUUUGCAGAAGGGACGAGAGGUAUUGCUCUGAAUUUGGAAUCUAAAAAUGUUGGGAU    9718.8
(((((((((((((.(((((((((((.((((((((..((.((((.....................((((((.(((.......(((((..........(((...(((((((((....((((((...................(.((((((.....)))))).)))))))..)))))))))...))))))))........)))))))))...........(((((((.....))))))).)))).)).))))))))).))).)))))))).))))...)))).).))))).
.................AUUUAGGCAUUAUUUUUCCCU...........................................................................................................................................................................................................................................................    3866.410815
..............AGAAUUUAGGCAUUAUUUUUCCCU...........................................................................................................................................................................................................................................................    0.796203
..............AGAAUUUAGGCAUUAUUUUUCCCUUG.........................................................................................................................................................................................................................................................    0.318481
..............AGAAUUUAGGCAUUAUUUUUCCCUU..........................................................................................................................................................................................................................................................    0.318481
...............GAAUUUAGGCAUUAUUUUUCCCU...........................................................................................................................................................................................................................................................    10.791522
...............GAAUUUAGGCAUUAUUUUUCCCUUG.........................................................................................................................................................................................................................................................    4.48567
...............GAAUUUAGGCAUUAUUUUUCCCUU..........................................................................................................................................................................................................................................................    43.9928
................AAUUUAGGCAUUAUUUUUCCCU...........................................................................................................................................................................................................................................................    68.734608
................AAUUUAGGCAUUAUUUUUCCCUUG.........................................................................................................................................................................................................................................................    2.31465
.................AUUUAGGCAUUAUUUUUCCCUUG.........................................................................................................................................................................................................................................................    231.317507
.................AUUUAGGCAUUAUUUUUCCCUU..........................................................................................................................................................................................................................................................    556.954966
.................AUUUAGGCAUUAUUUUUCCC............................................................................................................................................................................................................................................................    45.13044
.................AUUUAGGCAUUAUUUUUCCU............................................................................................................................................................................................................................................................    1.37654
.................AUUUAGGCAUUAUUUUUCUCU...........................................................................................................................................................................................................................................................    0.963578
.................AUUUAGGCAUUCUUUUUCCCU...........................................................................................................................................................................................................................................................    3.85431
.................AUUUAGGCAUUAUUUUUCCCC...........................................................................................................................................................................................................................................................    85.488197
.................AUUUAGGCAUUAUUUUUCCCA...........................................................................................................................................................................................................................................................    39.37336
.................AUUUAGGCAUUAUUUUUCCCG...........................................................................................................................................................................................................................................................    3.53534
.................CUUUAGGCAUUAUUUUUCCCU...........................................................................................................................................................................................................................................................    0.963578
..................UUUAGGCAUUAUUUUUCCCU...........................................................................................................................................................................................................................................................    469.603257
```
[truncated: 450,059 more chars]
