# Supplementary material for: Integrated Analysis of Large-Scale Omics Data Revealed Relationship Between Tissue Specificity and Evolutionary Dynamics of Small RNAs in Maize (Zea mays)
Source: Front Genet. 2020 Feb 11;11:51. doi: 10.3389/fgene.2020.00051 (PMC7026458; doi:10.3389/fgene.2020.00051)
Supplement: Supplementary file 16 [file Image_1.pdf]

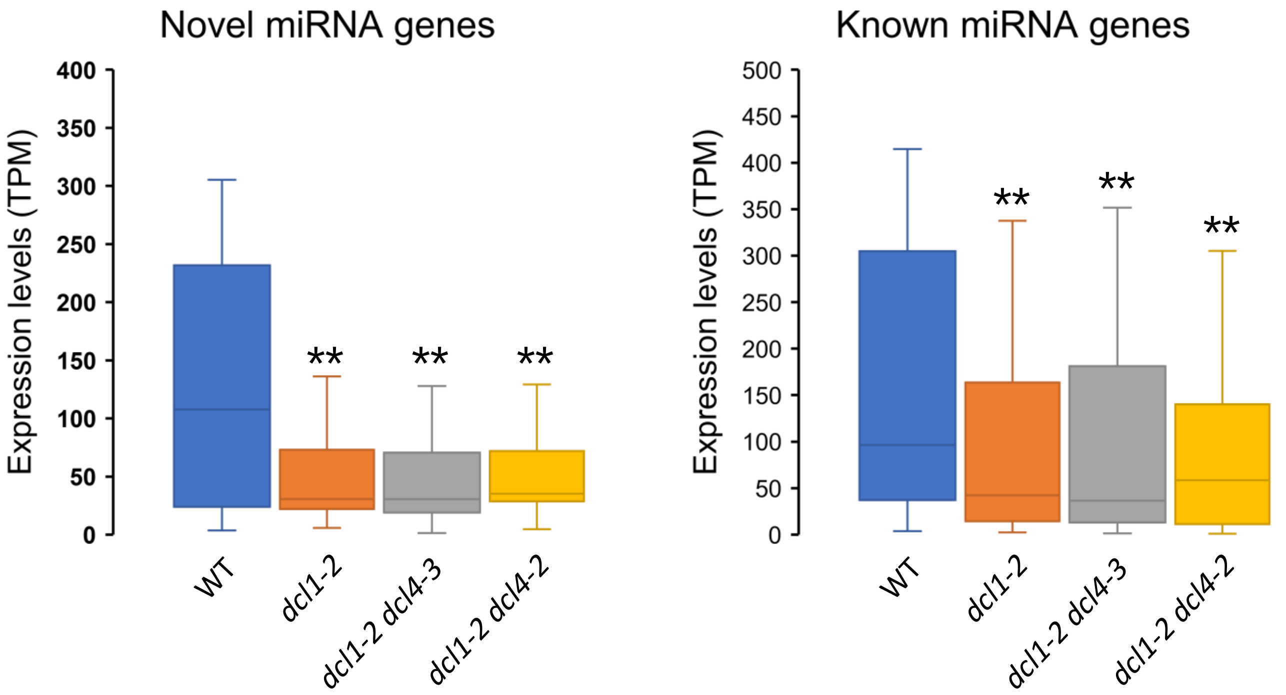

Supplementary Figure 1. Expression levels of miRNA genes were significantly reduced in *dcl1* mutants. Statistical analysis was conducted using the Student’s *t*-test. **, *P*-value < 0.001.
