# Supplementary figures and images for: Integrated Analysis of Large-Scale Omics Data Revealed Relationship Between Tissue Specificity and Evolutionary Dynamics of Small RNAs in Maize (Zea mays)
Source: Front Genet. 2020 Feb 11;11:51. doi: 10.3389/fgene.2020.00051 (PMC7026458; doi:10.3389/fgene.2020.00051)

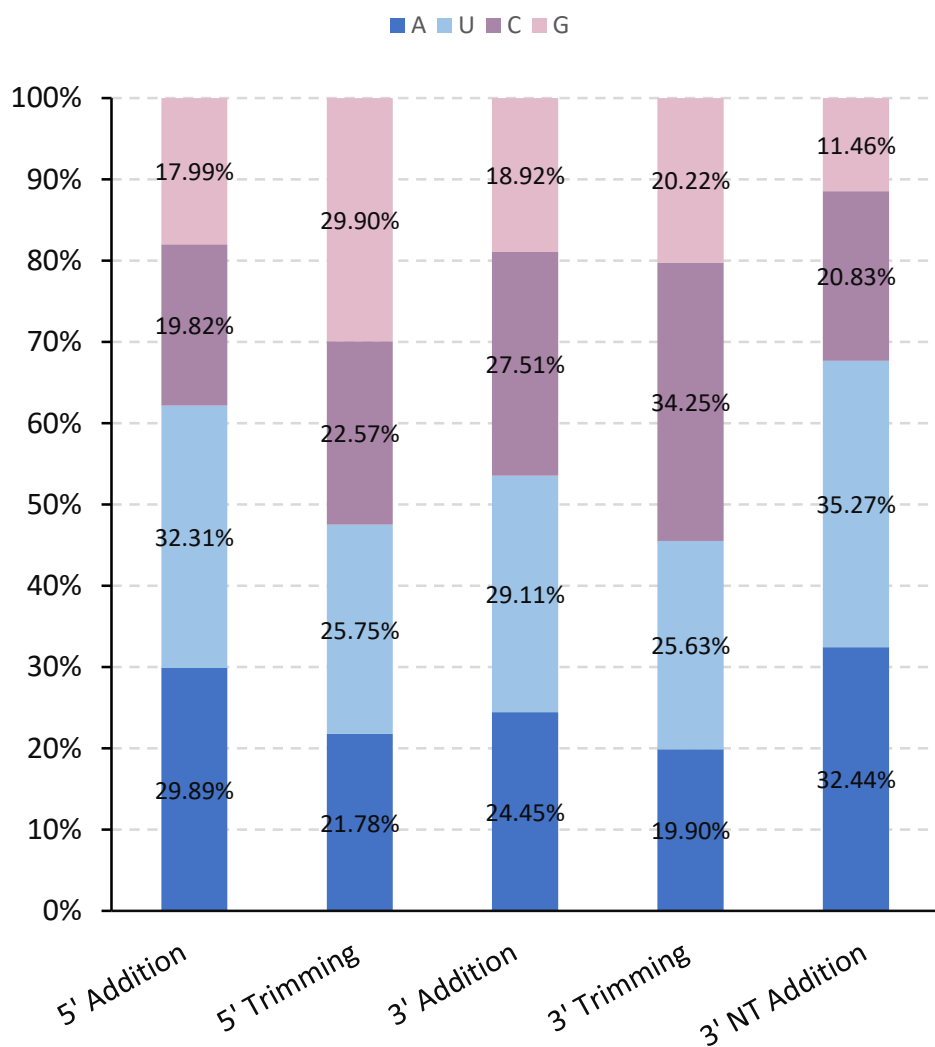

**Supplementary Figure 2.** Structure distribution of terminal nucleotides of isomiRs in maize.

Supplement: Supplementary file 17 [file Image_2.pdf]

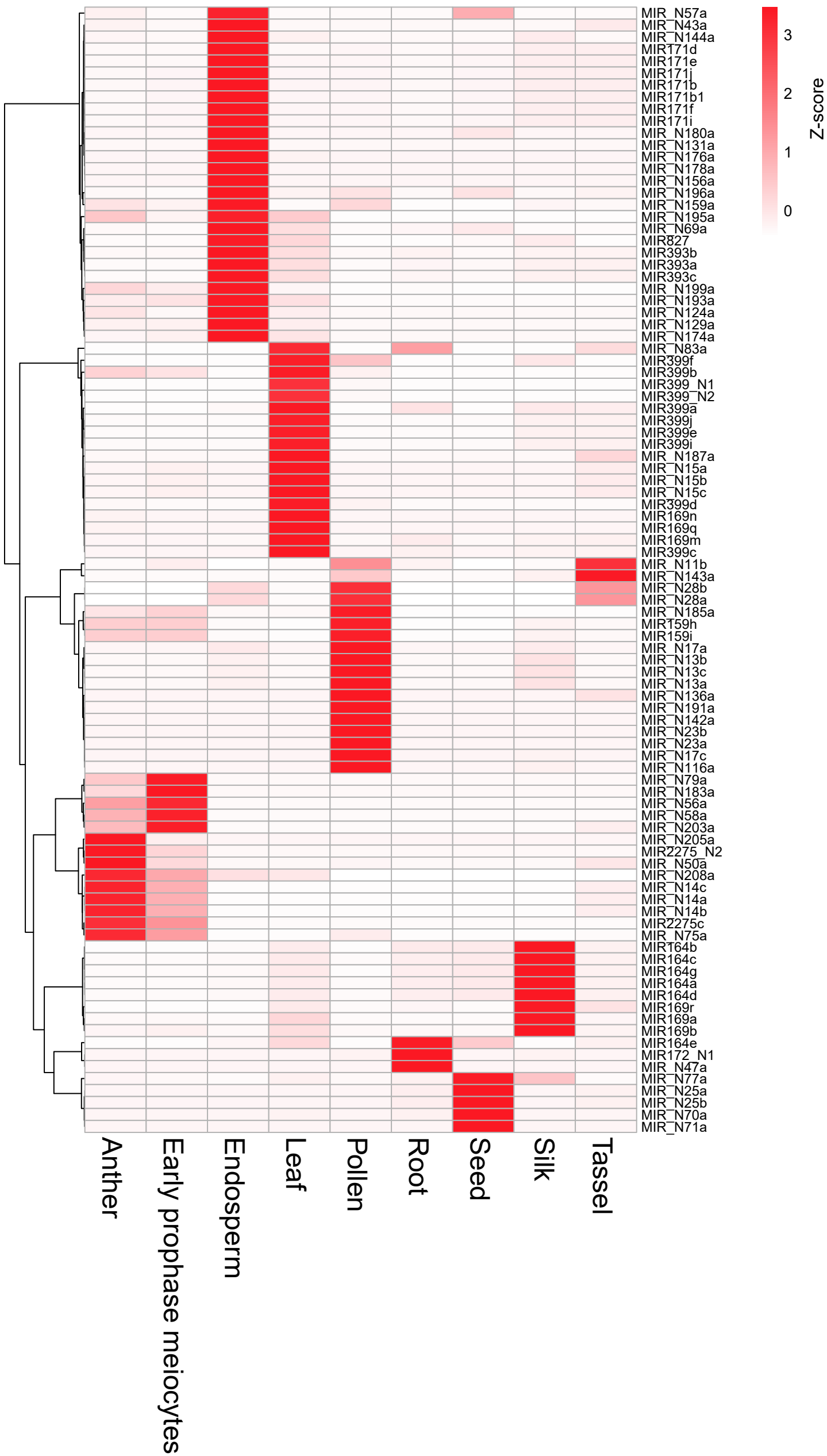

Supplementary Figure 3. Heatmap showing the expression of tissue-specific miRNA genes.

Supplement: Supplementary file 18 [file Image_3.pdf]

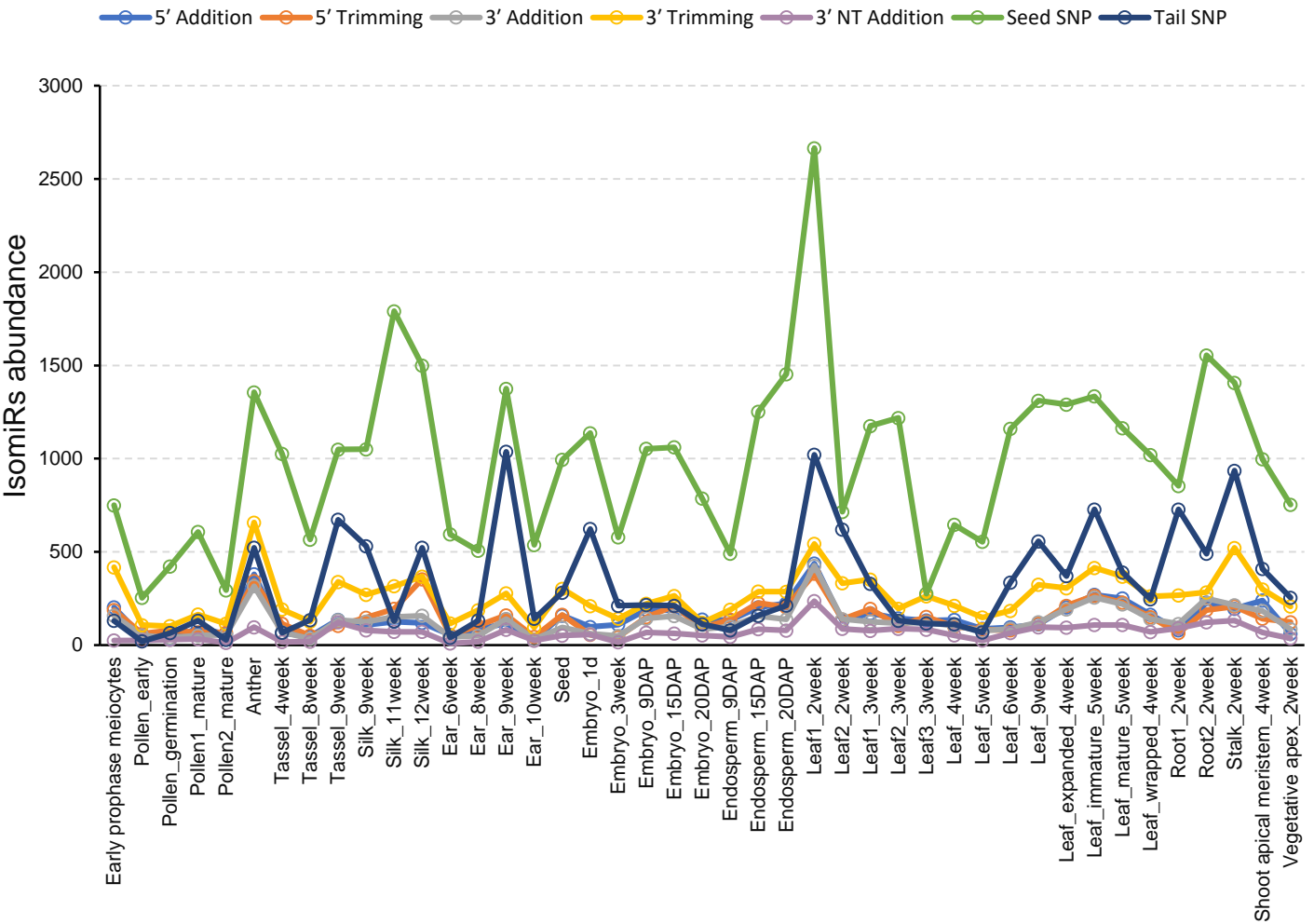

**Supplementary Figure 5.** Distribution of maize isomiR abundance in individual tissue samples.

Supplement: Supplementary file 20 [file Image_5.pdf]
