# Supplementary material for: Integrated Analysis of Large-Scale Omics Data Revealed Relationship Between Tissue Specificity and Evolutionary Dynamics of Small RNAs in Maize (Zea mays)
Source: Front Genet. 2020 Feb 11;11:51. doi: 10.3389/fgene.2020.00051 (PMC7026458; doi:10.3389/fgene.2020.00051)
Supplement: Supplementary file 19 [file Image_4.pdf]

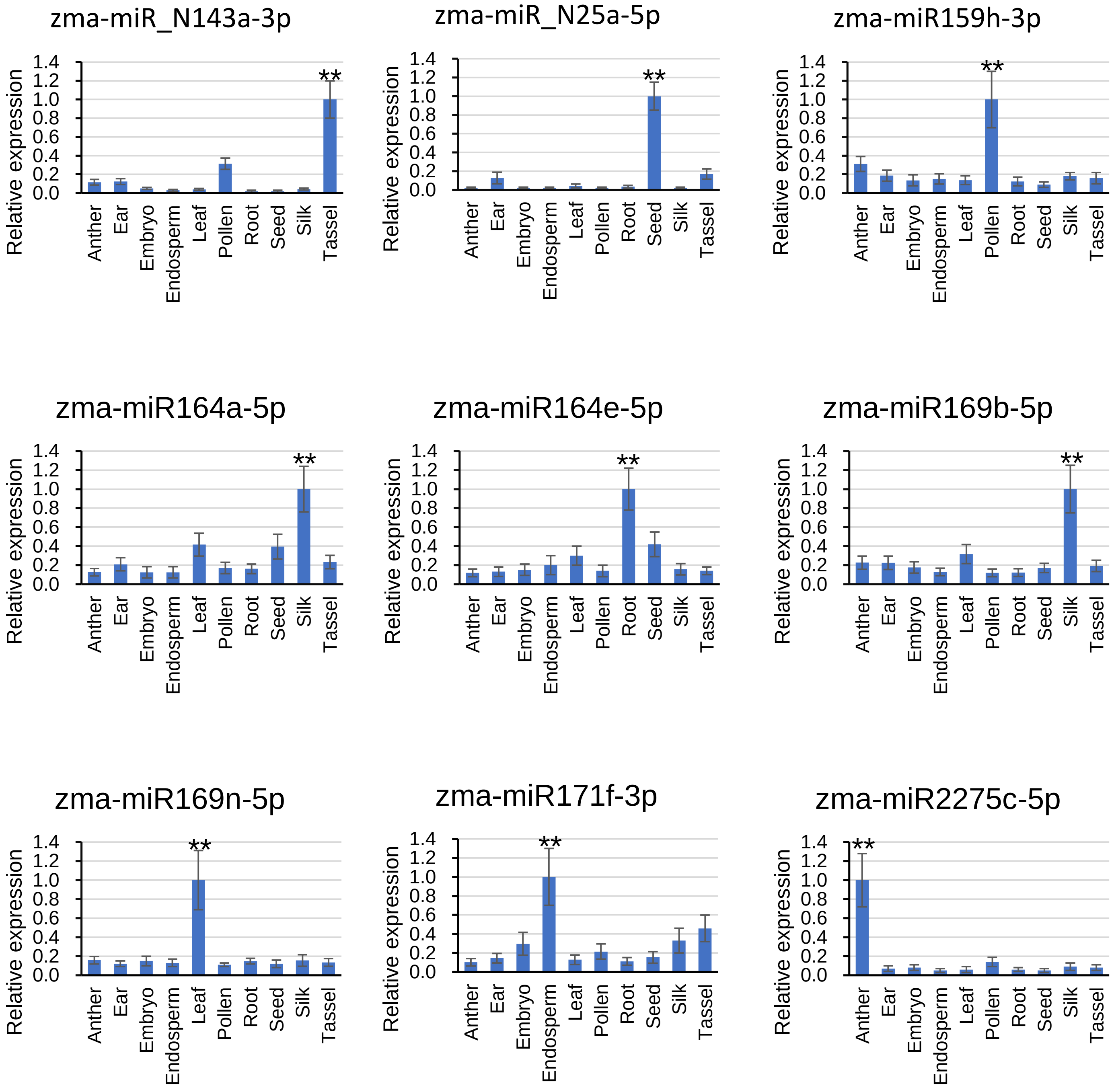

Supplementary Figure 4. Relative expression patterns of nine tissue-specific miRNAs were examined using stem-loop qRT-PCR. Data are means ± SE; **, *P*<0.001 by Student's *t*-test.
