# Supplementary material for: Integrated Analysis of Large-Scale Omics Data Revealed Relationship Between Tissue Specificity and Evolutionary Dynamics of Small RNAs in Maize (Zea mays)
Source: Front Genet. 2020 Feb 11;11:51. doi: 10.3389/fgene.2020.00051 (PMC7026458; doi:10.3389/fgene.2020.00051)
Supplement: Supplementary file 22 [file Image_7.pdf]

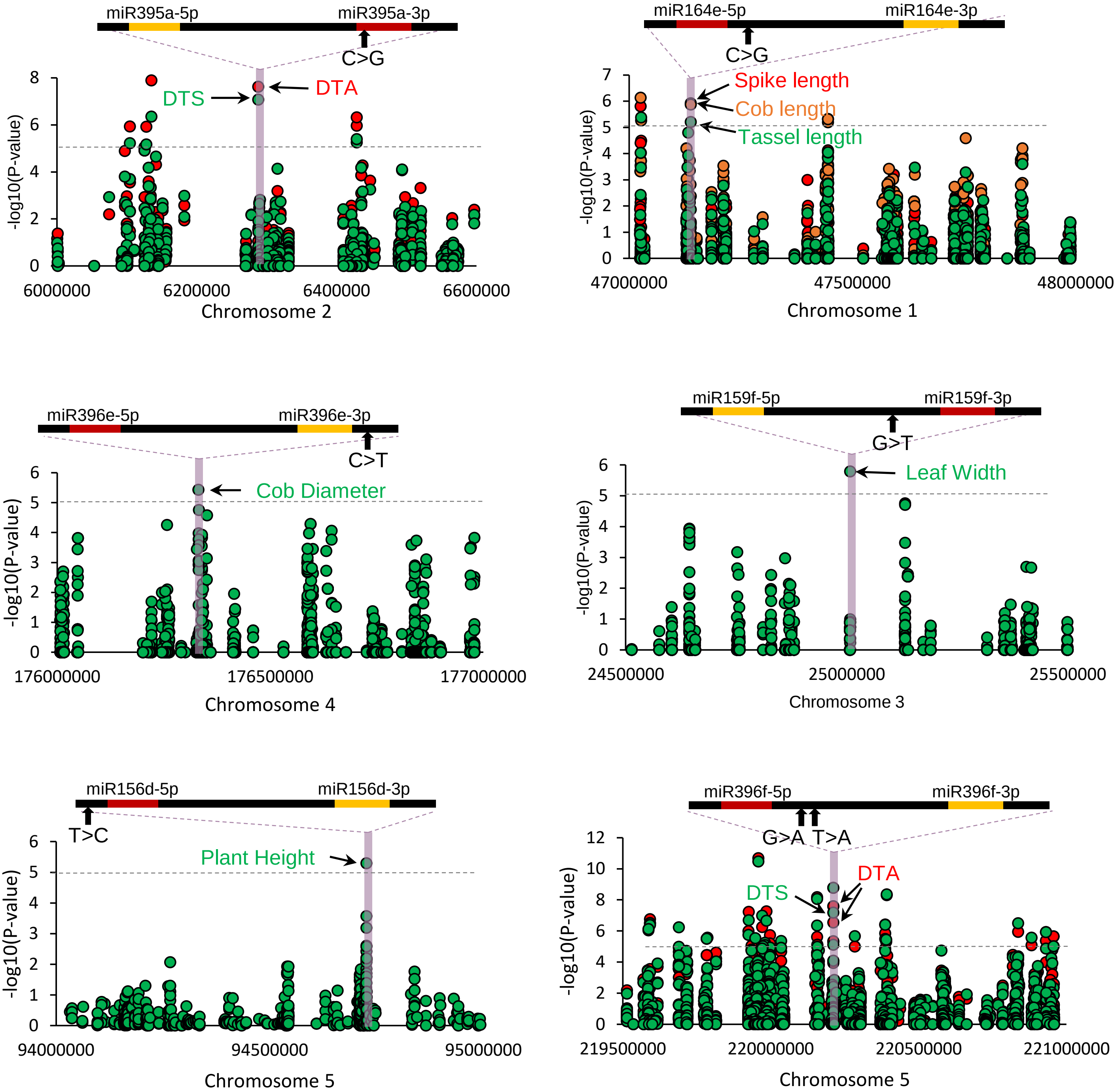

Supplementary Figure 7. Results of GWAS that overlapped with miRNA loci.
The gray horizontal dashed lines indicate the significance threshold of GWAS (1×10^{-5}). The mature miRNA sequences were highlighted in red and the miRNA* sequences were highlighted in yellow. Black arrows indicate significant mutation sites. Selected traits were labeled in red, green, and orange respectively.
